# Chiral π–Cu(ii)-catalyzed site-, *exo*/*endo*-, and enantioselective dearomative (3 + 2) cycloadditions of isoquinolinium ylides with enamides, dienamides, and a trienamide†

**DOI:** 10.1039/d4sc02946a

**Published:** 2024-06-11

**Authors:** Weiwei Guo, Jianhao Huang, Kazuaki Ishihara

**Affiliations:** a Graduate School of Engineering, Nagoya University B2-3(611), Furo-cho, Chikusa Nagoya 464-8603 Japan ishihara@cc.nagoya-u.ac.jp

## Abstract

Here, we report a highly effective dearomative (3 + 2) cycloaddition reaction between isoquinolinium ylides and α,β-enamides, α,β–γ,δ-dienamides, or an α,β–γ,δ–ε,ζ-trienamide, which is catalyzed by a chiral π–Cu(ii) complex (1–10 mol%) and proceeds in a site-selective, *exo*/*endo*-selective, and enantioselective manner. The (3 + 2) cycloaddition involving the α,β-enamides proceeds with high *exo*-selectivity and enantioselectivity. This method is applicable to various substrates including α-substituted, α,β-disubstituted, or β,β-disubstituted α,β-enamides, which are compounds with an intrinsically low reactivity. This method provides synthetic access to pyrroloisoquinoline derivatives with up to three chiral carbon centers, including those featuring fluorine and trifluoromethyl groups, as well as quaternary carbon centers. The (3 + 2) cycloaddition involving α,β–γ,δ-dienamides proceeds with high γ,δ-site-selectivity and enantioselectivity, whereby the *exo*/*endo*-selectivity depends on the substrates and ligands. Remarkably, the (3 + 2) cycloaddition of δ-phenyl-α,β–γ,δ-dienamide proceeds with high α,β-site-selectivity, *exo*-selectivity, and enantioselectivity. In a manner similar to the reaction with the α,β–γ,δ-dienamides, α,β–γ,δ–ε,ζ-trienamide furnishes a (3 + 2) cycloadduct with good ε,ζ-site-selectivity, *endo*-selectivity, and enantioselectivity.

## Abstract 2

We report a dearomative (3 + 2) cycloaddition between isoquinolinium ylides and enamides, dienamides, or a trienamide, which is catalyzed by a chiral π–Cu(ii) complex and proceeds in a site-selective, *exo*/*endo*-selective, and enantioselective manner.

## Graphical abstract

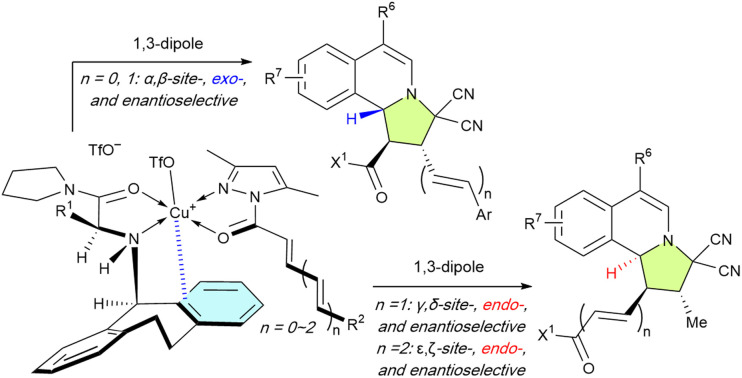

## Introduction

**Scheme 1:**
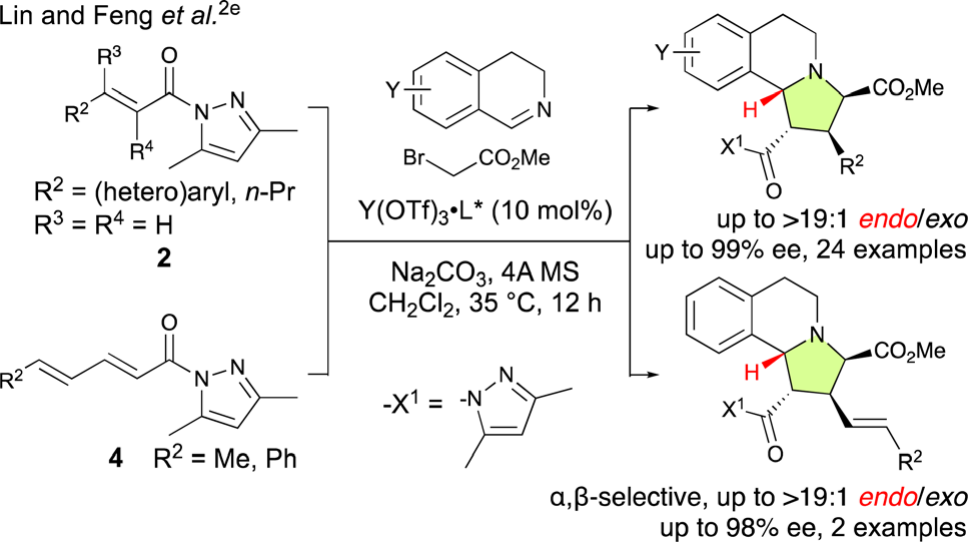
Catalytic α,β-site-selective, *endo*-selective, and enantioselective (3 + 2) cycloadditions of *in situ*-generated 2-(2-methoxy-2-oxoethyl)-3,4-dihydroisoquinolin-2-ium ylides starting from 2 and 4 as reported by Lin and Feng *et al.*^2*e*^

**Scheme 2:**
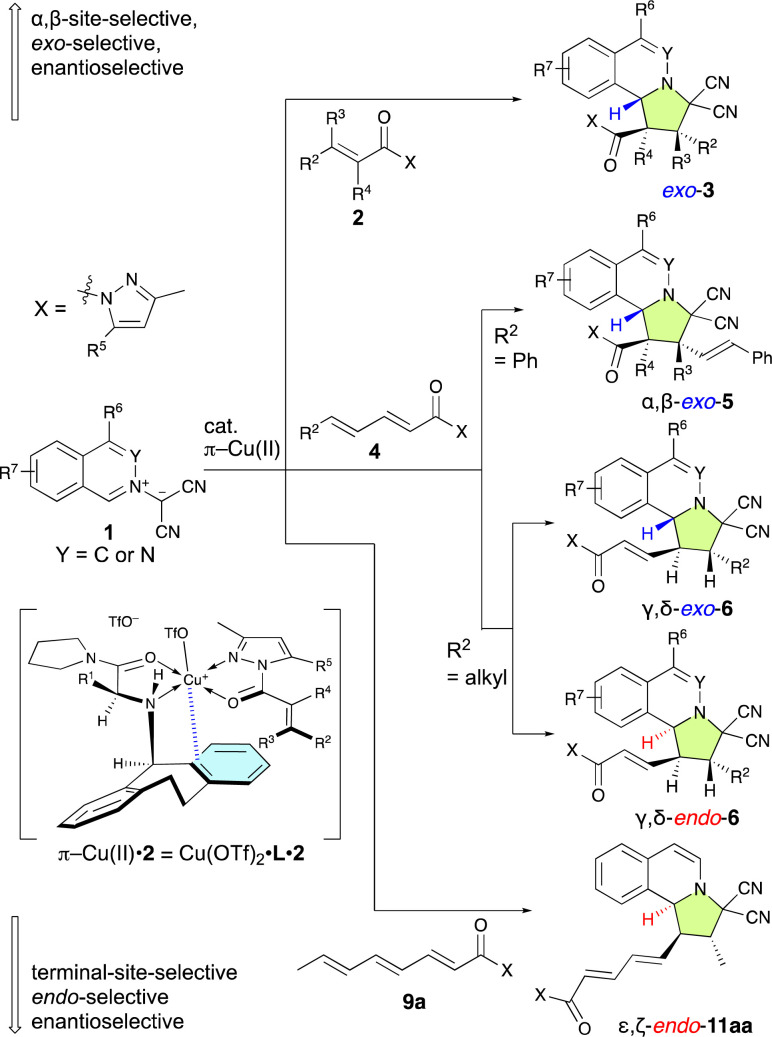
π–Cu(ii)-complex-catalyzed multiselective (3 + 2) cycloaddition reactions of 1 with 2, 4, and 9a reported in this work.

Catalytic enantioselective (3 + 2) cycloaddition reactions have emerged as important synthetic methods that facilitate the precise and enantioselective construction of numerous valuable heterocyclic motifs.^1^ In these reactions, acryloyl derivatives such as 2 are typically employed as dipolarophiles (Schemes 1 and 2). However, the use of these acryloyl derivatives is primarily restricted to β-substituted acryloyl derivatives, whilst α-substituted, α,β-disubstituted or β,β-disubstituted derivatives are rarely used due to their intrinsically low reactivity, thus significantly limiting the versatility and scope of these reactions.^1*d*^ If the substrate scope of enantioselective (3 + 2) cycloaddition reactions could be expanded to include acryloyl derivatives with more complex substitution patterns, the synthetic utility of these reactions would be significantly enhanced, thus enabling the construction of valuable heterocyclic moieties with all-carbon-substituted quaternary stereocenters bearing alkyl or fluoroalkyl groups.^2,3^ At the same time, enhancing other selectivity such as site-, chemo-, *exo*/*endo*-, or regioselectivity would improve the overall applicability of these transformative methods.

The pursuit of γ,δ-site-selective and enantioselective (3 + 2) cycloaddition reactions of α,β–γ,δ-dienamides (4) is highly attractive because it not only allows for the establishment of remote chiral carbon centers but also retains the α,β-unsaturation of the starting material, therefore enabling further activation and selective functionalization of the resulting products. However, achieving this goal is remarkably challenging because the largest coefficient of the lowest unoccupied molecular orbital (LUMO) is located at the β-position, which favors β-addition, and controlling the stereochemistry also presents significant difficulties as the δ-position is quite removed from the active site of the catalyst.^4*a*–*h*,*n*^ For example, Lin and Feng *et al.* have reported an enantioselective (3 + 2) cycloaddition of the *in situ*-generated 2-(2-methoxy-2-oxoethyl)-3,4-dihydroisoquinolin-2-ium ylides with α,β-enamides (2) and α,β–γ,δ-dienamides (4) catalyzed by a chiral *N*,*N*′-dioxide-Y(OTf)_3_ complex that is both α,β-site-selective and *endo*-selective (Scheme 1).^2*e*^

Despite the challenges associated with these reactions, some elegant strategies have already been designed that allow remote and enantioselective 1,6-additions.^4^ However, the methods for remote and enantioselective (3 + 2) cycloaddition reactions remain relatively underdeveloped. A breakthrough was made by the Jørgensen group in 2016 when they reported the first γ,δ-site-selective, *exo*-selective, and enantioselective (3 + 2) cycloadditions of nitrones with α,β–γ,δ-dienals *via* a vinylogous iminium-ion activation mode where 20 mol% of a chiral diarylprolinol silyl ether catalyst was employed.^5^ In 2020, a similar strategy was applied by Zhang and Guo *et al.* to achieve γ,δ-site-selective, *endo*-selective, and enantioselective (3 + 2) cycloadditions of phthalazinium dicyanomethanides with α,β–γ,δ-dienals using 20 mol% of MacMillan's catalyst.^6^ In general, the turnover frequency (TOF) of secondary-amine catalysts is relatively inefficient because a dehydrative condensation step between the amine catalyst and the aldehyde and a hydrolysis step are included in the catalytic cycle.

Our previously developed chiral π–Cu(ii) complexes have demonstrated exceptionally strong capabilities as Lewis-acidic catalysts in several highly useful asymmetric transformations.^7^ We envisioned that such a highly effective catalyst system could potentially accomplish several highly important functions: (1) promote *exo*/*endo*-selective and enantioselective (3 + 2) cycloadditions between isoquinolinium ylides (1) and α-substituted, α,β-disubstituted, or β,β-disubstituted α,β-enamides (2) to conveniently provide pyrroloisoquinoline derivatives (3); (2) promote challenging highly α,β/γ,δ-site-selective, *exo*/*endo*-selective, and enantioselective (3 + 2) cycloadditions between 1 and α,β–γ,δ-dienamides (4); and (3) promote challenging highly ε,ζ-site-selective, *endo*-selective, and enantioselective (3 + 2) cycloadditions between 1 and α,β-γ,δ–ε,ζ-trienamide 9a (Scheme 2). If successful, the remarkable versatility of this catalytic system would significantly broaden its practical utility. Here, we report on the site-selective, chemo-selective, *exo*/*endo*-selective, and enantioselective dearomative (3 + 2) cycloaddition reactions of 1 with 2, 4, and 9a catalyzed by a chiral π–Cu(ii) complex. These reactions proceed in the presence of 1–10 mol% of the π–Cu(ii) complexes to give *exo*-adducts 3, α,β-*exo*-adducts 5, γ,δ-*exo*/*endo*-adducts 6, and ε,ζ-*endo*-adduct 11aa with in a multi-selective manner under the optimized conditions developed in this study (Scheme 2).

## Results and discussion

### Chiral π–Cu(ii)-complex-catalyzed site-selective, *exo*/*endo*-selective and enantioselective dearomative (3 + 2) cycloaddition reactions between isoquinolinium ylides and α,β-enamides

In recent years, bench-stable isoquinolinium ylides (1) have garnered significant attention in synthetic organic chemistry due to their efficiency in the construction of pyrroloisoquinoline scaffolds, which are crucial structural motifs in various natural products and pharmaceutically active compounds.^8^ However, only a few examples for their use as dipolar compounds in dearomative (3 + 2) cycloaddition reactions have been reported so far.^7*l*,8*e*^ Therefore, the development of a catalytic enantioselective (3 + 2) cycloaddition reaction of 1 with α,β-enamides (2) for the construction of pyrroloisoquinoline derivatives (3) would be of considerable significance. As shown in Table 1, in our initial attempt, the enantioselective (3 + 2) cycloaddition reaction of 1a with 2a was conducted in the presence of 10 mol% of a chiral π–Cu(ii) complex of Cu(OTf)_2_ with l-alanine-derivatized ligand L in dichloromethane at −10 °C for 24 h. To our delight, when *N*-cyclopentyl-β-(2-naphthyl)-l-alanine amide (L1) was employed as the ligand, the reaction smoothly provided 3aa in high yield, albeit with poor enantioselectivity (Table 1, entry 1; 92%, −20% ee). Interestingly, the diastereoselectivity toward 3aa was 97% *exo*. The use of *N*-cyclobutyl-based L2 instead of *N*-cyclopentyl-based L1 did not improve the enantioselectivity (Table 1, entry 2). However, the use of *N*-dibenzosuberyl (Dbs)-based L3 significantly increased the ee to +88% with >99% *exo*-selectivity (Table 1, entry 3). This observed switch in the enantioselectivity can be interpreted in terms of a switch of the π–Cu(ii) interaction, which creates the asymmetric environment, from π(naphthyl)–Cu(ii) to π(Dbs)–Cu(ii). Inspired by these results, we investigated several other 3-substituted *N*-Dbs-alanine amides as chiral ligands. When l-leucine-derived L4 was employed, the reaction afforded 3aa in 97% yield with +94% ee and >99% *exo*-selectivity (Table 1, entry 4). When β-*tert*-butyl-l-alanine-derived ligand L5 was used, *exo*-3aa was obtained in a 98% yield with +98% ee (Table 1, entry 5).

**Table 1:** Optimizations of the ligand for the *exo*-selective and enantioselective (3 + 2) cycloaddition reaction of 1a with 2a^a^

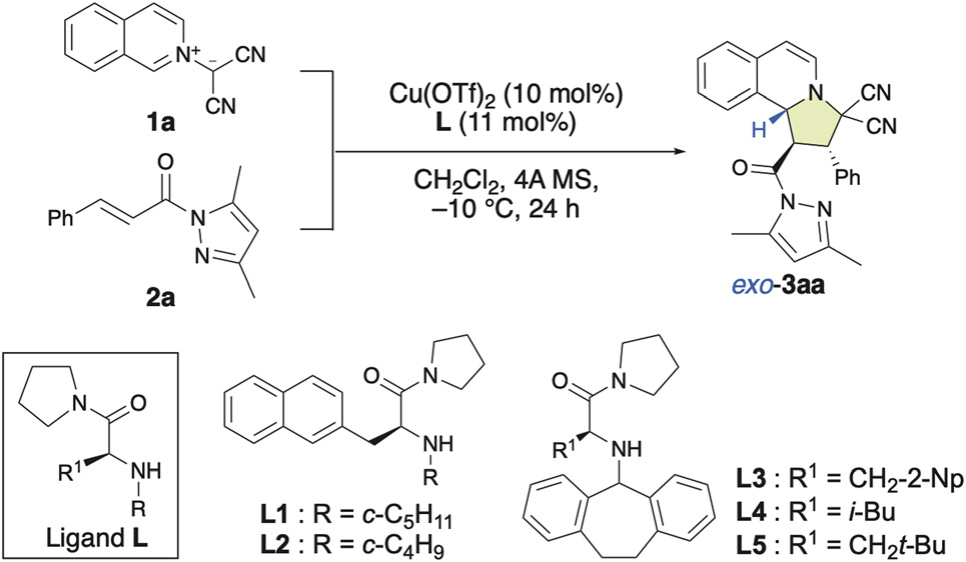				
Entry	Ligand	3aa		
		Yield/%	Ee/%	*Exo* : *endo*
1	(*S*)-L1	92	−20	97 : 3
2	(*S*)-L2	96	−17	98 : 2
3	(*S*)-L3	93	+88	>99 : 1
4	(*S*)-L4	97	+94	>99 : 1
5	(*S*)-L5	98	+98	>99 : 1

a Reaction conditions: 1a (0.22 mmol), 2a (0.20 mmol), Cu(OTf)_2_ (10 mol%), L (11 mol%), and 4A MS (150–200 mg) in dichloromethane (1.3 mL) at −10 °C for 24 h. Isolated yields are given. Enantiomeric-excess (ee) values were determined using HPLC. The *exo* : *endo* ratio was determined based on a ^1^H NMR analysis of the crude product.

**Fig. 1:**
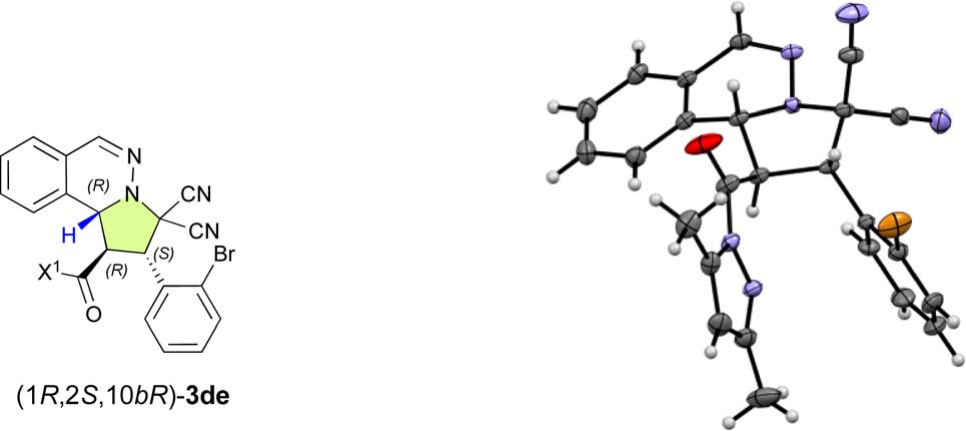
Molecular structure of (1*R*,2*S*,10*bR*)-3de in the single crystal.^10^

With the optimal ligand L5 in hand, we then focused on investigating the substrate scope of the catalytic enantioselective (3 + 2) cycloaddition reaction between 1 and 2. As shown in Table 2, a series of β-(hetero)aryl-α,β-enamides (2a–2h) were examined first. *Ortho*-, *meta*-, or *para*-substituted β-phenyl-α,β-enamides (2a–2g) and β-(3-thiophenyl)-α,β-enamide 2h are suitable for this reaction and afforded adducts 3aa–3ah in high yield with excellent *exo*- and enantioselectivities. It is noteworthy that the chemoselective, regioselective, and enantioselective (3 + 2) cycloaddition reaction of 1a with β-[*p*-((*E*)-3-oxobut-1-en-1-yl)phenyl]-α,β-enamide 1g gave the desired product (3ag) in 95% yield with 99% ee without any competitive α′,β′-cycloadducts. When bromoisoquinolinium ylides 1b and 1c were employed, the (3 + 2) cycloaddition efficiently furnished the desired products 3ba and 3ca in excellent yield as pure enantiomers. Additionally, the dipolar phthalazinium ylide^9^2d was also tolerated, and the desired products were obtained in excellent yield and ee (3da: 98%, >99% ee; 3de: 97%, >99% ee). The relative and absolute configuration of 3de was determined to be *exo*-(1*R*,2*S*,10*bR*) based on a single-crystal X-ray diffraction analysis, and the configuration of all other products were assigned accordingly (Fig. 1).^10^

**Table 2:** *Exo*-selective and enantioselective (3 + 2) cycloaddition reaction of 1 with β-substituted or β,β-disubstituted α,β-enamides (2)^a^

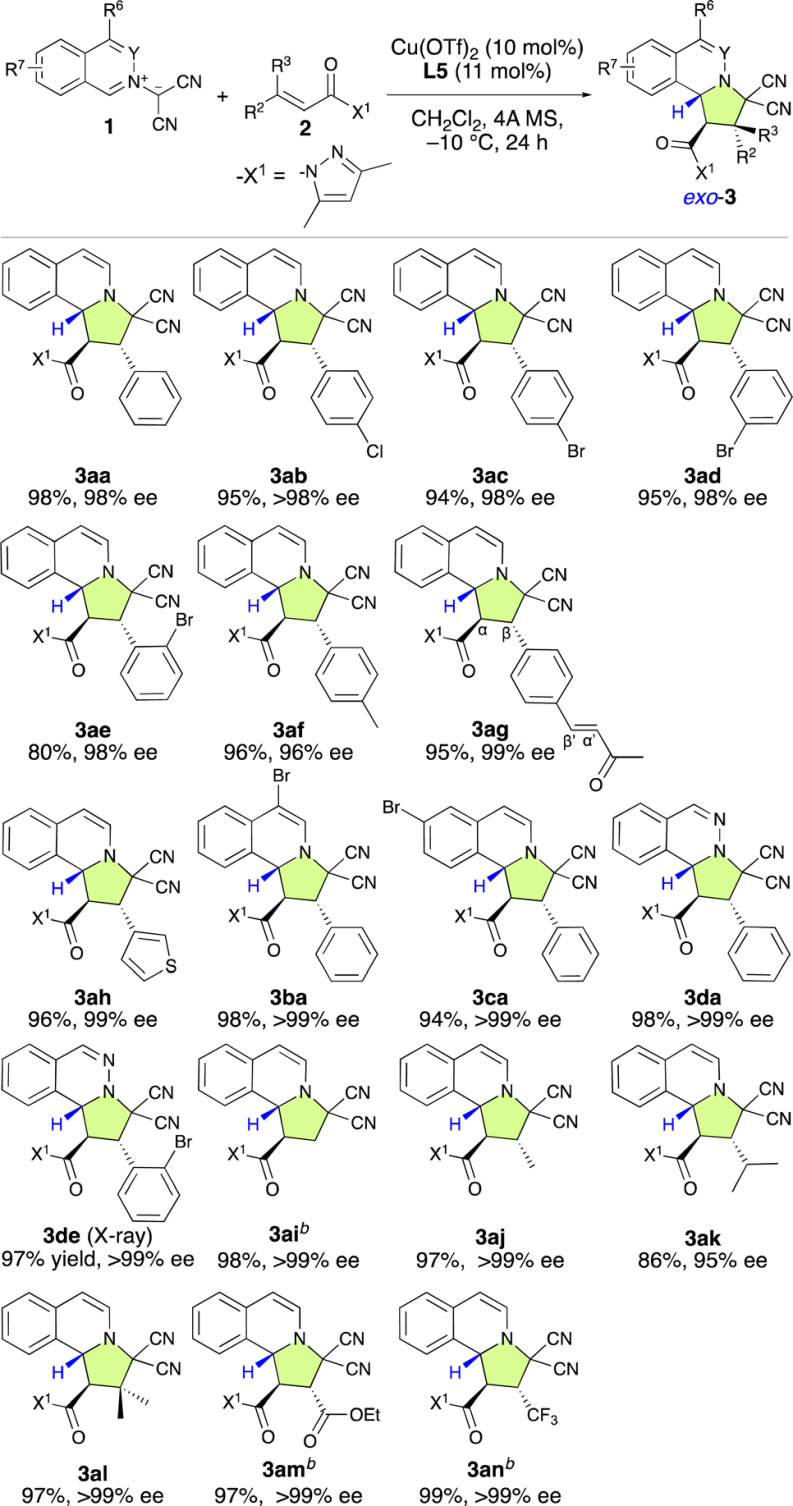

a Unless otherwise noted, the following reaction conditions were applied: 1 (0.2 mmol), 2 (0.22 mmol), Cu(OTf)_2_ (10 mol%), L5 (11 mol%), and 4A MS (150–200 mg) in dichloromethane (1.3 mL) at −10 °C for 24 h. Isolated yields are given. Enantiomeric-excess (ee) values were determined using HPLC. In all the cases, the *exo*-selectivity was >98%.

b −40 °C, 6 h.

Then, we turned to explore the applicability of β-nonaromatic-group-substituted α,β-enamides (2i–2n). α,β-Enamide 2i exhibited high reactivity and efficiency, furnishing the desired product (3ai) in 98% yield with >99% ee after 6 h at −40 °C (Table 2). The β-methyl- and β-isopropyl-α,β-enamides 2j and 2k were also reactive enough to furnish products 3aj and 3ak in 97% and 86% yields with >99% and 95% ee, respectively. Surprisingly, the reaction of 1a with β,β-dimethyl-α,β-enamide 2l smoothly furnished 3la, which bears quaternary carbon center, in 97% yield as enantiopure form. To our delight, the (3 + 2) cycloaddition with β-ethoxycarbonyl-α,β-enamide 2m proceeded very rapidly and 3am was furnished in 97% yield with >99% ee without an ester-group-induced regioisomeric product. The incorporation of the trifluoromethyl group into organic molecules has garnered considerable attention due to the unique properties it induces and the important applications of CF_3_-substituted compounds in the pharmaceutical industry.^11^ Furthermore, the enantioselective (3 + 2) cycloaddition of prochiral CF_3_-substituted substrates has become a popular method in recent years to build chiral CF_3_-containing pyrrolidine derivatives.^3^ Thus, we examined the use of prochiral β-trifluoromethyl-α,β-enamide 2n as a dipolarophile. To our delight, the reaction was exceptionally efficient, furnishing the desired product (3an) in 99% yield with >99% ee.

**Scheme 3:**
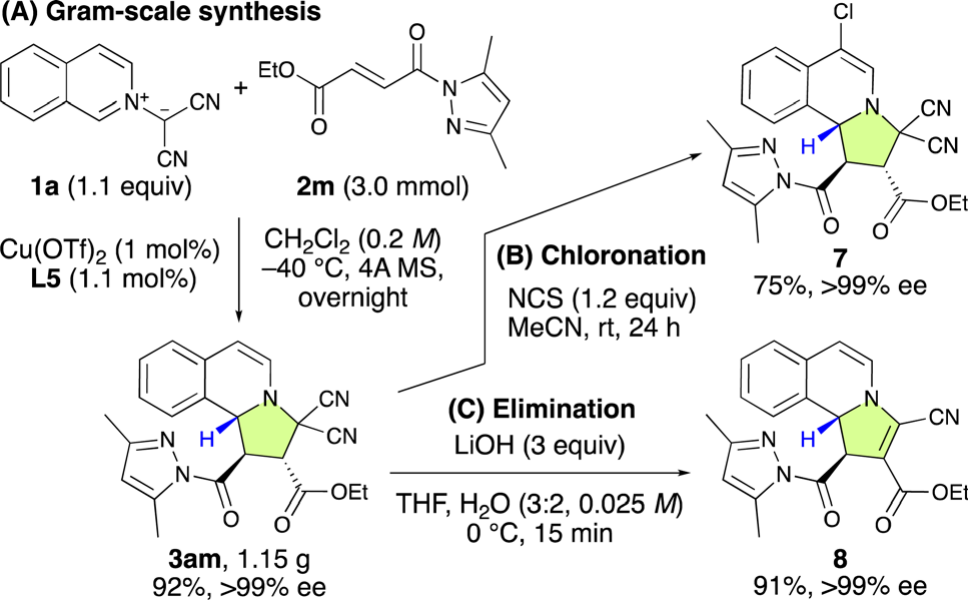
Gram-scale synthesis of 3am and its subsequent transformations.

To show the synthetic applicability of the (3 + 2) cycloaddition reaction between 1a and 2m, a gram-scale synthesis was undertaken (Scheme 3A). Even when employing a lower catalyst loading (1 mol%) of L5, the reaction efficiently yielded 3am in 92% yield with >99% ee. Subsequent transformations of 3am, including chlorination and elimination, were carried out successfully without any loss of enantioselectivity (Scheme 3B and C).

Based on these findings, our attention shifted to investigating the introduction of a chiral quaternary carbon center into the pyrroloisoquinoline structure using the enantioselective (3 + 2) cycloaddition of 1a with α-substituted α,β-enamides (2o–2s) (Table 3). Our initial attempt involved using a dipolarophile 2o, which contains a 3,5-dimethylpyrazolyl group (X^1^). However, even after 24 h, almost no reaction occurred, and 2o was recovered. Encouragingly, when we used 2p, which bears a monomethyl-substituted pyrazolyl group (X^2^), the reaction efficiently furnished the desired product (3ap) in 97% yield with 99% ee, resulting in a chiral quaternary carbon center at the α-position. The first result was attributed to the steric repulsion between the 5-Me group in X^1^ and the α-substituent of 2o, which disfavors activation by the chiral π–Cu(ii) complex. Consequently, rotation can be expected to occur, and the inherently low reactivity of this substrate results in almost no reaction. However, when X^2^ was employed in place of X^1^, the steric hindrance between the 5 H group of the X^2^ moiety and the α-substituent of 2p decreased significantly. Consequently, 2p is readily activated by the chiral π–Cu(ii) catalyst, which enables an efficient reaction with 1a. Furthermore, as the configuration of activated 2p is fixed by the chiral π–Cu(ii) catalyst, the enantioselectivity is very high. Subsequently, using X^2^ instead of X^1^, α,β-dimethyl-α,β-enamide 2q was examined, and the desired product (3aq) was obtained in a 93% yield with 99% ee. However, the reaction of α-methyl-α,β-enamide 2r with 1a gave 3ar in a relatively low ee value of 89%. Inspiringly, α-fluoro-α,β-enamide 2s was also well tolerated under the applied conditions. This led to the formation of the expected product (3as), which features a chiral quaternary carbon center that contains a fluoro group, in a 97% yield with >99% ee.

**Table 3:** *Exo*-selective and enantioselective (3 + 2) cycloaddition reaction between 1a and α-substituted α,β-enamides 2^a^

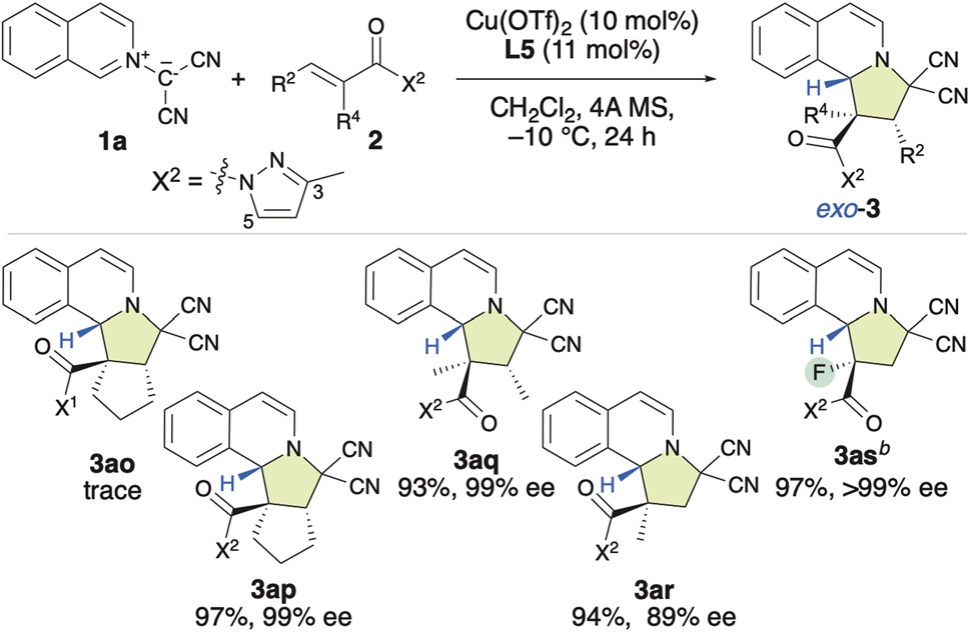

a Unless otherwise noted the following reaction conditions were applied: 2 (0.2 mmol), 1 (0.24 mmol), Cu(OTf)_2_ (10 mol%), L5 (11 mol%), and 4A MS (150–200 mg) in dichloromethane (1.3 mL) at −10 °C for 24 h. Isolated yields are given. Enantiomeric-excess (ee) values were determined using HPLC. In all the cases where the product was obtained, the *exo*-selectivity was >98%.

b −40 °C, overnight.

### Chiral π–Cu(ii)-complex-catalyzed site-selective, *exo*/*endo*-selective and enantioselective dearomative (3 + 2) cycloaddition between isoquinolinium ylides and α,β–γ,δ-dienamides

Generally, to facilitate a 1,6-addition, it is essential to generate a catalytic intermediate that enhances the electropositivity of the δ-position. The limited number of approaches known for LUMO-lowering activation are achieved through the formation of a vinylogous iminium ion using organoamine catalysts with α,β–γ,δ-unsaturated aldehydes or ketones.^4*e*^ Thus, we focused on the site-, *exo*/*endo*- and enantioselective (3 + 2) cycloaddition reaction between 1b and α,β,γ,δ-dienamide 4a (Table 4). Surprisingly, the (3 + 2) cycloaddition reaction proceeded slowly, even in the absence of Cu(OTf)_2_·L, at room temperature and exhibited both *endo*- and site-selectivity for the remote γ,δ-double bond, affording 6ba together with 5ba in moderate yield (Table 4, entry 1). In the presence of Cu(OTf)_2_, this reaction accelerated and both the γ,δ-site-selectivity and the *endo*-selectivity increased (Table 4, entry 2). On the other hand, in the presence of Cu(NTf_2_)_2_, γ,δ-site-selectivity and *exo*-selectivity were observed (Table 4, entry 3). When β-*tert*-butyl-l-alanine-derived ligand L5 was used with Cu(OTf)_2_, 6ba was obtained in the highest enantioselectivity (96% ee) and good *endo*-selectivity (86%), albeit that the γ,δ-site-selectivity was low (58%) (Table 4, entry 6). Interestingly, when α-l-phenylglycine-derived ligand L7 was used, excellent γ,δ-site-selectivity, *endo*-selectivity, and enantioselectivity were observed (Table 4, entries 9 and 10). In contrast, l-*tert*-butylglycine-derived ligand L6 induced lower γ,δ-site-selectivity and enantioselectivity than L7 (Table 4, entry 8). The use of L3–L7 gave α,β-adduct 5ba*exo*-selectively (Table 4, entries 4–9) as well as α,β-adducts 3*exo*-selectively (Table 2). To the best of our knowledge, our results represent the first example of a Lewis acid-catalyzed γ,δ-site-selective and enantioselective (3 + 2) cycloaddition reactions of α,β–γ,δ-dienamides.

**Table 4:** Optimization of the ligand for the site-selective, *exo*/*endo*-selective, and enantioselective (3 + 2) cycloaddition reaction between 1b and α,β–γ,δ-dienamide 4a^a^

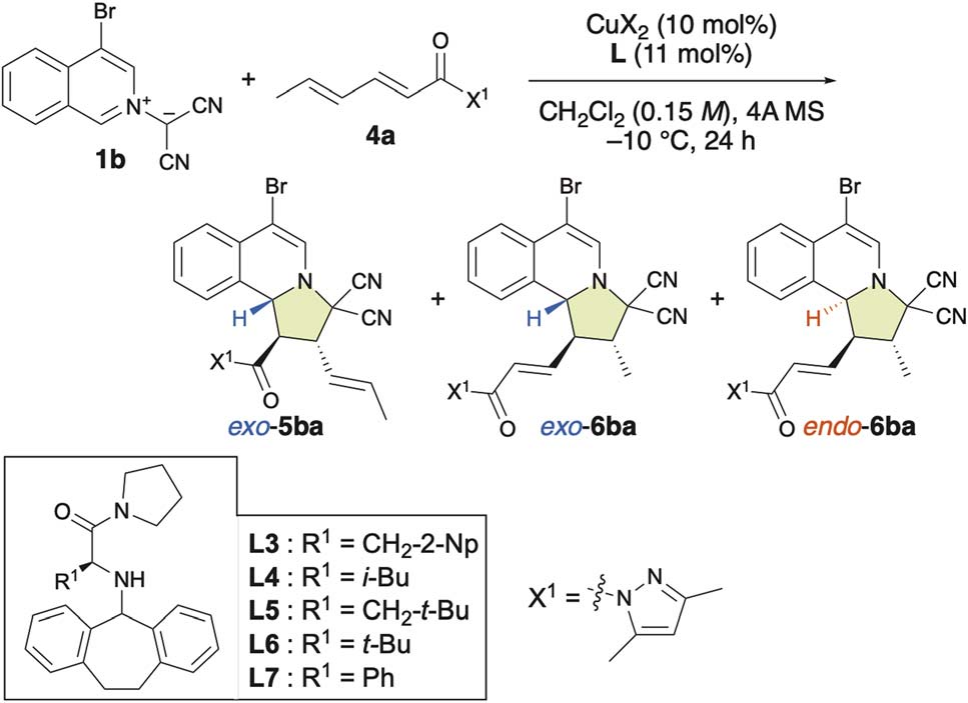						
Entry	CuX_2_·L	5ba + 6ba yield/%, 5ba/6ba	5ba*exo*/*endo*	*Exo*-5ba ee/%	6ba*exo*/*endo*	*Endo*-6ba ee/%
1^bb^	—	57, 22 : 78	26 : 74	—	19 : 81	—
2	Cu(OTf)_2_	>99, 8 : 92	39 : 61	—	6 : 94	—
3	Cu(NTf_2_)_2_ Cu(NTf_2_)_2_	>99, 11 : 89	—	—	90 : 10	—
4	Cu(OTf)_2_·L3	>99, 61 : 39	96 : 4	89	26 : 74	73
5	Cu(OTf)_2_·L4	>99, 63 : 37	>98 : <2	>99	18 : 82	92
6	Cu(OTf)_2_·L5	>99, 42 : 58	>98 : <2	>99	14 : 86	96
7	Cu(NTf_2_)_2_·L5	>99, 30 : 70	>98 : <2	>99	63 : 37	91
8	Cu(OTf)_2_·L6	90, 11 : 89	93 : 7	—	7 : 93	77
9	Cu(OTf)_2_·L7	>99, 7 : 93	85 : 15	>99	7 : 93	92
10	Cu(NTf_2_)_2_·L7	>99, 3 : 97	—	—	6 : 94	94

a Reaction conditions: 1b (0.22 mmol), 4a (0.20 mmol), Cu(OTf)_2_ (10 mol%), L (11 mol%), and 4A MS (150–200 mg) in dichloromethane (1.3 mL) at −10 °C for 24 h. Isolated yields are given. Enantiomeric-excess (ee) values were determined using HPLC. The *exo* : *endo* ratio was determined based on a ^1^H NMR analysis of the crude product.

b The cycloaddition was examined at room temperature in the absence of Cu(OTf)_2_ and L.

To explore the substrate scope of the γ,δ-site-selective, *exo*/*endo*-selective and enantioselective (3 + 2) cycloaddition reaction between 1 and 4, several substrates were examined in the presence of 10 mol% of Cu(OTf)_2_·L5 under the conditions shown in Table 4 (Table 5). The *exo*/*endo*-selectivity for γ,δ-adducts 6 was influenced by the structure of substrates 1 and 4. As expected, the reaction of 1a with 4a exhibited excellent γ,δ-site-selectivity and enantioselectivity to afford *endo*-6aa in high yield. On the other hand, δ-ethyl-α,β–γ,δ-dienamide 4b and δ-*n*-propoyl-α,β–γ,δ-dienamide 4c resulted in the formation of *exo*-6ab and *exo*-6ac in good yield with high γ,δ-site-selectivity and enantioselectivity. Interestingly, 6ac could be obtained *endo*-selectively and with more pronounced γ,δ-site-selectivity but lower enantioselectivity by using L7 instead of L5. Electron-rich isoquinolinium ylides such as 1e and 1f also reacted with 4a in a highly γ,δ-site-selective manner, furnishing the corresponding products *exo*-6ea and *endo*-6fa in good yield with high ee. When employing electron-poor isoquinolinium ylides such as 1b, 1g, and 1d, 1 : 1 mixtures of *exo*-5 and *endo*-6 were obtained in high yields with high enantioselectivity but almost without site-selectivity. Fortunately, *exo*-5 and *endo*-6 could be separated using column chromatography over silica gel. Moreover, the use of L7 instead of L5 significantly enhanced the γ,δ-site-selectivity and *endo*-selectivity, albeit at the expense of a decrease in enantioselectivity. Remarkably, δ-phenyl-α,β–γ,δ-dienamide 4d exhibited complete α,β-site-selectivity, yielding the corresponding products *exo*-5ad, *exo*-5bd, and *exo*-5dd in good yield with >99% ee due to the steric and resonance effects introduced by the δ-phenyl group of 4d.

**Table 5:** Site-selective, *exo*/*endo*-selective, and enantioselective (3 + 2) cycloaddition reaction between 1 and α,β–γ,δ-dienamides 4^a^

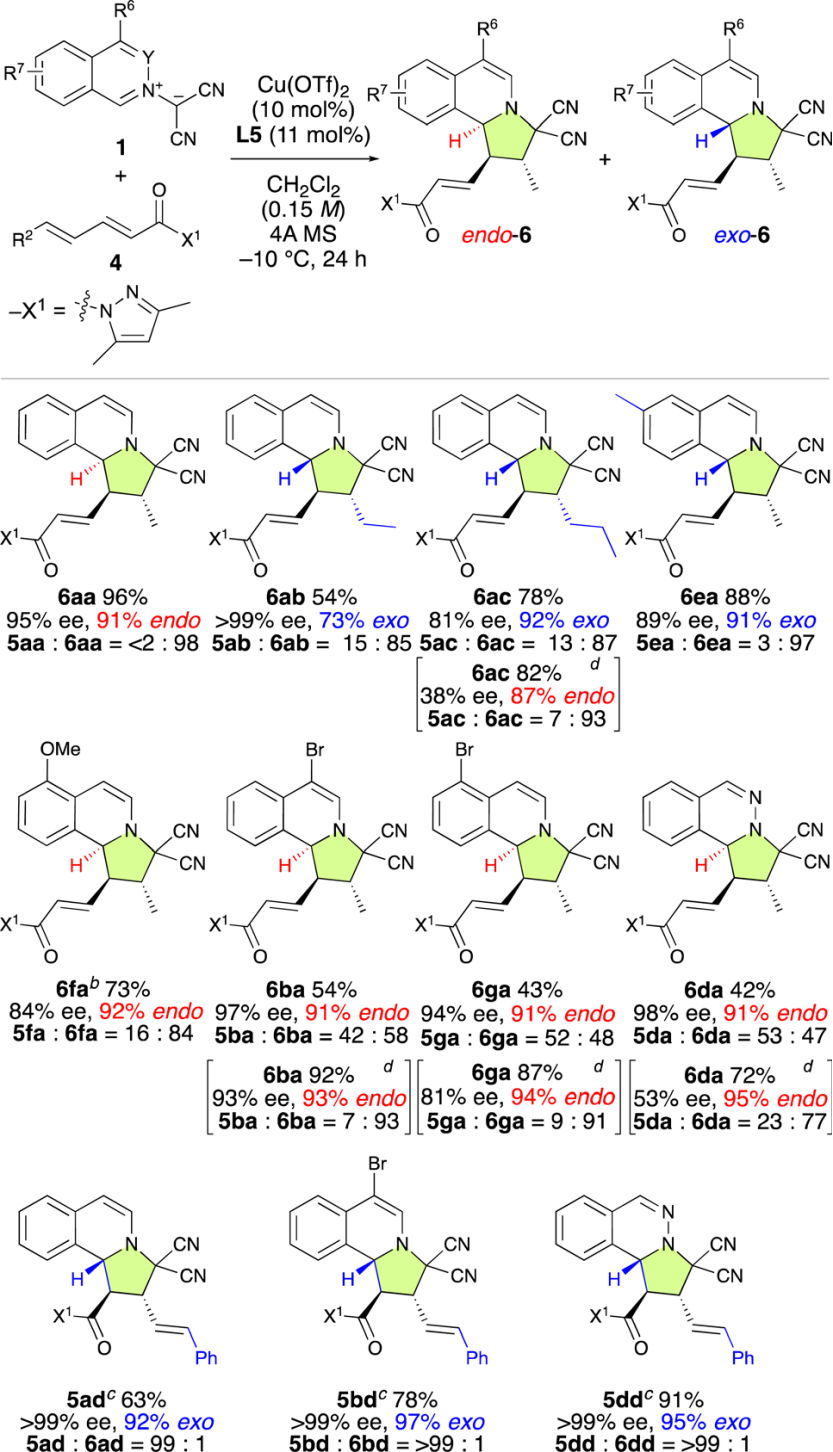

a Unless otherwise noted the reaction conditions were as follows: 4 (0.2 mmol), 1 (0.24 mmol), Cu(OTf)_2_ (10 mol%), L5 (11 mol%), and 4A MS (150–200 mg) in dichloromethane (1.3 mL) at −10 °C for 24 h. Isolated yields. Enantiomeric excess (ee) values were determined using HPLC. The ratios of 5/6 and *exo*/*endo* were determined from NMR analysis of the crude product.

b 40 h.

c 48 h.

d L7 was used instead of L5. −15 °C for 24, then −10 °C for 24 h.

### Mechanistic studies

**Scheme 4:**
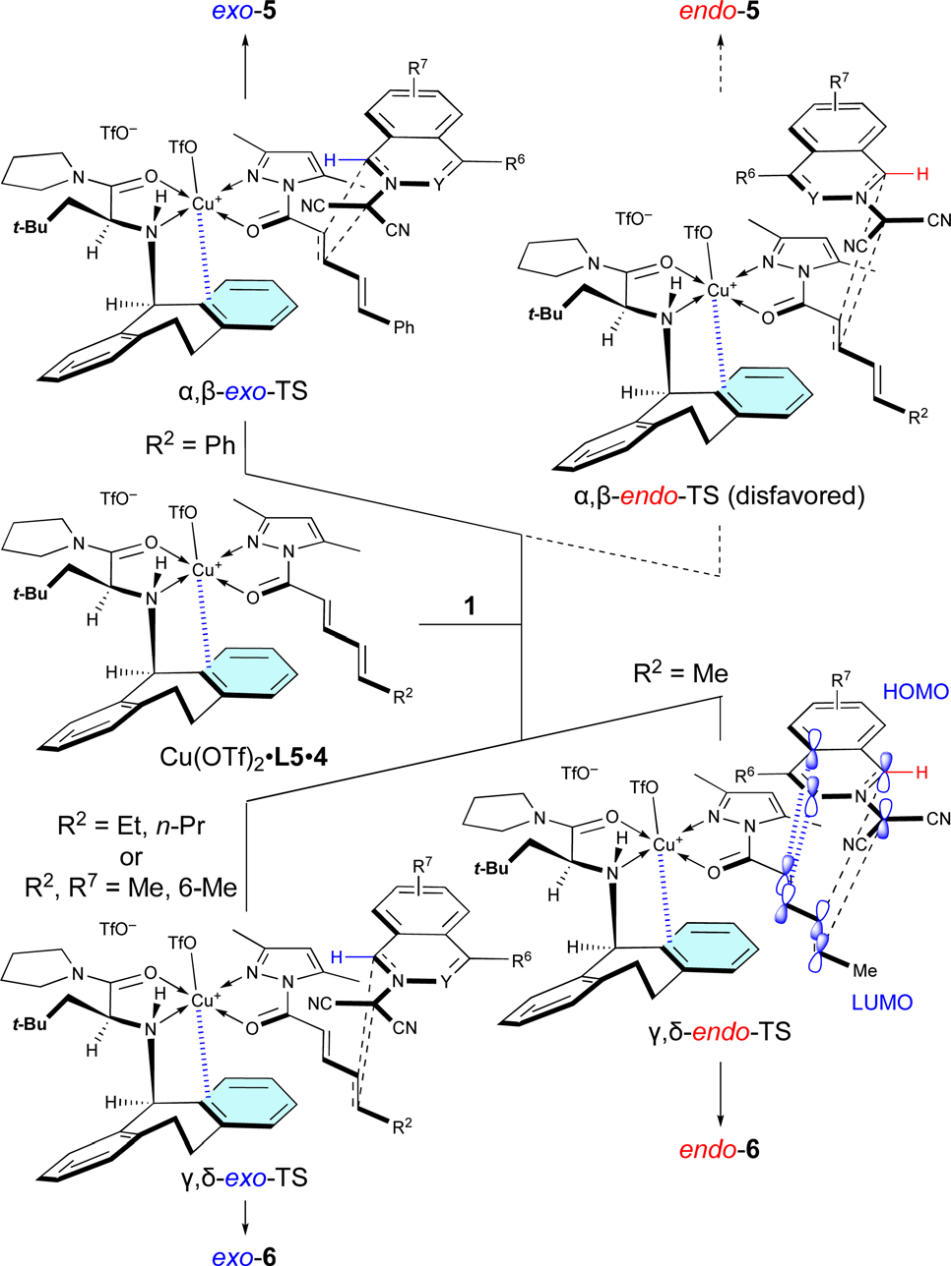
Proposed mechanism for the site-selective, regioselective, and enantioselectivity (3 + 2) cycloaddition of 1 with 4 catalyzed by Cu(OTf)_2_·L5.

The site-selective, *exo*/*endo*-selective, and enantioselective (3 + 2) cycloaddition between 1 and 4 induced by Cu(OTf)_2_·L5 can be rationally interpreted in terms of the mechanism proposed in Scheme 4, which is based upon our previous related studies.^7^ Cu(OTf)_2_·L5 chelates with 4 in such a way that the *N*-Dbs moiety of L5 and the 3,5-dimethylpyrazole moiety of 4 are *anti* to each other due to steric hindrance. Then, dipolar 1a approaches the α,β-double bond of 4c, activated by the π–Cu(ii) complex of Cu(OTf)_2_·L5, from the back of the *N*-Dbs group in a regio-, *exo*-, and enantioselective manner to give *exo*-5ac*via* α,β-*exo*-TS. The α,β-site-selectivity is explained by the fact that the largest coefficient of the LUMO of 4 is located at the β-position (for details, see Section 11 of the ESI†) and by the instability of α,β-*endo*-TS due to the steric repulsion between 1a and Cu(OTf)_2_·L5. However, in other examples, dipolar 1 approaches the γ,δ-double bond of 4a in a regio-, *endo*-, and enantioselective manner to give *endo*-6*via* γ,δ-*endo*-TS. These results mean that the δ-position of 4a is more reactive than the β-position as it is a sterically less-hindered site. The *endo*-selectivity of the reaction can be understood by extra-bond overlap between the HOMO and LUMO as shown in the γ,δ-*endo*-TS. However, the δ-*n*-propyl group of 4b and the 6-methyl group of 1e destabilize the γ,δ-*endo*-TS due to steric hindrance to give *exo*-6ab and *exo*-6ea*via* the γ,δ-*exo*-TS.

**Fig. 2:**
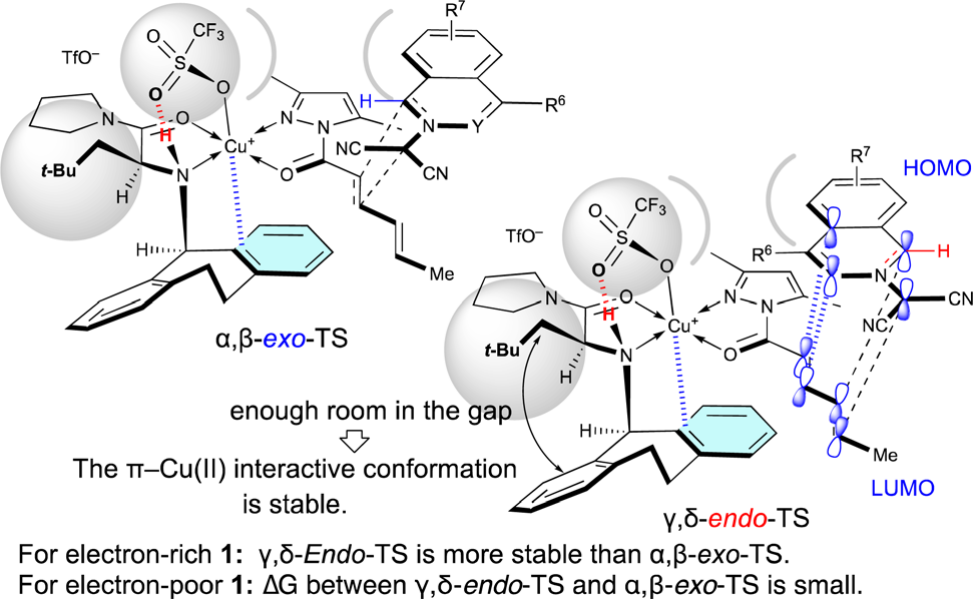
Steric effect of the α-substituent (*R*^1^ = *t*-BuCH_2_) of L5 on the γ,δ-site-selectivity and the *endo*-selectivity.

As shown in Table 5, when Cu(OTf)_2_·L5 was used as the catalyst for the (3 + 2) cycloaddition between 1 and 4a, the electron density of 1 influences the site-selectivity. Both α,β-*exo*-TS and γ,δ-*endo*-TS would be somewhat destabilized by the steric hindrance arising from the apical-OTf, which is conformationally restricted by a hydrogen-bonding interaction with the N–H moiety of L5 (Fig. 2).^7*k*^ Nevertheless, in the (3 + 2) cycloaddition of electron-rich 1, γ,δ-*endo*-TS would be stabilized to exhibit γ,δ-site-selectivity because the strong HOMO–LUMO interaction overcomes the steric hindrance. On the other hand, in the (3 + 2) cycloaddition of electron-poor 1, the site-selectivity is low because the steric hindrance cannot be overcome by the weak HOMO–LUMO interaction.

**Fig. 3:**
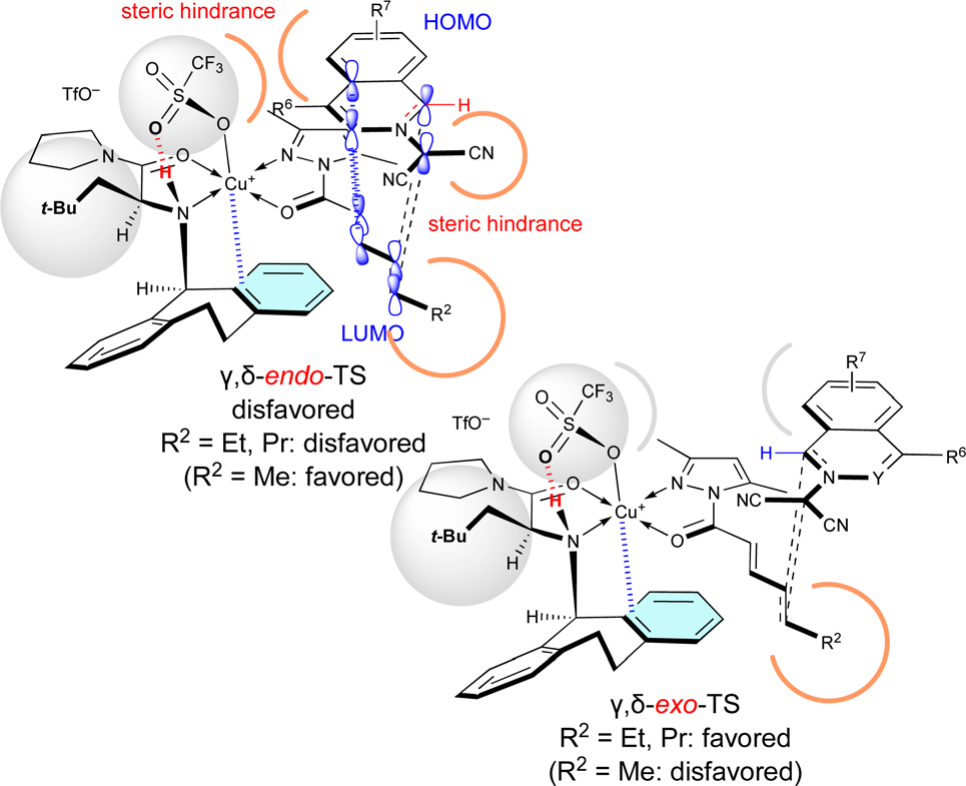
Steric effect of the δ-substituent (*R*^2^ = Et, Pr) of 4 on the γ,δ-site-selectivity and *exo*-selectivity.

Surprisingly, *R*^2^ of 4 also influenced *exo*/*endo*-selectivity of γ,δ-site-adduct 6 : 91% *endo* for 6aa (*R*^2^ = Me), 73% *exo* for 6ab (*R*^2^ = Et), and 92% *exo* for 6ac (*R*^2^ = Pr) (Table 5). As shown in Fig. 3, in the reaction of 1a with 4a, γ,δ-*endo*-TS is preferred due to the strong HOMO–LUMO interaction. However, in the reaction of 1a with 4b or 4c, γ,δ-*exo*-TS is preferred, because the steric hindrance between *R*^2^, 1a, and Cu-OTf is increased and overcomes the HOMO–LUMO interaction.

**Fig. 4:**
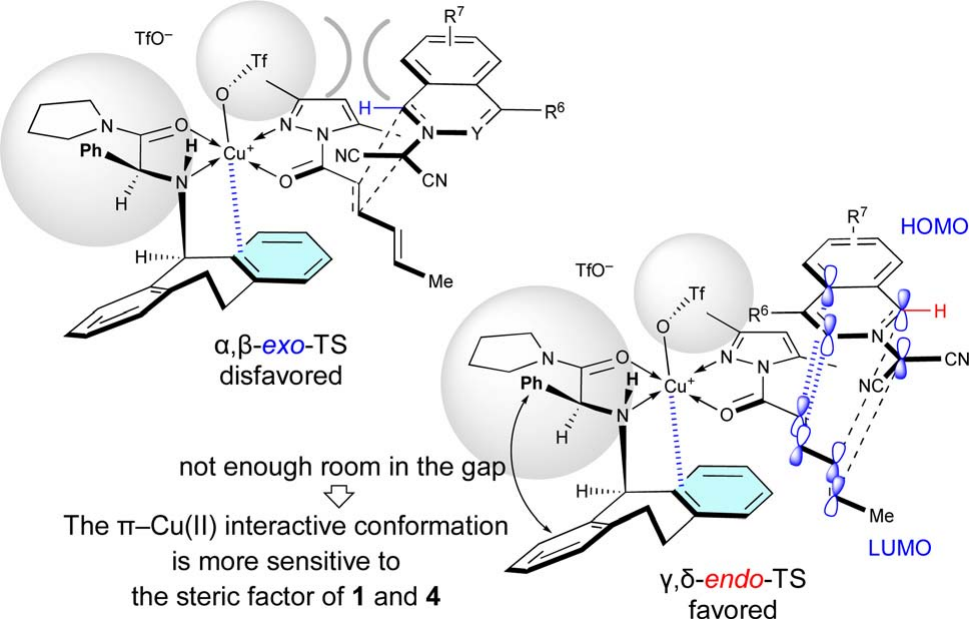
Steric effect of the α-substituent (*R*^1^ = Ph) of L7 on the γ,δ-site-selectivity and *endo*-selectivity.

When Cu(OTf)_2_·L7 was used as the catalyst for the (3 + 2) cycloaddition between electron-poor 1 and 4a, the γ,δ-site-selectivity increased (see Table 5). A hydrogen-bonding interaction between the apical-OTf group and the N–H moiety of L7 would not be expected due to the high steric demand of the α-Ph group of L7.^7*k*^ The apical-OTf group in the transition state would be oriented in the direction opposite to the *N*-acyl group of 4a due to steric hindrance with the α-Ph group of L7 (Fig. 4). Therefore, the α,β-*exo*-TS would be destabilized by the steric hindrance of the apical-OTf group. Thus, the γ,δ-*endo*-selectivity would be increased independent of the electron-density of 1. However, L5 has a more pronounced effect on the enantioselectivity than L7 in most cases, because the π–Cu(ii) interactive conformation in the γ,δ-*endo*-TS containing L7 may be more sensitive to the steric factor of 1 and 4 due to the lack of room in the gap between the α-Ph group and *N*-Dbs in L7.

### Chiral π–Cu(ii)-complex-catalyzed site-selective, *exo*/*endo*-selective and enantioselective dearomative (3 + 2) cycloaddition between an isoquinolinium ylide and an α,β–γ,δ–ε,ζ-trienamide

**Scheme 5:**
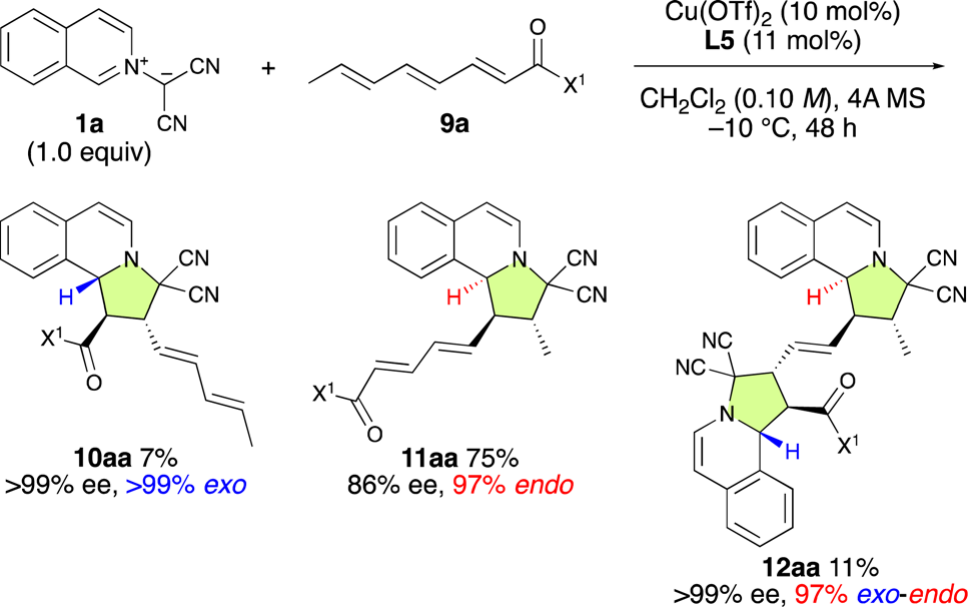
The ε,ζ-site-selective, *endo*-selective, and enantioselective (3 + 2) cycloaddition between 1a and 9a catalyzed by Cu(OTf)_2_·L5.

Finally, Cu(OTf)_2_·L5 was examined for the enantioselective (3 + 2) cycloaddition reaction between 1a and α,β–γ,δ–ε,ζ-trienamide 9a (Scheme 5). As expected, ε,ζ-*endo*-11aa was obtained as the major product in 86% ee. Interestingly, α,β-*exo*-10aa was obtained in 7% yield and >99% ee, while α,β-*exo*-ε,ζ-*endo*-12aa was obtained in 11% yield and >99% ee. These results suggest that the major enantiomer of ε,ζ-*endo*-11aa might be selectively activated by Cu(OTf)_2_·L5 to afford α,β-*exo*-ε,ζ-*endo*-12aa with 99% ee.

## Conclusions

In summary, we have developed a chiral π–Cu(ii) complex-catalyzed multi-selective dearomative (3 + 2) cycloaddition reaction between various substituted alkenes and isoquinolinium ylides for the construction of pyrroloisoquinoline derivatives. The key highlights of this catalytic method include: (1) highly effective chiral ligands L5 and L7 were developed and can be used in catalytic quantities as low as 1–10 mol%; (2) the reaction demonstrates an excellent ability to construct pyrroloisoquinoline derivatives with up to three chiral carbon centers; (3) poorly reactive α-substituted-, α,β-disubstituted, and β,β-disubstituted unsaturated *N*-acylpyrazoles are highly compliable in the reaction and in most cases furnished enantiopure or highly enantioenriched products in excellent yields; (4) valuable chiral F-containing and CF_3_-containing quaternary carbon centers can be accessed efficiently; (5) the first examples for chiral Lewis-acid-catalyzed γ,δ-site-selective, *exo*/*endo*-selective, and enantioselective (3 + 2) cycloaddition reactions that involve α,β–γ,δ-dienamides have been developed; and (6) the first example of an ε,ζ-site-selective, *endo*-selective, and enantioselective (3 + 2) cycloaddition reaction involving an α,β–γ,δ–ε,ζ-trienamide has been developed, which significantly expands the boundaries of this synthetic approach.

## Data availability

General information, detailed experimental procedures, characterization data for compounds, and NMR, HPLC, IR spectra are available in the ESI.†

## Author contributions

The authors confirm to the paper as follows: conception and design: K. I.; methodology and experiments: W. G.; purification and analysis: J. H.; draft manuscript preparation: K. I. and W. G.

## Conflicts of interest

There are no conflicts to declare.

## Supplementary Material

SC-015-D4SC02946A-s001

SC-015-D4SC02946A-s002
