# Supplementary material for: Chiral π–Cu(ii)-catalyzed site-, exo/endo-, and enantioselective dearomative (3 + 2) cycloadditions of isoquinolinium ylides with enamides, dienamides, and a trienamide
Source: Chem Sci. 2024 Jun 11;15(28):10926–34. doi: 10.1039/d4sc02946a (PMC11253176; doi:10.1039/d4sc02946a)
Supplement: SC-015-D4SC02946A-s001 [file SC-015-D4SC02946A-s001.pdf]

Electronic Supplementary Material (ESI) for Chemical Science.
This journal is © The Royal Society of Chemistry 2024

Supplementary Information

Chiral π–Cu(II)-catalyzed Site-, *Exo/Endo*-, and Enantioselective Dearomative [3+2] Cycloadditions of Isoquinolinium Ylides with Enamides, Dienamides and a Trienamide

Weiwei Guo, Jianhao Huang, and Kazuaki Ishihara*
Graduate School of Engineering, Nagoya University, Furo-cho, Chikusa-ku, Nagoya 464-8603, Japan

Table of Contents

1. General Methods S2

2. Preparation of Ligands S2

3. Preparation of 2 and 4 .... S3

4. General Procedure for the Dearomative [3+2] Cycloaddition Reactions of 1 with 2 or 4 (Using L5, Tables 2–5) S5

5. General Procedure for the Remote Asymmetric Dearomative [3+2] Cycloaddition Reactions of 1b, 1d and 1g with 2a and 2c (Using L7, Table 5) S5

6. The Corresponding Physical and Spectroscopic Data of Products 3, 5 and 6 .... S6

7. Synthetic Transformation of 3am (Scheme 3) S19

8. General Procedure for the Remote Asymmetric Dearomative [3+2] Cycloaddition Reactions of 1a with 9a S20

9. NOE Analysis of 6aa, 6ab and 6ac S22

10. X-ray Diffraction Analysis of (1*R*,2*S*,10b*R*)-3de (Fig. 1) S22

11. HOMO of 1a and LUMO of 4a S24

12. References S38

13. NMR Spectra and HPLC Charts S39

1. General Methods

IR spectra were recorded on a JASCO FT/IR-460 plus spectrometer. ^{1}H spectra were measured on a JEOL ECS-400 spectrometer (400 MHz) at ambient temperature. Chemical shifts are reported in ppm from Me_4Si resonance (0.00 ppm) as an internal standard for $CDCl_3$. Data were recorded as follows: chemical shift, multiplicity (s = singlet; d = doublet; t = triplet; q = quartet; m = multiplet, br = broad), coupling constant (Hz), and integration. ^{13}C NMR spectra were measured on a JEOL ECS-400 (100 MHz). ^{19}F NMR spectra were measured on a JEOL ECS-400 spectrometer (376 MHz). Chemical shifts were recorded in ppm from the solvent resonance employed as the internal standard ($CDCl_3$: 77.10 ppm). High-performance liquid chromatography (HPLC) analysis was conducted using Shimadzu LC-10 AD coupled diode array-detector SPD-MA-10A-VP and chiral column of Daicel CHIRALCEL OD-3 (4.6 mm × 25 cm), Daicel CHIRALCEL AD-3 (4.6 mm × 25 cm), Daicel CHIRALCEL IA-3 (4.6 mm × 25 cm), Daicel CHIRALCEL IC-3 (4.6 mm × 25 cm), Daicel CHIRALCEL AS-3 (4.6 mm × 25 cm). Optical rotations were measured on Rudolph Autopol IV digital polarimeter. For Thin-layer chromatography TLC analysis, Merck precoated TLC plates (silica gel 60 F_{254} 0.25 mm) or silica gel 60 NH_2 F_{254}s 0.20 mm) were used. The products were purified by column chromatography on silica gel (E. Merck Art. 9385; Kanto Chemical Co., Inc. 37560; Fuji Silysia Chemical Ltd. Chromatorex® NH-DM1020). X-ray diffraction analysis was performed by Rigaku PILATUS-200K. High resolution mass spectral analysis (HRMS) was performed at Chemical Instrument Facility, Nagoya University. Dry dichloromethane was purchased from Kanto as the "anhydrous" and stored under nitrogen. Dry acetonitrile was distilled from CaH_2 and dried over 4A molecular sieves. The isoquinolinium ylides were synthesized according to the procedure described in the literature.[8e] Other materials were obtained from commercial supplies and used without further purification.

2. Preparation of Ligands.

The ligands **L1–L7** were prepared according to the procedures described in the literatures.[7f,1]

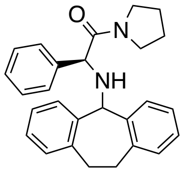

(*S*)-(+)-2-((10,11-dihydro-5*H*-dibenzo[*a*,*d*][7]annulen-5-yl)amino)-2-phenyl-1-(pyrrolidin-1-yl)ethan-1-one (L7): White solid; **[α]$^{26}_{D}$** +92.4 (*c* 0.90, $CHCl_3$); **^{1}H NMR** (400 MHz, $CDCl_3$) δ 7.35–7.24 (m, 3H), 7.24–7.00 (m, 10H), 4.77 (s, 1H), 4.13 (s, 2H), 3.80–3.59 (m, 2H), 3.54–3.42 (m, 1H), 3.08–2.65 (m, 5H), 1.92–1.64 (m, 4H); **^{13}C NMR** (100 MHz, $CDCl_3$) δ 170.6, 130.8, 130.2, 128.7, 128.1, 127.9, 127.7, 127.5, 125.9, 125.7, 61.7, 46.0, 45.7, 33.0, 32.1, 26.0, 24.2; **HRMS** (FAB^+) *m/z*: $[M+H]^+$ calcd for $C_{27}H_{29}N_2O$ 397.2274; found 397.2279.

3. Preparation of 2 and 4

2g, **2h**, **2p**–**2s**, **4b**–**4c** and **7a** were prepared based on the procedures described in the literatures:[2d,7a,12]

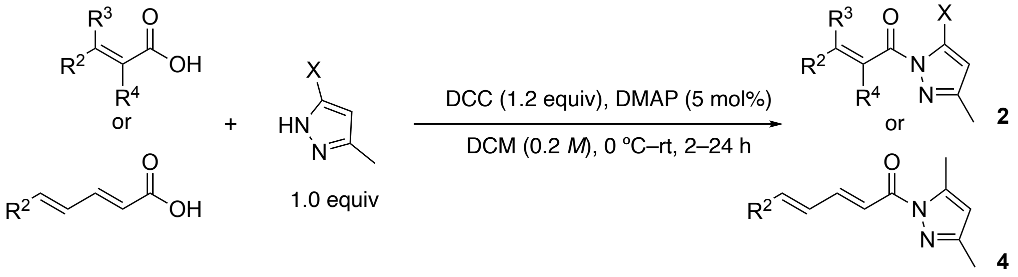

Pyrazole (5 mmol) and carboxylic acid (1.2 equiv, 6 mmol) were dissolved in CH_2Cl_2 (0.2 *M*) at 0 ºC. Then the DCC (6 mmol) and DMAP (0.05 mmol) were added. After stirring for 10 min, the reaction mixture was then moved to room temperature and stirred for several hours until the pyrazole was fully consumed (monitored by TLC). The reaction mixture was filtered, and the filtration was then concentrated *in vacuo*. Then the resultant mixture was purified by silica gel column chromatography (Hexane/EA = 25:1 to 10:1) to give **2**, **4** and **7**.

The corresponding physical and spectroscopic data for **2g**, **2h**, **2p**–**2s**, **4b**–**4c** and **7a** are as follows.

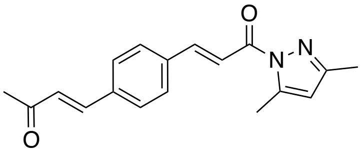

(*E*)-4-(4-((*E*)-3-(3,5-Dimethyl-1*H*-pyrazol-1-yl)-3-oxoprop-1-en-1-yl)phenyl)but-3-en-2-one (2g): White solid; **^{1}H NMR** (400 MHz, $CDCl_3$) δ 7.99 (d, *J* = 16.4 Hz, 1H), 7.87 (d, *J* = 16.0 Hz, 1H), 7.70 (d, *J* = 8.8 Hz, 2H), 7.59 (d, *J* = 8.4 Hz, 2H), 7.51 (d, *J* = 16.4 Hz, 1H), 6.76 (d, *J* = 16.0 Hz, 1H) 6.03 (s, 1H), 2.63 (s, 3H), 2.41 (s, 3H), 2.30 (s, 3H); **^{13}C NMR** (100 MHz, $CDCl_3$) δ 198.3, 165.2, 152.2, 144.8, 144.5, 142.3, 136.8, 136.5, 129.3, 128.7, 128.0, 119.3, 111.7, 27.8, 14.8, 13.9; **HRMS** (ESI+) calcd for $C_{18}H_{18}N_2NaO_2$ $[M+Na]^+$ 317.1260; found 317.1236.

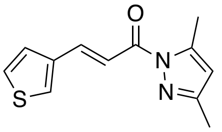

(*E*)-1-(3,5-Dimethyl-1*H*-pyrazol-1-yl)-3-(thiophen-3-yl)prop-2-en-1-one (2h): White solid; **^{1}H NMR** (400 MHz, $CDCl_3$) δ 7.89 (d, *J* = 16.0 Hz, 1H), 7.74 (d, *J* = 16.0, 1H), 7.61 (d, *J* = 2.8 Hz, 1H), 7.48 (d, *J* = 4.4 Hz, 1H), 7.38–7.31 (m, 1H), 6.01 (s, 1H), 2.62 (s, 3H), 2.29 (s, 3H); **^{13}C NMR** (100 MHz, $CDCl_3$) δ 165.8, 151.9, 144.5, 139.6, 138.2, 129.2, 126.9, 125.9, 117.6, 111.5, 14.8, 13.9; **HRMS** (ESI+) calcd for $[M+H]^+$$C_{12}H_{13}N_2OS$ 233.0743; found 233.0724.

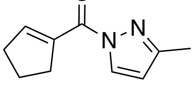

Cyclopent-1-en-1-yl(3-methyl-1*H*-pyrazol-1-yl)methanone (2p): Colorless oil; **^{1}H NMR** (400 MHz, $CDCl_3$) δ 8.21 (d, *J* = 2.8 Hz, 1H), 7.47–7.42(m, 1H), 6.25 (d, *J* = 2.8 Hz, 1H),

2.88–2.79 (m, 2H), 2.71–2.62 (m, 2H), 2.34 (s, 3H), 2.23–1.92 (m, 2H); **^{13}C NMR** (100 MHz, $CDCl_3$) δ 167.8, 152.9, 144.6, 111.8, 76.1, 72.6, 27.5, 14.5, 13.9; **HRMS** (ESI+) calcd for $C_{10}H_{12}N_2NaO$ $[M+Na]^+$ 199.0842; found 199.0826.

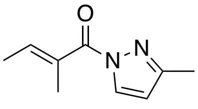

(*E*)-2-Methyl-1-(3-methyl-1*H*-pyrazol-1-yl)but-2-en-1-one (2q): Colorless oil; **^{1}H NMR** (400 MHz, $CDCl_3$) δ 8.14 (d, *J* = 2.8 Hz, 1H), 6.81–6.72(m, 1H), 6.24 (d, *J* = 2.8 Hz, 1H), 2.33 (s, 3H), 2.04 (m, 3H),1.91 (dd, *J* = 1.0, 7.0 Hz, 3H) ; **^{13}C NMR** (100 MHz, $CDCl_3$) δ 168.1, 153.7, 140.3, 131.1, 130.4, 109.6, 14.7, 14.1, 13.9; **HRMS** (ESI+) calcd for $C_{19}H_{12}N_2NaO$ $[M+Na]^+$ 187.0842; found 187.0820.

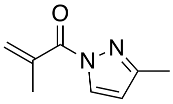

2-Methyl-1-(3-methyl-1*H*-pyrazol-1-yl)prop-2-en-1-one (2r): Colorless oil; **^{1}H NMR** (400 MHz, $CDCl_3$) δ 8.17 (d, *J* = 2.8 Hz, 1H), 6.28 (d, *J* = 2.8 Hz,1H), 6.04 (m, 1H), 5.85 (m, 1H), 2.23 (s, 3H), 2,16 (d, *J* = 2.8 Hz, 3H); **^{13}C NMR** (100 MHz, $CDCl_3$) δ 167.3, 154.3, 137.6, 130.8, 127.1, 110.2, 20.2, 14.1; **HRMS** (ESI+) calcd for $C_8H_{10}N_2NaO$ $[M+Na]^+$ 173.0685; found 173.0685.

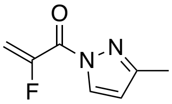

2-Fluoro-1-(3-methyl-1H-pyrazol-1-yl)prop-2-en-1-one (2s): Colorless oil; **^{1}H NMR** (400 MHz, $CDCl_3$) δ 8.24 (d, *J* = 2.8 Hz, 1H), 6.44 (dd, *J* = 3.8 Hz, 46.6 Hz, 1H), 6.32 (d, *J* = 3.2 Hz, 1H), 5.74 (dd, *J* = 3.6 Hz, 16.4 Hz, 1H), 2.35 (s, 3H); **^{13}C NMR** (100 MHz, $CDCl_3$) δ 157.5 (d, *J* = 35.3 Hz, 1C), 155.5, 143.5 (d, *J* = 255.6 Hz, 1C), 131.4 (d, *J* = 2.9 Hz, 1C), 111.1, 108.5 (d, *J* = 18.3 Hz, 1C), 14; **^{19}F NMR** (376 MHz, $CDCl_3$) δ –110.4; **HRMS** (ESI+) calcd for $C_{10}H_{12}N_2NaO$ $[M+Na]^+$ 199.0842; found 199.0826.

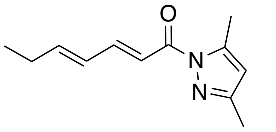

(2*E*,4*E*)-1-(3,5-Dimethyl-1*H*-pyrazol-1-yl)hepta-2,4-dien-1-one (4b): Colorless oil; **^{1}H NMR** (400 MHz, $CDCl_3$) δ 7.49 (dd, *J* = 10.0, 15.2 Hz, 1H), 7.26 (d, *J* = 15.6 Hz, 1H), 6.42–6.23 (m, 2H), 5.98 (s, 1H), 2.58 (s, 3H), 2.26 (s, 3H), 2.26–2.19 (m, 2H), 1.07 (t, *J* = 7.6 Hz, 3H); **^{13}C NMR** (100 MHz, $CDCl_3$) δ 165.9, 151.7, 147.6 147.0, 144.4, 128.2, 119.0, 111.2, 26.2, 14.7, 13.9, 12.9; **HRMS** (ESI+) calcd for $C_{12}H_{17}N_2O$ $[M+H]^+$ 205.1335; found 205.1343.

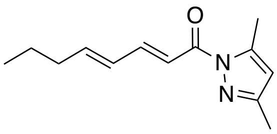

(2*E*,4*E*)-1-(3,5-Dimethyl-1*H*-pyrazol-1-yl)octa-2,4-dien-1-one (4c): Colorless oil; **^{1}H NMR** (400 MHz, $CDCl_3$) δ 7.49 (dd, *J* = 10.6, 15.4 Hz, 1H), 7.29–7.26 (m, 1H), 6.35 (dd, *J* = 10.4, 15.2 Hz, 1H), 6.29–6.20 (m, 1H), 5.98 (s, 1H), 2.59 (s, 3H), 2.26 (s, 3H), 2.23–

2.13 (m, 2H), 1.52–1.42 (m, 2H), 0.93 (t, J = 7.4 Hz, 3H); **^{13}C NMR** (100 MHz, $CDCl_3$) δ 165.9, 151.7, 146.9, 146.1, 144.3, 129.3, 119.0, 111.2, 32.2, 21.9, 14.7, 13.9, 13.8; **HRMS** (ESI+) calcd for $C_{13}H_{19}N_2O$ $[M+H]^+$ 219.1492; found 219.1455.

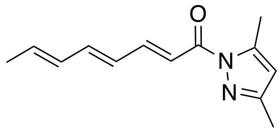

(2*E*,4*E*,6*E*)-1-(3,5-Dimethyl-1*H*-pyrazol-1-yl)octa-2,4,6-trien-1-one (9a): White solid; **^{1}H NMR** (400 MHz, $CDCl_3$) δ 7.52 (dd, J = 11.4, 15.0 Hz, 1H), 7.31 (d, J = 15.6 Hz, 1H), 6.62 (dd, J = 10.6, 15.0 Hz, 1H), 6.38 (dd, J = 11.0, 15.0 Hz, 1H), 6.26–6.14 (m, 1H), 6.05–5.92 (m, 2H), 2.59 (s, 3H), 2.26 (s, 3H), 1.85 (d, J = 7.2 Hz, 3H); **^{13}C NMR** (100 MHz, $CDCl_3$) δ 165.7, 151.7, 146.6, 144.3, 142.4, 135.9, 131.5, 128.4, 119.8, 111.3, 18.7, 14.7, 13.9; **HRMS** (ESI+) calcd for $C_{13}H_{17}N_2O$ $[M+H]^+$ 217.1335; found 217.1341.

4. General Procedure for the Dearomative [3+2] Cycloaddition Reactions of 1 with 2 or 4 (Using L5, Tables 2–5)

A mixture of monopeptide ligand **L5** (11 mol%), copper(II) triflate (10 mol%) and activated molecular sieves 4A (150–200 mg) were dissolved in anhydrous acetonitrile (1.0 mL). After stirring for 15 min at ambient temperature, the solution was concentrated under reduced pressure. The resultant residue was diluted with CH_2Cl_2 (1.3 mL) and cooled to –10ºC (–40 ºC for **3ai**, **3am** and **3an**). Isoquinolinium ylides **1** (0.22 mmol, 1.1 equiv) and **2** or **4** (0.2 mmol) were then added and the reaction mixture was stirred at the same temperature for 6–48 h. The reaction was quenched with a few drops of triethylamine and then passed through a short pad silica gel to remove ligand and copper. After evaporation of the filtrate under reduced pressure, the crude product was then purified by column chromatography on silica gel (hexane–EtOAc = 10:1 to 3:1) to give **3**, **5** and **6**.

5. General Procedure for the Remote Asymmetric Dearomative [3+2] Cycloaddition Reactions of 1b, 1d and 1g with 2a and 2c (Using L7, Table 5)

A mixture of monopeptide ligand **L7** (11 mol%), copper(II) triflate (10 mol%) and activated molecular sieves 4A (150–200 mg) were dissolved in anhydrous acetonitrile (1.0 mL). After stirring for 15 min at ambient temperature, the solution was concentrated under reduced pressure. The resultant residue was diluted with CH_2Cl_2 (1.3 mL) and cooled to –15ºC. Isoquinolinium ylides **1** (0.22 mmol, 1.1 equiv) and **2** or **4** (0.2 mmol) were then added. After stirring at the same temperature for 24 h, the reaction was then moved to –10ºC for another 24 h. The reaction was quenched with a few drops of triethylamine and then passed through a short pad silica gel to remove ligand and copper. After evaporation of the filtrate under reduced pressure, the crude product was then purified by column chromatography on silica gel (hexane–EtOAc = 10:1 to 3:1) to give the target products.

6. The Corresponding Physical and Spectroscopic Data of Products 3, 5 and 6

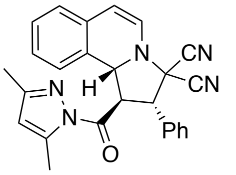

(1*R*,2*S*,10b*R*)-(+)-1-(3,5-Dimethyl-1*H*-pyrazole-1-carbonyl)-2-phenyl-1,10b-dihydropyrrolo[2,1-*a*]isoquinoline-3,3(2*H*)-dicarbonitrile (3aa) (Table 2): 98% yield; Yellow foam; **[α]$^{31}_{D}$** +576.7 (98% ee, *c* 0.80, $CHCl_3$); **^{1}H NMR** (400 MHz, $CDCl_3$) δ 7.67–7.58 (m, 2H), 7.39–7.34 (m, 3H), 7.21 (t, *J* = 7.4 Hz, 1H), 7.14–7.02 (m, 2H), 6.86 (d, *J* = 8.0 Hz, 1H), 6.40 (d, *J* = 7.2 Hz, 1H), 6.03–5.98 (m, 2H), 5.74 (dd, *J* = 8.8, 11.2 Hz, 1H), 5.47 (d, *J* = 8.8 Hz, 1H), 4.53 (d, *J* = 10.8 Hz, 1H), 2.49 (s, 3H), 2.23 (s, 3H); **^{13}C NMR** (100 MHz, $CDCl_3$) δ 170.6, 153.5, 144.7, 131.2, 130.1, 129.9, 129.3, 129.0, 128.4, 127.8, 127.0, 125.1, 124.3, 113.2, 112.9, 112.1, 110.7, 63.5, 61.9, 59.5, 50.1, 14.5, 13.8; **HRMS** (ESI+) calcd for $C_{26}H_{21}N_5NaO$ $[M+Na]^+$ 442.1633, found 442.1632; The ee was determined by **HPLC** analysis (Daicel Chiralcel OD-3 column, hexane–*i*-PrOH = 98: 2, flow rate = 1.0 mL/min) t_R= 9.9 (major enantiomer), 11.8 (minor) min.

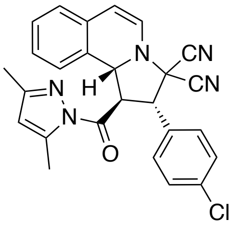

(1*R*,2*S*,10b*R*)-(+)-2-(4-chlorophenyl)-1-(3,5-dimethyl-1*H*-pyrazole-1-carbonyl)-1,10b-dihydropyrrolo[2,1-*a*]isoquinoline-3,3(2*H*)-dicarbonitrile (3ab) (Table 2): 95% yield; Yellow form; **[α]$^{30}_{D}$** +572.4 (>98% ee, *c* 0.92, $CHCl_3$); **^{1}H NMR** (400 MHz, $CDCl_3$) δ 7.56 (d, *J* = 8.8 Hz, 2H), 7.34 (d, *J* = 8.8 Hz, 2H), 7.21 (t, *J* = 7.4 Hz, 1H), 7.14–7.02 (m, 2H), 6.82 (d, *J* = 7.2 Hz, 1H), 6.39 (d, *J* = 7.2 Hz, 1H), 6.03 (s, 1H), 6.01 (d, *J* = 7.2 Hz, 1H), 5.69 (dd, *J* = 8.8, 11.2 Hz, 1H), 5.46 (d, *J* = 8.4 Hz, 1H), 4.48 (d, *J* = 10.8 Hz, 1H), 2.50 (s, 3H), 2.23 (s, 3H); **^{13}C NMR** (100 MHz, $CDCl_3$) δ 170.3, 153.7, 144.8, 136.1, 131.1, 130.7, 129.3, 128.9, 128.5, 127.9, 126.8, 125.2, 124.2, 113.0, 112.9, 119.5, 110.9, 63.4, 61.7, 58.9, 50.1, 14.5, 13.9; **HRMS** (ESI+) calcd for $C_{26}H_{20}ClKN_5O$ $[M+K]^+$ 492.0988, found 492.0932; The ee was determined by **HPLC** analysis (Daicel Chiralcel AD-3 column, hexane–*i*-PrOH = 98.4: 1.6, flow rate = 1.0 mL/min) t_R= 21.2 (major enantiomer), 30.7 (minor) min.

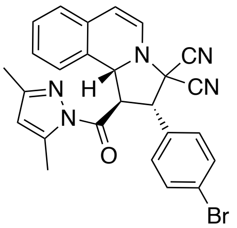

(1*R*,2*S*,10b*R*)-(+)-2-(4-Bromophenyl)-1-(3,5-dimethyl-1*H*-pyrazole-1-carbonyl)-1,10b-dihydropyrrolo[2,1-*a*]isoquinoline-3,3(2*H*)-dicarbonitrile (3ac) (Table 2): 94%

yield; Yellow foam; **[α]$^{29}_{D}$** +528.8 (98% ee, *c* 0.93, $CHCl_3$); **^{1}H NMR** (400 MHz, $CDCl_3$) δ 7.54–7.47 (m, 4H), 7.21 (t, *J* = 7.6 Hz, 1H), 7.14–7.02 (m, 2H), 6.82 (d, *J* = 7.2 Hz, 1H), 6.39 (dd, *J* = 1.8, 7.2 Hz, 1H), 6.02 (s, 1H), 6.00 (d, *J* = 7.6 Hz, 1H), 5.72–5.63 (m, 1H), 5.46 (d, *J* = 8.4 Hz, 1H), 4.47 (dd, *J* = 1.8, 10.8 Hz, 1H), 2.50 (s, 3H), 2.23 (s, 3H); **^{13}C NMR** (100 MHz, $CDCl_3$) δ 170.3, 153.7, 144.8, 132.2, 131.1, 130.0, 129.8, 128.9, 128.5, 127.9, 126.8, 125.2, 124.4, 124.2, 113.0, 112.9, 111.9, 110.9, 63.4, 61.6, 58.9, 50.0, 14.5, 13.9; **HRMS** (ESI+) calcd for $C_{26}H_{20}BrN_5NaO$ $[M+Na]^+$ 520.0743, found 520.0729; The ee was determined by **HPLC** analysis (Daicel Chiralcel OD-3 column, hexane–*i*-PrOH = 98: 2, flow rate = 1.0 mL/min) t_R= 12.8 (major enantiomer), 15.1 (minor) min.

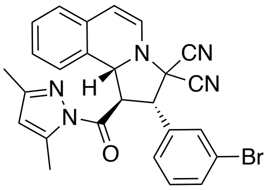

(1*R*,2*S*,10b*R*)-(+)-2-(3-Bromophenyl)-1-(3,5-dimethyl-1*H*-pyrazole-1-carbonyl)-1,10b-dihydropyrrolo[2,1-*a*]isoquinoline-3,3(2*H*)-dicarbonitrile (3ad) (Table 2): 95% yield; Yellow foam; **[α]$^{27}_{D}$** +518.1 (98% ee, *c* 0.68, $CHCl_3$); **^{1}H NMR** (400 MHz, $CDCl_3$) δ 7.91–7.84 (m, 1H), 7.56–7.45 (m, 2H), 7.28–7.18 (m, 2H), 7.14–7.03 (m, 2H), 6.81 (d, *J* = 7.2 Hz, 1H), 6.39 (d, *J* = 7.2 Hz, 1H), 6.06–5.98 (m, 2H), 5.70 (dd, *J* = 8.8, 11.2 Hz, 1H), 5.48 (d, *J* = 8.8 Hz, 1H), 4.45 (d, *J* = 11.2 Hz, 1H), 2.50 (s, 3H), 2.27(s, 3H); **^{13}C NMR** (100 MHz, $CDCl_3$) δ 170.2, 153.8, 144.8, 133.1, 133.0, 132.4, 131.1, 130.5, 128.9, 128.5, 128.1, 128.0, 126.8, 125.2, 124.1, 123.0, 113.1, 112.9, 111.8, 111.0, 63.3, 61.7, 58.8, 50.0, 14.5, 13.9; **HRMS** (ESI+) calcd for $C_{26}H_{20}BrN_5NaO$ $[M+Na]^+$ 520.0743, found 520.0746; The ee was determined by **HPLC** analysis (Daicel Chiralcel OD-3 column, hexane–*i*-PrOH = 98: 2, flow rate = 1.0 mL/min) t_R= 11.0 (major enantiomer), 14.7 (minor) min.

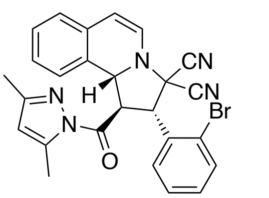

(1*R*,2*S*,10b*R*)-(+)-2-(2-Bromophenyl)-1-(3,5-dimethyl-1*H*-pyrazole-1-carbonyl)-1,10b-dihydropyrrolo[2,1-*a*]isoquinoline-3,3(2*H*)-dicarbonitrile (3ae) (Table 2): 80% yield; Yellow foam; **[α]$^{28}_{D}$** +185.6 (94% ee, *c* 0.67, $CHCl_3$); **^{1}H NMR** (400 MHz, $CDCl_3$) δ 7.69 (dd, *J* = 1.6, 8.0 Hz, 1H), 7.61 (dd, *J* = 1.0, 7.8 Hz, 1H), 7.38–7.30 (m, 1H), 7.29–7.19 (m, 2H), 7.18–6.09 (m, 2H), 6.88 (d, *J* = 8.0 Hz, 1H), 6.48 (d, *J* = 7.6 Hz, 1H), 6.04 (d, *J* = 7.6 Hz, 1H), 5.98 (d, *J* = 1.2 Hz, 1H), 5.43 (d, *J* = 8.8 Hz, 1H), 5.29 (t, *J* = 8.4 Hz, 1H), 5.13 (d, *J* = 8.4 Hz, 1H), 2.55 (s, 3H), 2.04 (s, 3H); **^{13}C NMR** (100 MHz, $CDCl_3$) δ 170.1, 153.3, 144.6, 134.0, 133.1, 131.5, 130.8, 129.9, 129.5, 128.5, 128.0, 127.8, 126.2, 125.1, 124.1, 112.8, 112.5, 111.5, 110.9, 62.4, 59.6, 57.2, 51.9, 14.4, 13.6; **HRMS** (ESI+) calcd for $C_{26}H_{20}BrN_5NaO$ $[M+Na]^+$ 520.0743, found 520.0758; The ee was determined by **HPLC** analysis (Daicel Chiralcel AD-3 column, hexane–*i*-PrOH = 98.4: 1.6, flow rate = 1.0 mL/min) t_R= 17.1 (major enantiomer), 24.8 (minor) min.

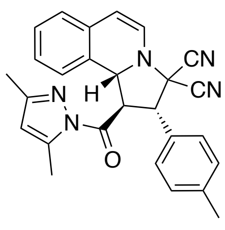

(1*R*,2*S*,10b*R*)-(+)-1-(3,5-Dimethyl-1*H*-pyrazole-1-carbonyl)-2-(*p*-tolyl)-1,10b-dihydropyrrolo[2,1-*a*]isoquinoline-3,3(2*H*)-dicarbonitrile (3af) (Table 2): 96% yield; Yellow form; **[α]$^{29}_{D}$** +568.4 (96% ee, *c* 0.83, $CHCl_3$); **^{1}H NMR** (400 MHz, $CDCl_3$) δ 7.50 (d, *J* = 8.0 Hz, 2H), 7.23–7.12 (m, 3H), 7.13–7.01 (m, 2H), 6.85 (d, *J* = 7.6 Hz, 1H), 6.39 (d, *J* = 7.2 Hz, 1H), 6.00 (s, 1H), 5.98 (d, *J* = 7.2 Hz, 1H), 5.75 (dd, *J* = 8.8, 11.2 Hz, 1H), 5.46 (d, *J* = 8.8 Hz, 1H), 4.52 (d, *J* = 11.6 Hz, 1H), 2.49 (s, 3H), 2.32 (s, 3H); 2.25 (s, 3H); **^{13}C NMR** (100 MHz, $CDCl_3$) δ 170.7, 153.4, 144.7, 139.9, 131.2, 129.7, 129.2, 129.0, 128.3, 127.8, 127.5, 127.0, 125.0, 124.3, 113.2, 112.9, 112.2, 110.5, 63.6, 62.1, 59.3, 49.9, 21.3, 14.5, 13.9; **HRMS** (ESI+) calcd for $C_{27}H_{24}N_5O$ $[M+H]^+$ 434.1975, found 434.1977; The ee was determined by **HPLC** analysis (Daicel Chiralcel IC-3 column, hexane–*i*-PrOH = 98: 2, flow rate = 1.0 mL/min) t_R= 14.9 (major enantiomer), 21.7 (minor) min.

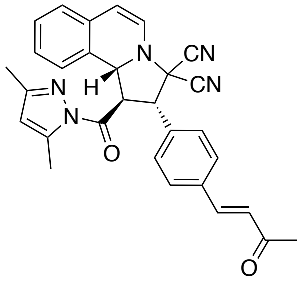

(1*R*,2*S*,10b*R*)-(+)-1-(3,5-Dimethyl-1*H*-pyrazole-1-carbonyl)-2-(4-((*E*)-3-oxobut-1-en-1-yl)phenyl)-1,10b-dihydropyrrolo[2,1-*a*]isoquinoline-3,3(2*H*)-dicarbonitrile (3ag) (Table 2): 95% yield; Yellow form; **[α]$^{29}_{D}$** +582.6 (99% ee, *c* 0.77, $CHCl_3$); **^{1}H NMR** (400 MHz, $CDCl_3$) δ 7.65 (d, *J* = 8.0 Hz, 2H), 7.54 (d, *J* = 8.8 Hz, 2H), 7.54 (d, *J* = 8.8 Hz, 2H), 7.46 (d, *J* = 16.8 Hz, 1H), 7.22(t, *J* = 7.6 Hz, 1H), 7.15–7.03 (m, 2H), 6.85 (d, *J* = 8.0 Hz, 1H), 6.70 (d, *J* = 16.0 Hz, 1H), 6.40 (d, *J* = 7.2 Hz, 1H), 6.05–5.98 (m, 2H), 5.71 (dd, *J* = 8.4, 10.8 Hz, 1H), 5.47 (d, *J* = 8.4 Hz, 1H), 4.53 (d, *J* = 10.8 Hz, 1H), 2.50 (s, 3H), 2.37 (s, 3H); 2.22 (s, 3H); **^{13}C NMR** (100 MHz, $CDCl_3$) δ 198.2, 170.3, 153.6, 144.8, 142.1, 133.2, 131.1, 130.0, 129.0, 128.7, 128.5, 128.2, 127.9, 126.9, 125.2, 124.2, 113.0, 111.9, 111.0, 63.4, 61.6, 59.2, 50.2, 27.7, 14.5, 13.9; **HRMS** (ESI+) calcd for $C_{30}H_{26}N_5O_2$ $[M+H]^+$ 488.2081, found 488.2071; The ee was determined by **HPLC** analysis (Daicel Chiralcel OD-3 column, hexane–*i*-PrOH = 90: 10, flow rate = 1.0 mL/min) t_R= 23.3 (major enantiomer), 30.1 (minor) min.

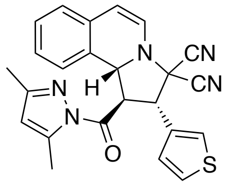

(1*R*,2*S*,10b*R*)-(+)-1-(3,5-Dimethyl-1*H*-pyrazole-1-carbonyl)-2-(thiophen-3-yl)-1,10b-dihydropyrrolo[2,1-*a*]isoquinoline-3,3(2*H*)-dicarbonitrile (3ah): 96% yield; Yellow form;

[α]^{30}D +556.2 (99% ee, *c* 0.79, $CHCl_3$); **^{1}H NMR** (400 MHz, $CDCl_3$) δ 7.70–7.63 (m, 1H), 7.39–7.29 (m, 2H), 7.19 (t, *J* = 7.4 Hz, 1H), 7.12–7.01 (m, 2H), 6.84 (d, *J* = 8.0 Hz, 1H), 6.38 (d, *J* = 7.2 Hz, 1H), 6.05 (s, 1H), 5.99 (d, *J* = 7.2 Hz, 1H), 5.71 (dd, *J* = 8.8, 11.2 Hz, 1H), 5.44 (d, *J* = 8.4 Hz, 1H), 4.69 (d, *J* = 11.6 Hz, 1H), 2.52 (s, 3H), 2.28 (s, 3H); **^{13}C NMR** (100 MHz, $CDCl_3$) δ 170.5, 137.7, 144.9, 131.5, 131.1, 128.9, 128.4, 127.9, 127.4, 127.1, 126.8, 126.2, 125.1, 124.3, 113.2, 113.0, 112.2, 110.8, 63.6, 61.6, 55.0, 50.7, 14.6, 14.0; **HRMS** (ESI+) calcd for $C_{24}H_{20}N_5OS$ $[M+H]^+$ 426.1383, found 426.1362. The ee was determined by **HPLC** analysis (Daicel Chiralcel AD-3 column, hexane–*i*-PrOH = 98.4: 1.6, flow rate = 1.0 mL/min) t_R= 27.7 (major enantiomer), 35.4 (minor) min.

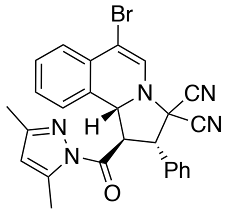

(1*R*,2*S*,10b*R*)-(+)-6-Bromo-1-(3,5-dimethyl-1*H*-pyrazole-1-carbonyl)-2-phenyl-1,10b-dihydropyrrolo[2,1-*a*]isoquinoline-3,3(2*H*)-dicarbonitrile (3ba) (Table 2): 98% yield; Yellow form; **[α]^{30}D** +500.2 (>99% ee, *c* 0.92, $CHCl_3$); **^{1}H NMR** (400 MHz, $CDCl_3$) δ 7.65–7.55 (m, 2H), 7.54–7.47 (m, 2H), 7.43–7.29 (m, 4H), 7.26–7.16 (m, 1H), 6.98 (d, *J* = 7.2 Hz, 1H), 6.77 (d, *J* = 3.6 Hz, 1H), 6.00 (s, 1H), 5.82–5.72 (m, 1H), 5.50 (d, *J* = 8.4, 11.2 Hz, 1H), 4.53 (dd, *J* = 4.0, 11.2 Hz, 1H), 2.48 (s, 3H), 2.24 (s, 3H); **^{13}C NMR** (100 MHz, $CDCl_3$) δ 170.3, 153.7, 144.8, 130.2, 130.1, 129.8, 129.3, 129.1, 128.7, 127.6, 125.3, 124.2, 113.0, 112.5, 111.9, 106.4, 63.4, 61.5, 59.5, 49.9, 14.5, 13.9; **HRMS** (ESI+) calcd for $C_{26}H_{20}BrN_5NaO$ $[M+Na]^+$ 520.0743, found 520.0764; The ee was determined by **HPLC** analysis (Daicel Chiralcel AD-3 column, hexane–*i*-PrOH = 98.4: 1.6, flow rate = 1.0 mL/min) t_R= 14.4 (major enantiomer),25.3(minor) min.

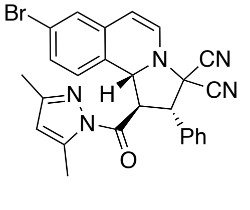

(1*R*,2S,10b*R*)-(+)-8-Bromo-1-(3,5-dimethyl-1*H*-pyrazole-1-carbonyl)-2-phenyl-1,10b-dihydropyrrolo[2,1-*a*]isoquinoline-3,3(2*H*)-dicarbonitrile (3ca) (Table 2): 94% yield; Yellow form; **[α]^{30}D** +483.8 (>99% ee, *c* 0.80, $CHCl_3$); **^{1}H NMR** (400 MHz, $CDCl_3$) δ 7.65–7.56 (m, 2H), 7.42–7.35 (m, 3H), 7.25–7.18 (m, 2H), 6.76 (d, *J* = 8.0 Hz, 1H), 6.44 (d, *J* = 7.6 Hz, 1H), 6.01 (s, 1H), 5.92 (d, *J* = 7.2 Hz, 1H), 5.73 (dd, *J* = 8.4, 11.2 Hz, 1H), 5.39 (d, *J* = 8.4 Hz, 1H), 4.53 (d, *J* = 11.6 Hz, 1H), 2.48 (s, 3H), 2.24 (s, 3H); **^{13}C NMR** (100 MHz, $CDCl_3$) δ 170.2, 153.7, 144.8, 133.3, 130.5, 130.4, 130.0, 129.2, 129.1, 128.2, 127.7, 127.6, 126.0, 122.4, 113.0, 112.9, 111.9, 109.4, 63.2, 61.7, 59.5, 49.8, 14.5, 13.9; **HRMS** (ESI+) calcd for $C_{26}H_{20}BrN_5NaO$ $[M+Na]^+$ 520.0743, found 520.0774; The ee was determined by **HPLC** analysis (Daicel Chiralcel AD-3 column, hexane–*i*-PrOH = 98.4: 1.6, flow rate = 1.0 mL/min) t_R= 15.7 (major enantiomer), 21.4 (minor) min.

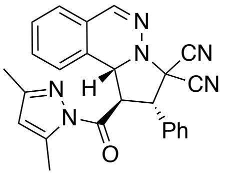

(1*R*,2*S*,10b*R*)-(+)-1-(3,5-dimethyl-1*H*-pyrazole-1-carbonyl)-2-phenyl-1,10b-dihydropyrrolo[2,1-*a*]phthalazine-3,3(2*H*)-dicarbonitrile (3da) (Table 2): 98% yield; White solid; **[α]^{30}$_{D}$** +262.2 (>99% ee, *c* 0.79, $CHCl_3$); **^{1}H NMR** (400 MHz, $CDCl_3$) δ 7.82 (s, 1H), 7.46–7.32 (m, 8H), 7.07–7.00 (m, 1H), 6.01 (s, 1H), 5.21 (dd, *J* = 7.6, 9.2 Hz, 1H), 5.09 (d, *J* = 8.4, 9.2 Hz, 1H), 4.35 (d, *J* = 7.6 Hz, 1H),2.56 (d, *J* = 1.2 Hz, 3H), 2.04 (s, 3H); **^{13}C NMR** (100 MHz, $CDCl_3$) δ 170.3, 153.4, 146.2, 144.7, 135.8, 132.7, 132.2, 129.4, 129.0, 128.9, 126.3, 125.3, 124.5, 123.9, 113.4, 112.7, 111.8, 62.3, 59.9, 57.9, 50.2, 14.4, 13.6; **HRMS** (ESI+) calcd for $C_{25}H_{20}N_6NaO$ $[M+Na]^+$ 443.1591, found 443.1602; The ee was determined by **HPLC** analysis (Daicel Chiralcel AD-3 column, hexane–*i*-PrOH = 95: 5, flow rate = 1.0 mL/min) t_R= 18.4 (major enantiomer), 22.6 (minor) min.

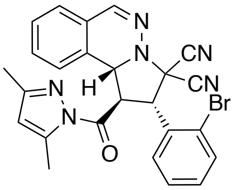

(1*R*,2*S*,10b*R*)-(+)-2-(2-Bromophenyl)-1-(3,5-dimethyl-1*H*-pyrazole-1-carbonyl)-1,10b-dihydropyrrolo[2,1-*a*]phthalazine-3,3(2*H*)-dicarbonitrile (3de) (Table 2): 97% yield; White solid; **[α]^{29}$_{D}$** +116.2 (98% ee, *c* 0.75, $CHCl_3$); **^{1}H NMR** (400 MHz, $CDCl_3$) δ 7.88 (s, 1H), 7.62 (dd, *J* = 1.0, 8.2 Hz, 1H), (dd, *J* = 1.4, 7.8 Hz, 1H), 7.50–7.38 (m, 3H), 7.38–7.30 (m, 1H), 7.25–7.18 (m, 1H), 7.11–7.02 (m, 1H), 6.01 (s, 1H), 5.17–5.09 (m, 1H), 4.99–4.91 (m, 1H), 2.59 (s, 3H), 1.94 (s, 3H); **^{13}C NMR** (100 MHz, $CDCl_3$) δ 169.8, 153.5, 147.3, 144.7, 137.1, 133.3, 132.8, 132.3, 130.4, 129.2, 129.0, 128.3, 126.4, 126.0, 125.4, 123.8, 112.8, 112.5, 111.3, 61.3, 58.8, 55.6, 50.7, 14.3, 13.5; **HRMS** (ESI+) calcd for $C_{25}H_{19}BrN_6NaO$ $[M+Na]^+$ 521.0696, found 521.0712;The ee was determined by **HPLC** analysis (Daicel Chiralcel AD-3 column, hexane–*i*-PrOH = 90: 10, flow rate = 1.0 mL/min) t_R= 11.2 (major enantiomer), 28.1 (minor) min.

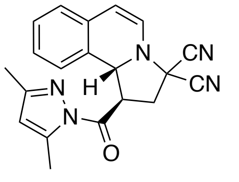

(1*R*,10b*R*)-(+)-1-(3,5-Dimethyl-1*H*-pyrazole-1-carbonyl)-1,10b-dihydropyrrolo[2,1-*a*]isoquinoline-3,3(2*H*)-dicarbonitrile (3ai) (Table 2): 98% yield; Yellow form; **[α]^{30}$_{D}$** +92.3 (>99% ee, *c* 0.91, $CHCl_3$); **^{1}H NMR** (400 MHz, $CDCl_3$) δ 7.21 (t, *J* = 7.6 Hz, 1H), 7.16–7.09 (m, 1H), 7.09–7.02 (m, 1H), 6.84 (d, *J* = 8.0 Hz, 1H), 6.44 (d, *J* = 7.6 Hz, 1H), 6.07 (d, *J* = 1.2 Hz, 1H), 6.00 (d, *J* = 7.2 Hz, 1H), 5.31 (d, *J* = 9.2 Hz, 1H), 4.89 (q, *J* = 9.2 Hz, 1H), 3.43 (dd, *J* = 9.4, 13.4 Hz, 1H), 2.94 (dd, *J* = 9.4, 13.4 Hz, 1H), 2.60 (s, 3H), 2.26 (s, 3H); **^{13}C NMR** (100 MHz, $CDCl_3$) δ 170.5, 153.8, 144.8, 131.2, 129.5, 128.3, 127.8, 127.6, 124.9, 124.4, 113.6, 113.4, 112.5,

110.9,61.3, 53.4, 46.6, 42.3, 36.0, 14.5, 14.0; **HRMS** (ESI+) calcd for $C_{20}H_{17}N_5NaO$ $[M+Na]^+$ 366.1325, found 366.1334; The ee was determined by **HPLC** analysis (Daicel Chiralcel AD-3 column, hexane–*i*-PrOH = 98.4: 1.6, flow rate = 1.0 mL/min) t_R= 23.1 (minor enantiomer), 26.1 (major) min.

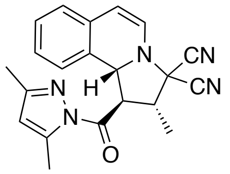

(1*R*,2*R*,10b*R*)-(+)-1-(3,5-Dimethyl-1*H*-pyrazole-1-carbonyl)-2-methyl-1,10b-dihydropyrrolo[2,1-*a*]isoquinoline-3,3(2*H*)-dicarbonitrile (3aj) (Table 2): 97% yield; Yellow form; **[α]^{30}$_D$** +460.1 (>99% ee, *c* 0.67, $CHCl_3$); **^{1}H NMR** (400 MHz, $CDCl_3$) δ 7.17 (t, *J* = 7.4 Hz, 1H), 7.11–6.99 (m, 2H), 6.69 (d, *J* = 7.6 Hz, 1H), 6.38 (d, *J* = 7.2 Hz, 1H), 6.12 (s, 1H), 5.96 (d, *J* = 7.2 Hz, 1H), 5.38 (d, *J* = 8.8 Hz, 1H), 4.97 (q, *J* = 8.8, 10.4 Hz, 1H), 3.38–3.28 (m, 1H), 2.61 (s, 3H), 2.27 (s, 3H); 1.47 (d, *J* = 6.8 Hz, 3H); **^{13}C NMR** (100 MHz, $CDCl_3$) δ 170.8, 153.7, 144.8, 131.0, 129.2, 128.2, 127.8, 127.1, 124.9, 123.9, 113.2, 113.0, 112.1, 110.6, 63.0, 60.7, 52.7, 49.8, 14.6, 14.5, 14.0; **HRMS** (ESI+) calcd for $C_{21}H_{20}N_5O$ $[M+H]^+$ 358.1662, found 358.1649; The ee was determined by **HPLC** analysis (Daicel Chiralcel OD-3 column, hexane–*i*-PrOH = 98: 2, flow rate = 1.0 mL/min) t_R= 9.2 (major enantiomer), 17.3 (minor) min.

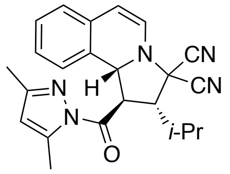

(1*R*,2*R*,10b*R*)-(+)-1-(3,5-Dimethyl-1*H*-pyrazole-1-carbonyl)-2-isopropyl-1,10b-dihydropyrrolo[2,1-*a*]isoquinoline-3,3(2*H*)-dicarbonitrile (3ak) (Table 2): 86% yield; Yellow form; **[α]^{27}$_D$** +262.9 (94% ee, *c* 0.87, $CHCl_3$); **^{1}H NMR** (400 MHz, $CDCl_3$) δ 7.22–7.13 (m, 2H), 7.12–7.05 (m, 1H), 7.03 (d, *J* = 7.6 Hz, 1H), 6.42 (d, *J* = 7.2 Hz, 1H), 6.10 (s, 1H), 6.07 (d, *J* = 7.6 Hz, 1H), 5.14–5.06 (m, 2H), 3.39–3.29 (m, 1H), 2.58 (s, 3H), 2.31 (s, 3H), 2.29–2.18 (m, 1H), 1.22 (d, *J* = 6.4 Hz, 3H), 0.93 (d, *J* = 6.8 Hz, 3H); **^{13}C NMR** (100 MHz, $CDCl_3$) δ 172.4, 153.6, 145.0, 131.3, 129.1, 128.2, 127.9, 127.1, 124.9, 124.5, 114.1, 112.9, 112.5, 112.4, 65.0, 61.0, 58.8, 50.1, 31.0, 22.2, 20.2, 14.7, 14.0; **HRMS** (ESI+) calcd for $C_{23}H_{23}N_5NaO$ $[M+Na]^+$ 408.1789, found 408.1797; The ee was determined by **HPLC** analysis (Daicel Chiralcel AS-3 column, hexane–*i*-PrOH = 99: 1, flow rate = 1.0 mL/min) t_R= 9.2 (minor enantiomer), 13.4 (major) min.

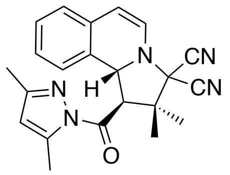

(1*R*,10b*R*)-(+)-1-(3,5-Dimethyl-1*H*-pyrazole-1-carbonyl)-2,2-dimethyl-1,10b-dihydropyrrolo[2,1-*a*]isoquinoline-3,3(2*H*)-dicarbonitrile (3al) (Table 2): 97% yield; Yellow form; **[α]^{25}$_D$** +283.2 (>99% ee, *c* 0.87, $CHCl_3$); **^{1}H NMR** (400 MHz, $CDCl_3$) δ 7.21–7.14 (m, 1H), 7.13–7.00 (m, 2H), 6.63 (d, *J* = 7.6 Hz, 1H), 6.42 (d, *J* = 7.6 Hz, 1H), 6.10 (s, 1H), 6.01 (d, *J* = 7.2

Hz, 1H), 5.40 (d, J = 9.2 Hz, 1H), 5.01 (d, J = 9.2 Hz, 1H), 2.61 (s, 3H), 2.27 (s, 3H); 1.57 (s, 3H), 1.36 (s, 3H); **^{13}C NMR** (100 MHz, $CDCl_3$) δ 169.7, 153.3, 144.7, 131.1, 130.0, 128.2, 127.9, 127.8, 125.0, 123.6, 112.6, 111.9, 111.0, 66.5, 60.9, 54.8, 50.9, 25.8, 21.5, 14.6, 14.0; **HRMS** (ESI+) calcd for $C_{22}H_{21}N_5NaO$ $[M+Na]^+$ 394.1638, found 394.1641; The ee was determined by **HPLC** analysis (Daicel Chiralcel AD-3 column, hexane–*i*-PrOH = 98.4: 1.6, flow rate = 1.0 mL/min) t_R= 8.6 (major enantiomer), 11.2 (minor) min.

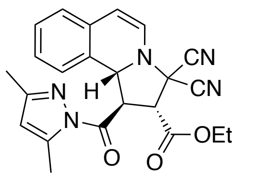

Ethyl (1*R*,2*R*,10b*R*)-(+)-3,3-dicyano-1-(3,5-dimethyl-1*H*-pyrazole-1-carbonyl)-1,2,3,10b-tetrahydropyrrolo[2,1-*a*]isoquinoline-2-carboxylate (3am) (Table 2): 97% yield; Yellow solid; **[α]$^{30}_{D}$** +338.2 (>99% ee, *c* 0.85, $CHCl_3$); **^{1}H NMR** (400 MHz, $CDCl_3$) δ 7.26–7.19 (m, 1H), 7.19–7.02 (m, 3H), 6.41 (d, J = 7.2 Hz, 1H), 6.13–6.08 (m, 2H), 5.36 (dd, J = 8.4, 9.6 Hz, 1H), 5.26 (d, J = 8.4 Hz, 1H), 4.32 (d, J = 10.4 Hz, 1H), 4.30–4.22 (m, 2H), 2.61 (s, 3H), 2.26 (s, 3H); 1.21 (d, J = 7.2 Hz, 3H); **^{13}C NMR** (100 MHz, $CDCl_3$) δ 170.1, 166.2, 153.5, 144.9, 130.8, 128.7, 128.5, 128.0, 126.6, 125.1, 124.6, 112.7, 112.7, 112.4, 111.8, 63.1, 62.9, 57.4, 56.2, 48.5, 14.5, 13.9, 13.7; **HRMS** (FAB+) calcd for $C_{23}H_{21}N_5NaO_3$ $[M+Na]^+$ 438.1537, found 538.1557; The ee was determined by **HPLC** analysis (Daicel Chiralcel OD-3 column, hexane–*i*-PrOH = 98: 2, flow rate = 1.0 mL/min) t_R= 14.5 (major enantiomer), 29.0 (minor) min.

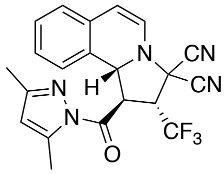

(1*R*,2*R*,10b*R*)-(+)-1-(3,5-Dimethyl-1*H*-pyrazole-1-carbonyl)-2-(trifluoromethyl)-1,10b-dihydropyrrolo[2,1-*a*]isoquinoline-3,3(2*H*)-dicarbonitrile (3an) (Table 2): 99% yield; Yellow foam; **[α]$^{26}_{D}$** +339.7 (>99% ee, *c* 0.93, $CHCl_3$); **^{1}H NMR** (400 MHz, $CDCl_3$) δ 7.28–7.21 (m, 1H), 7.21–7.11 (m, 2H), 7.11–7.07 (m, 1H), 6.42 (d, J = 7.2 Hz, 1H), 6.19 (d, J = 7.2 Hz, 1H), 6.11 (s, 1H), 5.37 (dd, J = 7.4, 8.8 Hz, 1H), 5.21 (d, J = 7.6 Hz, 1H), 4.40–4.29 (m, 1H), 2.59 (s, 3H), 2.26 (s, 3H); **^{13}C NMR** (100 MHz, $CDCl_3$) δ 169.1, 154.3, 145.0, 130.6, 128.8, 128.4, 128.1, 126.1, 125.4, 124.4, 114.3, 113.2, 112.0, 110.1, 64.1(2C), 56.3 (q, J_{CF} = 29.6 Hz), 54.4, 46.7, 14.4, 13.9; **^{19}F NMR** (376 MHz, $CDCl_3$) δ –66.3; **HRMS** (ESI+) calcd for $C_{21}H_{16}F_3N_5NaO$ $[M+Na]^+$ 434.1199, found 434.1198; The ee was determined by **HPLC** analysis (Daicel Chiralcel AD-3 column, hexane–*i*-PrOH = 98.4: 1.6, flow rate = 1.0 mL/min) t_R= 16.6 (major enantiomer), 19.7 (minor) min.

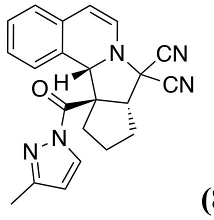

(8a*R*,11a*R*,11b*S*)-(–)-11a-(3-Methyl-1H-pyrazole-1-carbonyl)-8a,9,10,11,11a,11b-hexahydro-8H-cyclopenta[3,4]pyrrolo[2,1-*a*]isoquinoline-8,8-dicarbonitrile (**3ap**) **(Table 3)**: 97% yield; Yellow form; **[α]$^{26}_{D}$** –127.2 (99% ee, *c* 0.88, $CHCl_3$); **^{1}H NMR** (400 MHz, $CDCl_3$) δ 8.21 (d, *J* = 2.8 Hz, 1H), 7.16 (t, *J* = 7.6 Hz, 1H), 7.12–7.05 (m, 1H), 6.99 (d, *J* = 7.2 Hz, 1H), 6.94 (d, *J* = 7.2 Hz, 1H), 6.48 (d, *J* = 7.6 Hz, 1H), 6.26 (d, *J* = 3.2 Hz, 1H), 6.00 (s, 1H), 5.79 (d, *J* = 7.6 Hz, 1H), 4.38–4.30 (m, 1H), 2.99–2.88 (m, 1H), 2.37 (s, 3H), 2.33–2.20 (m, 1H), 2.20–2.10 (m, 2H), 1.92–1.72 (m, 2H); **^{13}C NMR** (100 MHz, $CDCl_3$) δ 170.6, 155.1, 132.3, 130.8, 129.6, 128.9, 128.2, 127.2, 125.1, 125.0, 114.5, 112.7, 110.9, 108.9, 67.3, 63.9, 58.8, 57.3, 33.0, 32.5, 25.9, 13.8; **HRMS** (ESI+) calcd for $C_{22}H_{19}N_5NaO$ $[M+Na]^+$ 392.1482, found 392.1490; The ee was determined by **HPLC** analysis (Daicel Chiralcel OD-3 column, hexane–*i*-PrOH = 98.0: 2.0, flow rate = 1 mL/min) t_R= 14.0 (major enantiomer), 20.1 (minor) min.

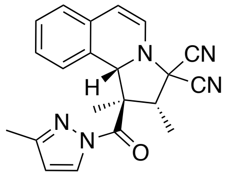

(1*R*,2*R*,10b*S*)-(+)-1,2-Dimethyl-1-(3-methyl-1*H*-pyrazole-1-carbonyl)-1,10b-dihydropyrrolo[2,1-*a*]isoquinoline-3,3(2*H*)-dicarbonitrile (3aq) (Table 3): 93% yield; Yellow form; **[α]$^{26}_{D}$** +270.5 (99% ee, *c* 0.98, $CHCl_3$); **^{1}H NMR** (400 MHz, $CDCl_3$) δ 7.27 (d, *J* = 2.8 Hz, 1H), 7.17–7.05 (m, 1H), 7.03–6.90 (m, 2H), 6.75 (s, 1H), 6.51 (d, *J* = 7.6 Hz, 1H), 6.40 (d, *J* = 7.6 Hz, 1H), 6.28 (d, *J* = 2.8 Hz, 1H), 5.58 (d, *J* = 8.0 Hz, 1H), 4.90–4.80 (m, 1H), 2.33 (s, 3H), 1.43 (s, 3H), 1.37 (d, *J* = 7.6 Hz, 3H); **^{13}C NMR** (100 MHz, $CDCl_3$) δ 169.5, 154.9, 132.0, 131.5, 128.3, 128.1, 127.9, 126.9, 125.4, 124.4, 114.7, 112.4, 110.5, 105.0, 66.8, 58.4, 57.7, 49.5, 14.2, 13.6, 11.0; **HRMS**: the compound is too unstable to undergo the conditions of the mass spectrometer; The ee was determined by **HPLC** analysis (Daicel Chiralcel OD-3 column, hexane–*i*-PrOH = 98: 2, flow rate = 1 mL/min) t_R= 11.1 (major enantiomer), 12.8 (minor) min.

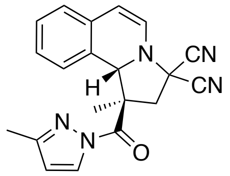

(1*R*,10b*S*)-(–)-1-Methyl-1-(3-methyl-1H-pyrazole-1-carbonyl)-1,10b-dihydropyrrolo[2,1-*a*]isoquinoline-3,3(2*H*)-dicarbonitrile (3ar) (Table 3): 94% yield; Yellow form; **[α]$^{29}_{D}$** –126.0 (89% ee, *c* 0.93, $CHCl_3$); **^{1}H NMR** (400 MHz, $CDCl_3$) δ 8.22 (d, *J* = 2.8 Hz, 1H), 7.18 (t, *J* = 7.4 Hz, 1H), 7.09 (t, *J* = 7.6 Hz, 1H), 7.06–6.98 (m, 2H), 6.45 (d, *J* = 7.6 Hz, 1H), 6.29 (d, *J* = 2.8 Hz, 1H), 5.83 (s, 1H), 5.74 (d, *J* = 7.6 Hz, 1H), 3.59 (d, *J* = 14.0 Hz, 1H), 3.26 (d, *J* = 14.0 Hz,

1H), 2.35 (s, 3H), 1.77 (s, 3H); **^{13}C NMR** (100 MHz, $CDCl_3$) δ 170.7, 155.0, 132.4, 130.8, 128.3, 127.9, 126.9, 126.1, 125.3,114.2, 114.1, 110.7, 107.5, 66.0, 52.9, 51.7, 48.9, 21.3, 14.1; **HRMS** (ESI+) calcd for $C_{20}H_{17}N_5NaO$ $[M+Na]^+$ 366.1325, found 366.1339; The ee was determined by **HPLC** analysis (Daicel Chiralcel OD-3 column, hexane–*i*-PrOH = 98.0: 2.0, flow rate = 1 mL/min) t_R= 24.5 (major enantiomer), 39.2 (minor) min.

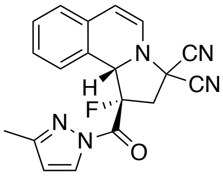

(1*S*,10b*S*)-(+)-1-Fluoro-1-(3-methyl-1*H*-pyrazole-1-carbonyl)-1,10b-dihydropyrrolo[2,1-*a*]isoquinoline-3,3(2*H*)-dicarbonitrile (3as) (Table 3): 97% yield; Yellow form; **[α]^{28}$_D$** +31.9 (>99% ee, *c* 0.84, $CHCl_3$); **^{1}H NMR** (400 MHz, $CDCl_3$) δ 8.28 (d, *J* = 2.8 Hz, 1H), 7.22 (t, *J* = 7.2 Hz, 1H), 7.18–7.02 (m, 2H), 6.94 (d, *J* = 7.2 Hz, 1H), 6.52 (d, *J* = 7.6 Hz, 1H), 6.37 (d, *J* = 2.8 Hz, 1H), 5.95 (d, *J* = 21.2 Hz, 1H), 5.87 (d, *J* = 7.2 Hz, 1H), 4.23–4.08 (m, 1H), 3.31 (dd, *J* = 15.0, 24.6 Hz, 1H), 2.37 (s, 3H); **^{13}C NMR** (100 MHz, $CDCl_3$) δ 163.6 (d, *J* = 26.9 Hz, 1C), 156.6, 131.8, 131.0, 128.8, 127.4 (d, *J* = 14.3 Hz, 1C), 125.5, 124.7, 124.6, 113.6, 113.1, 112.1, 108.8, 67.7, 67.5, 52.2, 48.8, 48.5, 13.9; **^{19}F NMR** (376 MHz, $CDCl_3$) δ –153.6; **HRMS** (ESI+) calcd for $C_{19}H_{15}FN_5O$ $[M+H]^+$ 348.1225, found 348.1251; The ee was determined by **HPLC** analysis (Daicel Chiralcel AD-3 column, hexane–*i*-PrOH = 95: 5, flow rate = 1 mL/min) t_R= 19.4 (major enantiomer), 22.7 (minor) min.

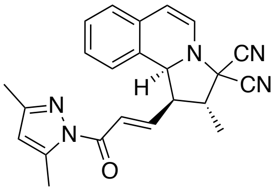

(1*R*,2*R*,10b*S*)-(+)-1-((*E*)-3-(3,5-Dimethyl-1*H*-pyrazol-1-yl)-3-oxoprop-1-en-1-yl)-2-methyl-1,10b-dihydropyrrolo[2,1-*a*]isoquinoline-3,3(2*H*)-dicarbonitrile (*endo*-6aa) (Use of L5, Table 5): 96% yield; Yellow form; **[α]^{30}$_D$** +353.4 (95% ee, *c* 0.75, $CHCl_3$); **^{1}H NMR** (400 MHz, $CDCl_3$) δ 7.73 (d, *J* = 15.6 Hz, 1H), 7.23–7.09 (m, 3H), 7.08–6.99 (m, 2H), 6.34 (d, *J* = 7.6 Hz, 1H), 6.05 (s, 1H), 5.94 (d, *J* = 7.2 Hz, 1H), 5.02 (d, *J* = 8.8 Hz, 1H), 3.36–3.23 (m, 1H), 2.97–2.83 (m, 1H), 2.61 (s, 3H), 2.28 (s, 3H), 1.46 (d, *J* = 6.8 Hz, 3H); **^{13}C NMR** (100 MHz, $CDCl_3$) δ 163.9, 152.7, 145.2, 144.7, 131.0, 129.5, 128.4, 127.8, 126.7, 126.0, 125.1, 123.7, 113.4, 112.3, 112.1, 110.2, 62.9, 60.3, 54.2, 49.3, 14.7, 13.9, 13.0; **HRMS** (ESI+) calcd for $C_{23}H_{21}N_5NaO$ $[M+Na]^+$ 406.1638, found 406.1626; The ee was determined by **HPLC** analysis (Daicel Chiralcel AD-3, hexane–*i*-PrOH = 98.4: 1.6, flow rate = 1.0 mL/min) t_R= 22.9 (minor enantiomer), 23.9 (major) min.

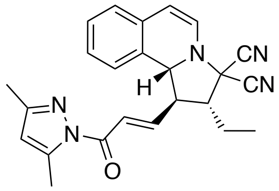

(1*R*,2*R*,10b*R*)-(+)-1-((*E*)-3-(3,5-dimethyl-1*H*-pyrazol-1-yl)-3-oxoprop-1-en-1-yl)-2-ethyl-1,10b-dihydropyrrolo[2,1-*a*]isoquinoline-3,3(2*H*)-dicarbonitrile (*exo*-6ab) (Use of L5, Table 5): 54% yield; Yellowish form; **[α]^{25}$_D$** +380.7 (>99% ee, *c* 0.50, $CHCl_3$); **^{1}H NMR** (400 MHz, $CDCl_3$) δ 7.73 (d, *J* = 15.6 Hz, 1H), 7.24–6.98 (m, 5H), 6.37 (d, *J* = 7.6 Hz, 1H), 6.05 (s, 1H), 5.93 (d, *J* = 7.6 Hz, 1H), 4.99 (d, *J* = 8.8 Hz, 1H), 3.39–3.26 (m, 1H), 2.86–2.72 (m, 1H), 2.62 (s, 3H), 2.28 (s, 3H), 2.06–1.91 (m, 1H), 1.90–1.76 (m, 1H), 1.22 (t, *J* = 7.6 Hz, 3H); **^{13}C NMR** (100 MHz, $CDCl_3$) δ 163.9, 152.7, 145.8, 144.7, 131.2, 129.6, 128.4, 127.7, 126.7, 126.0, 125.0, 123.7, 114.0, 112.2, 112.1, 109.9, 62.8, 58.5, 55.9, 53.4, 22.9, 14.7, 13.9; 12.3; **HRMS** (FAB+) calcd for $C_{24}H_{23}N_5NaO$ $[M+Na]^+$ 420.1800, found 420.1811; The ee was determined by **HPLC** analysis (Daicel Chiralcel IA-3, hexane–*i*-PrOH = 99: 1, flow rate = 1.0 mL/min) t_R= 21.9 (minor enantiomer), 26.5 (major) min.

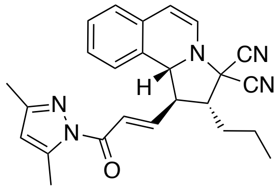

(1*R*,2*R*,10b*R*)-(+)-1-((*E*)-3-(3,5-Dimethyl-1*H*-pyrazol-1-yl)-3-oxoprop-1-en-1-yl)-2-propyl-1,10b-dihydropyrrolo[2,1-*a*]isoquinoline-3,3(2*H*)-dicarbonitrile (Use of L5, *exo*-6ac) (Table 5): 78% yield; Brown form; **[α]^{26}$_D$** +330.0 (81% ee, *c* 0.98, $CHCl_3$); **^{1}H NMR** (400 MHz, $CDCl_3$) δ 7.72 (d, *J* = 16.0 Hz, 1H), 7.22–7.09 (m, 3H), 7.09–6.99 (m, 2H), 6.37 (d, *J* = 7.2 Hz, 1H), 6.05 (s, 1H), 5.92 (d, *J* = 7.6 Hz, 1H), 4.98 (d, *J* = 8.8 Hz, 1H), 3.40–3.25 (m, 1H), 2.92–2.80 (m, 1H), 2.62 (s, 3H), 2.29 (s, 3H), 1.91–1.49 (m, 4H), 1.02 (t, *J* = 7.0 Hz, 3H); **^{13}C NMR** (100 MHz, $CDCl_3$) δ 163.9, 152.7, 145.8, 144.7, 131.2, 129.6, 128.3, 127.7, 126.7, 126.0, 125.0, 123.7, 113.9, 112.2, 112.1, 109.9, 62.7, 58.7, 54.1, 53.4, 31.7, 21.0, 14.7, 14.1, 13.9; **HRMS** (ESI+) calcd for $C_{25}H_{25}N_5NaO$ $[M+Na]^+$ 434.1951, found 434.1965; The ee was determined by **HPLC** analysis (Daicel Chiralcel IA-3, hexane–*i*-PrOH = 99: 1, flow rate = 1.0 mL/min) t_R= 21.7 (minor enantiomer), 23.2 (major) min.

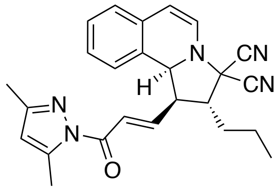

(1*R*,2*R*,10b*S*)-(+)-1-((*E*)-3-(3,5-dimethyl-1*H*-pyrazol-1-yl)-3-oxoprop-1-en-1-yl)-2-propyl-1,10b-dihydropyrrolo[2,1-*a*]isoquinoline-3,3(2*H*)-dicarbonitrile (*endo*-6ac) (Use of L7, Table 5): 82% yield; 38% ee; **^{1}H NMR** (400 MHz, $CDCl_3$) δ 7.46 (d, *J* = 15.6 Hz, 1H), 7.27–7.10 (m, 2H), 7.09–6.97 (m, 2H), 6.88 (d, *J* = 7.2 Hz, 1H), 6.44 (d, *J* = 7.6 Hz, 1H), 6.00 (s, 1H), 5.84

(d, J = 7.6 Hz, 1H), 4.92 (d, J = 6.0 Hz, 1H), 3.32–3.20 (m, 1H), 2.72–2.62 (m, 1H), 2.53 (s, 3H), 2.29 (s, 3H), 1.99–1.81 (m, 2H), 1.68–1.51 (m, 2H), 1.05 (t, J = 6.8 Hz, 3H); **^{13}C NMR** (100 MHz, $CDCl_3$) δ 163.9, 152.3, 148.1, 144.7, 131.9, 128.4, 128.0, 127.9, 127.3, 126.1, 125.4, 122.8, 113.4, 111.8, 111.3, 109.6, 61.7, 58.1, 56.0, 48.9, 33.9, 21.2, 14.7, 14.0; **HRMS** (ESI+) calcd for $C_{25}H_{25}N_5NaO$ $[M+Na]^+$ 434.1951, found 434.1972; The ee was determined by **HPLC** analysis (Daicel Chiralcel IA-3, hexane–*i*-PrOH = 98.4: 1.6, flow rate = 1.0 mL/min) t_R= 18.2 (major enantiomer), 24.5 (minor) min.

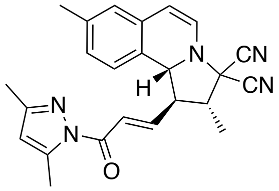

(1*R*,2*R*,10b*R*)-(+)-1-((*E*)-3-(3,5-Dimethyl-1*H*-pyrazol-1-yl)-3-oxoprop-1-en-1-yl)-2,8-dimethyl-1,10b-dihydropyrrolo[2,1-*a*]isoquinoline-3,3(2*H*)-dicarbonitrile (*exo*-6ea) (Use of L5, Table 5): 88% yield; Yellow form; **$[\alpha]^{24}_D$** +353.0 (89% ee, *c* 0.75, $CHCl_3$); **^{1}H NMR** (400 MHz, $CDCl_3$) δ 7.71 (d, J = 15.6 Hz, 1H), 7.17 (dd, J = 9.2, 16.0 Hz, 1H), 6.92 (s, 2H), 6.83 (s, 1H), 6.32 (d, J = 7.6 Hz, 1H), 6.05 (s, 1H), 5.90 (d, J = 7.2 Hz, 1H), 4.98 (d, J = 8.8 Hz, 1H), 3.31–3.21 (m, 1H), 2.94–2.81 (m, 1H), 2.61 (s, 3H), 2.28 (s, 3H). 2.27 (s, 3H), 1.45 (d, J = 6.8 Hz, 3H); **^{13}C NMR** (100 MHz, $CDCl_3$) δ 163.9, 152.7, 145.3, 144.7, 138.0, 130.8, 128.4, 126.8, 126.7, 125.9, 125.8, 123.6, 113.4, 112.3, 112.1, 110.3, 62.8, 60.3, 54.4, 49.3, 21.1, 14.7, 13.9, 13.0; **HRMS** (FAB+) calcd for $C_{24}H_{23}N_5NaO$ $[M+Na]^+$ 420.1800, found 420.1790; The ee was determined by **HPLC** analysis (Daicel Chiralcel AD-3 column, hexane–*i*-PrOH = 98.4: 1.6, flow rate = 1 mL/min) t_R= 15.6 (minor enantiomer), 17.5 (major) min.

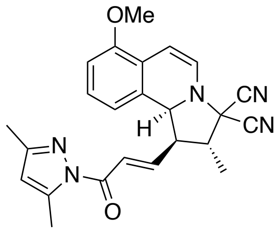

(1*R*,2*R*,10b*S*)-1-((*E*)-3-(3,5-Dimethyl-1H-pyrazol-1-yl)-3-oxoprop-1-en-1-yl)-7-methoxy-2-methyl-1,10b-dihydropyrrolo[2,1-*a*]isoquinoline-3,3(2*H*)-dicarbonitrile (*endo*-6fa) (Use of L5, Table 5): 73% yield; Yellow form; **$[\alpha]^{27}_D$** +71.4 (84% ee, *c* 0.98, $CHCl_3$); **^{1}H NMR** (400 MHz, $CDCl_3$) δ 7.47 (d, J = 15.6 Hz, 1H), 7.22 (dd, J = 10.0, 15.6 Hz, 1H), 7.08–6.99 (m, 1H), 6.71 (d, J = 8.4 Hz, 1H), 6.47 (d, J = 7.2 Hz, 1H), 6.41 (d, J = 7.6 Hz, 1H), 6.21 (d, J = 7.6 Hz, 1H), 6.00 (s, 1H), 4.99 (d, J = 6.4 Hz, 1H), 3.82 (s, 3H), 3.28–3.18 (m, 1H), 2.85–2.73 (m, 1H), 2.53 (s, 3H), 2.28 (s, 3H), 1.55 (d, J = 6.8 Hz, 3H); **^{13}C NMR** (100 MHz, $CDCl_3$) δ 163.8, 154.2, 152.3, 147.5, 144.7, 129.0, 128.2, 126.9, 123.0, 121.1, 118.3, 113.3, 111.7, 111.4, 110.3, 103.4, 61.5, 59.0, 55.7, 51.1, 50.5, 16.1, 14.7, 13.9; **HRMS** (ESI+) calcd for $C_{24}H_{23}N_5NaO_2$ $[M+Na]^+$ 436.1744, found 436.1730; The ee was determined by **HPLC** analysis (Daicel Chiralcel OD-3 column, hexane–*i*-PrOH

= 98: 2, flow rate = 1 mL/min) t_R= 30.6 (major enantiomer), 33.0 (minor) min.

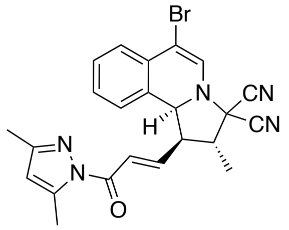

(1*R*,2*R*,10b*S*)-(–)-6-Bromo-1-((*E*)-3-(3,5-dimethyl-1H-pyrazol-1-yl)-3-oxoprop-1-en-1-yl)-2-methyl-1,10b-dihydropyrrolo[2,1-*a*]isoquinoline-3,3(2*H*)-dicarbonitrile (*endo*-6ba) (Use of L5, Table 5): 54% yield; Brown form; **[α]^{24}$_D$** –8.0 (97% ee, *c* 0.70, $CHCl_3$); **^{1}H NMR** (400 MHz, $CDCl_3$) δ 7.51 (d, *J* = 15.6 Hz, 1H), 7.48–7.43 (m, 1H), 7.30–7.23 (m, 1H), 7.21–7.11 (m, 2H), 6.86 (d, *J* = 7.2 Hz, 1H), 6.80 (s, 1H), 6.01 (d, *J* = 1.2 Hz, 1H), 5.08 (d, *J* = 6.4 Hz, 1H), 3.33–3.24 (m, 1H), 2.88–2.78 (m, 1H), 2.53 (s, 3H), 2.28 (s, 3H). 1.56 (t, *J* = 7.2 Hz, 3H); **^{13}C NMR** (100 MHz, $CDCl_3$) δ 163.6, 152.5, 146.4, 144.8, 130.8, 128.7, 128.6, 127.7, 125.9, 125.8, 123.6, 112.9, 111.9, 111.1, 103.1, 61.5, 58.7, 51.2, 50.3, 16.1, 14.6, 13.9; **HRMS** (ESI+) calcd for $C_{23}H_{20}BrN_5NaO$ $[M+Na]^+$ 484.0743, found 484.0759; The ee was determined by **HPLC** analysis (Daicel Chiralcel AD-3 column, hexane–*i*-PrOH = 98.4: 1.6, flow rate = 1 mL/min) t_R= 22.8 (major enantiomer), 28.0 (minor) min.

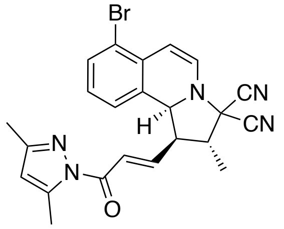

(1*R*,2*R*,10b*S*)-(+)-7-Bromo-1-((*E*)-3-(3,5-dimethyl-1H-pyrazol-1-yl)-3-oxoprop-1-en-1-yl)-2-methyl-1,10b-dihydropyrrolo[2,1-*a*]isoquinoline-3,3(2*H*)-dicarbonitrile (*endo*-6ga) (Use of L5, Table 5): 43% yield; Yellow form; **[α]^{26}$_D$** +104.2 (94% ee, *c* 0.61, $CHCl_3$); **^{1}H NMR** (400 MHz, $CDCl_3$) δ 7.49 (d, *J* = 16.0 Hz, 1H), 7.39 (d, *J* = 8.4 Hz, 1H), 7.18 (dd, *J* = 10.0, 15.6 Hz, 1H), 6.90 (t, *J* = 7.6 Hz, 1H), 6.80 (d, *J* = 7.6 Hz, 1H), 6.52 (d, *J* = 8.0 Hz, 1H), 6.20 (d, *J* = 7.6 Hz, 1H), 6.01 (s, 1H), 5.06 (d, *J* = 6.0 Hz, 1H), 3.31–3.21 (m, 1H), 2.88–2.78 (m, 1H), 2.54 (s, 3H), 2.28 (s, 3H). 1.56 (t, *J* = 7.2 Hz, 3H); **^{13}C NMR** (100 MHz, $CDCl_3$) δ 163.6, 152.5, 146.5, 144.8, 132.6, 131.6, 129.5, 128.1, 125.3, 123.6, 121.1, 113.0, 111.9, 111.2, 107.5, 61.5, 58.7, 51.2, 50.4, 16.2, 14.7, 13.9; **HRMS** (ESI+) calcd for $C_{23}H_{20}BrN_5NaO$ $[M+Na]^+$ 484.0743, found 484.0771; The ee was determined by **HPLC** analysis (Daicel Chiralcel AD-3 column, hexane–*i*-PrOH = 98.4: 1.6, flow rate = 1 mL/min) t_R= 29.2 (major enantiomer), 40.5 (minor) min.

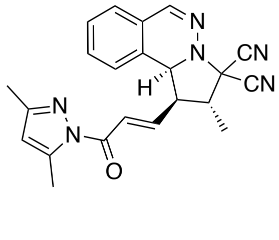

(1*R*,2*R*,10b*S*)-(–)-1-((*E*)-3-(3,5-Dimethyl-1*H*-pyrazol-1-yl)-3-oxoprop-1-

en-1-yl)-2-methyl-1,10b-dihydropyrrolo[2,1-*a*]phthalazine-3,3(2*H*)-dicarbonitrile (*endo*-6da) (Use of L5, Table 5): 42% yield; Brown form; **[α]^{26}$_{D}$** –46.7 (98% ee, *c* 1.5, $CHCl_3$); **^{1}H NMR** (400 MHz, $CDCl_3$) δ 7.71 (s, 1H), 7.51 (d, *J* = 15.6 Hz, 1H), 7.40–7.28 (m, 3H), 7.11 (dd, *J* = 10.8, 15.6 Hz, 1H), 7.08–7.02 (m, 1H), 6.02 (s, 1H), 4.70 (d, *J* = 7.6 Hz, 1H), 3.23–3.13 (m, 1H), 2.82–2.71 (m, 1H), 2.55 (s, 3H), 2.29 (s, 3H). 1.57 (t, *J* = 7.2 Hz, 3H); **^{13}C NMR** (100 MHz, $CDCl_3$) δ 169.9, 165.9, 153.7, 145.0, 129.6, 129.3, 128.7, 124.8, 124.2, 123.1, 120.4, 112.7, 111.9, 111.6, 63.2, 63.0, 57.1, 56.1, 48.2, 14.5, 13.9, 13.7; **HRMS** (ESI+) calcd for $C_{22}H_{20}N_6NaO$ $[M+Na]^+$ 407.1591, found 407.1617; The ee was determined by **HPLC** analysis (Daicel Chiralcel AD-3 column, hexane–*i*-PrOH = 90: 10, flow rate = 1 mL/min) t_R= 14.3 (minor enantiomer),15.3 (major) min.

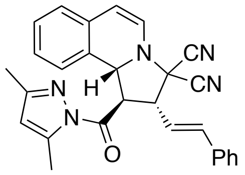

(1*R*,2*R*,10b*R*)-(+)-1-(3,5-Dimethyl-1*H*-pyrazole-1-carbonyl)-2-((*E*)-styryl)-1,10b-dihydropyrrolo[2,1-*a*]isoquinoline-3,3(2*H*)-dicarbonitrile (*exo*-5ad) (Use of L5, Table 5): 63% yield; Yellow form; **[α]^{30}$_{D}$** +621.9 (>99% ee, *c* 0.56, $CHCl_3$); **^{1}H NMR** (400 MHz, $CDCl_3$) δ 7.40–7.27 (m, 5H), 7.19 (t, *J* = 7.2 Hz, 1H), 7.09 (t, *J* = 7.2 Hz, 1H), 7.03 (d, *J* = 6.8 Hz, 1H), 6.80–6.71 (m, 2H), 6.40–6.29 (m, 2H), 6.02 (s, 1H), 5.99 (d, *J* = 7.6 Hz, 1H), 5.49 (d, *J* = 8.8 Hz, 1H), 5.37 (dd, *J* = 8.8, 10.8 Hz, 1H), 4.03–3.92 (m, 1H), 2.56 (s, 3H), 2.24 (s, 3H); **^{13}C NMR** (100 MHz, $CDCl_3$) δ 170.3, 153.6, 144.7, 138.1, 135.4, 140.0, 128.9, 128.7, 128.3, 127.9, 127.0, 126.7, 125.1, 124.0, 119.9, 113.0, 112.2, 110.8, 62.7, 60.7, 58.1, 52.1, 14.6, 13.9; **HRMS** (ESI+) calcd for $C_{22}H_{20}N_6NaO$ $[M+H]^+$ 446.1975, found 446.1977; The ee was determined by **HPLC** analysis (Daicel Chiralcel AD-3 column, hexane–*i*-PrOH = 98.4: 1.6, flow rate = 1 mL/min) t_R= 33.9 (major enantiomer), 37.0 (minor) min.

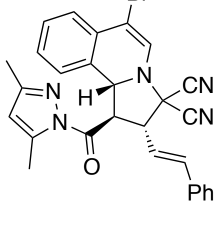

(1*R*,2*R*,10b*R*)-(+)-6-bromo-1-(3,5-dimethyl-1*H*-pyrazole-1-carbonyl)-2-((*E*)-styryl)-1,10b-dihydropyrrolo[2,1-*a*]isoquinoline-3,3(2*H*)-dicarbonitrile (*exo*-5bd) (Use of L5, Table 5): 78% yield; Yellowish form; **[α]^{25}$_{D}$** +607.3 (>99% ee, *c* 0.92, $CHCl_3$); **^{1}H NMR** (400 MHz, $CDCl_3$) δ 7.50 (d, *J* = 7.6 Hz, 1H), 7.42–7.27 (m, 6H), 7.21 (t, *J* = 7.6 Hz, 1H), 6.87–6.70 (m, 3H), 6.31 (dd, *J* = 9.2, 15.6 Hz, 1H), 6.03 (s, 1H), 5.52 (d, *J* = 8.4 Hz, 1H), 5.40 (dd, *J* = 8.4, 10.8 Hz, 1H), 4.04–3.93 (m, 1H), 2.56 (s, 3H), 2.26 (s, 3H); **^{13}C NMR** (100 MHz, $CDCl_3$) δ 170.0, 153.8, 144.8, 138.5, 135.2, 129.5, 128.7, 128.6, 127.2, 127.0, 123.9, 119.3, 113.1, 112.3, 111.9, 106.6, 62.5, 60.3, 58.2, 51.9, 14.5, 14.0; **HRMS** (ESI+) calcd for $C_{28}H_{22}BrN_5NaO$ $[M+Na]^+$ 546.0900, found 546.0892;

The ee was determined by **HPLC** analysis (Daicel Chiralcel IA-3 column, hexane–*i*-PrOH = 98.4: 1.6, flow rate = 1 mL/min) t_R= 12.9 (major enantiomer), 16.0 (minor) min.

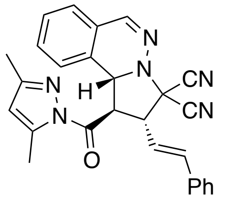

(1*R*,2*R*,10b*R*)-(+)-1-(3,5-dimethyl-1*H*-pyrazole-1-carbonyl)-2-((*E*)-styryl)-1,10b-dihydropyrrolo[2,1-*a*]phthalazine-3,3(2*H*)-dicarbonitrile (*exo*-5dd) (Use of L5, Table 5): 91% yield; White form; **[α]^{25}$_D$** +353.6 (>99% ee, *c* 0.88, $CHCl_3$); **^{1}H NMR** (400 MHz, $CDCl_3$) δ 7.76 (s, 1H), 7.44–7.25 (m, 8H), 6.98–6.89 (m, 1H), 6.67 (d, *J* = 15.6 Hz, 1H), 6.33 (dd, *J* = 10.0, 15.6 Hz, 1H), 6.04 (s, 1H), 5.10–4.93 (m, 2H), 3.98 (dd, *J* = 7.6, 9.6 Hz, 1H), 2.59 (s, 3H), 2.17 (s, 3H); **^{13}C NMR** (100 MHz, $CDCl_3$) δ 170.1, 153.6, 145.3, 144.7, 137.2, 135.5, 132.2, 128.9, 128.7, 127.0, 126.3, 125.0, 123.9, 122.7, 11 3.4, 112.9, 111.8, 61.2, 60.0, 56.3, 49.8, 14.5, 13.8; **HRMS** (ESI+) calcd for $C_{27}H_{23}N_6O$ $[M+H]^+$ 447.1928, found 447.1917; The ee was determined by **HPLC** analysis (Daicel Chiralcel AD-3 column, hexane–*i*-PrOH = 98: 2, flow rate = 1 mL/min) t_R= 35.0 (minor enantiomer), 45.5 (major) min.

7. Synthetic Transformation of 3am (Scheme 3)

<u>Chlorination:</u>

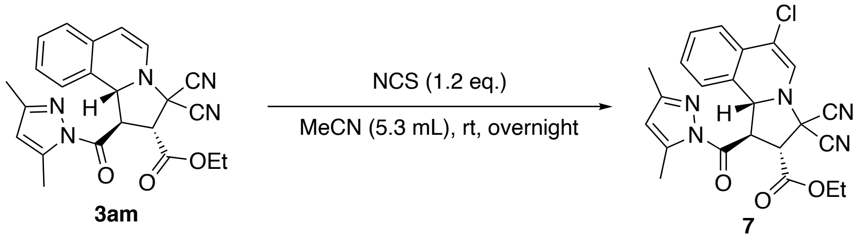

A mixture of **3am** (0.2 mmol) and *N*-Chlorosuccinimide (NCS, 1.2 equiv) were dissolved in anhydrous acetonitrile (5.3 mL). After stirring overnight at ambient temperature, the reaction was quenched by the addition of saturated *aq*. $Na_2S_2O_3$ (5 mL). The resultant mixture was extracted with ethyl acetate (3 x 10 mL). The combined organic layer was dried over Na_2SO_4, filtered and concentrated. The crude product was purified by column chromatography on silica gel using (hexane-EtOAc = 10:1 → 5:1) to give **7**.

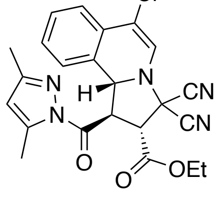

Ethyl (1*R*,2*R*,10b*R*)-(+)-6-Chloro-3,3-dicyano-1-(3,5-dimethyl-1*H*-pyrazole-1-carbonyl)-1,2,3,10b-tetrahydropyrrolo[2,1-a]isoquinoline-2-carboxylate (7): 75% yield; Yellow foam; **[α]^{25}$_D$** +424.1 (>99% ee, *c* 0.66, $CHCl_3$); **^{1}H NMR** (400 MHz, $CDCl_3$) δ 7.54 (d, *J* = 8.0 Hz,

1H), 7.39–7.27 (m, 3H), 6.62 (s, 1H), 6.10 (s, 1H), 5.40 (dd, J = 7.2, 9.6 Hz, 1H), 5.24 (d, J = 7.2 Hz, 1H), 4.40 (d, J = 9.6 Hz, 1H), 4.30–4.20 (m, 2H), 2.60 (s, 3H), 2.26 (s, 3H), 1.20 (t, J = 7.2 Hz, 3H); **^{13}C NMR** (100 MHz, $CDCl_3$) δ 169.9, 165.9, 153.7, 145.0, 129.7, 129.3, 128.7, 124.8, 124.2, 123.1, 120.4, 112.7, 111.9, 111.6, 63.2, 63.0, 57.1, 56.1, 48.2, 14.5, 13.9, 13.7; **HRMS** (FAB+) calcd for $C_{23}H_{20}ClN_5NaO_3$ $[M+Na]^+$ 472.1147; found 472.1167; The ee was determined by **HPLC** analysis (Daicel Chiralcel IA-3 column, hexane–*i*-PrOH = 95: 5, flow rate = 1.0 mL/min) t_R= 8.4 (minor enantiomer), 10.0 (major) min

Elimination:

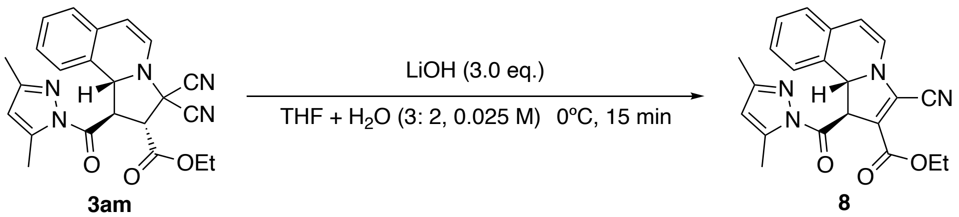

To a solution of **3am** (0.2 mmol) in THF/H_2O (3:2, 8 mL) was added LiOH (3.0 equiv) at ambient temperature. After stirring 15 minutes at ambient temperature, the reaction was quenched by the addition of 1 *M* HCl. The resultant mixture was extracted with ethyl acetate. The combined organic layer was dried over Na_2SO_4, filtered and concentrated. The crude product was purified by column chromatography on silica gel using (hexane-EtOAc = 10:1 → 5:1) to give **8**.

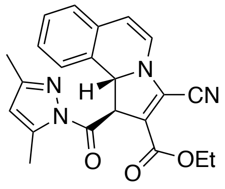

Ethyl (1*R*,10b*R*)-(+)-3-cyano-1-(3,5-dimethyl-1*H*-pyrazole-1-carbonyl)-1,10b-dihydropyrrolo[2,1-*a*]isoquinoline-2-carboxylate (8): 91% yield; Yellow foam; **$[\alpha]^{26}_{D}$** +327.0 (>99% ee, *c* 0.74, $CHCl_3$); **^{1}H NMR** (400 MHz, $CDCl_3$) δ 7.38 (d, J = 7.2 Hz, 1H), 7.28–7.21 (m, 1H), 7.20–7.14 (m, 1H), 7.09 (dd, J = 1,2, 7.2 Hz, 1H), 6.82 (d, J = 7.6 Hz, 1H), 6.19 (d, J = 10.6 Hz, 1H), 6.11 (d, J = 1.2 Hz, 1H), 6.00 (d, J = 8.0 Hz, 1H), 5.49 (d, J = 13.6 Hz, 1H), 4.14 (dd, J = 7.2, 9.2 Hz, 1H), 2.58 (s, 3H), 2.32 (s, 3H), 1.12 (t, J = 7.2 Hz, 3H); **^{13}C NMR** (100 MHz, $CDCl_3$) δ 173.1, 162.3, 152.9, 144.6, 131.5, 129.6, 128.5, 127.5, 125.0, 124.7, 124.4, 124.0, 116.0, 112.5, 110.1, 109.1, 66.2, 61.0, 51.3, 14.6, 14.1, 13.8; **HRMS** (ESI+) calcd for $C_{22}H_{20}N_4NaO_3$ $[M+Na]^+$ 411.1428; found 411.1448; The ee was determined by **HPLC** analysis (Daicel Chiralcel OD-3 column, hexane–*i*-PrOH = 98: 2, flow rate = 1.0 mL/min) t_R= 19.0 (major enantiomer), 25.8 (minor) min.

8. General Procedure for the Remote Asymmetric Dearomative [3+2] Cycloaddition Reactions of 1a with 9a.

A mixture of monopeptide ligand **L5** (11 mol%), copper(II) triflate (10 mol%) and activated molecular sieves 4A (150–200 mg) were dissolved in anhydrous acetonitrile (1.0 mL). After stirring

for 15 min at ambient temperature, the solution was concentrated under reduced pressure. The resultant residue was diluted with CH_2Cl_2 (2.0 mL) and cooled to –10ºC. Isoquinolinium ylides **1a** (0.2 mmol, 1.0 equiv) and **9a** (0.2 mmol) were then added and the reaction mixture was stirred at the same temperature for 48 h. The reaction was quenched with a few drops of triethylamine and then passed through a short pad silica gel to remove ligand and copper. After evaporation of the filtrate under reduced pressure, the crude product was then purified by column chromatography on silica gel (hexane–EtOAc = 10:1 to 5:1) to give products **10aa**, **11aa**, and **12aa**.

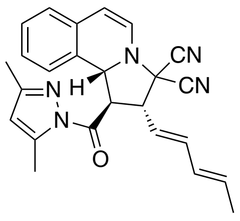

(1*R*,2*R*,10b*R*)-(+)-1-(3,5-Dimethyl-1*H*-pyrazole-1-carbonyl)-2-((1*E*,3*E*)-penta-1,3-dien-1-yl)-1,10b-dihydropyrrolo[2,1-*a*]isoquinoline-3,3(2*H*)-dicarbonitrile (10aa): 7% yield; Yellowish form; **[α]^{24}$_D$** +483.7 (>99% ee, *c* 0.53, $CHCl_3$); **^{1}H: NMR** (400 MHz, $CDCl_3$) δ 7.22–7.12 (m, 1H), 7.11–6.98 (m, 2H), 6.73 (d, *J* = 7.6 Hz, 1H), 6.38 (dd, *J* = 10.4, 15.2 Hz, 1H), 6.34 (d, *J* = 7.2 Hz, 1H), 6.08 (d, *J* = 0.8 Hz, 1H), 6.07–5.92 (m, 3H), 5.83–5.70 (m, 1H), 5.64 (dd, *J* = 9.6, 15.2 Hz, 1H), 5.40 (d, *J* = 8.8 Hz, 1H), 5.27 (dd, *J* = 8.8, 11.6 Hz, 1H), 3.86 (dd, *J* = 9.6, 10.8 Hz, 1H), 2.57 (s, 3H), 2.27 (s, 3H), 1.76 (dd, *J* = 1.0, 7.0 Hz, 3H); **^{13}C NMR** (100 MHz, $CDCl_3$) δ 170.5, 153.5, 144.6, 138.8, 133.6, 131.0, 129.9, 129.0, 128.3, 127.8, 126.7, 125.0, 124.1, 119.6, 113.1, 113.0, 112.2, 110.6, 63.0, 60.8, 58.0, 51.8, 18.3, 14.6, 14.0; **HRMS** (ESI+) calcd for $C_{25}H_{24}N_5O$ $[M+Na]^+$ 432.1795, found 432.1793; The ee was determined by **HPLC** analysis (Daicel Chiralcel IA-3, hexane–*i*-PrOH = 98.4: 16, flow rate = 1.0 mL/min) t_R= 12.3 (major enantiomer), 13.9 (minor) min.

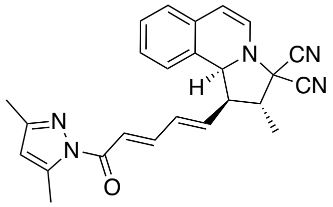

(1*R*,2*R*,10b*S*)-(+)-1-((1*E*,3*E*)-5-(3,5-Dimethyl-1*H*-pyrazol-1-yl)-5-oxopenta-1,3-dien-1-yl)-2-methyl-1,10b-dihydropyrrolo[2,1-*a*]isoquinoline-3,3(2*H*)-dicarbonitrile (11aa): 75% yield; Yellowish form; **[α]^{26}$_D$** +33.5 (86% ee, *c* 0.49, $CHCl_3$); **^{1}H NMR** (400 MHz, $CDCl_3$) δ 7.41–7.29 (m, 2H), 7.20–7.12 (m, 1H), 7.11–6.99 (m, 2H), 6.84 (d, *J* = 8.0 Hz, 1H), 6.52–6.39 (m, 2H), 6.22 (dd, *J* = 10.0, 15.2 Hz, 1H), 5.99 (s, 1H), 5.81 (d, *J* = 8.0 Hz, 1H), 5.00 (d, *J* = 6.8 Hz, 1H), 3.12–3.02 (m, 1H), 2.82–2.70 (m, 1H), 2.56 (s, 3H), 2.27 (s, 3H), 1.53 (d, *J* = 7.2 Hz, 3H); **^{13}C NMR** (100 MHz, $CDCl_3$) δ 165.2, 152.1, 144.5, 144.4, 141.5, 131.9, 131.3, 128.3, 128.0, 127.1, 126.3, 125.4, 121.9, 113.4, 111.5, 111.4, 108.8, 61.5, 59.1, 51.6, 51.3, 16.0, 14.7, 13.9; **HRMS** (ESI+) calcd for $C_{25}H_{24}N_5O$ $[M+H]^+$ 410.1975, found 410.1987; The ee was determined by **HPLC** analysis (Daicel Chiralcel IA-3, hexane–*i*-PrOH = 95: 5, flow rate = 1.0 mL/min) t_R= 24.6 (minor

enantiomer), 44.2 (major) min.

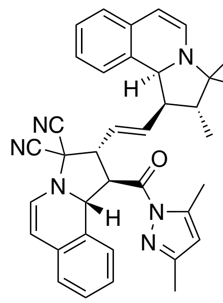

(1*R*,2*R*,10b*S*)-(+)-1-((*E*)-2-((1*R*,2*R*,10b*R*)-3,3-Dicyano-1-(3,5-dimethyl-1*H*-pyrazole-1-carbonyl)-1,2,3,10b-tetrahydropyrrolo[2,1-*a*]isoquinolin-2-yl)vinyl)-2-methyl-1,10b-dihydropyrrolo[2,1-*a*]isoquinoline-3,3(2*H*)-dicarbonitrile (12aa): 11% yield; Yellowish form; **$[\alpha]^{24}_{D}$** +240.0 (>99% ee, *c* 0.98, $CHCl_3$); **^{1}H NMR** (400 MHz, $CDCl_3$) δ 7.23–6.79 (m, 6H), 6.84 (d, J = 7.6 Hz, 1H), 6.65 (d, J = 7.2 Hz, 1H), 6.39–6.29 (m, 2H), 6.12–6.01 (m, 2H), 5.96 (d, J = 7.6 Hz, 1H), 5.86 (dd, J = 8.8, 15.6 Hz, 1H), 5.78 (d, J = 7.2 Hz, 1H), 5.38 (d, J = 8.8 Hz, 1H), 5.26 (d, J = 8.6, 11.4 Hz, 1H), 4.88 (d, J = 6.8 Hz, 1H), 3.78 (dd, J = 8.6, 11.0 Hz, 1H), 3.02–2.90 (m, 1H), 2.51 (s, 3H), 2.26 (s, 3H), 2.23–2.15 (m, 1H), 1.34 (d, J = 7.2 Hz, 3H); **^{13}C NMR** (100 MHz, $CDCl_3$) δ 169.9, 153.6, 145.1, 138.5, 131.7, 130.8, 128.8, 128.4, 128.0, 127.7, 127.5, 126.4, 126.3, 125.3, 125.1, 123.9, 123.8, 113.4, 112.9, 112.5, 112.2, 111.3, 110.8, 109.1, 62.7, 60.9, 60.0, 58.8, 57.2, 51.9, 51.2, 50.5, 15.8, 14.7, 14.0; **HRMS** (ESI+) calcd for $C_{37}H_{30}N_8NaO$ $[M+Na]^+$ 625.2435, found 625.2441; The ee was determined by **HPLC** analysis (Daicel Chiralcel IA-3, hexane–*i*-PrOH = 98.4: 16, flow rate = 1.0 mL/min) t_R= 33.4 (major enantiomer), 35.4 (minor) min.

9. NOE Analysis of 6aa, 6ab and 6ac

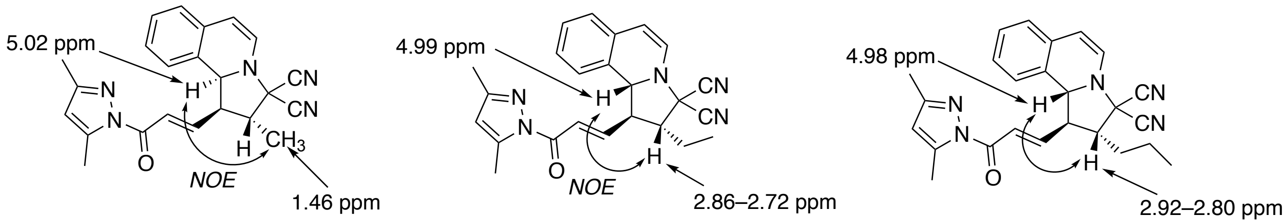

(*endo*)-**6aa** (*exo*)-**6ab** (*exo*)-**6ac**

10. X-ray Diffraction Analysis of (1*R*,2*S*,10b*R*)-3de (Fig. 1)

(1*R*,2*S*,10*bR*)-**3de** (98% ee) was recrystallized in hexane–CH_2Cl_2 at ambient temperature to obtain a single crystal (>99% ee).

Crystal data of (1*R*,2*S*,10*bR*)-**3de** (Figure S1): Formula $C_{25}H_{19}BrN_6O$, colorless, monoclinic, space group *P* 1 21 1, a = 8.2893(3) Å, b = 14.1630(4) Å, c =10.0325(3) Å, α = 90°, β = 103.686(3)°, γ = 90 °, V = 1144.39(6) Å^3, Z = 2, ρcalc = 1.449 g/cm^3, λ(MoKα) = 0.71073 Å, T = 123 K. 9917 reflections collected, and 5540 parameters were used for the solution of the structure. R_1 = 0.0246 and wR_2 = 0.0655. GOF = 10.75. Flack x parameter = 0.011(4). Crystallographic data (excluding

structure factors) for the structure reported in this paper have been deposited with the Cambridge Crystallographic Data Centre as supplementary publication no. CCDC-2325959. Copies of the data can be obtained free of charge on application to CCDC, 12 Union Road, Cambridge CB2 1EZ, UK [Fax: int. code + 44(1223)336-033; E-mail: deposit@ccdc.cam.ac.uk; Web page: http://www.ccdc.cam.ac.uk/pages/Home.aspx].

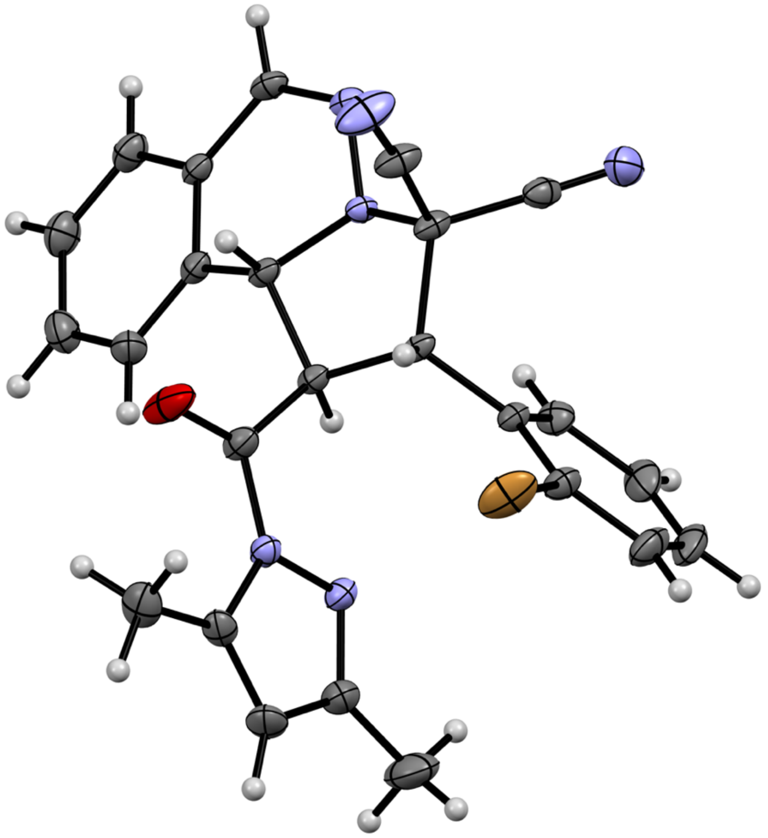

Fig. S1 X-ray diffraction analysis of (1*R*,2*S*,10*bR*)-3de (ORTEP Drawing).

11. HOMO of 1a and LUMO of 4a

The LUMO/HOMO coefficients of compound **4a, 4a•**HCl, **4a•**Cu(OTf)$_2$**•4a** and **1a** were calculated using DFT calculations with RB3LYP with 6-31G(d) basis sets (6-311G** for **4a•**Cu(OTf)$_2$**•4a**; Spartan'18 for Macintosh). The molecular orbital coefficients are shown in below and the results are summarized in Table S1.

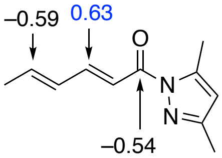

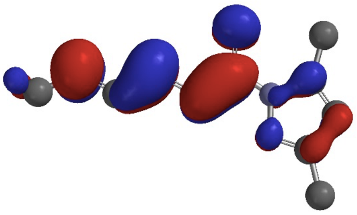

4a
LUMO coefficient

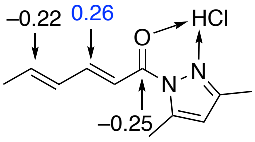

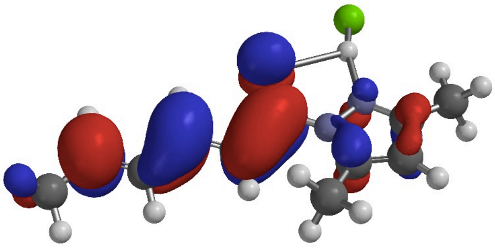

4a•HCl
LUMO coefficient

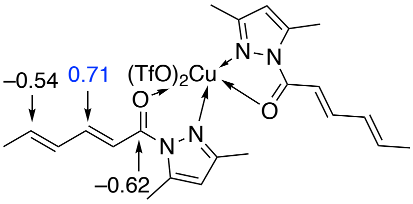

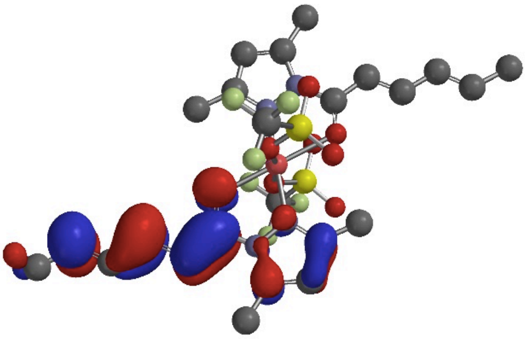

4a•Cu(OTf)$_2$•**4a**
LUMO coefficient

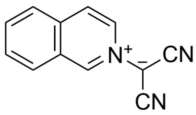

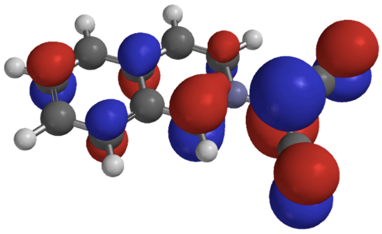

1a
HOMO coefficient

Table S1 Summary of calculation for molecular orbital coefficients of 4a, 4a•HCl, 4a•Cu(OTf)$_2$ and 1a.

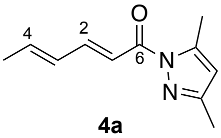

4a

```
SPARTAN '18 Quantum Mechanics Program:  (x86/Darwin)        build  1.3.0                    Parallel Job: 8 threads

Job type: Geometry optimization.
Method: RB3LYP
Basis set: 6-31G(D)
Number of basis functions: 238
Number of electrons: 102

SCF model:
 A restricted hybrid HF-DFT SCF calculation will be
 performed using Pulay DIIS + Geometric Direct Minimization
```

Optimization:

Step	Energy	Max Grad.	Max Dist.
1	-612.303619	0.018376	0.064515
2	-612.305870	0.004914	0.015896
3	-612.306083	0.000870	0.002602
4	-612.306092	0.000530	0.003335

Reason for exit: Successful completion

Quantum Calculation CPU Time : 6:54.91

Quantum Calculation Wall Time: 59.22

SPARTAN '18 Properties Program: (x86/Darwin) build 1.3.0

Use of molecular symmetry enabled

Cartesian Coordinates (Angstroms)

Atom		X	Y	Z
1 C	C6	-0.9648205	0.0000000	-0.3179337
2 N	N1	-0.3666913	0.0000000	-1.6055558
3 N	N2	1.0012275	0.0000000	-1.7415912
4 C	C7	1.2188309	0.0000000	-3.0456937
5 C	C8	-1.0110466	0.0000000	-2.8338870
6 C	C9	-0.0062819	0.0000000	-3.7713097
7 C	C11	2.6168086	0.0000000	-3.5934514
8 C	C10	-2.4935884	0.0000000	-3.0273499
9 O	O1	-2.1833003	0.0000000	-0.2158308
10 C	C1	-0.0323300	0.0000000	0.8205637
11 C	C2	-0.5211040	0.0000000	2.0795414
12 C	C3	0.2960092	0.0000000	3.2726726
13 C	C4	-0.2145824	0.0000000	4.5187408
14 C	C5	0.5906537	0.0000000	5.7801393
15 H	H15	-0.1327163	0.0000000	-4.8455147
16 H	H16	2.6059652	0.0000000	-4.6871693
17 H	H17	3.1716589	0.8827250	-3.2567735
18 H	H18	3.1716589	-0.8827250	-3.2567735
19 H	H19	-2.9615352	-0.8758258	-2.5683225
20 H	H20	-2.7132223	0.0000000	-4.0994595
21 H	H21	-2.9615352	0.8758258	-2.5683225
22 H	H22	1.0307441	0.0000000	0.6154028
23 H	H23	-1.6030333	0.0000000	2.2075054
24 H	H24	1.3779790	0.0000000	3.1369784
25 H	H25	-1.2995823	0.0000000	4.6342113
26 H	H26	0.3588784	-0.8793435	6.3970875
27 H	H27	0.3588784	0.8793435	6.3970875
28 H	H28	1.6660772	0.0000000	5.5750077

MO:		51	52	53	54	55
Eigenvalues:		-0.23062	-0.06568	-0.00114	0.05490	0.07647
(ev)		-6.27552	-1.78726	-0.03103	1.49398	2.08091
		A″	A″	A″	A″	A″
1 C6	S1	0.00000	0.00000	0.00000	0.00000	0.00000
2 C6	S	0.00000	0.00000	0.00000	0.00000	0.00000
3 C6	PX	0.00000	0.00000	0.00000	0.00000	0.00000
4 C6	PY	0.02252	-0.28289	-0.17641	0.25157	-0.06969
5 C6	PZ	0.00000	0.00000	0.00000	0.00000	0.00000
6 C6	S'	0.00000	0.00000	0.00000	0.00000	0.00000
7 C6	PX'	0.00000	0.00000	0.00000	0.00000	0.00000
8 C6	PY'	0.00853	-0.22194	-0.14964	0.21676	-0.07250
9 C6	PZ'	0.00000	0.00000	0.00000	0.00000	0.00000
10 C6	DXX	0.00000	0.00000	0.00000	0.00000	0.00000
11 C6	DYY	0.00000	0.00000	0.00000	0.00000	0.00000
12 C6	DZZ	0.00000	0.00000	0.00000	0.00000	0.00000
13 C6	DXY	0.01526	-0.02558	-0.00723	0.00800	0.00106
14 C6	DXZ	0.00000	0.00000	0.00000	0.00000	0.00000
15 C6	DYZ	0.01319	-0.00677	0.02038	0.00835	-0.00461
16 N1	S1	0.00000	0.00000	0.00000	0.00000	0.00000
17 N1	S	0.00000	0.00000	0.00000	0.00000	0.00000
18 N1	PX	0.00000	0.00000	0.00000	0.00000	0.00000
19 N1	PY	-0.08835	0.05819	-0.21173	-0.23687	0.10183
20 N1	PZ	0.00000	0.00000	0.00000	0.00000	0.00000
21 N1	S'	0.00000	0.00000	0.00000	0.00000	0.00000
22 N1	PX'	0.00000	0.00000	0.00000	0.00000	0.00000
23 N1	PY'	-0.06962	0.03340	-0.24215	-0.24160	0.12716
24 N1	PZ'	0.00000	0.00000	0.00000	0.00000	0.00000
25 N1	DXX	0.00000	0.00000	0.00000	0.00000	0.00000
26 N1	DYY	0.00000	0.00000	0.00000	0.00000	0.00000
27 N1	DZZ	0.00000	0.00000	0.00000	0.00000	0.00000
28 N1	DXY	-0.00430	0.00503	0.01194	0.00523	0.02180
29 N1	DXZ	0.00000	0.00000	0.00000	0.00000	0.00000
30 N1	DYZ	-0.00169	-0.01864	-0.02006	0.01067	0.01184
31 N2	S1	0.00000	0.00000	0.00000	0.00000	0.00000
32 N2	S	0.00000	0.00000	0.00000	0.00000	0.00000
33 N2	PX	0.00000	0.00000	0.00000	0.00000	0.00000
34 N2	PY	-0.10110	0.10543	0.28351	0.19580	0.15155
35 N2	PZ	0.00000	0.00000	0.00000	0.00000	0.00000
36 N2	S'	0.00000	0.00000	0.00000	0.00000	0.00000
37 N2	PX'	0.00000	0.00000	0.00000	0.00000	0.00000
38 N2	PY'	-0.06677	0.08273	0.32316	0.25334	0.20607
39 N2	PZ'	0.00000	0.00000	0.00000	0.00000	0.00000
40 N2	DXX	0.00000	0.00000	0.00000	0.00000	0.00000
41 N2	DYY	0.00000	0.00000	0.00000	0.00000	0.00000
42 N2	DZZ	0.00000	0.00000	0.00000	0.00000	0.00000
43 N2	DXY	0.00554	-0.00621	-0.00155	0.00374	-0.01610
44 N2	DXZ	0.00000	0.00000	0.00000	0.00000	0.00000
45 N2	DYZ	0.00217	-0.00168	-0.00157	0.00204	0.01424
46 C7	S1	0.00000	0.00000	0.00000	0.00000	0.00000
47 C7	S	0.00000	0.00000	0.00000	0.00000	0.00000
48 C7	PX	0.00000	0.00000	0.00000	0.00000	0.00000
49 C7	PY	0.02557	-0.09771	-0.22120	-0.17073	-0.35725
50 C7	PZ	0.00000	0.00000	0.00000	0.00000	0.00000
51 C7	S'	0.00000	0.00000	0.00000	0.00000	0.00000
52 C7	PX'	0.00000	0.00000	0.00000	0.00000	0.00000
53 C7	PY'	0.00707	-0.06317	-0.21901	-0.21956	-0.46165
54 C7	PZ'	0.00000	0.00000	0.00000	0.00000	0.00000
55 C7	DXX	0.00000	0.00000	0.00000	0.00000	0.00000
56 C7	DYY	0.00000	0.00000	0.00000	0.00000	0.00000
57 C7	DZZ	0.00000	0.00000	0.00000	0.00000	0.00000
58 C7	DXY	-0.00397	0.00183	0.00260	-0.00204	-0.01943
59 C7	DXZ	0.00000	0.00000	0.00000	0.00000	0.00000
60 C7	DYZ	-0.00750	0.00923	0.02130	0.01129	-0.00304
61 C8	S1	0.00000	0.00000	0.00000	0.00000	0.00000
62 C8	S	0.00000	0.00000	0.00000	0.00000	0.00000
63 C8	PX	0.00000	0.00000	0.00000	0.00000	0.00000
64 C8	PY	0.08842	0.12093	0.30544	0.09063	-0.30861
65 C8	PZ	0.00000	0.00000	0.00000	0.00000	0.00000
66 C8	S'	0.00000	0.00000	0.00000	0.00000	0.00000
67 C8	PX'	0.00000	0.00000	0.00000	0.00000	0.00000
68 C8	PY'	0.04903	0.14884	0.34211	0.07541	-0.42683
69 C8	PZ'	0.00000	0.00000	0.00000	0.00000	0.00000
70 C8	DXX	0.00000	0.00000	0.00000	0.00000	0.00000
71 C8	DYY	0.00000	0.00000	0.00000	0.00000	0.00000
72 C8	DZZ	0.00000	0.00000	0.00000	0.00000	0.00000
73 C8	DXY	0.00369	0.00034	-0.00591	-0.00527	0.01810
74 C8	DXZ	0.00000	0.00000	0.00000	0.00000	0.00000
75 C8	DYZ	-0.00704	0.00625	-0.00732	-0.01695	-0.01496
76 C9	S1	0.00000	0.00000	0.00000	0.00000	0.00000
77 C9	S	0.00000	0.00000	0.00000	0.00000	0.00000
78 C9	PX	0.00000	0.00000	0.00000	0.00000	0.00000
79 C9	PY	0.13600	-0.09225	-0.09852	0.05998	0.39448
80 C9	PZ	0.00000	0.00000	0.00000	0.00000	0.00000
81 C9	S'	0.00000	0.00000	0.00000	0.00000	0.00000
82 C9	PX'	0.00000	0.00000	0.00000	0.00000	0.00000
83 C9	PY'	0.11289	-0.11839	-0.14028	0.09043	0.61752
84 C9	PZ'	0.00000	0.00000	0.00000	0.00000	0.00000
85 C9	DXX	0.00000	0.00000	0.00000	0.00000	0.00000
86 C9	DYY	0.00000	0.00000	0.00000	0.00000	0.00000
87 C9	DZZ	0.00000	0.00000	0.00000	0.00000	0.00000
88 C9	DXY	-0.00178	-0.00793	-0.02313	-0.01444	-0.00465
89 C9	DXZ	0.00000	0.00000	0.00000	0.00000	0.00000
90 C9	DYZ	0.00321	0.00238	0.00554	-0.00180	-0.01857

91	C11	S1	0.00000	0.00000	0.00000	0.00000	0.00000
92	C11	S	0.00000	0.00000	0.00000	0.00000	0.00000
93	C11	PX	0.00000	0.00000	0.00000	0.00000	0.00000
94	C11	PY	-0.00547	0.00687	-0.01029	-0.03069	-0.05960
95	C11	PZ	0.00000	0.00000	0.00000	0.00000	0.00000
96	C11	S'	0.00000	0.00000	0.00000	0.00000	-0.00000
97	C11	PX'	0.00000	0.00000	0.00000	0.00000	0.00000
98	C11	PY'	0.00958	-0.04043	-0.12244	-0.11847	-0.18313
99	C11	PZ'	0.00000	0.00000	0.00000	0.00000	-0.00000
100	C11	DXX	0.00000	0.00000	0.00000	0.00000	0.00000
101	C11	DYY	0.00000	0.00000	0.00000	0.00000	0.00000
102	C11	DZZ	0.00000	0.00000	0.00000	0.00000	0.00000
103	C11	DXY	-0.00154	0.00612	0.01483	0.01251	0.02624
104	C11	DXZ	0.00000	0.00000	0.00000	0.00000	0.00000
105	C11	DYZ	-0.00048	0.00006	0.00090	0.00031	-0.00389
106	C10	S1	0.00000	0.00000	0.00000	0.00000	0.00000
107	C10	S	0.00000	0.00000	0.00000	0.00000	0.00000
108	C10	PX	0.00000	0.00000	0.00000	0.00000	0.00000
109	C10	PY	-0.01379	-0.02115	-0.00215	0.02547	-0.04060
110	C10	PZ	0.00000	0.00000	0.00000	0.00000	0.00000
111	C10	S'	0.00000	0.00000	0.00000	0.00000	0.00000
112	C10	PX'	0.00000	0.00000	0.00000	0.00000	0.00000
113	C10	PY'	0.01395	-0.04653	0.05590	0.14053	-0.10796
114	C10	PZ'	0.00000	0.00000	0.00000	0.00000	0.00000
115	C10	DXX	0.00000	0.00000	0.00000	0.00000	0.00000
116	C10	DYY	0.00000	0.00000	0.00000	0.00000	0.00000
117	C10	DZZ	0.00000	0.00000	0.00000	0.00000	0.00000
118	C10	DXY	0.00413	0.00717	0.01982	0.00743	-0.02359
119	C10	DXZ	0.00000	0.00000	0.00000	0.00000	0.00000
120	C10	DYZ	-0.00149	-0.00061	-0.00338	-0.00296	0.00109
121	O1	S1	0.00000	0.00000	0.00000	0.00000	0.00000
122	O1	S	0.00000	0.00000	0.00000	0.00000	0.00000
123	O1	PX	0.00000	0.00000	0.00000	0.00000	0.00000
124	O1	PY	-0.15851	0.25849	0.13106	-0.12624	0.03656
125	O1	PZ	0.00000	0.00000	0.00000	0.00000	0.00000
126	O1	S'	0.00000	0.00000	0.00000	0.00000	0.00000
127	O1	PX'	0.00000	0.00000	0.00000	0.00000	0.00000
128	O1	PY'	-0.11943	0.24872	0.12888	-0.14880	0.04107
129	O1	PZ'	0.00000	0.00000	0.00000	0.00000	0.00000
130	O1	DXX	0.00000	0.00000	0.00000	0.00000	0.00000
131	O1	DYY	0.00000	0.00000	0.00000	0.00000	0.00000
132	O1	DZZ	0.00000	0.00000	0.00000	0.00000	0.00000
133	O1	DXY	-0.00592	0.00124	-0.00083	0.00557	-0.00245
134	O1	DXZ	0.00000	0.00000	0.00000	0.00000	0.00000
135	O1	DYZ	0.00251	-0.00095	0.00257	0.00053	-0.00009
136	C1	S1	0.00000	0.00000	0.00000	0.00000	0.00000
137	C1	S	0.00000	0.00000	0.00000	0.00000	0.00000
138	C1	PX	0.00000	0.00000	0.00000	0.00000	0.00000
139	C1	PY	0.31795	-0.20912	0.20219	-0.01415	-0.03394
140	C1	PZ	0.00000	0.00000	0.00000	0.00000	0.00000
141	C1	S'	0.00000	0.00000	0.00000	0.00000	0.00000
142	C1	PX'	0.00000	0.00000	0.00000	0.00000	0.00000
143	C1	PY'	0.24712	-0.24098	0.24241	0.09986	-0.02745
144	C1	PZ'	0.00000	0.00000	0.00000	0.00000	0.00000
145	C1	DXX	0.00000	0.00000	0.00000	0.00000	0.00000
146	C1	DYY	0.00000	0.00000	0.00000	0.00000	0.00000
147	C1	DZZ	0.00000	0.00000	0.00000	0.00000	0.00000
148	C1	DXY	-0.00591	0.00314	0.00417	0.00058	0.00109
149	C1	DXZ	0.00000	0.00000	0.00000	0.00000	0.00000
150	C1	DYZ	0.00606	0.02476	0.00184	-0.02142	0.00466
151	C2	S1	0.00000	0.00000	0.00000	0.00000	0.00000
152	C2	S	0.00000	0.00000	0.00000	0.00000	0.00000
153	C2	PX	0.00000	0.00000	0.00000	0.00000	0.00000
154	C2	PY	0.19178	0.29661	-0.06476	-0.23132	0.07041
155	C2	PZ	0.00000	0.00000	0.00000	0.00000	0.00000
156	C2	S'	0.00000	0.00000	0.00000	0.00000	0.00000
157	C2	PX'	0.00000	0.00000	0.00000	0.00000	0.00000
158	C2	PY'	0.13008	0.32098	-0.07081	-0.38464	0.09620
159	C2	PZ'	0.00000	0.00000	0.00000	0.00000	0.00000
160	C2	DXX	0.00000	0.00000	0.00000	0.00000	0.00000
161	C2	DYY	0.00000	0.00000	0.00000	0.00000	0.00000
162	C2	DZZ	0.00000	0.00000	0.00000	0.00000	0.00000
163	C2	DXY	0.00108	0.00113	-0.00162	0.01254	-0.00354
164	C2	DXZ	0.00000	0.00000	0.00000	0.00000	0.00000
165	C2	DYZ	-0.02085	0.01300	-0.01686	0.01420	-0.00070
166	C3	S1	0.00000	0.00000	0.00000	0.00000	0.00000
167	C3	S	0.00000	0.00000	0.00000	0.00000	0.00000
168	C3	PX	0.00000	0.00000	0.00000	0.00000	0.00000
169	C3	PY	-0.23736	0.10319	-0.18302	0.35464	-0.07519
170	C3	PZ	0.00000	0.00000	0.00000	0.00000	0.00000
171	C3	S'	0.00000	0.00000	0.00000	0.00000	0.00000
172	C3	PX'	0.00000	0.00000	0.00000	0.00000	0.00000
173	C3	PY'	-0.17080	0.11177	-0.23101	0.55483	-0.11792
174	C3	PZ'	0.00000	0.00000	0.00000	0.00000	0.00000
175	C3	DXX	0.00000	0.00000	0.00000	0.00000	0.00000
176	C3	DYY	0.00000	0.00000	0.00000	0.00000	0.00000
177	C3	DZZ	0.00000	0.00000	0.00000	0.00000	0.00000
178	C3	DXY	0.00230	-0.00299	-0.00166	0.01462	-0.00400
179	C3	DXZ	0.00000	0.00000	0.00000	0.00000	0.00000
180	C3	DYZ	-0.01816	-0.02361	0.01243	-0.00407	-0.00059
181	C4	S1	0.00000	0.00000	0.00000	0.00000	0.00000
182	C4	S	0.00000	0.00000	0.00000	0.00000	0.00000
183	C4	PX	0.00000	0.00000	0.00000	0.00000	0.00000
184	C4	PY	-0.29962	-0.28867	0.20994	-0.24960	0.04507
185	C4	PZ	0.00000	0.00000	0.00000	0.00000	0.00000
186	C4	S'	0.00000	0.00000	0.00000	-0.00000	0.00000
187	C4	PX'	0.00000	0.00000	0.00000	0.00000	0.00000
188	C4	PY'	-0.23249	-0.29676	0.26308	-0.42584	0.08509
189	C4	PZ'	0.00000	0.00000	0.00000	-0.00000	0.00000
190	C4	DXX	0.00000	0.00000	0.00000	0.00000	0.00000
191	C4	DYY	0.00000	0.00000	0.00000	0.00000	0.00000
192	C4	DZZ	0.00000	0.00000	0.00000	0.00000	0.00000
193	C4	DXY	-0.00301	0.00113	-0.00395	0.01115	-0.00260
194	C4	DXZ	0.00000	0.00000	0.00000	0.00000	0.00000
195	C4	DYZ	0.01248	-0.00438	0.00912	-0.01835	0.00407
196	C5	S1	0.00000	0.00000	0.00000	0.00000	0.00000
197	C5	S	0.00000	0.00000	0.00000	0.00000	0.00000
198	C5	PX	0.00000	0.00000	0.00000	0.00000	0.00000
199	C5	PY	0.07891	0.02401	-0.00182	-0.01936	0.00541
200	C5	PZ	0.00000	0.00000	0.00000	0.00000	0.00000
201	C5	S'	0.00000	0.00000	-0.00000	0.00000	-0.00000
202	C5	PX'	0.00000	0.00000	0.00000	-0.00000	0.00000
203	C5	PY'	0.03097	-0.01760	0.02014	0.00243	-0.00247
204	C5	PZ'	0.00000	0.00000	0.00000	0.00000	0.00000
205	C5	DXX	0.00000	0.00000	0.00000	0.00000	0.00000
206	C5	DYY	0.00000	0.00000	0.00000	0.00000	0.00000
207	C5	DZZ	0.00000	0.00000	0.00000	0.00000	0.00000
208	C5	DXY	0.00244	0.00556	-0.00522	0.00804	-0.00162
209	C5	DXZ	0.00000	0.00000	0.00000	0.00000	0.00000
210	C5	DYZ	0.01650	0.01677	-0.01305	0.01658	-0.00313
211	H15	S	0.00000	0.00000	0.00000	0.00000	0.00000
212	H15	S'	0.00000	0.00000	0.00000	0.00000	-0.00000
213	H16	S	0.00000	0.00000	0.00000	0.00000	0.00000
214	H16	S'	0.00000	0.00000	0.00000	-0.00000	-0.00000
215	H17	S	-0.00593	0.01979	0.04488	0.03606	0.07349
216	H17	S'	-0.01539	0.06253	0.19189	0.19168	0.33673
217	H18	S	0.00593	-0.01979	-0.04488	-0.03606	-0.07349
218	H18	S'	0.01539	-0.06253	-0.19189	-0.19168	-0.33673
219	H19	S	0.01633	0.02327	0.05831	0.02490	-0.06442
220	H19	S'	0.04249	0.00299	0.16718	0.17172	-0.24803
221	H20	S	0.00000	0.00000	0.00000	0.00000	0.00000
222	H20	S'	0.00000	0.00000	0.00000	0.00000	0.00000
223	H21	S	-0.01633	-0.02327	-0.05831	-0.02490	0.06442
224	H21	S'	-0.04249	-0.00299	-0.16718	-0.17172	0.24803
225	H22	S	0.00000	0.00000	0.00000	0.00000	0.00000
226	H22	S'	0.00000	0.00000	0.00000	0.00000	0.00000
227	H23	S	0.00000	0.00000	0.00000	0.00000	0.00000
228	H23	S'	0.00000	0.00000	0.00000	0.00000	0.00000
229	H24	S	0.00000	0.00000	0.00000	0.00000	0.00000
230	H24	S'	0.00000	0.00000	0.00000	0.00000	0.00000
231	H25	S	0.00000	0.00000	0.00000	0.00000	0.00000
232	H25	S'	0.00000	0.00000	0.00000	0.00000	0.00000
233	H26	S	-0.07055	-0.05628	0.04105	-0.04877	0.00894
234	H26	S'	-0.09815	-0.12054	0.10375	-0.13037	0.02435

235	H27	S	0.07055	0.05628	-0.04105	0.04877	-0.00894
236	H27	S'	0.09815	0.12054	-0.10375	0.13037	-0.02435

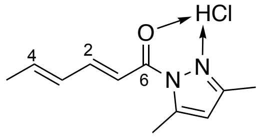

4a•HCl

SPARTAN '18 Quantum Mechanics Program: (x86/Darwin) build 1.3.0

Job type: Geometry optimization.
Method: RB3LYP
Basis set: 6-31G(D)
Number of basis functions: 259
Number of electrons: 120
Parallel Job: 8 threads

SCF model:
A restricted hybrid HF-DFT SCF calculation will be
performed using Pulay DIIS + Geometric Direct Minimization

Optimization:

Step	Energy	Max Grad.	Max Dist.
1	-1072.823833	0.130926	0.156773
2	-1072.894278	0.100359	0.164955
3	-1072.945216	0.075410	0.162483
4	-1072.982635	0.055150	0.159841
5	-1073.009282	0.039189	0.149404
6	-1073.028516	0.027172	0.133966
7	-1073.043005	0.018038	0.114815
8	-1073.054732	0.013077	0.118709
9	-1073.064912	0.012859	0.123393
10	-1073.074069	0.012215	0.126609
11	-1073.082204	0.010932	0.130875
12	-1073.088853	0.008209	0.133658
13	-1073.093294	0.006332	0.132882
14	-1073.095702	0.008822	0.113862
15	-1073.097415	0.008134	0.110163
16	-1073.098827	0.005603	0.111118
17	-1073.099887	0.003244	0.116913
18	-1073.100551	0.001553	0.144373
19	-1073.100958	0.000846	0.154690
20	-1073.101241	0.000826	0.169536
21	-1073.101467	0.000866	0.132014
22	-1073.101681	0.001374	0.130523
23	-1073.101926	0.001026	0.120991
24	-1073.102250	0.001613	0.129436
25	-1073.102632	0.002290	0.137072
26	-1073.102959	0.001303	0.146188
27	-1073.103140	0.000661	0.091098
28	-1073.103232	0.000886	0.102457
29	-1073.103315	0.001053	0.072466
30	-1073.103355	0.000642	0.047352
31	-1073.103379	0.000407	0.032468
32	-1073.103395	0.000337	0.081692

Reason for exit: Successful completion
Quantum Calculation CPU Time : 1:12:05.17
Quantum Calculation Wall Time: 9:33.39

SPARTAN '18 Properties Program: (x86/Darwin) build 1.3.0
Use of molecular symmetry disabled

Cartesian Coordinates (Angstroms)

Atom			X	Y	Z
1	C	C1	0.4338406	-0.2118767	-5.9455800
2	C	C2	0.9096536	0.1245998	-4.5681559
3	C	C3	0.2512824	-0.1584856	-3.4274147
4	C	C4	0.7607832	0.1827586	-2.1197729
5	C	C5	0.1336641	-0.0918903	-0.9535957
6	C	C6	0.7788875	0.2550000	0.3210752
7	O	O1	1.9287818	0.6318832	0.4222299
8	N	N2	-0.0064517	0.0789064	1.5153307
9	N	N1	0.6834009	-0.0863828	2.6861258
10	C	C10	-0.2223708	-0.1430270	3.6484066
11	C	C11	-1.3715449	0.1569871	1.7505208
12	C	C12	-1.5278630	0.0135895	3.1085345
13	C	C13	0.1771866	-0.3583500	5.0756578
14	C	C14	-2.4370446	0.4101048	0.7278169
15	H	H2	2.5382269	-0.0407444	2.9100588
16	Cl	Cl1	3.7720781	-0.1421979	3.3843425
17	H	H17	0.3371996	0.6940449	-6.5592465
18	H	H18	1.1560599	-0.8594515	-6.4611163
19	H	H19	-0.5345731	-0.7217530	-5.9284608
20	H	H20	1.8697454	0.6377351	-4.4977023
21	H	H21	-0.7084514	-0.6739046	-3.4753142
22	H	H22	1.7211828	0.6936128	-2.0680753
23	H	H23	-0.8150823	-0.6148446	-0.9492940
24	H	H24	-2.4646053	0.0357026	3.6487006
25	H	H25	1.2652903	-0.3608346	5.1738770
26	H	H26	-0.2309381	0.4297318	5.7188910
27	H	H27	-0.2047965	-1.3163375	5.4488430
28	H	H28	-2.1655200	1.2070177	0.0283869
29	H	H29	-3.3514217	0.7124239	1.2454977
30	H	H30	-2.6766005	-0.4840174	0.1394327

237	H28	S	0.00000	0.00000	0.00000	0.00000	0.00000
238	H28	S'	0.00000	0.00000	0.00000	0.00000	0.00000

MO:			61	62	63	64	65
Eigenvalues:			-0.08312	-0.01248	0.03837	0.05404	0.07493
(ev)			-2.26174	-0.33956	1.04407	1.47048	2.03897
			A	A	A	A	A
1	C1	S1	0.00008	0.00041	-0.00037	-0.00035	-0.00380
2	C1	S	-0.00068	-0.00099	0.00179	0.00207	0.01281
3	C1	PX	-0.01294	0.00087	0.00901	0.00272	0.04597
4	C1	PY	0.02387	-0.00280	-0.01211	0.00073	0.03259
5	C1	PZ	-0.00180	-0.00133	0.00147	0.00171	-0.00286
6	C1	S'	0.00337	-0.00433	-0.00574	-0.01209	-0.01877
7	C1	PX'	0.00617	-0.00895	0.00187	0.00204	0.10091
8	C1	PY'	-0.01239	0.01299	0.00617	0.00134	0.01613
9	C1	PZ'	0.00266	-0.00209	-0.00673	-0.00444	-0.03507
10	C1	DXX	0.00139	-0.00194	0.00323	0.00019	0.00104
11	C1	DYY	-0.00315	0.00320	-0.00455	-0.00004	0.00238
12	C1	DZZ	0.00181	-0.00122	0.00118	-0.00034	-0.00458
13	C1	DXY	-0.00056	0.00099	-0.00178	-0.00013	0.00252
14	C1	DXZ	0.00834	-0.00677	0.00824	0.00021	-0.00015
15	C1	DYZ	-0.01539	0.01239	-0.01473	-0.00060	0.00515
16	C2	S1	-0.00019	-0.00061	-0.00178	-0.00072	-0.02042
17	C2	S	0.00085	0.00097	0.00225	0.00158	0.03302
18	C2	PX	0.13717	-0.10247	0.11725	0.00507	0.05084
19	C2	PY	-0.25880	0.19166	-0.21037	-0.00653	0.10436
20	C2	PZ	0.01574	-0.01054	0.01576	0.00417	0.01908
21	C2	S'	-0.01153	-0.00140	0.04073	0.02300	0.29417
22	C2	PX'	0.14369	-0.13016	0.18282	-0.00810	0.05678
23	C2	PY'	-0.25905	0.24307	-0.35543	-0.02160	0.20994
24	C2	PZ'	0.01547	-0.01774	0.02205	-0.00636	-0.05035
25	C2	DXX	0.00129	-0.00426	0.00914	-0.00009	-0.01195
26	C2	DYY	-0.00086	0.00359	-0.00870	-0.00048	0.00034
27	C2	DZZ	-0.00056	0.00028	-0.00066	0.00042	0.00797
28	C2	DXY	-0.00092	0.00368	-0.00809	-0.00057	0.00097
29	C2	DXZ	-0.00173	0.00364	-0.00642	0.00036	0.00312

30	C2	DYZ	0.00310	-0.00778	0.01369	0.00089	-0.00369
31	C3	S1	-0.00017	0.00058	0.00188	0.00310	0.01687
32	C3	S	-0.00017	-0.00105	-0.00215	-0.00589	-0.02499
33	C3	PX	-0.04519	0.08894	-0.14551	0.00373	0.17782
34	C3	PY	0.08268	-0.17097	0.29972	0.01669	-0.04814
35	C3	PZ	-0.00587	0.00787	-0.01526	-0.00084	0.03906
36	C3	S'	0.00287	0.00123	-0.03675	-0.01851	-0.21083
37	C3	PX'	-0.03862	0.12674	-0.21986	0.01852	0.44201
38	C3	PY'	0.09179	-0.21574	0.46331	0.03534	-0.03260
39	C3	PZ'	-0.01694	0.00976	0.00469	0.03960	0.17693
40	C3	DXX	0.00100	-0.00344	0.01017	0.00078	-0.00030
41	C3	DYY	0.00085	0.00215	-0.00943	-0.00025	0.00947
42	C3	DZZ	-0.00230	0.00158	-0.00027	0.00111	-0.00502
43	C3	DXY	-0.00157	0.00284	-0.00811	-0.00022	0.00675
44	C3	DXZ	-0.01142	0.00535	-0.00077	-0.00037	-0.00347
45	C3	DYZ	0.02135	-0.01075	0.00211	-0.00017	0.00155
46	C4	S1	-0.00140	-0.00286	-0.00248	0.00260	-0.01232
47	C4	S	0.00050	0.00743	0.00763	0.00336	0.02635
48	C4	PX	-0.14829	0.01987	0.10042	0.00837	0.00349
49	C4	PY	0.26969	-0.04966	-0.17859	-0.00635	0.17566
50	C4	PZ	-0.00845	0.01056	0.01867	-0.00500	0.02070
51	C4	S'	0.05402	0.03876	-0.01246	-0.15690	-0.06584
52	C4	PX'	-0.18549	0.00882	0.17435	0.07371	0.01382
53	C4	PY'	0.27807	-0.04919	-0.28022	0.01709	0.33477
54	C4	PZ'	0.01316	0.03017	0.01353	-0.03311	0.09037
55	C4	DXX	-0.00116	0.00117	0.00557	0.00091	-0.01706
56	C4	DYY	-0.00035	0.00045	-0.00758	0.00064	0.00379
57	C4	DZZ	0.00172	-0.00140	0.00145	-0.00084	0.00808
58	C4	DXY	0.00086	-0.00114	-0.00423	-0.00032	0.00095
59	C4	DXZ	0.00524	-0.00854	0.00836	0.00025	0.00273
60	C4	DYZ	-0.01098	0.01566	-0.01312	-0.00039	0.00165
61	C5	S1	0.00031	0.00260	0.01318	0.00203	0.04528
62	C5	S	0.00736	-0.00232	-0.02560	0.00215	-0.07692
63	C5	PX	0.10272	-0.09368	0.03466	0.00100	0.22947
64	C5	PY	-0.17834	0.18731	-0.01040	0.00205	-0.02641
65	C5	PZ	0.01084	-0.01393	-0.01923	-0.00304	0.00248
66	C5	S'	-0.05635	-0.05943	-0.12686	-0.02994	-0.63660
67	C5	PX'	0.09790	-0.12372	0.05646	0.03889	0.61052
68	C5	PY'	-0.22184	0.18807	0.07752	0.02273	0.02893
69	C5	PZ'	0.05311	0.00798	-0.03002	-0.16686	-0.08847
70	C5	DXX	-0.00323	0.00093	0.00345	0.00035	0.00096
71	C5	DYY	0.00084	-0.00305	-0.00061	0.00123	0.00976
72	C5	DZZ	0.00308	0.00250	-0.00095	-0.00066	-0.00647
73	C5	DXY	0.00164	-0.00232	0.00027	0.00086	0.00891
74	C5	DXZ	0.01180	0.00052	-0.00924	0.00063	0.00211
75	C5	DYZ	-0.02270	-0.00410	0.01873	0.00142	-0.00293
76	C6	S1	0.00000	0.00339	0.00181	-0.00667	0.00145
77	C6	S	0.00325	-0.00384	-0.00562	-0.00494	-0.01890
78	C6	PX	0.08299	0.05666	-0.08720	-0.00686	-0.00458
79	C6	PY	-0.25493	-0.17086	0.26341	0.01535	0.10516
80	C6	PZ	0.01094	0.00537	-0.04439	-0.01108	-0.06420
81	C6	S'	-0.01626	-0.05257	-0.02993	0.19825	0.01036
82	C6	PX'	0.08822	0.02472	-0.11383	-0.09730	-0.15615
83	C6	PY'	-0.18728	-0.14874	0.23233	-0.02153	0.09911
84	C6	PZ'	0.00352	-0.02236	-0.03315	-0.00430	0.03741
85	C6	DXX	-0.01543	0.00059	0.01055	-0.00209	-0.00967
86	C6	DYY	0.01456	0.00311	-0.00582	-0.00077	-0.00135
87	C6	DZZ	0.00094	-0.00365	-0.00476	-0.00023	0.01008
88	C6	DXY	0.01859	0.01236	-0.00381	-0.00181	-0.00523
89	C6	DXZ	-0.00621	0.00437	0.00029	0.00084	-0.01089
90	C6	DYZ	0.00872	-0.01734	-0.00902	-0.00236	0.00202
91	O1	S1	0.00159	-0.00346	-0.00284	-0.00025	0.00163
92	O1	S	-0.00204	0.00573	0.00210	0.00326	-0.01772
93	O1	PX	-0.06934	-0.06030	0.02991	0.00563	0.05325
94	O1	PY	0.25131	0.10908	-0.16323	0.00348	-0.05108
95	O1	PZ	-0.01915	-0.01141	0.03382	0.02239	0.03476
96	O1	S'	-0.01973	0.04273	0.04515	0.00730	0.06023
97	O1	PX'	-0.06360	-0.06585	0.02876	0.01271	0.03154
98	O1	PY'	0.23777	0.11121	-0.17941	-0.00309	-0.06633
99	O1	PZ'	-0.01400	-0.00412	0.03295	0.00935	0.02684
100	O1	DXX	0.00154	-0.00155	0.00035	0.00123	-0.00229
101	O1	DYY	-0.00032	-0.00076	-0.00453	0.00227	-0.00422
102	O1	DZZ	0.00077	-0.00071	-0.00093	-0.00038	-0.00296
103	O1	DXY	-0.00203	0.00178	-0.00350	-0.00064	-0.00100
104	O1	DXZ	0.00013	0.00020	0.00084	0.00111	0.00395
105	O1	DYZ	0.00025	-0.00160	0.00037	-0.00119	0.00151
106	N2	S1	-0.00376	-0.00329	-0.00127	-0.00082	-0.00502
107	N2	S	0.00394	0.00424	0.00311	0.01257	0.01464
108	N2	PX	0.00124	-0.00989	-0.02177	-0.01081	0.02495
109	N2	PY	0.03922	-0.21452	-0.25862	-0.02422	-0.00420
110	N2	PZ	0.00123	-0.02610	-0.05183	0.00751	-0.04177
111	N2	S'	0.05766	0.04113	-0.01952	-0.07757	-0.03375
112	N2	PX'	0.04716	0.01671	-0.05541	-0.00526	0.04374
113	N2	PY'	0.01239	-0.24610	-0.26697	-0.04161	-0.02317
114	N2	PZ'	0.02398	-0.03586	-0.09041	0.06662	0.05007
115	N2	DXX	0.00187	0.00160	-0.00773	-0.00266	-0.00398
116	N2	DYY	-0.00225	-0.00465	0.00101	0.00814	0.00550
117	N2	DZZ	-0.00303	0.00133	0.00529	-0.00269	-0.00511
118	N2	DXY	-0.00705	-0.01052	0.00456	-0.02274	0.00620
119	N2	DXZ	-0.00475	-0.00302	0.00747	-0.00336	0.00848
120	N2	DYZ	0.01547	0.02015	-0.00452	-0.01173	-0.00296
121	N1	S1	0.00474	0.00402	-0.00607	0.00037	0.00150
122	N1	S	-0.00732	-0.00781	0.01160	0.00259	0.00278
123	N1	PX	0.01927	0.01832	-0.00932	-0.00908	-0.00623
124	N1	PY	0.08012	0.27648	0.19222	-0.22592	0.01311
125	N1	PZ	0.03875	0.04765	-0.00982	-0.01253	-0.00513
126	N1	S'	-0.07311	-0.05099	0.08748	-0.04673	-0.05638
127	N1	PX'	0.03368	0.03004	-0.03127	0.01069	0.01684
128	N1	PY'	0.08512	0.30772	0.23149	-0.27673	0.02207
129	N1	PZ'	0.06256	0.04662	-0.02559	-0.02798	0.01009
130	N1	DXX	-0.00024	0.00035	0.00159	-0.00197	-0.00101
131	N1	DYY	0.00286	0.00026	-0.00220	-0.00295	0.00266
132	N1	DZZ	-0.00057	0.00076	-0.00056	0.00504	0.00075
133	N1	DXY	-0.00221	-0.00206	0.00304	-0.00254	0.00173
134	N1	DXZ	-0.00208	-0.00034	0.00191	0.00197	-0.00259
135	N1	DYZ	-0.00455	0.00193	0.00651	0.02142	-0.00062
136	C10	S1	0.00008	0.00029	-0.00005	0.00081	-0.00865
137	C10	S	0.00040	-0.00054	-0.00118	-0.00414	0.01391
138	C10	PX	0.00151	-0.00546	-0.02117	0.03209	-0.01331
139	C10	PY	-0.10251	-0.19549	-0.10928	0.39453	-0.01231
140	C10	PZ	-0.01656	-0.02780	-0.01269	0.05678	-0.00878
141	C10	S'	0.00054	0.00733	0.00964	0.03524	0.13133
142	C10	PX'	-0.00575	0.00434	-0.03228	0.05895	-0.04513
143	C10	PY'	-0.09022	-0.20935	-0.14937	0.48768	-0.03480
144	C10	PZ'	-0.03972	-0.04802	-0.00647	0.04542	-0.00833
145	C10	DXX	0.00230	0.00298	-0.00076	0.00335	-0.00051
146	C10	DYY	-0.00023	-0.00039	-0.00026	-0.00451	-0.00003
147	C10	DZZ	-0.00186	-0.00194	0.00065	0.00177	-0.00197
148	C10	DXY	0.00619	0.01879	0.01173	0.01230	-0.00046
149	C10	DXZ	0.00039	0.00145	0.00081	0.00047	-0.00086
150	C10	DYZ	-0.00202	-0.00934	-0.00646	0.01434	0.00070
151	C11	S1	-0.00571	0.00041	0.00945	-0.00065	-0.00382
152	C11	S	0.01290	0.00010	-0.03025	-0.00267	-0.03426
153	C11	PX	0.02913	0.03809	-0.02085	0.01699	-0.03439
154	C11	PY	0.12002	0.31280	0.16715	0.23381	-0.02772
155	C11	PZ	0.00085	0.03659	0.03342	0.03217	-0.02159
156	C11	S'	0.09345	0.03048	-0.08301	0.05033	0.16016
157	C11	PX'	0.02001	0.04233	0.00378	0.05736	-0.06480
158	C11	PY'	0.10304	0.35002	0.19303	0.33663	-0.10075
159	C11	PZ'	0.02229	0.08360	0.01371	0.02843	-0.16165
160	C11	DXX	0.00101	-0.00136	-0.00313	0.00305	-0.00551
161	C11	DYY	0.00027	0.00256	0.00244	0.00461	0.00268
162	C11	DZZ	-0.00029	0.00003	-0.00020	-0.00798	-0.00478
163	C11	DXY	0.00279	-0.00936	-0.01567	0.00585	-0.00430
164	C11	DXZ	0.00146	-0.00107	-0.00503	-0.00148	-0.00702
165	C11	DYZ	-0.00295	-0.00329	0.00049	-0.02273	0.00236
166	C12	S1	0.00093	-0.00011	-0.00261	-0.00245	0.00559
167	C12	S	0.00206	0.00132	0.00263	0.00266	-0.01538
168	C12	PX	-0.00498	-0.01077	-0.01218	-0.03843	0.00706
169	C12	PY	-0.07826	-0.13508	-0.03460	-0.35761	0.02259
170	C12	PZ	0.00213	-0.01379	-0.01801	-0.02429	0.00485
171	C12	S'	-0.04358	-0.03224	0.03956	0.04818	-0.02951
172	C12	PX'	-0.01417	-0.01873	-0.03368	-0.09543	-0.01914
173	C12	PY'	-0.08107	-0.18017	-0.04628	-0.58014	0.08302

174	C12	PZ'	0.03047	0.00972	-0.04619	-0.01214	-0.06022
175	C12	DXX	-0.00016	-0.00090	-0.00120	0.00049	-0.00170
176	C12	DYY	0.00261	0.00533	0.00305	-0.00188	0.00165
177	C12	DZZ	-0.00194	-0.00499	-0.00290	0.00026	-0.00166
178	C12	DXY	-0.00273	-0.00766	-0.00697	0.01954	-0.00291
179	C12	DXZ	-0.00226	-0.00373	0.00066	0.00233	0.00468
180	C12	DYZ	-0.00729	-0.02017	-0.01222	-0.00316	0.00092
181	C13	S1	0.00171	0.00021	-0.00402	0.00329	0.00208
182	C13	S	-0.00441	-0.00076	0.00778	-0.00653	-0.00194
183	C13	PX	0.00107	-0.00060	-0.00431	0.00302	0.00253
184	C13	PY	0.01226	0.01407	0.00155	0.06815	-0.00662
185	C13	PZ	0.00803	0.00291	-0.00499	0.01693	0.00859
186	C13	S'	0.00561	0.00609	0.03445	-0.03015	-0.02635
187	C13	PX'	-0.00433	-0.01663	-0.01076	-0.00562	0.01534
188	C13	PY'	0.00502	0.01666	0.01571	0.25310	-0.00766
189	C13	PZ'	-0.00033	0.00461	0.00817	0.04508	0.02586
190	C13	DXX	0.00029	0.00087	0.00093	-0.00120	0.00017
191	C13	DYY	-0.00166	-0.00375	-0.00250	0.00798	-0.00004
192	C13	DZZ	0.00186	0.00290	0.00088	-0.00644	0.00070
193	C13	DXY	0.00068	0.00251	0.00154	0.00169	-0.00034
194	C13	DXZ	-0.00012	0.00068	0.00142	-0.00106	0.00063
195	C13	DYZ	0.00561	0.01172	0.00708	-0.02764	0.00076
196	C14	S1	-0.00677	0.00212	0.01328	0.00001	0.07036
197	C14	S	0.01878	-0.00465	-0.02083	0.00240	-0.07647
198	C14	PX	0.01393	0.00428	0.00912	-0.01315	0.05940
199	C14	PY	0.00993	0.00450	0.02917	0.04519	0.10978
200	C14	PZ	-0.01654	0.01515	0.01926	0.03479	0.06191
201	C14	S'	0.05328	-0.01558	-0.17548	-0.01425	-1.11008
202	C14	PX'	-0.03596	0.04282	0.06675	-0.07454	0.08718
203	C14	PY'	0.05025	0.06765	0.13491	0.10950	0.33742
204	C14	PZ'	-0.00894	0.00448	0.05422	0.10736	0.19481
205	C14	DXX	-0.00060	0.00185	0.00091	-0.00007	0.00341
206	C14	DYY	0.00135	-0.00396	-0.00190	-0.00155	0.00810
207	C14	DZZ	0.00045	0.00217	0.00369	0.00150	0.00871
208	C14	DXY	0.00406	0.01137	0.00644	0.01277	-0.00584
209	C14	DXZ	0.00350	0.00448	0.00170	0.00565	-0.00293
210	C14	DYZ	0.00427	0.01739	0.01190	0.01204	0.00153
211	H2	S	0.00268	0.00718	0.00806	-0.01101	-0.00146
212	H2	S'	-0.00836	0.01890	0.05734	-0.03956	0.00674
213	Cl1	S1	0.00011	-0.00034	-0.00089	0.00066	-0.00019
214	Cl1	S2	-0.00011	0.00196	0.00392	-0.00322	0.00049
215	Cl1	PX2	-0.00109	-0.00418	-0.00494	0.00458	0.00000
216	Cl1	PY2	0.00208	0.00600	0.00495	-0.00310	0.00101
217	Cl1	PZ2	0.00378	0.00157	-0.00379	0.00109	-0.00210
218	Cl1	S	0.00194	-0.00247	-0.00968	0.00644	-0.00307
219	Cl1	PX	0.00253	0.01085	0.01327	-0.01201	-0.00005
220	Cl1	PY	-0.00615	-0.01811	-0.01526	0.00936	-0.00328
221	Cl1	PZ	-0.01122	-0.00541	0.01113	-0.00246	0.00695
222	Cl1	S'	0.00016	-0.01307	-0.02527	0.02071	0.00017
223	Cl1	PX'	0.00054	0.01561	0.02967	-0.02448	0.00264
224	Cl1	PY'	0.00066	0.00214	0.00170	0.00009	0.00138
225	Cl1	PZ'	0.00021	0.00729	0.00903	-0.01027	-0.00289
226	Cl1	DXX	0.00108	0.00187	0.00153	-0.00253	-0.00039
227	Cl1	DYY	0.00019	-0.00036	-0.00129	0.00111	-0.00027
228	Cl1	DZZ	-0.00024	-0.00044	-0.00040	0.00068	-0.00005
229	Cl1	DXY	-0.00032	-0.00161	-0.00165	0.00150	-0.00016
230	Cl1	DXZ	-0.00007	0.00040	0.00156	-0.00158	0.00009
231	Cl1	DYZ	-0.00003	-0.00037	-0.00054	0.00068	-0.00013
232	H17	S	0.05712	-0.04193	0.04637	0.00191	-0.01751
233	H17	S'	0.11863	-0.10172	0.11411	0.00239	-0.11368
234	H18	S	-0.05736	0.04258	-0.04715	-0.00133	0.00615
235	H18	S'	-0.11837	0.10557	-0.12576	-0.00597	-0.07531
236	H19	S	0.00021	-0.00039	0.00118	-0.00009	0.01603
237	H19	S'	-0.00029	-0.00098	0.00806	0.00539	0.18284
238	H20	S	0.00052	0.00031	-0.00206	-0.00222	-0.03674
239	H20	S'	-0.00065	0.00149	-0.02274	-0.00563	-0.45743
240	H21	S	-0.00027	-0.00009	-0.00127	0.00082	0.02683
241	H21	S'	0.00240	0.00702	0.05130	0.05825	0.64095
242	H22	S	-0.00171	-0.00037	-0.00389	0.00087	-0.05151
243	H22	S'	0.01451	0.00243	-0.04927	-0.02229	-0.40506
244	H23	S	-0.00647	-0.00785	0.01325	0.01111	0.05116
245	H23	S'	0.00118	-0.00980	0.17077	0.06140	1.00491
246	H24	S	0.00494	0.00291	-0.00717	-0.00401	-0.01505
247	H24	S'	-0.00054	0.00084	-0.03344	-0.08645	0.02320
248	H25	S	0.00066	0.00351	0.00538	-0.00708	0.00168
249	H25	S'	0.00433	0.01800	0.01101	-0.00657	-0.00067
250	H26	S	0.01992	0.03460	0.01638	-0.07941	0.00173
251	H26	S'	0.03209	0.04440	-0.00037	-0.39973	0.01389
252	H27	S	-0.01961	-0.03802	-0.02259	0.08637	-0.00257
253	H27	S'	-0.02814	-0.06249	-0.06130	0.44216	-0.01753
254	H28	S	-0.00482	-0.05535	-0.03619	-0.03674	0.00613
255	H28	S'	-0.08787	-0.15635	-0.08638	-0.07175	0.21161
256	H29	S	-0.01839	-0.01155	-0.00562	-0.02321	0.01422
257	H29	S'	-0.07609	-0.01688	0.03246	-0.21202	0.30751
258	H30	S	0.02603	0.06390	0.04731	0.05736	0.02150
259	H30	S'	0.05708	0.21892	0.31471	0.29216	0.83979

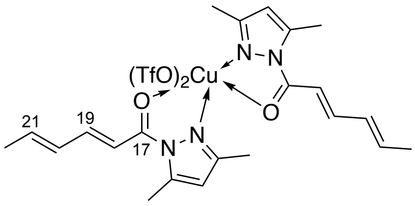

4a•Cu(OTf)$_2$•4a

SPARTAN '18 Quantum Mechanics Program: (x86/Darwin) build 1.3.0

Job type: Geometry optimization.
Method: UB3LYP
Basis set: 6-311G**
Number of basis functions: 1014
Number of electrons: 379 (1 unpaired)
Parallel Job: 8 threads

SCF model:
An unrestricted hybrid HF-DFT SCF calculation will be performed using Pulay DIIS + Geometric Direct Minimization

Optimization:

Step	Energy	Max Grad.	Max Dist.
1	-4788.717519	0.097541	0.163819
2	-4788.771084	0.064680	0.155669
3	-4788.799830	0.033522	0.113838
4	-4788.810099	0.018370	0.064226
5	-4788.812929	0.011941	0.077012
6	-4788.814283	0.007557	0.119958
7	-4788.814875	0.004871	0.038904
8	-4788.815046	0.002814	0.038054
9	-4788.815140	0.002109	0.015197
10	-4788.815197	0.001544	0.019733
11	-4788.815226	0.001057	0.020112
12	-4788.815195	0.000730	0.015057
13	-4788.815265	0.000665	0.030911
14	-4788.815273	0.000489	0.015099

Reason for exit: Successful completion
Quantum Calculation CPU Time : 53:00:09.84
Quantum Calculation Wall Time: 6:49:48.46

SPARTAN '18 Properties Program: (x86/Darwin) build 1.3.0
Use of molecular symmetry enabled

Cartesian Coordinates (Angstroms)

	Atom		X	Y	Z
1	N	N1	1.9106751	0.0000001	-0.7304400
2	C	C2	3.1074934	-0.0000009	-0.1761514
3	C	C3	4.0928643	-0.0000015	-1.1905566
4	C	C4	3.4310050	-0.0000009	-2.3918266
5	N	N2	2.0801197	0.0000002	-2.0887339
6	C	C1	0.8819120	0.0000010	-2.8639818
7	C	C5	0.9675530	0.0000035	-4.3167456
8	O	O1	-0.1786319	-0.0000001	-2.2588862
9	C	C7	-0.1698259	-0.0000008	-5.0451798
10	C	C8	-0.2227965	0.0000024	-6.4841672
11	C	C9	-1.3817797	-0.0000024	-7.1652179
12	C	C10	-1.5143974	0.0000011	-8.6517922
13	C	C11	3.2889127	-0.0000016	1.3048727
14	C	C12	4.0553286	-0.0000025	-3.7484613
15	Cu	Cu1	0.0328566	0.0000005	0.0672101
16	O	O2	0.2496030	0.0000005	2.4226810
17	N	N3	-1.8561350	0.0000003	0.8568840
18	C	C13	-3.0557005	-0.0000002	0.3123068
19	C	C14	-4.0363557	-0.0000016	1.3397205
20	C	C15	-3.3736375	-0.0000021	2.5337857
21	N	N4	-2.0152473	-0.0000007	2.2245249
22	C	C17	-0.8219232	0.0000001	3.0030723
23	C	C18	-0.9279314	0.0000006	4.4631844
24	C	C19	0.1966284	0.0000009	5.2104894
25	C	C20	0.2248833	0.0000017	6.6513734
26	C	C21	1.3694417	0.0000013	7.3563045
27	C	C22	1.4705159	0.0000017	8.8454943
28	C	C23	-3.2880232	0.0000006	-1.1576513
29	C	C24	-4.0025182	-0.0000042	3.8898601
30	O	O3	0.0833236	2.0047473	0.2472795
31	S	S1	-0.1906970	3.0354538	-0.8356129
32	O	O5	0.8322668	3.0631507	-1.8821785
33	O	O4	-1.5943218	3.0919556	-1.2410422
34	C	C26	0.0980176	4.5866712	0.1771345
35	F	F1	1.3441204	4.6111150	0.6651795
36	F	F3	-0.7606260	4.6482803	1.2026510
37	F	F2	-0.0797880	5.6674941	-0.5906062
38	O	O6	0.0833236	-2.0047450	0.2472794
39	S	S2	-0.1906972	-3.0354519	-0.8356125
40	O	O7	0.8322672	-3.0631505	-1.8821774
41	O	O8	-1.5943217	-3.0919528	-1.2410428
42	C	C6	0.0980150	-4.5866690	0.1771359
43	F	F4	-0.7606301	-4.6482770	1.2026513
44	F	F5	1.3441171	-4.6111134	0.6651826
45	F	F6	-0.0797904	-5.6674923	-0.5906044
46	H	H46	5.1622623	-0.0000026	-1.0551067
47	H	H47	1.9254375	0.0000097	-4.8071167
48	H	H48	-1.1141238	-0.0000071	-4.5090790
49	H	H49	0.7199048	0.0000090	-7.0274109
50	H	H50	-2.3102925	-0.0000091	-6.5968838
51	H	H51	-2.0777399	-0.8773681	-8.9893195
52	H	H52	-0.5433100	0.0000066	-9.1507528
53	H	H53	-2.0777468	0.8773678	-8.9893147
54	H	H54	4.3494603	-0.0000053	1.5584327
55	H	H55	2.8158672	-0.8801267	1.7449324
56	H	H56	2.8158732	0.8801266	1.7449325
57	H	H57	3.7864609	-0.8886126	-4.3244912
58	H	H58	3.7864673	0.8886099	-4.3244906
59	H	H59	5.1380791	-0.0000063	-3.6227124
60	H	H60	-5.1063454	-0.0000023	1.2079088
61	H	H61	-1.8917992	0.0000012	4.9421568
62	H	H62	1.1500484	0.0000003	4.6897673
63	H	H63	-0.7280255	0.0000025	7.1764641
64	H	H64	2.3101397	0.0000005	6.8080454
65	H	H65	2.0265907	-0.8771491	9.1952066
66	H	H66	2.0265898	0.8771533	9.1952063
67	H	H67	0.4890179	0.0000011	9.3233712
68	H	H68	-3.8569763	0.8899074	-1.4383929
69	H	H69	-3.8569743	-0.8899071	-1.4383941
70	H	H70	-2.3526147	0.0000020	-1.7088178
71	H	H71	-3.7406722	-0.8878997	4.4706182
72	H	H72	-3.7406768	0.8878921	4.4706190
73	H	H73	-5.0843701	-0.0000070	3.7570327

MO:			191	192	193	194	195
Eigenvalues:			-0.10370	-0.09380	-0.03530	-0.02810	0.02002
(ev)			-2.82176	-2.55235	-0.96055	-0.76466	0.54473
			A″	A″	A″	A″	A″
1	N1	S1	0.00000	-0.00000	0.00000	0.00000	-0.00000
2	N1	S	0.00000	-0.00000	0.00000	0.00000	-0.00000
3	N1	PX	0.00000	0.00000	0.00000	0.00000	-0.00000
4	N1	PY	0.00630	0.05207	0.02268	0.12778	-0.00815
5	N1	PZ	-0.00000	-0.00000	0.00000	0.00000	-0.00000
6	N1	S'	-0.00000	0.00000	-0.00000	-0.00000	0.00000
7	N1	PX'	0.00000	0.00000	0.00000	0.00000	-0.00000
8	N1	PY'	0.00814	0.07382	0.03146	0.18320	-0.01334
9	N1	PZ'	-0.00000	-0.00000	0.00000	0.00000	0.00000
10	N1	S″	0.00000	0.00000	-0.00000	-0.00000	0.00000
11	N1	PX″	0.00000	0.00000	0.00000	0.00000	-0.00000
12	N1	PY″	0.01817	0.11678	0.05652	0.29157	-0.01291
13	N1	PZ″	-0.00000	-0.00000	0.00000	0.00000	-0.00000
14	N1	DZ**2	0.00000	-0.00000	-0.00000	-0.00000	0.00000
15	N1	DXX-YY	0.00000	0.00000	0.00000	-0.00000	-0.00000
16	N1	DXY	-0.00054	-0.00229	-0.00126	-0.00298	0.00055
17	N1	DXZ	-0.00000	-0.00000	0.00000	0.00000	0.00000
18	N1	DYZ	-0.00034	-0.00661	0.00057	0.00160	0.00015
19	C2	S1	-0.00000	-0.00000	-0.00000	-0.00000	-0.00000
20	C2	S	-0.00000	-0.00000	-0.00000	-0.00000	-0.00000
21	C2	PX	-0.00000	-0.00000	-0.00000	-0.00000	0.00000
22	C2	PY	-0.00822	-0.06510	-0.01986	-0.09529	0.01054
23	C2	PZ	-0.00000	-0.00000	-0.00000	-0.00000	0.00000
24	C2	S'	0.00000	-0.00000	0.00000	0.00000	0.00000
25	C2	PX'	-0.00000	-0.00000	-0.00000	-0.00000	0.00000
26	C2	PY'	-0.01209	-0.10255	-0.03131	-0.14859	0.01356
27	C2	PZ'	-0.00000	-0.00000	-0.00000	-0.00000	0.00000
28	C2	S″	0.00000	0.00000	-0.00000	0.00000	0.00000
29	C2	PX″	0.00000	0.00000	0.00000	0.00000	-0.00000
30	C2	PY″	-0.01507	-0.12570	-0.04114	-0.20837	0.02911
31	C2	PZ″	-0.00000	-0.00000	-0.00000	-0.00000	0.00000
32	C2	DZ**2	0.00000	0.00000	0.00000	-0.00000	-0.00000
33	C2	DXX-YY	-0.00000	-0.00000	-0.00000	-0.00000	0.00000
34	C2	DXY	-0.00101	-0.01031	-0.00404	-0.02489	0.00090
35	C2	DXZ	-0.00000	0.00000	-0.00000	-0.00000	0.00000
36	C2	DYZ	-0.00017	0.00071	-0.00042	-0.00176	0.00174
37	C3	S1	0.00000	0.00000	0.00000	0.00000	0.00000
38	C3	S	0.00000	0.00000	0.00000	0.00000	0.00000
39	C3	PX	0.00000	-0.00000	-0.00000	-0.00000	-0.00000
40	C3	PY	-0.00150	-0.03834	-0.00509	-0.04609	-0.00979
41	C3	PZ	0.00000	-0.00000	0.00000	0.00000	-0.00000
42	C3	S'	-0.00000	-0.00000	-0.00000	-0.00000	-0.00000
43	C3	PX'	0.00000	-0.00000	-0.00000	-0.00000	0.00000
44	C3	PY'	-0.00252	-0.05813	-0.00694	-0.06478	-0.01301
45	C3	PZ'	0.00000	-0.00000	0.00000	0.00000	-0.00000
46	C3	S″	-0.00000	-0.00000	0.00000	0.00000	-0.00000
47	C3	PX″	0.00000	-0.00000	-0.00000	-0.00000	0.00000
48	C3	PY″	-0.00254	-0.08831	-0.01947	-0.15612	-0.03590
49	C3	PZ″	-0.00000	-0.00000	-0.00000	-0.00000	-0.00000
50	C3	DZ**2	-0.00000	0.00000	-0.00000	0.00000	0.00000
51	C3	DXX-YY	0.00000	0.00000	-0.00000	-0.00000	-0.00000
52	C3	DXY	0.00009	0.00033	0.00040	0.00053	-0.00110
53	C3	DXZ	-0.00000	-0.00000	-0.00000	-0.00000	-0.00000
54	C3	DYZ	-0.00176	-0.01463	-0.00525	-0.02812	0.00096
55	C4	S1	0.00000	0.00000	-0.00000	-0.00000	-0.00000
56	C4	S	0.00000	0.00000	-0.00000	-0.00000	-0.00000
57	C4	PX	0.00000	0.00000	0.00000	0.00000	0.00000
58	C4	PY	0.00779	0.07367	0.02386	0.13692	0.00400
59	C4	PZ	-0.00000	-0.00000	-0.00000	-0.00000	0.00000
60	C4	S'	-0.00000	-0.00000	0.00000	0.00000	0.00000

61	C4	PX'	0.00000	0.00000	0.00000	0.00000	0.00000
62	C4	PY'	0.01260	0.11721	0.03617	0.20711	0.00630
63	C4	PZ'	-0.00000	-0.00000	-0.00000	-0.00000	-0.00000
64	C4	S"	-0.00000	-0.00000	0.00000	-0.00000	0.00000
65	C4	PX"	0.00000	0.00000	-0.00000	-0.00000	-0.00000
66	C4	PY"	0.01357	0.15305	0.06281	0.37086	0.01739
67	C4	PZ"	0.00000	0.00000	-0.00000	-0.00000	0.00000
68	C4	DZ**2	0.00000	-0.00000	-0.00000	-0.00000	-0.00000
69	C4	DXX-YY	0.00000	-0.00000	0.00000	0.00000	0.00000
70	C4	DXY	0.00004	-0.00529	0.00206	0.00884	-0.00231
71	C4	DXZ	-0.00000	0.00000	-0.00000	-0.00000	0.00000
72	C4	DYZ	0.00008	-0.00100	-0.00081	-0.00626	-0.00104
73	N2	S1	0.00000	0.00000	-0.00000	-0.00000	-0.00000
74	N2	S	0.00000	0.00000	-0.00000	-0.00000	-0.00000
75	N2	PX	0.00000	0.00000	-0.00000	-0.00000	-0.00000
76	N2	PY	-0.00128	0.01232	-0.02094	-0.10898	0.00623
77	N2	PZ	0.00000	0.00000	0.00000	-0.00000	-0.00000
78	N2	S'	0.00000	-0.00000	0.00000	0.00000	0.00000
79	N2	PX'	-0.00000	0.00000	-0.00000	-0.00000	0.00000
80	N2	PY'	-0.00237	0.02285	-0.03048	-0.15661	0.00994
81	N2	PZ'	0.00000	0.00000	-0.00000	-0.00000	-0.00000
82	N2	S"	-0.00000	-0.00000	0.00000	-0.00000	-0.00000
83	N2	PX"	0.00000	0.00000	-0.00000	-0.00000	-0.00000
84	N2	PY"	-0.00181	0.00399	-0.04901	-0.26195	0.00901
85	N2	PZ"	-0.00000	-0.00000	0.00000	0.00000	-0.00000
86	N2	DZ**2	0.00000	0.00000	-0.00000	-0.00000	0.00000
87	N2	DXX-YY	0.00000	0.00000	0.00000	0.00000	0.00000
88	N2	DXY	0.00136	0.01471	0.00241	0.01520	0.00153
89	N2	DXZ	0.00000	0.00000	0.00000	-0.00000	-0.00000
90	N2	DYZ	0.00107	0.01246	0.00158	0.01039	-0.00053
91	C1	S1	0.00000	0.00000	-0.00000	-0.00000	0.00000
92	C1	S	0.00000	0.00000	-0.00000	-0.00000	0.00000
93	C1	PX	0.00000	0.00000	-0.00000	-0.00000	-0.00000
94	C1	PY	-0.01097	-0.13396	-0.00710	-0.05237	-0.01080
95	C1	PZ	-0.00000	-0.00000	-0.00000	-0.00000	-0.00000
96	C1	S'	-0.00000	-0.00000	0.00000	0.00000	-0.00000
97	C1	PX'	0.00000	0.00000	0.00000	-0.00000	-0.00000
98	C1	PY'	-0.01900	-0.21984	-0.01201	-0.08657	-0.01775
99	C1	PZ'	-0.00000	-0.00000	-0.00000	-0.00000	-0.00000
100	C1	S"	0.00000	-0.00000	-0.00000	-0.00000	0.00000
101	C1	PX"	0.00000	0.00000	0.00000	-0.00000	-0.00000
102	C1	PY"	-0.01734	-0.22569	-0.01217	-0.10031	-0.01999
103	C1	PZ"	0.00000	-0.00000	-0.00000	-0.00000	0.00000
104	C1	DZ**2	0.00000	0.00000	-0.00000	-0.00000	0.00000
105	C1	DXX-YY	0.00000	0.00000	-0.00000	-0.00000	0.00000
106	C1	DXY	-0.00169	-0.02259	-0.00233	-0.01714	-0.00029
107	C1	DXZ	-0.00000	-0.00000	0.00000	0.00000	-0.00000
108	C1	DYZ	0.00099	0.01947	-0.00428	-0.02065	0.00016
109	C5	S1	-0.00000	-0.00000	-0.00000	-0.00000	0.00000
110	C5	S	-0.00000	0.00000	-0.00000	0.00000	0.00000
111	C5	PX	0.00000	0.00000	-0.00000	-0.00000	-0.00000
112	C5	PY	-0.00450	-0.07587	0.02070	0.10311	0.00346
113	C5	PZ	-0.00000	-0.00000	0.00000	0.00000	0.00000
114	C5	S'	-0.00000	-0.00000	0.00000	-0.00000	-0.00000
115	C5	PX'	0.00000	0.00000	-0.00000	-0.00000	-0.00000
116	C5	PY'	-0.00639	-0.11535	0.03158	0.15757	0.00490
117	C5	PZ'	-0.00000	-0.00000	0.00000	0.00000	0.00000
118	C5	S"	0.00000	0.00000	0.00000	0.00000	0.00000
119	C5	PX"	-0.00000	-0.00000	-0.00000	-0.00000	-0.00000
120	C5	PY"	-0.01282	-0.19299	0.05010	0.24962	0.00652
121	C5	PZ"	-0.00000	-0.00000	0.00000	0.00000	0.00000
122	C5	DZ**2	-0.00000	-0.00000	0.00000	0.00000	-0.00000
123	C5	DXX-YY	0.00000	0.00000	-0.00000	-0.00000	0.00000
124	C5	DXY	-0.00129	-0.01703	0.00110	0.00376	-0.00053
125	C5	DXZ	0.00000	0.00000	-0.00000	-0.00000	-0.00000
126	C5	DYZ	-0.00208	-0.02795	0.00069	0.00049	-0.00155
127	O1	S1	-0.00000	-0.00000	0.00000	0.00000	-0.00000
128	O1	S	-0.00000	-0.00000	0.00000	0.00000	-0.00000
129	O1	PX	0.00000	0.00000	-0.00000	-0.00000	-0.00000
130	O1	PY	0.00899	0.11507	0.00190	0.03155	0.00483
131	O1	PZ	0.00000	0.00000	0.00000	0.00000	0.00000
132	O1	S'	0.00000	0.00000	-0.00000	-0.00000	0.00000
133	O1	PX'	0.00000	0.00000	-0.00000	-0.00000	0.00000
134	O1	PY'	0.01110	0.15955	0.00223	0.04280	0.00766
135	O1	PZ'	0.00000	0.00000	0.00000	0.00000	0.00000
136	O1	S"	0.00000	0.00000	-0.00000	-0.00000	0.00000
137	O1	PX"	0.00000	0.00000	0.00000	-0.00000	-0.00000
138	O1	PY"	0.02374	0.22303	0.00719	0.07056	0.00712
139	O1	PZ"	-0.00000	0.00000	0.00000	0.00000	0.00000
140	O1	DZ**2	-0.00000	0.00000	-0.00000	0.00000	0.00000
141	O1	DXX-YY	0.00000	0.00000	0.00000	0.00000	-0.00000
142	O1	DXY	-0.00023	-0.00272	-0.00049	-0.00218	-0.00065
143	O1	DXZ	-0.00000	-0.00000	-0.00000	-0.00000	0.00000
144	O1	DYZ	0.00034	0.00314	0.00033	0.00085	0.00044
145	C7	S1	0.00000	0.00000	0.00000	0.00000	-0.00000
146	C7	S	0.00000	0.00000	0.00000	0.00000	-0.00000
147	C7	PX	-0.00000	-0.00000	0.00000	0.00000	-0.00000
148	C7	PY	0.01025	0.13935	-0.01109	-0.04414	0.00461
149	C7	PZ	0.00000	0.00000	-0.00000	-0.00000	0.00000
150	C7	S'	-0.00000	-0.00000	-0.00000	-0.00000	-0.00000
151	C7	PX'	-0.00000	-0.00000	0.00000	0.00000	-0.00000
152	C7	PY'	0.01509	0.20620	-0.01736	-0.06986	0.00615
153	C7	PZ'	0.00000	0.00000	-0.00000	-0.00000	0.00000
154	C7	S"	-0.00000	-0.00000	-0.00000	-0.00000	0.00000
155	C7	PX"	-0.00000	-0.00000	-0.00000	-0.00000	-0.00000
156	C7	PY"	0.02393	0.32085	-0.02481	-0.09544	0.01751
157	C7	PZ"	0.00000	0.00000	-0.00000	-0.00000	0.00000
158	C7	DZ**2	-0.00000	-0.00000	0.00000	0.00000	0.00000
159	C7	DXX-YY	0.00000	0.00000	-0.00000	-0.00000	0.00000
160	C7	DXY	-0.00028	-0.00575	0.00226	0.01144	0.00032
161	C7	DXZ	-0.00000	0.00000	-0.00000	-0.00000	-0.00000
162	C7	DYZ	-0.00057	-0.01094	0.00368	0.01921	0.00154
163	C8	S1	-0.00000	-0.00000	0.00000	0.00000	0.00000
164	C8	S	-0.00000	-0.00000	0.00000	0.00000	0.00000
165	C8	PX	-0.00000	-0.00000	0.00000	0.00000	0.00000
166	C8	PY	0.00155	0.03169	-0.01530	-0.08307	-0.01051
167	C8	PZ	0.00000	0.00000	-0.00000	-0.00000	-0.00000
168	C8	S'	-0.00000	0.00000	-0.00000	-0.00000	-0.00000
169	C8	PX'	-0.00000	-0.00000	0.00000	0.00000	0.00000
170	C8	PY'	0.00243	0.04925	-0.02250	-0.12156	-0.01488
171	C8	PZ'	0.00000	0.00000	-0.00000	-0.00000	-0.00000
172	C8	S"	0.00000	-0.00000	-0.00000	-0.00000	-0.00000
173	C8	PX"	0.00000	0.00000	0.00000	0.00000	0.00000
174	C8	PY"	0.00305	0.07015	-0.04385	-0.24369	-0.03697
175	C8	PZ"	0.00000	0.00000	-0.00000	-0.00000	0.00000
176	C8	DZ**2	0.00000	0.00000	-0.00000	-0.00000	-0.00000
177	C8	DXX-YY	-0.00000	-0.00000	0.00000	0.00000	-0.00000
178	C8	DXY	0.00101	0.01433	-0.00235	-0.01180	-0.00110
179	C8	DXZ	-0.00000	-0.00000	0.00000	0.00000	-0.00000
180	C8	DYZ	0.00180	0.02538	-0.00307	-0.01397	-0.00001
181	C9	S1	-0.00000	-0.00000	0.00000	0.00000	-0.00000
182	C9	S	-0.00000	-0.00000	0.00000	0.00000	-0.00000
183	C9	PX	0.00000	0.00000	-0.00000	-0.00000	-0.00000
184	C9	PY	-0.00886	-0.12616	0.02129	0.10656	0.00852
185	C9	PZ	-0.00000	-0.00000	0.00000	0.00000	0.00000
186	C9	S'	-0.00000	-0.00000	-0.00000	-0.00000	0.00000
187	C9	PX'	0.00000	0.00000	-0.00000	-0.00000	-0.00000
188	C9	PY'	-0.01316	-0.18712	0.03105	0.15483	0.01169
189	C9	PZ'	-0.00000	-0.00000	0.00000	0.00000	0.00000
190	C9	S"	0.00000	0.00000	0.00000	0.00000	0.00000
191	C9	PX"	0.00000	0.00000	-0.00000	-0.00000	-0.00000
192	C9	PY"	-0.01890	-0.27794	0.05863	0.30298	0.03207
193	C9	PZ"	-0.00000	-0.00000	0.00000	0.00000	0.00000
194	C9	DZ**2	0.00000	0.00000	-0.00000	-0.00000	0.00000
195	C9	DXX-YY	-0.00000	-0.00000	0.00000	0.00000	0.00000
196	C9	DXY	0.00009	0.00247	-0.00182	-0.01014	-0.00151
197	C9	DXZ	-0.00000	-0.00000	0.00000	0.00000	0.00000
198	C9	DYZ	0.00019	0.00344	-0.00132	-0.00700	-0.00074
199	C10	S1	0.00000	0.00000	-0.00000	-0.00000	-0.00000
200	C10	S	0.00000	0.00000	-0.00000	-0.00000	-0.00000
201	C10	PX	-0.00000	-0.00000	0.00000	0.00000	-0.00000
202	C10	PY	0.00105	0.01375	-0.00103	-0.00428	0.00016
203	C10	PZ	0.00000	0.00000	0.00000	0.00000	0.00000
204	C10	S'	0.00000	0.00000	0.00000	0.00000	0.00000

205	C10	PX'	-0.00000	-0.00000	0.00000	0.00000	-0.00000
206	C10	PY'	0.00133	0.01714	-0.00101	-0.00377	0.00049
207	C10	PZ'	0.00000	0.00000	0.00000	0.00000	0.00000
208	C10	S″	-0.00000	-0.00000	0.00000	0.00000	0.00000
209	C10	PX″	-0.00000	-0.00000	-0.00000	-0.00000	-0.00000
210	C10	PY″	0.00053	0.00619	-0.00119	-0.00742	-0.00340
211	C10	PZ″	-0.00000	-0.00000	0.00000	0.00000	0.00000
212	C10	DZ**2	-0.00000	-0.00000	0.00000	0.00000	0.00000
213	C10	DXX-YY	0.00000	-0.00000	0.00000	0.00000	0.00000
214	C10	DXY	-0.00047	-0.00662	0.00116	0.00591	0.00057
215	C10	DXZ	0.00000	0.00000	-0.00000	-0.00000	-0.00000
216	C10	DYZ	-0.00125	-0.01810	0.00339	0.01725	0.00157
217	C11	S1	-0.00000	-0.00000	-0.00000	0.00000	0.00000
218	C11	S	-0.00000	-0.00000	-0.00000	0.00000	0.00000
219	C11	PX	-0.00000	-0.00000	-0.00000	-0.00000	0.00000
220	C11	PY	-0.00571	0.00651	-0.00390	-0.00053	0.01083
221	C11	PZ	0.00000	-0.00000	0.00000	0.00000	-0.00000
222	C11	S'	0.00000	0.00000	-0.00000	-0.00000	-0.00000
223	C11	PX'	-0.00000	-0.00000	-0.00000	-0.00000	0.00000
224	C11	PY'	-0.00482	0.00891	-0.00374	-0.00220	0.00853
225	C11	PZ'	0.00000	-0.00000	0.00000	0.00000	-0.00000
226	C11	S″	0.00000	-0.00000	0.00000	-0.00000	-0.00000
227	C11	PX″	-0.00000	-0.00000	-0.00000	-0.00000	0.00000
228	C11	PY″	-0.03989	-0.01944	-0.03884	-0.07378	0.07386
229	C11	PZ″	0.00000	0.00000	0.00000	0.00000	0.00000
230	C11	DZ**2	-0.00000	0.00000	0.00000	0.00000	0.00000
231	C11	DXX-YY	0.00000	-0.00000	-0.00000	-0.00000	-0.00000
232	C11	DXY	-0.00012	-0.00189	-0.00087	-0.00419	0.00134
233	C11	DXZ	0.00000	-0.00000	0.00000	0.00000	-0.00000
234	C11	DYZ	0.00096	0.00917	0.00317	0.01499	-0.00256
235	C12	S1	0.00000	0.00000	-0.00000	-0.00000	0.00000
236	C12	S	0.00000	0.00000	-0.00000	-0.00000	0.00000
237	C12	PX	0.00000	0.00000	-0.00000	-0.00000	-0.00000
238	C12	PY	-0.00004	0.00360	-0.00143	-0.00501	-0.00007
239	C12	PZ	0.00000	0.00000	-0.00000	-0.00000	0.00000
240	C12	S'	-0.00000	-0.00000	0.00000	0.00000	-0.00000
241	C12	PX'	0.00000	0.00000	-0.00000	-0.00000	-0.00000
242	C12	PY'	0.00005	0.00583	-0.00143	-0.00424	-0.00001
243	C12	PZ'	0.00000	0.00000	-0.00000	-0.00000	0.00000
244	C12	S″	0.00000	0.00000	0.00000	0.00000	-0.00000
245	C12	PX″	-0.00000	-0.00000	0.00000	0.00000	-0.00000
246	C12	PY″	0.00378	0.03119	0.00144	0.01334	-0.00698
247	C12	PZ″	0.00000	0.00000	-0.00000	-0.00000	0.00000
248	C12	DZ**2	-0.00000	-0.00000	-0.00000	-0.00000	0.00000
249	C12	DXX-YY	-0.00000	0.00000	0.00000	0.00000	0.00000
250	C12	DXY	-0.00019	-0.00141	-0.00066	-0.00376	-0.00023
251	C12	DXZ	0.00000	0.00000	0.00000	0.00000	0.00000
252	C12	DYZ	0.00115	0.01138	0.00415	0.02448	0.00081
253	Cu1	S	0.00000	-0.00000	0.00000	-0.00000	0.00000
254	Cu1	S	-0.00000	0.00000	-0.00000	0.00000	-0.00000
255	Cu1	S	0.00000	-0.00000	0.00000	-0.00000	0.00000
256	Cu1	S	0.00000	-0.00000	0.00000	-0.00000	0.00000
257	Cu1	S	-0.00000	0.00000	-0.00000	0.00000	0.00000
258	Cu1	S	0.00000	-0.00000	0.00000	0.00000	-0.00000
259	Cu1	PX	0.00000	-0.00000	0.00000	-0.00000	-0.00000
260	Cu1	PY	0.00229	0.00212	0.00490	0.00362	0.00194
261	Cu1	PZ	0.00000	-0.00000	0.00000	-0.00000	-0.00000
262	Cu1	PX	-0.00000	0.00000	-0.00000	0.00000	0.00000
263	Cu1	PY	-0.00636	-0.00589	-0.01378	-0.01016	-0.00548
264	Cu1	PZ	-0.00000	0.00000	-0.00000	0.00000	0.00000
265	Cu1	PX	0.00000	-0.00000	-0.00000	0.00000	0.00000
266	Cu1	PY	-0.00116	-0.00123	-0.00160	-0.00156	-0.00065
267	Cu1	PZ	0.00000	0.00000	-0.00000	0.00000	0.00000
268	Cu1	PX	-0.00000	0.00000	0.00000	-0.00000	-0.00000
269	Cu1	PY	0.01931	0.02133	0.03897	0.03174	0.01713
270	Cu1	PZ	0.00000	-0.00000	0.00000	-0.00000	-0.00000
271	Cu1	DZ**2	0.00000	-0.00000	-0.00000	-0.00000	0.00000
272	Cu1	DXX-YY	0.00000	0.00000	0.00000	0.00000	0.00000
273	Cu1	DXY	0.00592	-0.00937	0.02114	-0.03360	0.02417
274	Cu1	DXZ	-0.00000	-0.00000	-0.00000	-0.00000	0.00000
275	Cu1	DYZ	-0.01766	0.02637	-0.01123	0.01866	0.00158
276	Cu1	DZ**2	0.00000	-0.00000	-0.00000	-0.00000	0.00000
277	Cu1	DXX-YY	0.00000	0.00000	0.00000	0.00000	0.00000
278	Cu1	DXY	0.00290	-0.00460	0.01102	-0.01733	0.01248
279	Cu1	DXZ	-0.00000	-0.00000	-0.00000	-0.00000	-0.00000
280	Cu1	DYZ	-0.00822	0.01445	-0.00533	0.01002	0.00019
281	Cu1	DZ**2	0.00000	-0.00000	0.00000	-0.00000	0.00000
282	Cu1	DXX-YY	0.00000	0.00000	0.00000	0.00000	0.00000
283	Cu1	DXY	0.00212	-0.00309	0.00331	-0.00586	0.00322
284	Cu1	DXZ	-0.00000	-0.00000	-0.00000	-0.00000	0.00000
285	Cu1	DYZ	-0.00987	0.00405	-0.00433	0.00100	0.00357
286	Cu1	DZ**2	0.00000	0.00000	0.00000	0.00000	-0.00000
287	Cu1	DXX-YY	-0.00000	-0.00000	-0.00000	0.00000	-0.00000
288	Cu1	DXY	-0.00340	0.00390	-0.00696	0.01766	-0.03120
289	Cu1	DXZ	0.00000	-0.00000	0.00000	0.00000	0.00000
290	Cu1	DYZ	0.05047	-0.01713	0.02198	0.00814	-0.02506
291	O2	S1	-0.00000	-0.00000	0.00000	-0.00000	-0.00000
292	O2	S	-0.00000	-0.00000	0.00000	-0.00000	-0.00000
293	O2	PX	-0.00000	0.00000	-0.00000	0.00000	-0.00000
294	O2	PY	0.11600	-0.01084	0.04411	-0.01461	-0.06192
295	O2	PZ	-0.00000	-0.00000	-0.00000	-0.00000	0.00000
296	O2	S'	0.00000	0.00000	-0.00000	0.00000	0.00000
297	O2	PX'	-0.00000	0.00000	-0.00000	0.00000	0.00000
298	O2	PY'	0.16066	-0.01577	0.06044	-0.01984	-0.08328
299	O2	PZ'	-0.00000	-0.00000	-0.00000	-0.00000	0.00000
300	O2	S″	0.00000	0.00000	-0.00000	0.00000	-0.00000
301	O2	PX″	-0.00000	0.00000	-0.00000	-0.00000	0.00000
302	O2	PY″	0.22178	-0.01777	0.09421	-0.03056	-0.14283
303	O2	PZ″	-0.00000	0.00000	-0.00000	0.00000	-0.00000
304	O2	DZ**2	0.00000	-0.00000	0.00000	-0.00000	-0.00000
305	O2	DXX-YY	-0.00000	-0.00000	-0.00000	0.00000	0.00000
306	O2	DXY	0.00216	0.00011	0.00282	0.00014	-0.00494
307	O2	DXZ	0.00000	0.00000	-0.00000	0.00000	0.00000
308	O2	DYZ	-0.00232	0.00001	-0.00102	-0.00019	0.00268
309	N3	S1	0.00000	0.00000	-0.00000	0.00000	-0.00000
310	N3	S	0.00000	0.00000	-0.00000	0.00000	-0.00000
311	N3	PX	-0.00000	-0.00000	-0.00000	-0.00000	-0.00000
312	N3	PY	0.04363	-0.00323	0.12307	-0.02683	0.08313
313	N3	PZ	0.00000	0.00000	0.00000	-0.00000	-0.00000
314	N3	S'	-0.00000	-0.00000	0.00000	-0.00000	0.00000
315	N3	PX'	-0.00000	-0.00000	-0.00000	-0.00000	-0.00000
316	N3	PY'	0.06234	-0.00502	0.17668	-0.03951	0.11918
317	N3	PZ'	0.00000	0.00000	0.00000	-0.00000	-0.00000
318	N3	S″	0.00000	-0.00000	0.00000	-0.00000	-0.00000
319	N3	PX″	-0.00000	-0.00000	-0.00000	-0.00000	0.00000
320	N3	PY″	0.09600	-0.00454	0.28074	-0.05669	0.19656
321	N3	PZ″	0.00000	0.00000	0.00000	-0.00000	-0.00000
322	N3	DZ**2	0.00000	-0.00000	-0.00000	0.00000	0.00000
323	N3	DXX-YY	0.00000	0.00000	0.00000	0.00000	-0.00000
324	N3	DXY	0.00230	0.00018	0.00271	0.00019	0.00313
325	N3	DXZ	-0.00000	-0.00000	0.00000	-0.00000	0.00000
326	N3	DYZ	0.00707	-0.00075	-0.00035	-0.00011	-0.00699
327	C13	S1	-0.00000	-0.00000	-0.00000	0.00000	0.00000
328	C13	S	-0.00000	-0.00000	-0.00000	0.00000	0.00000
329	C13	PX	0.00000	0.00000	0.00000	-0.00000	0.00000
330	C13	PY	-0.06056	0.00210	-0.09266	0.01654	-0.05149
331	C13	PZ	-0.00000	-0.00000	-0.00000	0.00000	-0.00000
332	C13	S'	0.00000	0.00000	0.00000	-0.00000	-0.00000
333	C13	PX'	0.00000	0.00000	0.00000	-0.00000	0.00000
334	C13	PY'	-0.09581	0.00477	-0.14619	0.02579	-0.07752
335	C13	PZ'	-0.00000	0.00000	-0.00000	0.00000	-0.00000
336	C13	S″	-0.00000	0.00000	-0.00000	-0.00000	0.00000
337	C13	PX″	0.00000	0.00000	0.00000	-0.00000	0.00000
338	C13	PY″	-0.12020	-0.00268	-0.21524	0.03694	-0.15495
339	C13	PZ″	-0.00000	-0.00000	-0.00000	-0.00000	0.00000
340	C13	DZ**2	0.00000	0.00000	0.00000	0.00000	0.00000
341	C13	DXX-YY	-0.00000	0.00000	-0.00000	0.00000	0.00000
342	C13	DXY	0.00845	-0.00035	0.02381	-0.00539	0.01555
343	C13	DXZ	0.00000	0.00000	0.00000	0.00000	0.00000
344	C13	DYZ	-0.00144	0.00011	0.00010	-0.00007	0.00064
345	C14	S1	0.00000	0.00000	0.00000	0.00000	-0.00000
346	C14	S	0.00000	0.00000	0.00000	0.00000	-0.00000
347	C14	PX	0.00000	-0.00000	0.00000	-0.00000	-0.00000
348	C14	PY	-0.04082	0.00324	-0.06155	0.01485	-0.01667

349	C14	PZ	-0.00000	0.00000	-0.00000	0.00000	0.00000
350	C14	S'	-0.00000	-0.00000	-0.00000	-0.00000	0.00000
351	C14	PX'	0.00000	-0.00000	0.00000	-0.00000	-0.00000
352	C14	PY'	-0.06313	0.00486	-0.08984	0.02173	-0.02343
353	C14	PZ'	-0.00000	0.00000	-0.00000	0.00000	0.00000
354	C14	S"	0.00000	0.00000	0.00000	0.00000	0.00000
355	C14	PX"	0.00000	0.00000	0.00000	0.00000	-0.00000
356	C14	PY"	-0.08674	0.00929	-0.18775	0.04543	-0.05966
357	C14	PZ"	0.00000	0.00000	0.00000	0.00000	-0.00000
358	C14	DZ**2	0.00000	0.00000	0.00000	-0.00000	0.00000
359	C14	DXX-YY	0.00000	0.00000	0.00000	-0.00000	0.00000
360	C14	DXY	-0.00052	0.00003	-0.00012	-0.00025	-0.00207
361	C14	DXZ	-0.00000	-0.00000	-0.00000	0.00000	0.00000
362	C14	DYZ	0.01265	-0.00061	0.02721	-0.00557	0.01687
363	C15	S1	0.00000	0.00000	-0.00000	0.00000	-0.00000
364	C15	S	0.00000	0.00000	-0.00000	0.00000	-0.00000
365	C15	PX	-0.00000	-0.00000	-0.00000	0.00000	0.00000
366	C15	PY	0.06480	-0.00286	0.14100	-0.02957	0.07438
367	C15	PZ	0.00000	-0.00000	0.00000	-0.00000	-0.00000
368	C15	S'	-0.00000	-0.00000	0.00000	-0.00000	0.00000
369	C15	PX'	-0.00000	-0.00000	-0.00000	0.00000	0.00000
370	C15	PY'	0.10401	-0.00481	0.21344	-0.04504	0.10833
371	C15	PZ'	0.00000	-0.00000	0.00000	-0.00000	-0.00000
372	C15	S"	-0.00000	-0.00000	-0.00000	-0.00000	0.00000
373	C15	PX"	-0.00000	-0.00000	-0.00000	-0.00000	-0.00000
374	C15	PY"	0.13080	-0.00610	0.38410	-0.08031	0.21429
375	C15	PZ"	-0.00000	0.00000	0.00000	-0.00000	0.00000
376	C15	DZ**2	-0.00000	0.00000	0.00000	-0.00000	0.00000
377	C15	DXX-YY	-0.00000	0.00000	0.00000	-0.00000	0.00000
378	C15	DXY	0.00672	-0.00059	-0.00498	0.00093	-0.01680
379	C15	DXZ	0.00000	-0.00000	0.00000	-0.00000	-0.00000
380	C15	DYZ	0.00153	-0.00004	0.00839	-0.00195	0.00784
381	N4	S1	0.00000	-0.00000	0.00000	-0.00000	-0.00000
382	N4	S	0.00000	-0.00000	0.00000	-0.00000	-0.00000
383	N4	PX	-0.00000	0.00000	0.00000	-0.00000	0.00000
384	N4	PY	0.02353	-0.00261	-0.09576	0.01971	-0.11744
385	N4	PZ	0.00000	-0.00000	-0.00000	-0.00000	-0.00000
386	N4	S'	-0.00000	0.00000	-0.00000	0.00000	0.00000
387	N4	PX'	-0.00000	0.00000	0.00000	-0.00000	0.00000
388	N4	PY'	0.03998	-0.00463	-0.13605	0.02800	-0.17474
389	N4	PZ'	-0.00000	-0.00000	-0.00000	0.00000	-0.00000
390	N4	S"	-0.00000	0.00000	-0.00000	0.00000	0.00000
391	N4	PX"	-0.00000	-0.00000	0.00000	-0.00000	0.00000
392	N4	PY"	0.02645	-0.00341	-0.23780	0.04830	-0.26513
393	N4	PZ"	0.00000	-0.00000	-0.00000	-0.00000	-0.00000
394	N4	DZ**2	0.00000	0.00000	0.00000	-0.00000	-0.00000
395	N4	DXX-YY	0.00000	-0.00000	0.00000	-0.00000	0.00000
396	N4	DXY	-0.01339	0.00080	-0.01695	0.00358	0.00390
397	N4	DXZ	0.00000	0.00000	-0.00000	0.00000	0.00000
398	N4	DYZ	-0.01159	0.00099	-0.01087	0.00249	0.00266
399	C17	S1	0.00000	-0.00000	0.00000	-0.00000	0.00000
400	C17	S	0.00000	-0.00000	0.00000	-0.00000	0.00000
401	C17	PX	0.00000	-0.00000	0.00000	-0.00000	-0.00000
402	C17	PY	-0.12570	0.00885	-0.06946	0.01318	0.10975
403	C17	PZ	0.00000	0.00000	0.00000	-0.00000	-0.00000
404	C17	S'	-0.00000	0.00000	-0.00000	0.00000	0.00000
405	C17	PX'	0.00000	-0.00000	0.00000	-0.00000	-0.00000
406	C17	PY'	-0.20825	0.01307	-0.11658	0.02162	0.18805
407	C17	PZ'	0.00000	-0.00000	0.00000	-0.00000	-0.00000
408	C17	S"	-0.00000	0.00000	-0.00000	-0.00000	-0.00000
409	C17	PX"	0.00000	-0.00000	0.00000	-0.00000	0.00000
410	C17	PY"	-0.21162	0.01873	-0.13267	0.02893	0.20928
411	C17	PZ"	-0.00000	0.00000	0.00000	0.00000	-0.00000
412	C17	DZ**2	0.00000	0.00000	0.00000	0.00000	0.00000
413	C17	DXX-YY	-0.00000	-0.00000	-0.00000	0.00000	0.00000
414	C17	DXY	0.02226	-0.00222	0.01844	-0.00500	-0.00140
415	C17	DXZ	-0.00000	0.00000	0.00000	0.00000	-0.00000
416	C17	DYZ	-0.02146	0.00232	0.01855	-0.00287	0.01162
417	C18	S1	-0.00000	-0.00000	0.00000	-0.00000	-0.00000
418	C18	S	-0.00000	-0.00000	0.00000	-0.00000	-0.00000
419	C18	PX	-0.00000	0.00000	0.00000	-0.00000	-0.00000
420	C18	PY	-0.08523	0.00873	0.10034	-0.01634	0.00638
421	C18	PZ	0.00000	-0.00000	-0.00000	0.00000	0.00000
422	C18	S'	-0.00000	0.00000	-0.00000	0.00000	0.00000
423	C18	PX'	-0.00000	0.00000	0.00000	-0.00000	-0.00000
424	C18	PY'	-0.12786	0.01337	0.15246	-0.02518	-0.00065
425	C18	PZ'	0.00000	-0.00000	-0.00000	0.00000	0.00000
426	C18	S"	0.00000	-0.00000	0.00000	-0.00000	0.00000
427	C18	PX"	0.00000	0.00000	0.00000	0.00000	-0.00000
428	C18	PY"	-0.22657	0.02099	0.24641	-0.04027	0.12757
429	C18	PZ"	0.00000	0.00000	-0.00000	0.00000	-0.00000
430	C18	DZ**2	-0.00000	0.00000	-0.00000	0.00000	0.00000
431	C18	DXX-YY	-0.00000	-0.00000	-0.00000	-0.00000	-0.00000
432	C18	DXY	0.01684	-0.00148	-0.00267	-0.00004	-0.01483
433	C18	DXZ	-0.00000	0.00000	-0.00000	0.00000	0.00000
434	C18	DYZ	0.02786	-0.00219	0.00238	-0.00080	-0.02024
435	C19	S1	0.00000	-0.00000	0.00000	0.00000	0.00000
436	C19	S	0.00000	-0.00000	0.00000	0.00000	0.00000
437	C19	PX	0.00000	-0.00000	0.00000	-0.00000	-0.00000
438	C19	PY	0.14173	-0.01203	-0.03431	0.00354	-0.11129
439	C19	PZ	-0.00000	0.00000	0.00000	-0.00000	0.00000
440	C19	S'	0.00000	0.00000	0.00000	0.00000	-0.00000
441	C19	PX'	0.00000	-0.00000	0.00000	0.00000	-0.00000
442	C19	PY'	0.20856	-0.01796	-0.05601	0.00540	-0.14919
443	C19	PZ'	-0.00000	0.00000	0.00000	-0.00000	0.00000
444	C19	S"	-0.00000	0.00000	-0.00000	-0.00000	0.00000
445	C19	PX"	0.00000	-0.00000	0.00000	-0.00000	-0.00000
446	C19	PY"	0.33835	-0.02790	-0.06916	0.00796	-0.44819
447	C19	PZ"	-0.00000	0.00000	0.00000	0.00000	0.00000
448	C19	DZ**2	-0.00000	0.00000	0.00000	-0.00000	-0.00000
449	C19	DXX-YY	-0.00000	0.00000	0.00000	-0.00000	0.00000
450	C19	DXY	0.00665	-0.00076	-0.01096	0.00183	-0.00440
451	C19	DXZ	-0.00000	0.00000	-0.00000	0.00000	-0.00000
452	C19	DYZ	0.01202	-0.00131	-0.01962	0.00333	0.02087
453	C20	S1	0.00000	0.00000	0.00000	-0.00000	-0.00000
454	C20	S	0.00000	0.00000	0.00000	-0.00000	-0.00000
455	C20	PX	0.00000	-0.00000	-0.00000	0.00000	-0.00000
456	C20	PY	0.03510	-0.00413	-0.08754	0.01522	0.16592
457	C20	PZ	-0.00000	0.00000	0.00000	-0.00000	0.00000
458	C20	S'	-0.00000	-0.00000	-0.00000	-0.00000	0.00000
459	C20	PX'	0.00000	-0.00000	0.00000	0.00000	-0.00000
460	C20	PY'	0.05448	-0.00632	-0.12739	0.02208	0.22980
461	C20	PZ'	-0.00000	0.00000	0.00000	-0.00000	0.00000
462	C20	S"	0.00000	0.00000	-0.00000	0.00000	0.00000
463	C20	PX"	-0.00000	0.00000	-0.00000	0.00000	-0.00000
464	C20	PY"	0.07580	-0.00989	-0.26350	0.04605	0.63730
465	C20	PZ"	-0.00000	0.00000	0.00000	-0.00000	0.00000
466	C20	DZ**2	0.00000	-0.00000	0.00000	0.00000	0.00000
467	C20	DXX-YY	-0.00000	0.00000	0.00000	-0.00000	-0.00000
468	C20	DXY	-0.01418	0.00128	0.01158	-0.00190	-0.01775
469	C20	DXZ	-0.00000	0.00000	-0.00000	0.00000	0.00000
470	C20	DYZ	-0.02617	0.00234	0.01299	-0.00182	0.00603
471	C21	S1	-0.00000	0.00000	0.00000	0.00000	0.00000
472	C21	S	-0.00000	0.00000	0.00000	0.00000	0.00000
473	C21	PX	-0.00000	0.00000	0.00000	-0.00000	-0.00000
474	C21	PY	-0.13001	0.01182	0.10604	-0.01727	-0.11997
475	C21	PZ	0.00000	-0.00000	-0.00000	0.00000	0.00000
476	C21	S'	0.00000	-0.00000	-0.00000	-0.00000	-0.00000
477	C21	PX'	-0.00000	0.00000	0.00000	-0.00000	-0.00000
478	C21	PY'	-0.19274	0.01747	0.15335	-0.02497	-0.15868
479	C21	PZ'	0.00000	-0.00000	-0.00000	0.00000	0.00000
480	C21	S"	0.00000	-0.00000	-0.00000	-0.00000	-0.00000
481	C21	PX"	-0.00000	0.00000	0.00000	-0.00000	-0.00000
482	C21	PY"	-0.28820	0.02729	0.31087	-0.05167	-0.51043
483	C21	PZ"	0.00000	-0.00000	-0.00000	0.00000	-0.00000
484	C21	DZ**2	0.00000	-0.00000	-0.00000	0.00000	-0.00000
485	C21	DXX-YY	-0.00000	0.00000	0.00000	-0.00000	0.00000
486	C21	DXY	-0.00283	0.00037	0.01074	-0.00192	-0.02458
487	C21	DXZ	-0.00000	0.00000	0.00000	-0.00000	-0.00000
488	C21	DYZ	-0.00371	0.00041	0.00734	-0.00127	-0.01083
489	C22	S1	0.00000	-0.00000	0.00000	-0.00000	-0.00000
490	C22	S	0.00000	-0.00000	0.00000	-0.00000	-0.00000
491	C22	PX	0.00000	0.00000	0.00000	-0.00000	-0.00000
492	C22	PY	0.01420	-0.00118	-0.00403	0.00050	-0.00296

493	C22	PZ	0.00000	0.00000	-0.00000	-0.00000	0.00000
494	C22	S’	-0.00000	0.00000	-0.00000	0.00000	0.00000
495	C22	PX’	0.00000	-0.00000	0.00000	-0.00000	-0.00000
496	C22	PY’	0.01764	-0.00144	-0.00328	0.00031	-0.00892
497	C22	PZ’	0.00000	-0.00000	-0.00000	-0.00000	0.00000
498	C22	S″	-0.00000	0.00000	0.00000	-0.00000	0.00000
499	C22	PX″	-0.00000	0.00000	0.00000	-0.00000	-0.00000
500	C22	PY″	0.00772	-0.00071	-0.01204	0.00189	0.08581
501	C22	PZ″	0.00000	-0.00000	-0.00000	0.00000	0.00000
502	C22	DZ**2	-0.00000	0.00000	0.00000	-0.00000	0.00000
503	C22	DXX-YY	-0.00000	0.00000	0.00000	-0.00000	-0.00000
504	C22	DXY	0.00644	-0.00058	-0.00557	0.00092	0.00804
505	C22	DXZ	0.00000	-0.00000	-0.00000	0.00000	0.00000
506	C22	DYZ	0.01882	-0.00174	-0.01742	0.00289	0.02301
507	C23	S1	-0.00000	0.00000	-0.00000	0.00000	-0.00000
508	C23	S	-0.00000	0.00000	-0.00000	0.00000	-0.00000
509	C23	PX	-0.00000	-0.00000	-0.00000	-0.00000	-0.00000
510	C23	PY	0.01062	-0.00140	0.01231	-0.00266	0.00377
511	C23	PZ	0.00000	0.00000	0.00000	0.00000	0.00000
512	C23	S’	0.00000	0.00000	0.00000	-0.00000	0.00000
513	C23	PX’	-0.00000	0.00000	-0.00000	-0.00000	-0.00000
514	C23	PY’	0.01360	-0.00178	0.01419	-0.00342	0.00323
515	C23	PZ’	0.00000	0.00000	0.00000	0.00000	-0.00000
516	C23	S″	-0.00000	-0.00000	-0.00000	-0.00000	0.00000
517	C23	PX″	-0.00000	-0.00000	-0.00000	-0.00000	-0.00000
518	C23	PY″	0.02330	0.00749	0.04654	-0.00201	0.03396
519	C23	PZ″	-0.00000	0.00000	0.00000	0.00000	0.00000
520	C23	DZ**2	-0.00000	0.00000	-0.00000	0.00000	-0.00000
521	C23	DXX-YY	0.00000	0.00000	0.00000	0.00000	0.00000
522	C23	DXY	-0.00327	-0.00003	-0.00452	0.00047	-0.00267
523	C23	DXZ	0.00000	-0.00000	0.00000	-0.00000	0.00000
524	C23	DYZ	-0.00810	0.00021	-0.01440	0.00251	-0.00891
525	C24	S1	0.00000	-0.00000	0.00000	-0.00000	-0.00000
526	C24	S	0.00000	-0.00000	0.00000	-0.00000	-0.00000
527	C24	PX	-0.00000	0.00000	0.00000	0.00000	-0.00000
528	C24	PY	0.00526	-0.00079	-0.00403	-0.00014	0.00417
529	C24	PZ	-0.00000	-0.00000	0.00000	-0.00000	0.00000
530	C24	S’	-0.00000	0.00000	-0.00000	0.00000	0.00000
531	C24	PX’	-0.00000	0.00000	0.00000	0.00000	-0.00000
532	C24	PY’	0.00757	-0.00102	-0.00302	-0.00054	0.00930
533	C24	PZ’	-0.00000	-0.00000	0.00000	-0.00000	0.00000
534	C24	S″	0.00000	0.00000	-0.00000	0.00000	0.00001
535	C24	PX″	0.00000	-0.00000	-0.00000	0.00000	-0.00000
536	C24	PY″	0.03566	-0.00356	0.01447	-0.00810	0.06844
537	C24	PZ″	-0.00000	-0.00000	0.00000	-0.00000	0.00000
538	C24	DZ**2	-0.00000	0.00000	-0.00000	0.00000	-0.00000
539	C24	DXX-YY	-0.00000	-0.00000	-0.00000	-0.00000	0.00000
540	C24	DXY	0.00127	-0.00003	0.00429	-0.00090	0.00263
541	C24	DXZ	0.00000	-0.00000	0.00000	-0.00000	-0.00000
542	C24	DYZ	-0.01007	0.00041	-0.02549	0.00545	-0.01475
543	O3	S1	0.00265	0.00160	0.00374	0.00276	0.00060
544	O3	S	0.00431	0.00262	0.00615	0.00455	0.00102
545	O3	PX	0.00151	-0.00091	0.00519	-0.00789	0.00740
546	O3	PY	0.00494	0.00806	0.00774	0.00616	0.00339
547	O3	PZ	-0.00225	0.00719	0.00062	0.00581	0.00081
548	O3	S’	-0.01271	-0.00790	-0.01943	-0.01459	-0.00380
549	O3	PX’	0.00175	-0.00135	0.00674	-0.01098	0.00972
550	O3	PY’	0.00665	0.01025	0.00956	0.00715	0.00350
551	O3	PZ’	-0.00309	0.00923	0.00158	0.00889	0.00205
552	O3	S″	-0.03220	-0.01963	-0.04010	-0.02996	-0.00201
553	O3	PX″	0.00532	0.00128	0.01157	-0.01135	0.01499
554	O3	PY″	0.00738	0.01856	0.01658	0.01620	0.01262
555	O3	PZ″	0.00045	0.02148	0.00516	0.01172	-0.00098
556	O3	DZ**2	0.00011	-0.00069	-0.00066	-0.00082	-0.00046
557	O3	DXX-YY	-0.00012	-0.00015	-0.00012	0.00011	-0.00016
558	O3	DXY	0.00010	-0.00035	0.00064	-0.00124	0.00104
559	O3	DXZ	-0.00005	-0.00016	-0.00061	0.00012	-0.00095
560	O3	DYZ	-0.00064	0.00040	-0.00057	0.00045	-0.00006
561	S1	S1	0.00032	-0.00017	0.00040	0.00017	-0.00007
562	S1	S2	0.00094	-0.00046	0.00118	0.00051	-0.00020
563	S1	S	-0.00224	0.00078	-0.00290	-0.00139	0.00034
564	S1	S’	-0.00306	0.00294	-0.00352	-0.00084	0.00124
565	S1	S″	0.00278	-0.01194	0.00213	-0.00412	-0.00352
566	S1	S	0.02908	0.00870	0.03754	0.02959	-0.00684
567	S1	PX2	-0.00031	0.00094	-0.00080	0.00075	-0.00085
568	S1	PY2	0.00107	0.00063	0.00059	0.00076	-0.00062
569	S1	PZ2	-0.00122	-0.00084	-0.00162	-0.00118	-0.00015
570	S1	PX	-0.00058	0.00172	-0.00147	0.00137	-0.00152
571	S1	PY	0.00193	0.00098	0.00109	0.00129	-0.00110
572	S1	PZ	-0.00228	-0.00159	-0.00298	-0.00218	-0.00025
573	S1	PX’	0.00091	-0.00227	0.00205	-0.00174	0.00192
574	S1	PY’	-0.00251	-0.00023	-0.00163	-0.00127	0.00130
575	S1	PZ’	0.00355	0.00260	0.00438	0.00335	0.00039
576	S1	PX″	0.00166	-0.01021	0.00757	-0.00843	0.00918
577	S1	PY″	-0.00971	-0.00896	-0.00362	-0.00688	0.00844
578	S1	PZ″	0.00881	0.00707	0.01135	0.00705	-0.00105
579	S1	PX	0.00149	-0.01367	-0.00326	-0.00845	-0.00004
580	S1	PY	-0.02580	-0.05154	-0.01890	-0.02409	-0.00098
581	S1	PZ	-0.01372	-0.03551	-0.00095	-0.01138	0.00695
582	S1	DZ**2	0.00127	0.00298	0.00224	0.00405	-0.00021
583	S1	DXX-YY	0.00006	-0.00061	0.00294	0.00058	0.00310
584	S1	DXY	-0.00104	-0.00222	-0.00159	0.00075	-0.00173
585	S1	DXZ	0.00104	-0.00081	0.00257	-0.00368	0.00336
586	S1	DYZ	-0.00174	0.00166	-0.00019	0.00134	0.00119
587	O5	S1	0.00007	-0.00107	0.00026	-0.00021	-0.00001
588	O5	S	0.00009	-0.00175	0.00043	-0.00034	0.00001
589	O5	PX	0.00003	0.00188	-0.00040	0.00064	-0.00045
590	O5	PY	0.00243	0.00069	0.00241	0.00140	-0.00015
591	O5	PZ	-0.00246	-0.00152	-0.00340	0.00082	-0.00181
592	O5	S’	0.00026	0.00568	-0.00131	0.00119	-0.00052
593	O5	PX’	-0.00023	0.00273	-0.00097	0.00047	-0.00058
594	O5	PY’	0.00365	0.00193	0.00336	0.00253	-0.00029
595	O5	PZ’	-0.00405	-0.00332	-0.00504	0.00122	-0.00252
596	O5	S″	-0.00689	0.00277	-0.00572	-0.00139	0.00178
597	O5	PX″	0.00181	0.00267	0.00206	0.00377	-0.00064
598	O5	PY″	0.00507	0.00269	0.00530	0.00296	-0.00036
599	O5	PZ″	-0.00321	0.00455	-0.00717	0.00126	-0.00397
600	O5	DZ**2	-0.00004	-0.00024	0.00003	0.00012	0.00005
601	O5	DXX-YY	0.00001	0.00011	0.00001	0.00006	0.00003
602	O5	DXY	-0.00007	0.00022	-0.00003	0.00015	0.00002
603	O5	DXZ	0.00015	0.00036	0.00023	0.00032	0.00005
604	O5	DYZ	0.00003	-0.00052	-0.00001	-0.00023	-0.00006
605	O4	S1	0.00014	0.00079	0.00025	0.00058	-0.00005
606	O4	S	0.00022	0.00126	0.00042	0.00093	-0.00007
607	O4	PX	-0.00115	-0.00102	-0.00011	-0.00172	0.00187
608	O4	PY	0.00297	0.00761	0.00335	0.00372	0.00094
609	O4	PZ	-0.00005	0.00079	-0.00046	-0.00291	0.00139
610	O4	S’	-0.00021	-0.00302	-0.00103	-0.00239	0.00005
611	O4	PX’	-0.00159	-0.00184	-0.00041	-0.00266	0.00245
612	O4	PY’	0.00396	0.01025	0.00508	0.00517	0.00149
613	O4	PZ’	-0.00043	0.00047	-0.00098	-0.00456	0.00219
614	O4	S″	-0.00573	-0.01779	-0.00621	-0.01417	0.00357
615	O4	PX″	-0.00370	-0.00483	-0.00087	-0.00660	0.00527
616	O4	PY″	0.00761	0.01791	0.00635	0.00829	0.00119
617	O4	PZ″	0.00067	0.00313	-0.00080	-0.00457	0.00182
618	O4	DZ**2	0.00013	0.00019	0.00013	0.00019	-0.00003
619	O4	DXX-YY	-0.00010	-0.00034	-0.00014	-0.00019	-0.00008
620	O4	DXY	0.00016	0.00053	0.00018	0.00013	0.00014
621	O4	DXZ	0.00008	0.00002	0.00006	-0.00010	0.00004
622	O4	DYZ	0.00010	0.00034	0.00011	0.00008	0.00009
623	C26	S1	0.00078	0.00032	0.00142	0.00065	0.00052
624	C26	S	0.00135	0.00077	0.00240	0.00117	0.00088
625	C26	PX	0.00049	-0.00040	0.00118	-0.00003	0.00048
626	C26	PY	0.00203	0.00013	0.00333	0.00124	0.00079
627	C26	PZ	0.00161	0.00090	0.00339	0.00227	0.00120
628	C26	S’	-0.00619	-0.00938	-0.00864	-0.00628	-0.00292
629	C26	PX’	0.00121	-0.00004	0.00193	0.00037	0.00061
630	C26	PY’	0.00383	0.00314	0.00592	0.00319	0.00135
631	C26	PZ’	0.00351	0.00335	0.00674	0.00457	0.00253
632	C26	S″	0.01046	0.05598	-0.00624	0.01494	-0.00647
633	C26	PX″	-0.00170	-0.00527	0.00534	-0.00193	0.00173
634	C26	PY″	0.00401	-0.00886	0.00662	-0.00110	0.00025
635	C26	PZ″	-0.00090	0.00060	0.00579	0.00842	0.00237
636	C26	DZ**2	-0.00026	-0.00053	-0.00070	-0.00075	-0.00020

637	C26	DXX-YY	−0.00033	−0.00001	−0.00068	−0.00032	−0.00041
638	C26	DXY	−0.00006	0.00023	−0.00020	0.00032	−0.00050
639	C26	DXZ	−0.00005	0.00027	−0.00008	0.00020	−0.00024
640	C26	DYZ	−0.00065	−0.00087	−0.00095	−0.00076	−0.00035
641	F1	S1	−0.00002	−0.00010	0.00007	0.00000	−0.00005
642	F1	S	−0.00002	−0.00017	0.00012	0.00000	−0.00008
643	F1	PX	−0.00024	0.00016	0.00001	−0.00001	0.00038
644	F1	PY	−0.00012	0.00003	0.00014	0.00015	−0.00007
645	F1	PZ	−0.00066	−0.00111	−0.00105	−0.00143	−0.00009
646	F1	S'	−0.00018	0.00085	−0.00053	0.00008	0.00024
647	F1	PX'	−0.00050	−0.00030	−0.00010	−0.00040	0.00078
648	F1	PY'	0.00018	0.00046	0.00077	0.00046	−0.00010
649	F1	PZ'	−0.00098	−0.00146	−0.00141	−0.00191	0.00013
650	F1	S″	0.00151	−0.00242	−0.00071	−0.00119	0.00025
651	F1	PX″	0.00038	0.00461	0.00078	0.00247	−0.00042
652	F1	PY″	−0.00171	−0.00134	−0.00201	−0.00073	−0.00011
653	F1	PZ″	−0.00075	−0.00142	−0.00188	−0.00263	−0.00105
654	F1	DZ**2	−0.00004	−0.00001	−0.00009	−0.00006	−0.00003
655	F1	DXX-YY	−0.00002	−0.00008	−0.00004	−0.00004	−0.00000
656	F1	DXY	−0.00005	0.00000	−0.00009	−0.00005	0.00002
657	F1	DXZ	−0.00001	−0.00004	−0.00004	−0.00004	−0.00002
658	F1	DYZ	−0.00002	−0.00002	−0.00003	−0.00005	0.00002
659	F3	S1	−0.00010	0.00014	−0.00007	0.00022	−0.00002
660	F3	S	−0.00016	0.00022	−0.00011	0.00036	−0.00003
661	F3	PX	−0.00020	−0.00048	−0.00049	−0.00068	−0.00010
662	F3	PY	−0.00062	0.00091	−0.00042	0.00088	0.00006
663	F3	PZ	−0.00055	0.00084	−0.00114	0.00029	−0.00061
664	F3	S'	0.00049	−0.00017	0.00015	−0.00101	0.00006
665	F3	PX'	−0.00013	−0.00020	−0.00050	−0.00070	−0.00009
666	F3	PY'	−0.00067	0.00188	−0.00040	0.00174	0.00017
667	F3	PZ'	−0.00075	0.00103	−0.00146	0.00044	−0.00092
668	F3	S″	0.00090	−0.00773	0.00210	−0.00527	0.00049
669	F3	PX″	−0.00098	−0.00447	−0.00150	−0.00312	−0.00031
670	F3	PY″	−0.00204	−0.00039	−0.00168	−0.00020	−0.00007
671	F3	PZ″	−0.00054	0.00433	−0.00268	0.00138	−0.00095
672	F3	DZ**2	−0.00004	−0.00005	−0.00009	−0.00007	−0.00005
673	F3	DXX-YY	−0.00000	−0.00006	0.00000	−0.00005	0.00000
674	F3	DXY	0.00002	0.00001	0.00006	0.00003	0.00001
675	F3	DXZ	−0.00001	0.00007	−0.00003	0.00001	−0.00001
676	F3	DYZ	−0.00002	0.00002	−0.00006	−0.00001	−0.00003
677	F2	S1	−0.00018	−0.00018	−0.00038	−0.00027	−0.00019
678	F2	S	−0.00031	−0.00031	−0.00064	−0.00045	−0.00031
679	F2	PX	−0.00020	0.00054	−0.00034	0.00029	−0.00003
680	F2	PY	−0.00156	−0.00098	−0.00293	−0.00185	−0.00110
681	F2	PZ	−0.00047	−0.00053	−0.00061	−0.00058	−0.00004
682	F2	S'	0.00134	0.00142	0.00216	0.00148	0.00093
683	F2	PX'	−0.00022	0.00079	−0.00030	0.00035	0.00004
684	F2	PY'	−0.00217	−0.00145	−0.00401	−0.00259	−0.00147
685	F2	PZ'	−0.00035	−0.00018	−0.00062	−0.00052	−0.00004
686	F2	S″	−0.00095	−0.00183	0.00388	0.00317	0.00302
687	F2	PX″	−0.00062	0.00058	−0.00143	0.00064	−0.00024
688	F2	PY″	−0.00160	0.00088	−0.00520	−0.00284	−0.00241
689	F2	PZ″	−0.00253	−0.00444	−0.00212	−0.00255	0.00005
690	F2	DZ**2	0.00004	−0.00000	0.00010	0.00005	0.00003
691	F2	DXX-YY	0.00005	0.00006	0.00009	0.00006	0.00003
692	F2	DXY	−0.00000	0.00004	−0.00001	0.00003	−0.00001
693	F2	DXZ	0.00001	0.00001	0.00002	0.00002	0.00001
694	F2	DYZ	0.00001	0.00006	−0.00001	0.00000	0.00000
695	O6	S1	−0.00265	−0.00160	−0.00374	−0.00276	−0.00060
696	O6	S	−0.00431	−0.00262	−0.00615	−0.00455	−0.00102
697	O6	PX	−0.00151	0.00091	−0.00519	0.00789	−0.00740
698	O6	PY	0.00494	0.00806	0.00774	0.00616	0.00339
699	O6	PZ	0.00225	−0.00719	−0.00062	−0.00581	−0.00081
700	O6	S'	0.01271	0.00790	0.01943	0.01459	0.00380
701	O6	PX'	−0.00175	0.00135	−0.00674	0.01098	−0.00972
702	O6	PY'	0.00665	0.01025	0.00956	0.00715	0.00350
703	O6	PZ'	0.00309	−0.00923	−0.00158	−0.00889	−0.00205
704	O6	S″	0.03220	0.01963	0.04010	0.02996	0.00201
705	O6	PX″	−0.00532	−0.00128	−0.01157	0.01135	−0.01499
706	O6	PY″	0.00738	0.01856	0.01658	0.01620	0.01262
707	O6	PZ″	−0.00045	−0.02148	−0.00516	−0.01172	0.00098
708	O6	DZ**2	−0.00011	0.00069	0.00066	0.00082	0.00046
709	O6	DXX-YY	0.00012	0.00015	0.00012	−0.00011	0.00016
710	O6	DXY	0.00010	−0.00035	0.00064	−0.00124	0.00104
711	O6	DXZ	0.00005	0.00016	0.00061	−0.00012	0.00095
712	O6	DYZ	−0.00064	0.00040	−0.00057	0.00045	−0.00006
713	S2	S1	−0.00032	0.00017	−0.00040	−0.00017	0.00007
714	S2	S2	−0.00094	0.00046	−0.00118	−0.00051	0.00020
715	S2	S	0.00224	−0.00078	0.00290	0.00139	−0.00034
716	S2	S'	0.00306	−0.00294	0.00352	0.00084	−0.00124
717	S2	S″	−0.00278	0.01194	−0.00213	0.00412	0.00352
718	S2	S	−0.02908	−0.00870	−0.03754	−0.02959	0.00684
719	S2	PX2	0.00031	−0.00094	0.00080	−0.00075	0.00085
720	S2	PY2	0.00107	0.00063	0.00059	0.00076	−0.00062
721	S2	PZ2	0.00122	0.00084	0.00162	0.00118	0.00015
722	S2	PX	0.00058	−0.00172	0.00147	−0.00137	0.00152
723	S2	PY	0.00193	0.00098	0.00109	0.00129	−0.00110
724	S2	PZ	0.00228	0.00159	0.00298	0.00218	0.00025
725	S2	PX'	−0.00091	0.00227	−0.00205	0.00174	−0.00192
726	S2	PY'	−0.00251	−0.00023	−0.00163	−0.00127	0.00130
727	S2	PZ'	−0.00355	−0.00260	−0.00438	−0.00335	−0.00039
728	S2	PX″	−0.00166	0.01021	−0.00757	0.00843	−0.00918
729	S2	PY″	−0.00971	−0.00896	−0.00362	−0.00688	0.00844
730	S2	PZ″	−0.00881	−0.00707	−0.01135	−0.00705	0.00105
731	S2	PX	−0.00149	0.01367	0.00326	0.00845	0.00004
732	S2	PY	−0.02580	−0.05154	−0.01890	−0.02409	−0.00098
733	S2	PZ	0.01372	0.03551	0.00095	0.01138	−0.00695
734	S2	DZ**2	−0.00127	−0.00298	−0.00224	−0.00405	0.00021
735	S2	DXX-YY	−0.00006	0.00061	−0.00294	−0.00058	−0.00310
736	S2	DXY	−0.00104	−0.00222	−0.00159	0.00075	−0.00173
737	S2	DXZ	−0.00104	0.00081	−0.00257	0.00368	−0.00336
738	S2	DYZ	−0.00174	0.00166	−0.00019	0.00134	0.00119
739	O7	S1	−0.00007	0.00107	−0.00026	0.00021	0.00001
740	O7	S	−0.00009	0.00175	−0.00043	0.00034	−0.00001
741	O7	PX	−0.00003	−0.00188	0.00040	−0.00064	0.00045
742	O7	PY	0.00243	0.00069	0.00241	0.00140	−0.00015
743	O7	PZ	0.00246	0.00152	0.00340	−0.00082	0.00181
744	O7	S'	−0.00026	−0.00568	0.00131	−0.00119	0.00052
745	O7	PX'	0.00023	−0.00273	0.00097	−0.00047	0.00058
746	O7	PY'	0.00365	0.00193	0.00336	0.00253	−0.00029
747	O7	PZ'	0.00405	0.00332	0.00504	−0.00122	0.00252
748	O7	S″	0.00689	−0.00277	0.00572	0.00139	−0.00178
749	O7	PX″	−0.00181	−0.00267	−0.00206	−0.00377	0.00064
750	O7	PY″	0.00507	0.00269	0.00530	0.00296	−0.00036
751	O7	PZ″	0.00321	−0.00455	0.00717	−0.00126	0.00397
752	O7	DZ**2	0.00004	0.00024	−0.00003	−0.00012	−0.00005
753	O7	DXX-YY	−0.00001	−0.00011	−0.00001	−0.00006	−0.00003
754	O7	DXY	−0.00007	0.00022	−0.00003	0.00015	0.00002
755	O7	DXZ	−0.00015	−0.00036	−0.00023	−0.00032	−0.00005
756	O7	DYZ	0.00003	−0.00052	−0.00001	−0.00023	−0.00006
757	O8	S1	−0.00014	−0.00079	−0.00025	−0.00058	0.00005
758	O8	S	−0.00022	−0.00126	−0.00042	−0.00093	0.00007
759	O8	PX	0.00115	0.00102	0.00011	0.00172	−0.00187
760	O8	PY	0.00297	0.00761	0.00335	0.00372	0.00094
761	O8	PZ	0.00005	−0.00079	0.00046	0.00291	−0.00139
762	O8	S'	0.00021	0.00302	0.00103	0.00239	−0.00005
763	O8	PX'	0.00159	0.00184	0.00041	0.00266	−0.00245
764	O8	PY'	0.00396	0.01025	0.00508	0.00517	0.00149
765	O8	PZ'	0.00043	−0.00047	0.00098	0.00456	−0.00219
766	O8	S″	0.00573	0.01779	0.00621	0.01417	−0.00357
767	O8	PX″	0.00370	0.00483	0.00087	0.00660	−0.00527
768	O8	PY″	0.00761	0.01791	0.00635	0.00829	0.00119
769	O8	PZ″	−0.00067	−0.00313	0.00080	0.00457	−0.00182
770	O8	DZ**2	−0.00013	−0.00019	−0.00013	−0.00019	0.00003
771	O8	DXX-YY	0.00010	0.00034	0.00014	0.00019	0.00008
772	O8	DXY	0.00016	0.00053	0.00018	0.00013	0.00014
773	O8	DXZ	−0.00008	−0.00002	−0.00006	0.00010	−0.00004
774	O8	DYZ	0.00010	0.00034	0.00011	0.00008	0.00009
775	C6	S1	−0.00078	−0.00032	−0.00142	−0.00065	−0.00052
776	C6	S	−0.00135	−0.00077	−0.00240	−0.00117	−0.00088
777	C6	PX	−0.00049	0.00040	−0.00118	0.00003	−0.00048
778	C6	PY	0.00203	0.00013	0.00333	0.00124	0.00079
779	C6	PZ	−0.00161	−0.00090	−0.00339	−0.00227	−0.00120
780	C6	S'	0.00619	0.00938	0.00864	0.00628	0.00292

781	C6	PX'	-0.00121	0.00004	-0.00193	-0.00037	-0.00061
782	C6	PY'	0.00383	0.00314	0.00592	0.00319	0.00135
783	C6	PZ'	-0.00351	-0.00335	-0.00674	-0.00457	-0.00253
784	C6	S″	-0.01046	-0.05598	0.00624	-0.01494	0.00647
785	C6	PX″	0.00170	0.00527	-0.00534	0.00193	-0.00173
786	C6	PY″	0.00401	-0.00886	0.00662	-0.00110	0.00025
787	C6	PZ″	0.00090	-0.00060	-0.00579	-0.00842	-0.00237
788	C6	DZ**2	0.00026	0.00053	0.00070	0.00075	0.00020
789	C6	DXX-YY	0.00033	0.00001	0.00068	0.00032	0.00041
790	C6	DXY	-0.00006	0.00023	-0.00020	0.00032	-0.00050
791	C6	DXZ	0.00005	-0.00027	0.00008	-0.00020	0.00024
792	C6	DYZ	-0.00065	-0.00087	-0.00095	-0.00076	-0.00035
793	F4	S1	0.00010	-0.00014	0.00007	-0.00022	0.00002
794	F4	S	0.00016	-0.00022	0.00011	-0.00036	0.00003
795	F4	PX	0.00020	0.00048	0.00049	0.00068	0.00010
796	F4	PY	-0.00062	0.00091	-0.00042	0.00088	0.00006
797	F4	PZ	0.00055	-0.00084	0.00114	-0.00029	0.00061
798	F4	S'	-0.00049	0.00017	-0.00015	0.00101	-0.00006
799	F4	PX'	0.00013	0.00020	0.00050	0.00070	0.00009
800	F4	PY'	-0.00067	0.00188	-0.00040	0.00174	0.00017
801	F4	PZ'	0.00075	-0.00103	0.00146	-0.00044	0.00092
802	F4	S″	-0.00090	0.00773	-0.00210	0.00527	-0.00049
803	F4	PX″	0.00098	0.00447	0.00150	0.00312	0.00031
804	F4	PY″	-0.00204	-0.00039	-0.00168	-0.00020	-0.00007
805	F4	PZ″	0.00054	-0.00433	0.00268	-0.00138	0.00095
806	F4	DZ**2	0.00004	0.00005	0.00009	0.00007	0.00005
807	F4	DXX-YY	0.00000	0.00006	-0.00000	0.00005	-0.00000
808	F4	DXY	0.00002	0.00001	0.00006	0.00003	0.00001
809	F4	DXZ	0.00001	-0.00007	0.00003	-0.00001	0.00001
810	F4	DYZ	-0.00002	0.00002	-0.00006	-0.00001	-0.00003
811	F5	S1	0.00002	0.00010	-0.00007	-0.00000	0.00005
812	F5	S	0.00002	0.00017	-0.00012	-0.00000	0.00008
813	F5	PX	0.00024	-0.00016	-0.00001	0.00001	-0.00038
814	F5	PY	-0.00012	0.00003	0.00014	0.00015	-0.00007
815	F5	PZ	0.00066	0.00111	0.00105	0.00143	0.00009
816	F5	S'	0.00018	-0.00085	0.00053	-0.00008	-0.00024
817	F5	PX'	0.00050	0.00030	0.00010	0.00040	-0.00078
818	F5	PY'	0.00018	0.00046	0.00077	0.00046	-0.00010
819	F5	PZ'	0.00098	0.00146	0.00141	0.00191	-0.00013
820	F5	S″	-0.00151	0.00242	0.00071	0.00119	-0.00025
821	F5	PX″	-0.00038	-0.00461	-0.00078	-0.00247	0.00042
822	F5	PY″	-0.00171	-0.00134	-0.00201	-0.00073	-0.00011
823	F5	PZ″	0.00075	0.00142	0.00188	0.00263	0.00105
824	F5	DZ**2	0.00004	0.00001	0.00009	0.00006	0.00003
825	F5	DXX-YY	0.00002	0.00008	0.00004	0.00004	0.00000
826	F5	DXY	-0.00005	0.00000	-0.00009	-0.00005	0.00002
827	F5	DXZ	0.00001	0.00004	0.00004	0.00004	0.00002
828	F5	DYZ	-0.00002	-0.00002	-0.00003	-0.00005	0.00002
829	F6	S1	0.00018	0.00018	0.00038	0.00027	0.00019
830	F6	S	0.00031	0.00031	0.00064	0.00045	0.00031
831	F6	PX	0.00020	-0.00054	0.00034	-0.00029	0.00003
832	F6	PY	-0.00156	-0.00098	-0.00293	-0.00185	-0.00110
833	F6	PZ	0.00047	0.00053	0.00061	0.00058	0.00004
834	F6	S'	-0.00134	-0.00142	-0.00216	-0.00148	-0.00093
835	F6	PX'	0.00022	-0.00079	0.00030	-0.00035	-0.00004
836	F6	PY'	-0.00217	-0.00145	-0.00401	-0.00259	-0.00147
837	F6	PZ'	0.00035	0.00018	0.00062	0.00052	0.00004
838	F6	S″	0.00095	0.00183	-0.00388	-0.00317	-0.00302
839	F6	PX″	0.00062	-0.00058	0.00143	-0.00064	0.00024
840	F6	PY″	-0.00160	0.00088	-0.00520	-0.00284	-0.00241
841	F6	PZ″	0.00253	0.00444	0.00212	0.00255	-0.00005
842	F6	DZ**2	-0.00004	0.00000	-0.00010	-0.00005	-0.00003
843	F6	DXX-YY	-0.00005	-0.00006	-0.00009	-0.00006	-0.00003
844	F6	DXY	-0.00000	0.00004	-0.00001	0.00003	-0.00001
845	F6	DXZ	-0.00001	-0.00001	-0.00002	-0.00002	-0.00001
846	F6	DYZ	0.00001	0.00006	-0.00001	0.00000	0.00000
847	H46	S	0.00000	-0.00000	0.00000	-0.00000	-0.00000
848	H46	S'	0.00000	-0.00000	0.00000	0.00000	-0.00000
849	H46	S″	-0.00000	0.00000	0.00000	-0.00000	0.00000
850	H46	PX	0.00000	0.00000	-0.00000	-0.00000	0.00000
851	H46	PY	-0.00025	-0.00438	-0.00054	-0.00528	-0.00122
852	H46	PZ	-0.00000	-0.00000	0.00000	0.00000	0.00000
853	H47	S	0.00000	0.00000	0.00000	0.00000	0.00000
854	H47	S'	0.00000	0.00000	0.00000	0.00000	0.00000
855	H47	S″	-0.00000	-0.00000	0.00000	0.00000	0.00000
856	H47	PX	0.00000	0.00000	-0.00000	-0.00000	0.00000
857	H47	PY	-0.00046	-0.00815	0.00210	0.01030	0.00045
858	H47	PZ	-0.00000	-0.00000	0.00000	0.00000	-0.00000
859	H48	S	0.00000	0.00000	0.00000	0.00000	-0.00000
860	H48	S'	-0.00000	-0.00000	-0.00000	0.00000	-0.00000
861	H48	S″	-0.00000	-0.00000	-0.00000	-0.00000	-0.00000
862	H48	PX	-0.00000	-0.00000	0.00000	0.00000	0.00000
863	H48	PY	0.00097	0.01274	-0.00104	-0.00423	0.00060
864	H48	PZ	0.00000	0.00000	-0.00000	-0.00000	-0.00000
865	H49	S	0.00000	0.00000	0.00000	0.00000	0.00000
866	H49	S'	-0.00000	-0.00000	-0.00000	-0.00000	-0.00000
867	H49	S″	0.00000	0.00000	-0.00000	0.00000	0.00000
868	H49	PX	-0.00000	-0.00000	0.00000	0.00000	0.00000
869	H49	PY	0.00015	0.00300	-0.00156	-0.00862	-0.00126
870	H49	PZ	-0.00000	0.00000	-0.00000	-0.00000	-0.00000
871	H50	S	-0.00000	-0.00000	-0.00000	-0.00000	-0.00000
872	H50	S'	0.00000	0.00000	0.00000	0.00000	-0.00000
873	H50	S″	0.00000	0.00000	0.00000	0.00000	-0.00000
874	H50	PX	0.00000	0.00000	-0.00000	-0.00000	-0.00000
875	H50	PY	-0.00078	-0.01129	0.00216	0.01100	0.00102
876	H50	PZ	-0.00000	-0.00000	0.00000	0.00000	-0.00000
877	H51	S	-0.00210	-0.02961	0.00490	0.02450	0.00197
878	H51	S'	-0.00449	-0.06378	0.00998	0.04884	0.00250
879	H51	S″	-0.00523	-0.07735	0.01681	0.08704	0.00850
880	H51	PX	-0.00007	-0.00091	-0.00002	-0.00025	-0.00016
881	H51	PY	-0.00002	-0.00024	-0.00006	-0.00040	-0.00012
882	H51	PZ	0.00015	0.00229	-0.00068	-0.00362	-0.00047
883	H52	S	-0.00000	-0.00000	0.00000	0.00000	0.00000
884	H52	S'	-0.00000	-0.00000	0.00000	0.00000	0.00000
885	H52	S″	-0.00000	-0.00000	0.00000	0.00000	0.00000
886	H52	PX	-0.00000	-0.00000	0.00000	0.00000	0.00000
887	H52	PY	0.00013	0.00177	-0.00024	-0.00113	-0.00003
888	H52	PZ	0.00000	0.00000	-0.00000	-0.00000	-0.00000
889	H53	S	0.00210	0.02961	-0.00490	-0.02450	-0.00197
890	H53	S'	0.00449	0.06378	-0.00998	-0.04884	-0.00250
891	H53	S″	0.00523	0.07735	-0.01681	-0.08704	-0.00850
892	H53	PX	0.00007	0.00091	0.00002	0.00025	0.00016
893	H53	PY	-0.00002	-0.00024	-0.00006	-0.00040	-0.00012
894	H53	PZ	-0.00015	-0.00229	0.00068	0.00362	0.00047
895	H54	S	-0.00000	-0.00000	-0.00000	-0.00000	-0.00000
896	H54	S'	-0.00000	-0.00000	-0.00000	-0.00000	0.00000
897	H54	S″	0.00000	-0.00000	0.00000	-0.00000	0.00000
898	H54	PX	0.00000	0.00000	0.00000	-0.00000	-0.00000
899	H54	PY	0.00002	0.00084	0.00001	0.00024	0.00022
900	H54	PZ	0.00000	-0.00000	-0.00000	-0.00000	-0.00000
901	H55	S	-0.00072	-0.01505	-0.00417	-0.02179	0.00283
902	H55	S'	-0.00475	-0.03899	-0.01329	-0.07022	0.00469
903	H55	S″	-0.05062	-0.05257	-0.05782	-0.11677	0.10857
904	H55	PX	0.00126	-0.00089	0.00070	-0.00051	-0.00158
905	H55	PY	-0.00001	-0.00044	0.00006	-0.00033	-0.00103
906	H55	PZ	-0.00024	0.00020	-0.00008	0.00019	0.00103
907	H56	S	0.00072	0.01505	0.00417	0.02179	-0.00283
908	H56	S'	0.00475	0.03899	0.01329	0.07022	-0.00469
909	H56	S″	0.05062	0.05257	0.05782	0.11677	-0.10857
910	H56	PX	-0.00126	0.00089	-0.00070	0.00051	0.00158
911	H56	PY	-0.00001	-0.00044	0.00006	-0.00033	-0.00103
912	H56	PZ	0.00024	-0.00020	0.00008	-0.00019	-0.00103
913	H57	S	0.00150	0.01313	0.00563	0.03198	0.00072
914	H57	S'	0.00377	0.02671	0.01319	0.07125	-0.00144
915	H57	S″	0.00826	0.09354	0.01779	0.11969	0.00124
916	H57	PX	-0.00005	-0.00147	0.00052	0.00251	0.00006
917	H57	PY	0.00000	-0.00046	0.00001	-0.00034	-0.00016
918	H57	PZ	-0.00005	-0.00123	-0.00042	-0.00303	-0.00034
919	H58	S	-0.00150	-0.01313	-0.00563	-0.03198	-0.00072
920	H58	S'	-0.00377	-0.02671	-0.01319	-0.07125	0.00144
921	H58	S″	-0.00826	-0.09354	-0.01779	-0.11969	-0.00124
922	H58	PX	0.00005	0.00147	-0.00052	-0.00251	-0.00006
923	H58	PY	0.00000	-0.00046	0.00001	-0.00034	-0.00016
924	H58	PZ	0.00005	0.00123	0.00042	0.00303	0.00034

925	H59	S	0.00000	0.00000	0.00000	0.00000	0.00000
926	H59	S'	0.00000	0.00000	-0.00000	-0.00000	-0.00000
927	H59	S"	0.00000	0.00000	-0.00000	-0.00000	0.00000
928	H59	PX	-0.00000	0.00000	0.00000	0.00000	0.00000
929	H59	PY	-0.00007	-0.00002	-0.00018	-0.00082	-0.00004
930	H59	PZ	0.00000	0.00000	-0.00000	-0.00000	0.00000
931	H60	S	-0.00000	0.00000	-0.00000	0.00000	0.00000
932	H60	S'	0.00000	0.00000	-0.00000	0.00000	0.00000
933	H60	S"	0.00000	0.00000	0.00000	0.00000	-0.00000
934	H60	PX	0.00000	0.00000	0.00000	0.00000	0.00000
935	H60	PY	-0.00462	0.00031	-0.00708	0.00177	-0.00191
936	H60	PZ	-0.00000	0.00000	-0.00000	0.00000	-0.00000
937	H61	S	0.00000	-0.00000	0.00000	-0.00000	-0.00000
938	H61	S'	0.00000	-0.00000	0.00000	0.00000	-0.00000
939	H61	S"	-0.00000	0.00000	0.00000	-0.00000	-0.00000
940	H61	PX	-0.00000	0.00000	0.00000	0.00000	0.00000
941	H61	PY	-0.00883	0.00097	0.01005	-0.00150	-0.00055
942	H61	PZ	0.00000	-0.00000	-0.00000	0.00000	-0.00000
943	H62	S	-0.00000	0.00000	0.00000	-0.00000	0.00000
944	H62	S'	-0.00000	0.00000	0.00000	0.00000	0.00000
945	H62	S"	-0.00000	0.00000	0.00000	0.00000	0.00000
946	H62	PX	0.00000	-0.00000	0.00000	0.00000	0.00000
947	H62	PY	0.01300	-0.00102	-0.00317	0.00055	-0.01426
948	H62	PZ	-0.00000	0.00000	0.00000	0.00000	-0.00000
949	H63	S	-0.00000	0.00000	0.00000	-0.00000	-0.00000
950	H63	S'	-0.00000	0.00000	0.00000	0.00000	-0.00000
951	H63	S"	0.00000	-0.00000	-0.00000	0.00000	-0.00001
952	H63	PX	0.00000	-0.00000	-0.00000	0.00000	0.00000
953	H63	PY	0.00334	-0.00039	-0.00918	0.00166	0.02073
954	H63	PZ	-0.00000	-0.00000	0.00000	-0.00000	-0.00000
955	H64	S	0.00000	-0.00000	-0.00000	0.00000	0.00000
956	H64	S'	0.00000	-0.00000	0.00000	-0.00000	-0.00000
957	H64	S"	-0.00000	-0.00000	0.00000	0.00000	0.00000
958	H64	PX	-0.00000	0.00000	0.00000	-0.00000	0.00000
959	H64	PY	-0.01174	0.00109	0.01115	-0.00185	-0.01526
960	H64	PZ	0.00000	-0.00000	-0.00000	-0.00000	-0.00000
961	H65	S	-0.03053	0.00276	0.02437	-0.00397	-0.02759
962	H65	S'	-0.06552	0.00590	0.04716	-0.00757	-0.02190
963	H65	S"	-0.07877	0.00742	0.08579	-0.01461	-0.11254
964	H65	PX	0.00097	-0.00008	0.00028	-0.00007	-0.00293
965	H65	PY	-0.00024	0.00002	-0.00046	0.00009	0.00219
966	H65	PZ	-0.00234	0.00024	0.00373	-0.00065	-0.00758
967	H66	S	0.03053	-0.00276	-0.02437	0.00397	0.02759
968	H66	S'	0.06552	-0.00590	-0.04716	0.00757	0.02190
969	H66	S"	0.07877	-0.00742	-0.08579	0.01461	0.11254
970	H66	PX	-0.00097	0.00008	-0.00028	0.00007	0.00293
971	H66	PY	-0.00024	0.00002	-0.00046	0.00009	0.00219
972	H66	PZ	0.00234	-0.00024	-0.00373	0.00065	0.00758
973	H67	S	-0.00000	0.00000	0.00000	-0.00000	-0.00000
974	H67	S'	-0.00000	0.00000	0.00000	-0.00000	-0.00000
975	H67	S"	-0.00000	0.00000	0.00000	-0.00000	-0.00000
976	H67	PX	-0.00000	0.00000	-0.00000	0.00000	0.00000
977	H67	PY	0.00180	-0.00016	-0.00106	0.00017	-0.00002
978	H67	PZ	-0.00000	0.00000	0.00000	-0.00000	-0.00000
979	H68	S	0.01479	-0.00155	0.02157	-0.00422	0.01138
980	H68	S'	0.02593	-0.00758	0.03522	-0.01031	0.01470
981	H68	S"	0.01970	-0.00292	0.02658	-0.00606	0.02002
982	H68	PX	0.00033	-0.00031	-0.00005	-0.00025	-0.00063
983	H68	PY	-0.00030	0.00031	-0.00016	0.00025	0.00023
984	H68	PZ	-0.00151	-0.00022	-0.00397	0.00062	-0.00277
985	H69	S	-0.01479	0.00155	-0.02157	0.00422	-0.01138
986	H69	S'	-0.02593	0.00758	-0.03522	0.01031	-0.01470
987	H69	S"	-0.01970	0.00292	-0.02658	0.00606	-0.02002
988	H69	PX	-0.00033	0.00031	0.00005	0.00025	0.00063
989	H69	PY	-0.00030	0.00031	-0.00016	0.00025	0.00023
990	H69	PZ	0.00151	0.00022	0.00397	-0.00062	0.00277
991	H70	S	0.00000	0.00000	0.00000	-0.00000	-0.00000
992	H70	S'	0.00000	-0.00000	0.00000	0.00000	-0.00000
993	H70	S"	0.00000	0.00000	0.00000	0.00000	0.00000
994	H70	PX	-0.00000	0.00000	-0.00000	0.00000	0.00000
995	H70	PY	0.00138	0.00195	0.00189	0.00037	0.00079
996	H70	PZ	0.00000	0.00000	0.00000	0.00000	0.00000
997	H71	S	0.01095	-0.00031	0.03284	-0.00682	0.02024
998	H71	S'	0.02197	-0.00059	0.07122	-0.01486	0.06725
999	H71	S"	0.09221	-0.00701	0.12787	-0.03371	0.11129
1000	H71	PX	0.00173	-0.00019	-0.00250	0.00041	-0.00199
1001	H71	PY	-0.00061	0.00005	-0.00062	0.00020	0.00041
1002	H71	PZ	0.00125	-0.00007	0.00350	-0.00082	0.00212
1003	H72	S	-0.01095	0.00031	-0.03284	0.00682	-0.02024
1004	H72	S'	-0.02197	0.00059	-0.07122	0.01486	-0.06725
1005	H72	S"	-0.09221	0.00701	-0.12787	0.03371	-0.11130
1006	H72	PX	-0.00173	0.00019	0.00250	-0.00041	0.00199
1007	H72	PY	-0.00061	0.00005	-0.00062	0.00020	0.00041
1008	H72	PZ	-0.00125	0.00007	-0.00350	0.00082	-0.00212
1009	H73	S	0.00000	-0.00000	0.00000	-0.00000	-0.00000
1010	H73	S'	0.00000	-0.00000	0.00000	-0.00000	-0.00000
1011	H73	S"	0.00000	-0.00000	0.00000	-0.00000	-0.00000
1012	H73	PX	0.00000	-0.00000	-0.00000	0.00000	0.00000
1013	H73	PY	0.00006	-0.00002	-0.00086	0.00016	-0.00036
1014	H73	PZ	-0.00000	-0.00000	0.00000	-0.00000	0.00000

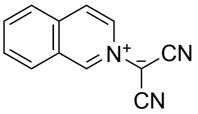

1a

SPARTAN '18 Quantum Mechanics Program: (x86/Darwin) build 1.3.0

Job type: Geometry optimization.
Method: RB3LYP
Basis set: 6-31G(D)
Number of basis functions: 239
Number of electrons: 100
Parallel Job: 8 threads

SCF model:
A restricted hybrid HF-DFT SCF calculation will be
performed using Pulay DIIS + Geometric Direct Minimization

Optimization:

Step	Energy	Max Grad.	Max Dist.	
1	-625.632394	0.043248	0.121413	
2	-625.651939	0.019627	0.116814	
3	-625.658187	0.010374	0.065019	
4	-625.660078	0.005667	0.063700	
5	-625.660987	0.004918	0.076735	
6	-625.661601	0.004894	0.043262	
7	-625.661818	0.002419	0.028972	
8	-625.661884	0.000785	0.008293	
8	-625.661884	0.001282	0.037009	Switching to cartesian
9	-625.661890	0.000892	0.008318	
10	-625.661872	0.004320	0.022853	
11	-625.661890	0.000707	0.012895	
12	-625.661890	0.000980	0.005151	
13	-625.661892	0.000042	0.000806	

Reason for exit: Successful completion
Quantum Calculation CPU Time : 25:10.27
Quantum Calculation Wall Time: 3:25.25

SPARTAN '18 Properties Program: (x86/Darwin) build 1.3.0
Use of molecular symmetry enabled

Cartesian Coordinates (Angstroms)

Atom	X	Y	Z

1	C	C1	1.2012142	0.0000003	2.4380257
2	C	C4	−1.5350777	−0.0000004	1.7371603
3	C	C2	0.2208799	0.0000002	3.4067560
4	C	C6	0.8437978	0.0000001	1.0674731
5	C	C5	−0.5448025	−0.0000003	0.7184055
6	C	C3	−1.1539057	−0.0000002	3.0582995
7	C	C7	−0.8905418	−0.0000003	−0.6460101
8	N	N1	0.0320006	−0.0000001	−1.6087550
9	C	C9	1.3728929	0.0000000	−1.2917911
10	C	C10	1.7908106	0.0000001	0.0095683
11	C	C8	−0.3844595	0.0000003	−3.0173063
12	C	C11	−0.5001265	1.2542076	−3.6228956
13	C	C12	−0.5001275	−1.2542073	−3.6228959
14	N	N2	−0.5940119	2.3252791	−4.0919675
15	N	N3	−0.5940142	−2.3252792	−4.0919681
16	H	H16	2.2519742	0.0000005	2.7132212
17	H	H17	−2.5846115	−0.0000006	1.4572489
18	H	H18	0.5019742	0.0000005	4.4559406
19	H	H19	−1.9046336	−0.0000002	3.8423962
20	H	H20	−1.9178437	−0.0000004	−0.9911467
21	H	H21	2.0347173	0.0000001	−2.1470733
22	H	H22	2.8538947	0.0000003	0.2273142

12. References

The references are numbered according to the numbering in the manuscript file.

2 (*e*) Z. Li, N. Xu, N. Guo, Y. Zhou, L. Lin and X. Feng, *Chem. Eur. J.*, 2021, **27**, 14841–14845.

7 (*a*) K. Ishihara and M. Fushimi, *Org. Lett.*, 2006, **8**, 1921−1924; (*f*) M. Hori, A. Sakakura and K. Ishihara, *J. Am. Chem. Soc.*, 2014, **136***,* 13198–13201; (*l*) W. Guo, M. Hori, Y. Ogua, K. Nishimura, K. Oki, T. Ikai, E. Yashima and K. Ishihara, *J. Am. Chem. Soc.*, 2023, **145***,* 27080–27088.

8 (*e*) N. Fernandez, L. Carrillo, J. L. Vicario, D. Badia and E. Reyes, *Chem. Commun.*, 2011, **47***,* 12313–12315.

12 (*a*) Q. Yao, Z. Wang, Y. H. Zhang, X. H. Liu, L. L. Lin and X. Feng, *J. Org. Chem*., 2015, **80**, 5704−5712; (*b*) Y. Zheng, Y. Yao, L. Ye, Z. Shi, X. Li and Z. Zhao, *Tetrahedron*, 2016, **72**, 973−978.

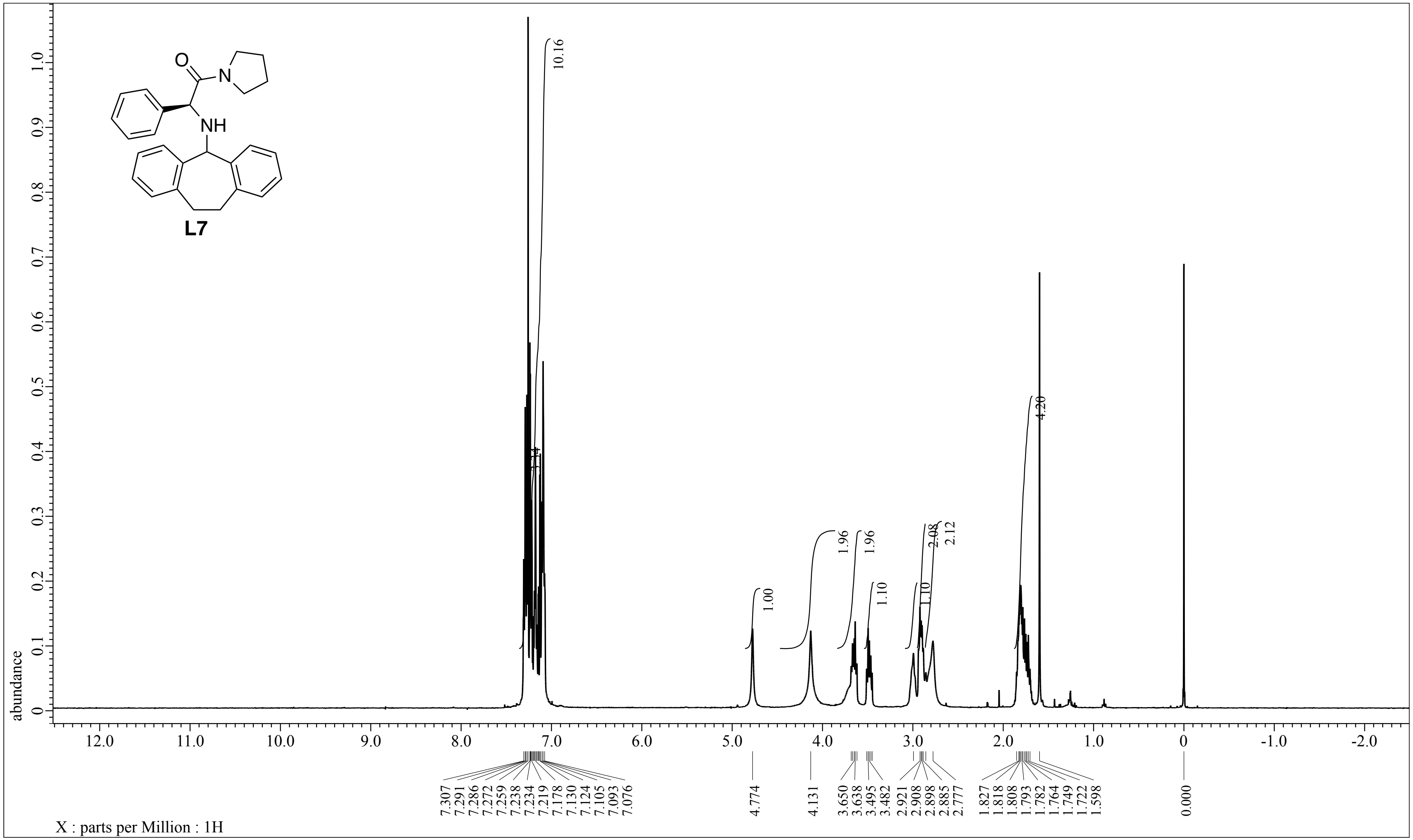

O
N
NH
L7
abundance
X : parts per Million : 1H
10.16
1.00
1.96
1.96
1.10
1.10
2.08
2.12
4.20
7.307
7.291
7.286
7.272
7.259
7.238
7.234
7.219
7.178
7.130
7.124
7.105
7.093
7.076
4.774
4.131
3.650
3.638
3.495
3.482
2.921
2.908
2.898
2.885
2.777
1.827
1.818
1.808
1.793
1.782
1.764
1.749
1.722
1.598
0.000

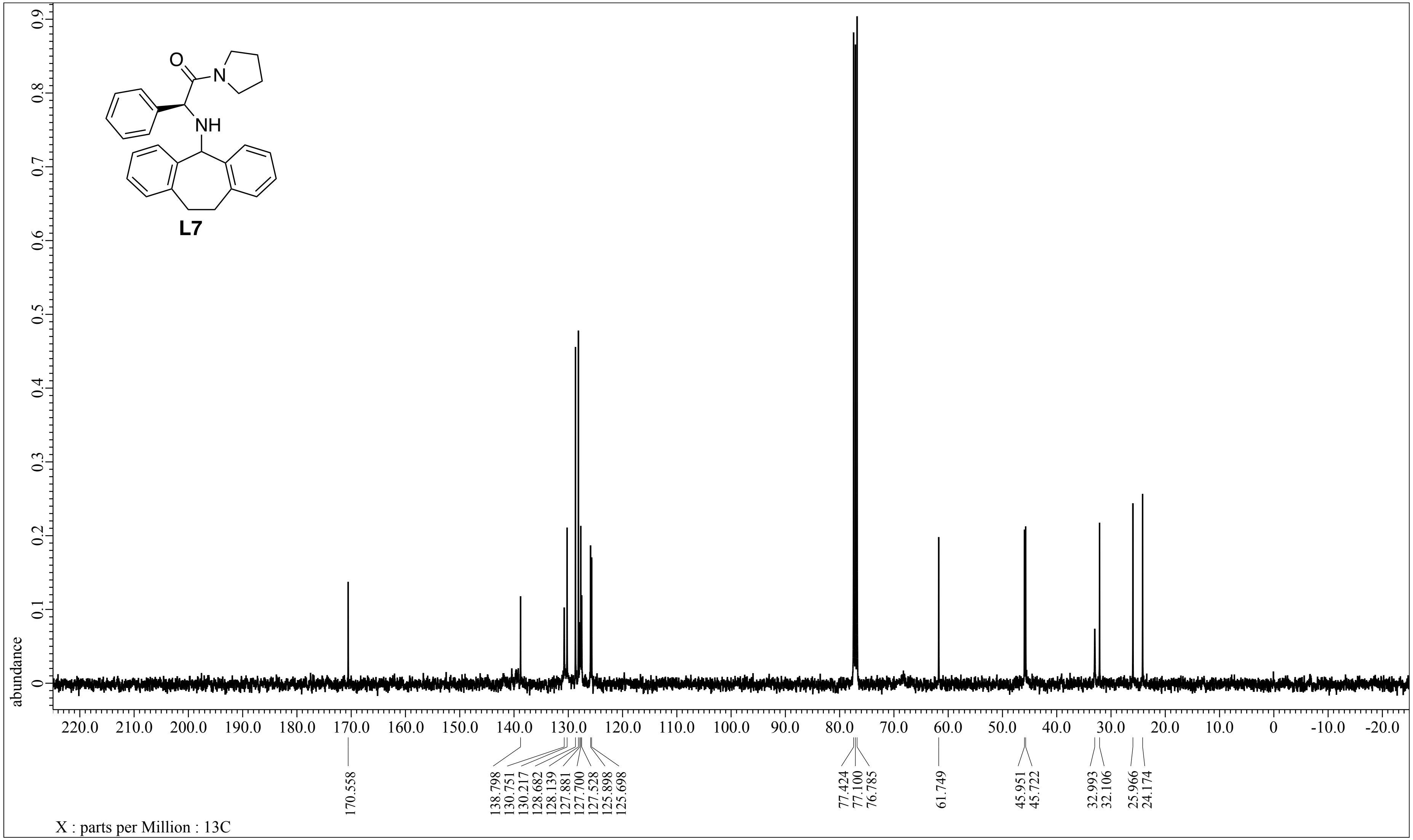

O
N
NH
L7
abundance
X : parts per Million : 13C
170.558
138.798
130.751
130.217
128.682
128.139
127.881
127.700
127.528
125.898
125.698
77.424
77.100
76.785
61.749
45.951
45.722
32.993
32.106
25.966
24.174

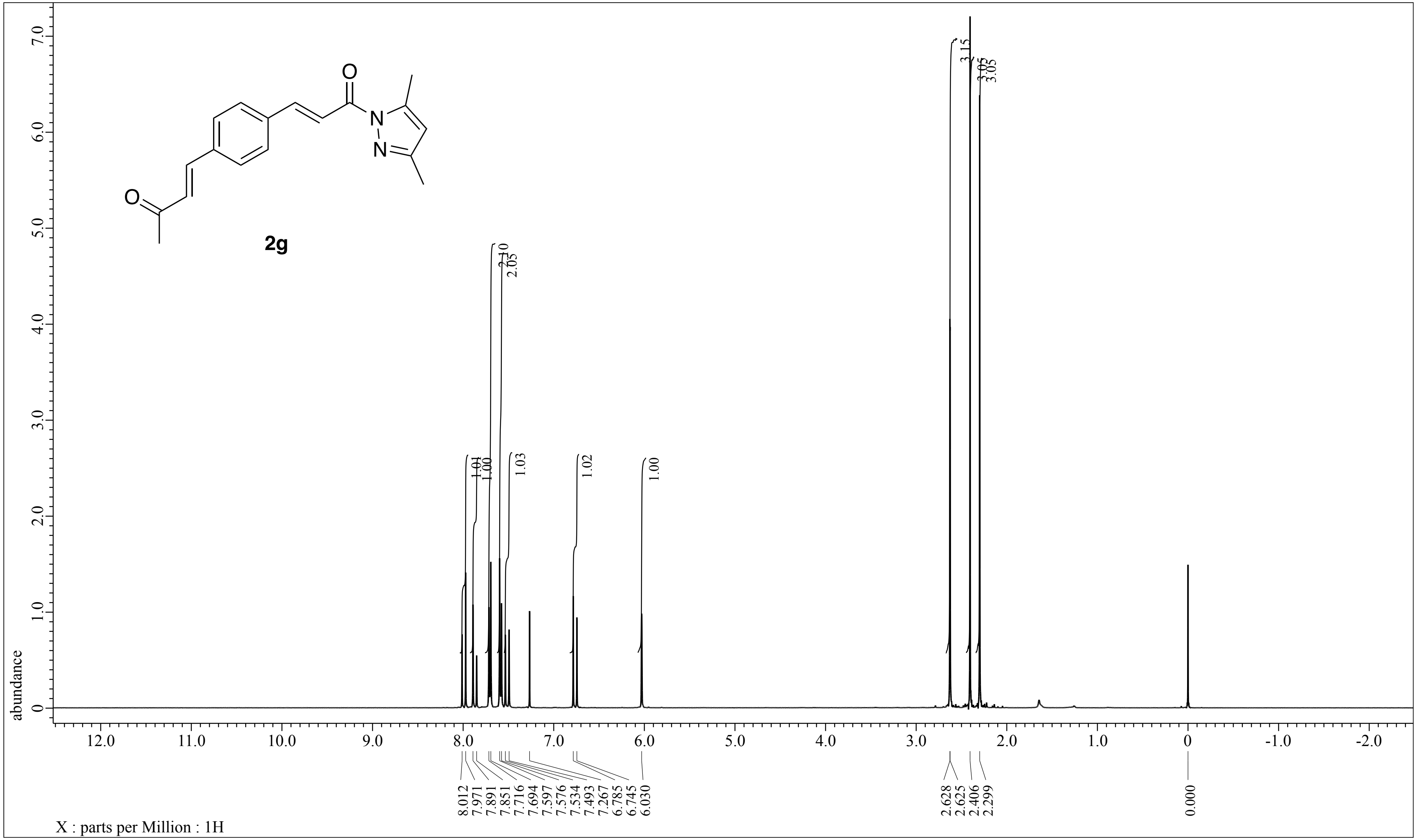
2g
abundance
X : parts per Million : 1H
8.012
7.971
7.891
7.851
7.716
7.694
7.597
7.576
7.534
7.493
7.267
6.785
6.745
6.030
2.628
2.625
2.406
2.299
0.000
1.01
1.00
2.10
2.05
1.03
1.02
1.00
3.15
3.07
3.05

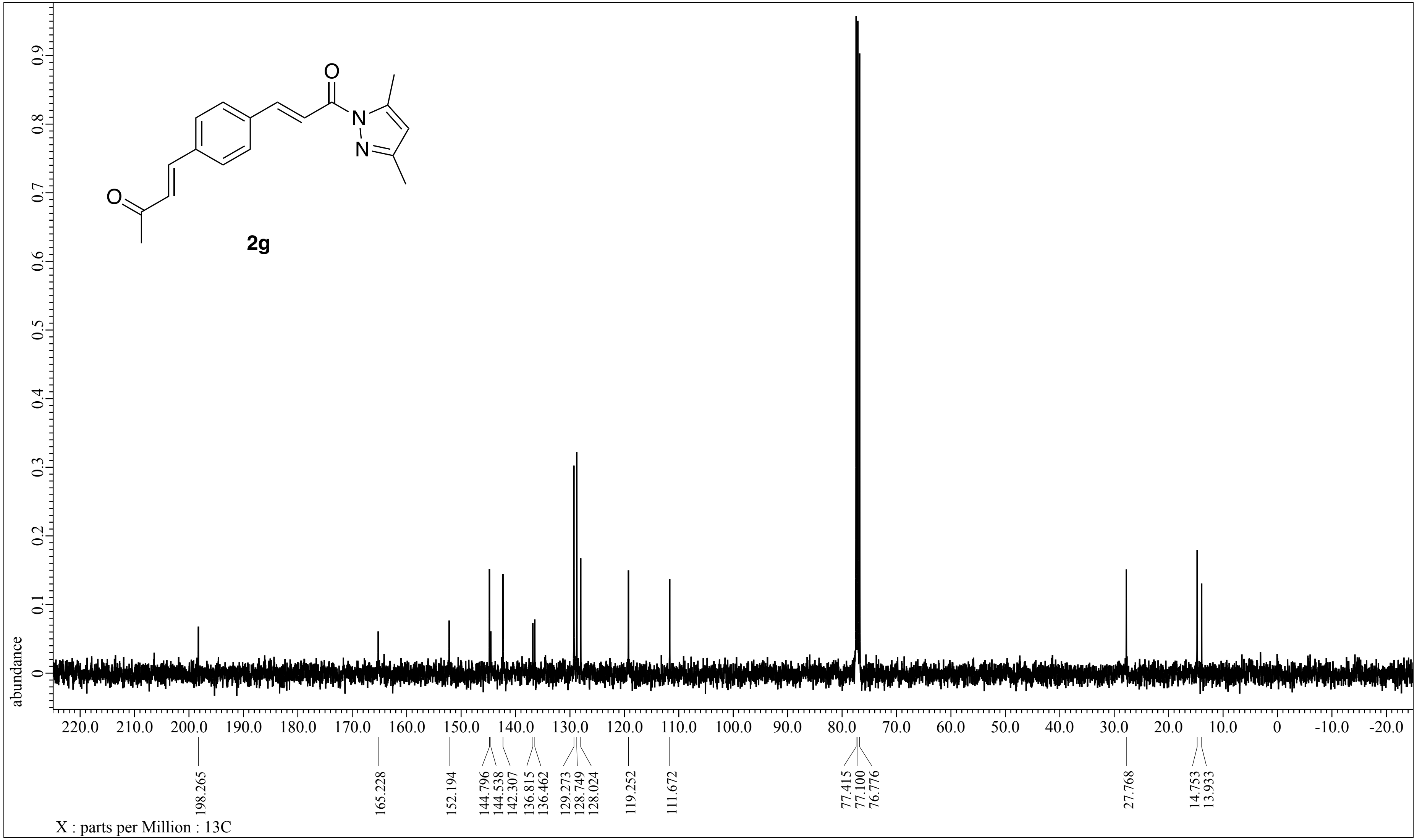
2g
abundance
X : parts per Million : 13C
198.265
165.228
152.194
144.796
144.538
142.307
136.815
136.462
129.273
128.749
128.024
119.252
111.672
77.415
77.100
76.776
27.768
14.753
13.933

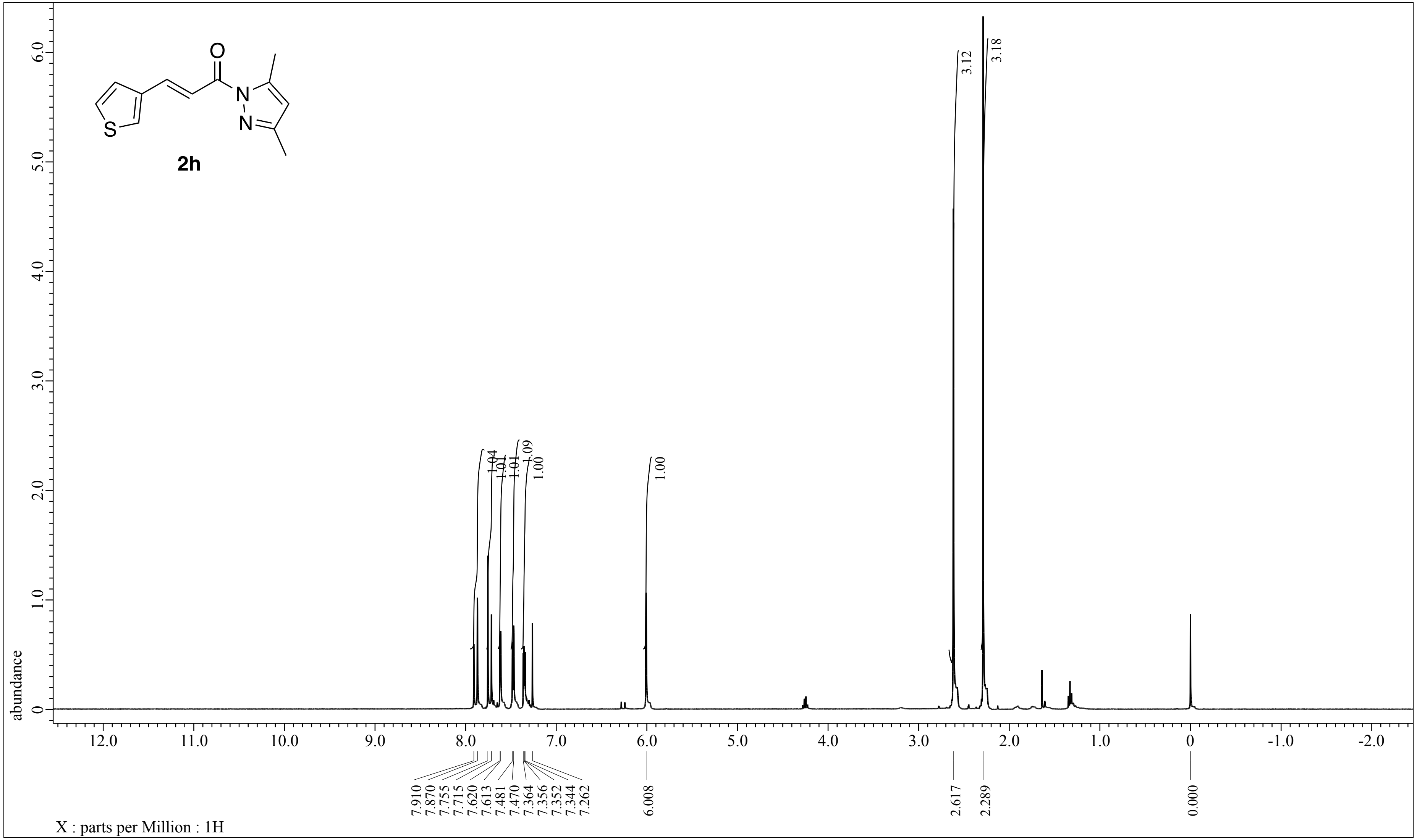

O
N
N
S
2h
abundance
X : parts per Million : 1H
7.910
7.870
7.755
7.715
7.620
7.613
7.481
7.470
7.364
7.356
7.352
7.344
7.262
6.008
2.617
2.289
0.000
1.04
1.01
1.01
1.09
1.00
1.00
3.12
3.18

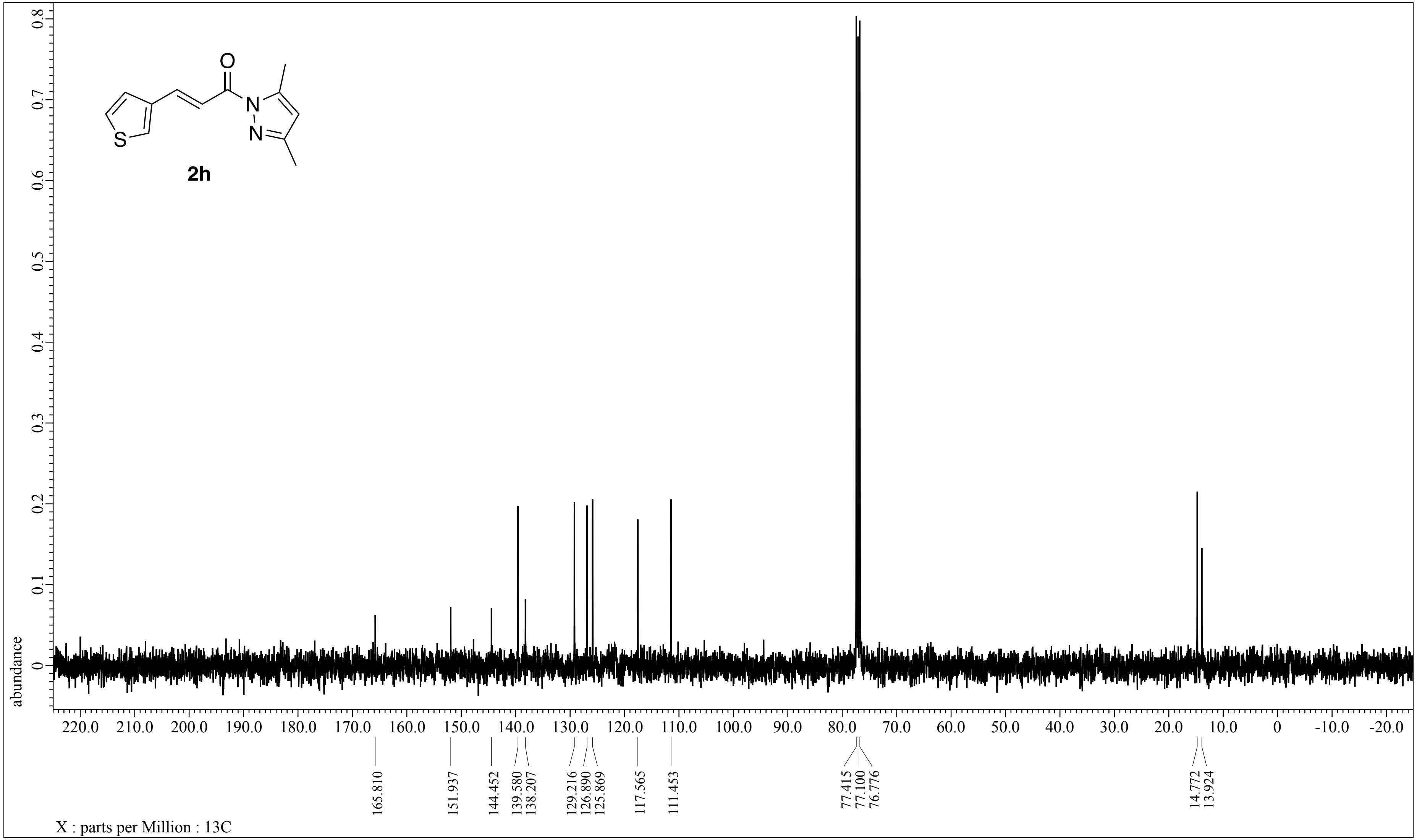

O
N
N
S
2h
abundance
0.8
0.7
0.6
0.5
0.4
0.3
0.2
0.1
0
220.0 210.0 200.0 190.0 180.0 170.0 160.0 150.0 140.0 130.0 120.0 110.0 100.0 90.0 80.0 70.0 60.0 50.0 40.0 30.0 20.0 10.0 0 -10.0 -20.0
165.810
151.937
144.452
139.580
138.207
129.216
126.890
125.869
117.565
111.453
77.415
77.100
76.776
14.772
13.924
X : parts per Million : 13C

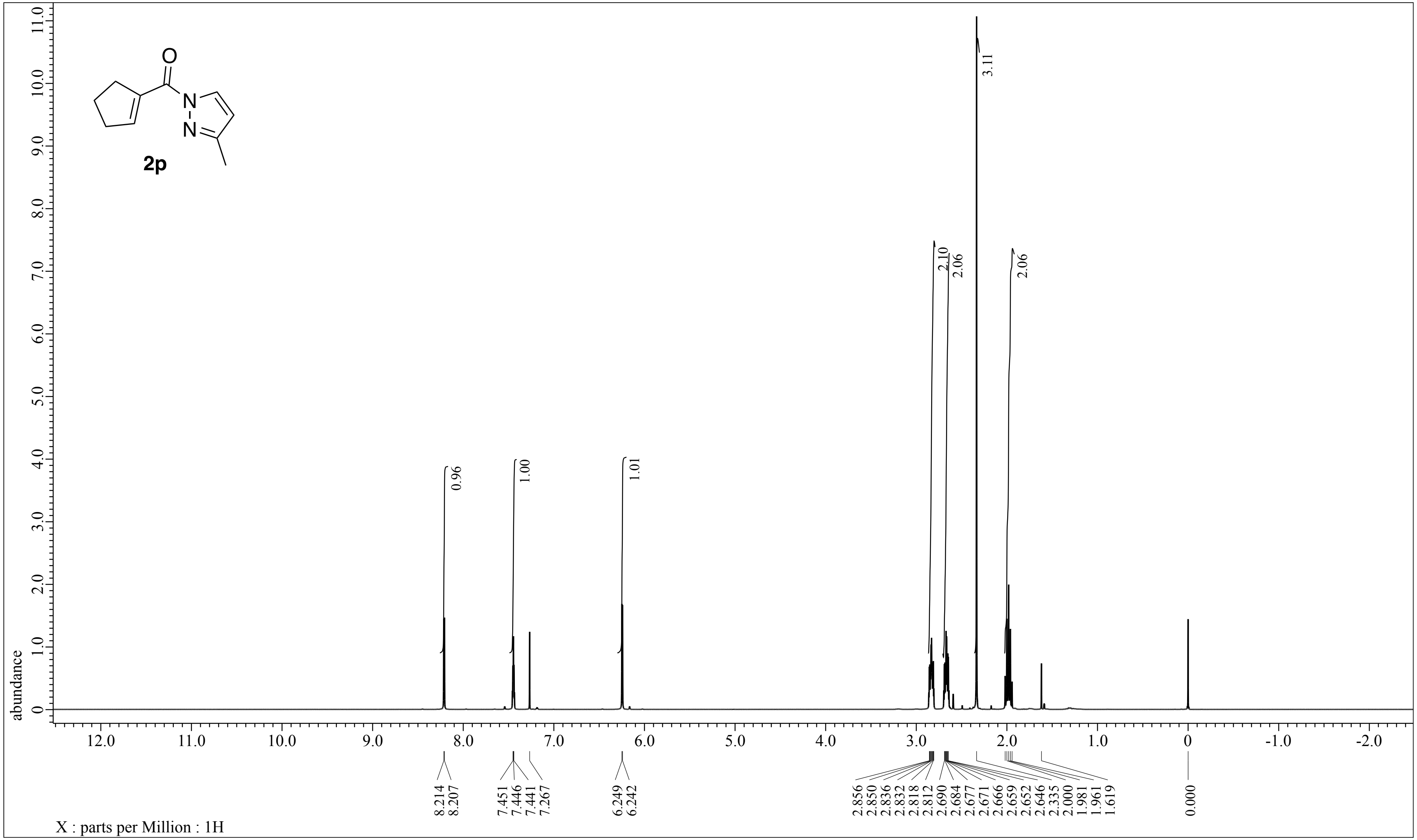
2p
abundance
X : parts per Million : 1H
0.96
1.00
1.01
2.10
2.06
3.11
2.06
8.214
8.207
7.451
7.446
7.441
7.267
6.249
6.242
2.856
2.850
2.836
2.832
2.818
2.812
2.690
2.684
2.677
2.671
2.666
2.659
2.652
2.646
2.335
2.000
1.981
1.961
1.619
0.000

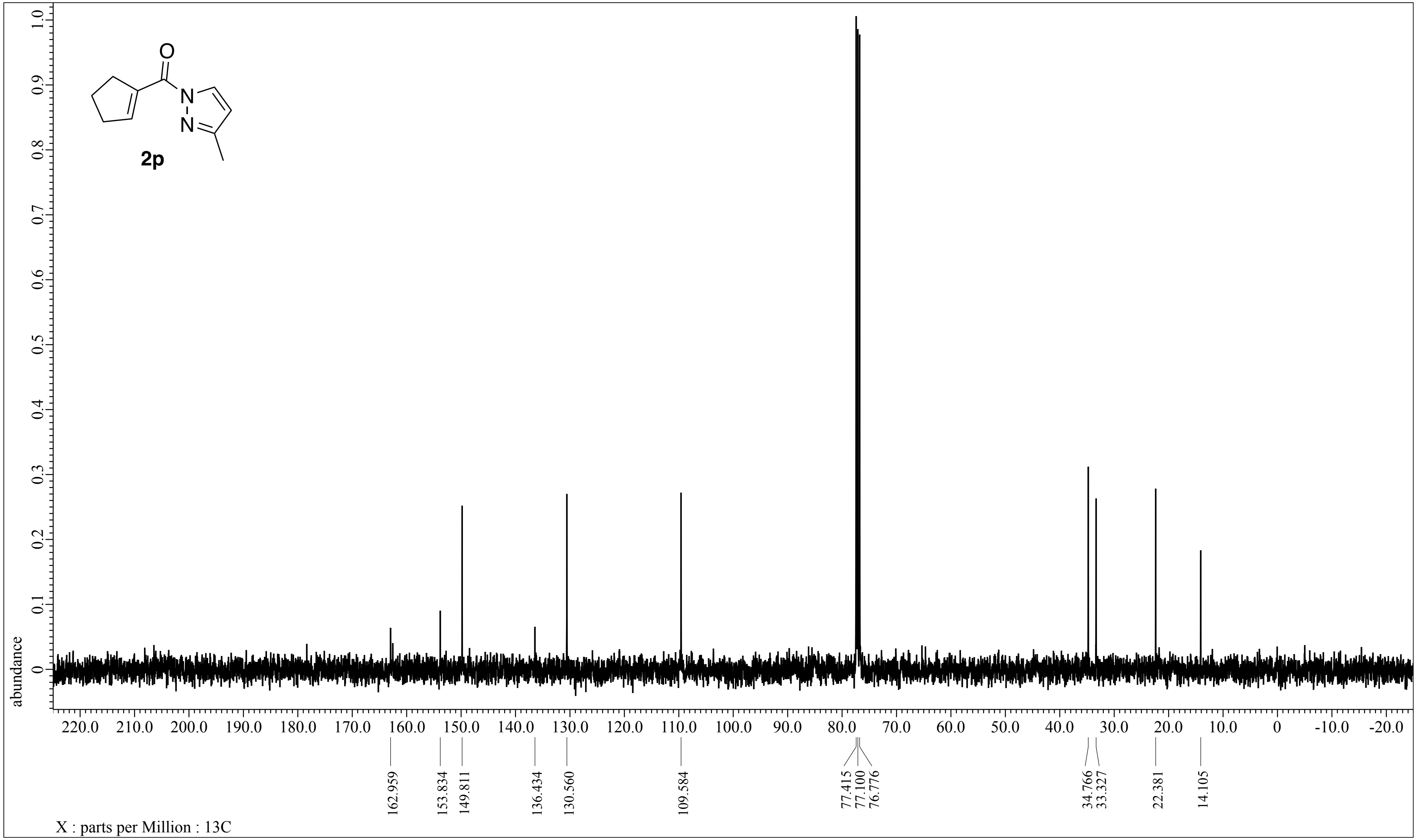

O
N
N
2p
abundance
1.0
0.9
0.8
0.7
0.6
0.5
0.4
0.3
0.2
0.1
0
220.0 210.0 200.0 190.0 180.0 170.0 160.0 150.0 140.0 130.0 120.0 110.0 100.0 90.0 80.0 70.0 60.0 50.0 40.0 30.0 20.0 10.0 0 -10.0 -20.0
162.959
153.834
149.811
136.434
130.560
109.584
77.415
77.100
76.776
34.766
33.327
22.381
14.105
X : parts per Million : 13C

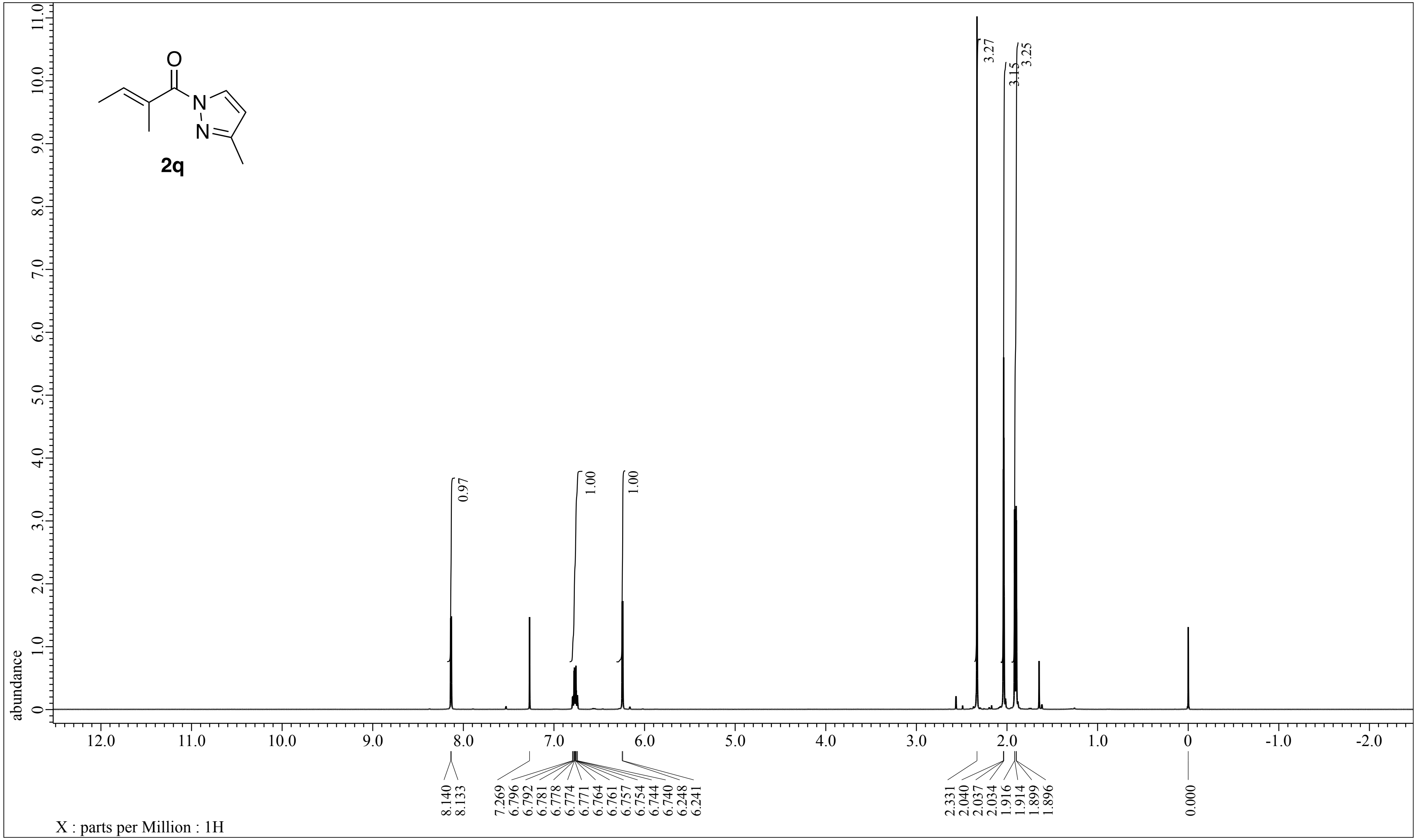
2q
abundance
X : parts per Million : 1H
0.97
1.00
1.00
3.27
3.15
3.25
8.140
8.133
7.269
6.796
6.792
6.781
6.778
6.774
6.771
6.764
6.761
6.757
6.754
6.744
6.740
6.248
6.241
2.331
2.040
2.037
2.034
1.916
1.914
1.899
1.896
0.000

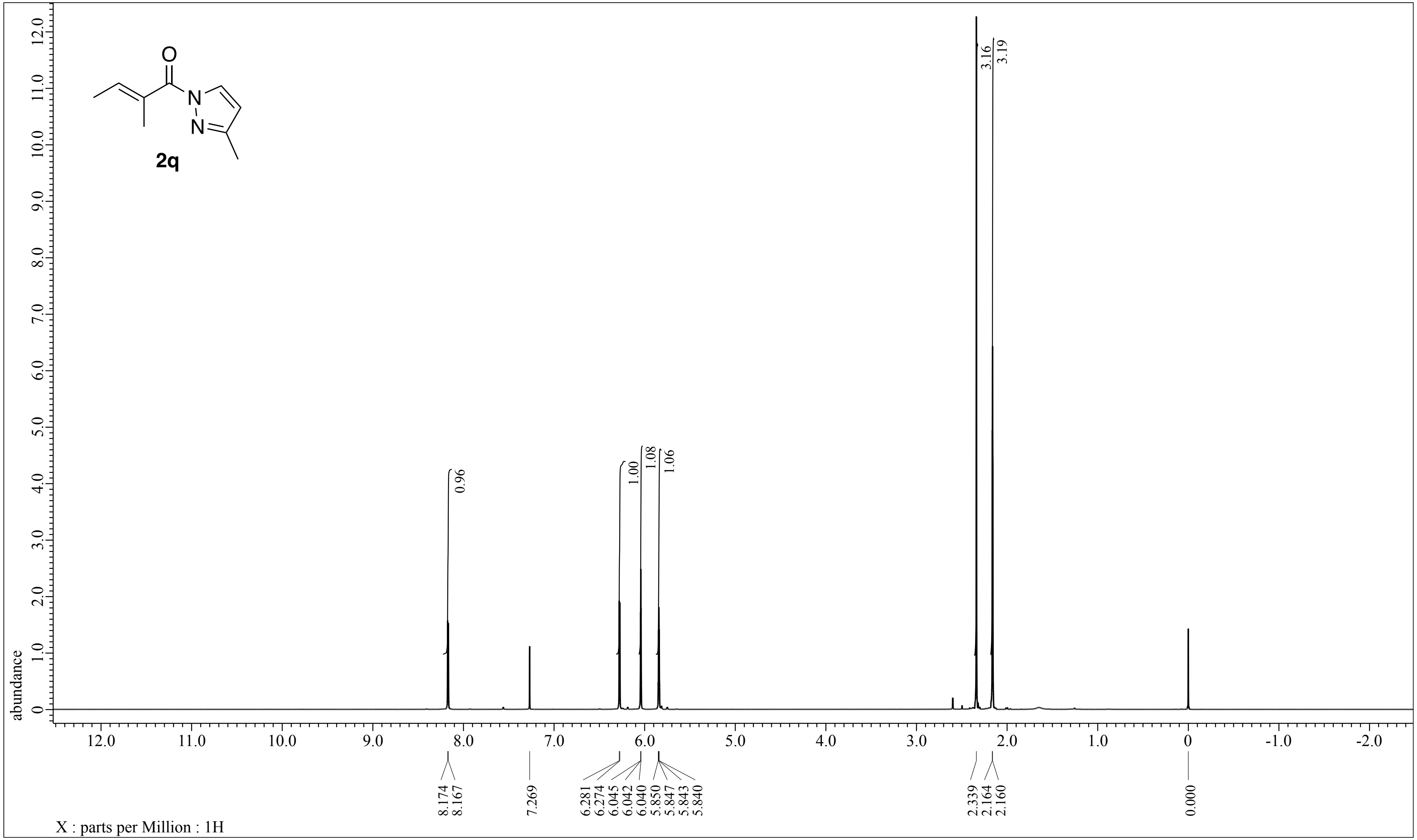
2q
abundance
X : parts per Million : 1H
0.96
1.00
1.08
1.06
3.16
3.19
8.174
8.167
7.269
6.281
6.274
6.045
6.042
6.040
5.850
5.847
5.843
5.840
2.339
2.164
2.160
0.000

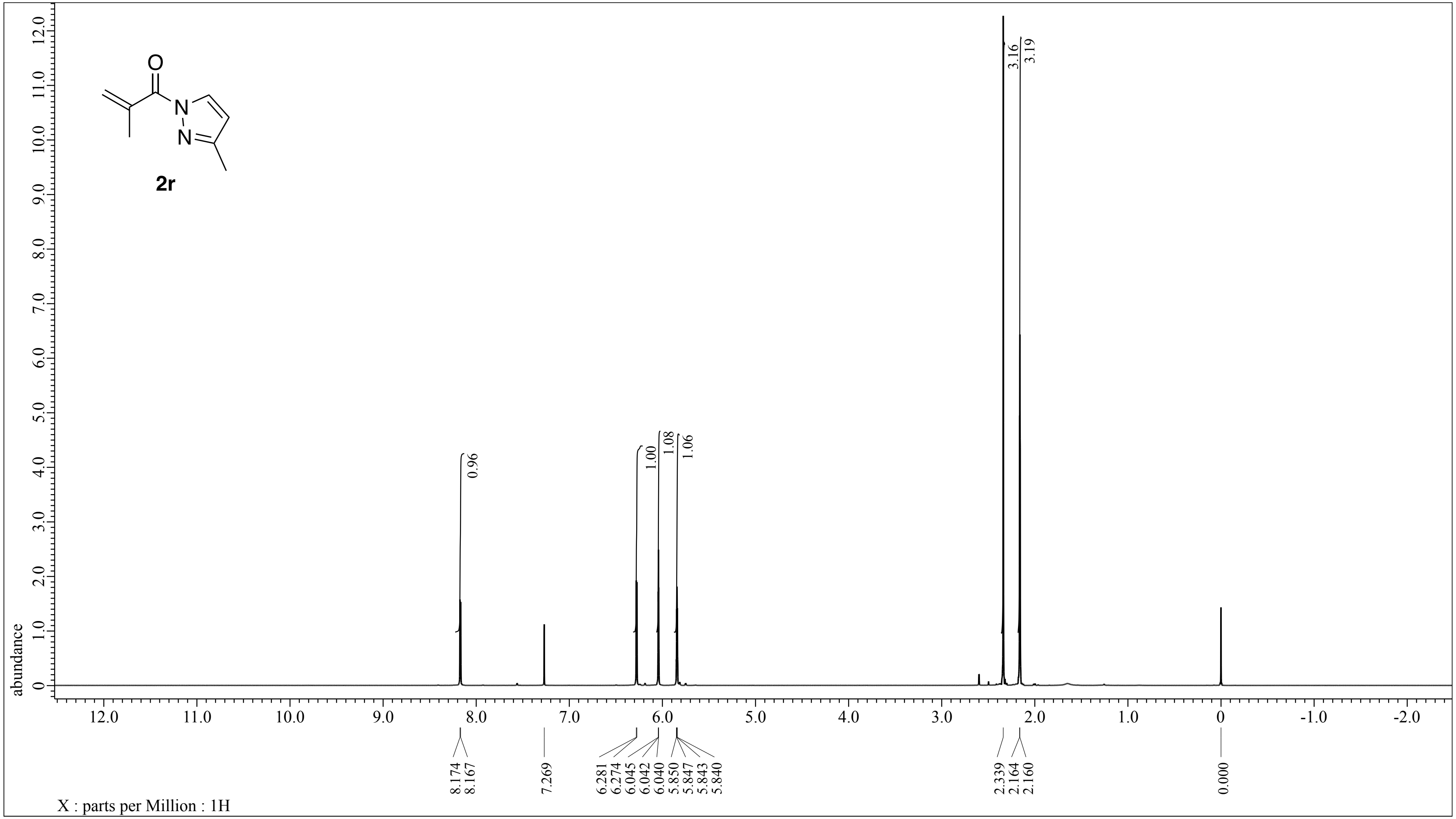
2r
abundance
X : parts per Million : 1H
0.96
1.00
1.08
1.06
3.16
3.19
8.174
8.167
7.269
6.281
6.274
6.045
6.042
6.040
5.850
5.847
5.843
5.840
2.339
2.164
2.160
0.000

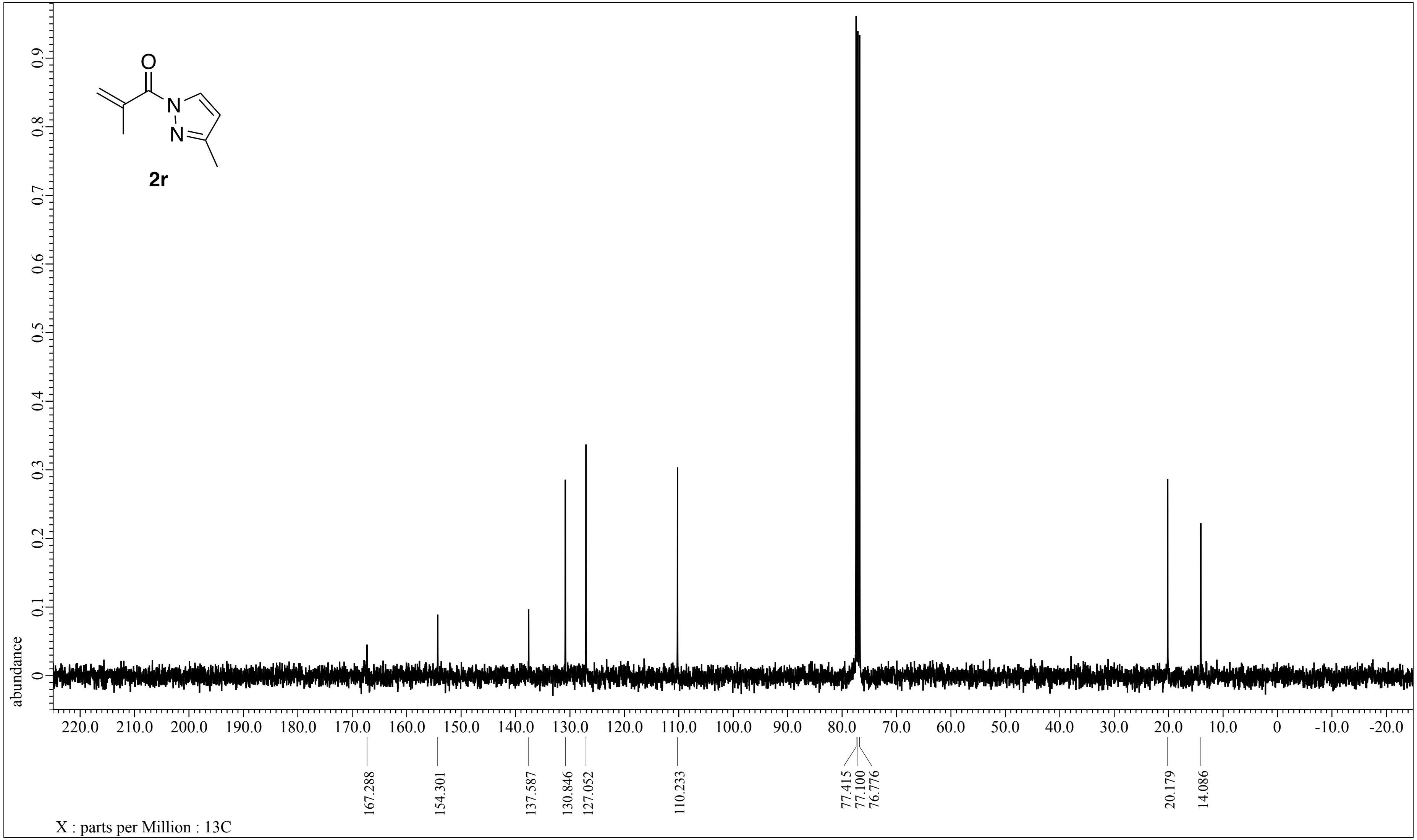

O
N
N
2r
abundance
0
0.1
0.2
0.3
0.4
0.5
0.6
0.7
0.8
0.9
220.0 210.0 200.0 190.0 180.0 170.0 160.0 150.0 140.0 130.0 120.0 110.0 100.0 90.0 80.0 70.0 60.0 50.0 40.0 30.0 20.0 10.0 0 -10.0 -20.0
167.288
154.301
137.587
130.846
127.052
110.233
77.415
77.100
76.776
20.179
14.086
X : parts per Million : 13C

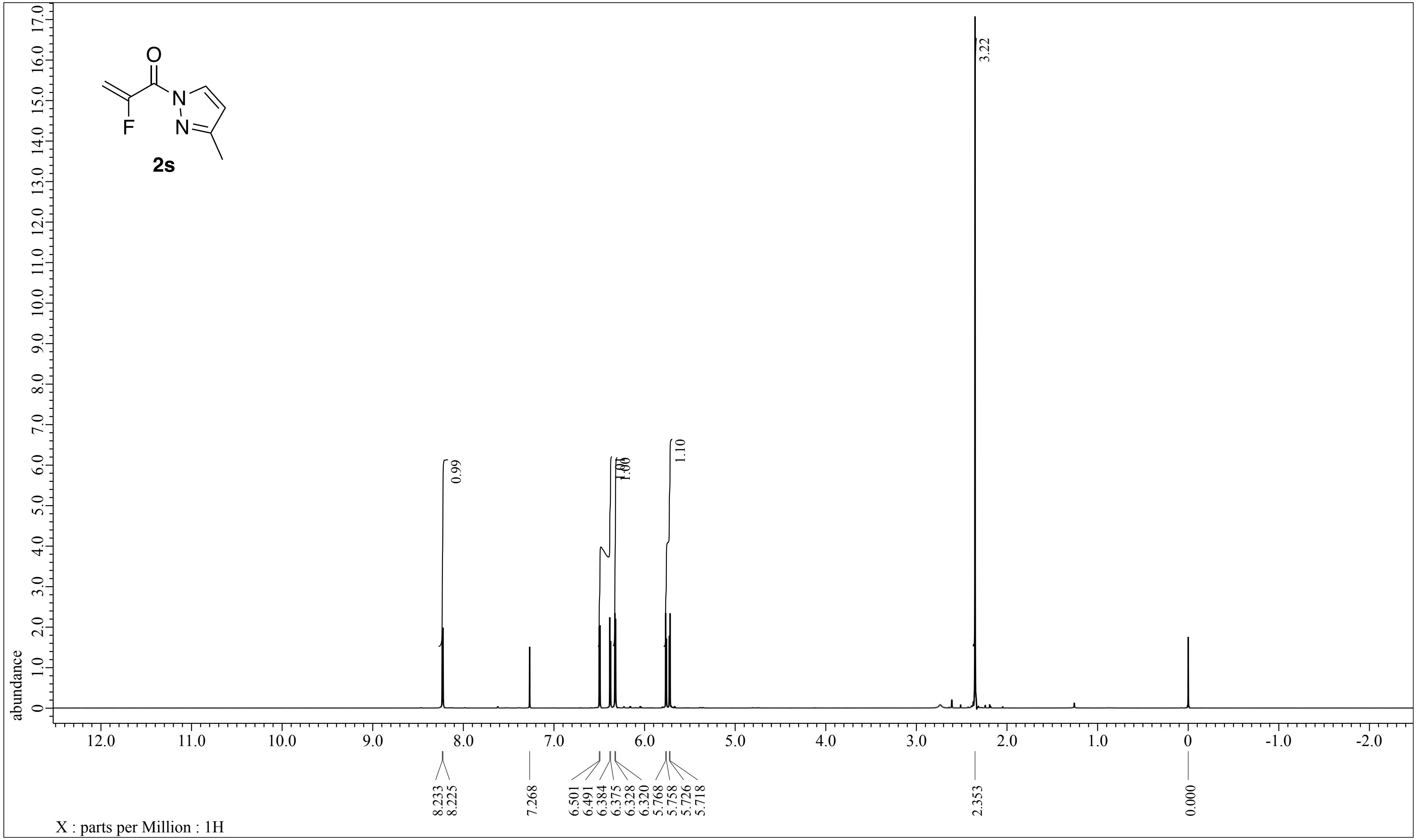

2s
0.99
1.01
1.00
1.10
3.22
abundance
X : parts per Million : 1H
8.233
8.225
7.268
6.501
6.491
6.384
6.375
6.328
6.320
5.768
5.758
5.726
5.718
2.353
0.000

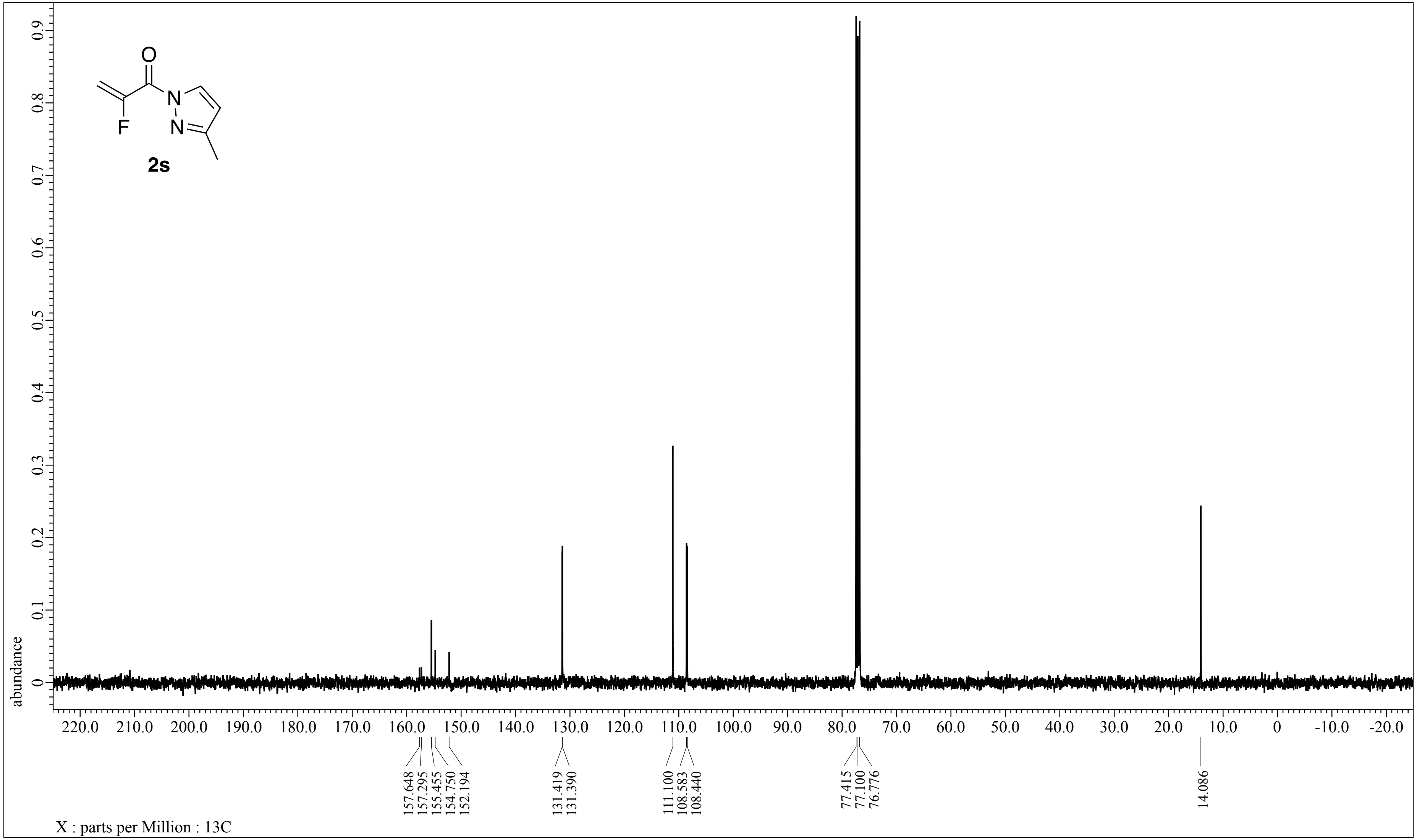

O
N
N
F
2s
abundance
X : parts per Million : 13C
157.648
157.295
155.455
154.750
152.194
131.419
131.390
111.100
108.583
108.440
77.415
77.100
76.776
14.086

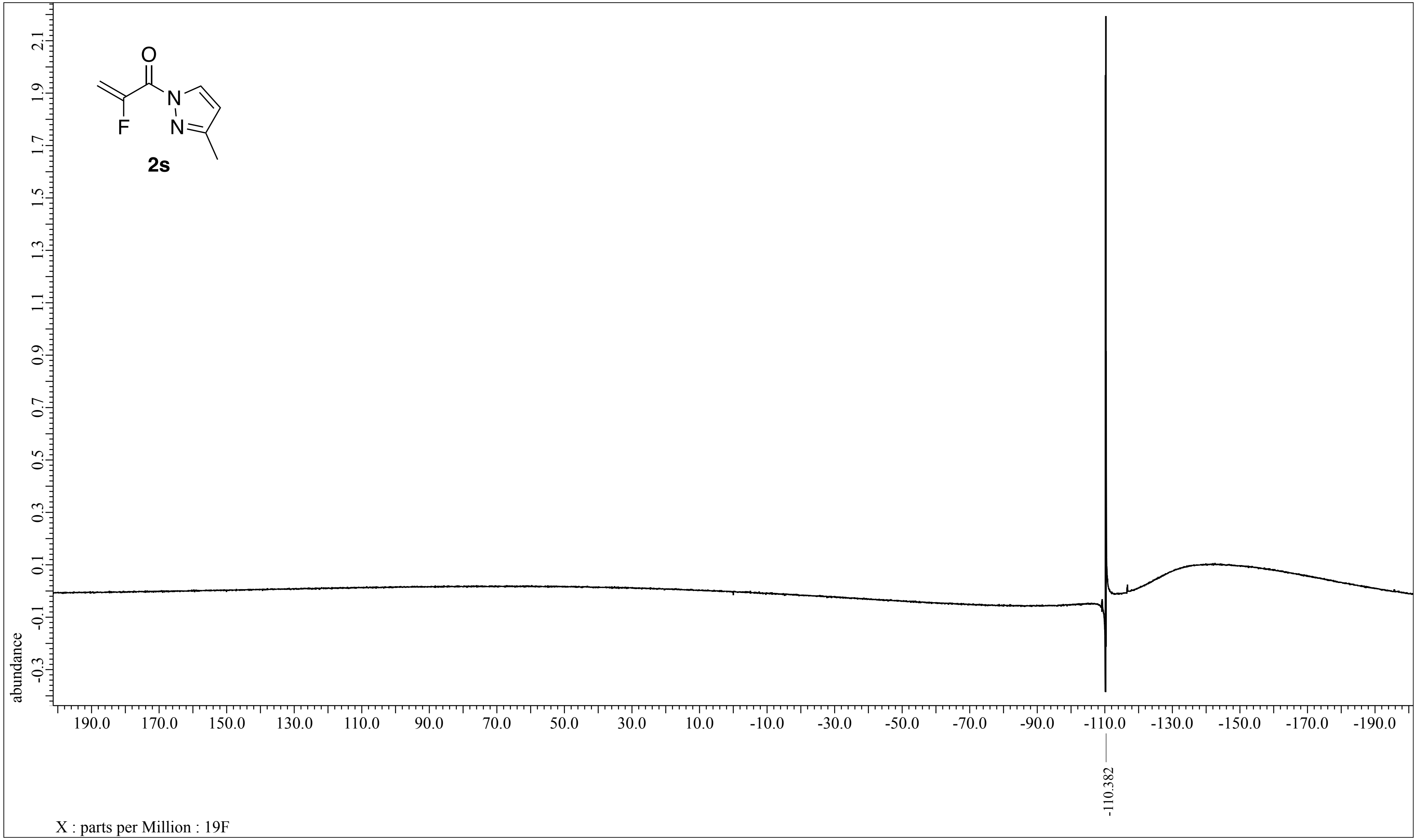
O
N
N
F
2s
abundance
-0.3 -0.1 0.1 0.3 0.5 0.7 0.9 1.1 1.3 1.5 1.7 1.9 2.1
190.0 170.0 150.0 130.0 110.0 90.0 70.0 50.0 30.0 10.0 -10.0 -30.0 -50.0 -70.0 -90.0 -110.0 -130.0 -150.0 -170.0 -190.0
-110.382
X : parts per Million : 19F

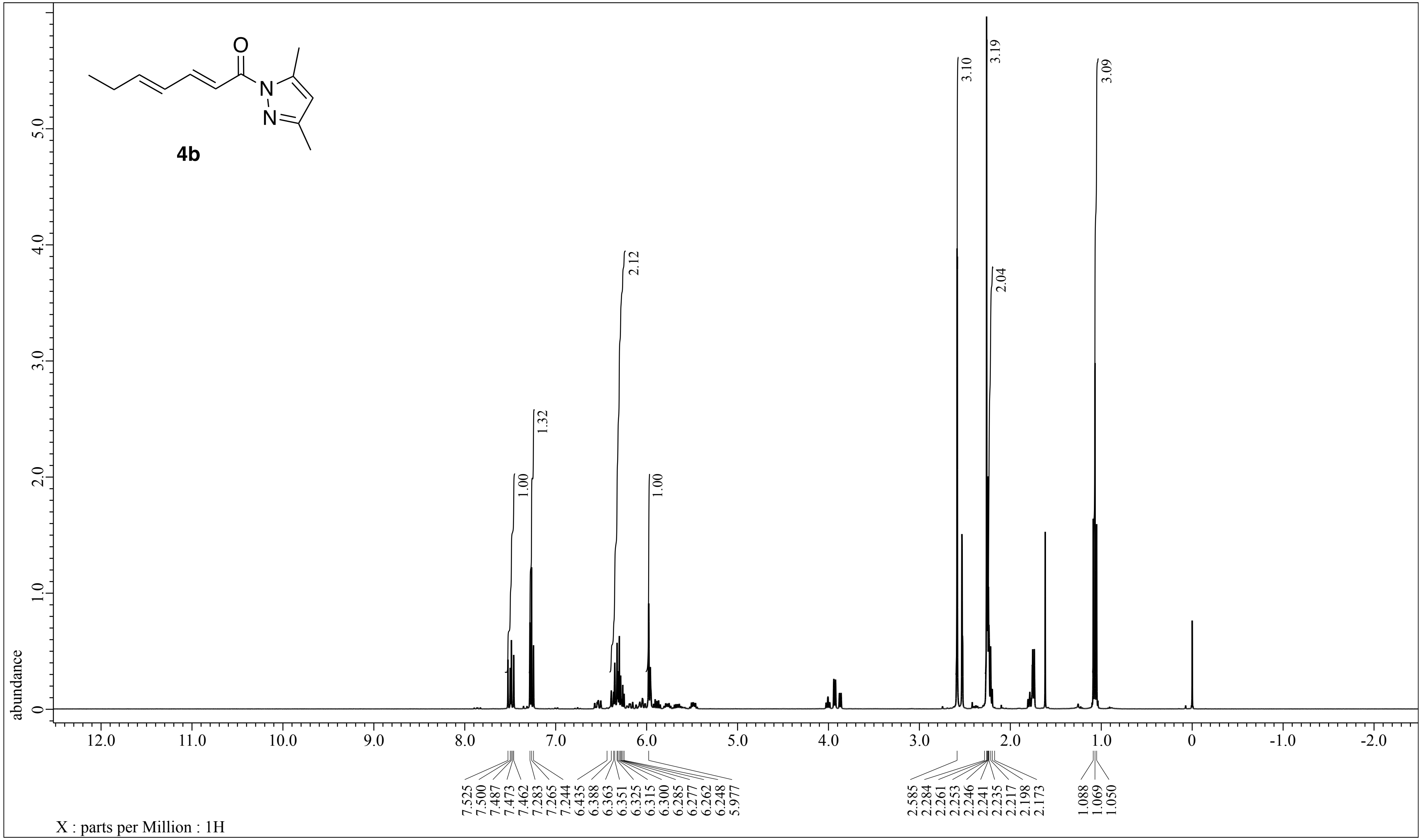

4b
abundance
X : parts per Million : 1H
1.00
1.32
2.12
1.00
3.10
3.19
2.04
3.09
7.525
7.500
7.487
7.473
7.462
7.283
7.265
7.244
6.435
6.388
6.363
6.351
6.325
6.315
6.300
6.285
6.277
6.262
6.248
5.977
2.585
2.284
2.261
2.253
2.246
2.241
2.235
2.217
2.198
2.173
1.088
1.069
1.050

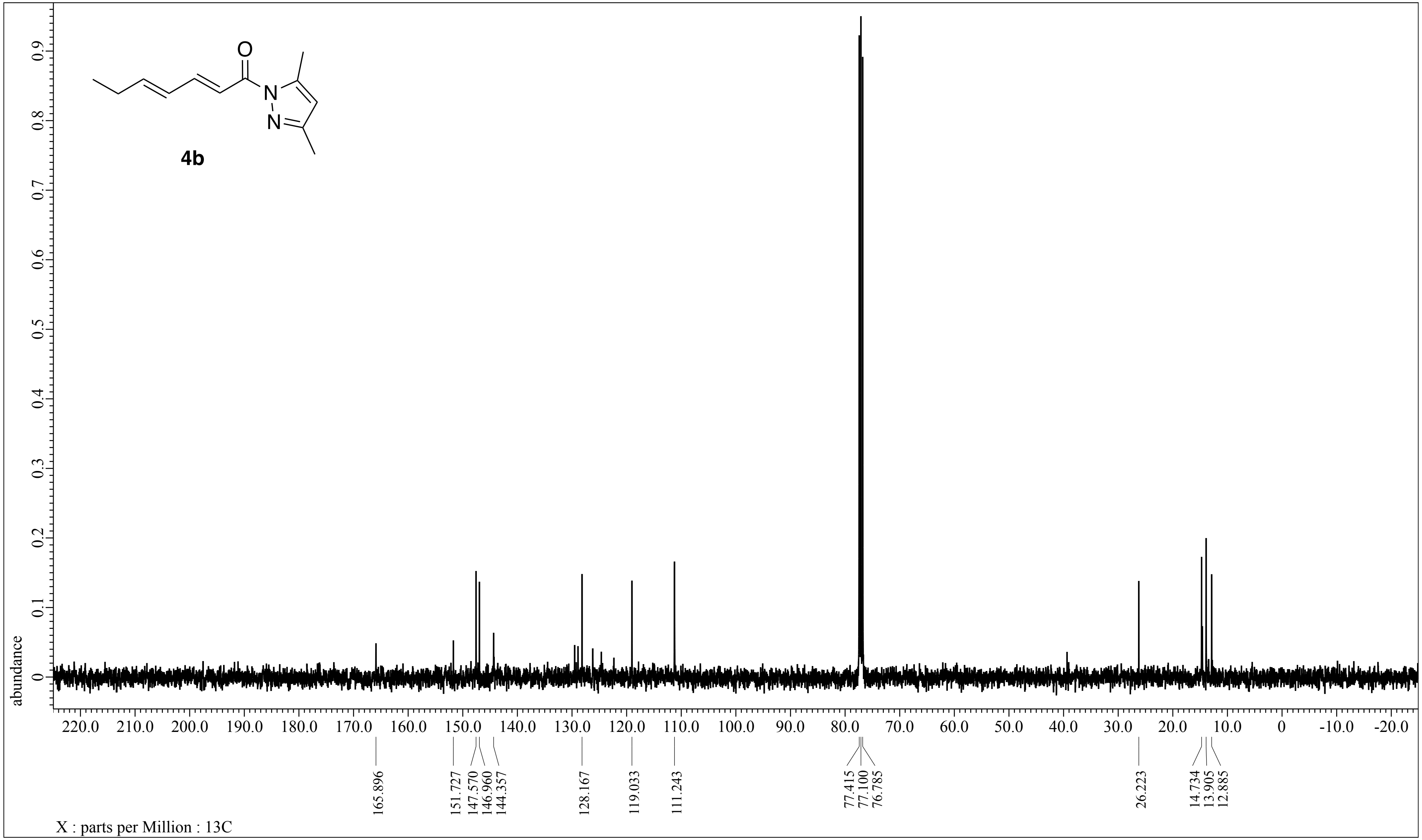
4b
abundance
X : parts per Million : 13C
165.896
151.727
147.570
146.960
144.357
128.167
119.033
111.243
77.415
77.100
76.785
26.223
14.734
13.905
12.885

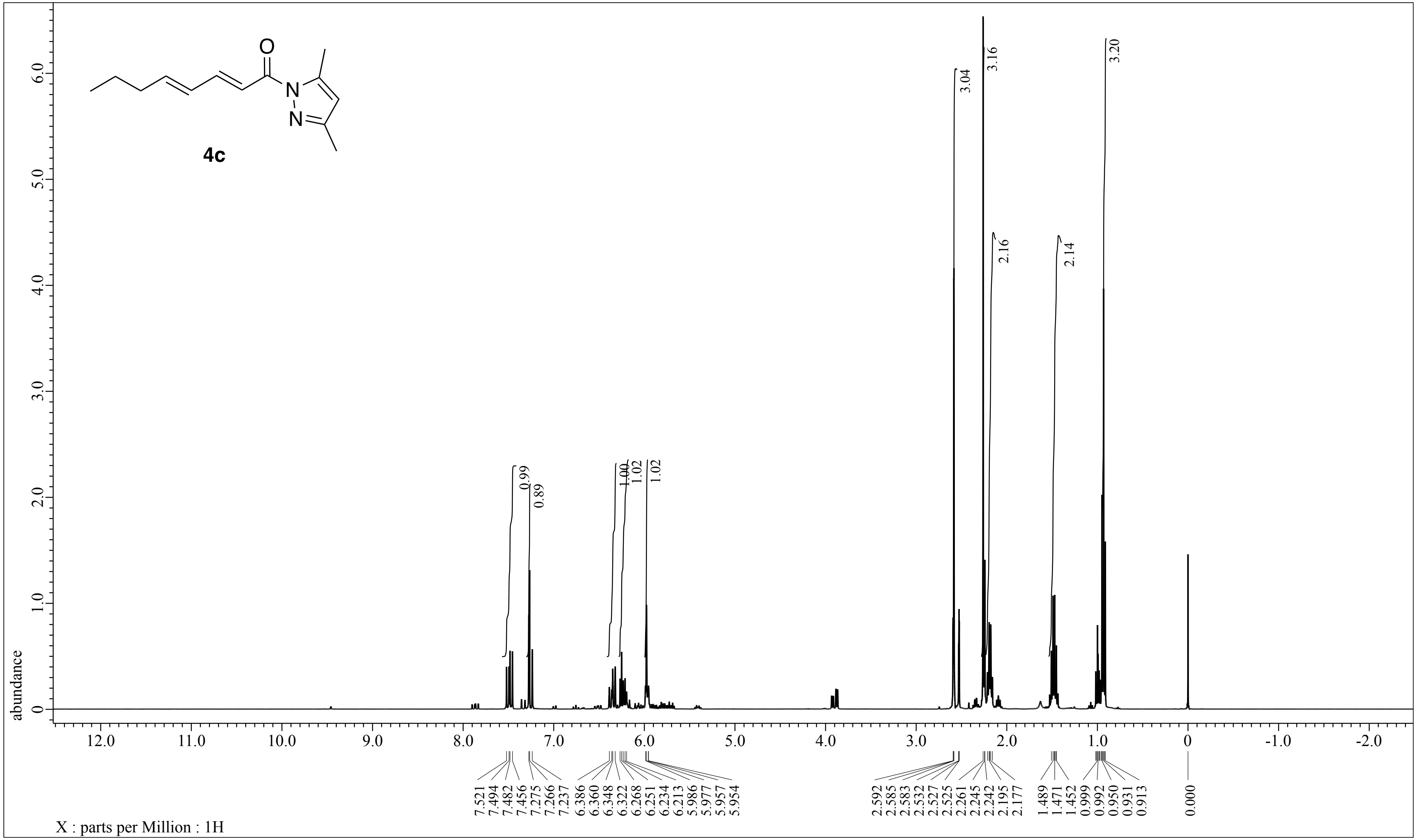

4c
O
N
N
abundance
X : parts per Million : 1H
3.04
3.16
3.20
2.16
2.14
0.99
0.89
1.00
1.02
1.02
7.521
7.494
7.482
7.456
7.275
7.266
7.237
6.386
6.360
6.348
6.322
6.268
6.251
6.234
6.213
5.986
5.977
5.957
5.954
2.592
2.585
2.583
2.532
2.527
2.525
2.261
2.245
2.242
2.195
2.177
1.489
1.471
1.452
0.999
0.992
0.950
0.931
0.913
0.000

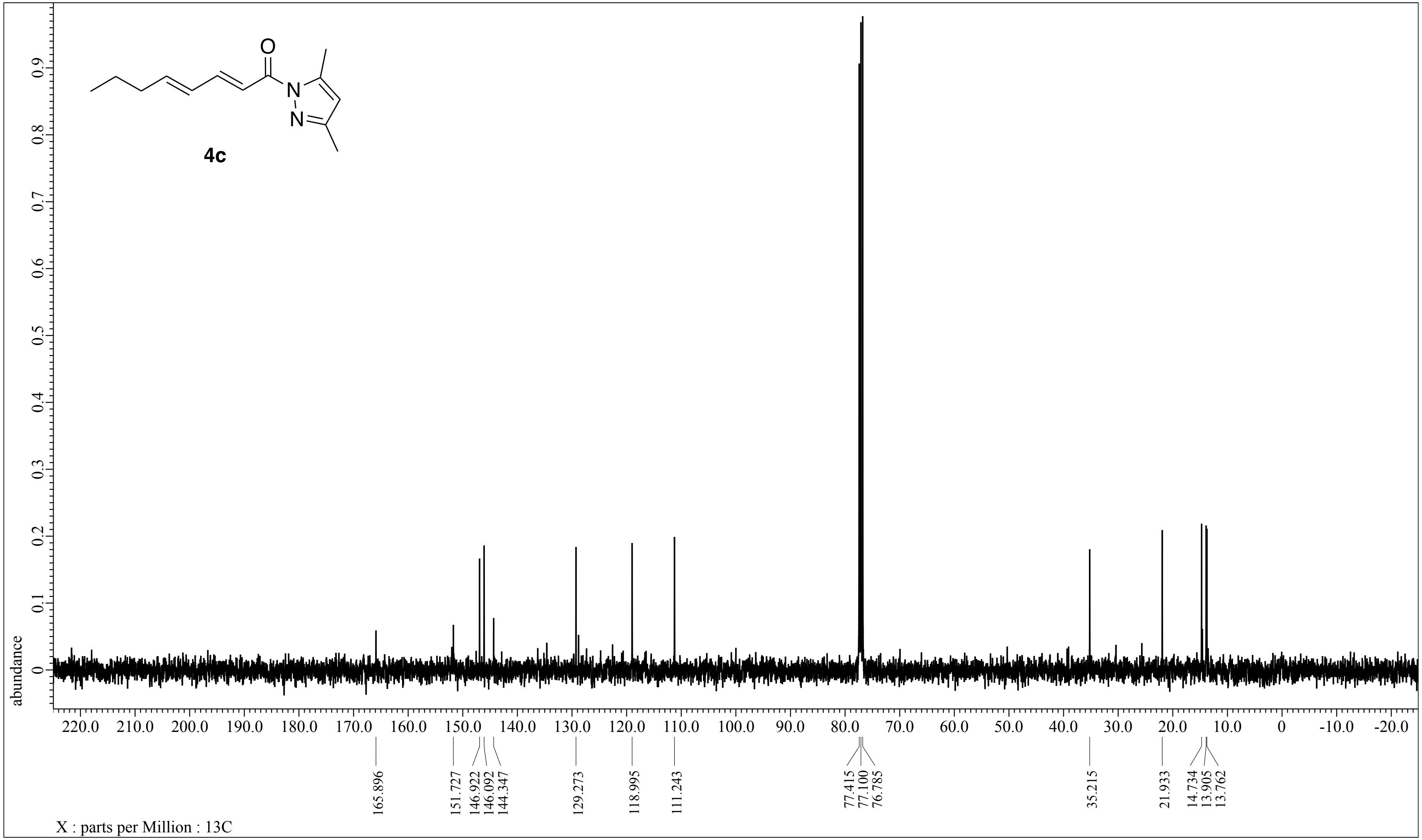
O
N
N
4c
abundance
0
0.1
0.2
0.3
0.4
0.5
0.6
0.7
0.8
0.9
220.0 210.0 200.0 190.0 180.0 170.0 160.0 150.0 140.0 130.0 120.0 110.0 100.0 90.0 80.0 70.0 60.0 50.0 40.0 30.0 20.0 10.0 0 -10.0 -20.0
165.896
151.727
146.922
146.092
144.347
129.273
118.995
111.243
77.415
77.100
76.785
35.215
21.933
14.734
13.905
13.762
X : parts per Million : 13C

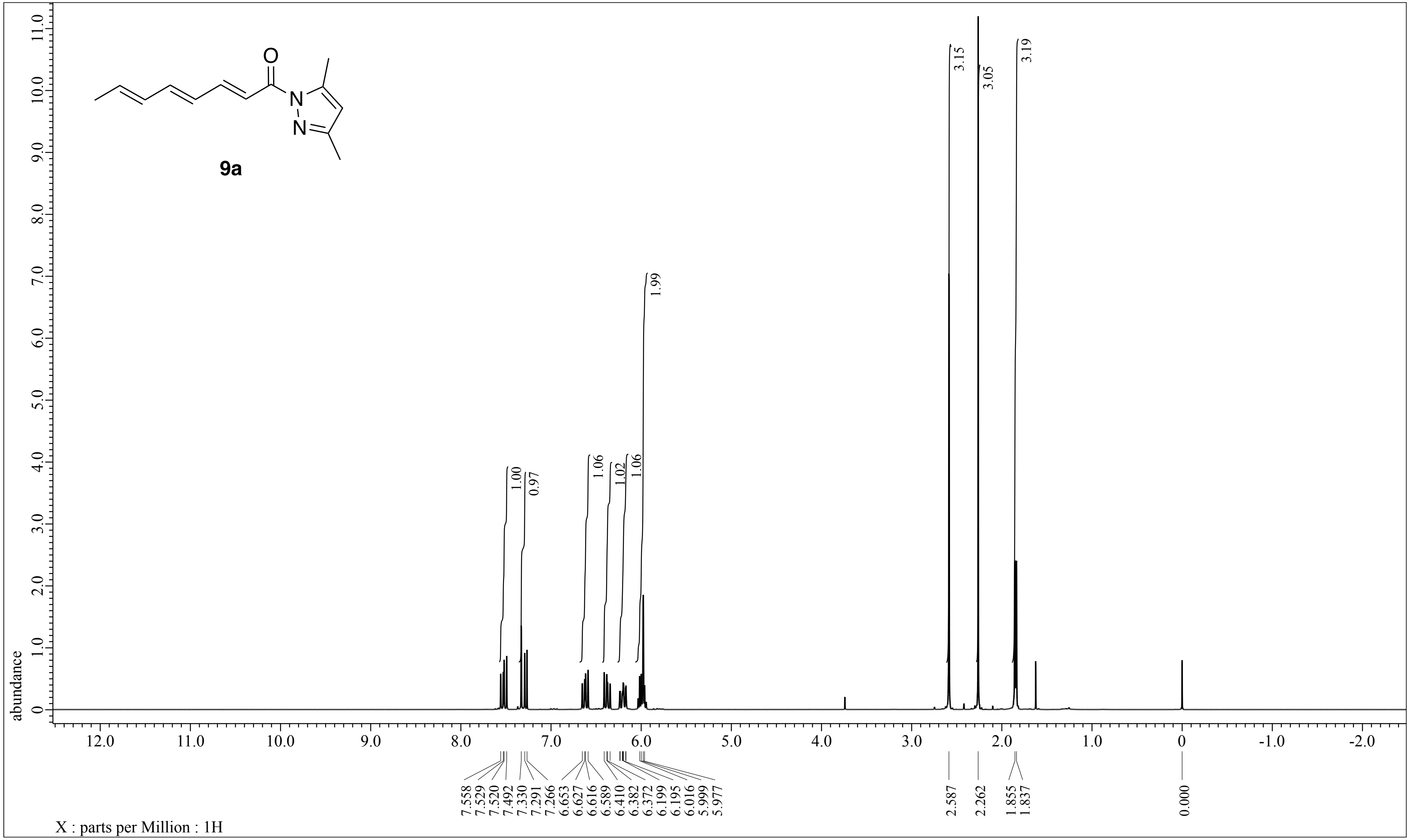
9a
X : parts per Million : 1H
abundance
7.558
7.529
7.520
7.492
7.330
7.291
7.266
6.653
6.627
6.616
6.589
6.410
6.382
6.372
6.199
6.195
6.016
5.999
5.977
2.587
2.262
1.855
1.837
0.000
1.00
0.97
1.06
1.02
1.06
1.99
3.15
3.05
3.19

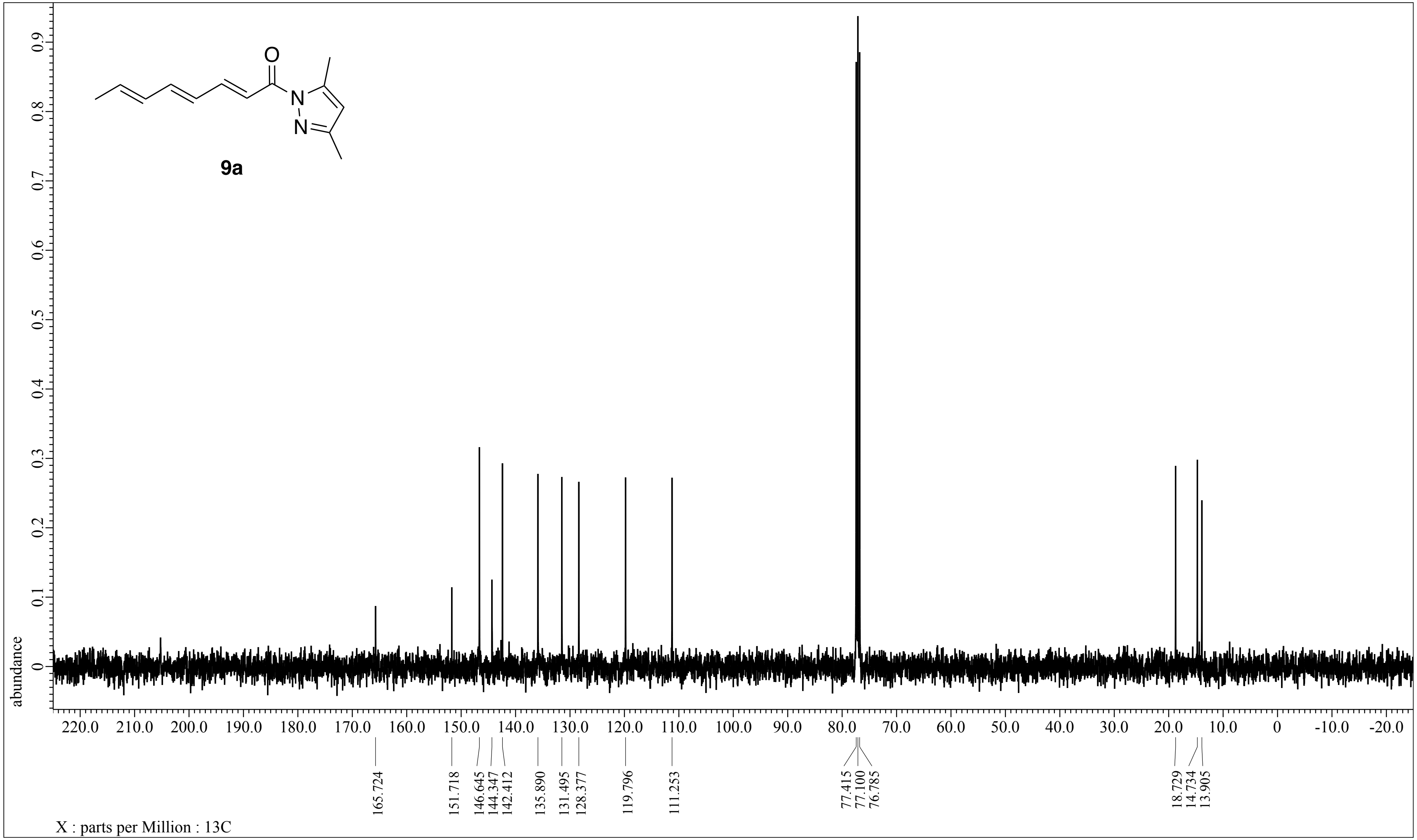

O
N
N
9a
abundance
0.9
0.8
0.7
0.6
0.5
0.4
0.3
0.2
0.1
0
220.0 210.0 200.0 190.0 180.0 170.0 160.0 150.0 140.0 130.0 120.0 110.0 100.0 90.0 80.0 70.0 60.0 50.0 40.0 30.0 20.0 10.0 0 -10.0 -20.0
165.724
151.718
146.645
144.347
142.412
135.890
131.495
128.377
119.796
111.253
77.415
77.100
76.785
18.729
14.734
13.905
X : parts per Million : 13C

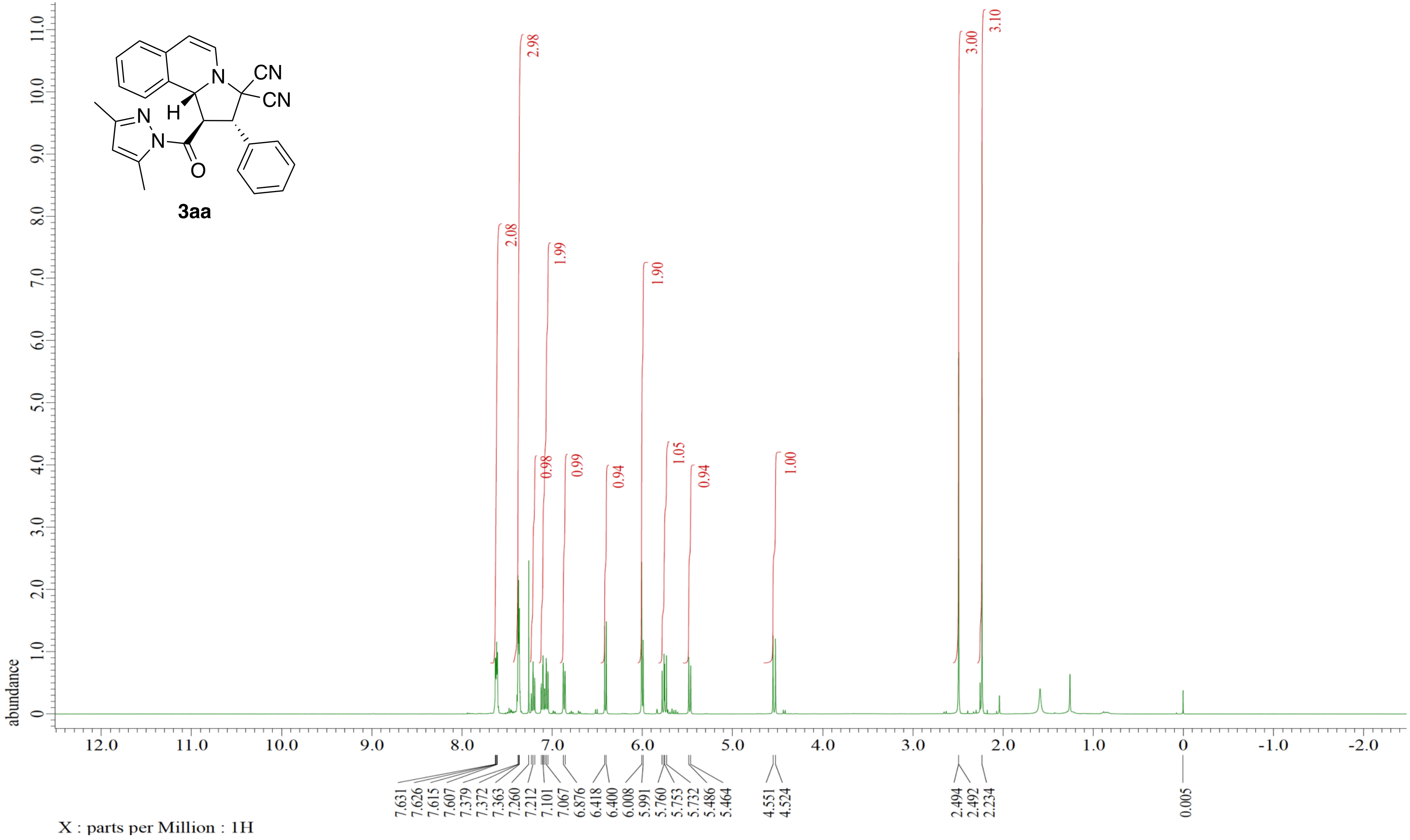
3aa
CN
CN
N
H
N
N
O
abundance
X : parts per Million : 1H
2.08
2.98
0.98
1.99
0.99
0.94
1.90
1.05
0.94
1.00
3.00
3.10
7.631
7.626
7.615
7.607
7.379
7.372
7.363
7.260
7.212
7.101
7.067
6.876
6.418
6.400
6.008
5.991
5.760
5.753
5.732
5.486
5.464
4.551
4.524
2.494
2.492
2.234
0.005

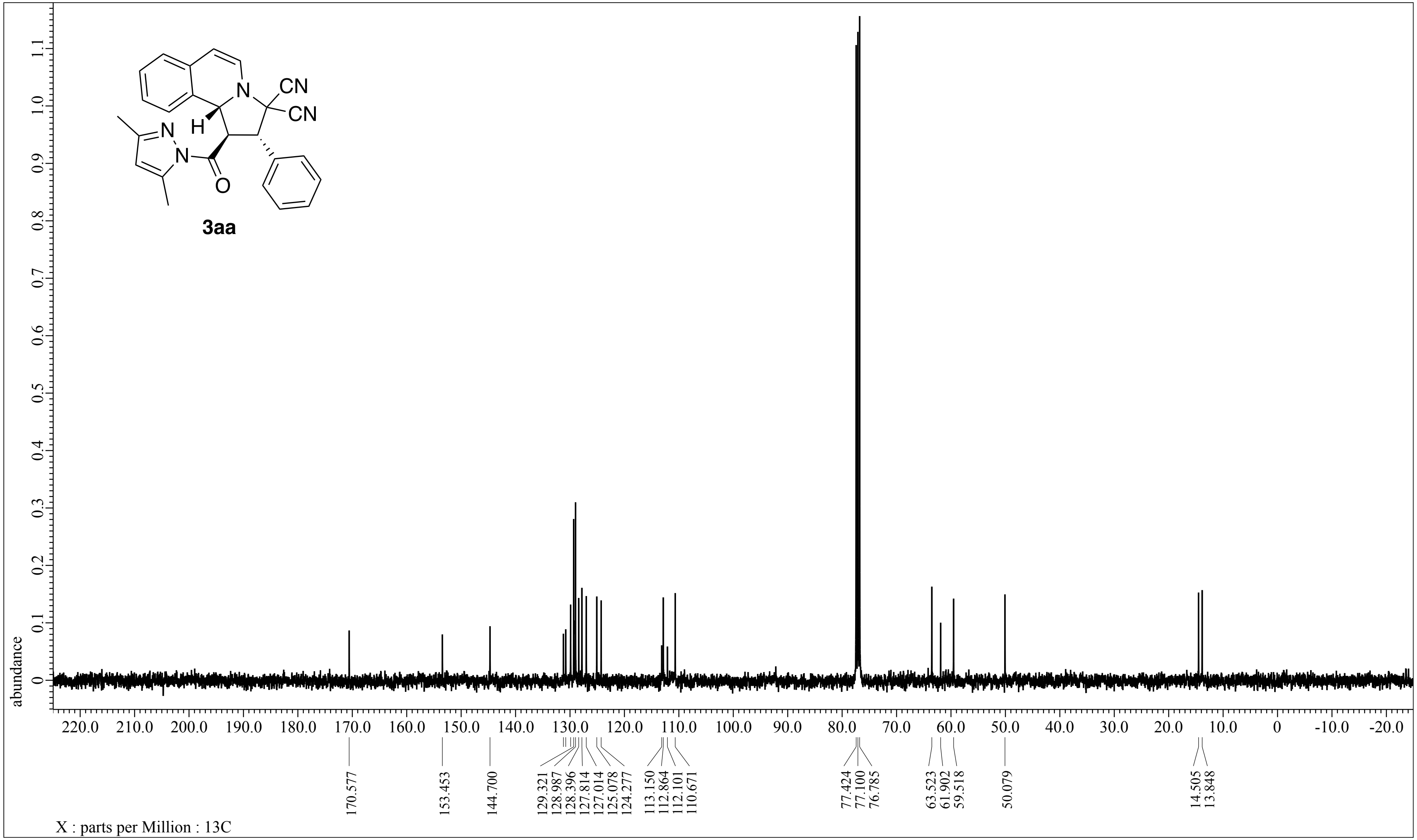
3aa
abundance
X : parts per Million : 13C
170.577
153.453
144.700
129.321
128.987
128.396
127.814
127.014
125.078
124.277
113.150
112.864
112.101
110.671
77.424
77.100
76.785
63.523
61.902
59.518
50.079
14.505
13.848

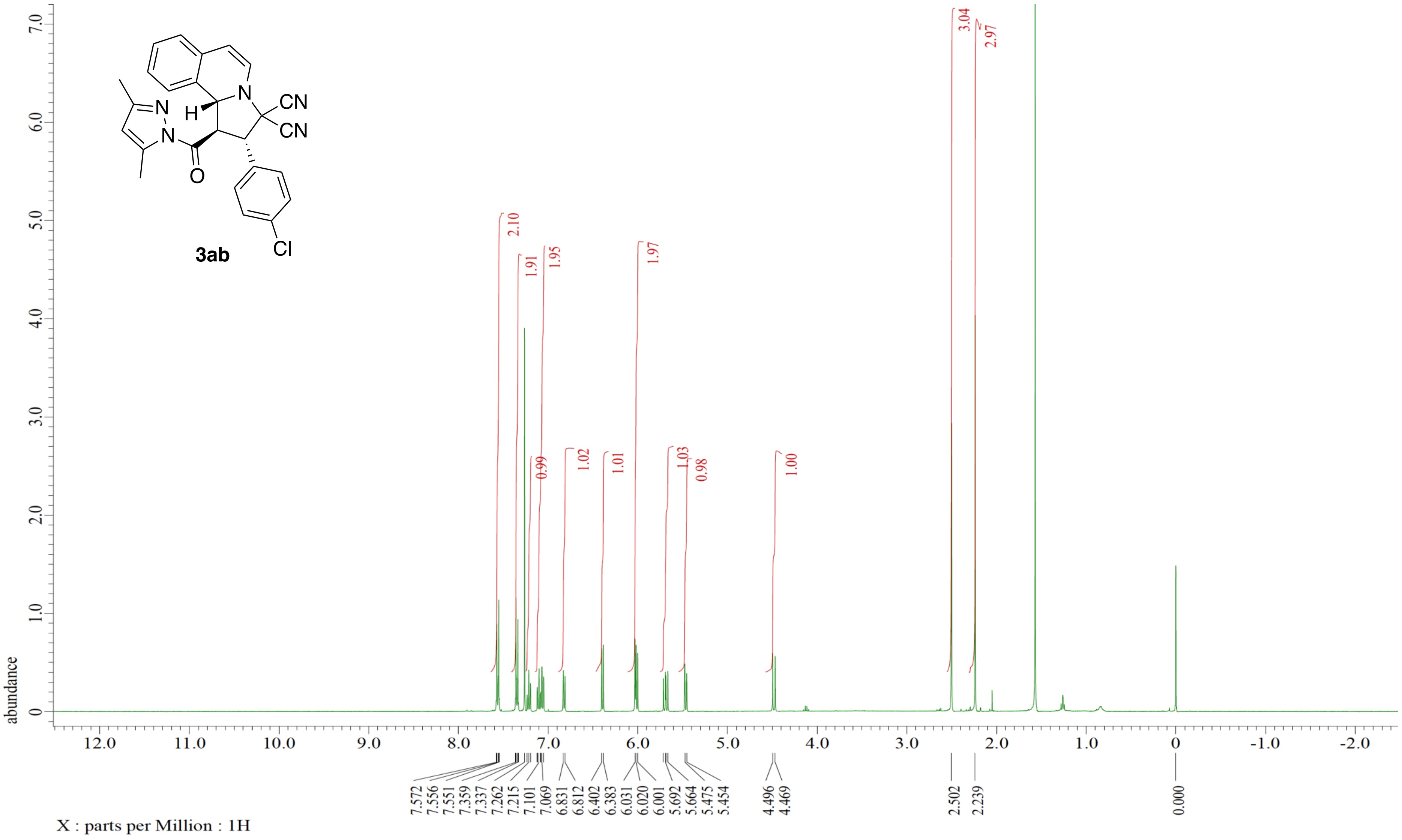
3ab
abundance
X : parts per Million : 1H
7.572
7.556
7.551
7.359
7.337
7.262
7.215
7.101
7.069
6.831
6.812
6.402
6.383
6.031
6.020
6.001
5.692
5.664
5.475
5.454
4.496
4.469
2.502
2.239
0.000
2.10
1.91
1.95
0.99
1.02
1.01
1.97
1.03
0.98
1.00
3.04
2.97

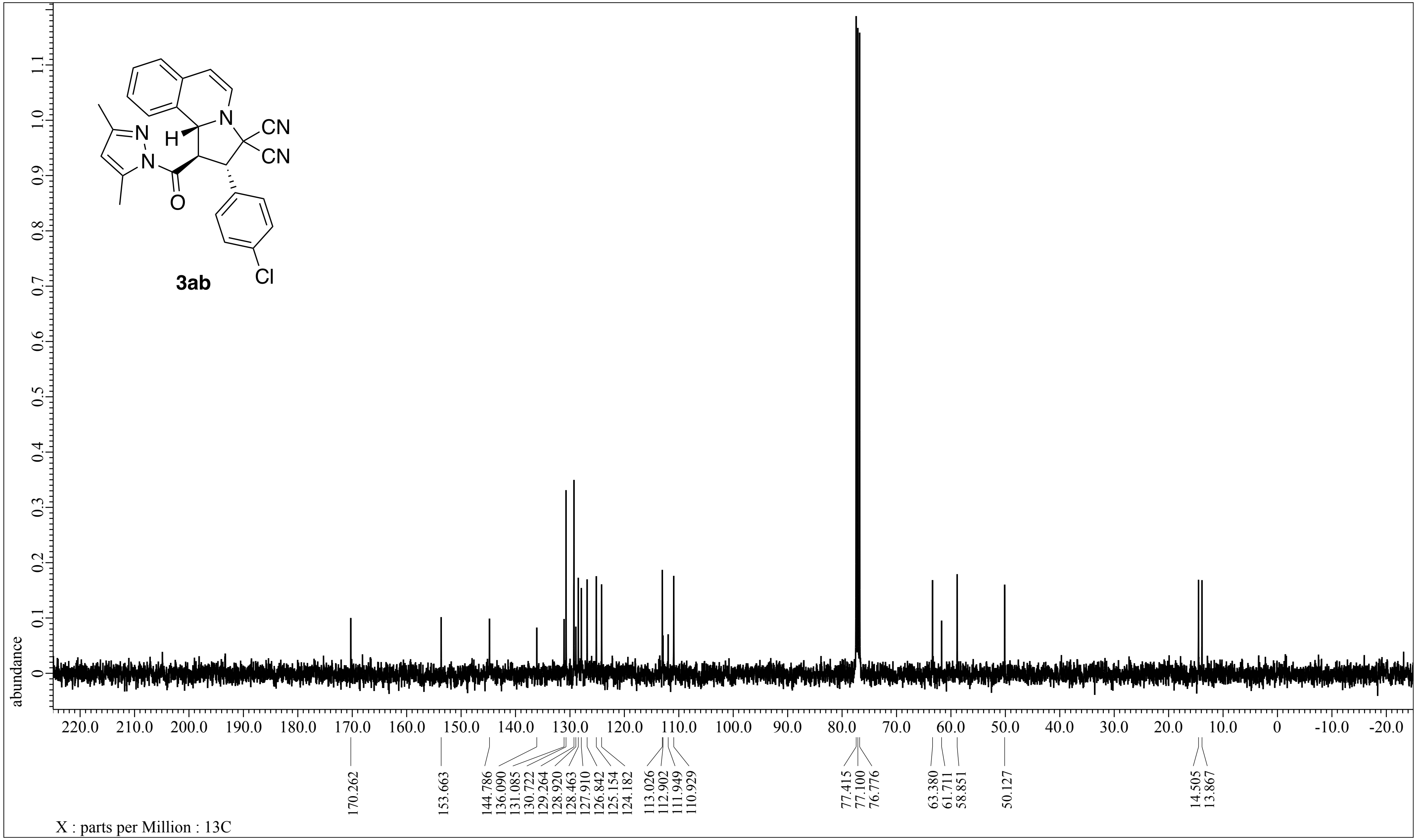
N
N
H
N
CN
CN
O
Cl
3ab
abundance
X : parts per Million : 13C
170.262
153.663
144.786
136.090
131.085
130.722
129.264
128.920
128.463
127.910
126.842
125.154
124.182
113.026
112.902
111.949
110.929
77.415
77.100
76.776
63.380
61.711
58.851
50.127
14.505
13.867

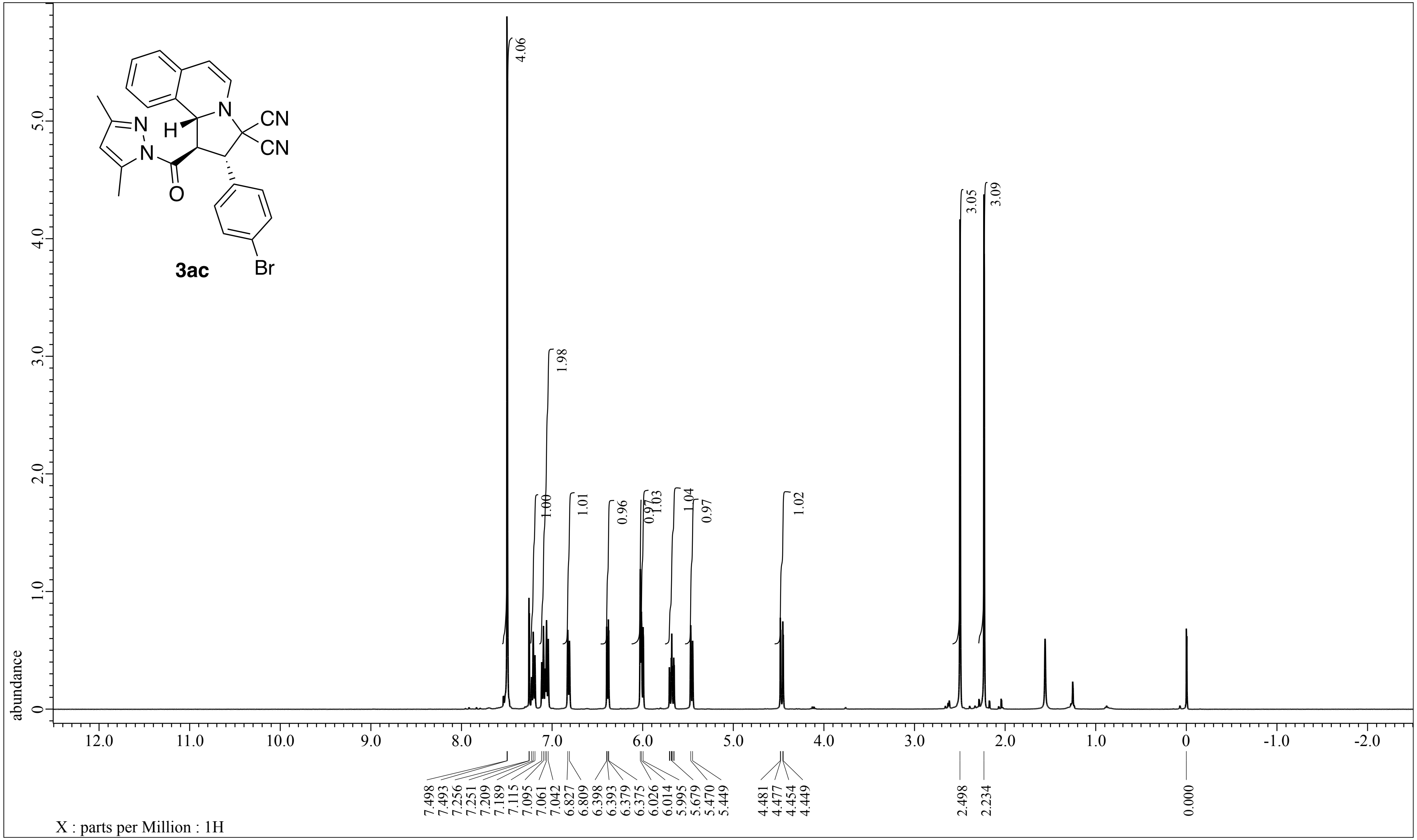
3ac
CN
CN
N
N
N
H
O
Br
4.06
1.98
1.00
1.01
0.96
0.97
1.03
1.04
0.97
1.02
3.05
3.09
abundance
X : parts per Million : 1H
7.498
7.493
7.256
7.251
7.209
7.189
7.115
7.095
7.061
7.042
6.827
6.809
6.398
6.393
6.379
6.375
6.026
6.014
5.995
5.679
5.470
5.449
4.481
4.477
4.454
4.449
2.498
2.234
0.000

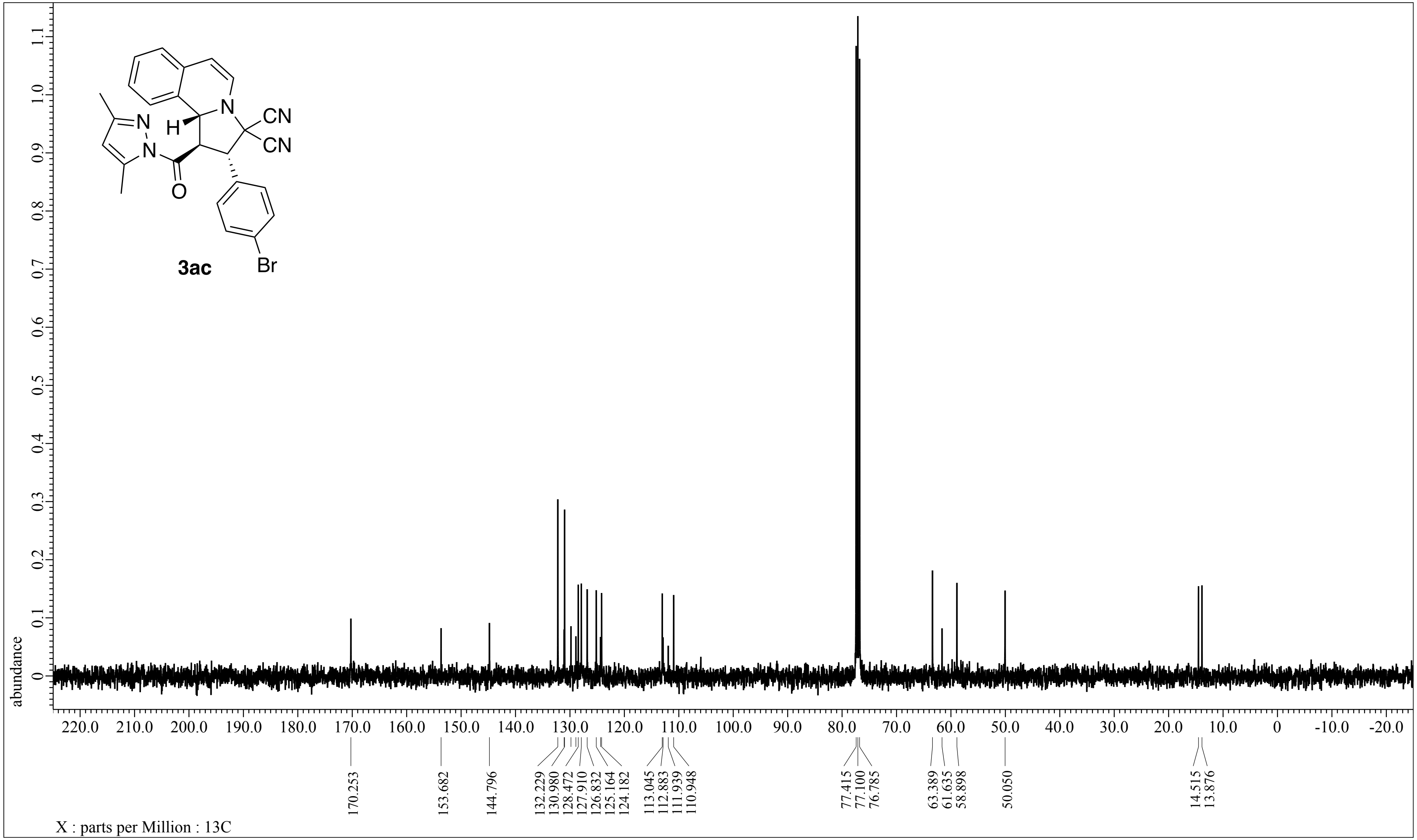

3ac
CN
CN
N
N
N
H
O
Br
abundance
X : parts per Million : 13C
170.253
153.682
144.796
132.229
130.980
128.472
127.910
126.832
125.164
124.182
113.045
112.883
111.939
110.948
77.415
77.100
76.785
63.389
61.635
58.898
50.050
14.515
13.876

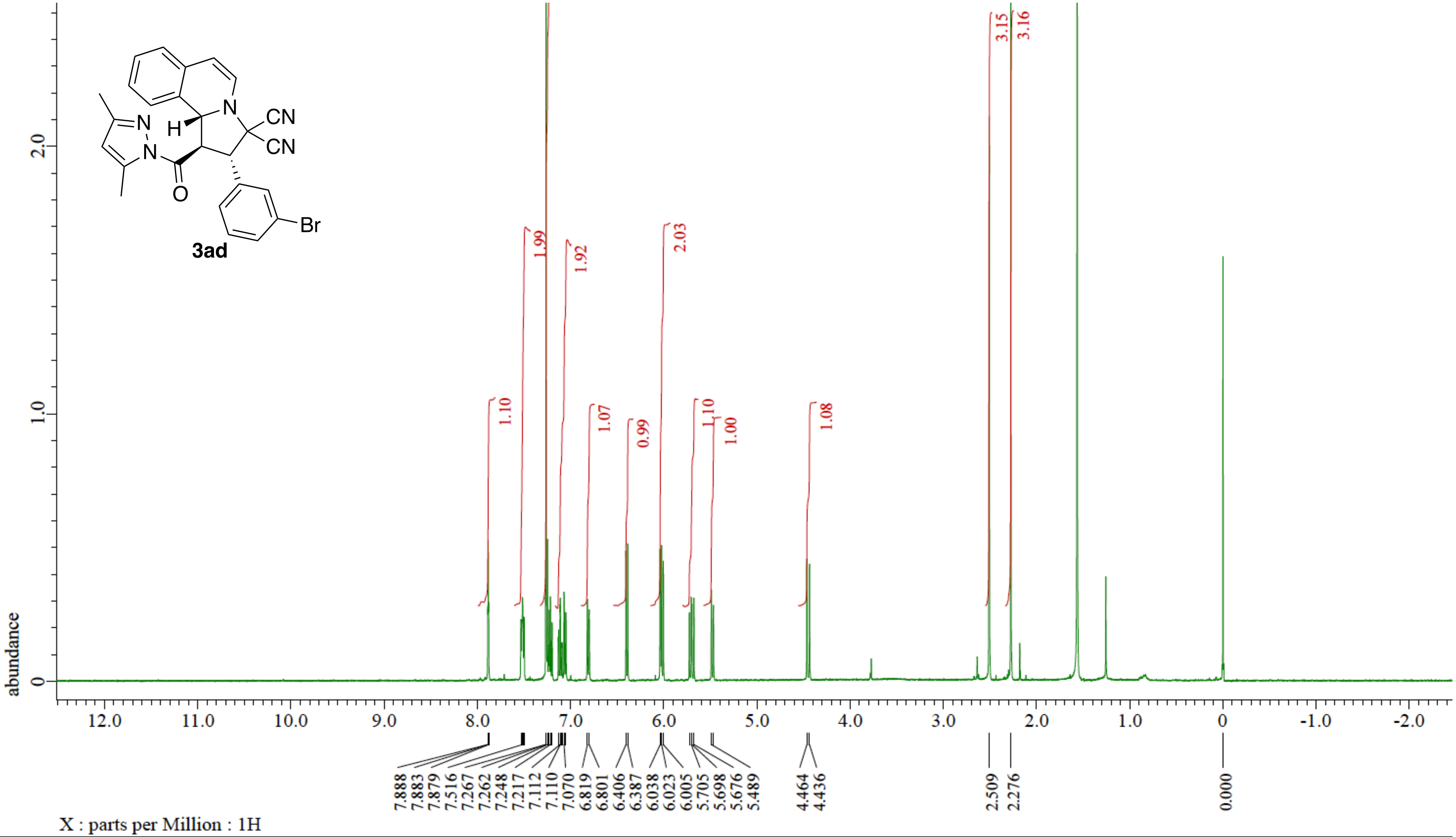
3ad
abundance
X : parts per Million : 1H
7.888
7.883
7.879
7.516
7.267
7.262
7.248
7.217
7.112
7.110
7.070
6.819
6.801
6.406
6.387
6.038
6.023
6.005
5.705
5.698
5.676
5.489
4.464
4.436
2.509
2.276
0.000
1.10
1.99
1.92
1.07
0.99
2.03
1.10
1.00
1.08
3.15
3.16

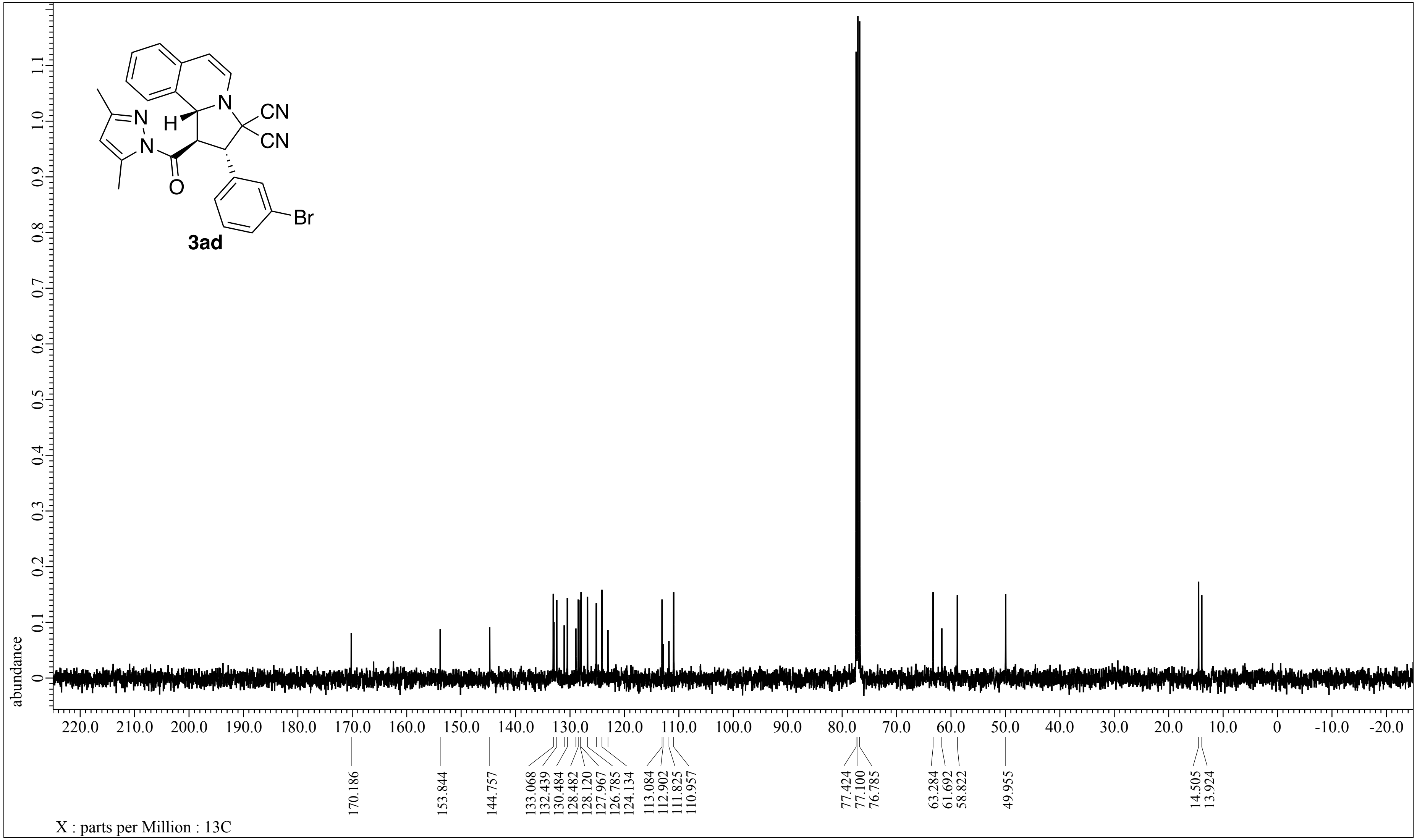
3ad
CN
CN
Br
abundance
X : parts per Million : 13C
170.186
153.844
144.757
133.068
132.439
130.484
128.482
128.120
127.967
126.785
124.134
113.084
112.902
111.825
110.957
77.424
77.100
76.785
63.284
61.692
58.822
49.955
14.505
13.924

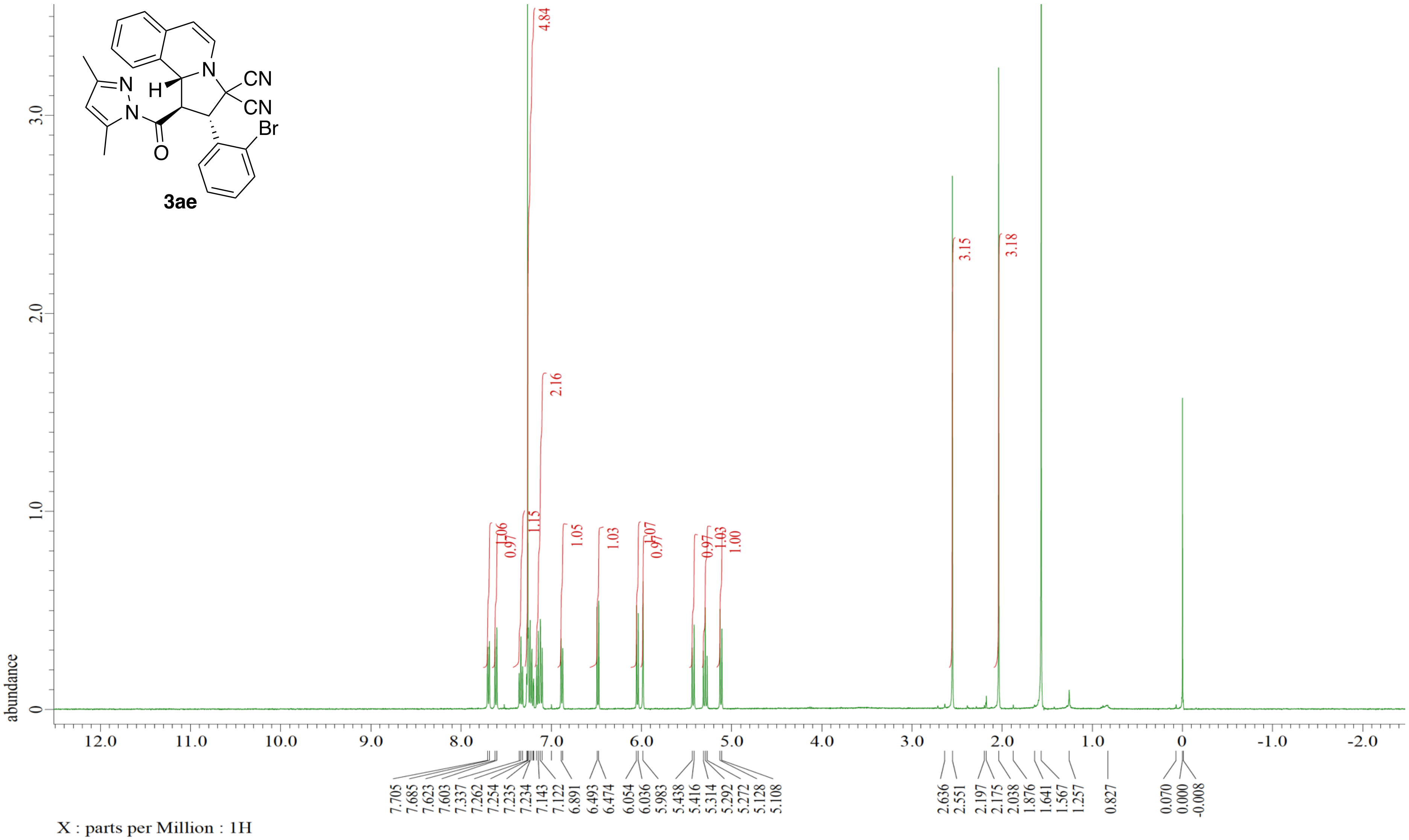
3ae
abundance
X : parts per Million : 1H

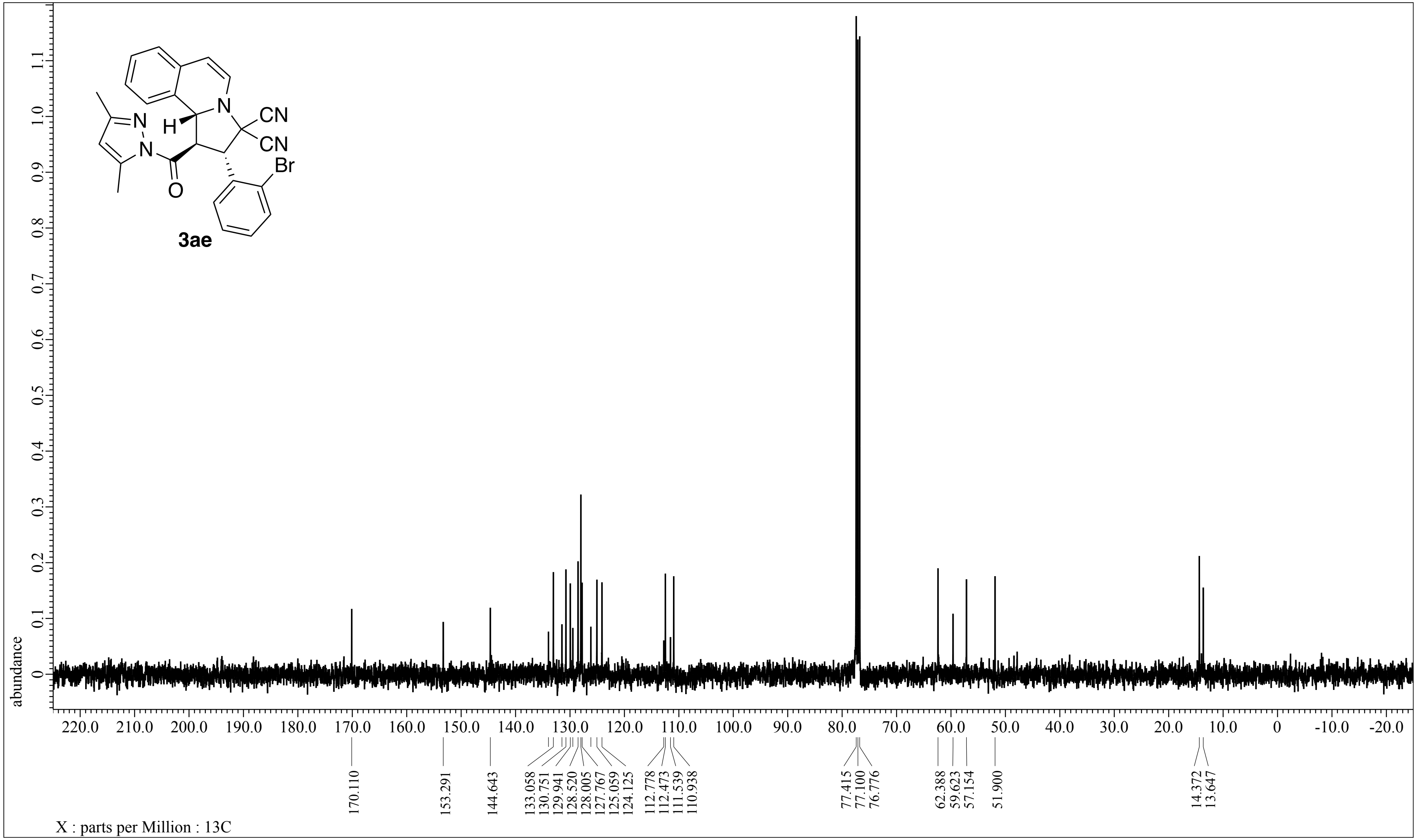
3ae
abundance
X : parts per Million : 13C
170.110
153.291
144.643
133.058
130.751
129.941
128.520
128.005
127.767
125.059
124.125
112.778
112.473
111.539
110.938
77.415
77.100
76.776
62.388
59.623
57.154
51.900
14.372
13.647

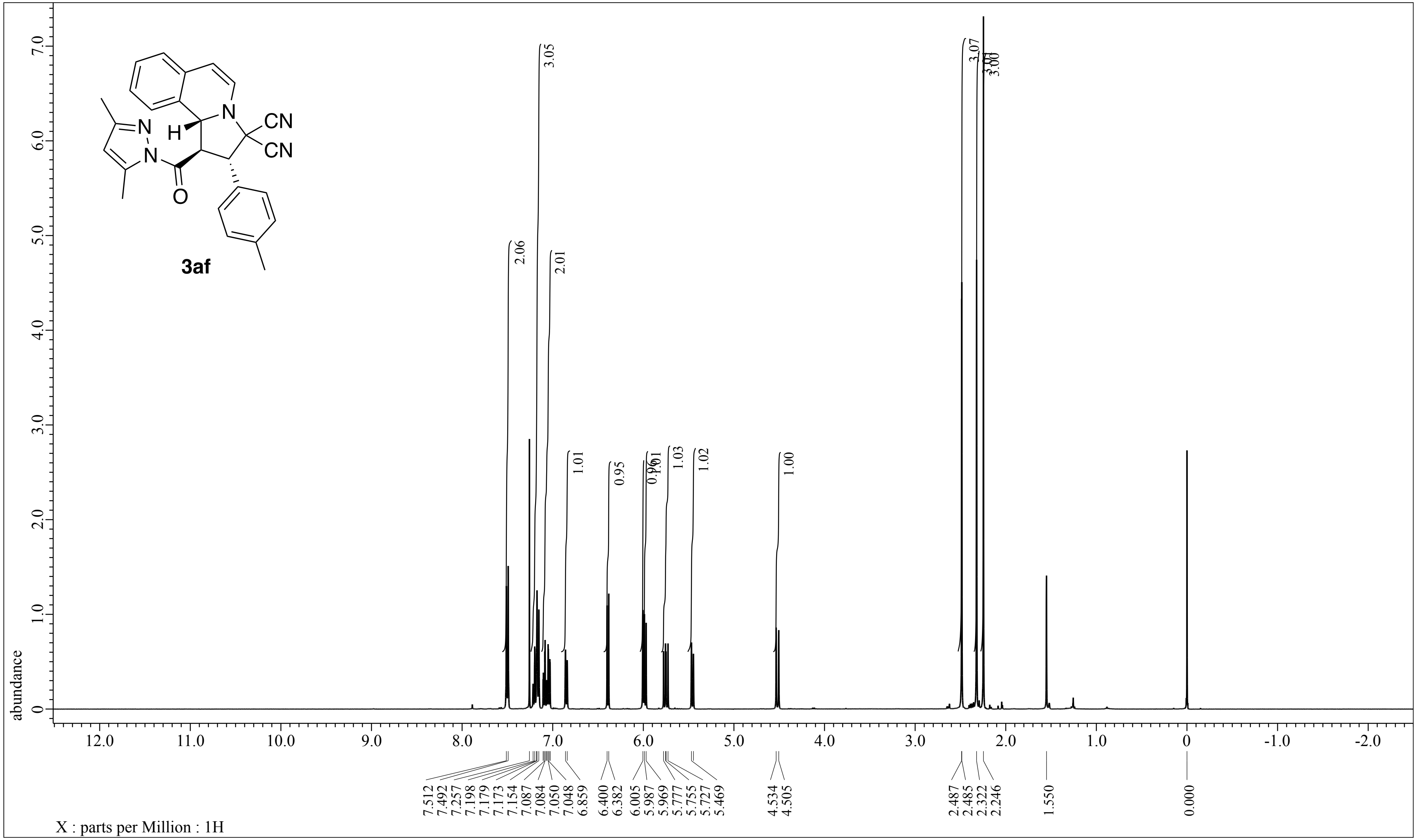
N
N
H
N
CN
CN
O
3af
7.0
6.0
5.0
4.0
3.0
2.0
1.0
0
abundance
3.05
2.06
2.01
1.01
0.95
0.96
1.01
1.03
1.02
1.00
3.07
3.01
3.00
12.0
11.0
10.0
9.0
8.0
7.0
6.0
5.0
4.0
3.0
2.0
1.0
0
-1.0
-2.0
7.512
7.492
7.257
7.198
7.179
7.173
7.154
7.087
7.084
7.050
7.048
6.859
6.400
6.382
6.005
5.987
5.969
5.777
5.755
5.727
5.469
4.534
4.505
2.487
2.485
2.322
2.246
1.550
0.000
X : parts per Million : 1H

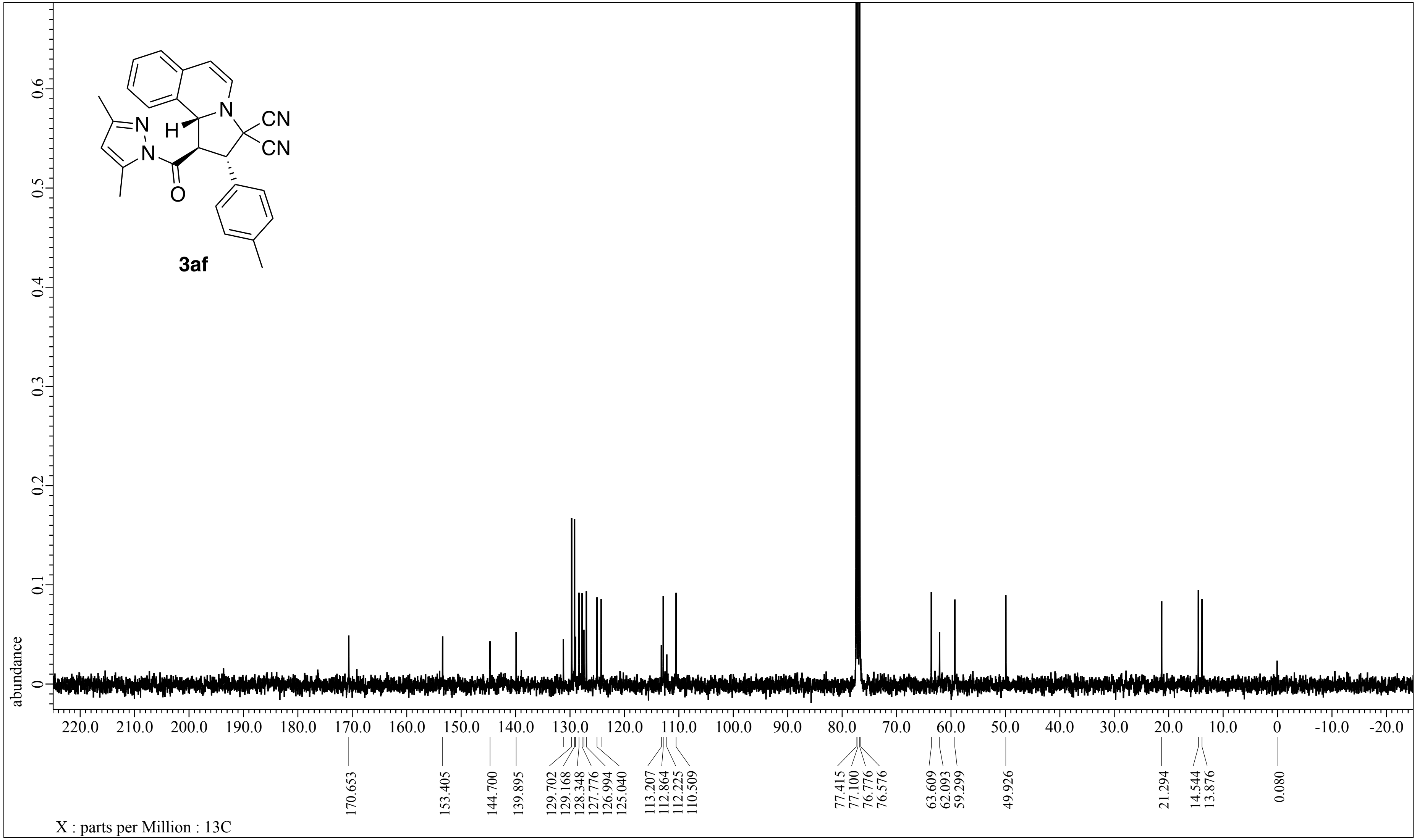
N
N
H
N
CN
CN
O
3af
abundance
0.6
0.5
0.4
0.3
0.2
0.1
0
220.0 210.0 200.0 190.0 180.0 170.0 160.0 150.0 140.0 130.0 120.0 110.0 100.0 90.0 80.0 70.0 60.0 50.0 40.0 30.0 20.0 10.0 0 -10.0 -20.0
170.653
153.405
144.700
139.895
129.702
129.168
128.348
127.776
126.994
125.040
113.207
112.864
112.225
110.509
77.415
77.100
76.776
76.576
63.609
62.093
59.299
49.926
21.294
14.544
13.876
0.080
X : parts per Million : 13C

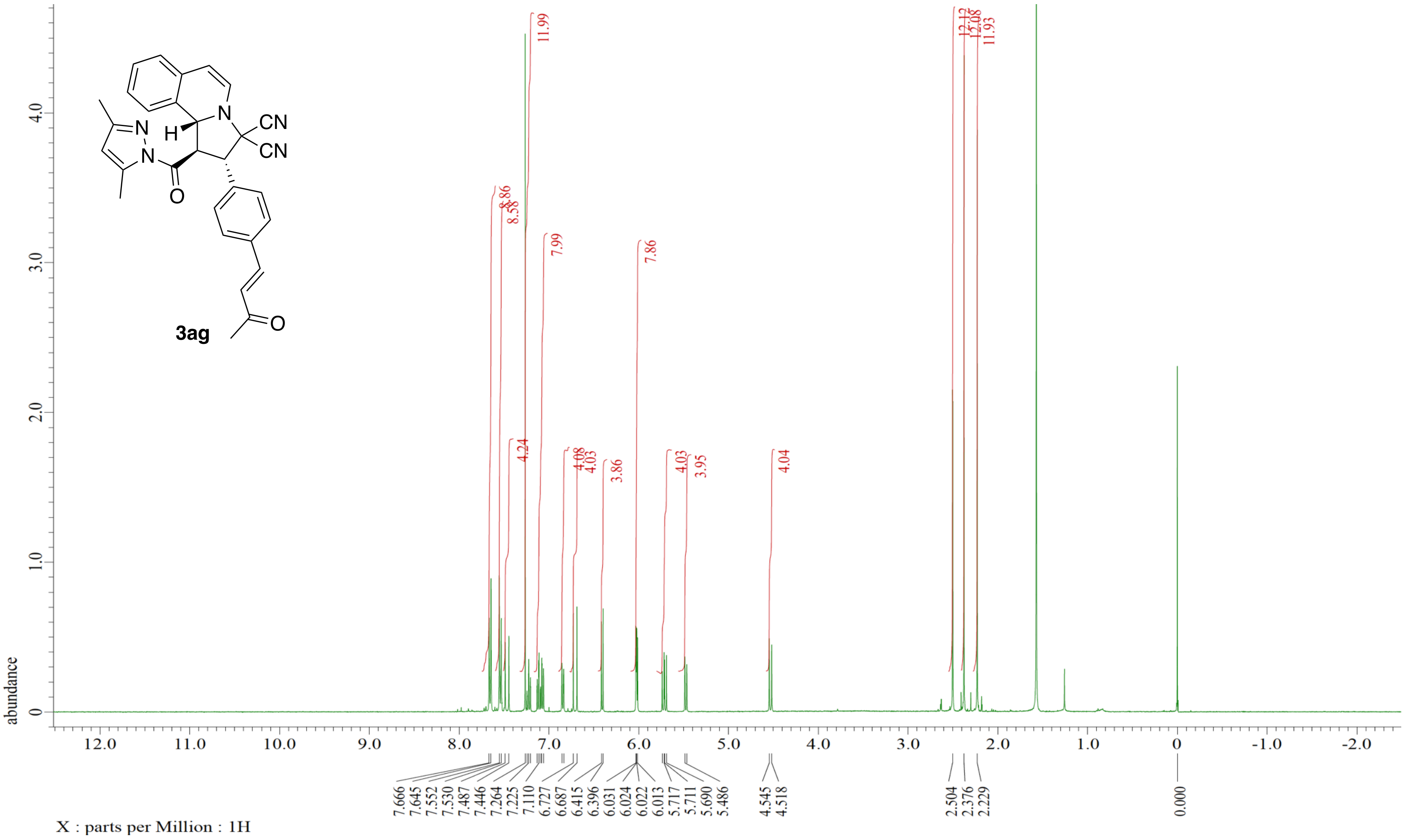
3ag
abundance
X : parts per Million : 1H

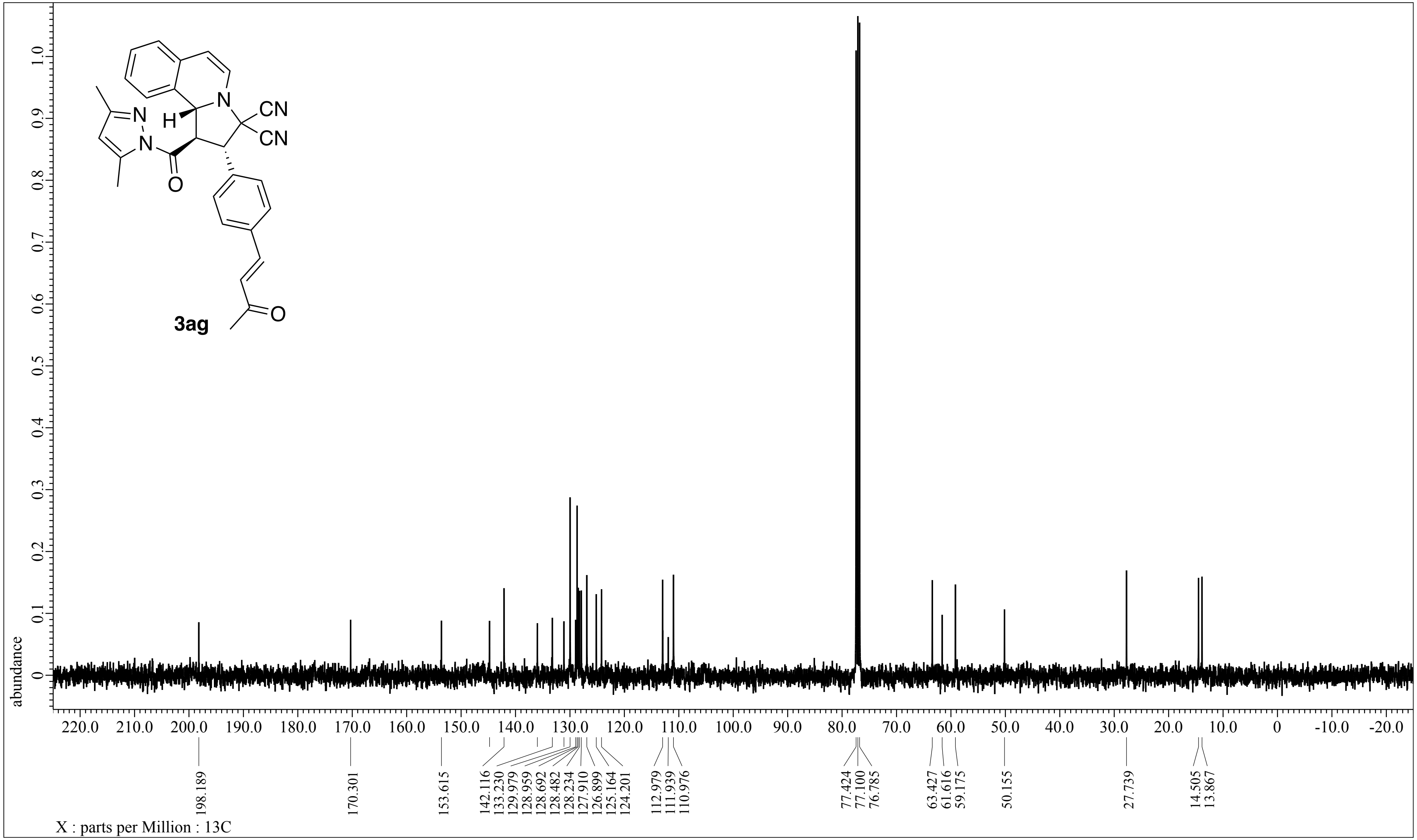

3ag
CN
CN
N
N
N
H
O
O
abundance
X : parts per Million : 13C
198.189
170.301
153.615
142.116
133.230
129.979
128.959
128.692
128.482
128.234
127.910
126.899
125.164
124.201
112.979
111.939
110.976
77.424
77.100
76.785
63.427
61.616
59.175
50.155
27.739
14.505
13.867

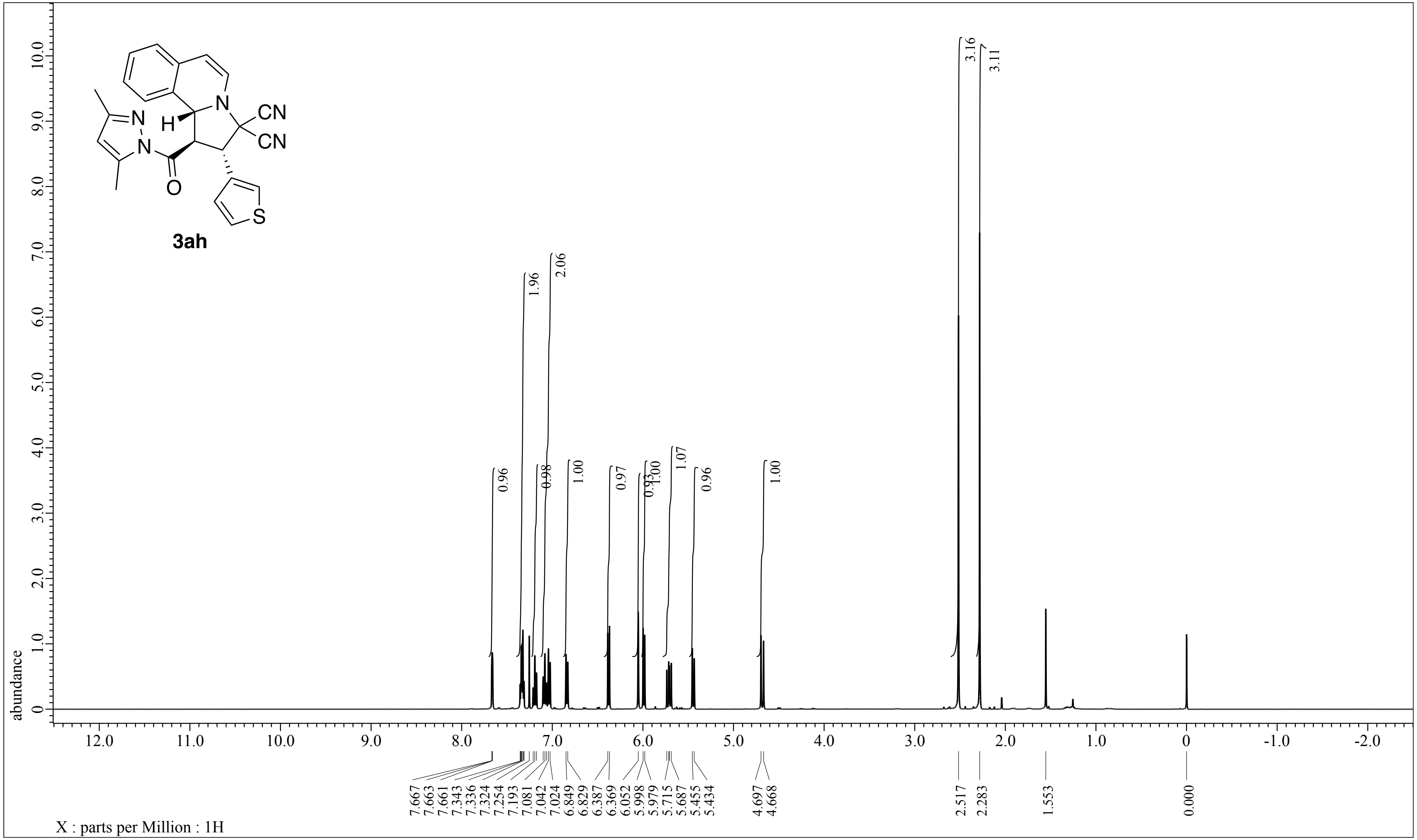

3ah
abundance
X : parts per Million : 1H
7.667
7.663
7.661
7.343
7.336
7.324
7.254
7.193
7.081
7.042
7.024
6.849
6.829
6.387
6.369
6.052
5.998
5.979
5.715
5.687
5.455
5.434
4.697
4.668
2.517
2.283
1.553
0.000

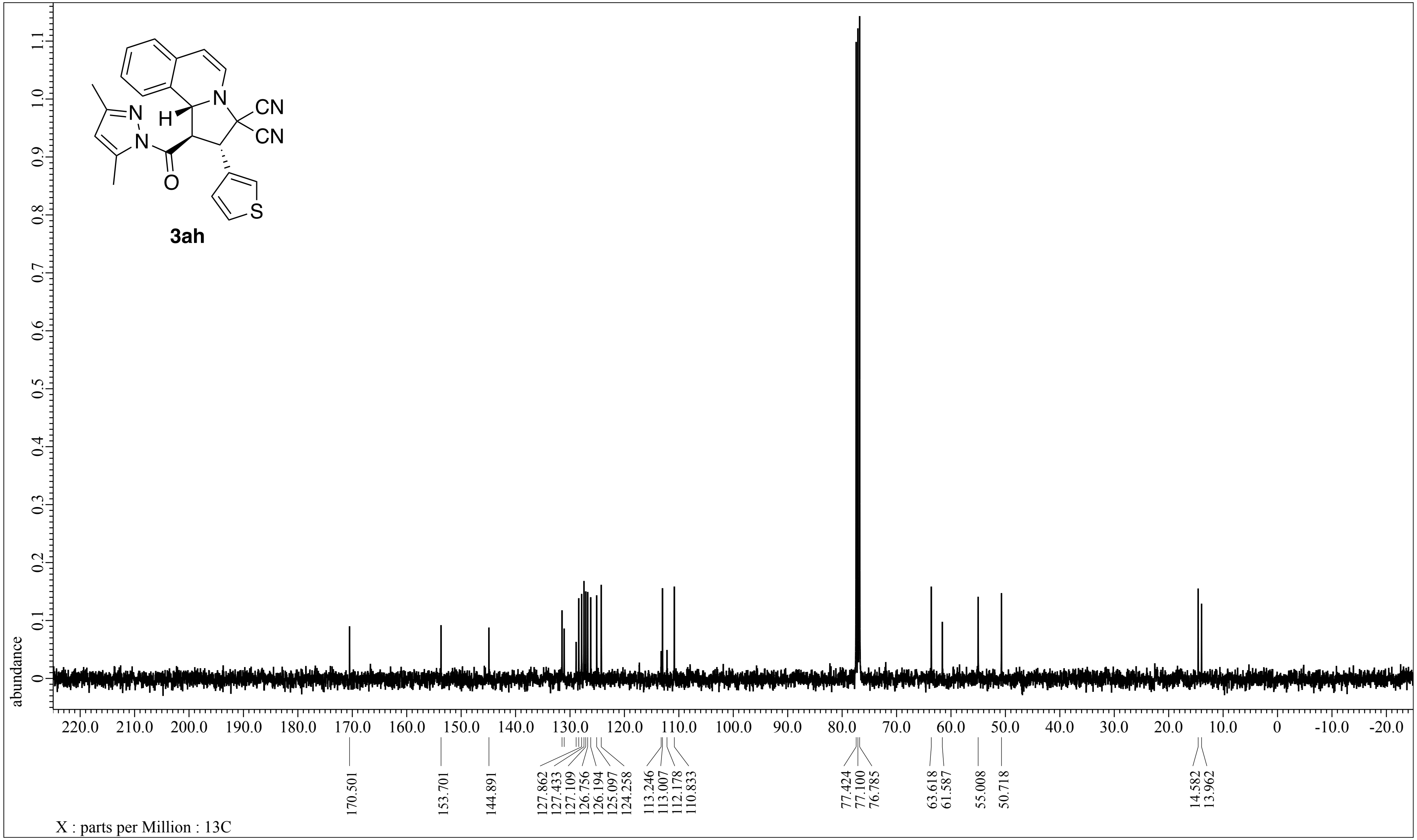

3ah
CN
CN
N
N
N
H
O
S
abundance
X : parts per Million : 13C
170.501
153.701
144.891
127.862
127.433
127.109
126.756
126.194
125.097
124.258
113.246
113.007
112.178
110.833
77.424
77.100
76.785
63.618
61.587
55.008
50.718
14.582
13.962

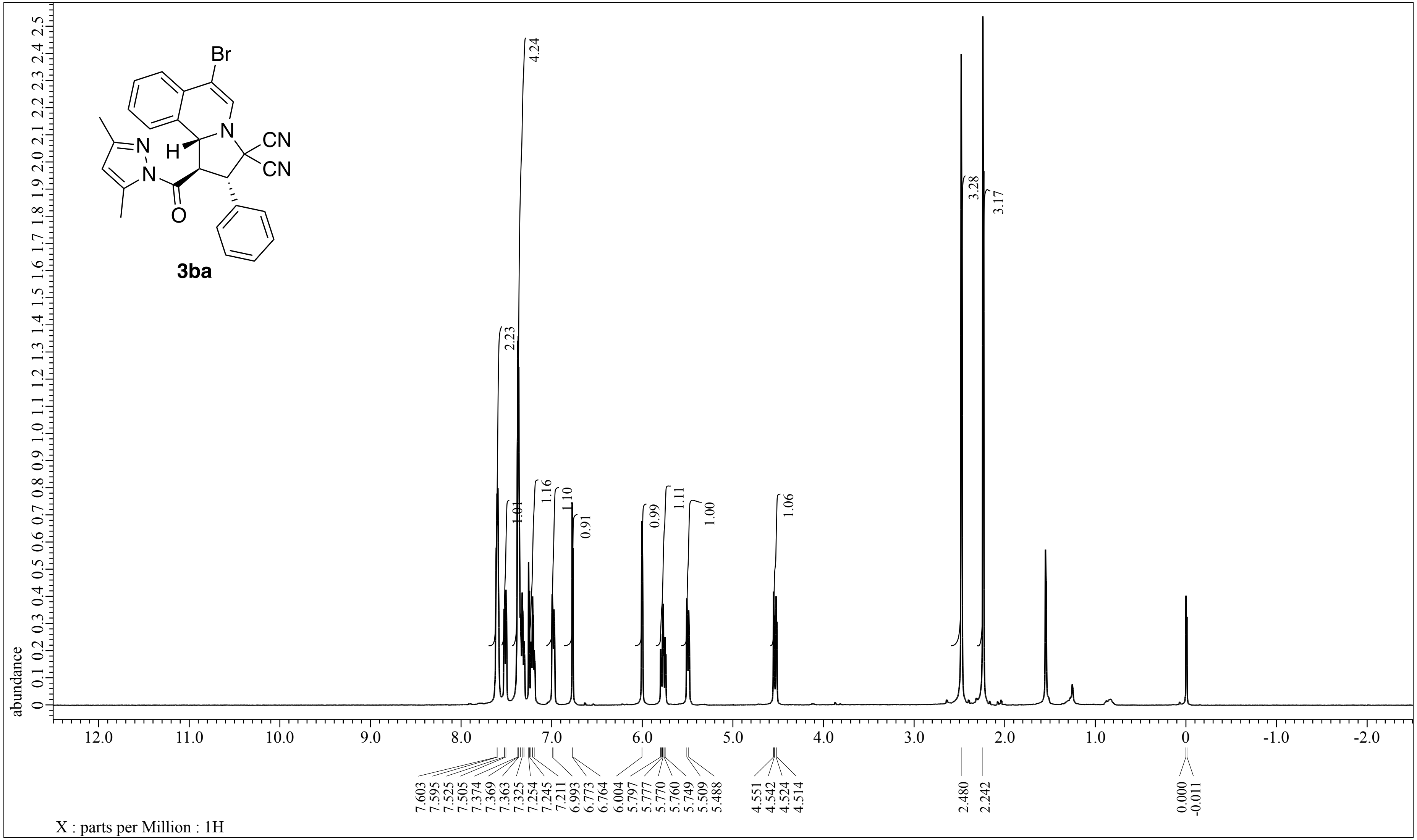

Br
N
CN
CN
H
N
N
O
3ba
4.24
2.23
1.01
1.16
1.10
0.91
0.99
1.11
1.00
1.06
3.28
3.17
abundance
X : parts per Million : 1H
7.603
7.595
7.525
7.505
7.374
7.369
7.363
7.325
7.254
7.245
7.211
6.993
6.773
6.764
6.004
5.797
5.777
5.770
5.760
5.749
5.509
5.488
4.551
4.542
4.524
4.514
2.480
2.242
0.000
-0.011

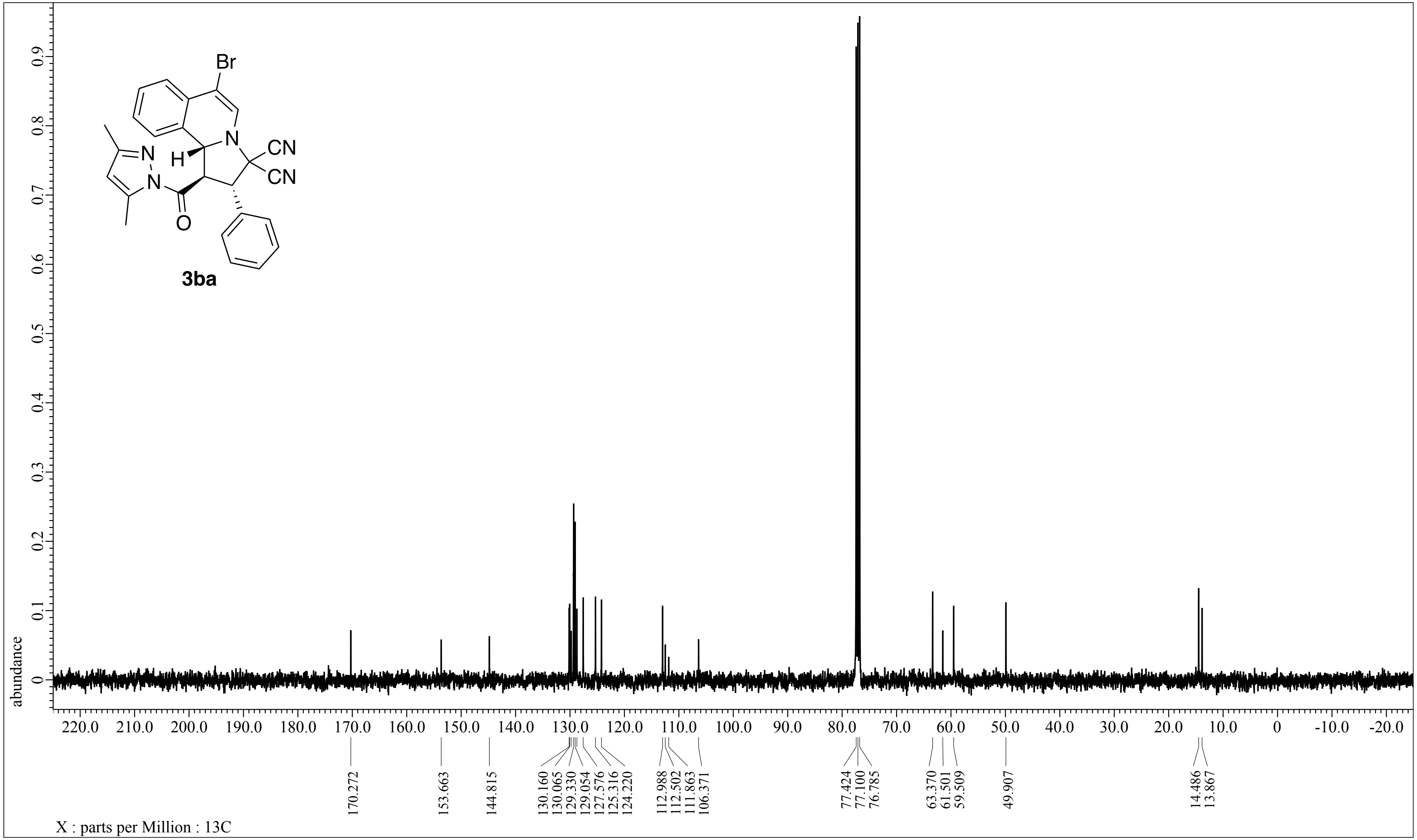

Br
N
CN
CN
H
N
N
O
3ba
abundance
X : parts per Million : 13C
170.272
153.663
144.815
130.160
130.065
129.330
129.054
127.576
125.316
124.220
112.988
112.502
111.863
106.371
77.424
77.100
76.785
63.370
61.501
59.509
49.907
14.486
13.867

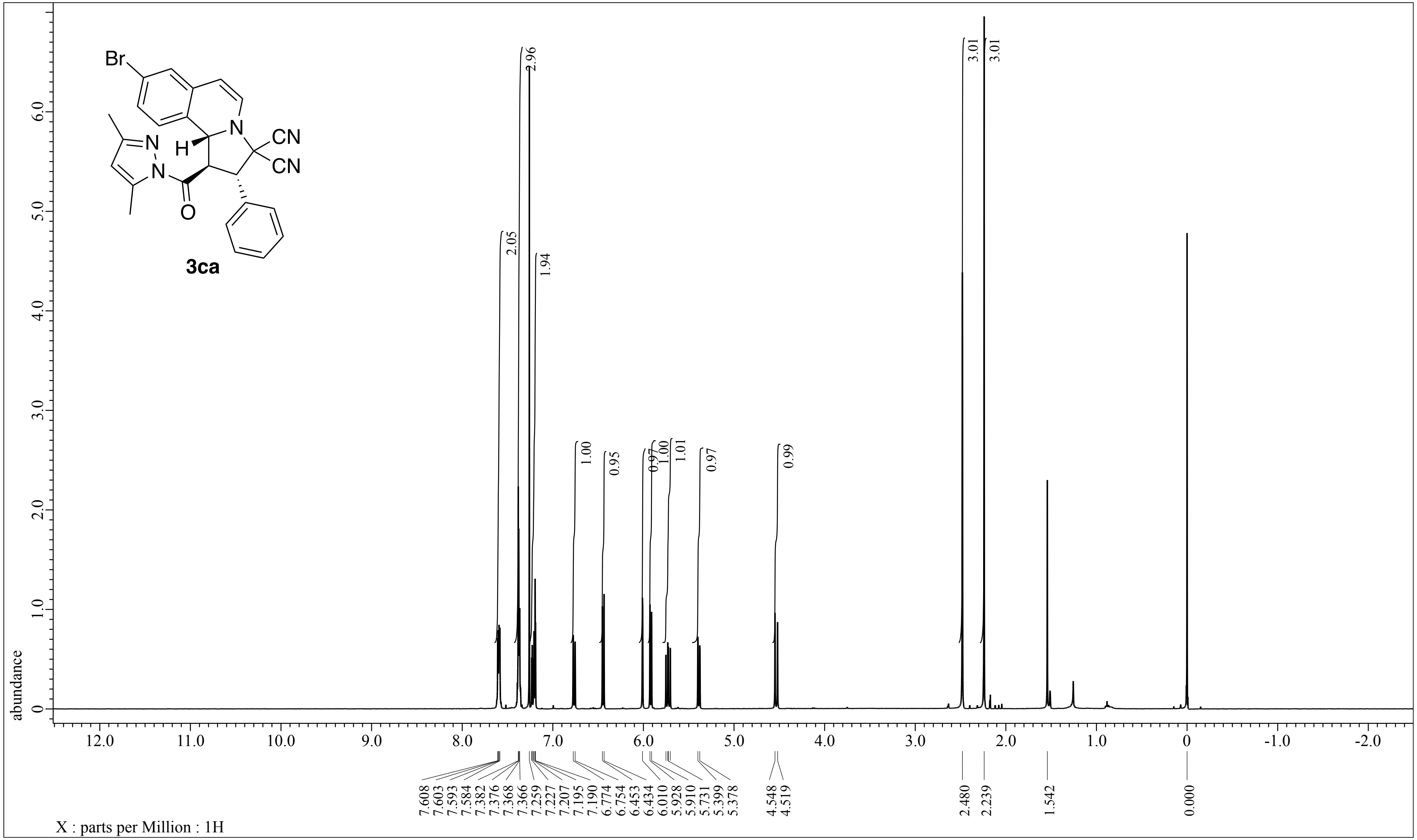

Br
N
H
N
CN
CN
N
O
3ca
2.96
3.01
3.01
2.05
1.94
1.00
0.95
0.97
1.00
1.01
0.97
0.99
abundance
X : parts per Million : 1H
7.608
7.603
7.593
7.584
7.382
7.376
7.368
7.366
7.259
7.227
7.207
7.195
7.190
6.774
6.754
6.453
6.434
6.010
5.928
5.910
5.731
5.399
5.378
4.548
4.519
2.480
2.239
1.542
0.000

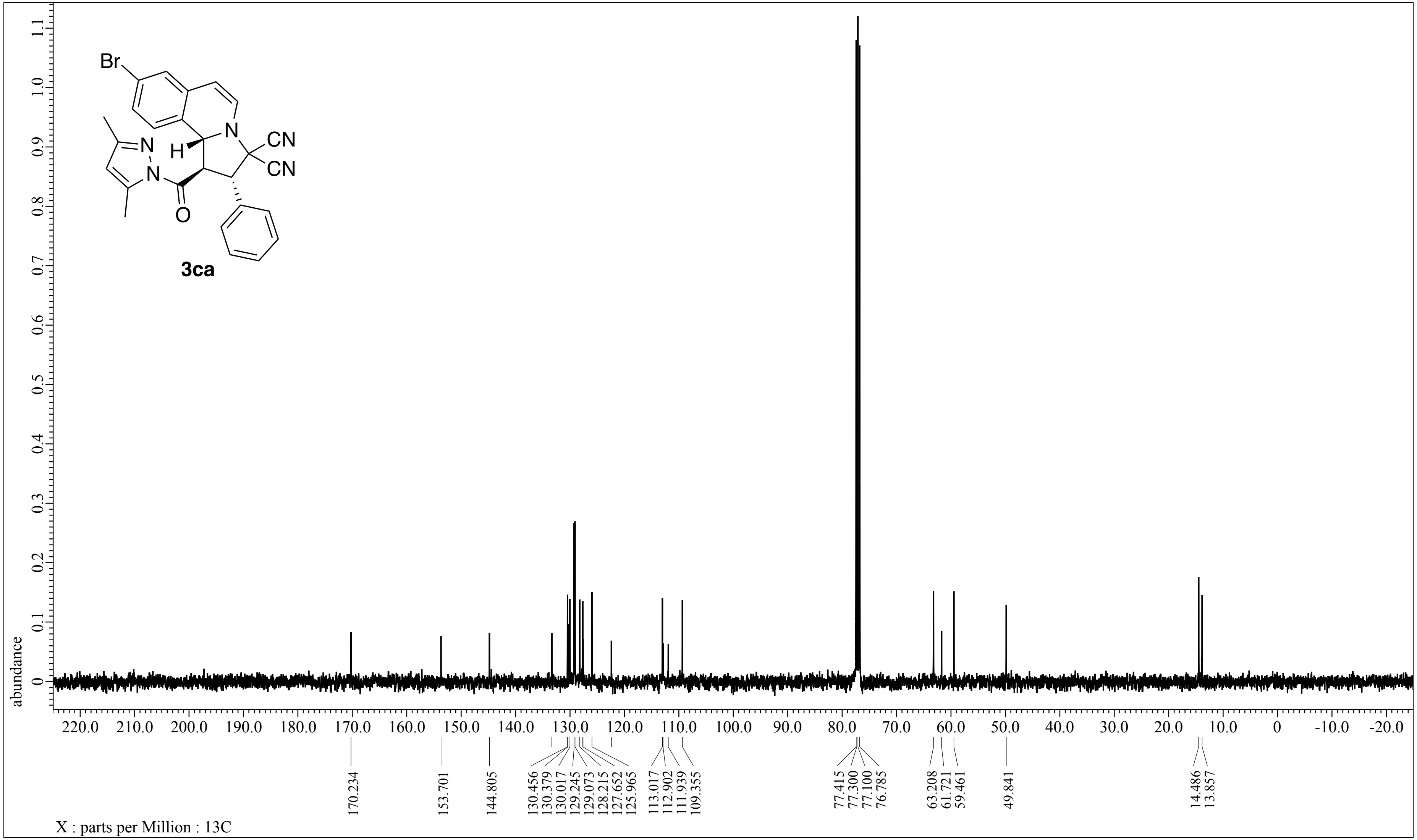

Br
N
N
H
N
CN
CN
O
3ca
abundance
1.1
1.0
0.9
0.8
0.7
0.6
0.5
0.4
0.3
0.2
0.1
0
220.0 210.0 200.0 190.0 180.0 170.0 160.0 150.0 140.0 130.0 120.0 110.0 100.0 90.0 80.0 70.0 60.0 50.0 40.0 30.0 20.0 10.0 0 -10.0 -20.0
170.234
153.701
144.805
130.456
130.379
130.017
129.245
129.073
128.215
127.652
125.965
113.017
112.902
111.939
109.355
77.415
77.300
77.100
76.785
63.208
61.721
59.461
49.841
14.486
13.857
X : parts per Million : 13C

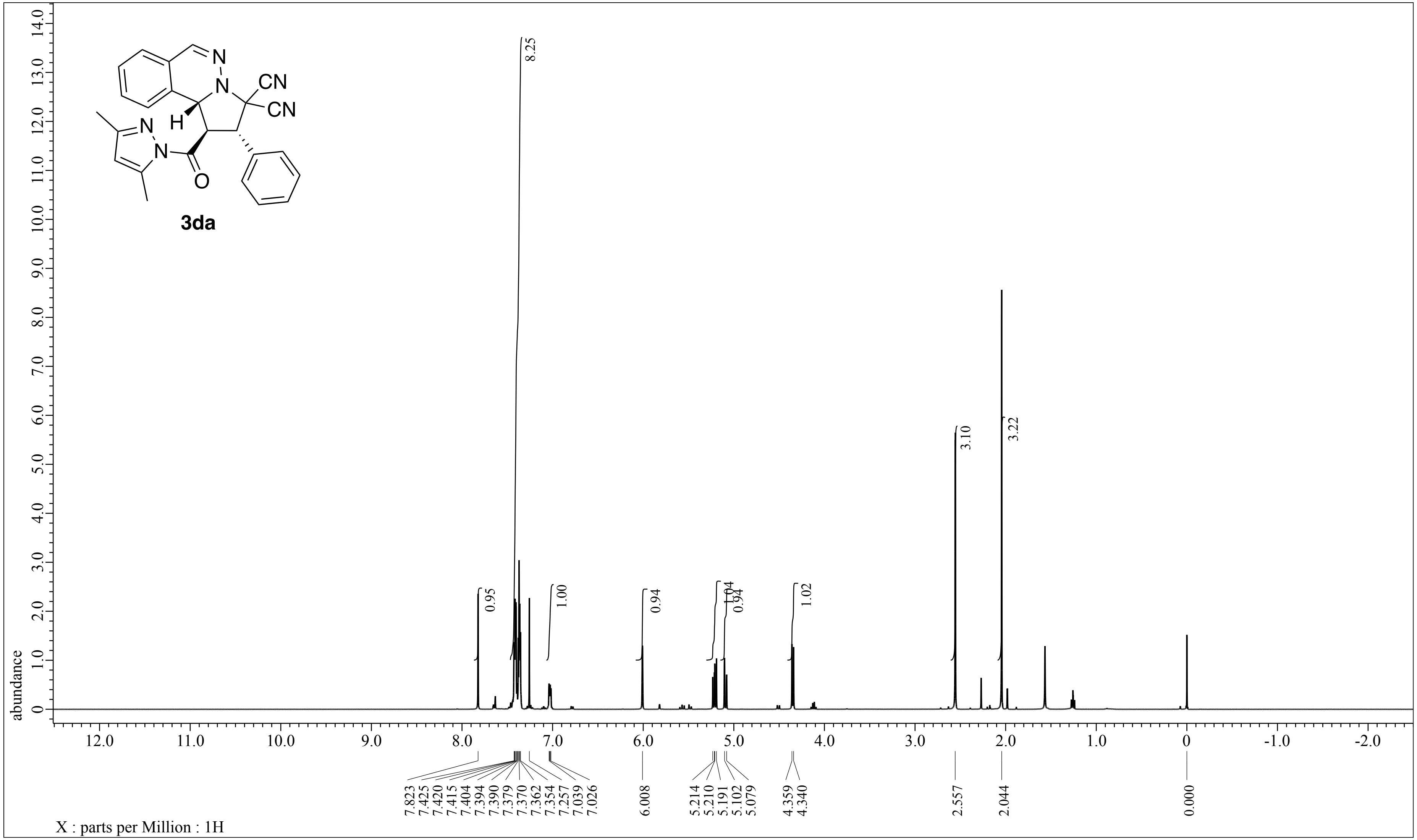

3da
abundance
X : parts per Million : 1H
8.25
0.95
1.00
0.94
1.04
0.94
1.02
3.10
3.22
7.823
7.425
7.420
7.415
7.404
7.394
7.390
7.379
7.370
7.362
7.354
7.257
7.039
7.026
6.008
5.214
5.210
5.191
5.102
5.079
4.359
4.340
2.557
2.044
0.000

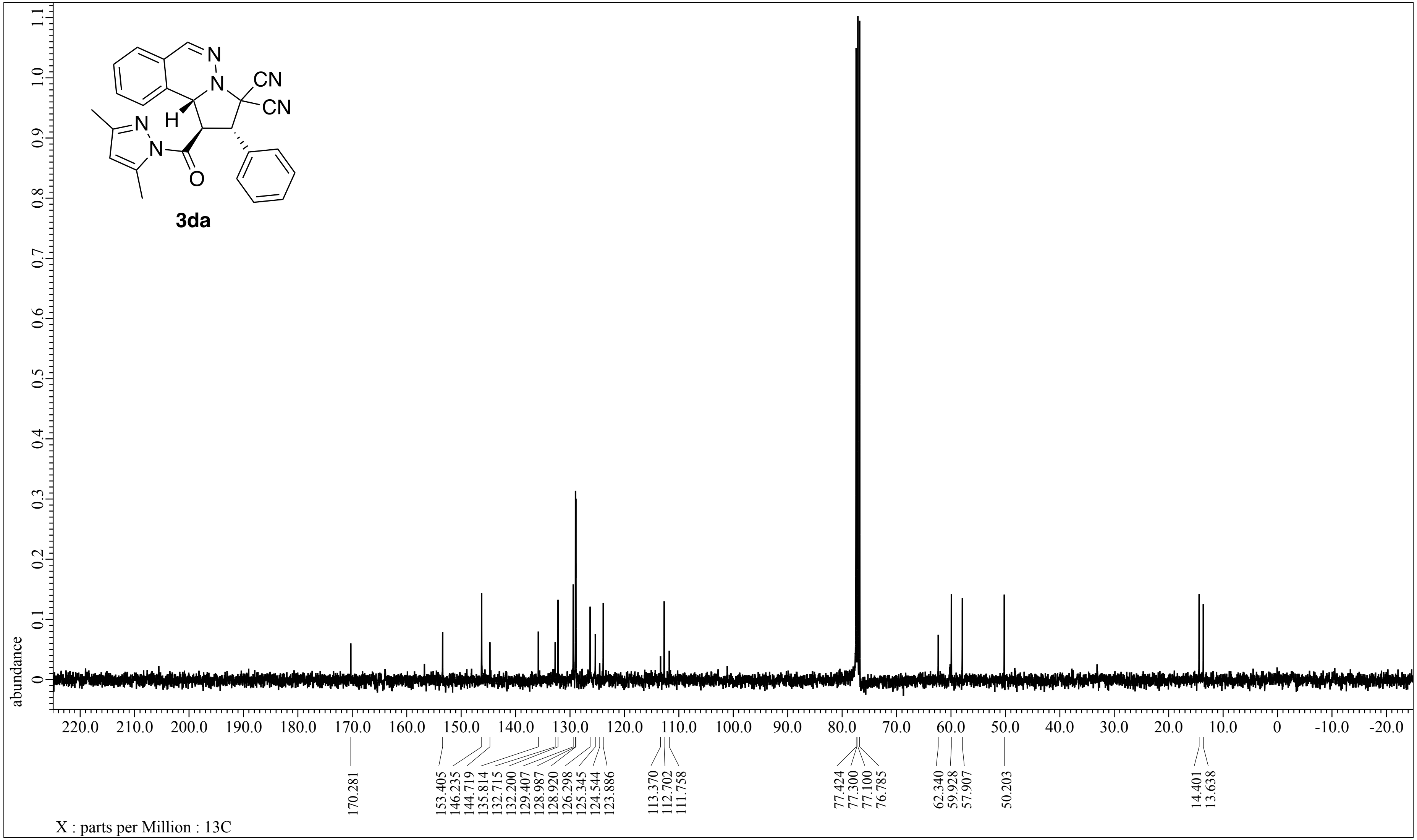

3da
abundance
X : parts per Million : 13C
170.281
153.405
146.235
144.719
135.814
132.715
132.200
129.407
128.987
128.920
126.298
125.345
124.544
123.886
113.370
112.702
111.758
77.424
77.300
77.100
76.785
62.340
59.928
57.907
50.203
14.401
13.638

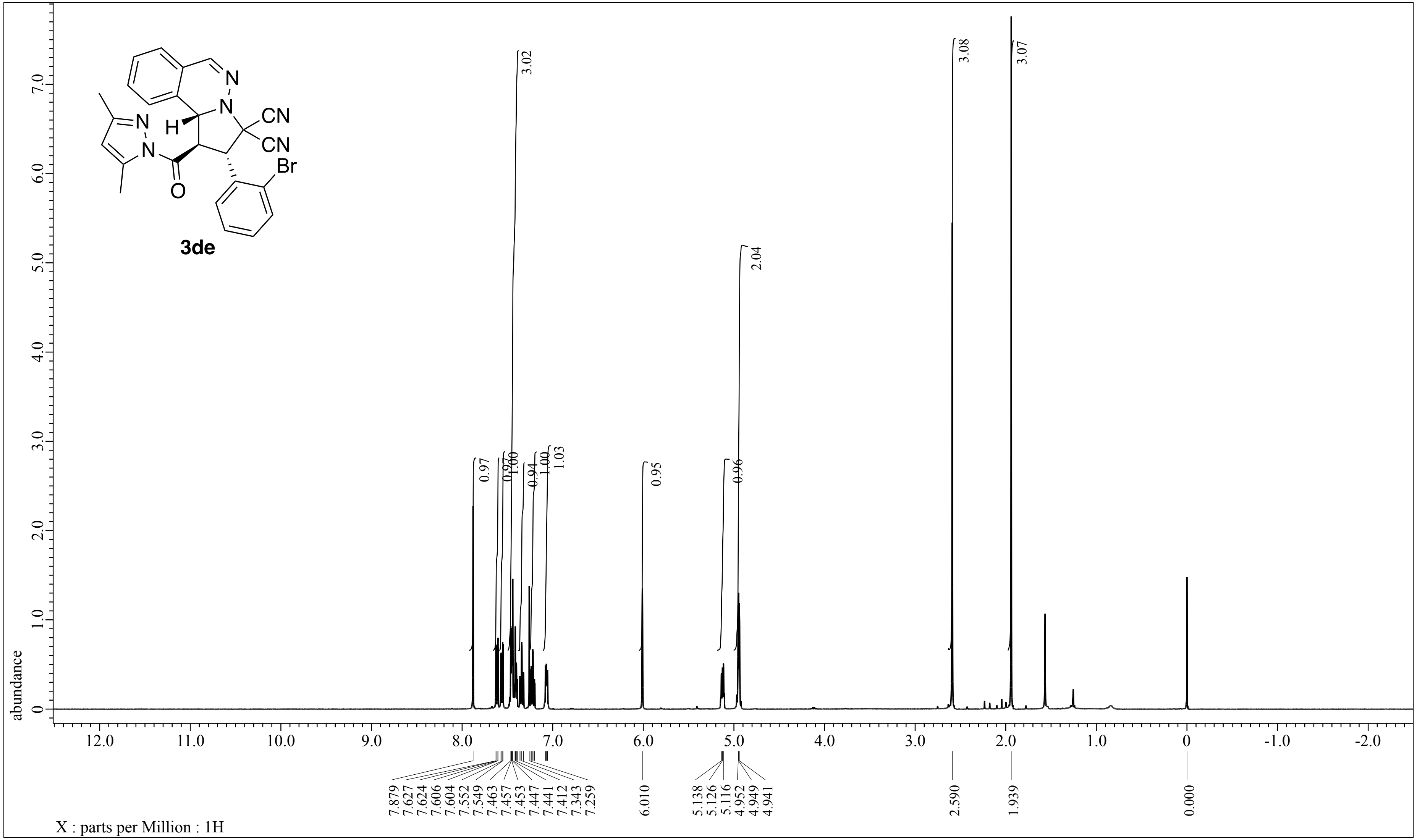
3de
abundance
X : parts per Million : 1H
7.879 7.627 7.624 7.606 7.604 7.552 7.549 7.463 7.457 7.453 7.447 7.441 7.412 7.343 7.259
6.010
5.138 5.126 5.116 4.952 4.949 4.941
2.590
1.939
0.000
0.97 0.96 1.00 0.94 1.00 1.03 3.02 0.95 0.96 2.04 3.08 3.07

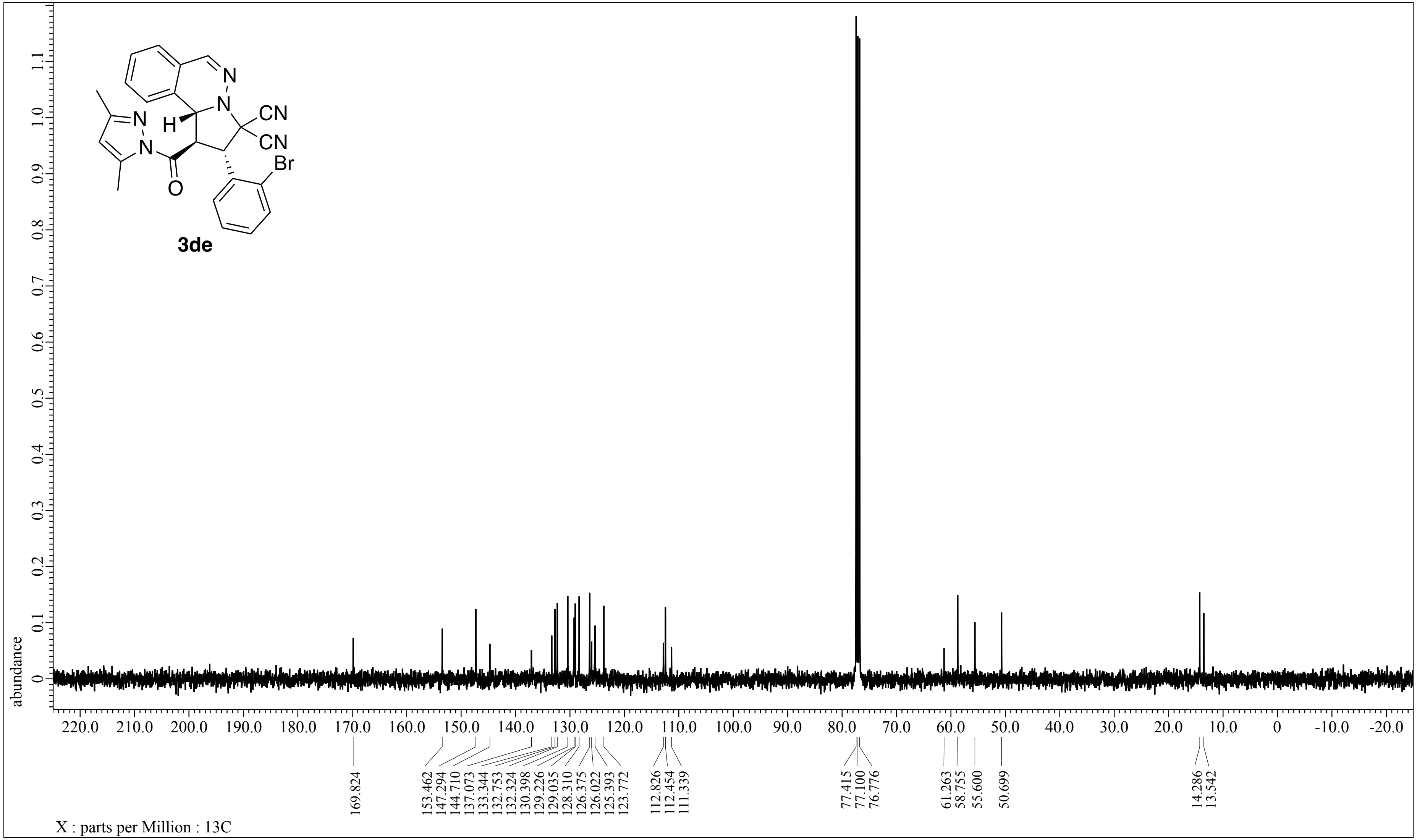
3de
abundance
X : parts per Million : 13C
169.824
153.462
147.294
144.710
137.073
133.344
132.753
132.324
130.398
129.226
129.035
128.310
126.375
126.022
125.393
123.772
112.826
112.454
111.339
77.415
77.100
76.776
61.263
58.755
55.600
50.699
14.286
13.542

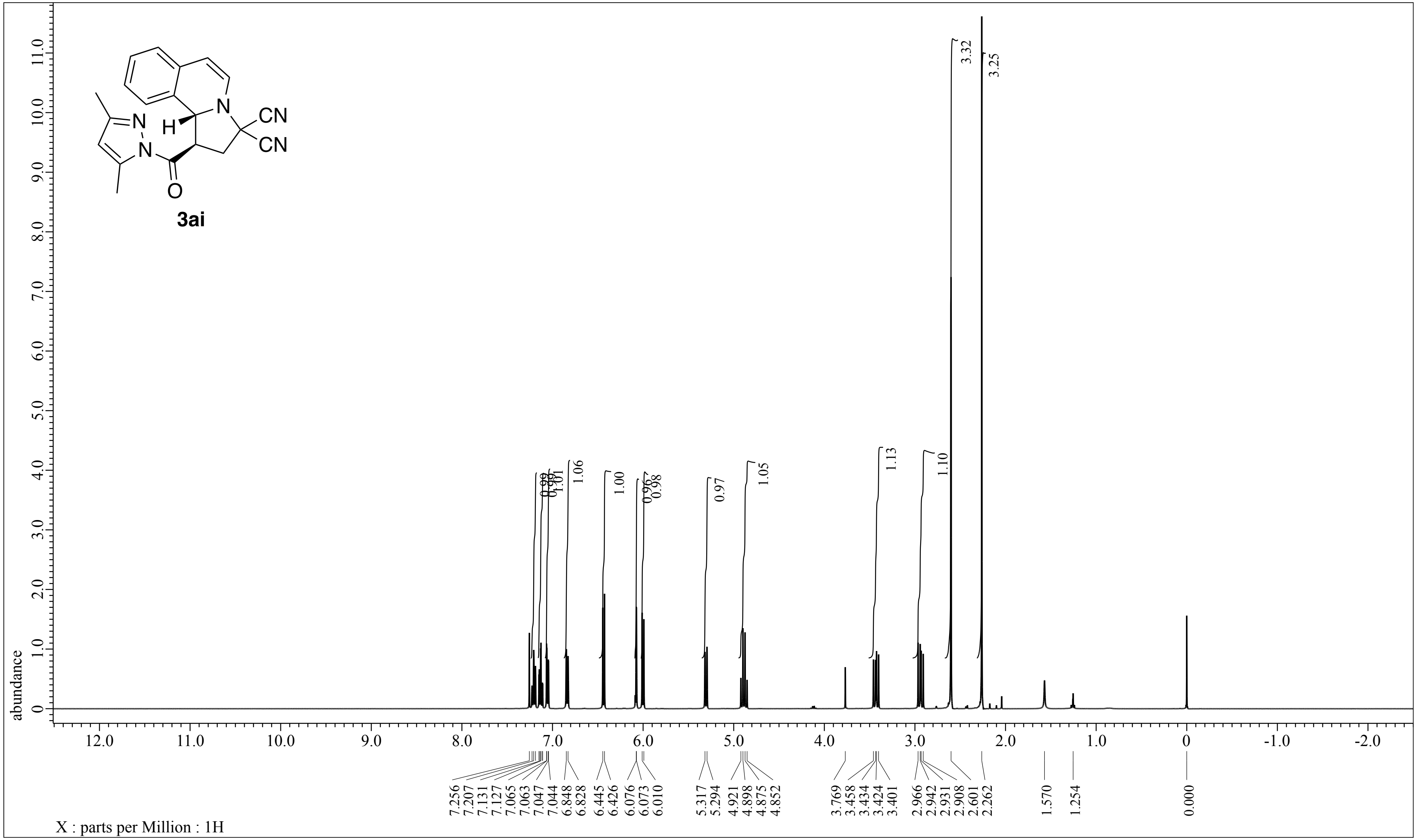
3ai
CN
CN
N
H
abundance
X : parts per Million : 1H
7.256
7.207
7.131
7.127
7.065
7.063
7.047
7.044
6.848
6.828
6.445
6.426
6.076
6.073
6.010
5.317
5.294
4.921
4.898
4.875
4.852
3.769
3.458
3.434
3.424
3.401
2.966
2.942
2.931
2.908
2.601
2.262
1.570
1.254
0.000
3.32
3.25
1.13
1.10
1.06
1.00
0.97
1.05
0.96
0.98

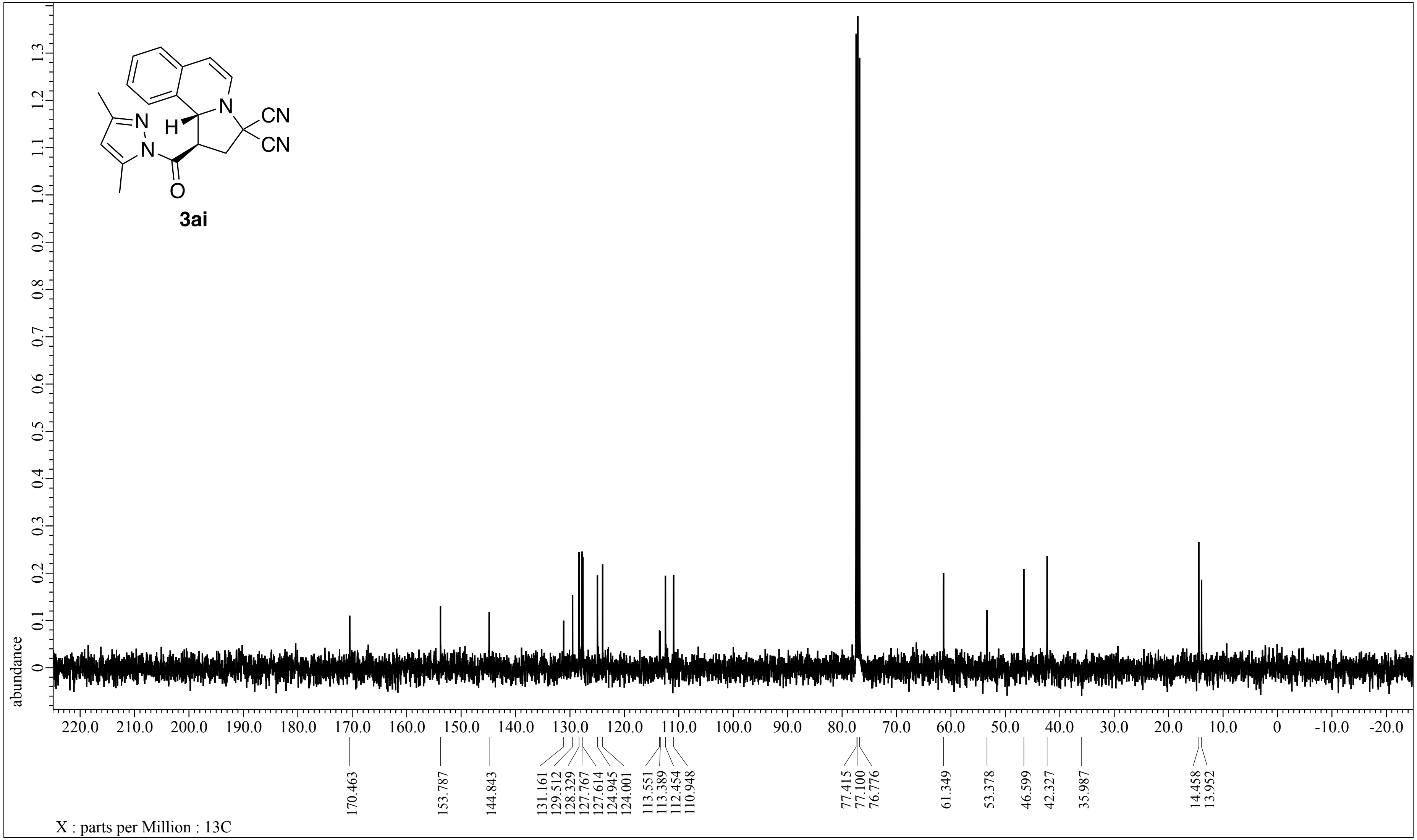
3ai
CN
CN
N
N
N
H
O
abundance
0
0.1
0.2
0.3
0.4
0.5
0.6
0.7
0.8
0.9
1.0
1.1
1.2
1.3
220.0 210.0 200.0 190.0 180.0 170.0 160.0 150.0 140.0 130.0 120.0 110.0 100.0 90.0 80.0 70.0 60.0 50.0 40.0 30.0 20.0 10.0 0 -10.0 -20.0
170.463
153.787
144.843
131.161
129.512
128.329
127.767
127.614
124.945
124.001
113.551
113.389
112.454
110.948
77.415
77.100
76.776
61.349
53.378
46.599
42.327
35.987
14.458
13.952
X : parts per Million : 13C

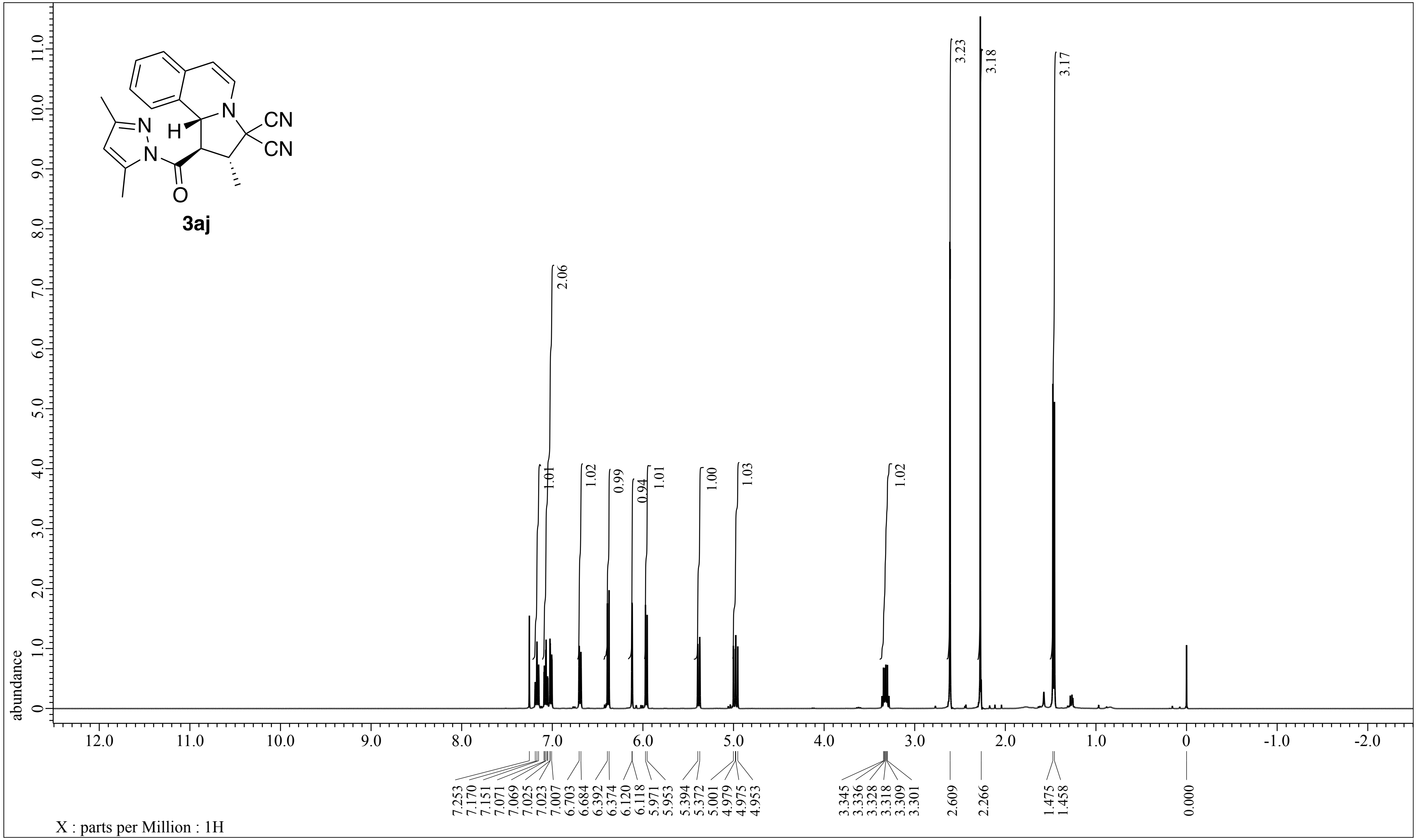
3aj
abundance
X : parts per Million : 1H
3.23
3.18
3.17
2.06
1.01
1.02
0.99
0.94
1.01
1.00
1.03
1.02
7.253
7.170
7.151
7.071
7.069
7.025
7.023
7.007
6.703
6.684
6.392
6.374
6.120
6.118
5.971
5.953
5.394
5.372
5.001
4.979
4.975
4.953
3.345
3.336
3.328
3.318
3.309
3.301
2.609
2.266
1.475
1.458
0.000

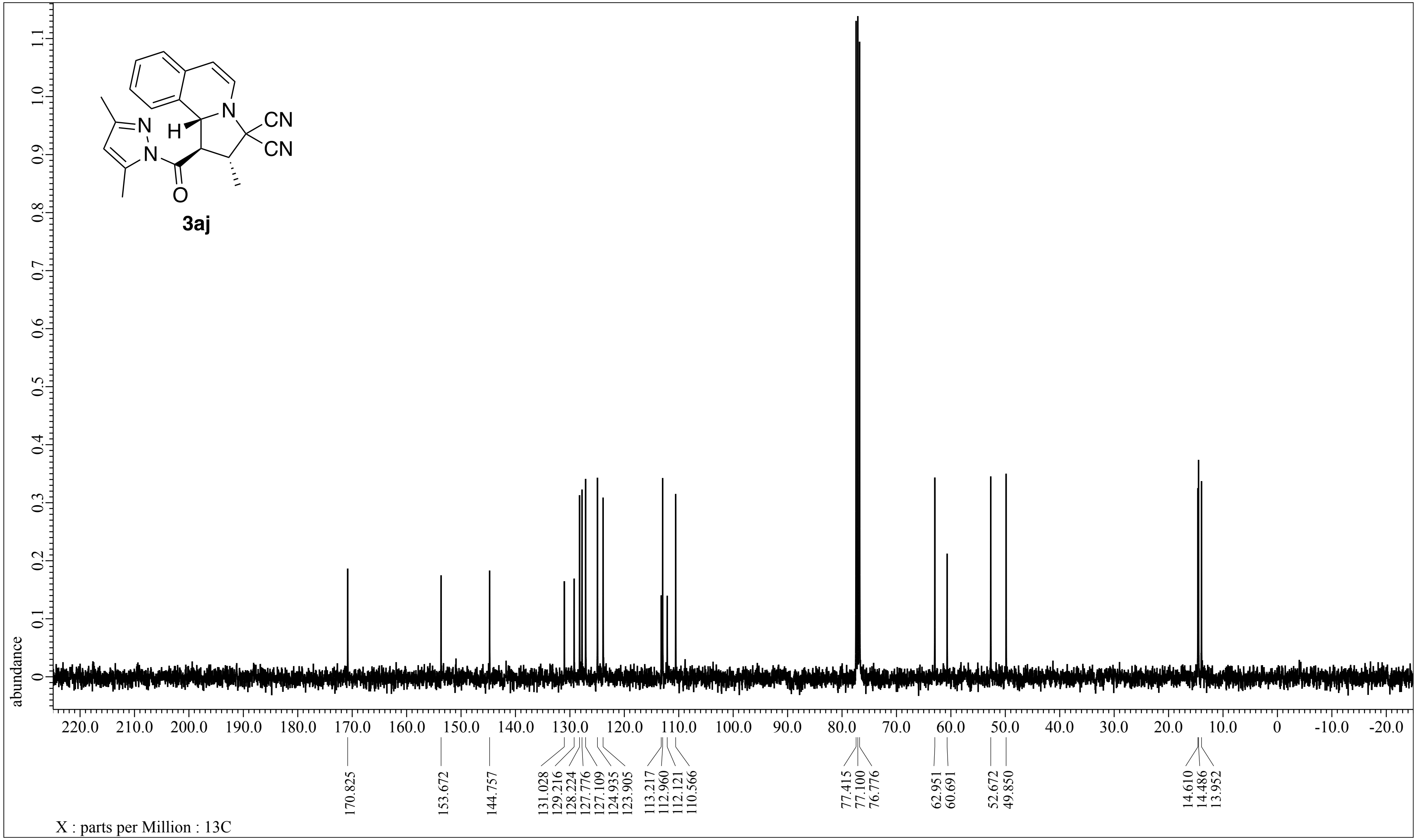

CN
CN
N
H
N
N
O
3aj
abundance
X : parts per Million : 13C
170.825
153.672
144.757
131.028
129.216
128.224
127.776
127.109
124.935
123.905
113.217
112.960
112.121
110.566
77.415
77.100
76.776
62.951
60.691
52.672
49.850
14.610
14.486
13.952

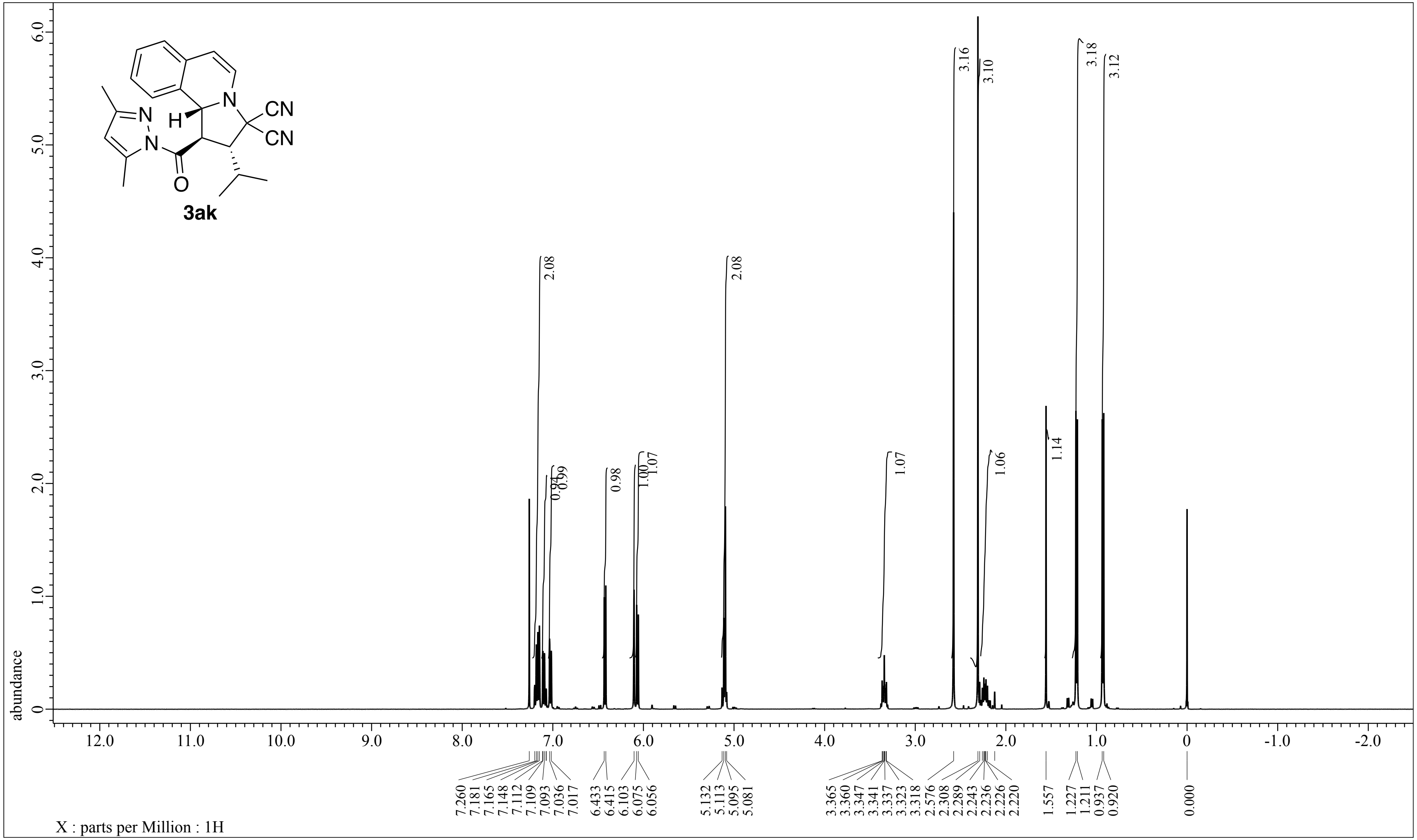
3ak
CN
CN
N
N
N
O
H
abundance
X : parts per Million : 1H
2.08
0.94
0.99
0.98
1.00
1.07
2.08
1.07
3.16
3.10
1.06
1.14
3.18
3.12
7.260
7.181
7.165
7.148
7.112
7.109
7.093
7.036
7.017
6.433
6.415
6.103
6.075
6.056
5.132
5.113
5.095
5.081
3.365
3.360
3.347
3.341
3.337
3.323
3.318
2.576
2.308
2.289
2.243
2.236
2.226
2.220
1.557
1.227
1.211
0.937
0.920
0.000

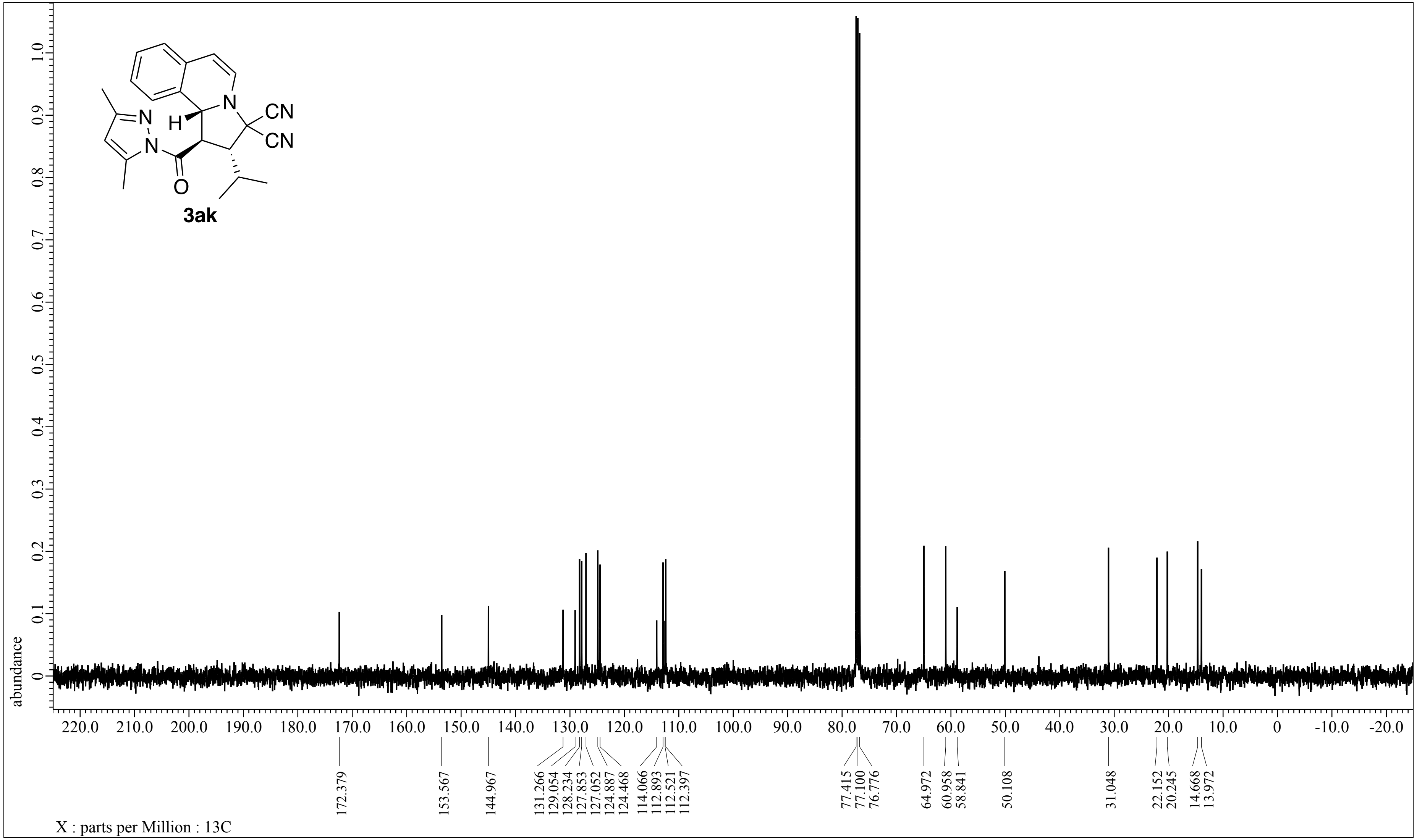

3ak
CN
CN
N
N
N
H
O
abundance
X : parts per Million : 13C
172.379
153.567
144.967
131.266
129.054
128.234
127.853
127.052
124.887
124.468
114.066
112.893
112.521
112.397
77.415
77.100
76.776
64.972
60.958
58.841
50.108
31.048
22.152
20.245
14.668
13.972

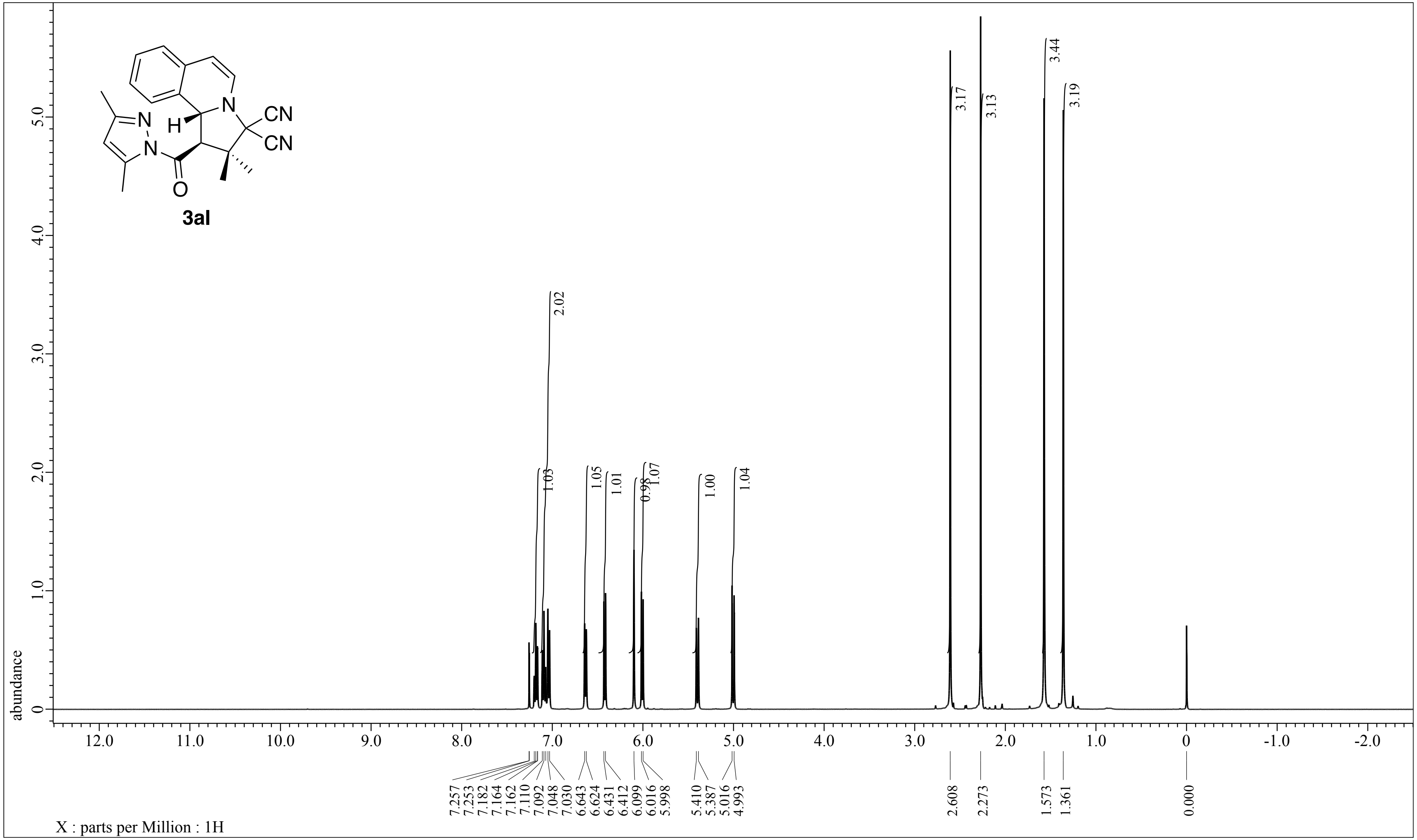

N
N
H
N
CN
CN
O
3al
abundance
3.17
3.13
3.44
3.19
2.02
1.03
1.05
1.01
0.98
1.07
1.00
1.04
X : parts per Million : 1H
7.257
7.253
7.182
7.164
7.162
7.110
7.092
7.048
7.030
6.643
6.624
6.431
6.412
6.099
6.016
5.998
5.410
5.387
5.016
4.993
2.608
2.273
1.573
1.361
0.000

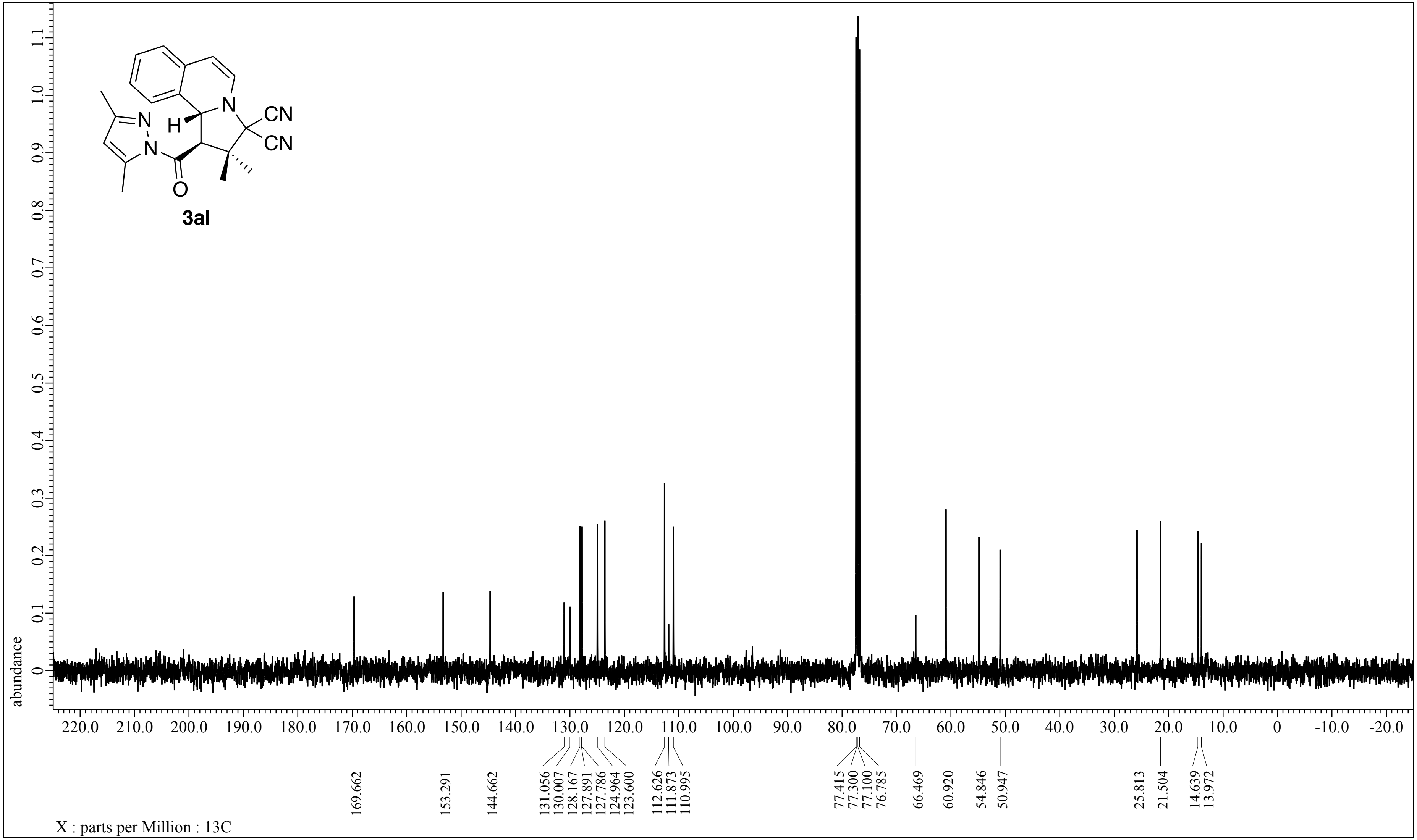

N
N
H
N
CN
CN
O
3al
abundance
X : parts per Million : 13C
169.662
153.291
144.662
131.056
130.007
128.167
127.891
127.786
124.964
123.600
112.626
111.873
110.995
77.415
77.300
77.100
76.785
66.469
60.920
54.846
50.947
25.813
21.504
14.639
13.972

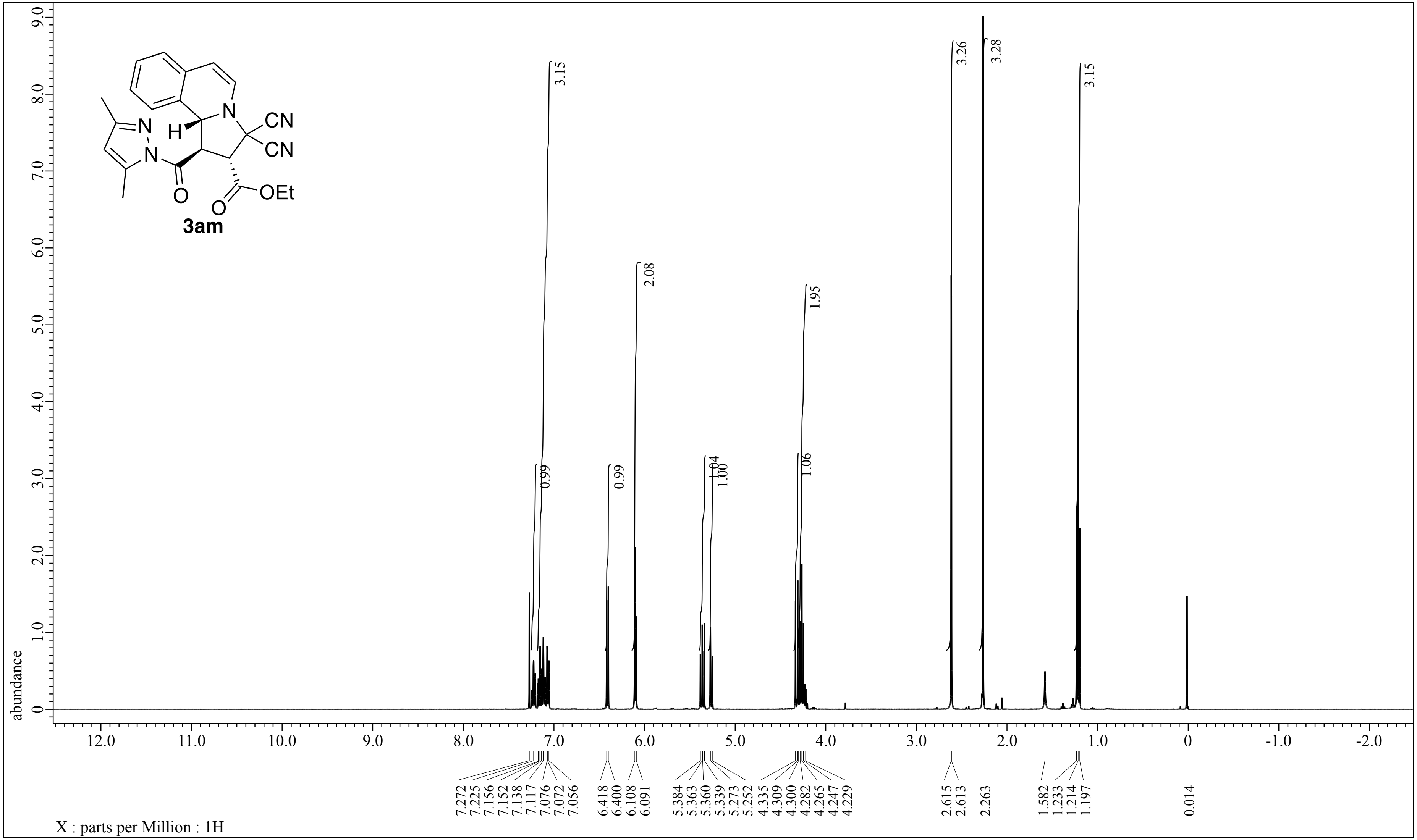
3am
CN
CN
OEt
abundance
X : parts per Million : 1H
3.15
0.99
0.99
2.08
1.04
1.00
1.06
1.95
3.26
3.28
3.15
7.272
7.225
7.156
7.152
7.138
7.117
7.076
7.072
7.056
6.418
6.400
6.108
6.091
5.384
5.363
5.360
5.339
5.273
5.252
4.335
4.309
4.300
4.282
4.265
4.247
4.229
2.615
2.613
2.263
1.582
1.233
1.214
1.197
0.014

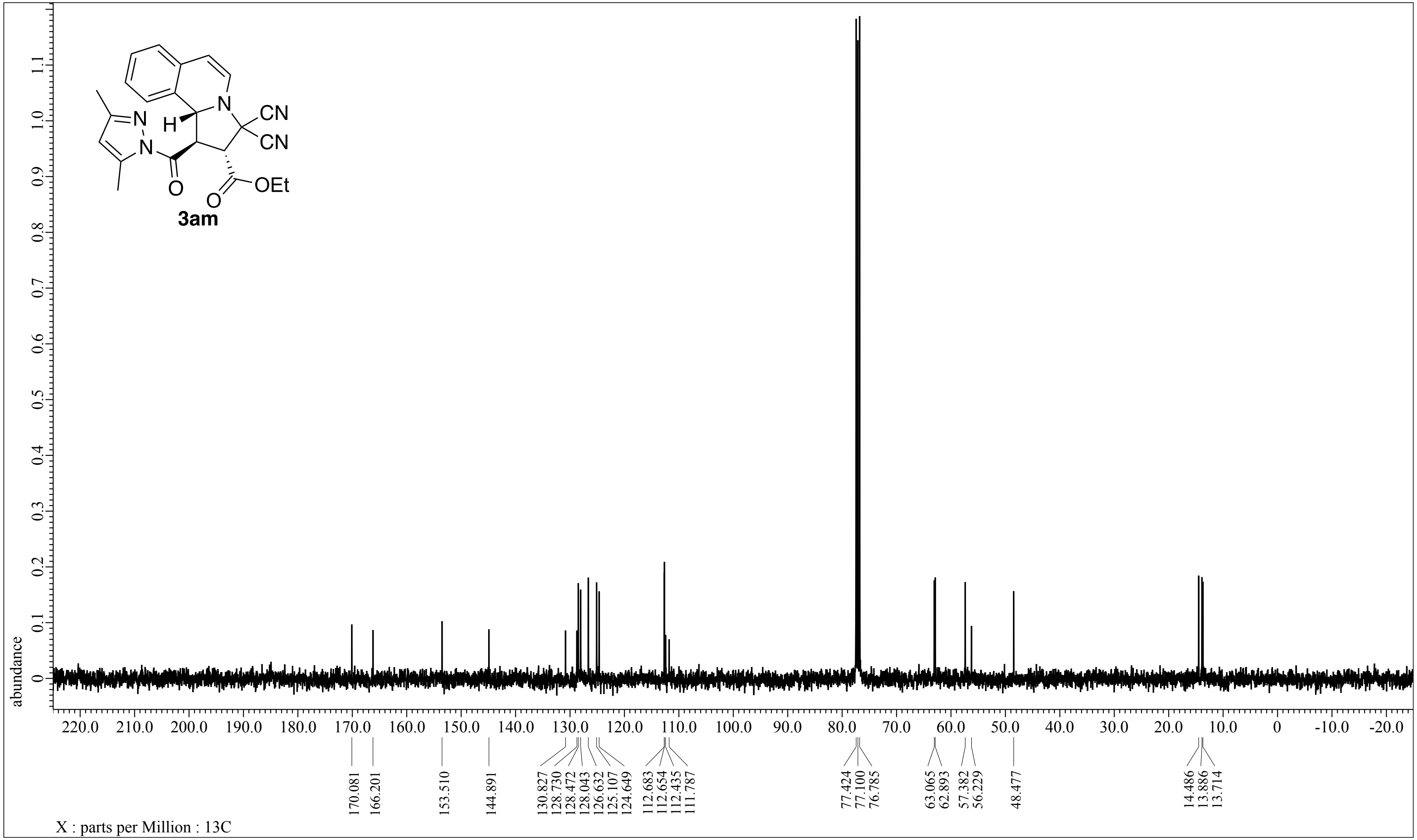
3am
CN
CN
N
N
N
H
O
O
OEt
abundance
X : parts per Million : 13C
170.081
166.201
153.510
144.891
130.827
128.730
128.472
128.043
126.632
125.107
124.649
112.683
112.654
112.435
111.787
77.424
77.100
76.785
63.065
62.893
57.382
56.229
48.477
14.486
13.886
13.714

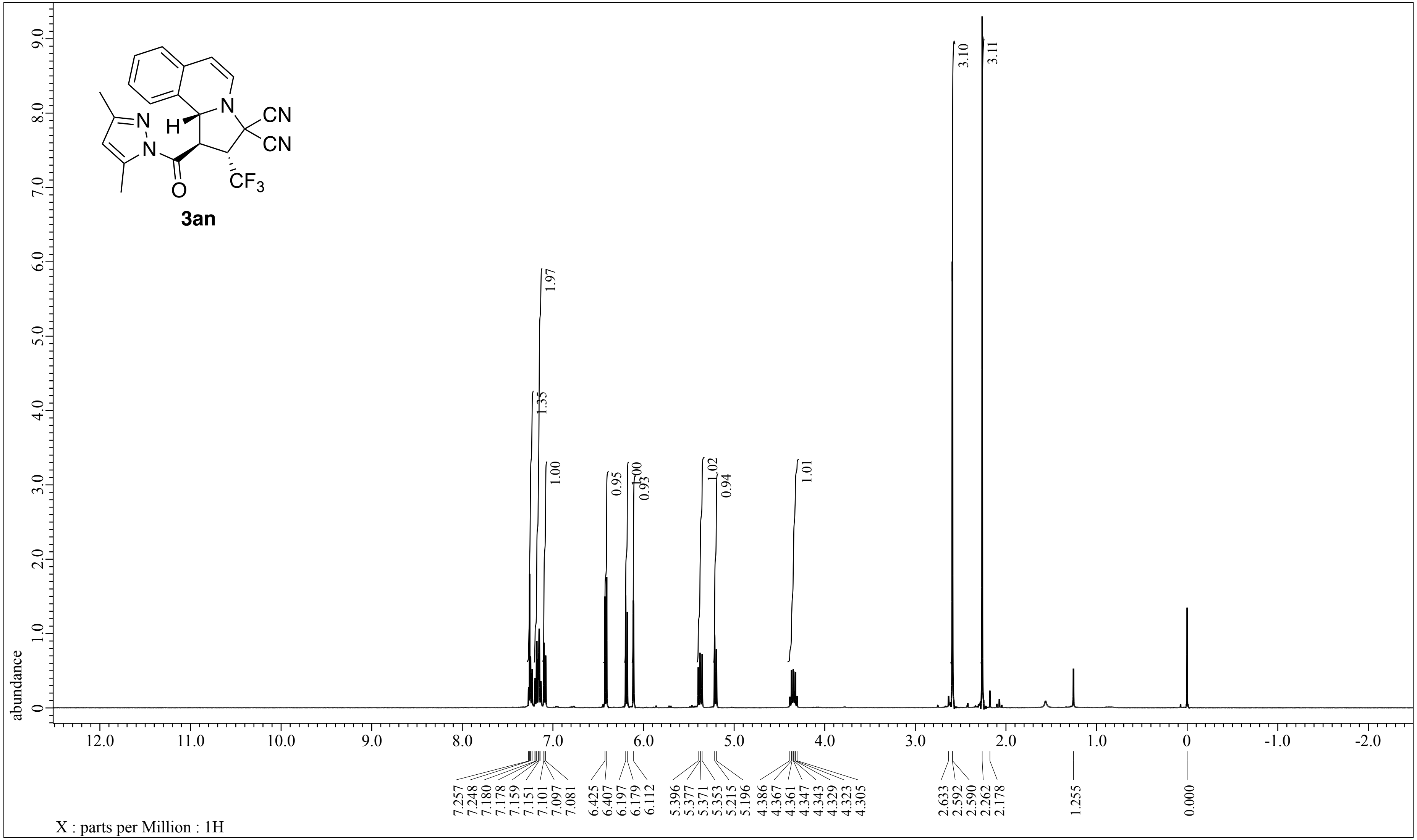

3an
CN
CN
N
N
N
H
O
CF3
abundance
X : parts per Million : 1H
12.0 11.0 10.0 9.0 8.0 7.0 6.0 5.0 4.0 3.0 2.0 1.0 0 -1.0 -2.0
0 1.0 2.0 3.0 4.0 5.0 6.0 7.0 8.0 9.0
1.97
1.35
1.00
0.95
1.00
0.93
1.02
0.94
1.01
3.10
3.11
7.257 7.248 7.180 7.178 7.159 7.151 7.101 7.097 7.081
6.425 6.407 6.197 6.179 6.112
5.396 5.377 5.371 5.353 5.215 5.196
4.386 4.367 4.361 4.347 4.343 4.329 4.323 4.305
2.633 2.592 2.590 2.262 2.178
1.255
0.000

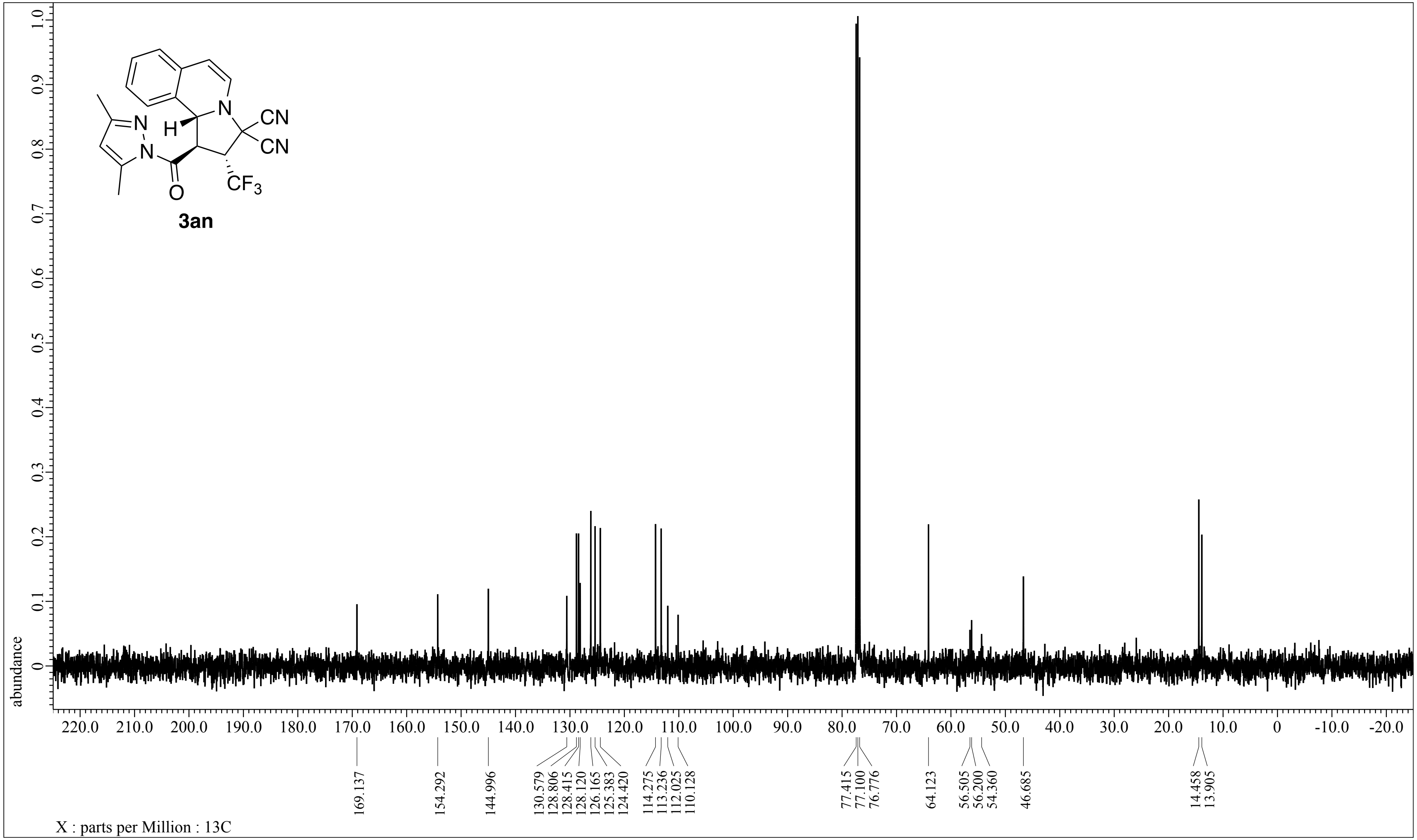

CN
CN
N
N
N
H
O
CF3
3an
abundance
X : parts per Million : 13C
169.137
154.292
144.996
130.579
128.806
128.415
128.120
126.165
125.383
124.420
114.275
113.236
112.025
110.128
77.415
77.100
76.776
64.123
56.505
56.200
54.360
46.685
14.458
13.905

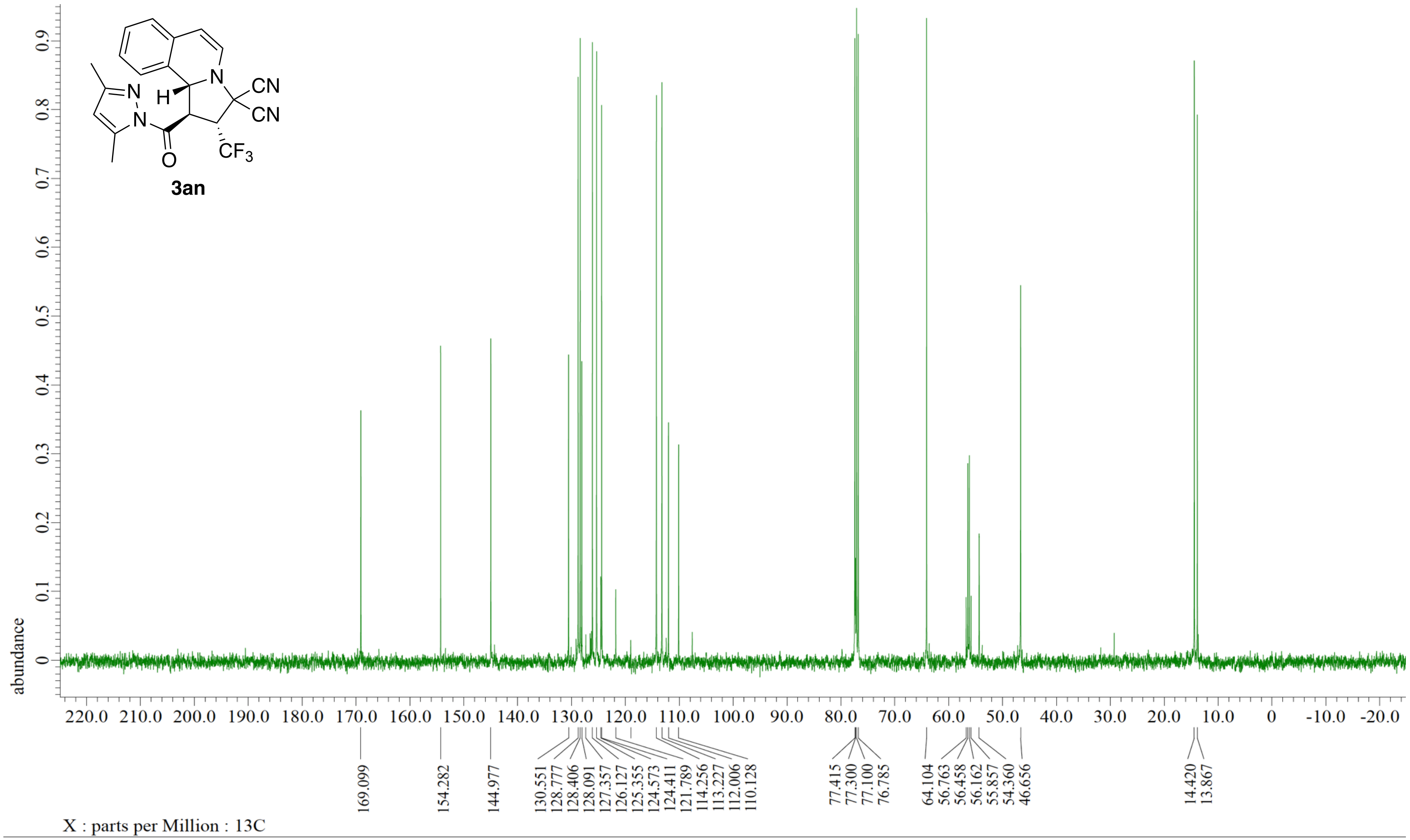
CN
CN
N
N
N
H
O
CF3
3an
abundance
0.9
0.8
0.7
0.6
0.5
0.4
0.3
0.2
0.1
0
220.0 210.0 200.0 190.0 180.0 170.0 160.0 150.0 140.0 130.0 120.0 110.0 100.0 90.0 80.0 70.0 60.0 50.0 40.0 30.0 20.0 10.0 0 -10.0 -20.0
169.099
154.282
144.977
130.551
128.777
128.406
128.091
127.357
126.127
125.355
124.573
124.411
121.789
114.256
113.227
112.006
110.128
77.415
77.300
77.100
76.785
64.104
56.763
56.458
56.162
55.857
54.360
46.656
14.420
13.867
X : parts per Million : 13C

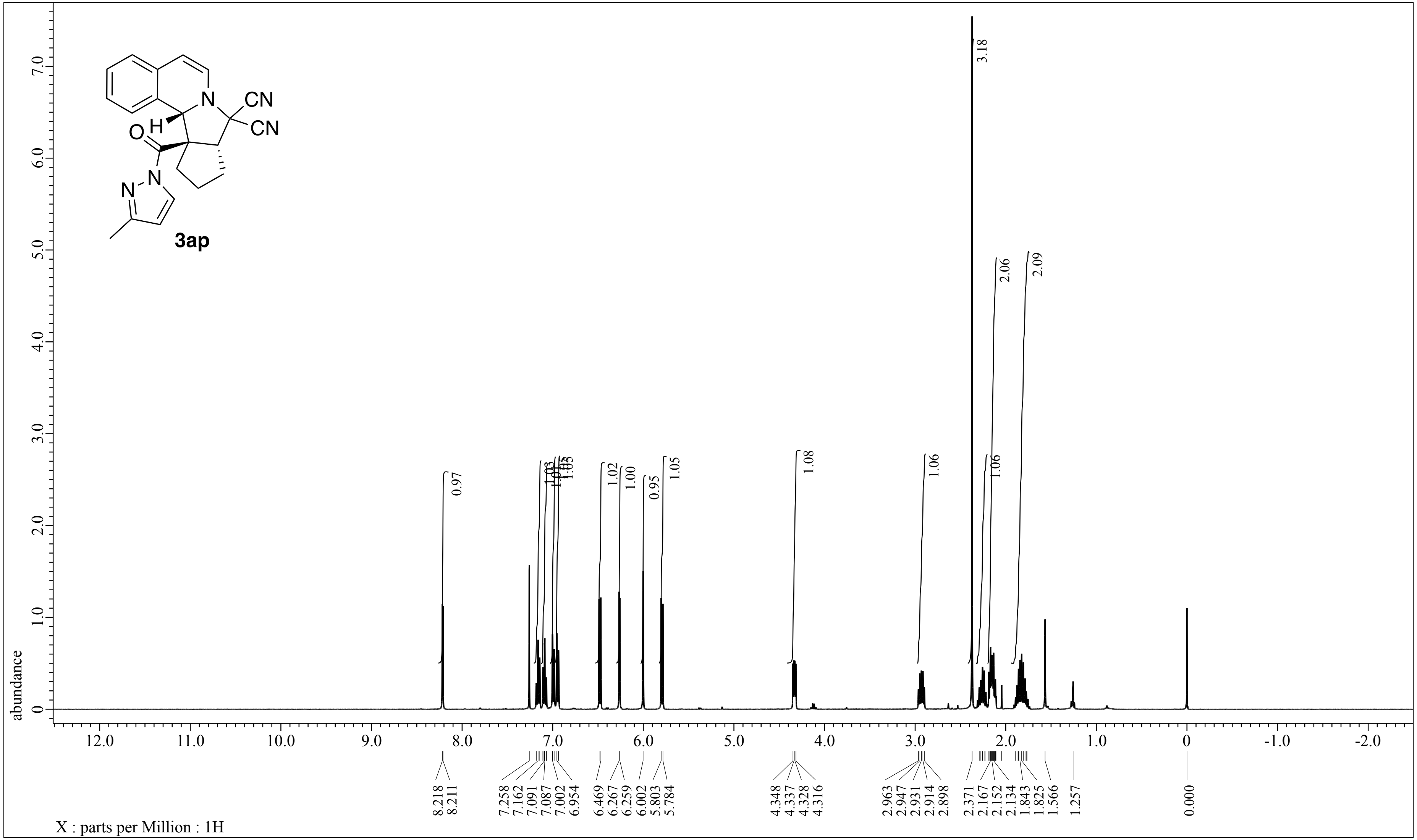

CN
CN
N
H
O
N
N
3ap
abundance
X : parts per Million : 1H
0.97
1.03
1.01
1.05
1.05
1.02
1.00
0.95
1.05
1.08
1.06
3.18
1.06
2.06
2.09
8.218
8.211
7.258
7.162
7.091
7.087
7.002
6.954
6.469
6.267
6.259
6.002
5.803
5.784
4.348
4.337
4.328
4.316
2.963
2.947
2.931
2.914
2.898
2.371
2.167
2.152
2.134
1.843
1.825
1.566
1.257
0.000

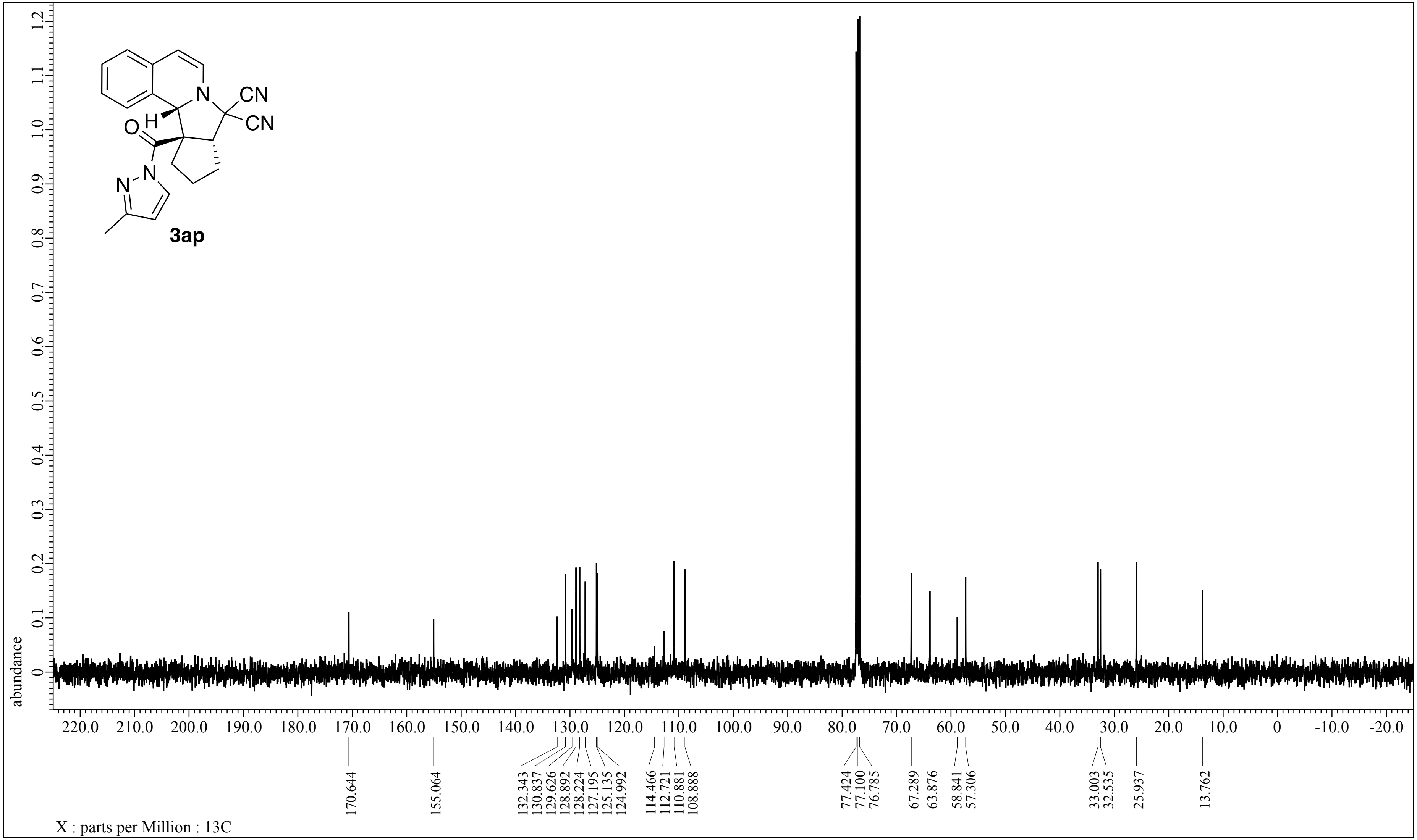

CN
CN
N
H
O
N
N
3ap
abundance
X : parts per Million : 13C
170.644
155.064
132.343
130.837
129.626
128.892
128.224
127.195
125.135
124.992
114.466
112.721
110.881
108.888
77.424
77.100
76.785
67.289
63.876
58.841
57.306
33.003
32.535
25.937
13.762

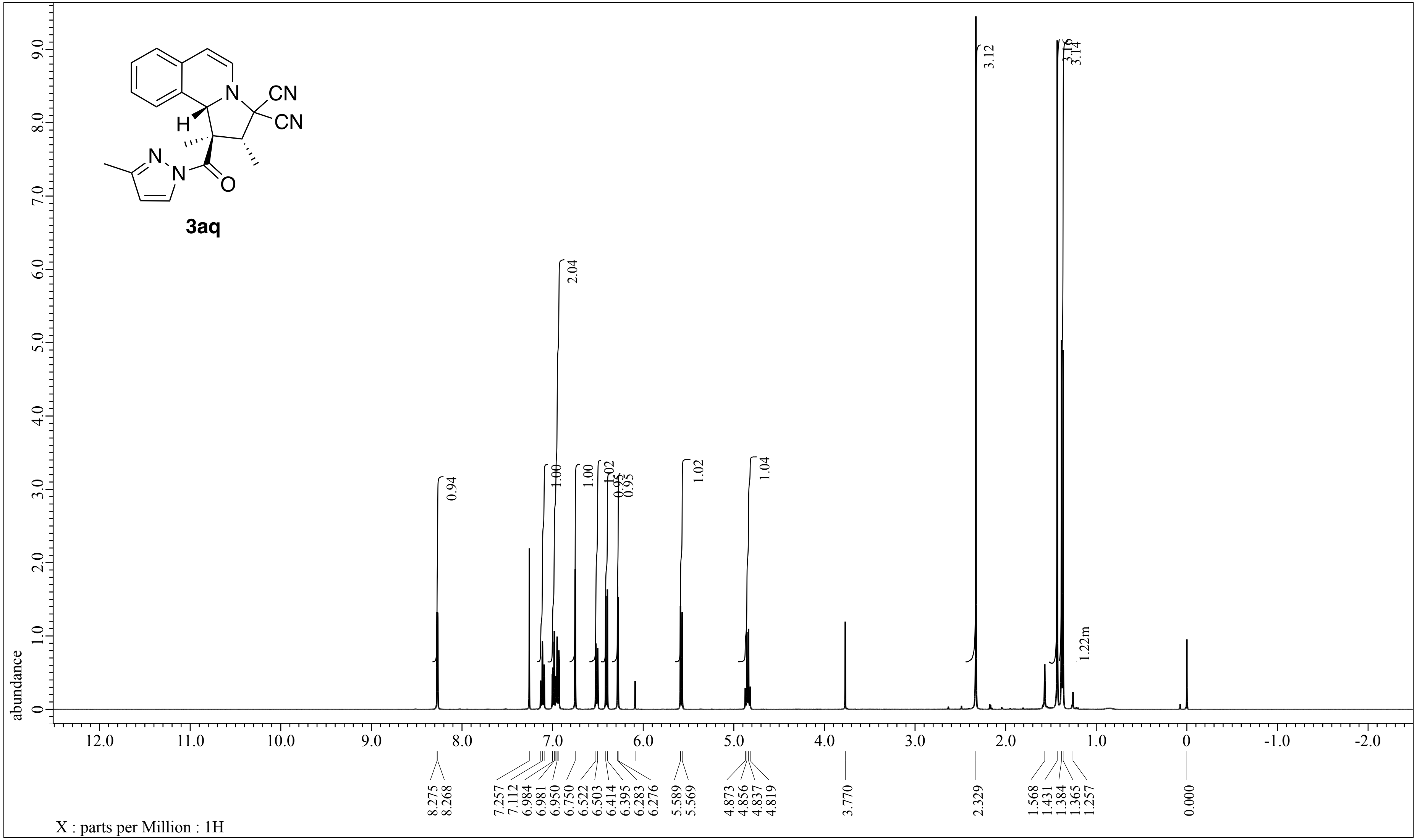
3aq
CN
CN
N
H
N
N
O
abundance
X : parts per Million : 1H
0.94
1.00
1.00
1.02
0.95
0.95
2.04
1.02
1.04
3.12
3.16
3.14
1.22m
8.275
8.268
7.257
7.112
6.984
6.981
6.950
6.750
6.522
6.503
6.414
6.395
6.283
6.276
5.589
5.569
4.873
4.856
4.837
4.819
3.770
2.329
1.568
1.431
1.384
1.365
1.257
0.000

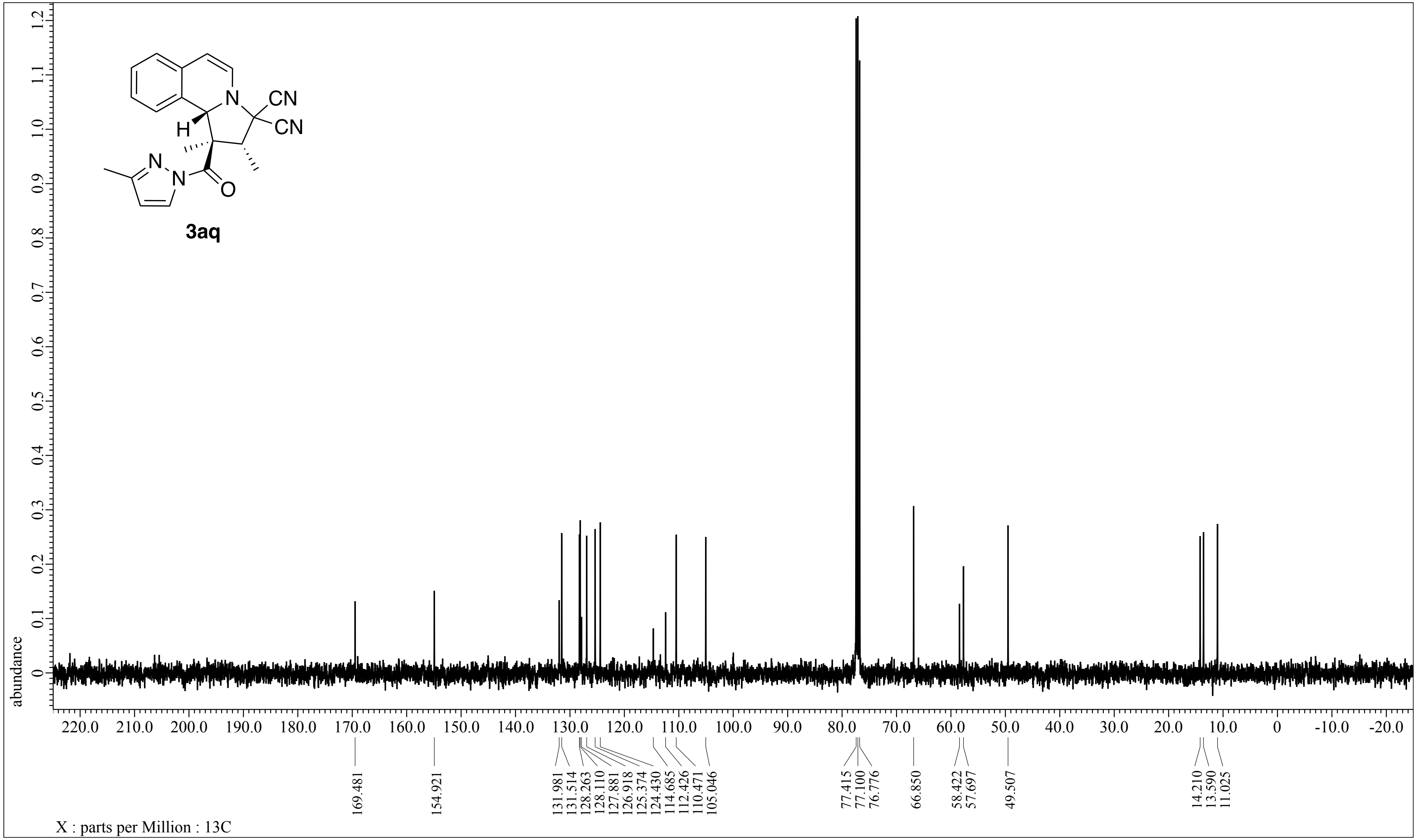
CN
CN
N
H
N
N
O
3aq
abundance
X : parts per Million : 13C
169.481
154.921
131.981
131.514
128.263
128.110
127.881
126.918
125.374
124.430
114.685
112.426
110.471
105.046
77.415
77.100
76.776
66.850
58.422
57.697
49.507
14.210
13.590
11.025

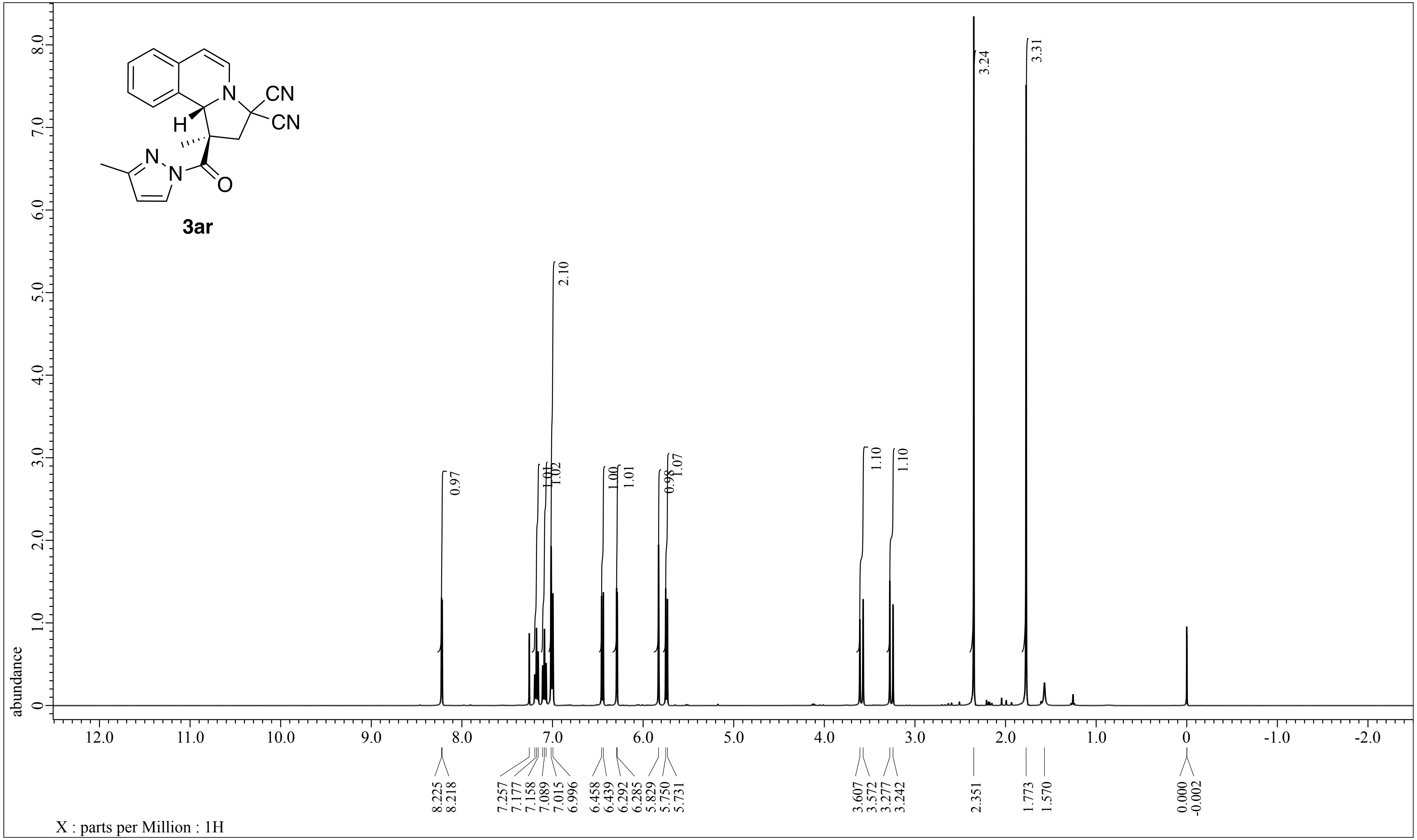

3ar
CN
CN
N
H
N
N
O
abundance
X : parts per Million : 1H
0.97
1.01
1.02
2.10
1.00
1.01
0.98
1.07
1.10
1.10
3.24
3.31
8.225
8.218
7.257
7.177
7.158
7.089
7.015
6.996
6.458
6.439
6.292
6.285
5.829
5.750
5.731
3.607
3.572
3.277
3.242
2.351
1.773
1.570
0.000
-0.002

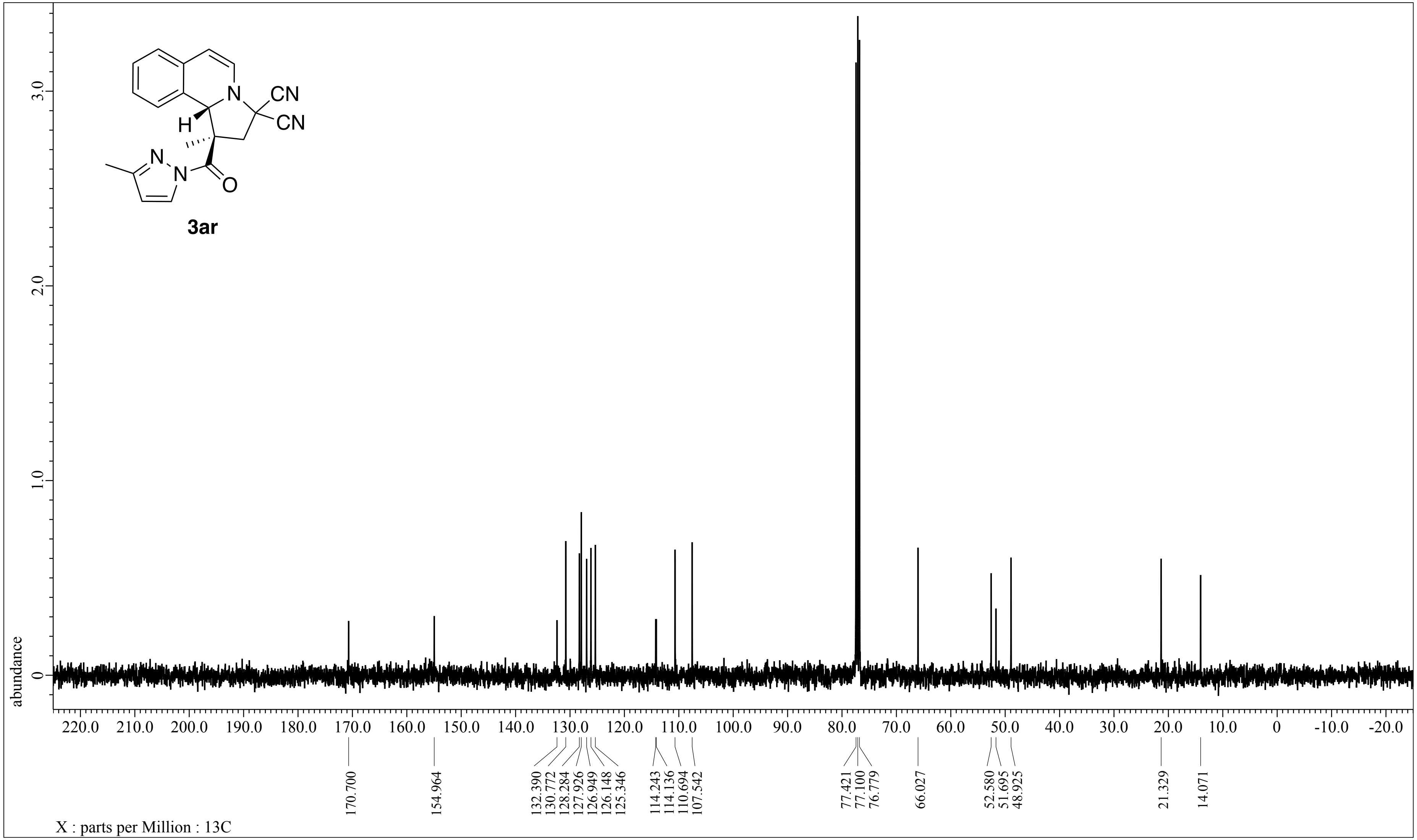

CN
CN
N
H
N
N
O
3ar
abundance
3.0
2.0
1.0
0
220.0 210.0 200.0 190.0 180.0 170.0 160.0 150.0 140.0 130.0 120.0 110.0 100.0 90.0 80.0 70.0 60.0 50.0 40.0 30.0 20.0 10.0 0 -10.0 -20.0
170.700
154.964
132.390
130.772
128.284
127.926
126.949
126.148
125.346
114.243
114.136
110.694
107.542
77.421
77.100
76.779
66.027
52.580
51.695
48.925
21.329
14.071
X : parts per Million : 13C

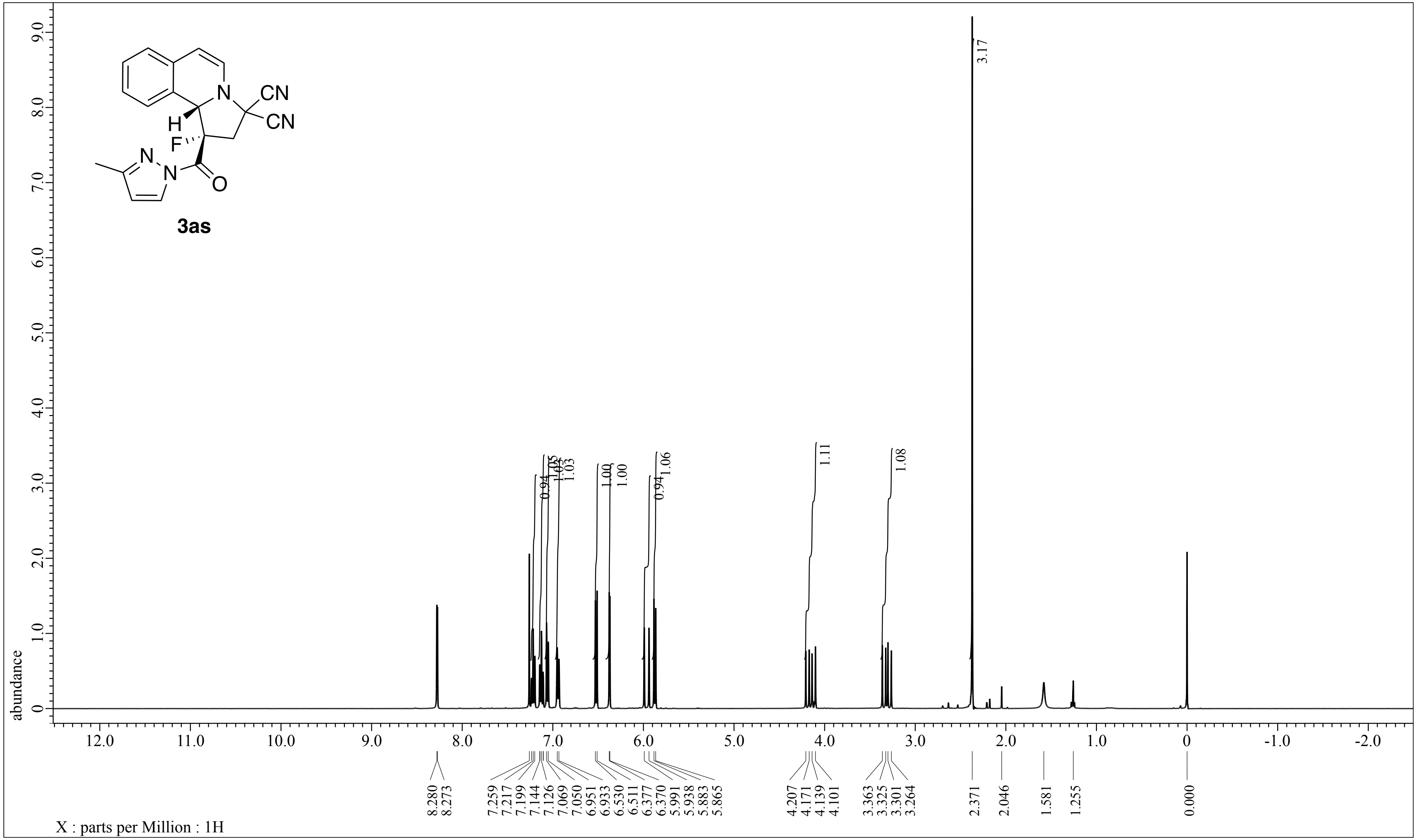
3as
CN
CN
N
H
F
N
N
O
abundance
X : parts per Million : 1H
8.280
8.273
7.259
7.217
7.199
7.144
7.126
7.069
7.050
6.951
6.933
6.530
6.511
6.377
6.370
5.991
5.938
5.883
5.865
4.207
4.171
4.139
4.101
3.363
3.325
3.301
3.264
2.371
2.046
1.581
1.255
0.000
3.17
1.11
1.08
1.06
0.94
1.00
1.00
0.94
1.05
1.02
1.03

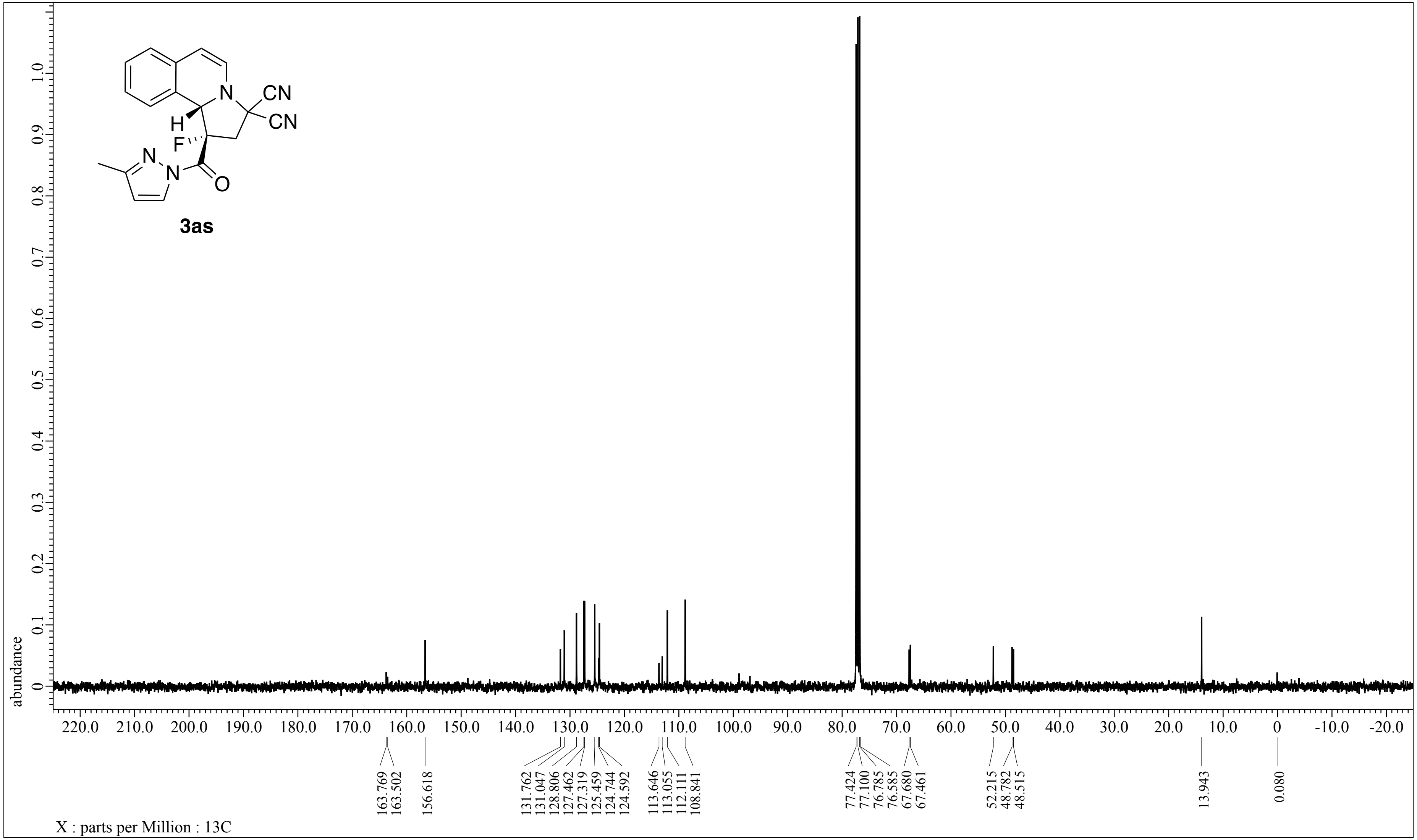

CN
CN
N
H
F
N
N
O
3as
abundance
X : parts per Million : 13C
163.769
163.502
156.618
131.762
131.047
128.806
127.462
127.319
125.459
124.744
124.592
113.646
113.055
112.111
108.841
77.424
77.100
76.785
76.585
67.680
67.461
52.215
48.782
48.515
13.943
0.080

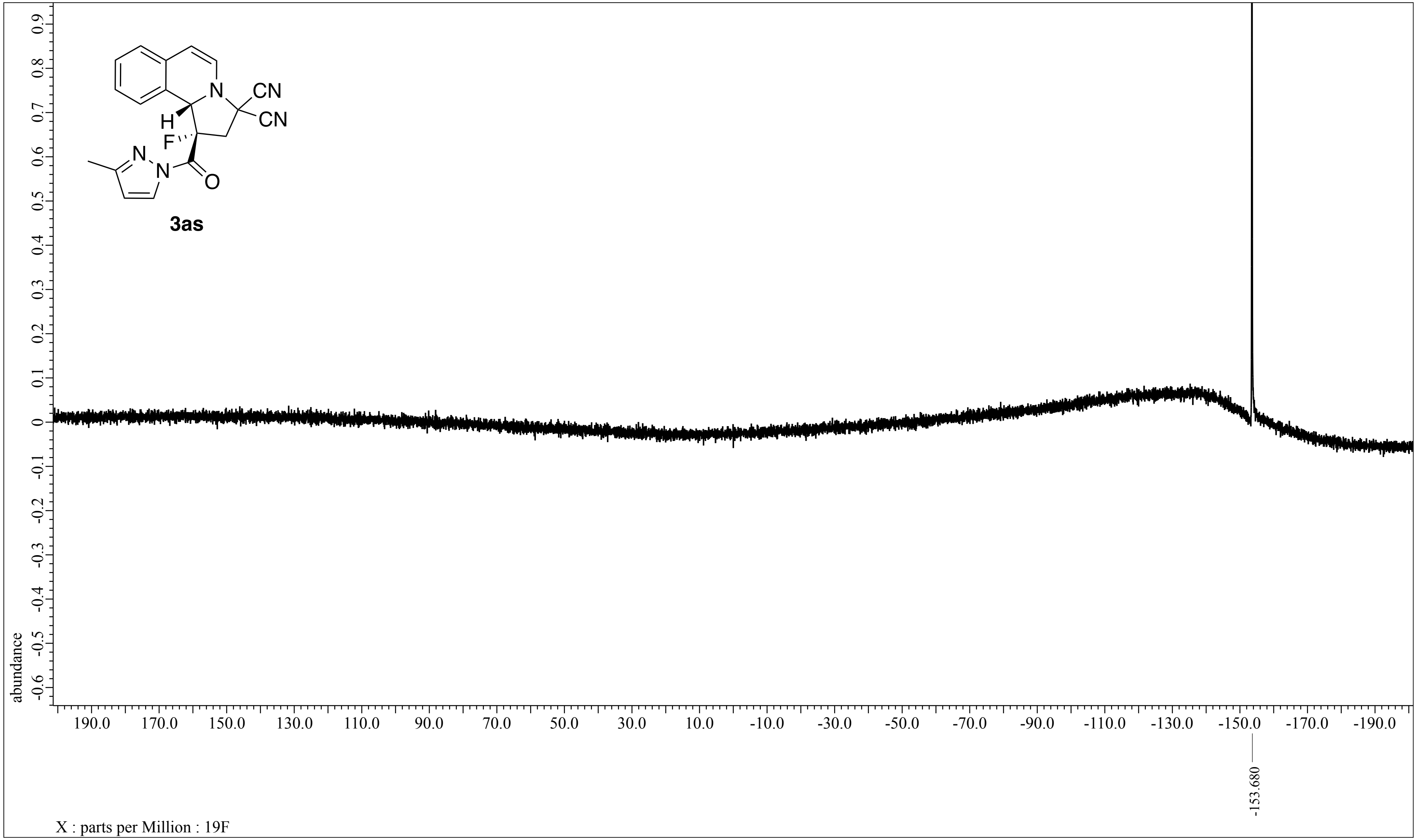

3as
abundance
0.9
0.8
0.7
0.6
0.5
0.4
0.3
0.2
0.1
0
-0.1
-0.2
-0.3
-0.4
-0.5
-0.6
190.0
170.0
150.0
130.0
110.0
90.0
70.0
50.0
30.0
10.0
-10.0
-30.0
-50.0
-70.0
-90.0
-110.0
-130.0
-150.0
-170.0
-190.0
-153.680
X : parts per Million : 19F

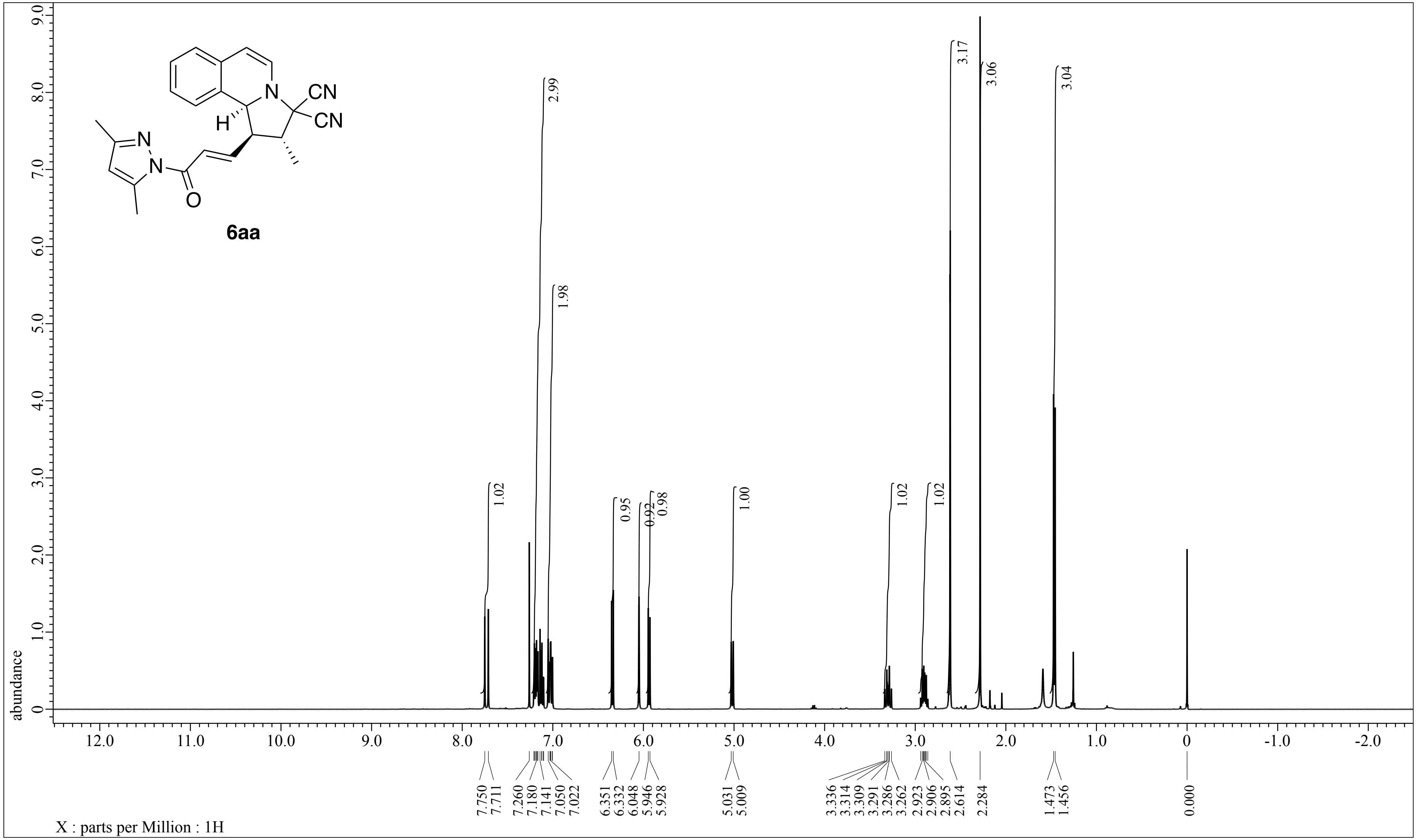

6aa
abundance
X : parts per Million : 1H
1.02
2.99
1.98
0.95
0.92
0.98
1.00
1.02
1.02
3.17
3.06
3.04
7.750
7.711
7.260
7.180
7.141
7.050
7.022
6.351
6.332
6.048
5.946
5.928
5.031
5.009
3.336
3.314
3.309
3.291
3.286
3.262
2.923
2.906
2.895
2.614
2.284
1.473
1.456
0.000

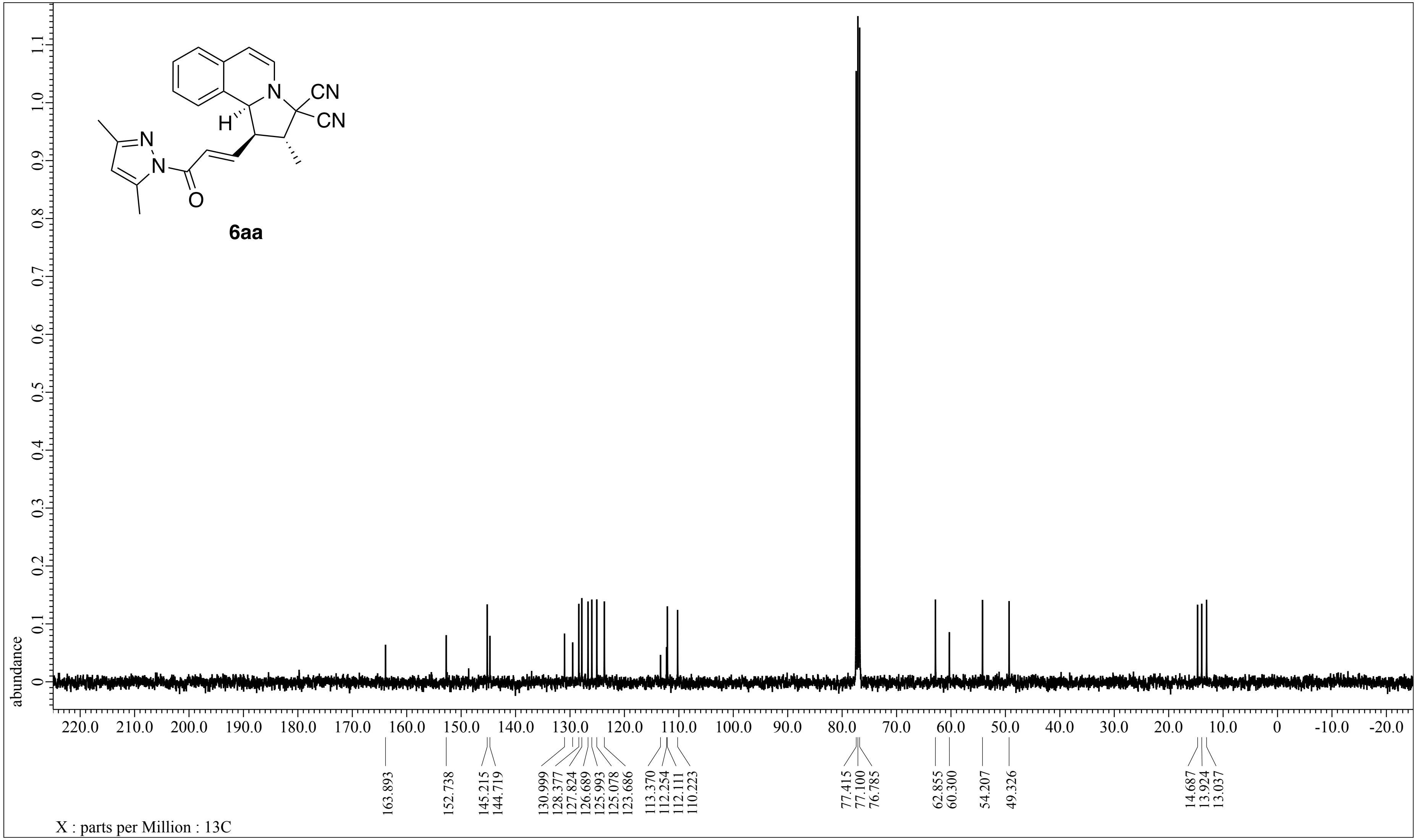
N
N
N
CN
CN
H
O
6aa
abundance
X : parts per Million : 13C
163.893
152.738
145.215
144.719
130.999
128.377
127.824
126.689
125.993
125.078
123.686
113.370
112.254
112.111
110.223
77.415
77.100
76.785
62.855
60.300
54.207
49.326
14.687
13.924
13.037

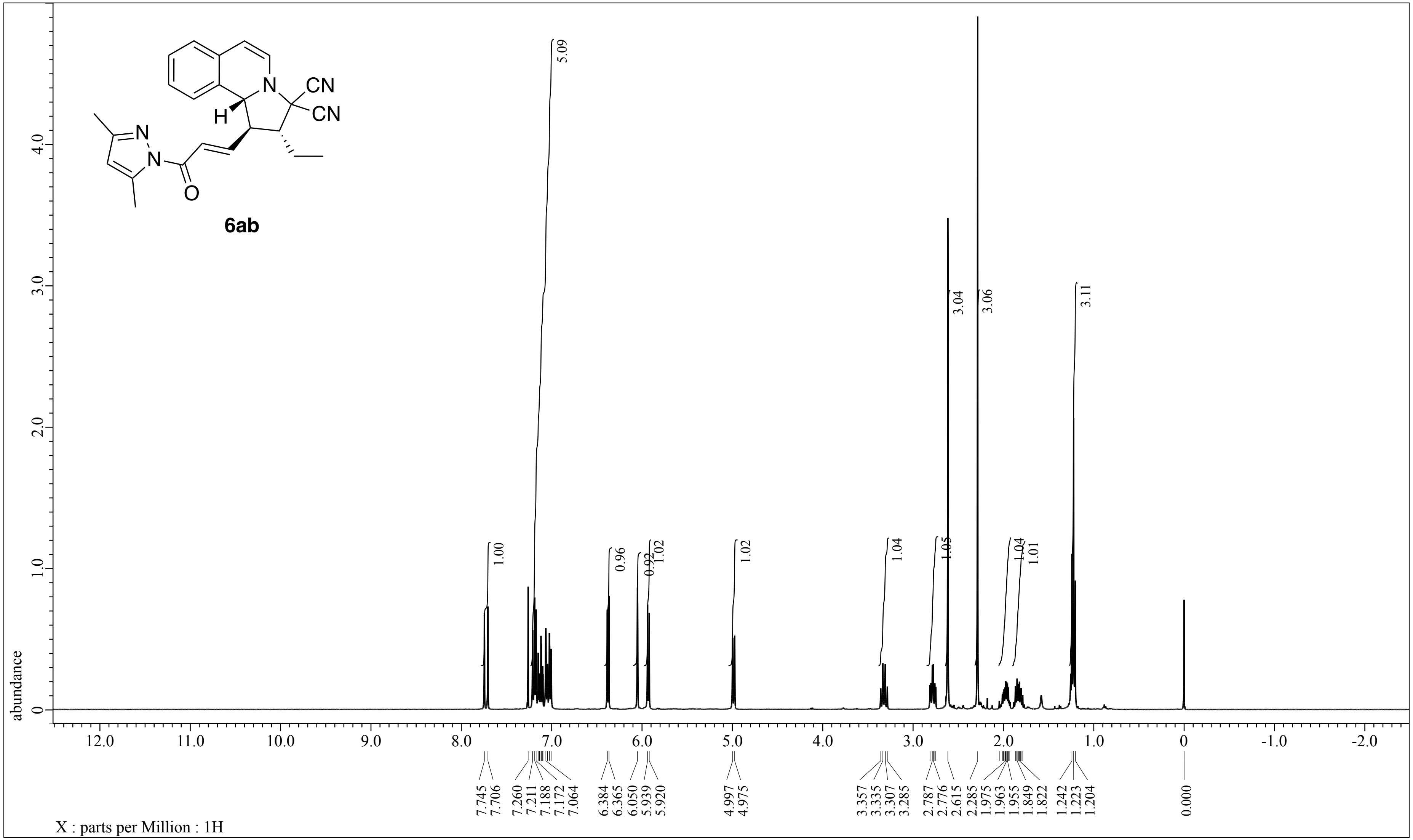
6ab
abundance
X : parts per Million : 1H
7.745
7.706
7.260
7.211
7.188
7.172
7.064
6.384
6.365
6.050
5.939
5.920
4.997
4.975
3.357
3.335
3.307
3.285
2.787
2.776
2.615
2.285
1.975
1.963
1.955
1.849
1.822
1.242
1.223
1.204
0.000
1.00
5.09
0.96
0.92
1.02
1.02
1.04
1.05
3.04
3.06
1.04
1.01
3.11

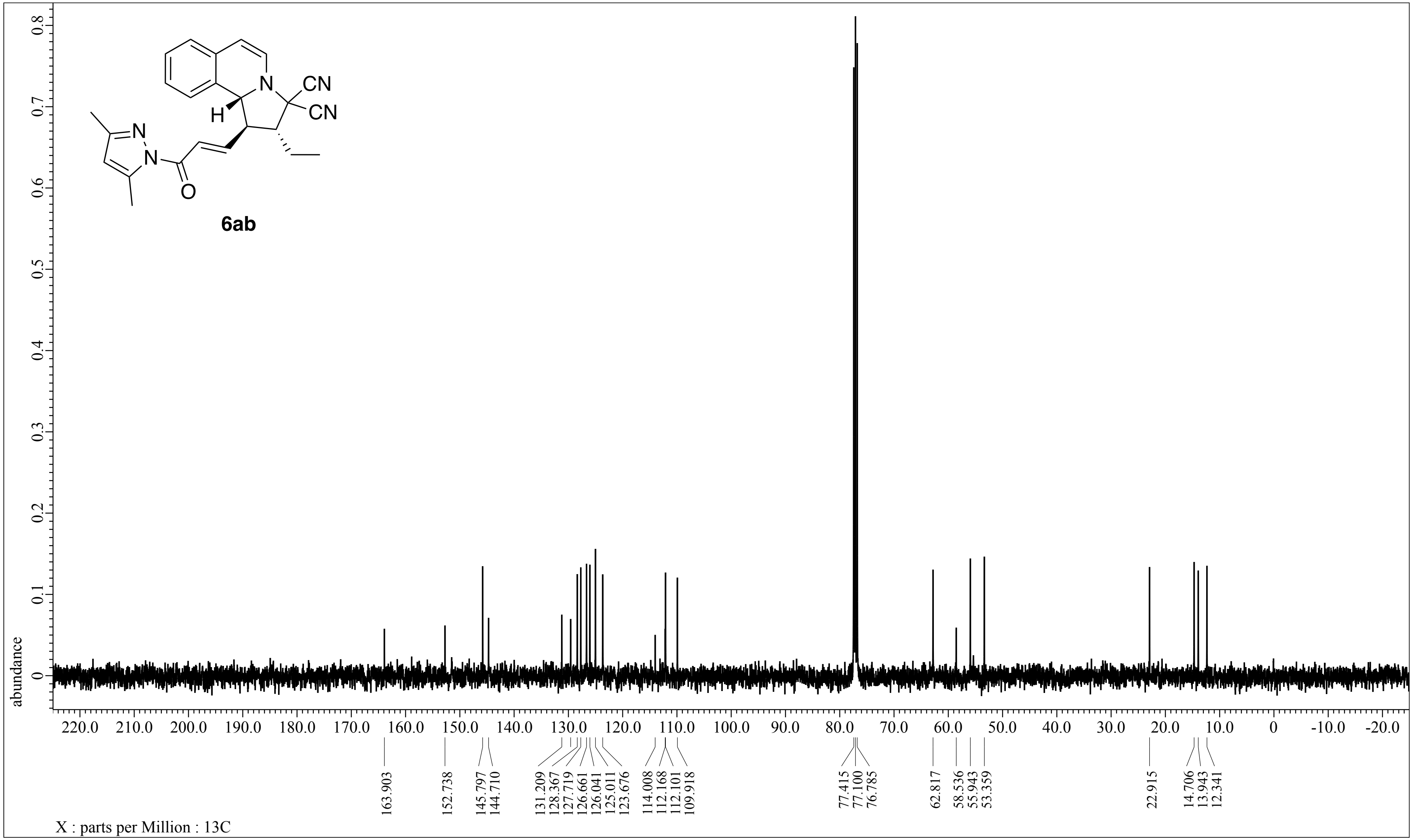
6ab
CN
CN
N
H
N
N
O
abundance
X : parts per Million : 13C
163.903
152.738
145.797
144.710
131.209
128.367
127.719
126.661
126.041
125.011
123.676
114.008
112.168
112.101
109.918
77.415
77.100
76.785
62.817
58.536
55.943
53.359
22.915
14.706
13.943
12.341

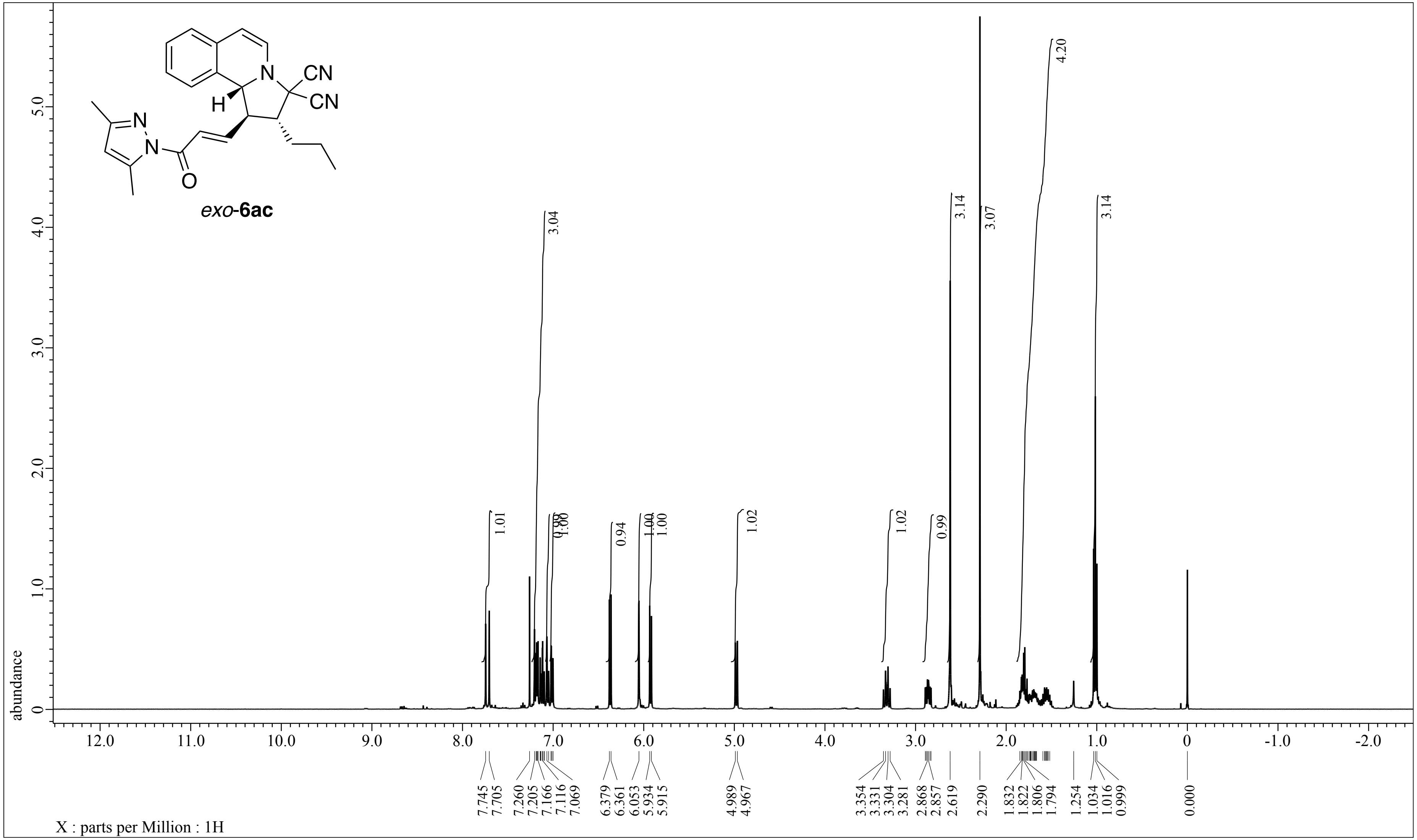

CN
CN
N
H
N
N
O
exo-6ac
5.0
4.0
3.0
2.0
1.0
0
abundance
12.0
11.0
10.0
9.0
8.0
7.0
6.0
5.0
4.0
3.0
2.0
1.0
0
-1.0
-2.0
X : parts per Million : 1H
1.01
3.04
0.99
1.00
0.94
1.00
1.00
1.02
1.02
0.99
3.14
3.07
4.20
3.14
7.745
7.705
7.260
7.205
7.166
7.116
7.069
6.379
6.361
6.053
5.934
5.915
4.989
4.967
3.354
3.331
3.304
3.281
2.868
2.857
2.619
2.290
1.832
1.822
1.806
1.794
1.254
1.034
1.016
0.999
0.000

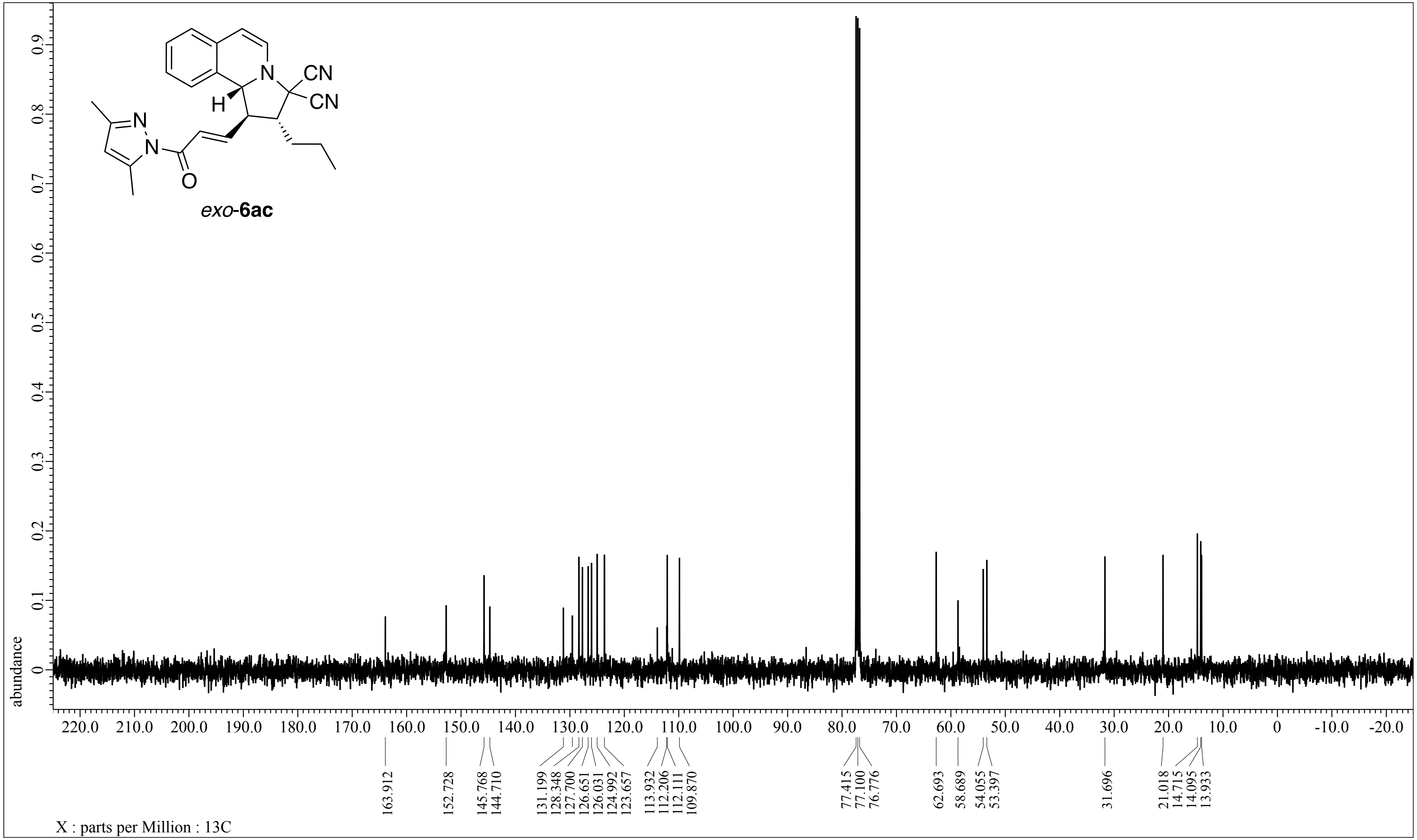
CN
CN
N
H
N
N
O
exo-6ac
abundance
0
0.1
0.2
0.3
0.4
0.5
0.6
0.7
0.8
0.9
220.0 210.0 200.0 190.0 180.0 170.0 160.0 150.0 140.0 130.0 120.0 110.0 100.0 90.0 80.0 70.0 60.0 50.0 40.0 30.0 20.0 10.0 0 -10.0 -20.0
163.912
152.728
145.768
144.710
131.199
128.348
127.700
126.651
126.031
124.992
123.657
113.932
112.206
112.111
109.870
77.415
77.100
76.776
62.693
58.689
54.055
53.397
31.696
21.018
14.715
14.095
13.933
X : parts per Million : 13C

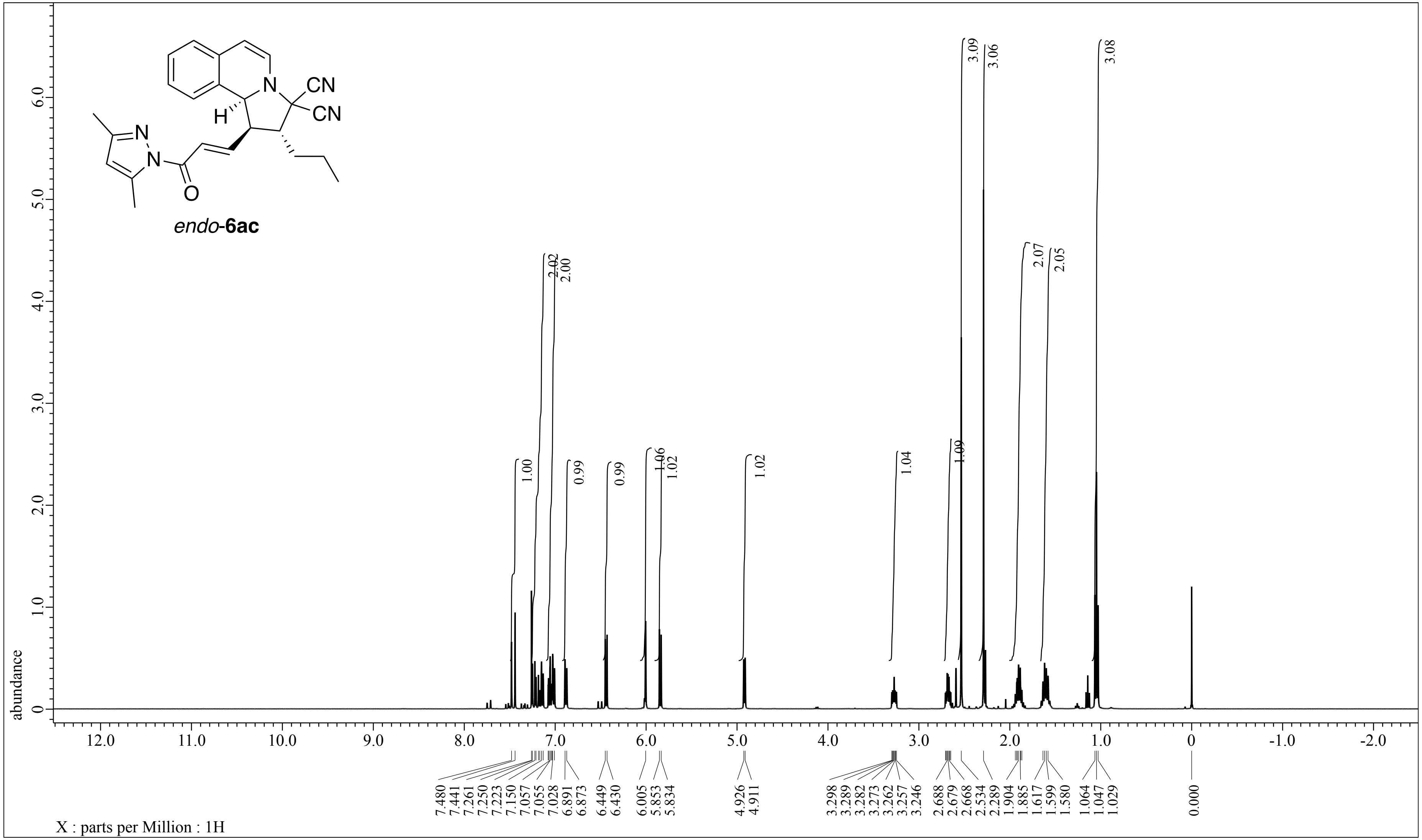
endo-6ac
abundance
X : parts per Million : 1H
1.00
2.02
2.00
0.99
0.99
1.06
1.02
1.02
1.04
1.09
3.09
3.06
2.07
2.05
3.08
7.480
7.441
7.261
7.250
7.223
7.150
7.057
7.055
7.028
6.891
6.873
6.449
6.430
6.005
5.853
5.834
4.926
4.911
3.298
3.289
3.282
3.273
3.262
3.257
3.246
2.688
2.679
2.668
2.534
2.289
1.904
1.885
1.617
1.599
1.580
1.064
1.047
1.029
0.000

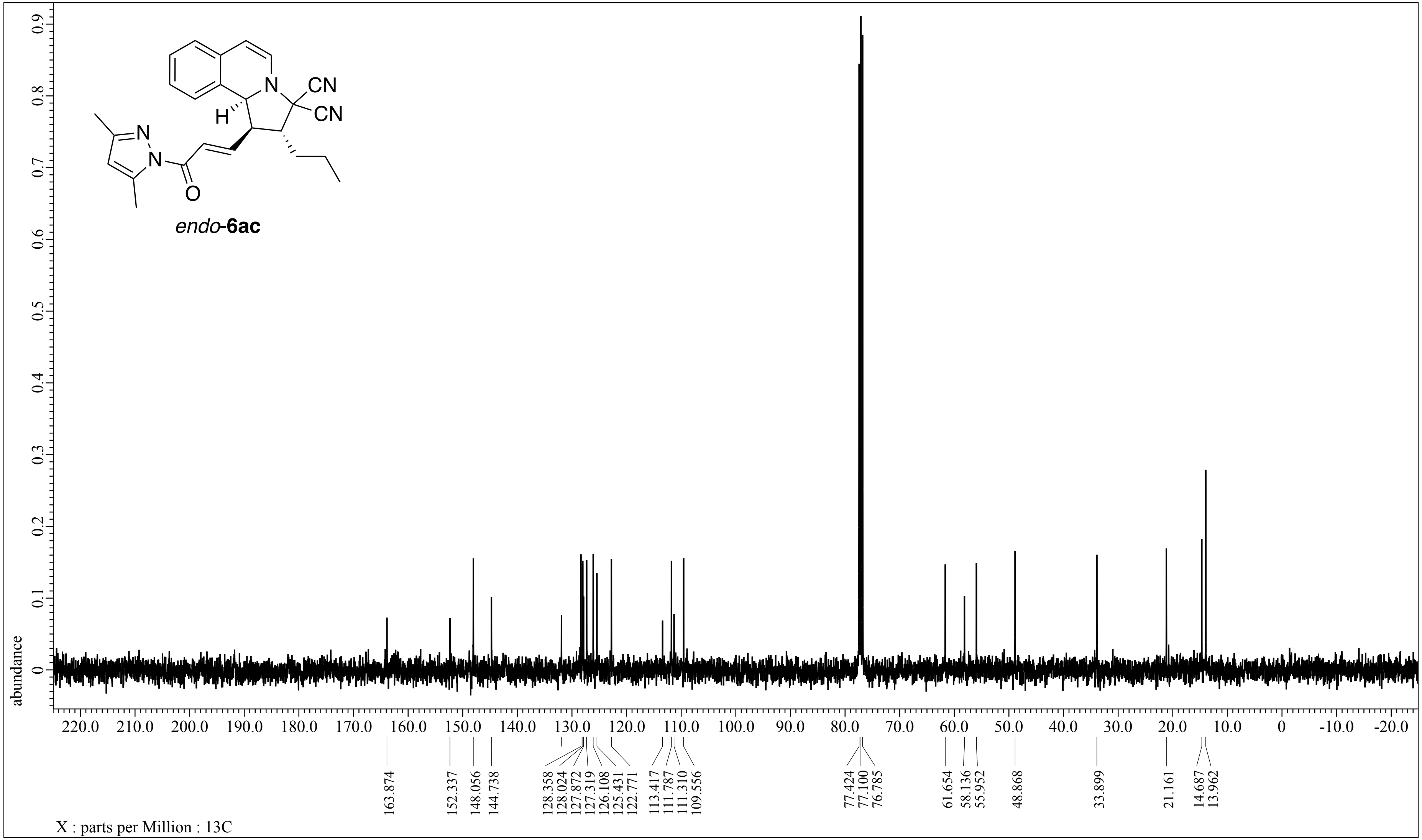
CN
CN
N
H
N
N
O
endo-6ac
abundance
X : parts per Million : 13C
163.874
152.337
148.056
144.738
128.358
128.024
127.872
127.319
126.108
125.431
122.771
113.417
111.787
111.310
109.556
77.424
77.100
76.785
61.654
58.136
55.952
48.868
33.899
21.161
14.687
13.962

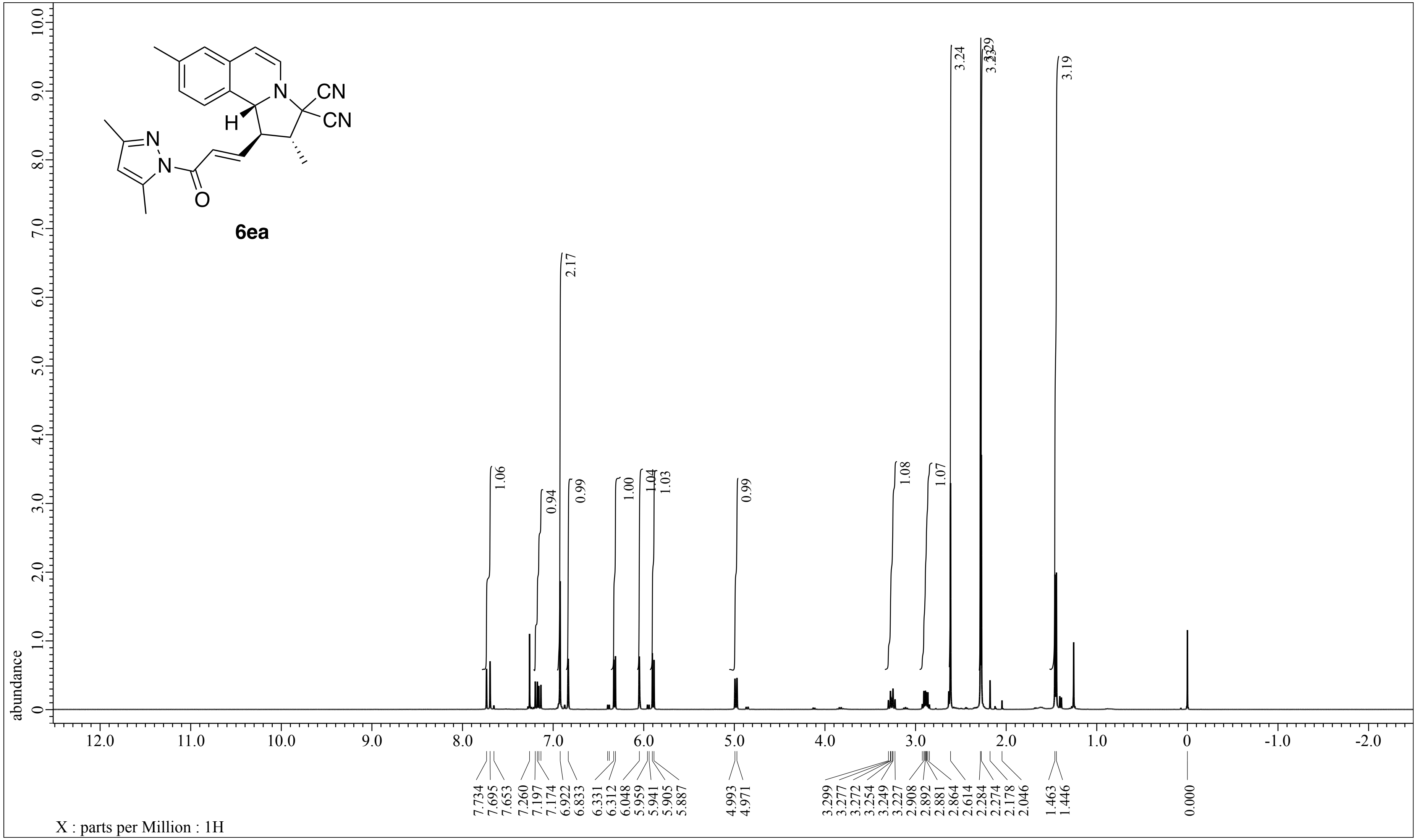

6ea
CN
CN
H
N
N
N
O
abundance
X : parts per Million : 1H
1.06
0.94
0.99
2.17
1.00
1.04
1.03
0.99
1.08
1.07
3.24
3.29
3.23
3.19
7.734
7.695
7.653
7.260
7.197
7.174
6.922
6.833
6.331
6.312
6.048
5.959
5.941
5.905
5.887
4.993
4.971
3.299
3.277
3.272
3.254
3.249
3.227
2.908
2.892
2.881
2.864
2.614
2.284
2.274
2.178
2.046
1.463
1.446
0.000

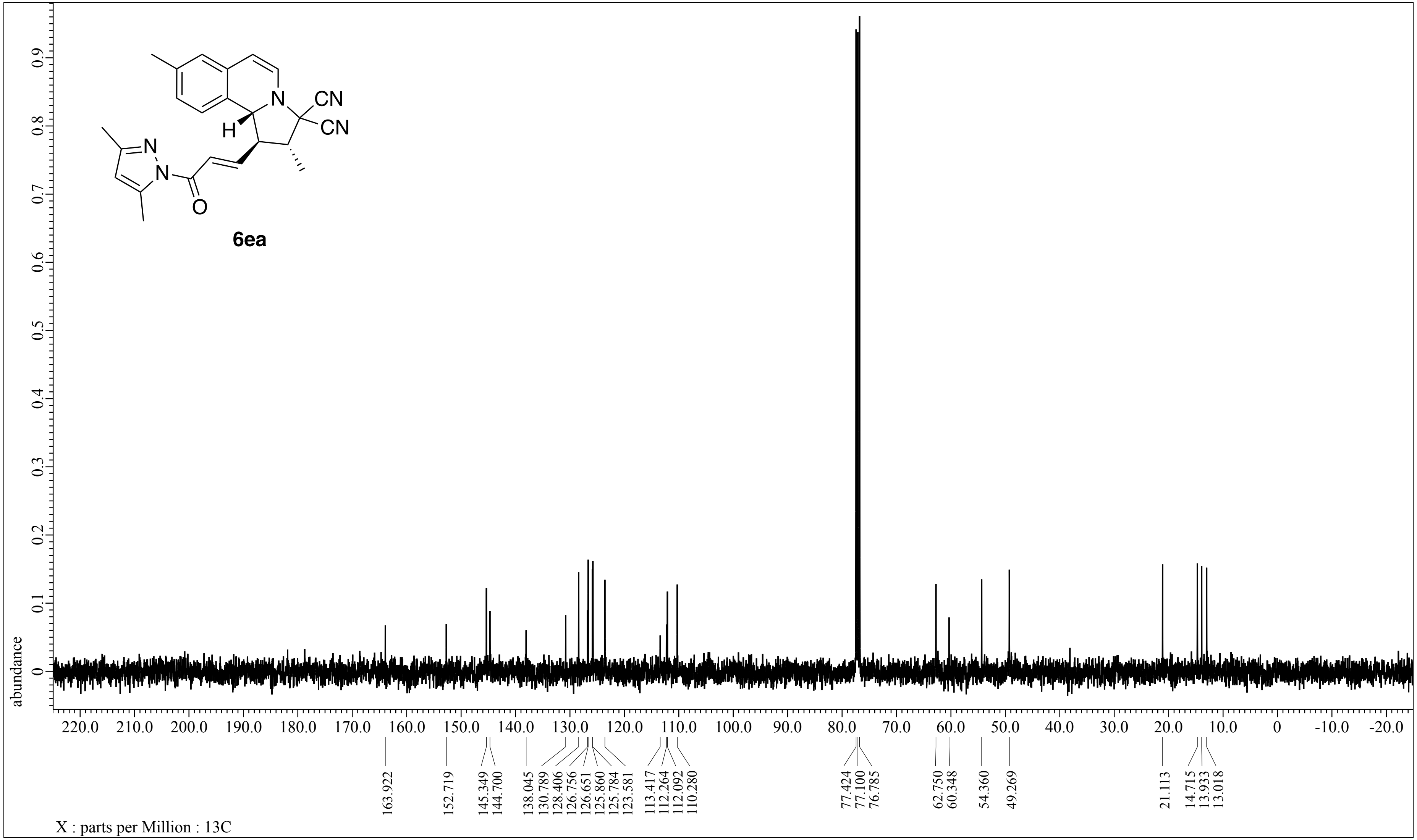
CN
CN
N
H
N
N
O
6ea
abundance
X : parts per Million : 13C
163.922
152.719
145.349
144.700
138.045
130.789
128.406
126.756
126.651
125.860
125.784
123.581
113.417
112.264
112.092
110.280
77.424
77.100
76.785
62.750
60.348
54.360
49.269
21.113
14.715
13.933
13.018

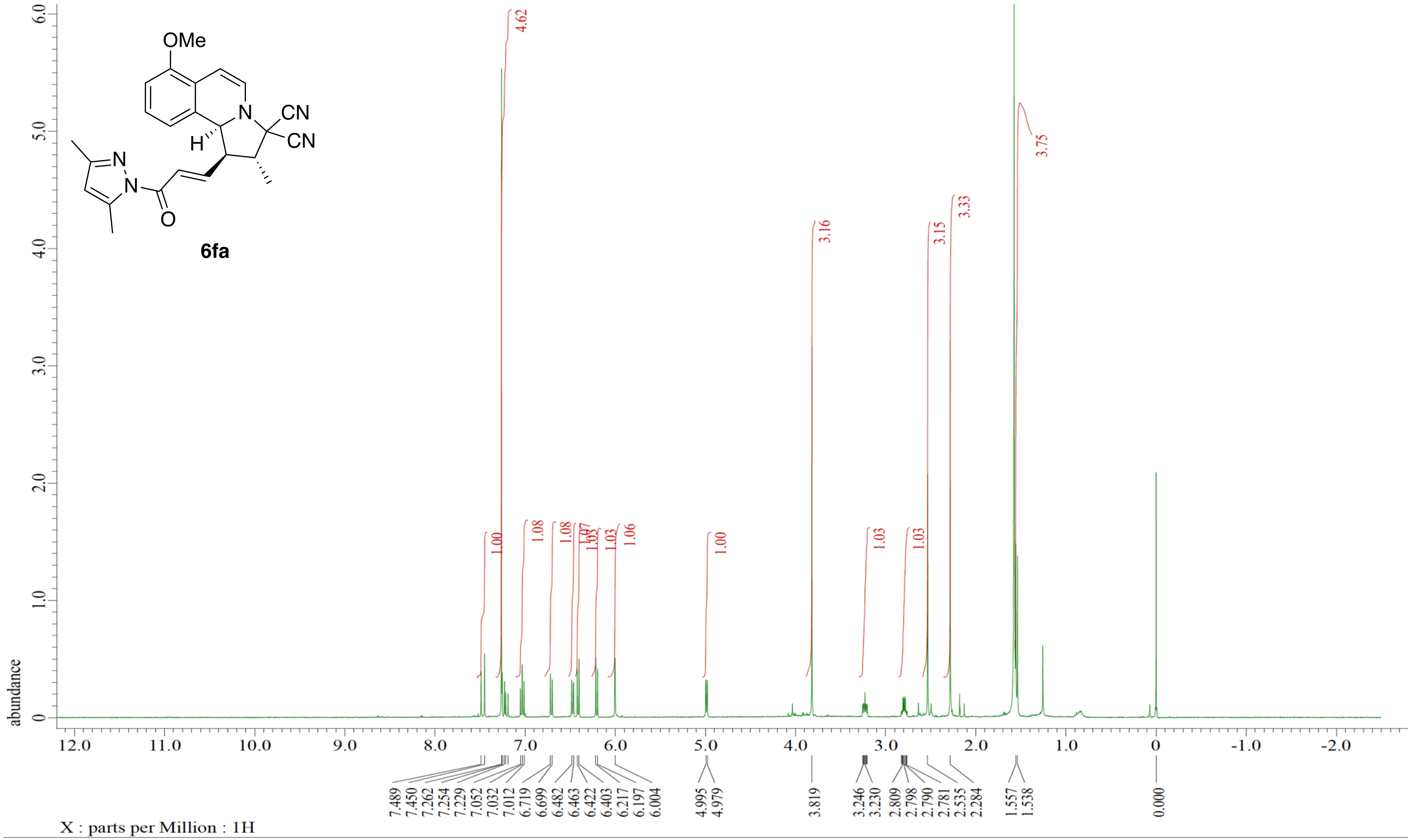
OMe
N
CN
CN
H
N
N
O
6fa
abundance
X : parts per Million : 1H
7.489
7.450
7.262
7.254
7.229
7.052
7.032
7.012
6.719
6.699
6.482
6.463
6.422
6.403
6.217
6.197
6.004
4.995
4.979
3.819
3.246
3.230
2.809
2.798
2.790
2.781
2.535
2.284
1.557
1.538
0.000

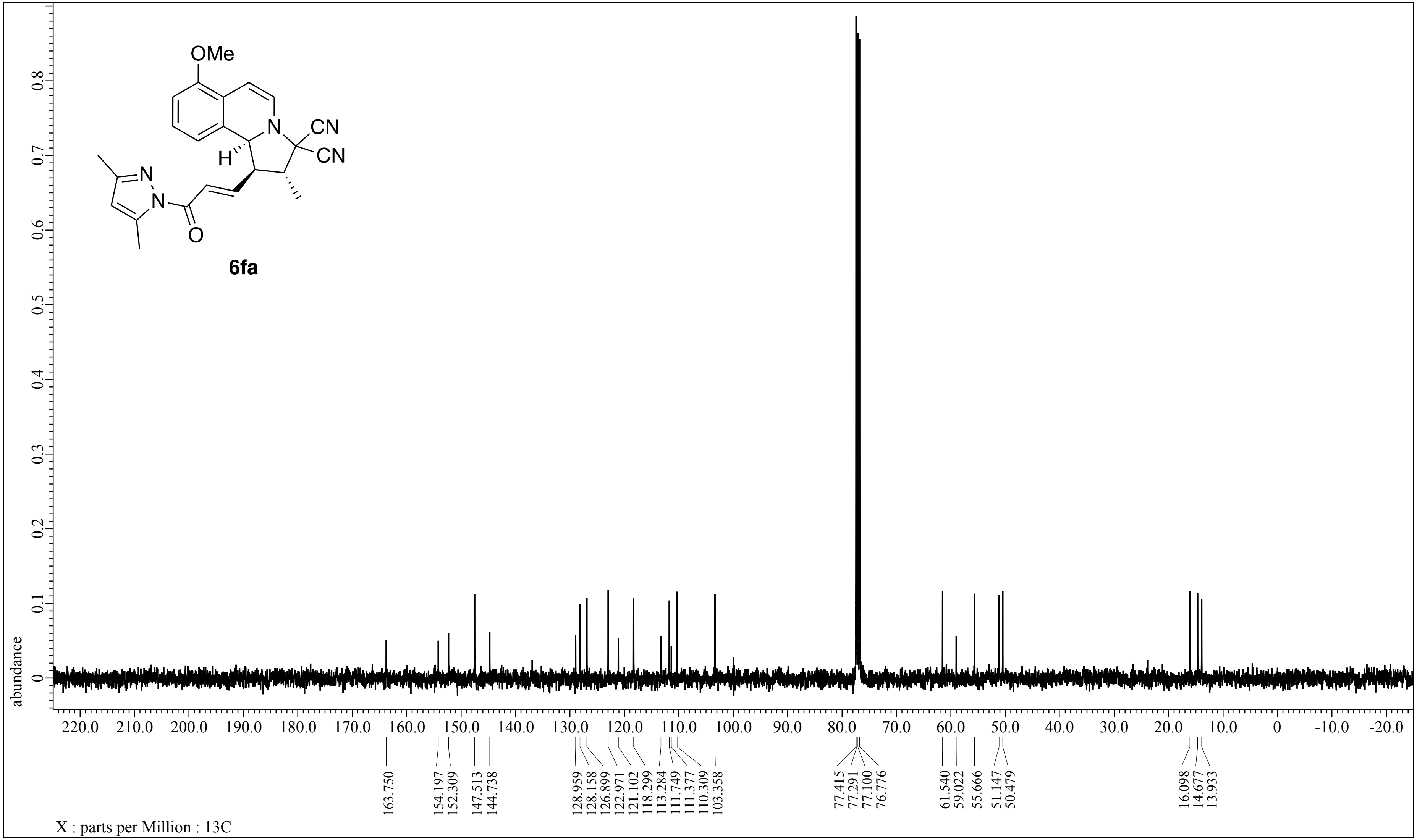
OMe
N
CN
CN
H
N
N
O
6fa
abundance
0
0.1
0.2
0.3
0.4
0.5
0.6
0.7
0.8
220.0 210.0 200.0 190.0 180.0 170.0 160.0 150.0 140.0 130.0 120.0 110.0 100.0 90.0 80.0 70.0 60.0 50.0 40.0 30.0 20.0 10.0 0 -10.0 -20.0
163.750
154.197
152.309
147.513
144.738
128.959
128.158
126.899
122.971
121.102
118.299
113.284
111.749
111.377
110.309
103.358
77.415
77.291
77.100
76.776
61.540
59.022
55.666
51.147
50.479
16.098
14.677
13.933
X : parts per Million : 13C

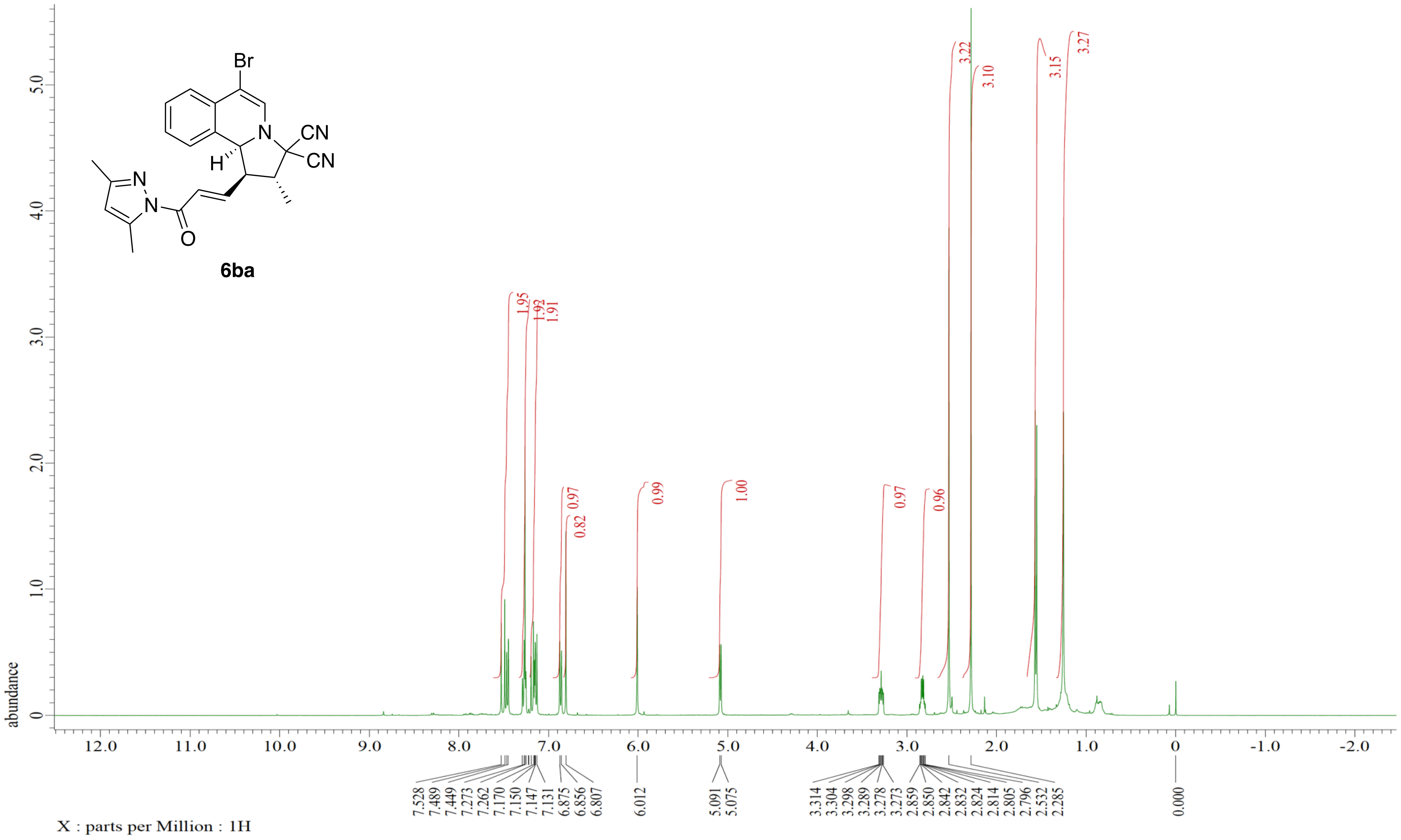
Br
N
CN
CN
H
N
N
O
6ba
abundance
5.0
4.0
3.0
2.0
1.0
0
12.0
11.0
10.0
9.0
8.0
7.0
6.0
5.0
4.0
3.0
2.0
1.0
0
-1.0
-2.0
1.95
1.92
1.91
0.97
0.82
0.99
1.00
0.97
0.96
3.22
3.10
3.15
3.27
7.528
7.489
7.449
7.273
7.262
7.170
7.150
7.147
7.131
6.875
6.856
6.807
6.012
5.091
5.075
3.314
3.304
3.298
3.289
3.278
3.273
2.859
2.850
2.842
2.832
2.824
2.814
2.805
2.796
2.532
2.285
0.000
X : parts per Million : 1H

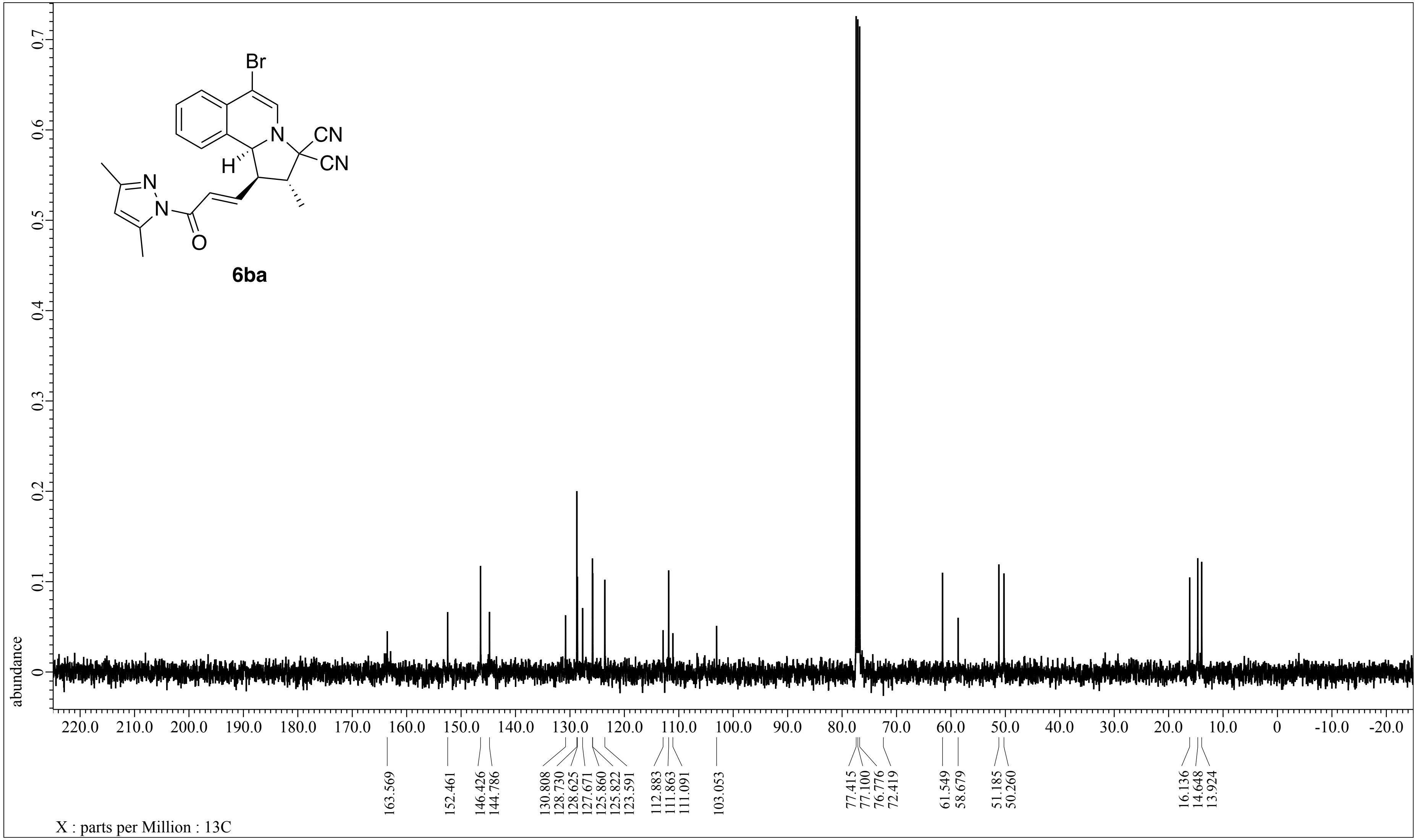

Br
N
CN
CN
H
N
N
O
6ba
abundance
X : parts per Million : 13C
163.569
152.461
146.426
144.786
130.808
128.730
128.625
127.671
125.860
125.822
123.591
112.883
111.863
111.091
103.053
77.415
77.100
76.776
72.419
61.549
58.679
51.185
50.260
16.136
14.648
13.924

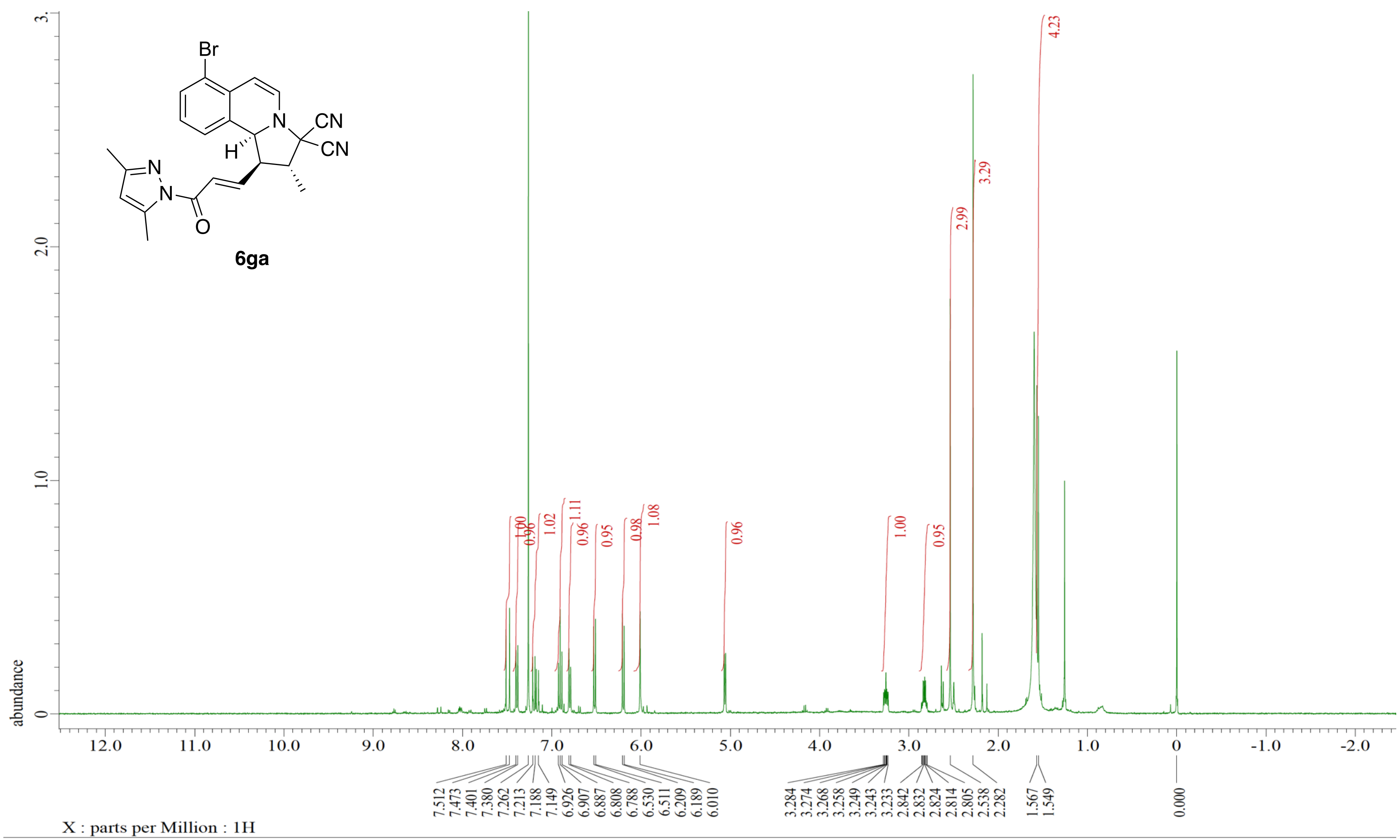

Br
N
CN
CN
H
N
N
O
6ga
abundance
X : parts per Million : 1H

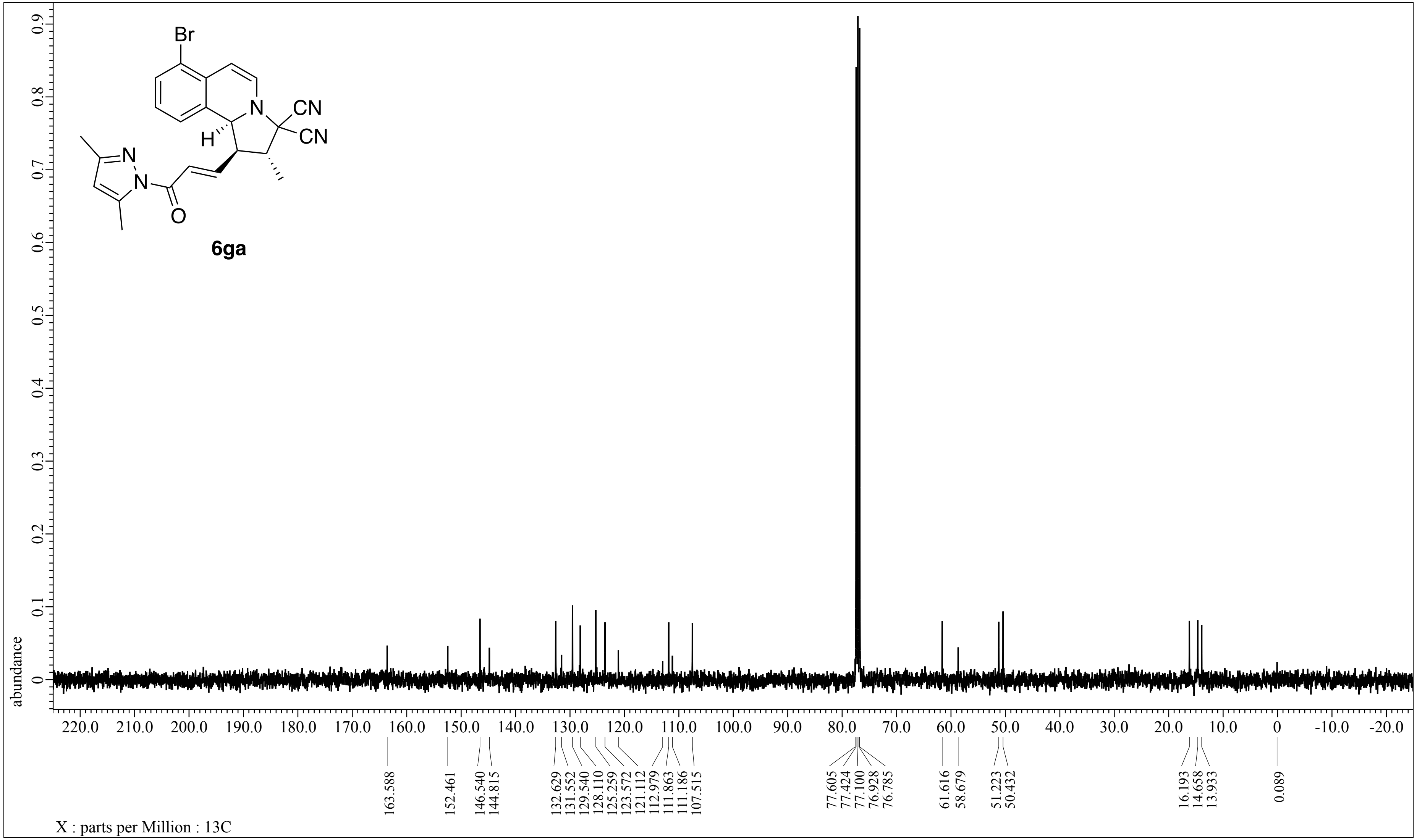
Br
N
CN
CN
H
N
N
O
6ga
abundance
0.9
0.8
0.7
0.6
0.5
0.4
0.3
0.2
0.1
0
220.0 210.0 200.0 190.0 180.0 170.0 160.0 150.0 140.0 130.0 120.0 110.0 100.0 90.0 80.0 70.0 60.0 50.0 40.0 30.0 20.0 10.0 0 -10.0 -20.0
163.588
152.461
146.540
144.815
132.629
131.552
129.540
128.110
125.259
123.572
121.112
112.979
111.863
111.186
107.515
77.605
77.424
77.100
76.928
76.785
61.616
58.679
51.223
50.432
16.193
14.658
13.933
0.089
X : parts per Million : 13C

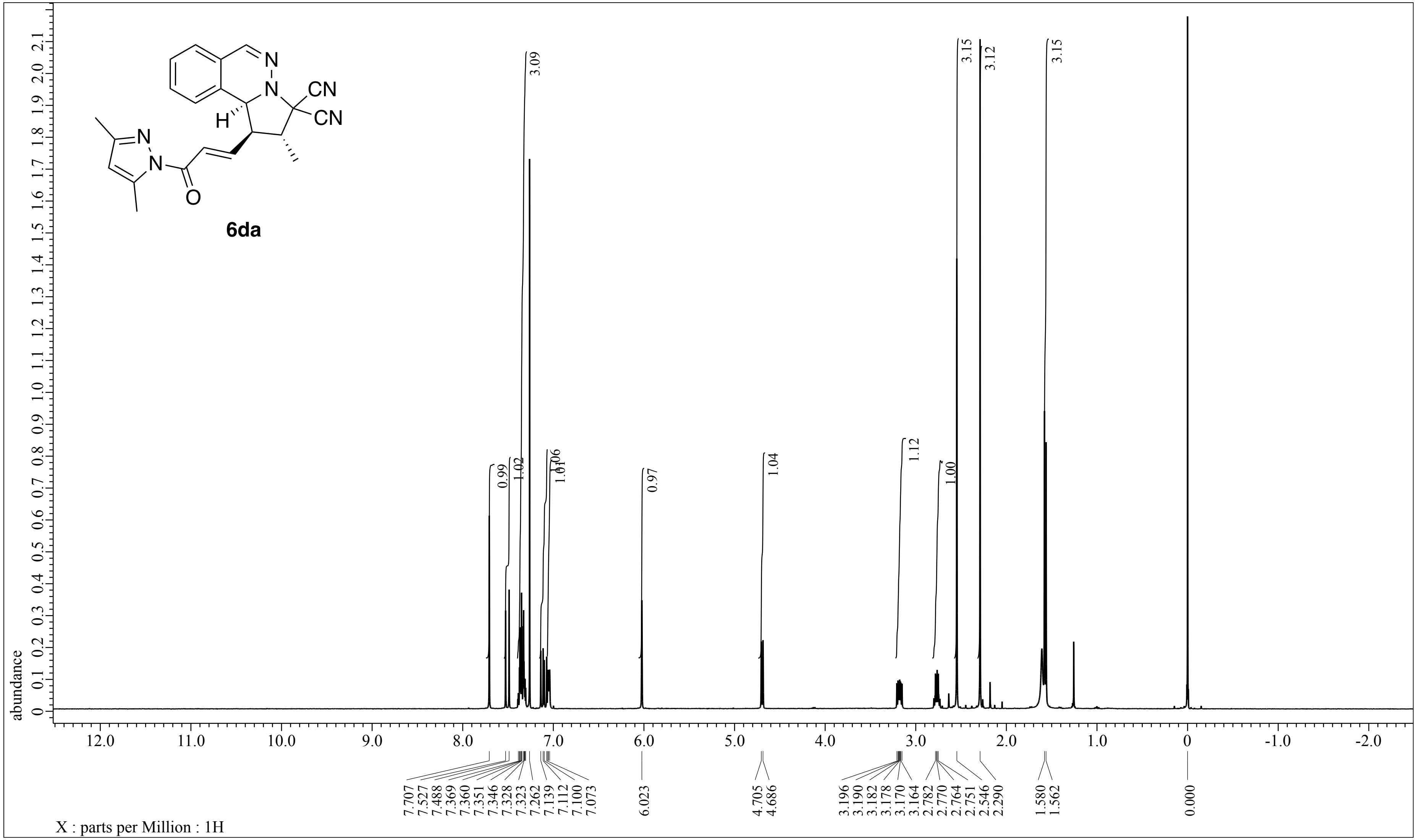
6da
abundance
X : parts per Million : 1H

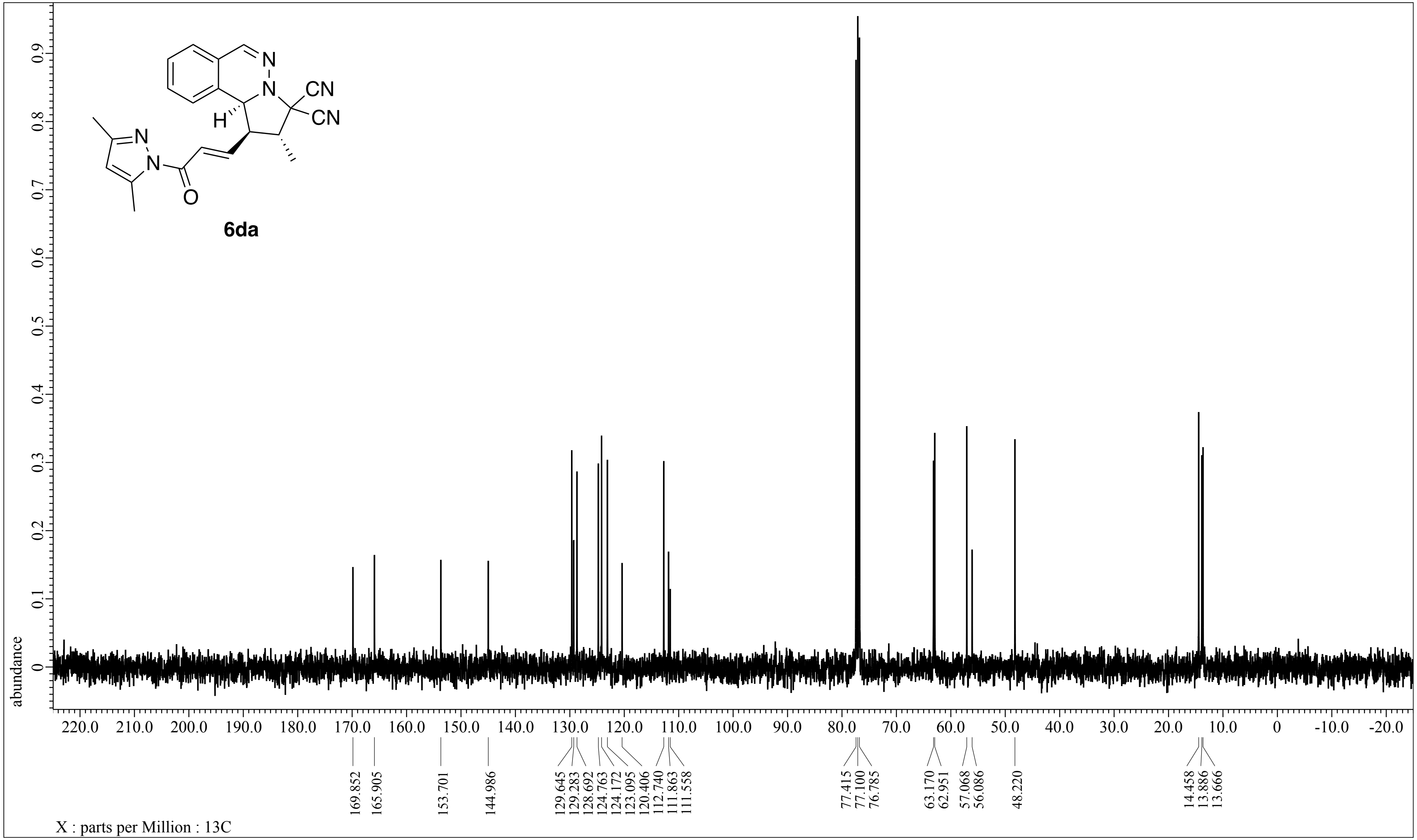

N
N
CN
CN
H
N
N
O
6da
abundance
0.9
0.8
0.7
0.6
0.5
0.4
0.3
0.2
0.1
0
220.0 210.0 200.0 190.0 180.0 170.0 160.0 150.0 140.0 130.0 120.0 110.0 100.0 90.0 80.0 70.0 60.0 50.0 40.0 30.0 20.0 10.0 0 -10.0 -20.0
169.852
165.905
153.701
144.986
129.645
129.283
128.692
124.763
124.172
123.095
120.406
112.740
111.863
111.558
77.415
77.100
76.785
63.170
62.951
57.068
56.086
48.220
14.458
13.886
13.666
X : parts per Million : 13C

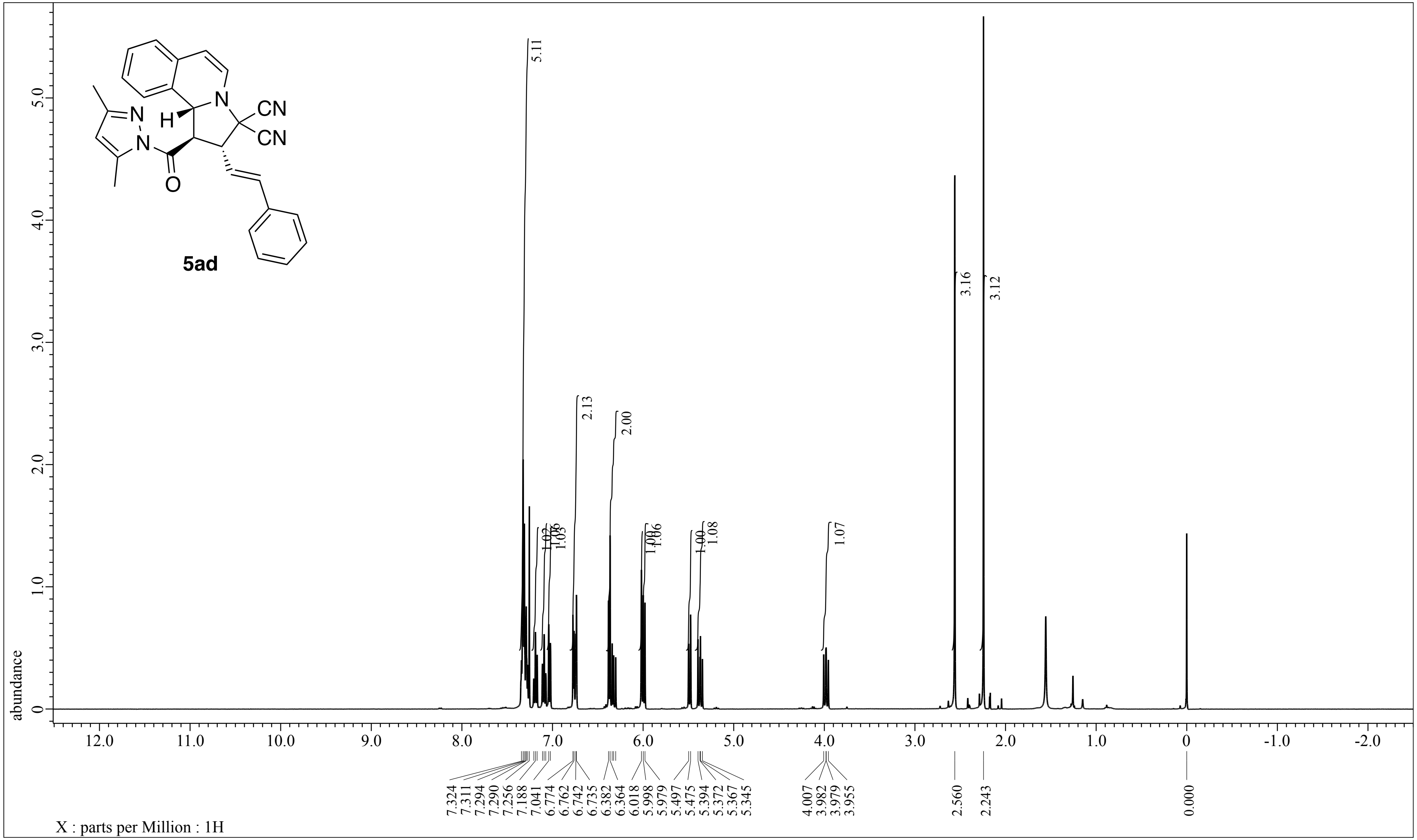

N
N
N
H
CN
CN
O
5ad
5.11
3.16
3.12
2.13
2.00
1.07
1.08
abundance
0
1.0
2.0
3.0
4.0
5.0
12.0
11.0
10.0
9.0
8.0
7.0
6.0
5.0
4.0
3.0
2.0
1.0
0
-1.0
-2.0
7.324
7.311
7.294
7.290
7.256
7.188
7.041
6.774
6.762
6.742
6.735
6.382
6.364
6.018
5.998
5.979
5.497
5.475
5.394
5.372
5.367
5.345
4.007
3.982
3.979
3.955
2.560
2.243
0.000
X : parts per Million : 1H

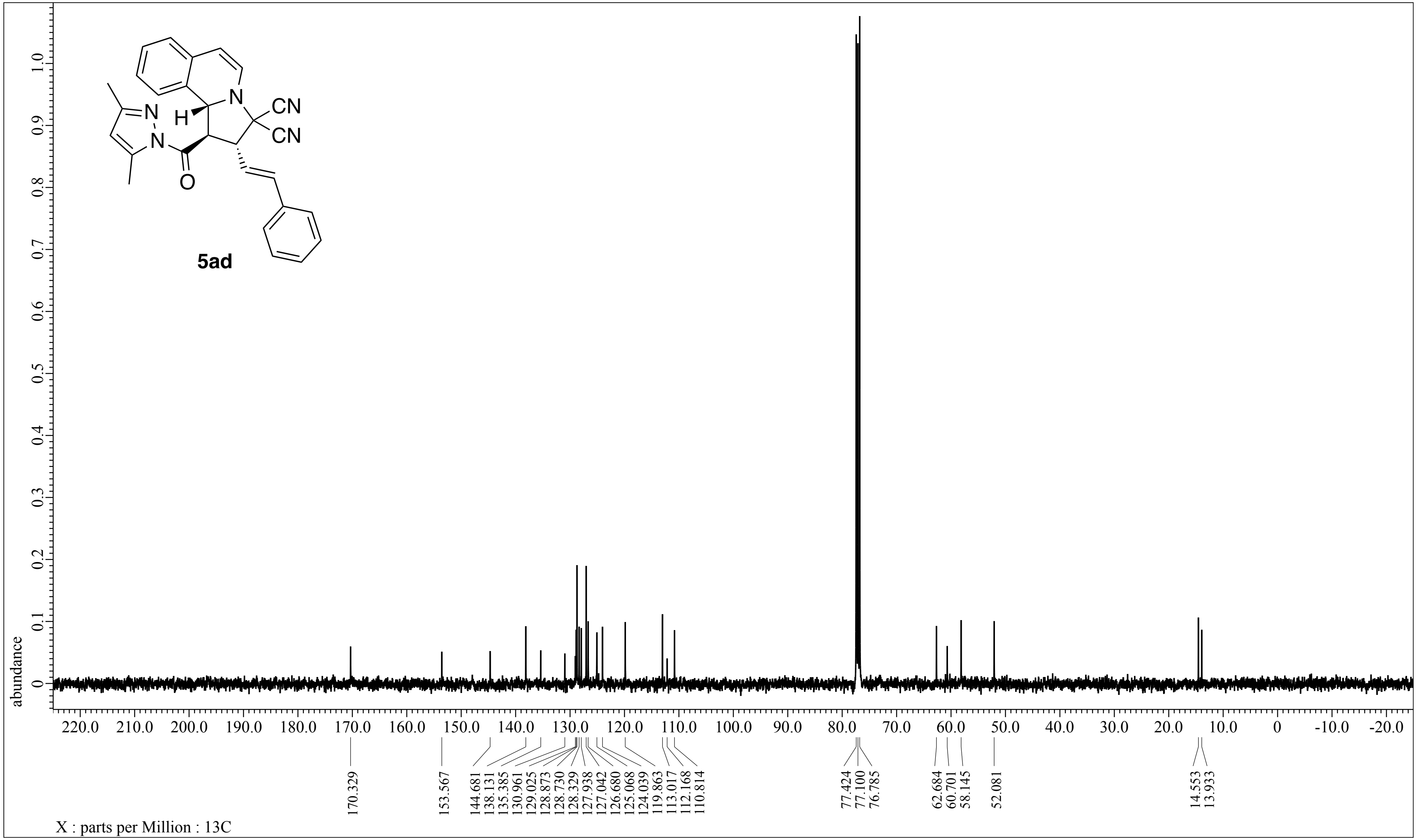

N
N
H
N
CN
CN
O
5ad
abundance
X : parts per Million : 13C
170.329
153.567
144.681
138.131
135.385
130.961
129.025
128.873
128.730
128.329
127.938
127.042
126.680
125.068
124.039
119.863
113.017
112.168
110.814
77.424
77.100
76.785
62.684
60.701
58.145
52.081
14.553
13.933

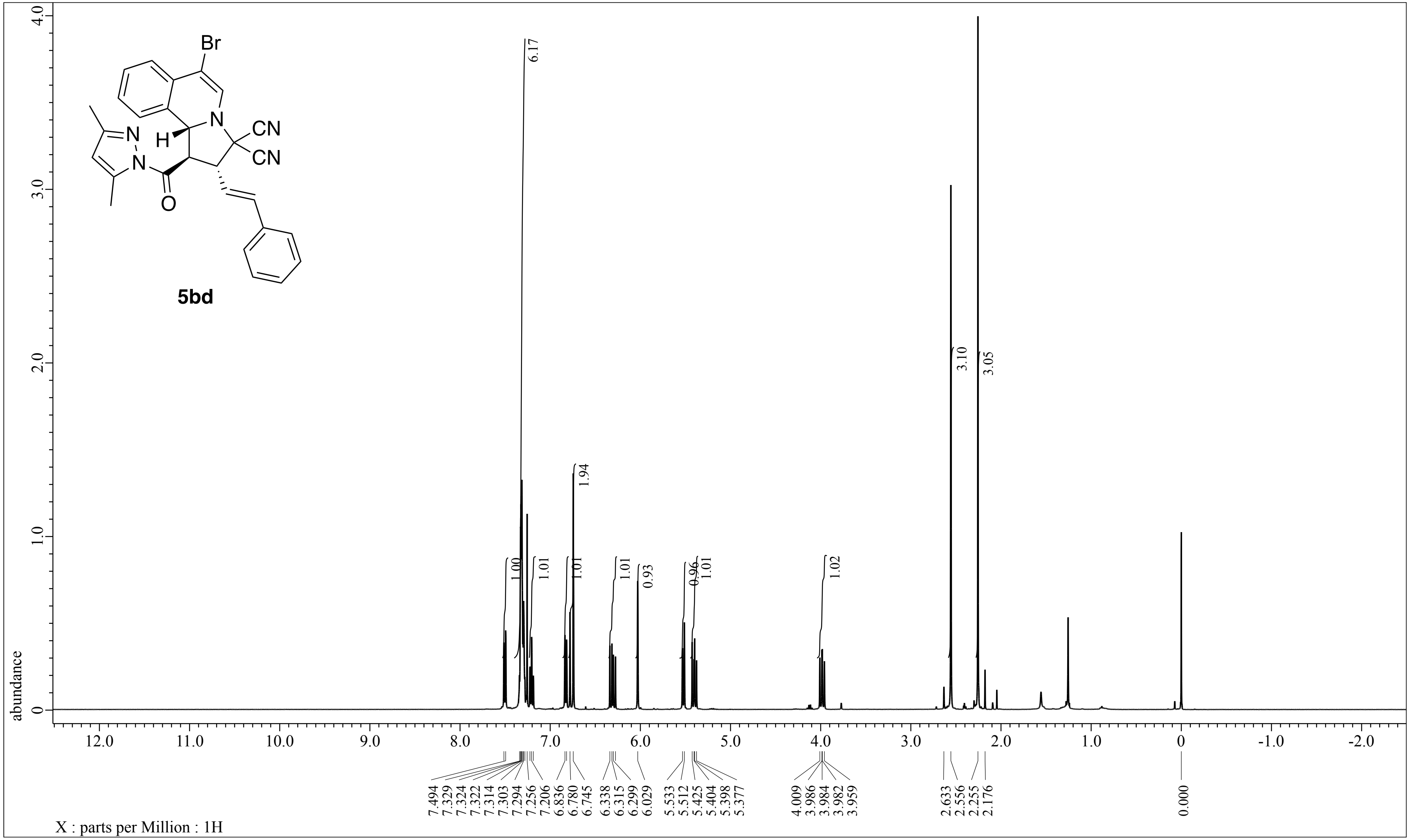

Br
N
CN
CN
H
N
N
O
5bd
abundance
X : parts per Million : 1H
6.17
1.00
1.01
1.94
1.01
1.01
0.93
0.96
1.01
1.02
3.10
3.05
7.494
7.329
7.324
7.322
7.314
7.303
7.294
7.256
7.206
6.836
6.780
6.745
6.338
6.315
6.299
6.029
5.533
5.512
5.425
5.404
5.398
5.377
4.009
3.986
3.984
3.982
3.959
2.633
2.556
2.255
2.176
0.000

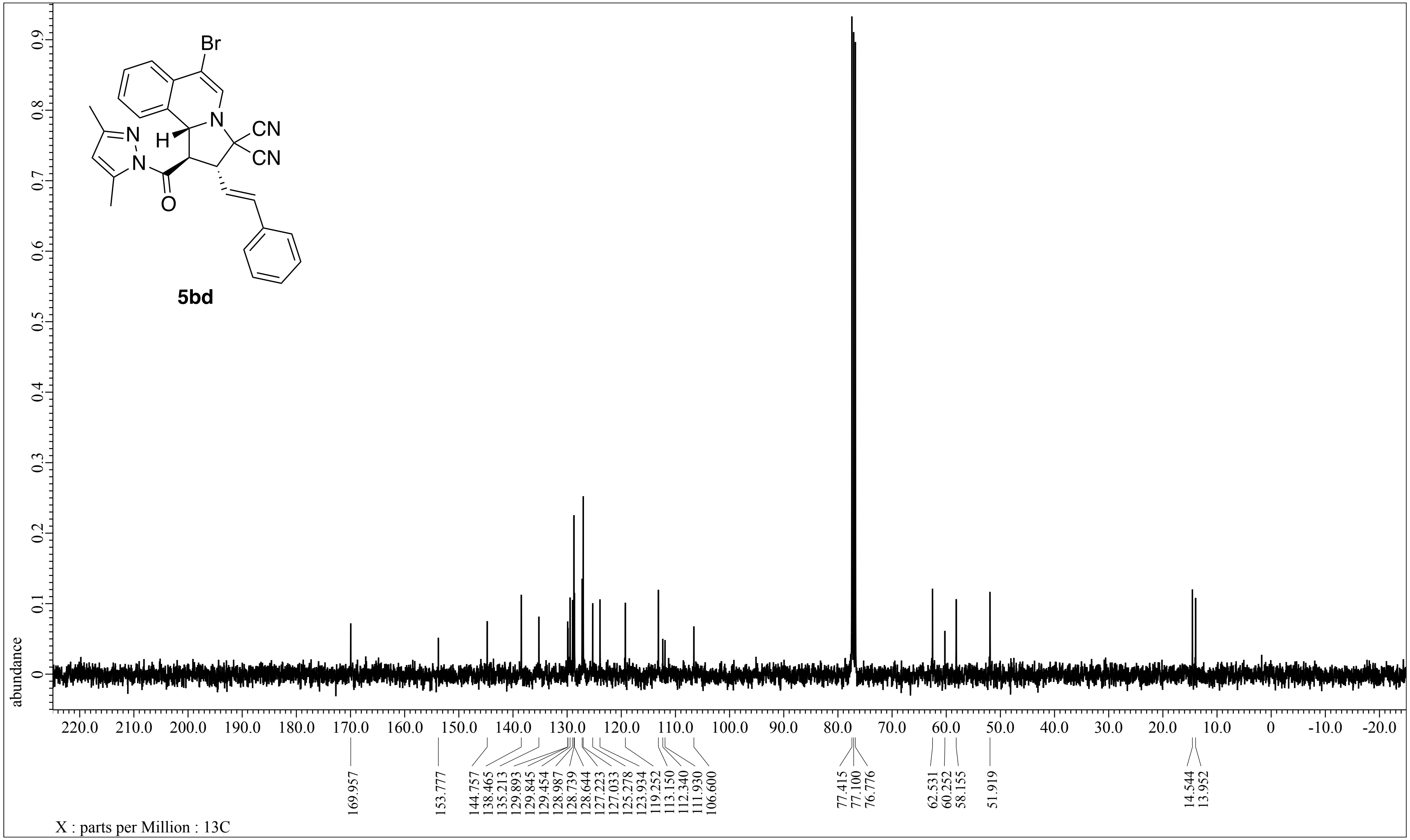

5bd
abundance
X : parts per Million : 13C
169.957
153.777
144.757
138.465
135.213
129.893
129.845
129.454
128.987
128.739
128.644
127.223
127.033
125.278
123.934
119.252
113.150
112.340
111.930
106.600
77.415
77.100
76.776
62.531
60.252
58.155
51.919
14.544
13.952

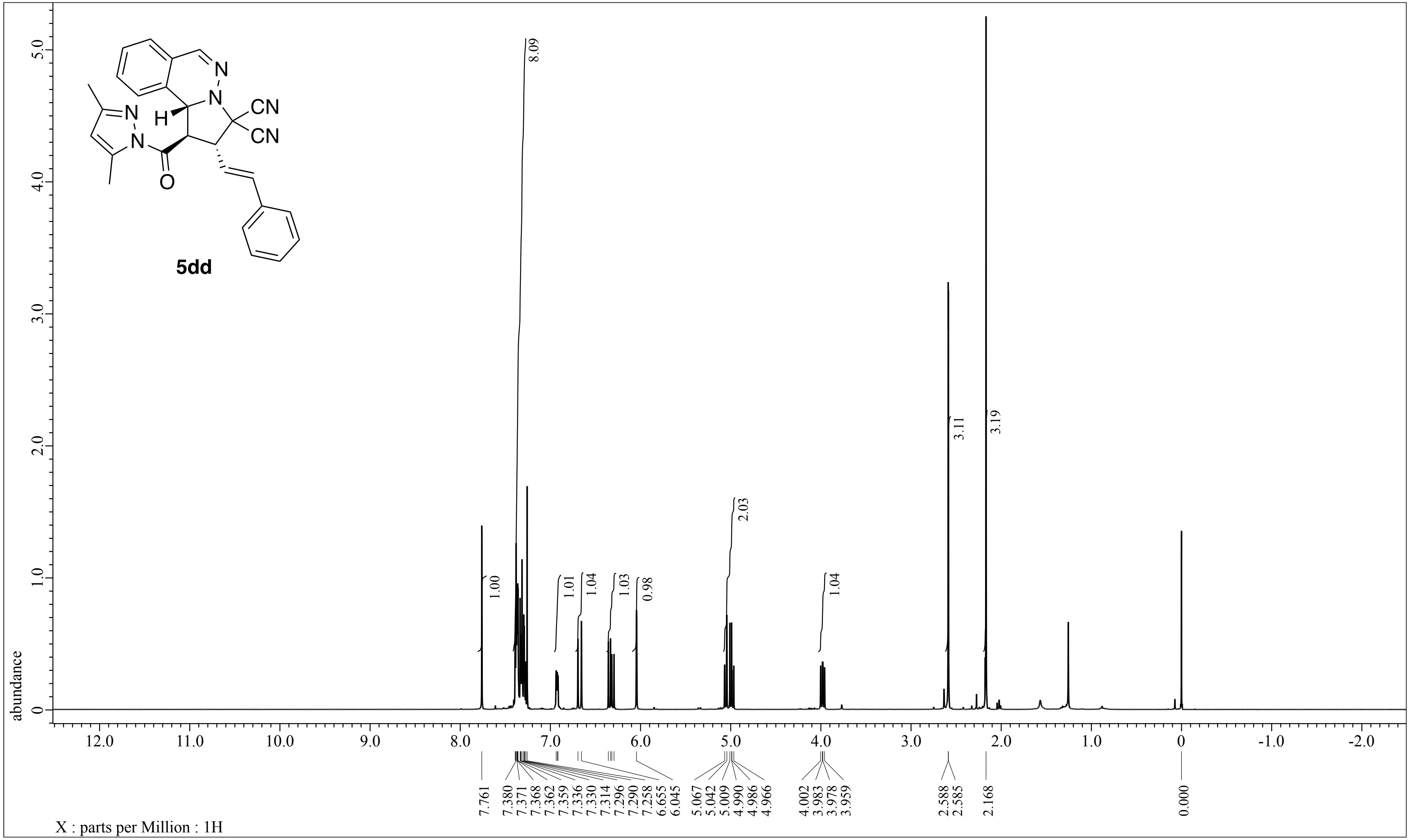
5dd
CN
CN
H
N
N
O
abundance
X : parts per Million : 1H
8.09
1.00
1.01
1.04
1.03
0.98
2.03
1.04
3.11
3.19
7.761
7.380
7.371
7.368
7.362
7.359
7.336
7.330
7.314
7.296
7.290
7.258
6.655
6.045
5.067
5.042
5.009
4.990
4.986
4.966
4.002
3.983
3.978
3.959
2.588
2.585
2.168
0.000

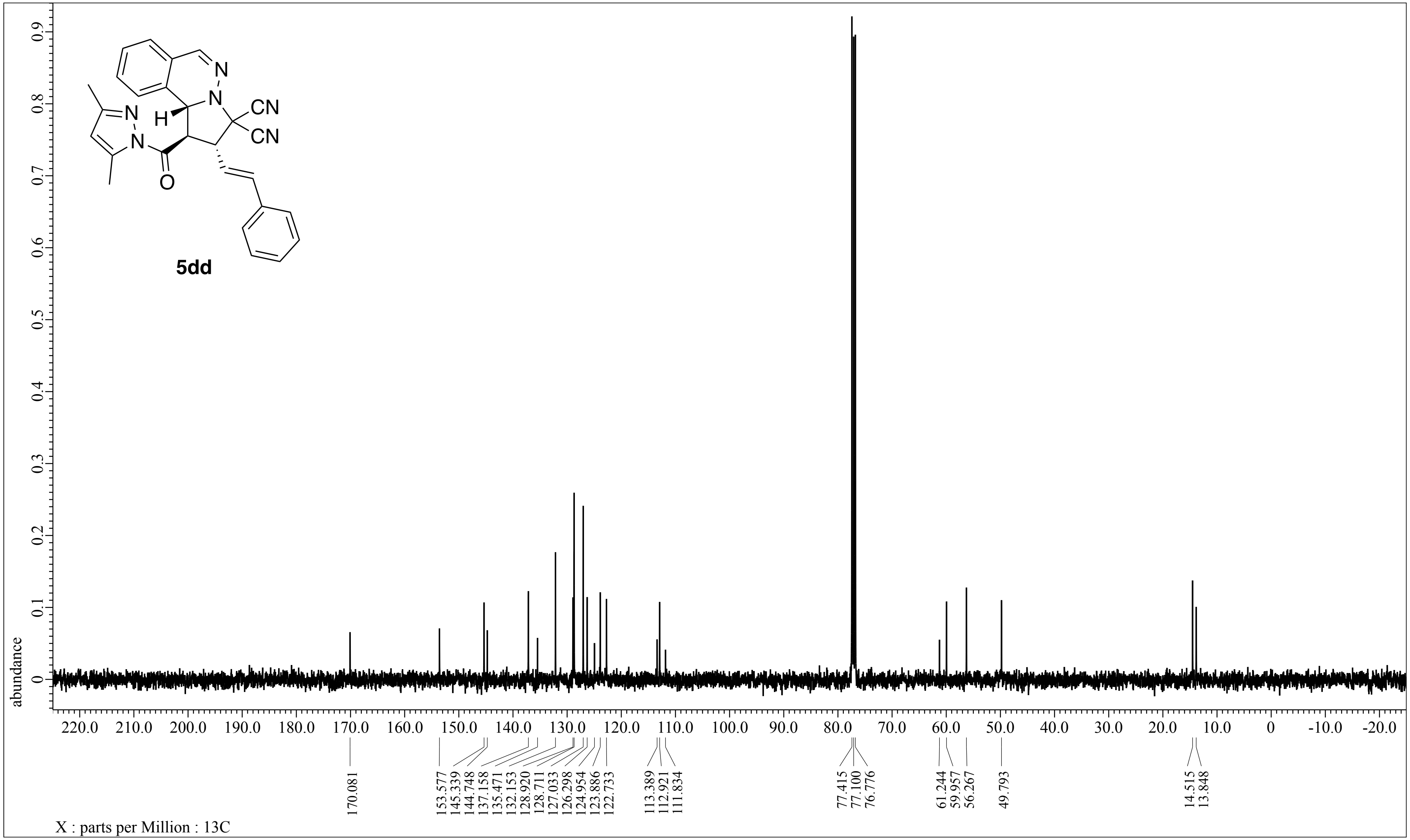
5dd
abundance
X : parts per Million : 13C
170.081
153.577
145.339
144.748
137.158
135.471
132.153
128.920
128.711
127.033
126.298
124.954
123.886
122.733
113.389
112.921
111.834
77.415
77.100
76.776
61.244
59.957
56.267
49.793
14.515
13.848

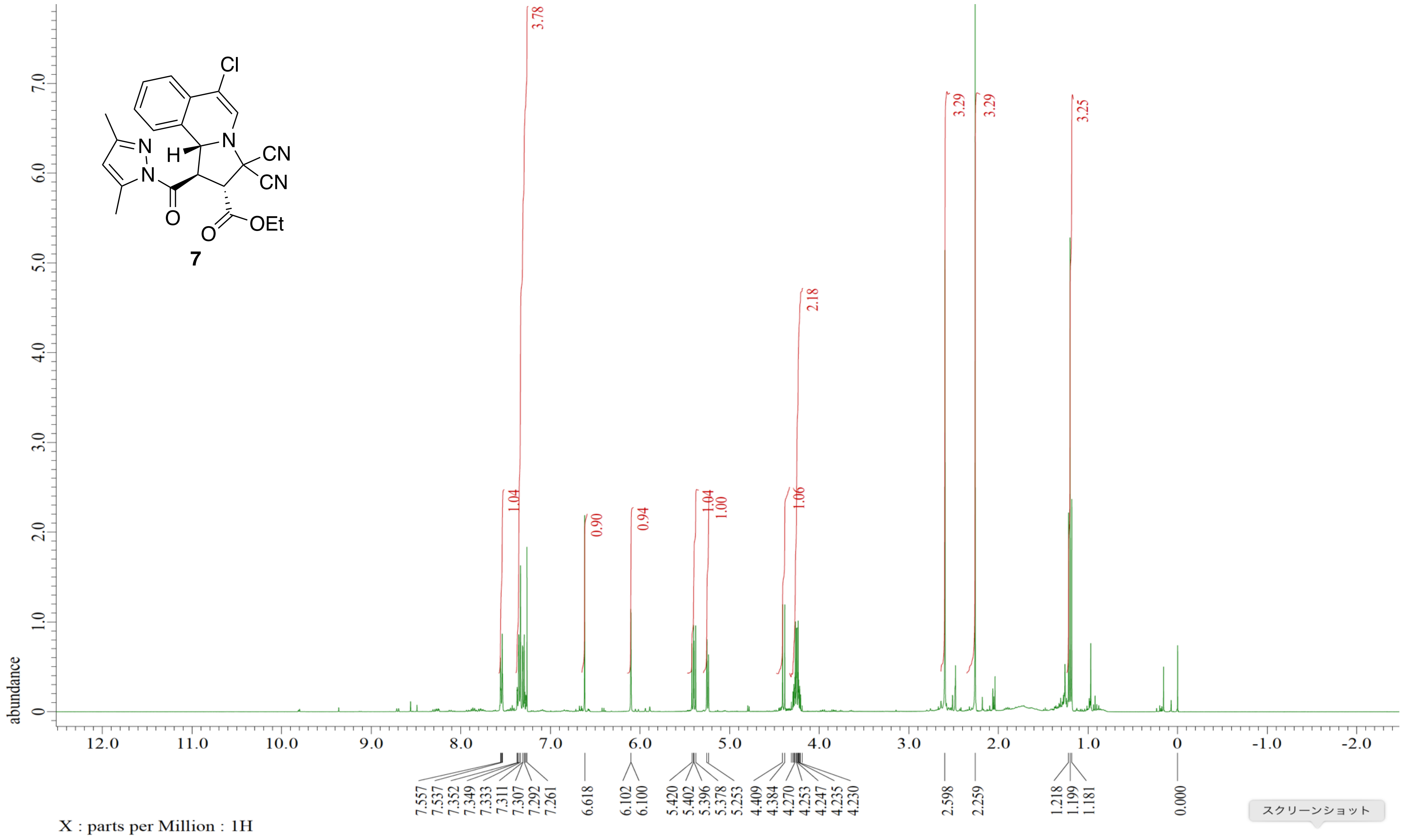
Cl
N
N
H
N
CN
CN
O
O
OEt
7
abundance
X : parts per Million : 1H
3.78
3.29
3.29
3.25
2.18
1.04
0.90
0.94
1.04
1.00
1.06
7.557
7.537
7.352
7.349
7.333
7.311
7.307
7.292
7.261
6.618
6.102
6.100
5.420
5.402
5.396
5.378
5.253
4.409
4.384
4.270
4.253
4.247
4.235
4.230
2.598
2.259
1.218
1.199
1.181
0.000
スクリーンショット

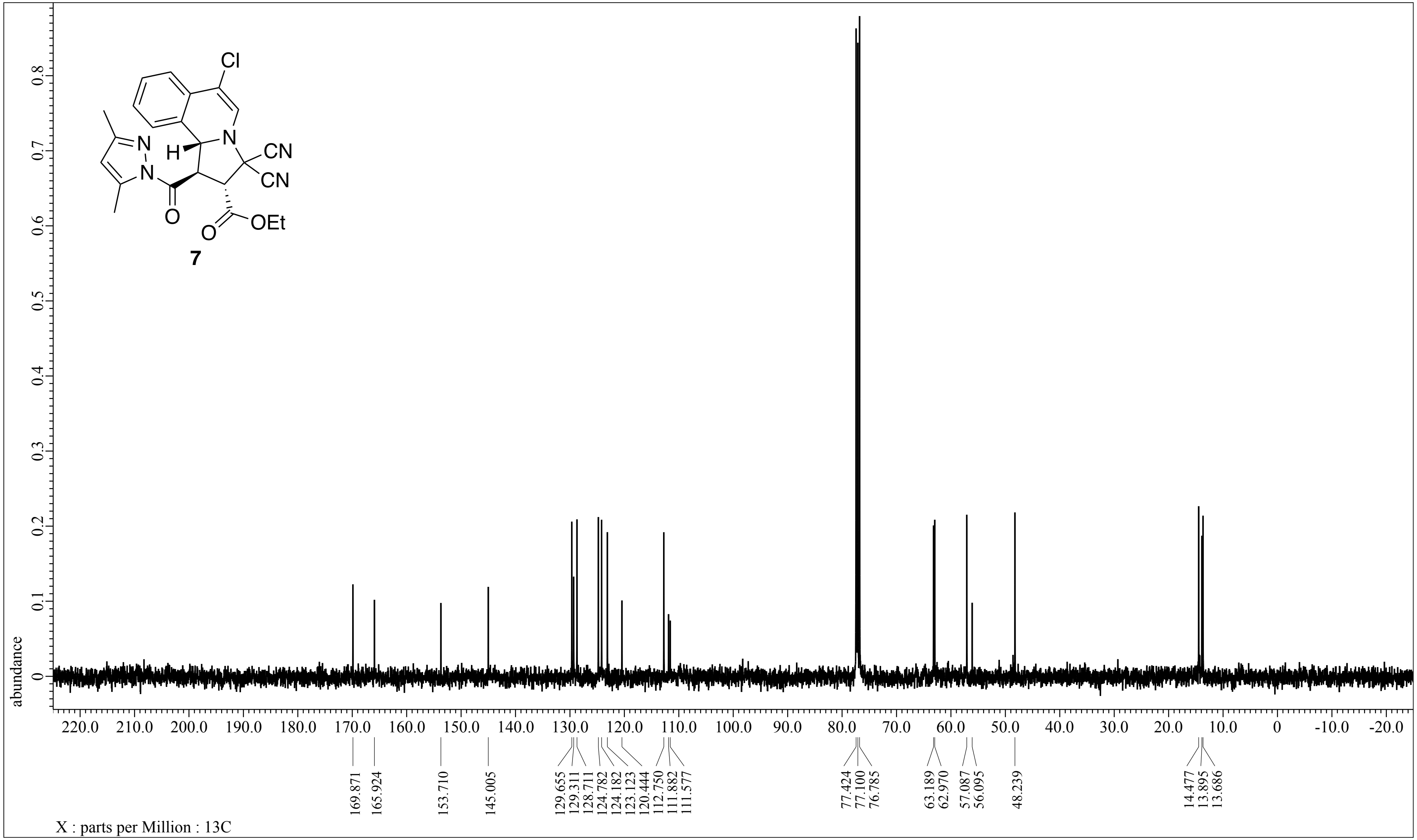

Cl
N
H
CN
CN
N
N
O
O
OEt
7
abundance
0.8
0.7
0.6
0.5
0.4
0.3
0.2
0.1
0
220.0 210.0 200.0 190.0 180.0 170.0 160.0 150.0 140.0 130.0 120.0 110.0 100.0 90.0 80.0 70.0 60.0 50.0 40.0 30.0 20.0 10.0 0 -10.0 -20.0
169.871
165.924
153.710
145.005
129.655
129.311
128.711
124.782
124.182
123.123
120.444
112.750
111.882
111.577
77.424
77.100
76.785
63.189
62.970
57.087
56.095
48.239
14.477
13.895
13.686
X : parts per Million : 13C

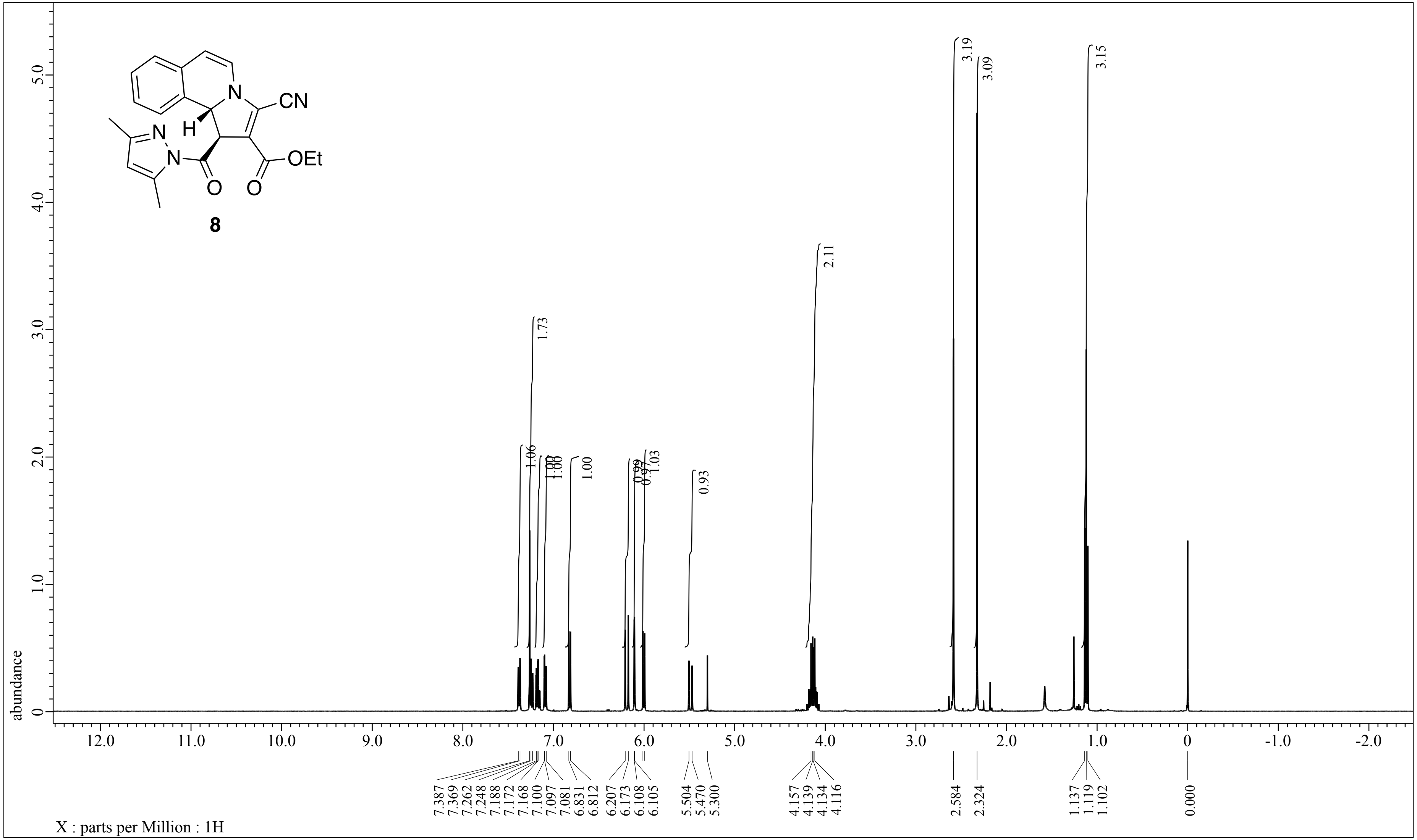

CN
N
H
N
N
OEt
O
O
8
abundance
X : parts per Million : 1H
7.387
7.369
7.262
7.248
7.188
7.172
7.168
7.100
7.097
7.081
6.831
6.812
6.207
6.173
6.108
6.105
5.504
5.470
5.300
4.157
4.139
4.134
4.116
2.584
2.324
1.137
1.119
1.102
0.000
1.06
1.73
1.00
1.00
0.99
0.97
1.03
0.93
2.11
3.19
3.09
3.15

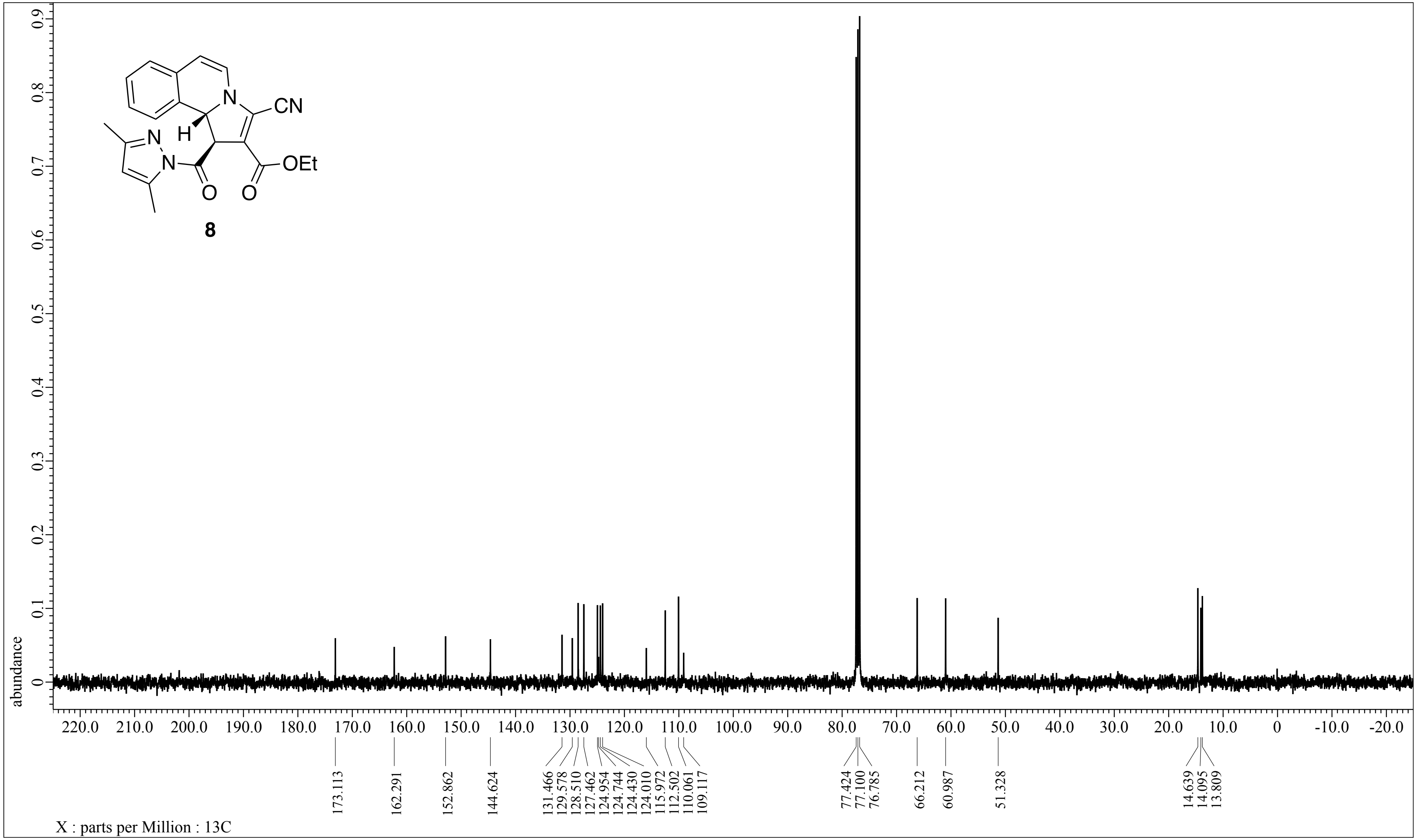

CN
N
N
N
H
OEt
O
O
8
abundance
0.9
0.8
0.7
0.6
0.5
0.4
0.3
0.2
0.1
0
220.0 210.0 200.0 190.0 180.0 170.0 160.0 150.0 140.0 130.0 120.0 110.0 100.0 90.0 80.0 70.0 60.0 50.0 40.0 30.0 20.0 10.0 0 -10.0 -20.0
173.113
162.291
152.862
144.624
131.466
129.578
128.510
127.462
124.954
124.744
124.430
124.010
115.972
112.502
110.061
109.117
77.424
77.100
76.785
66.212
60.987
51.328
14.639
14.095
13.809
X : parts per Million : 13C

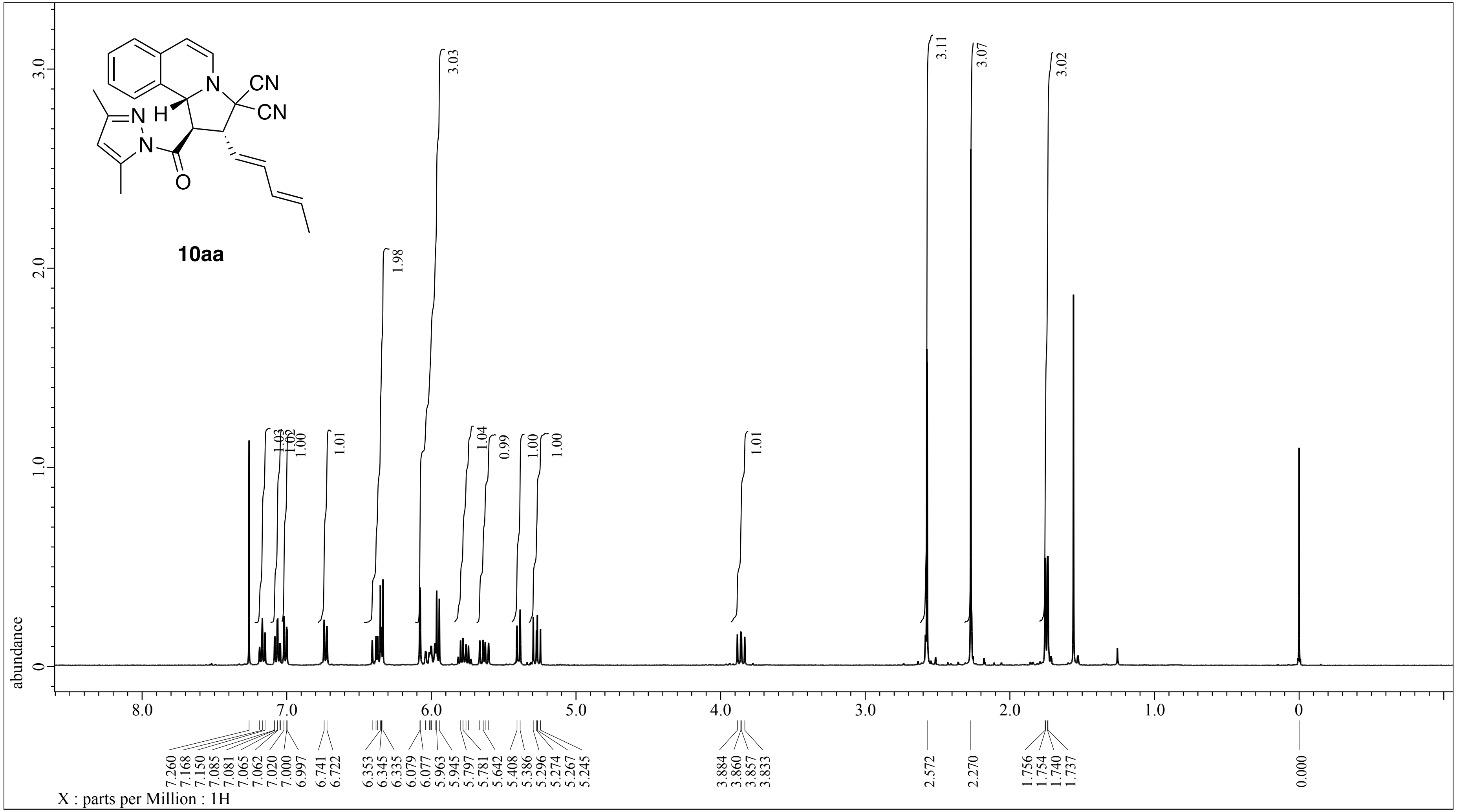
10aa
X : parts per Million : 1H
abundance

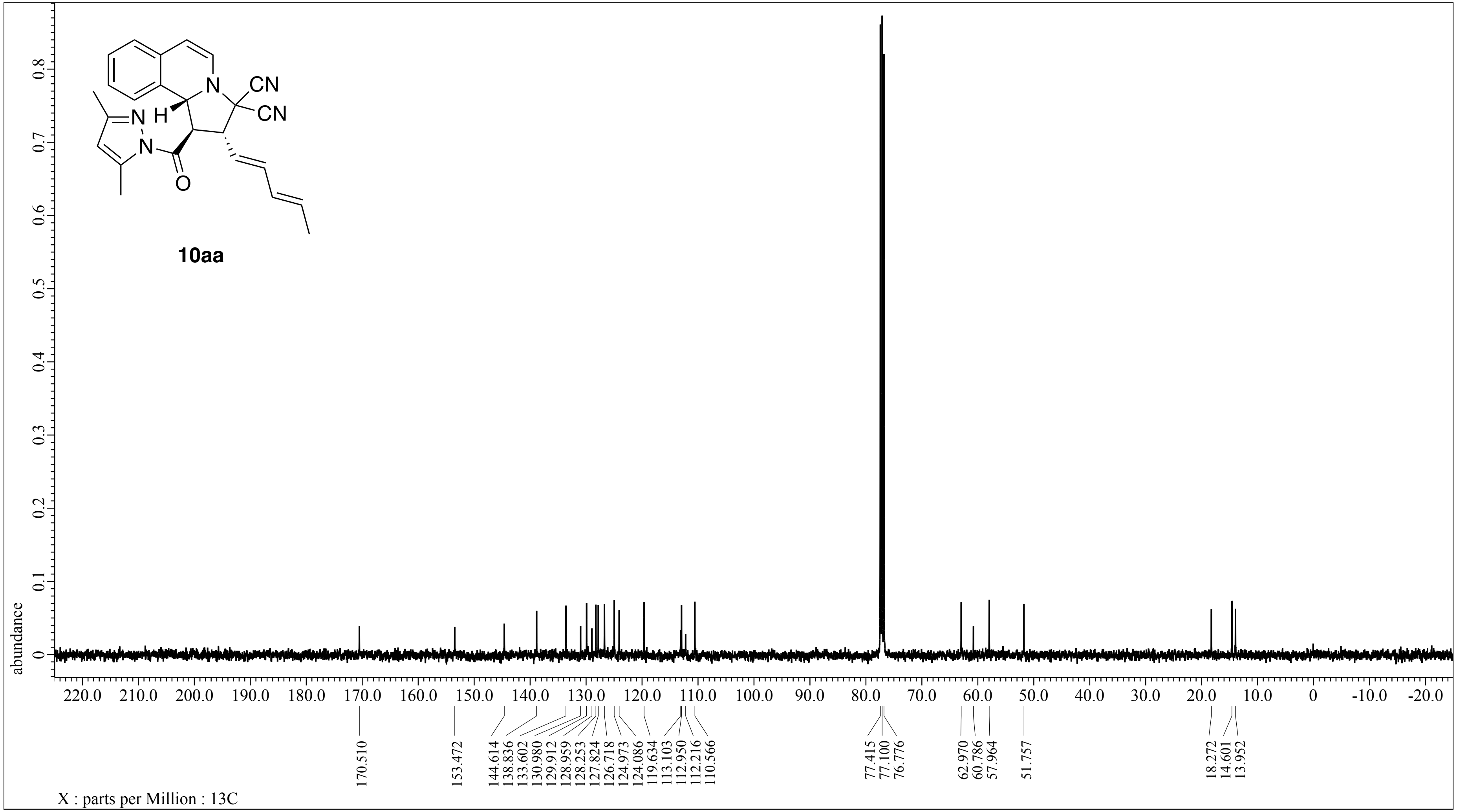
10aa
CN
CN
N
N
N
H
O
abundance
X : parts per Million : 13C
170.510
153.472
144.614
138.836
133.602
130.980
129.912
128.959
128.253
127.824
126.718
124.973
124.086
119.634
113.103
112.950
112.216
110.566
77.415
77.100
76.776
62.970
60.786
57.964
51.757
18.272
14.601
13.952

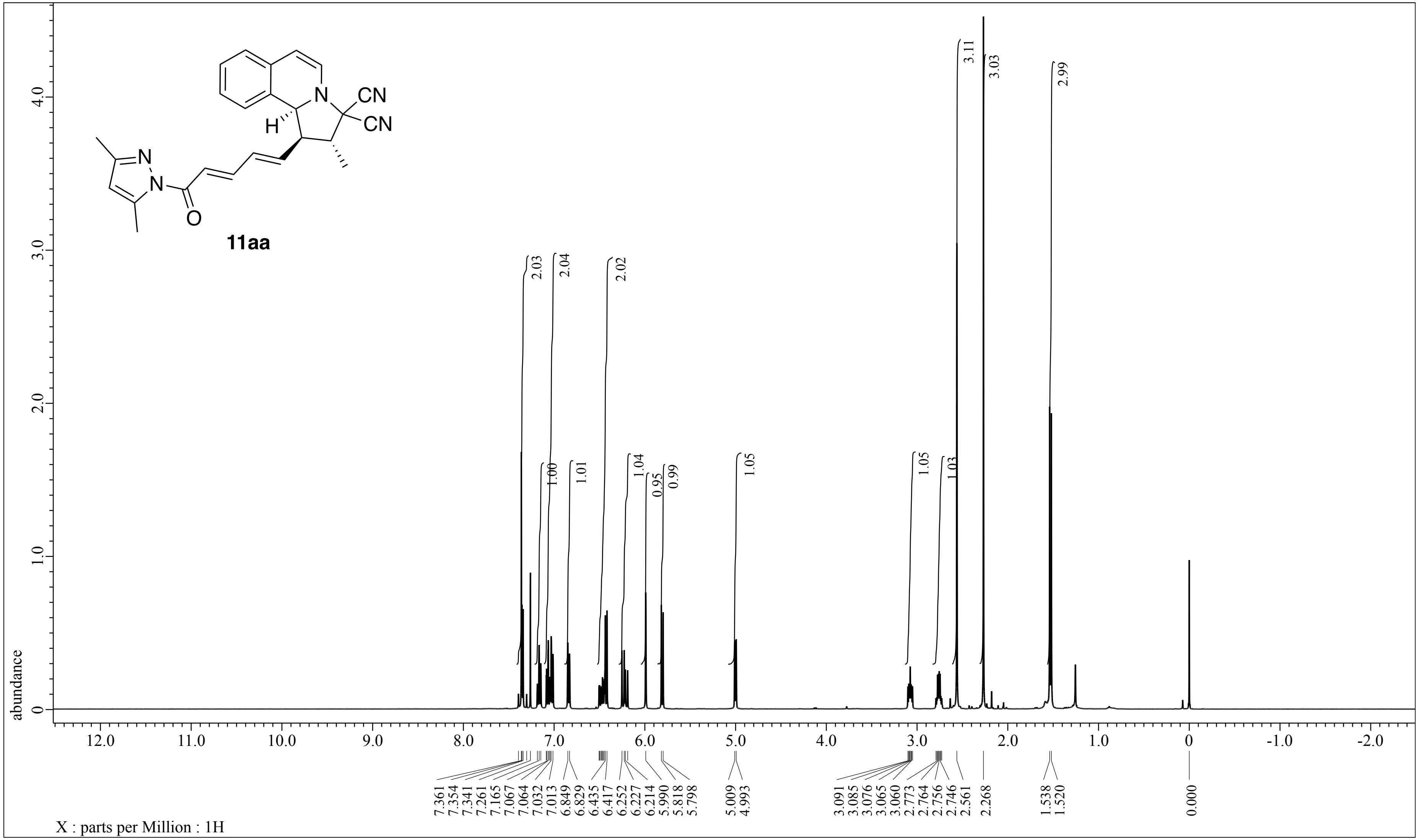
11aa
CN
CN
N
H
N
N
O
abundance
X : parts per Million : 1H
2.03
2.04
2.02
1.00
1.01
1.04
0.95
0.99
1.05
1.05
1.03
3.11
3.03
2.99
7.361
7.354
7.341
7.261
7.165
7.067
7.064
7.032
7.013
6.849
6.829
6.435
6.417
6.252
6.227
6.214
5.990
5.818
5.798
5.009
4.993
3.091
3.085
3.076
3.065
3.060
2.773
2.764
2.756
2.746
2.561
2.268
1.538
1.520
0.000

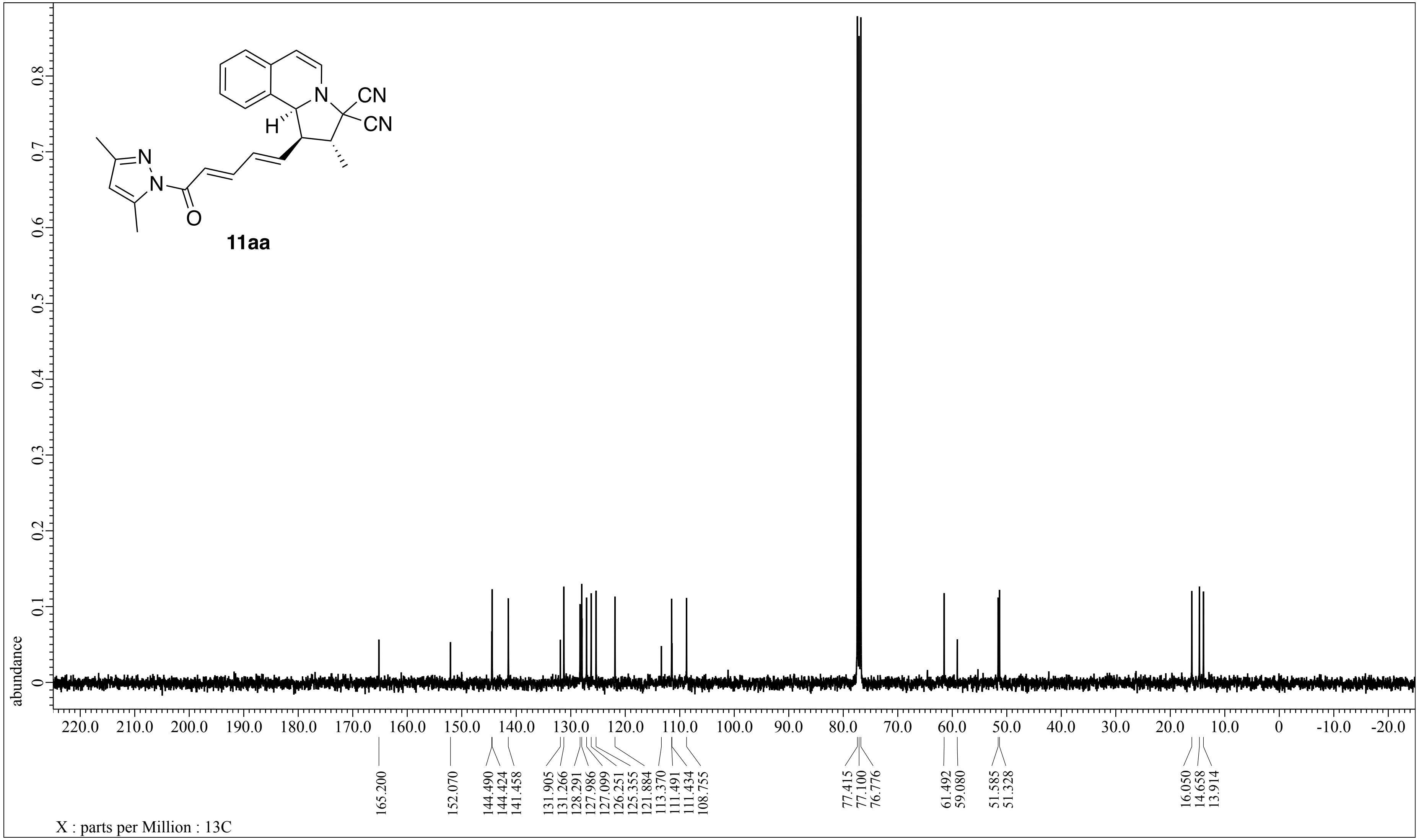
11aa
abundance
X : parts per Million : 13C
165.200
152.070
144.490
144.424
141.458
131.905
131.266
128.291
127.986
127.099
126.251
125.355
121.884
113.370
111.491
111.434
108.755
77.415
77.100
76.776
61.492
59.080
51.585
51.328
16.050
14.658
13.914

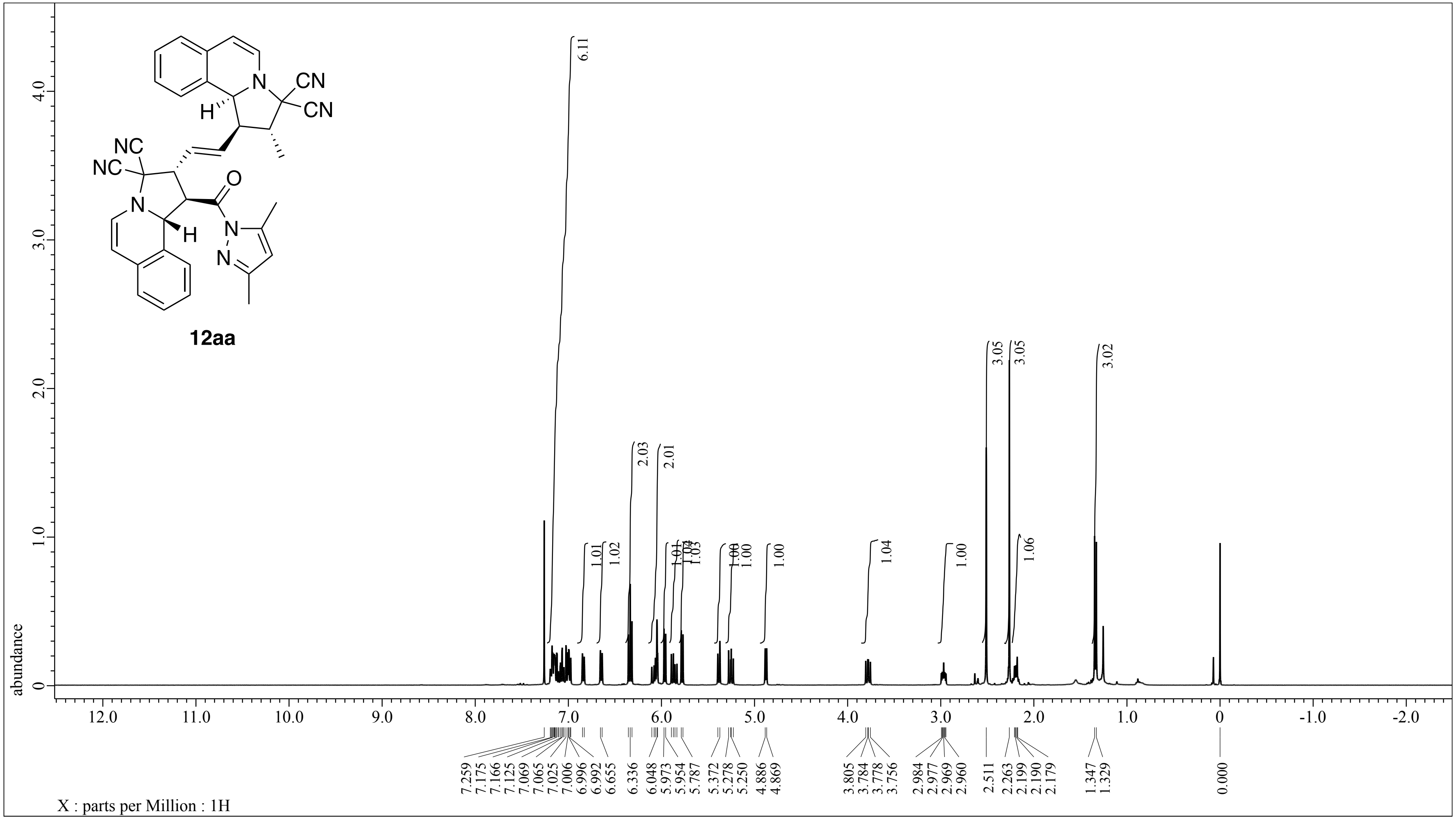
12aa
abundance
X : parts per Million : 1H
12.0 11.0 10.0 9.0 8.0 7.0 6.0 5.0 4.0 3.0 2.0 1.0 0 -1.0 -2.0
0 1.0 2.0 3.0 4.0
6.11
1.01
1.02
2.03
2.01
1.01
1.04
1.03
1.00
1.00
1.00
1.04
1.00
3.05
3.05
1.06
3.02
7.259 7.175 7.166 7.125 7.069 7.065 7.025 7.006 6.996 6.992 6.655
6.336
6.048 5.973 5.954 5.787
5.372 5.278 5.250 4.886 4.869
3.805 3.784 3.778 3.756
2.984 2.977 2.969 2.960
2.511
2.263 2.199 2.190 2.179
1.347 1.329
0.000

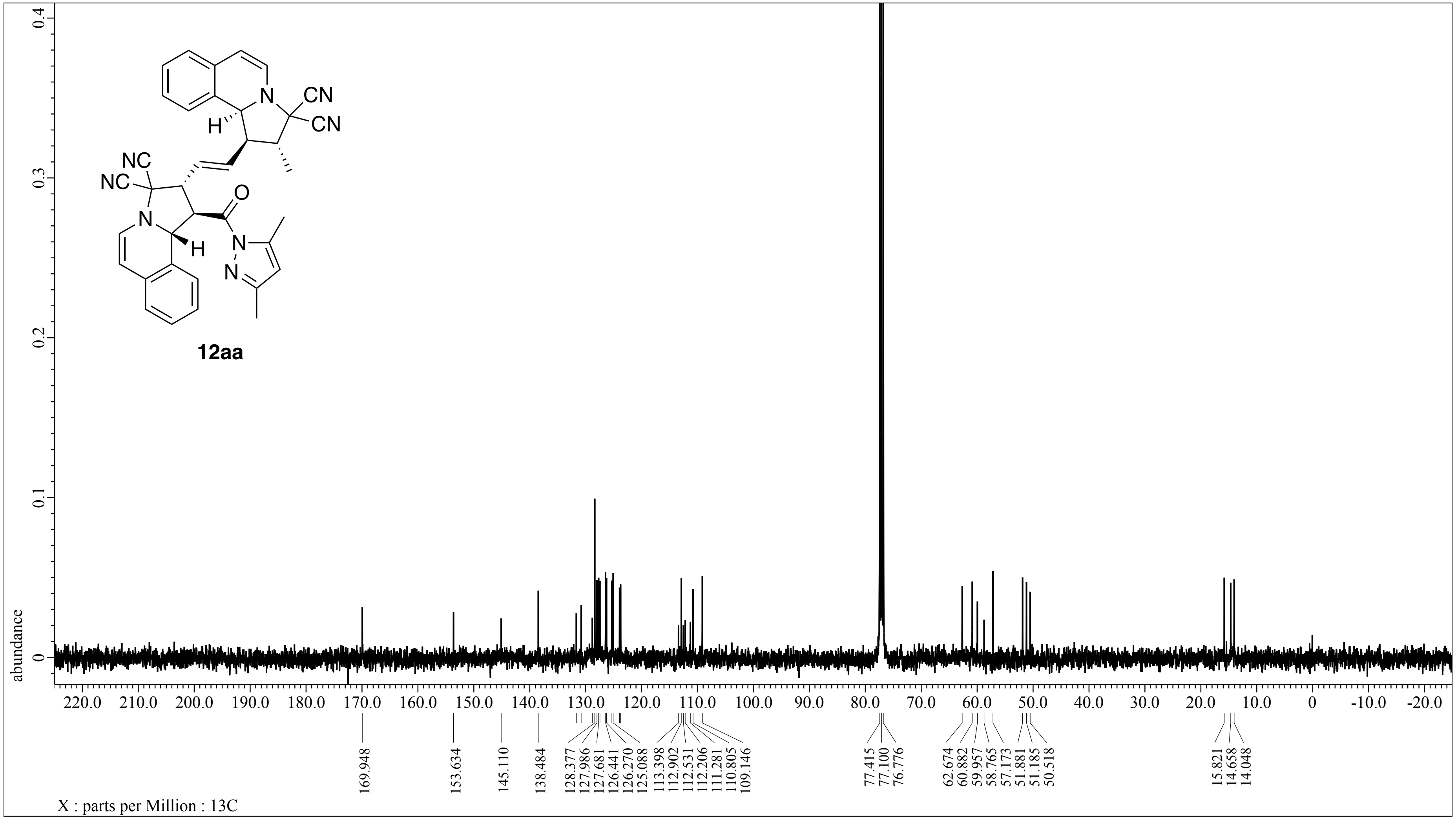

CN
CN
H
NC
NC
N
N
O
N
N
H
12aa
abundance
0.4
0.3
0.2
0.1
0
220.0 210.0 200.0 190.0 180.0 170.0 160.0 150.0 140.0 130.0 120.0 110.0 100.0 90.0 80.0 70.0 60.0 50.0 40.0 30.0 20.0 10.0 0 -10.0 -20.0
169.948
153.634
145.110
138.484
128.377
127.986
127.681
126.441
126.270
125.088
113.398
112.902
112.531
112.206
111.281
110.805
109.146
77.415
77.100
76.776
62.674
60.882
59.957
58.765
57.173
51.881
51.185
50.518
15.821
14.658
14.048
X : parts per Million : 13C

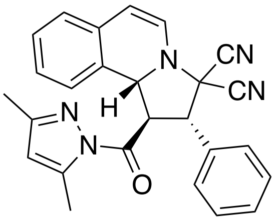

3aa

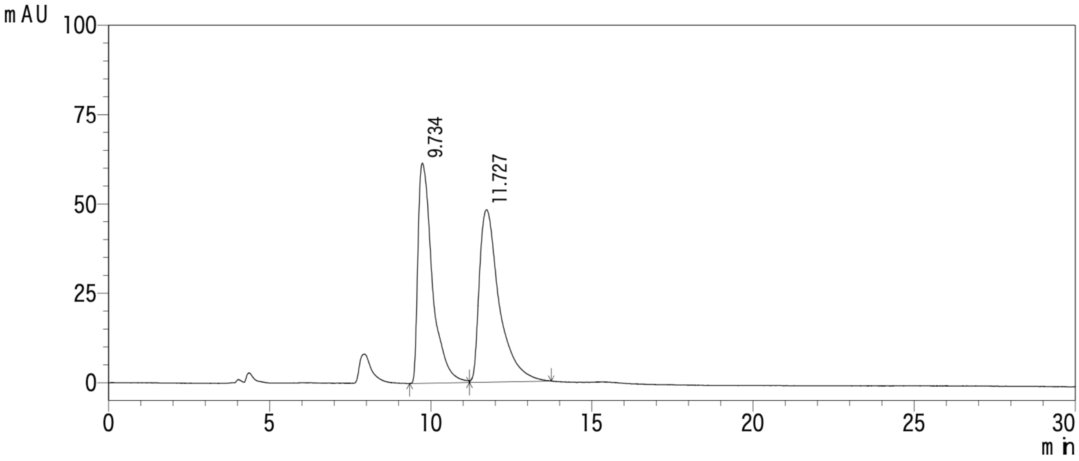

PDA Ch1 210nm

Peak No.	RT (min)	Area	Height	% Area
1	9.734	1914563	61589	48.066
2	11.727	2068602	48236	51.934
Total		3983165	109825	100.000

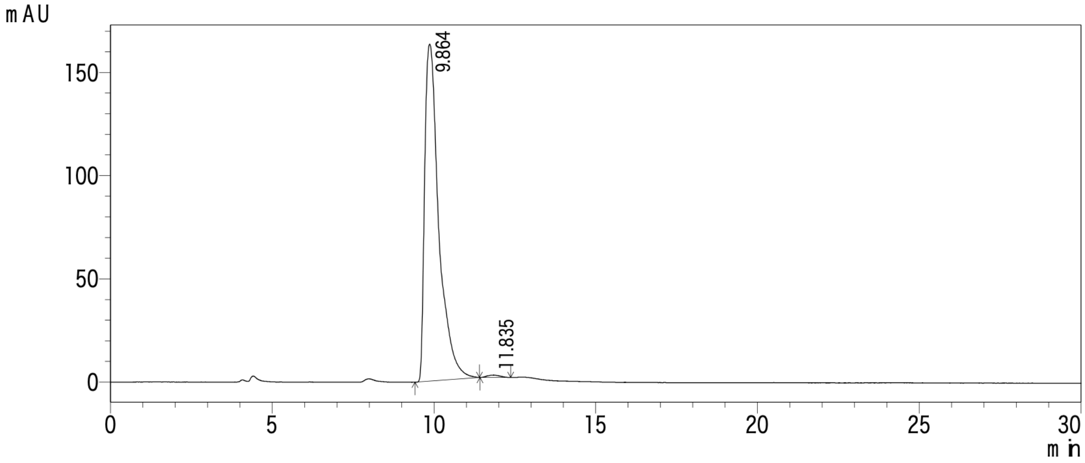

PDA Ch1 210nm

Peak No.	RT (min)	Area	Height	% Area
1	9.864	4941380	163371	99.369
2	11.835	31381	1154	0.631
Total		4972761	164526	100.000

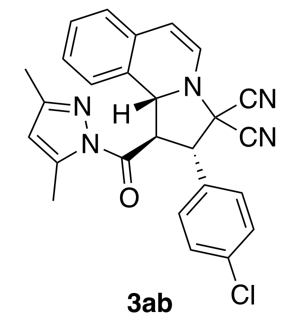

3ab

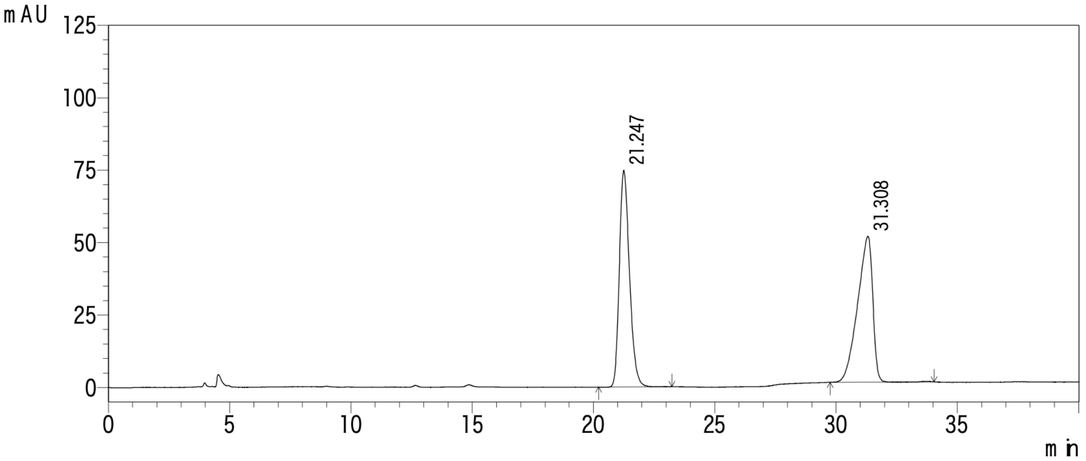

PDA Ch1 210nm

Peak No.	RT (min)	Area	Height	% Area
1	21.247	2164505	74719	49.831
2	31.308	2179162	50276	50.169
Total		4343668	124995	100.000

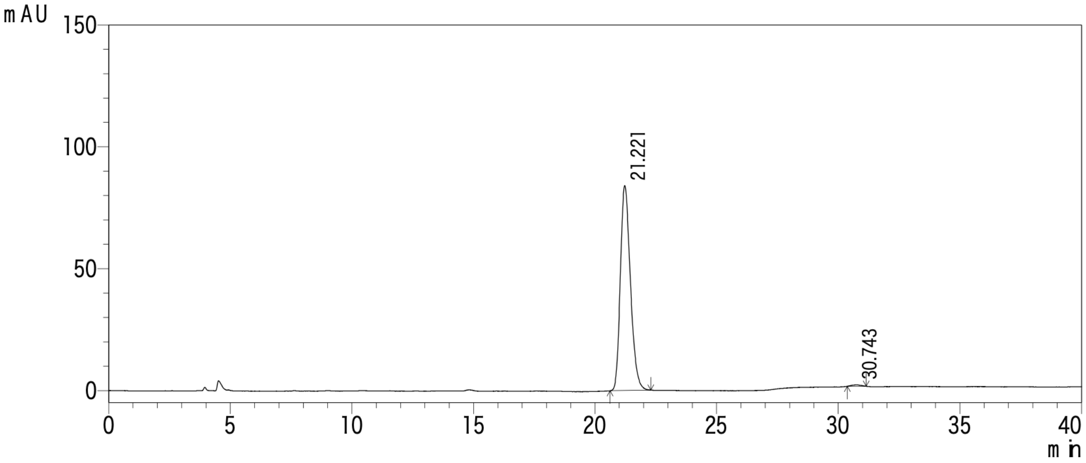

PDA Ch1 210nm

Peak No.	RT (min)	Area	Height	% Area
1	21.221	2418761	84095	99.373
2	30.743	15249	590	0.627
Total		2434011	84685	100.000

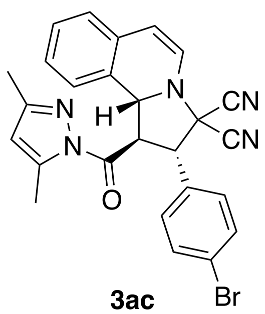

3ac

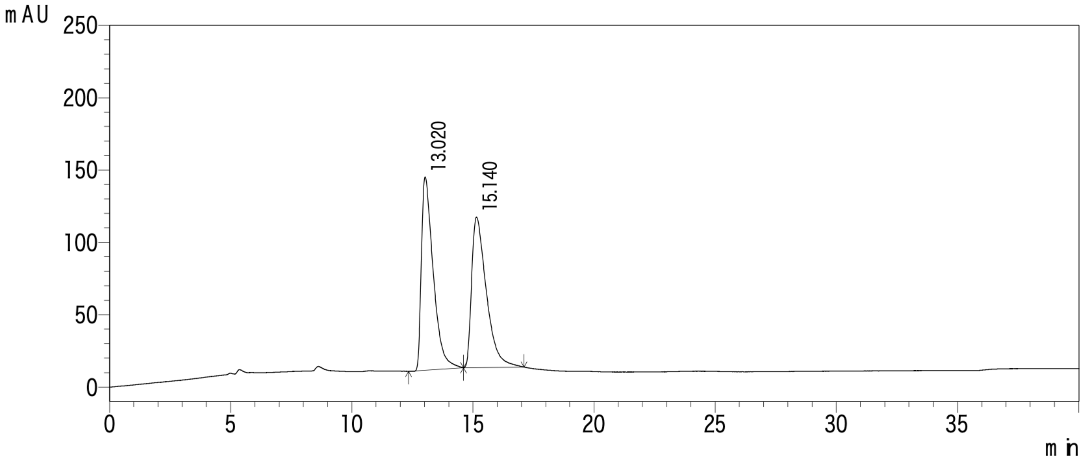

PDA Ch1 210nm

Peak No.	RT (min)	Area	Height	% Area
1	13.020	4525516	133518	50.740
2	15.140	4393486	103935	49.260
Total		8919002	237453	100.000

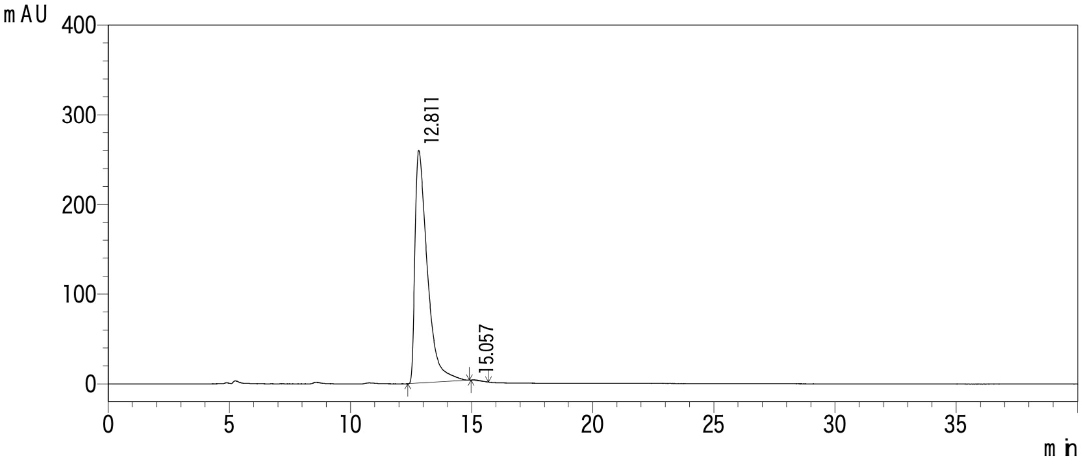

PDA Ch1 210nm

Peak No.	RT (min)	Area	Height	% Area
1	12.811	9217153	259619	99.862
2	15.057	12736	426	0.138
Total		9229889	260045	100.000

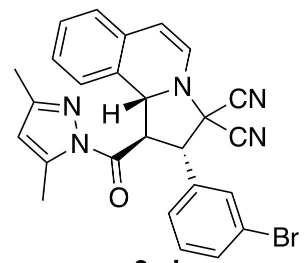

3ad

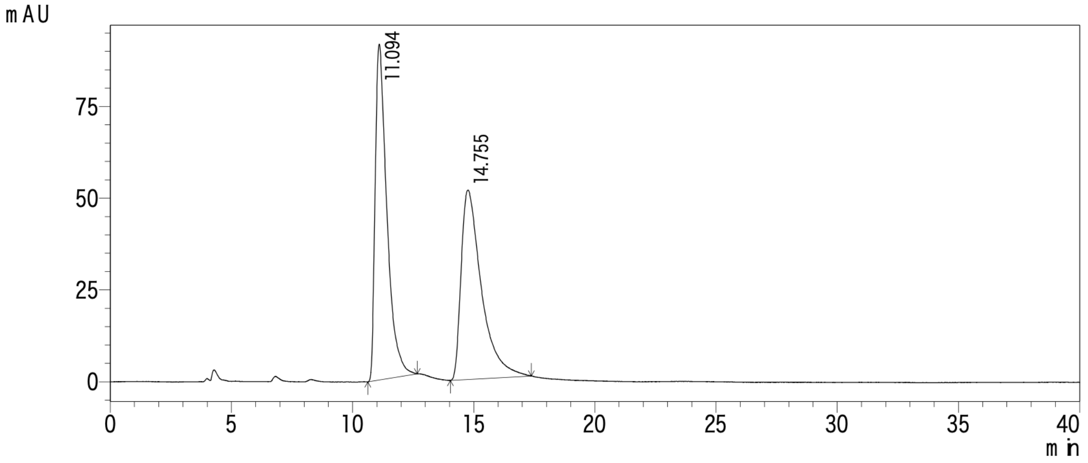

PDA Ch1 210nm

Peak No.	RT (min)	Area	Height	% Area
1	11.094	3080545	91545	50.598
2	14.755	3007754	51574	49.402
Total		6088298	143119	100.000

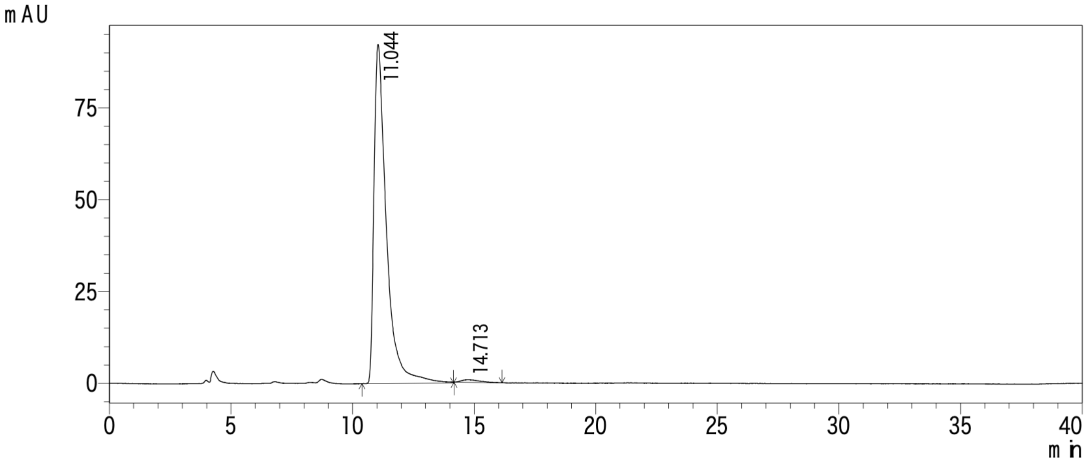

PDA Ch1 210nm

Peak No.	RT (min)	Area	Height	% Area
1	11.044	3276476	92378	98.924
2	14.713	35626	729	1.076
Total		3312101	93107	100.000

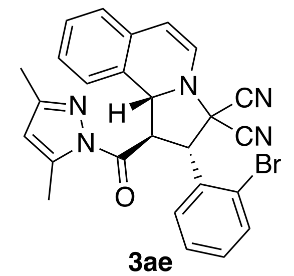

3ae

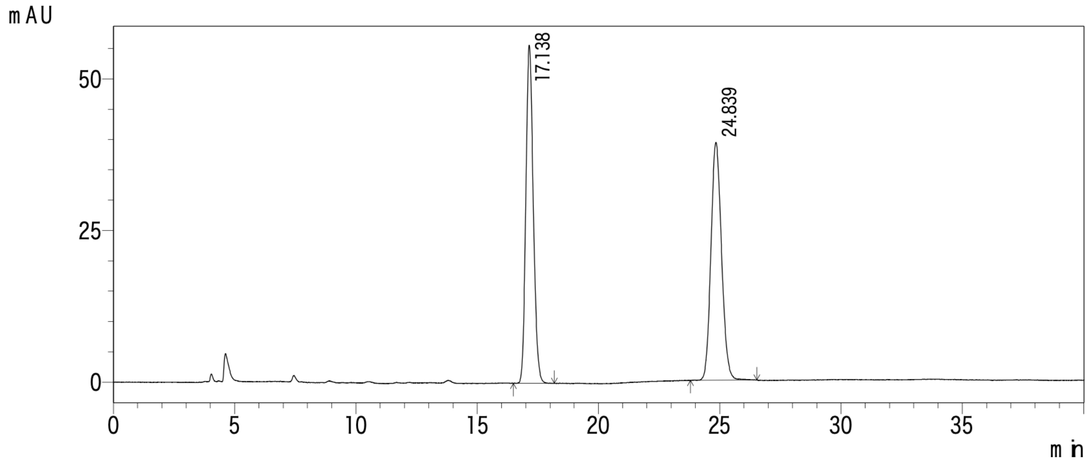

PDA Ch1 210nm

Peak No.	RT (min)	Area	Height	%Area
1	17.138	1183923	55776	49.834
2	24.839	1191830	39180	50.166
Total		2375753	94956	100.000

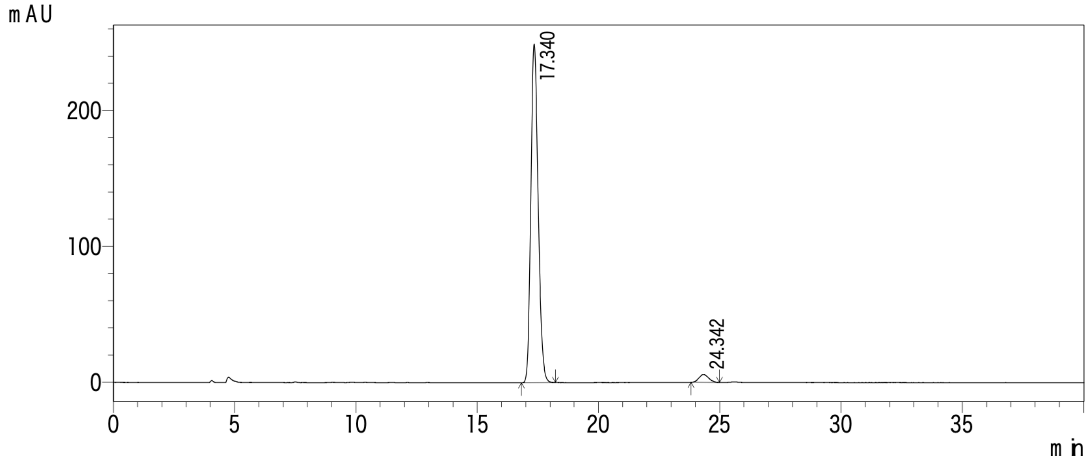

PDA Ch1 210nm

Peak No.	RT (min)	Area	Height	%Area
1	17.340	5223970	249226	96.997
2	24.342	161707	5785	3.003
Total		5385677	255011	100.000

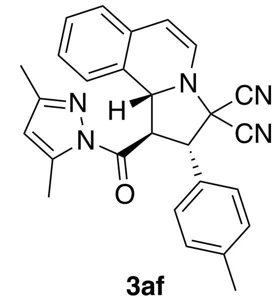

3af

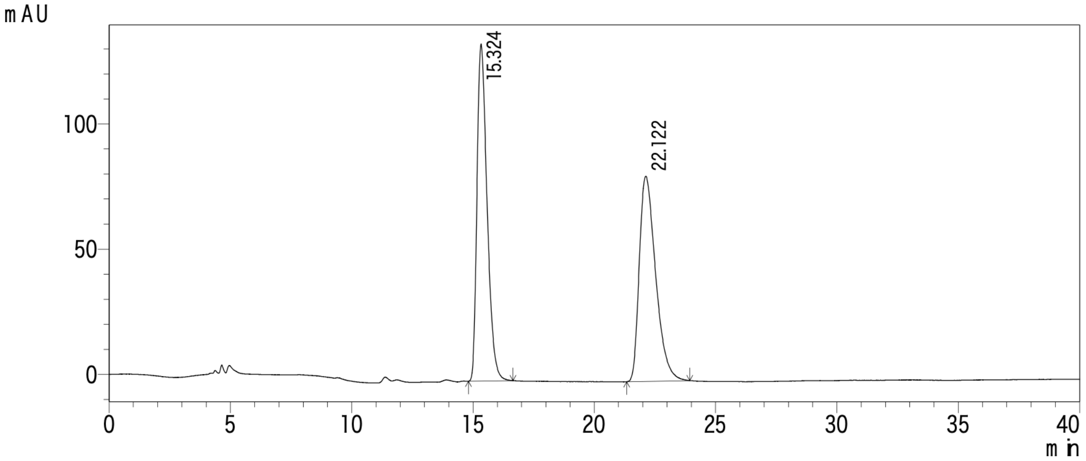

PDA Ch1 210nm

Peak No.	RT (min)	Area	Height	% Area
1	15.324	3889834	134683	50.326
2	22.122	3839366	81899	49.674
Total		7729200	216582	100.000

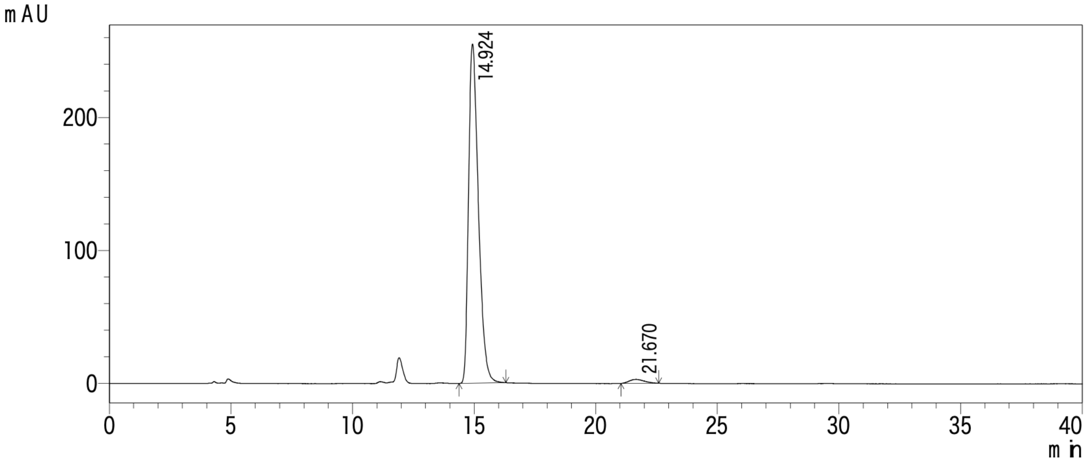

PDA Ch1 210nm

Peak No.	RT (min)	Area	Height	% Area
1	14.924	7179393	255016	98.268
2	21.670	126565	2968	1.732
Total		7305958	257984	100.000

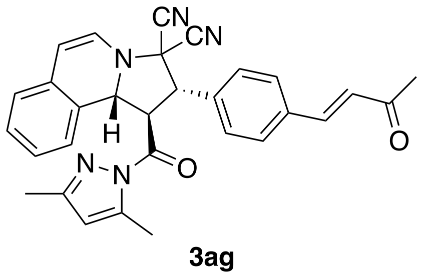

3ag

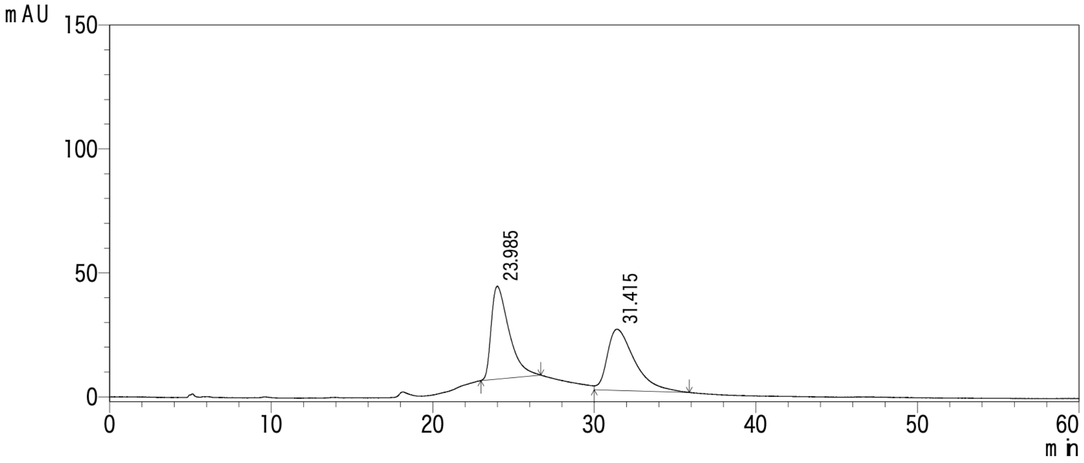

PDA Ch1 210nm

Peak No.	RT (min)	Area	Height	% Area
1	23.985	2878328	37466	49.566
2	31.415	2928721	24758	50.434
Total		5807049	62224	100.000

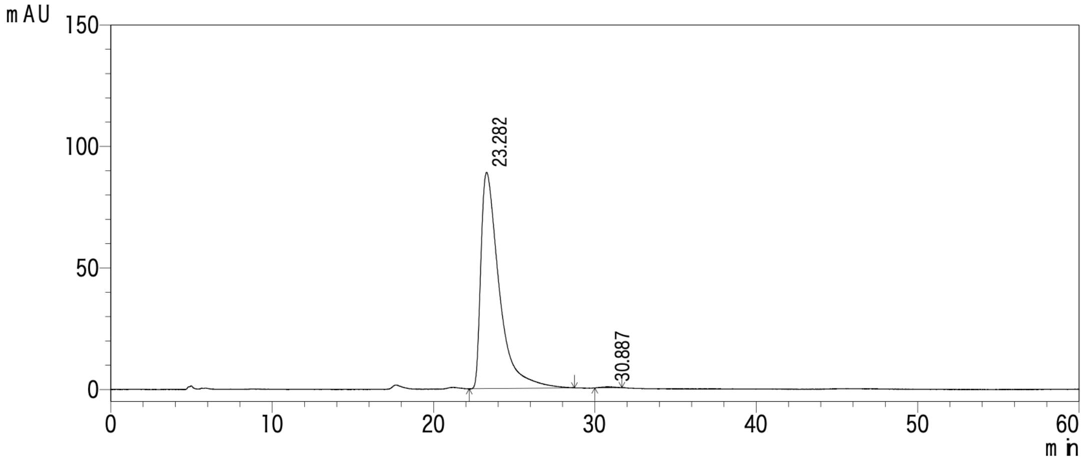

PDA Ch1 210nm

Peak No.	RT (min)	Area	Height	% Area
1	23.282	7015802	89047	99.692
2	30.887	21665	396	0.308
Total		7037467	89443	100.000

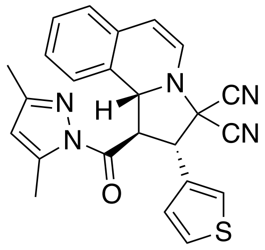

3ah

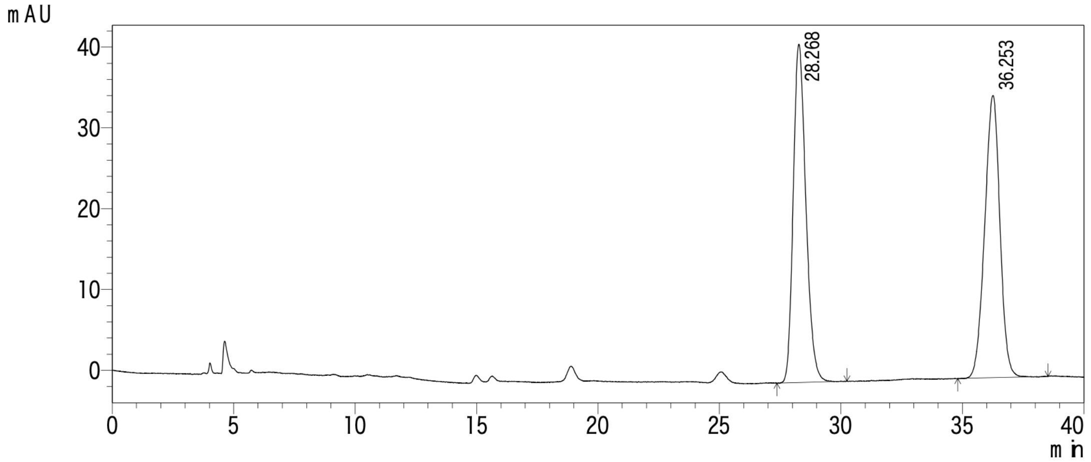

PDA Ch1 210nm

Peak No.	RT (min)	Area	Height	%Area
1	28.268	1511136	41857	49.919
2	36.253	1516026	34921	50.081
Total		3027162	76778	100.000

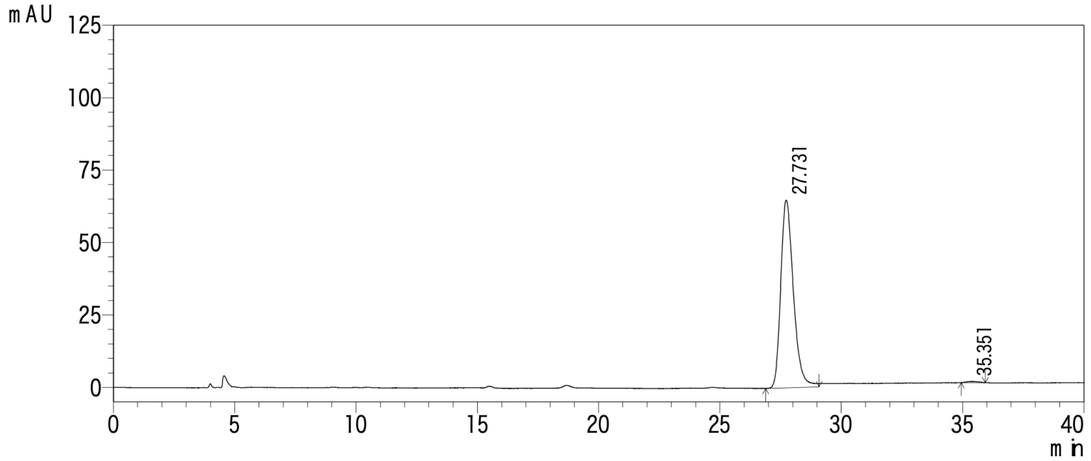

PDA Ch1 210nm

Peak No.	RT (min)	Area	Height	%Area
1	27.731	2348973	64759	99.405
2	35.351	14067	435	0.595
Total		2363039	65195	100.000

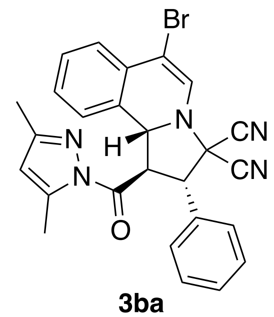

3ba

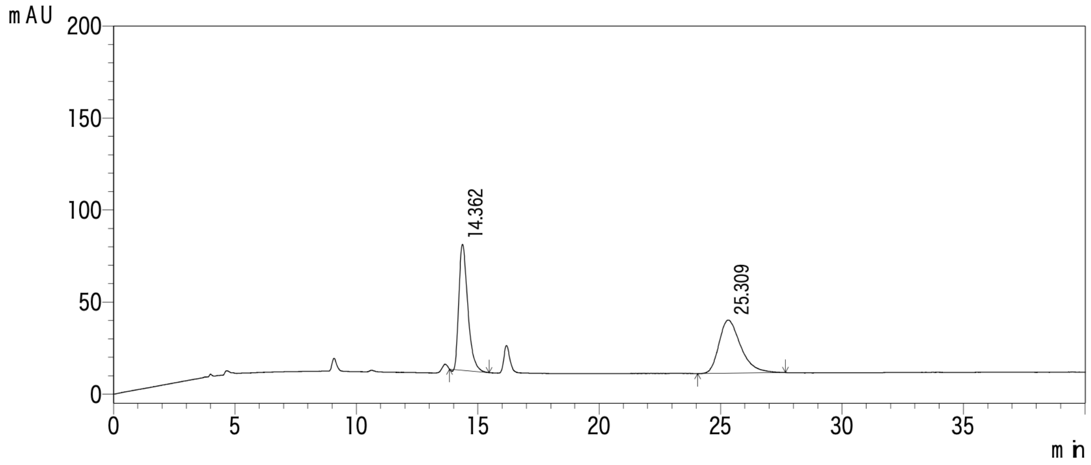

PDA Ch1 210nm

Peak No.	RT (min)	Area	Height	% Area
1	14.362	1744605	68508	49.272
2	25.309	1796172	28848	50.728
Total		3540777	97356	100.000

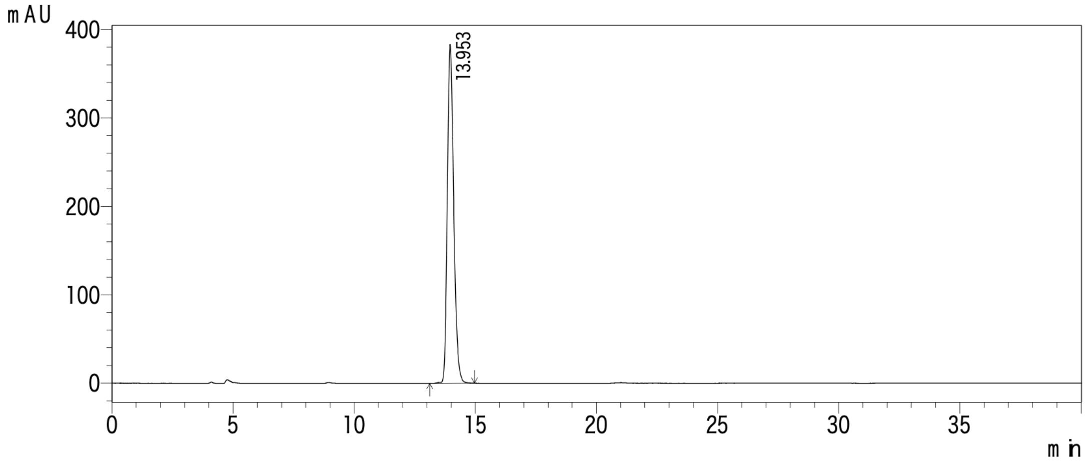

PDA Ch1 210nm

Peak No.	RT (min)	Area	Height	% Area
1	13.953	7031637	383320	100.000
Tota		7031637	383320	100.000

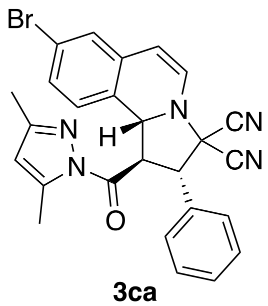

3ca

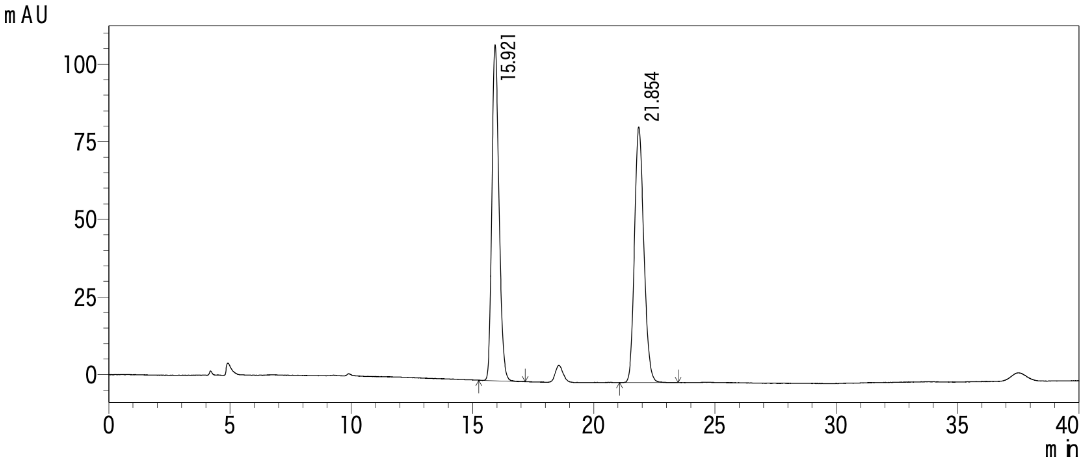

PDA Ch1 210nm

Peak No.	RT (min)	Area	Height	% Area
1	15.921	2209964	108242	50.003
2	21.854	2209661	82282	49.997
Total		4419624	190524	100.000

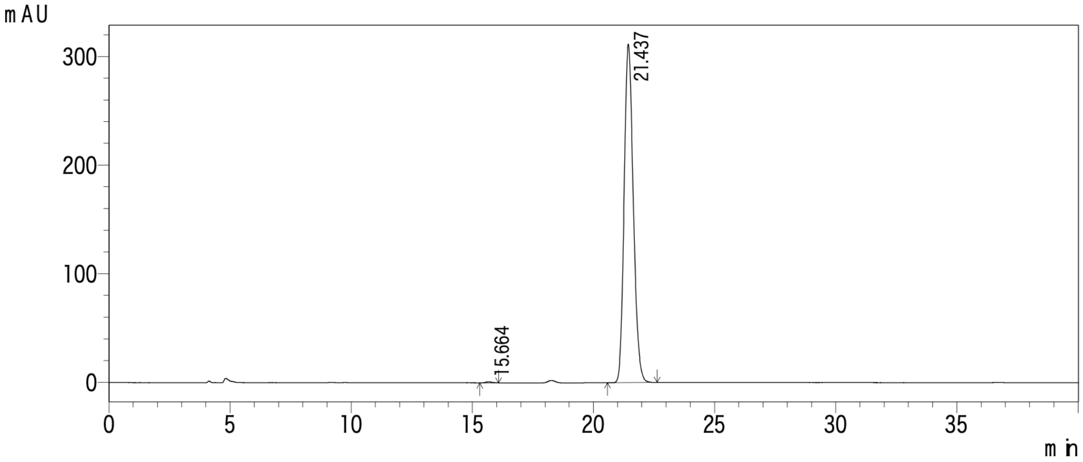

PDA Ch1 210nm

Peak No.	RT (min)	Area	Height	% Area
1	15.664	18488	979	0.225
2	21.437	8194733	311592	99.775
Total		8213222	312571	100.000

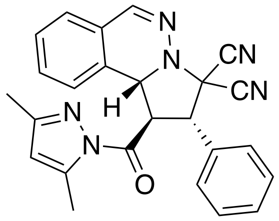

3da

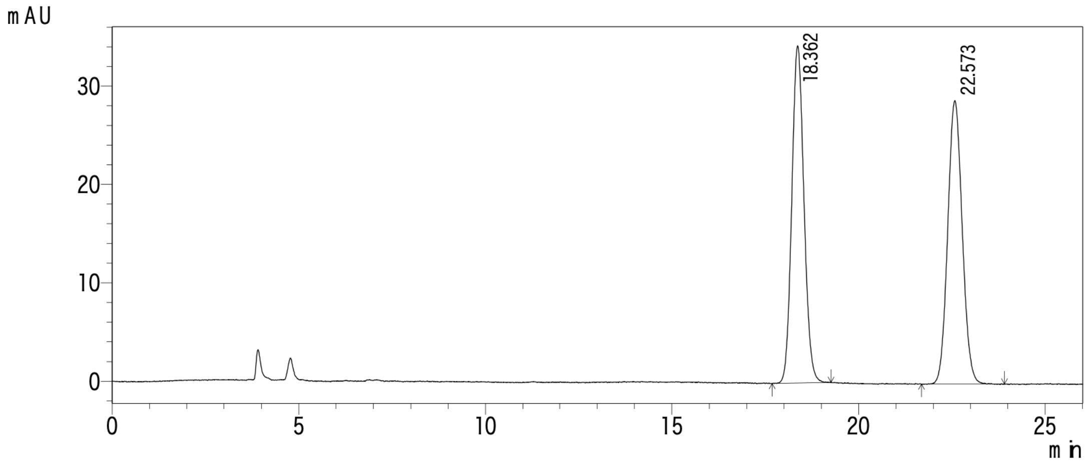

PDA Ch1 210nm

Peak No.	RT (min)	Area	Height	%Area
1	18.362	775812	34290	49.944
2	22.573	777545	28821	50.056
Total		1553357	63111	100.000

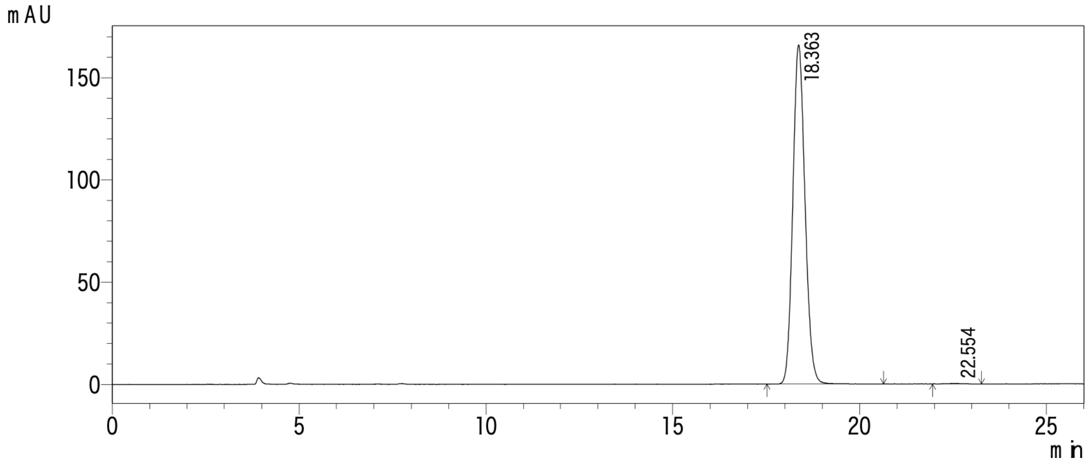

PDA Ch1 210nm

Peak No.	RT (min)	Area	Height	%Area
1	18.363	3754401	165946	99.863
2	22.554	5136	241	0.137
Total		3759537	166187	100.000

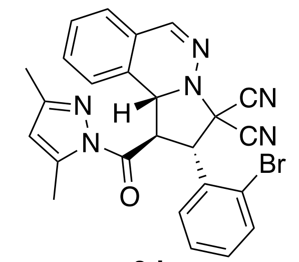

3de

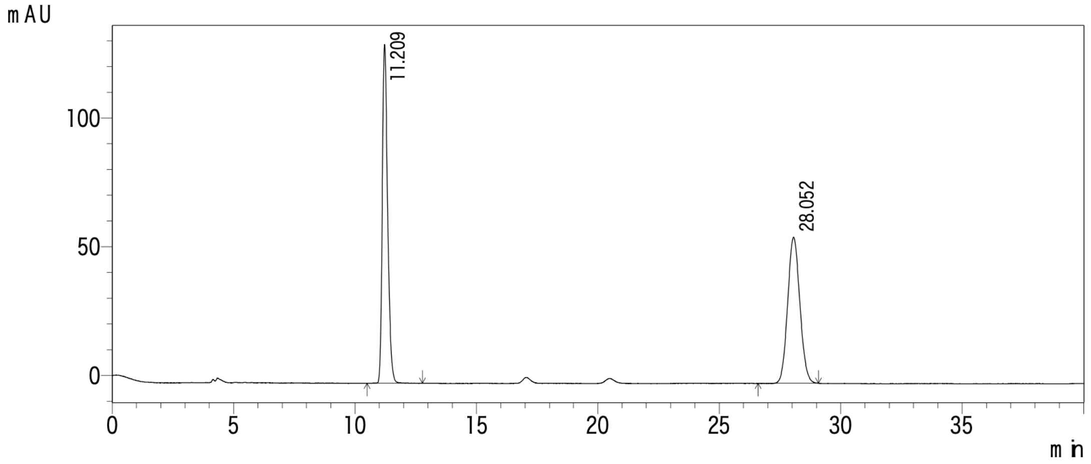

PDA Ch1 210nm

Peak No.	RT (min)	Area	Height	% Area
1	11.209	2001089	131698	50.248
2	28.052	1981369	56855	49.752
Total		3982459	188553	100.000

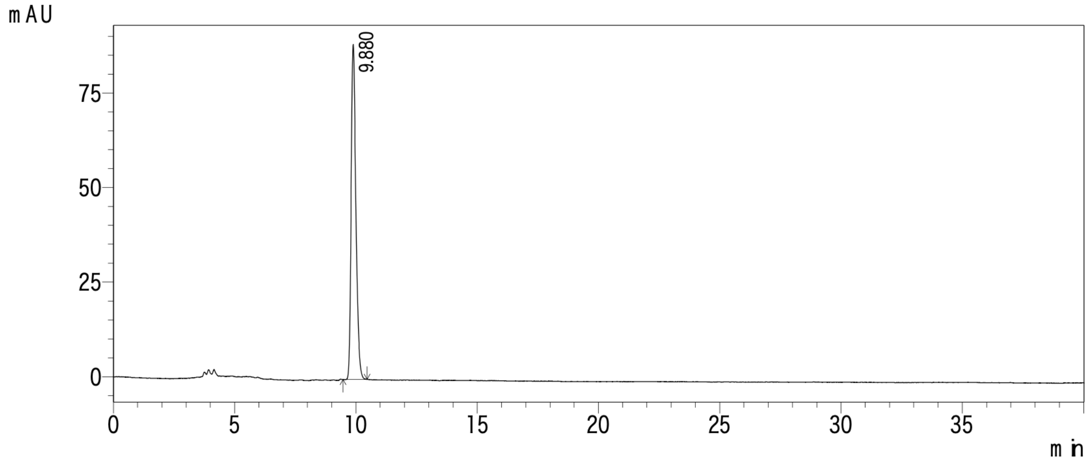

PDA Ch1 210nm

Peak No.	RT (min)	Area	Height	% Area
1	9.880	1201435	88576	100.000
Total		1201435	88576	100.000

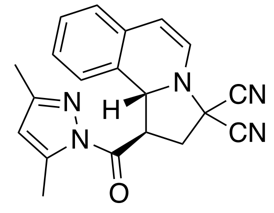

3ai

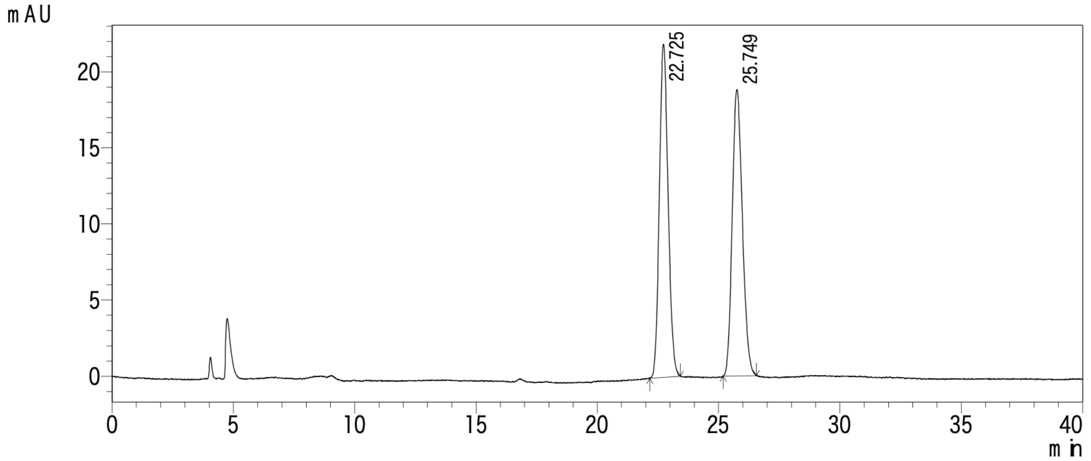

PDA Ch1 210nm

Peak No.	RT (min)	Area	Height	% Area
1	22.725	547794	21906	50.042
2	25.749	546883	18823	49.958
Total		1094677	40729	100.000

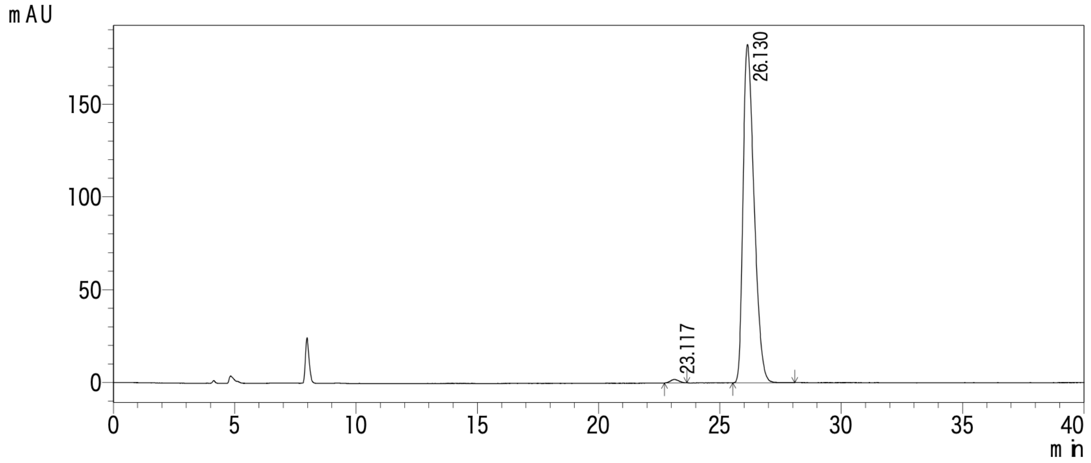

PDA Ch1 210nm

Peak No.	RT (min)	Area	Height	% Area
1	23.117	45707	1869	0.787
2	26.130	5763235	182355	99.213
Total		5808942	184224	100.000

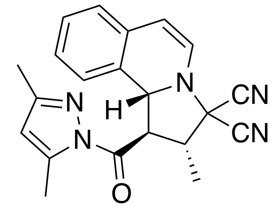

3aj

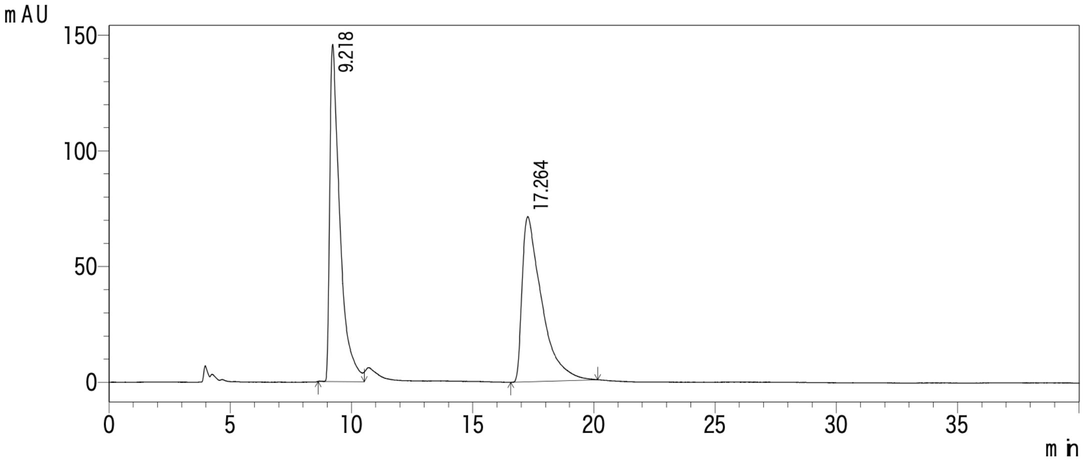

PDA Ch1 210nm

Peak No.	RT (min)	Area	Height	%Area
1	9.218	4243433	145762	51.399
2	17.264	4012410	71400	48.601
Total		8255842	217162	100.000

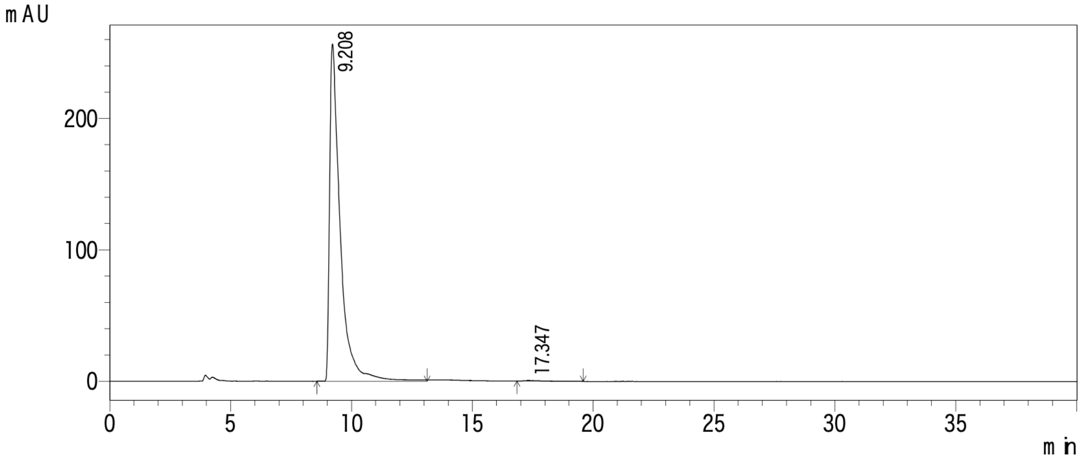

PDA Ch1 210nm

Peak No.	RT (min)	Area	Height	%Area
1	9.208	7851822	256708	99.733
2	17.347	21003	442	0.267
Total		7872825	257151	100.000

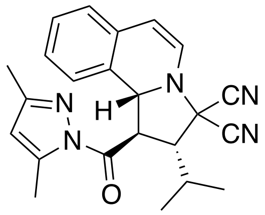

3ak

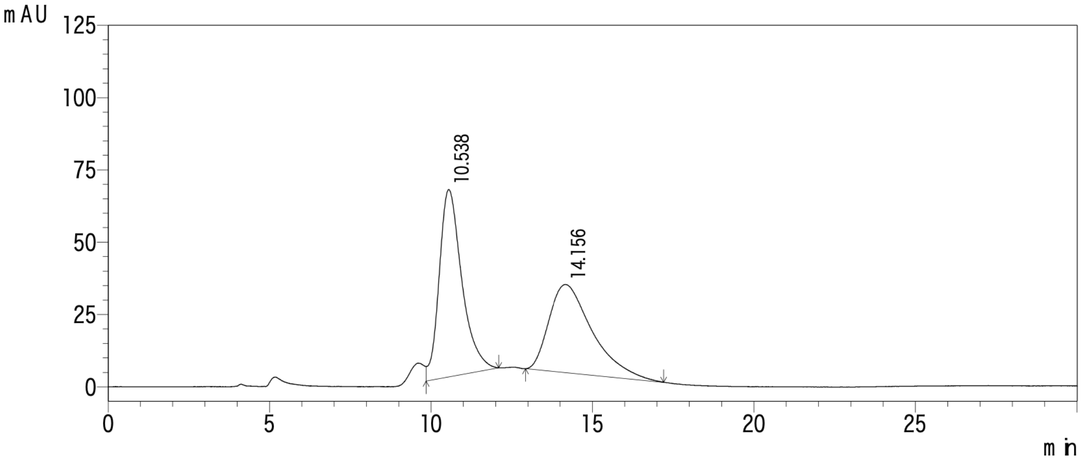

PDA Ch1 210nm

Peak No.	RT (min)	Area	Height	% Area
1	10.538	3168080	64804	52.658
2	14.156	2848227	30473	47.342
Total		6016307	95277	100.000

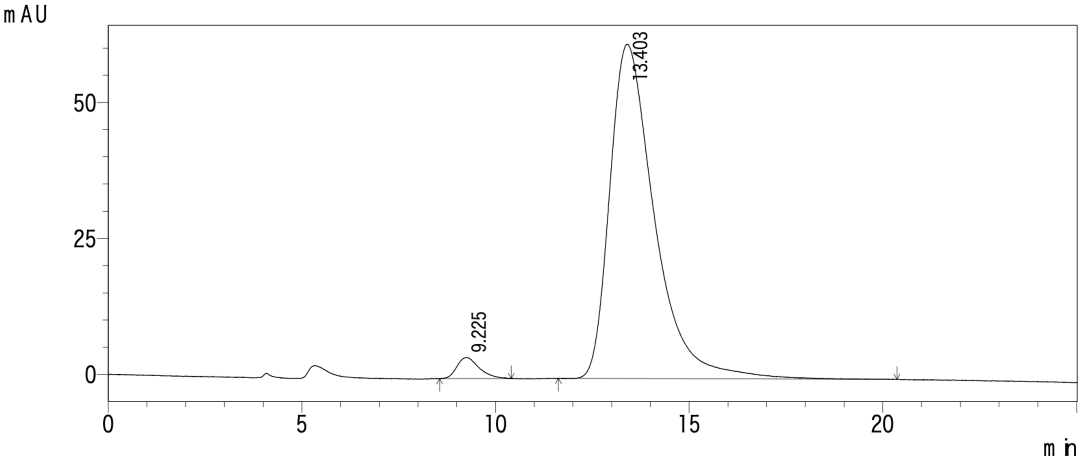

PDA Ch1 210nm

Peak No.	RT (min)	Area	Height	% Area
1	9.225	154707	3873	3.036
2	13.403	4940719	61447	96.964
Total		5095427	65320	100.000

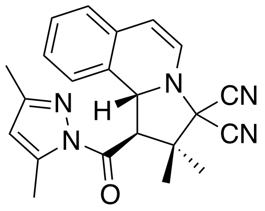

3al

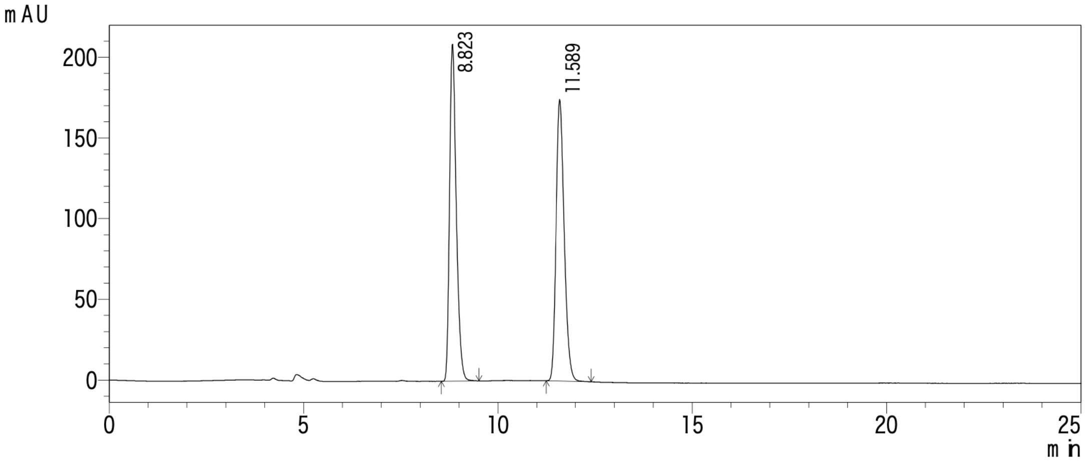

PDA Ch1 210nm

Peak No.	RT (min)	Area	Height	% Area
1	8.823	2537856	208799	49.779
2	11.589	2560369	174534	50.221
Total		5098225	383332	100.000

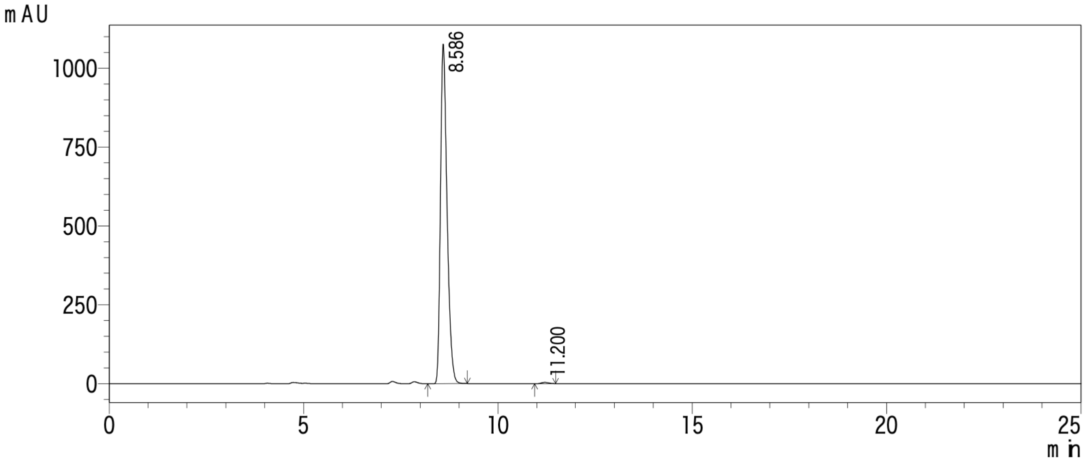

PDA Ch1 210nm

Peak No.	RT (min)	Area	Height	% Area
1	8.586	12660942	1076877	99.573
2	11.200	54314	4046	0.427
Total		12715255	1080922	100.000

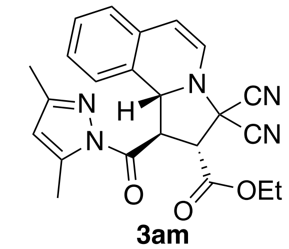

3am

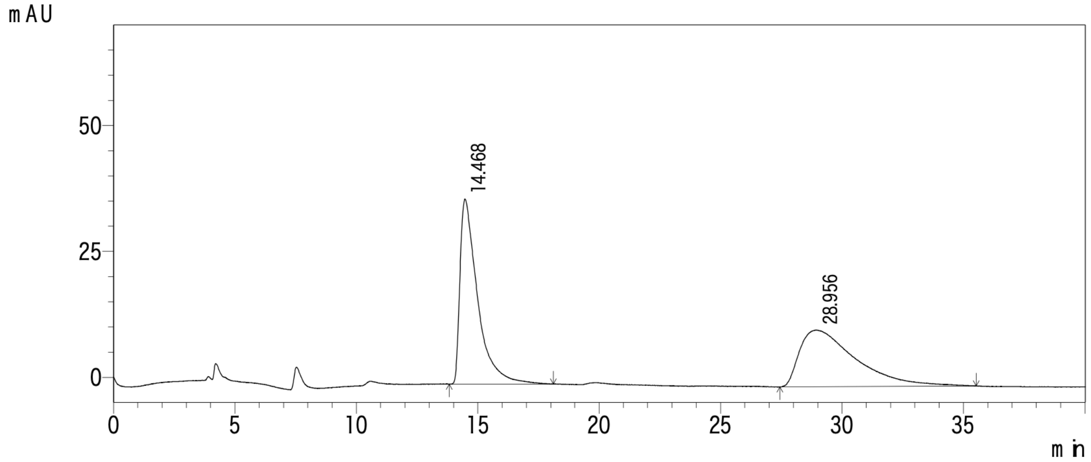

PDA Ch1 210nm

Peak No.	RT (min)	Area	Height	%Area
1	14.468	1858350	36798	50.815
2	28.956	1798738	11249	49.185
Total		3657088	48047	100.000

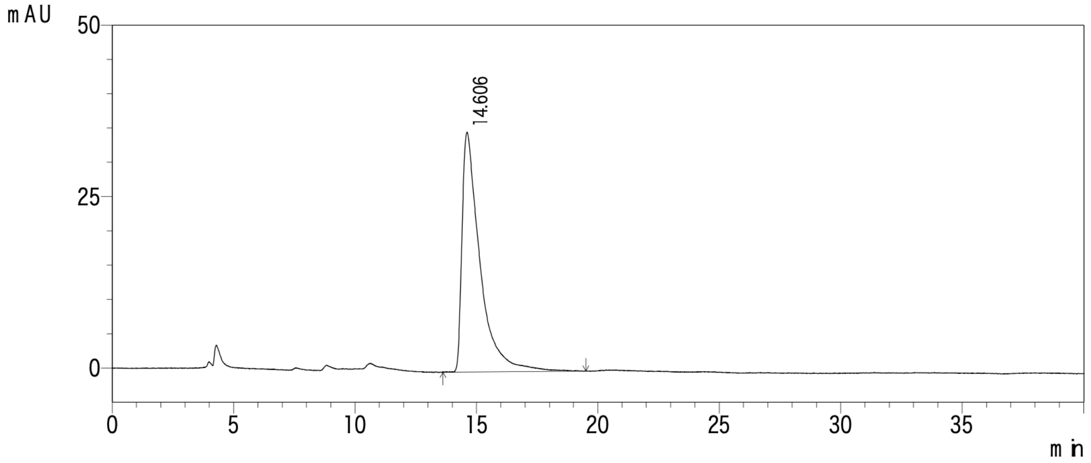

PDA Ch1 210nm

Peak No.	RT (min)	Area	Height	%Area
1	14.606	1798382	34992	100.000
Total		1798382	34992	100.000

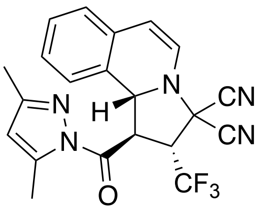

3an

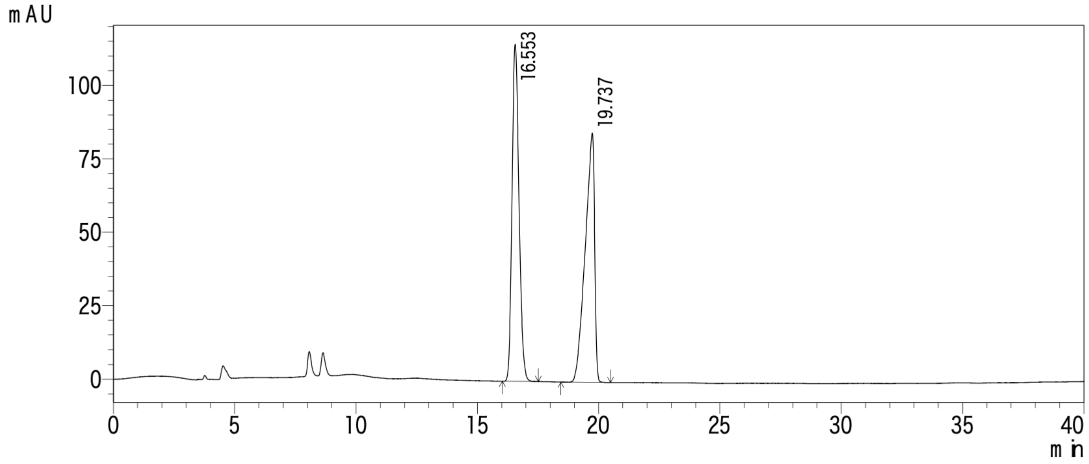

PDA Ch1 210nm

Peak No.	RT (min)	Area	Height	% Area
1	16.553	2284412	114746	50.043
2	19.737	2280523	84797	49.957
Total		4564935	199543	100.000

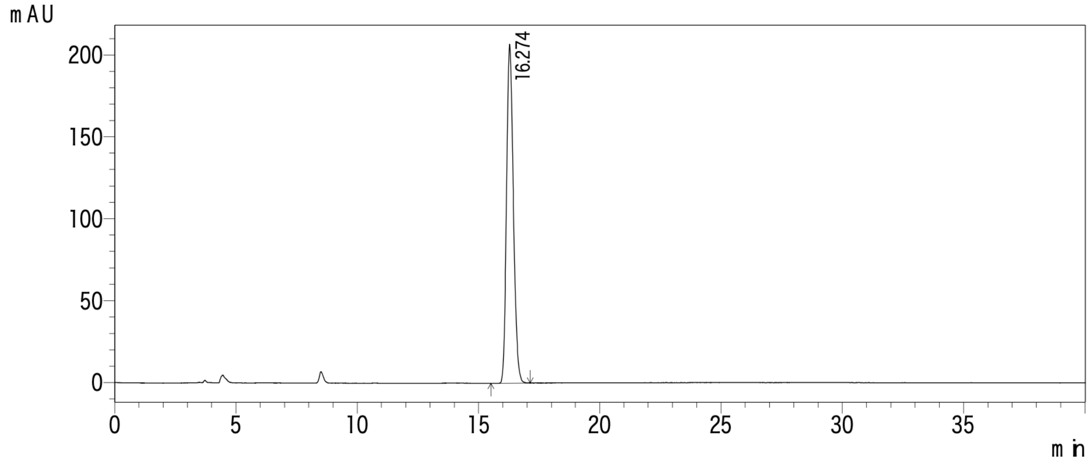

PDA Ch1 210nm

Peak No.	RT (min)	Area	Height	% Area
1	16.274	4033147	206943	100.000
Total		4033147	206943	100.000

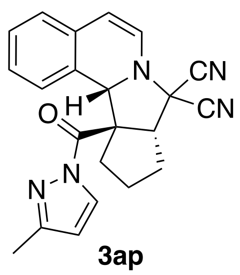

3ap

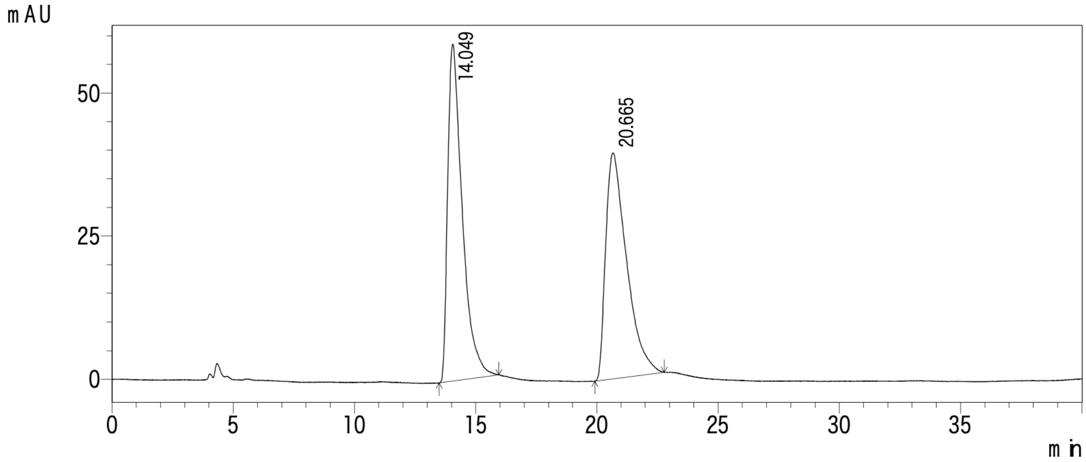

PDA Ch1 210nm

Peak No.	RT (min)	Area	Height	% Area
1	14.049	2455788	58802	50.966
2	20.665	2362730	39421	49.034
Total		4818518	98223	100.000

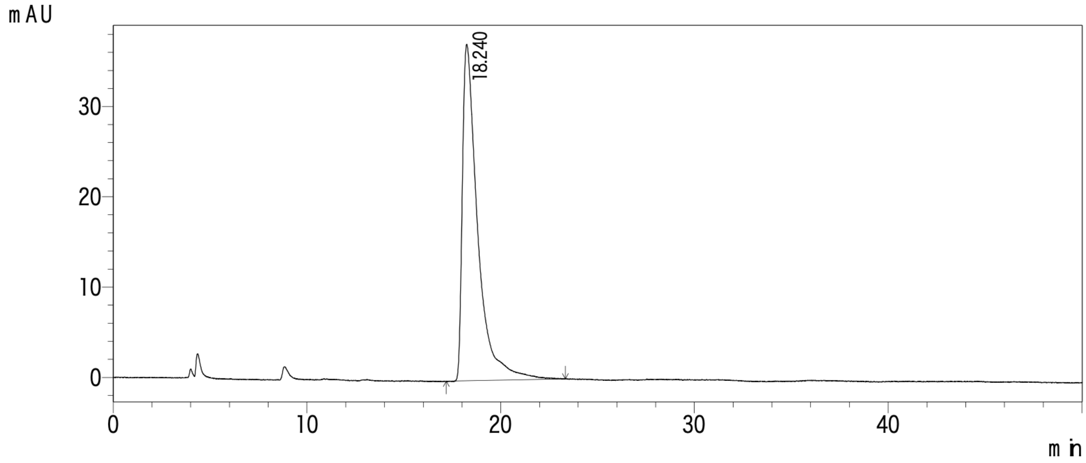

PDA Ch1 210nm

Peak No.	RT (min)	Area	Height	% Area
1	18.240	2048963	37279	100.000
Total		2048963	37279	100.000

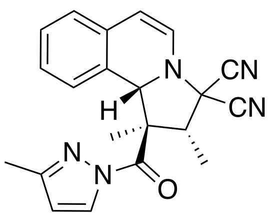

3aq

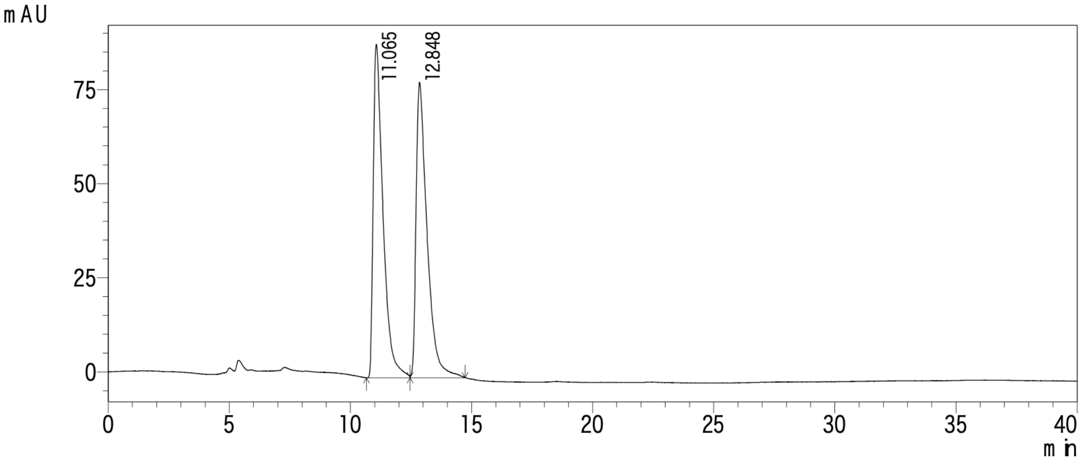

PDA Ch1 210nm

Peak No.	RT (min)	Area	Height	% Area
1	11.065	2488449	88633	50.179
2	12.848	2470651	78538	49.821
Total		4959101	167171	100.000

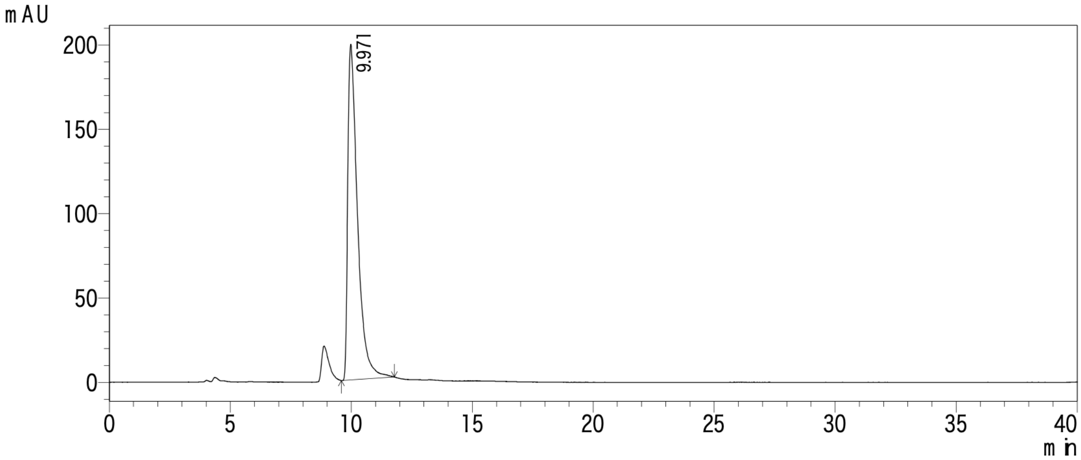

PDA Ch1 210nm

Peak No.	RT (min)	Area	Height	% Area
1	9.971	5565173	199066	100.000
Total		5565173	199066	100.000

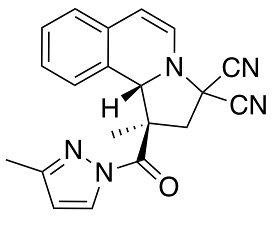

3ar

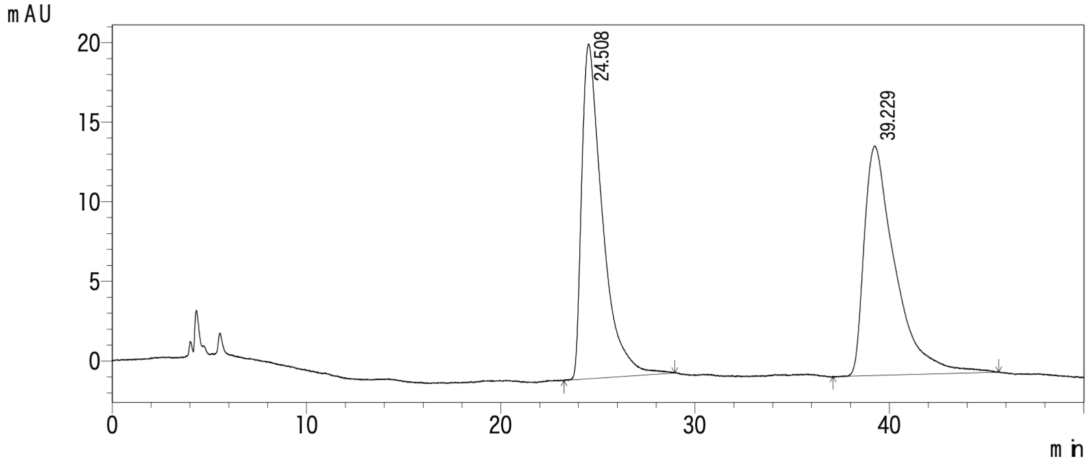

PDA Ch1 210nm

Peak No.	RT (min)	Area	Height	% Area
1	24.508	1564182	21040	49.445
2	39.229	1599317	14413	50.555
Total		3163498	35453	100.000

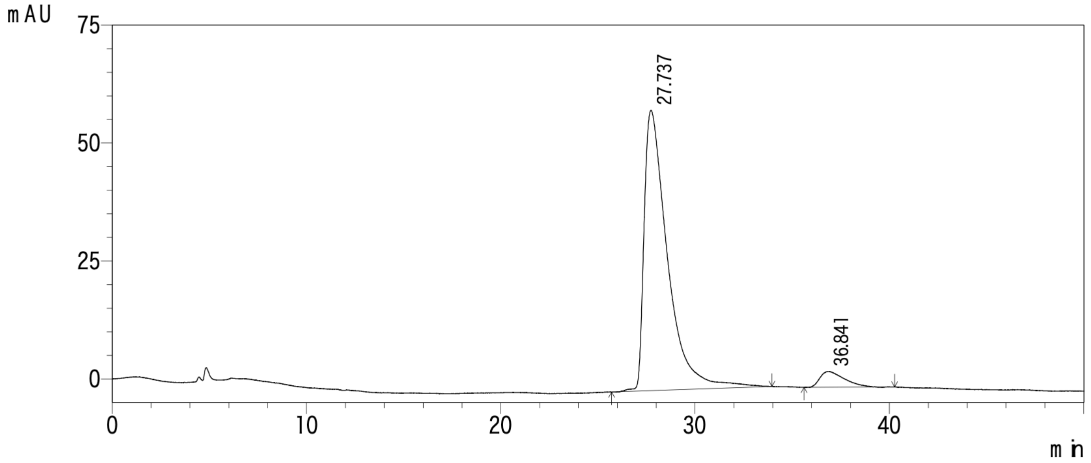

PDA Ch1 210nm

Peak No.	RT (min)	Area	Height	% Area
1	27.737	4967348	59416	94.510
2	36.841	288525	3362	5.490
Total		5255874	62778	100.000

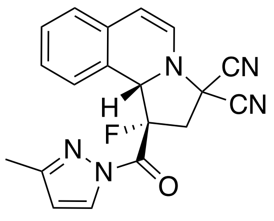

3as

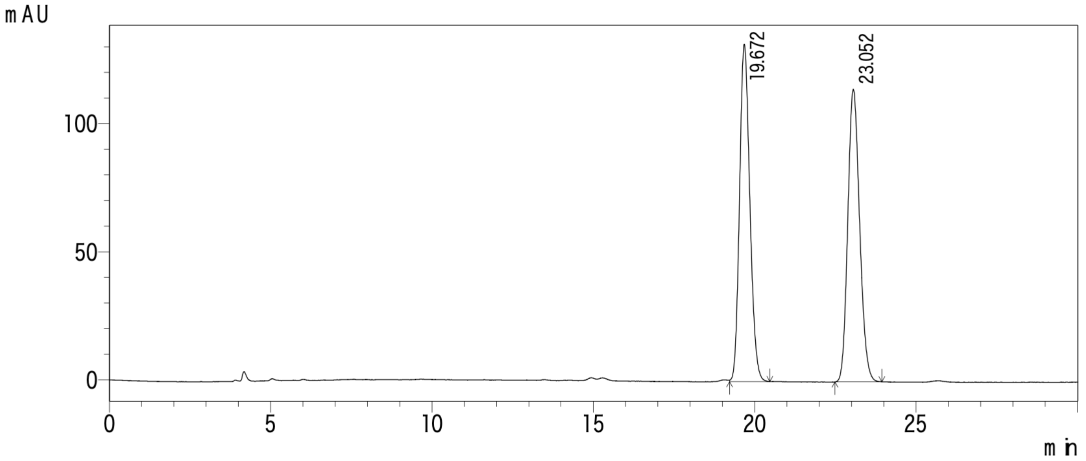

PDA Ch1 210nm

Peak No.	RT (min)	Area	Height	% Area
1	19.672	2911435	131705	50.028
2	23.052	2908126	114275	49.972
Total		5819562	245980	100.000

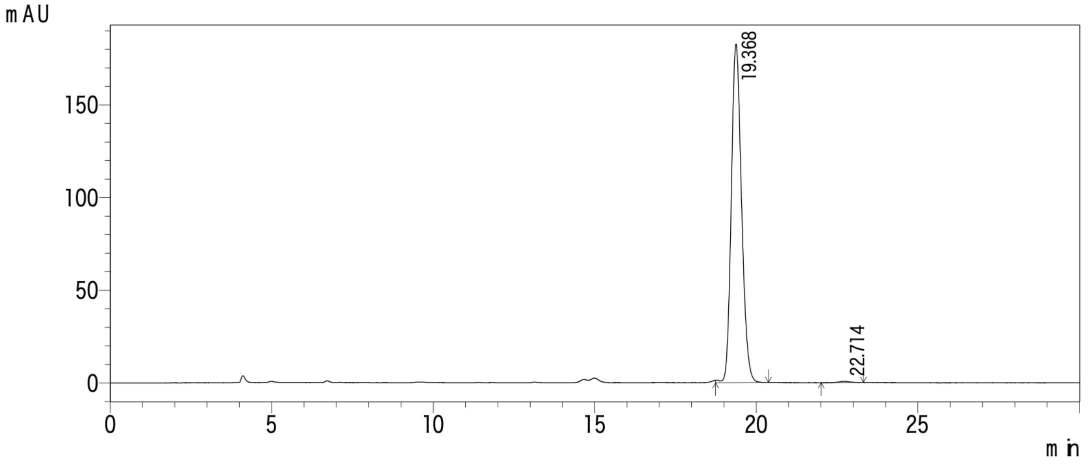

PDA Ch1 210nm

Peak No.	RT (min)	Area	Height	% Area
1	19.368	4035036	182753	99.645
2	22.714	14391	636	0.355
Total		4049428	183389	100.000

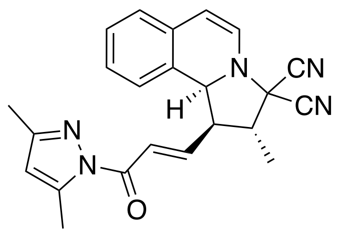

6aa

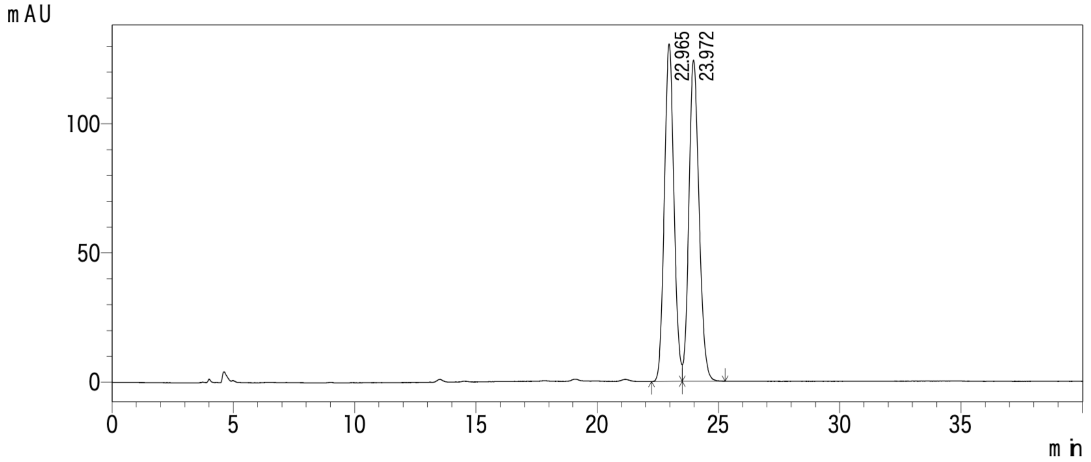

PDA Ch1 210nm

Peak No.	RT (min)	Area	Height	% Area
1	22.965	3585502	130720	49.719
2	23.972	3625988	124417	50.281
Total		7211490	255137	100.000

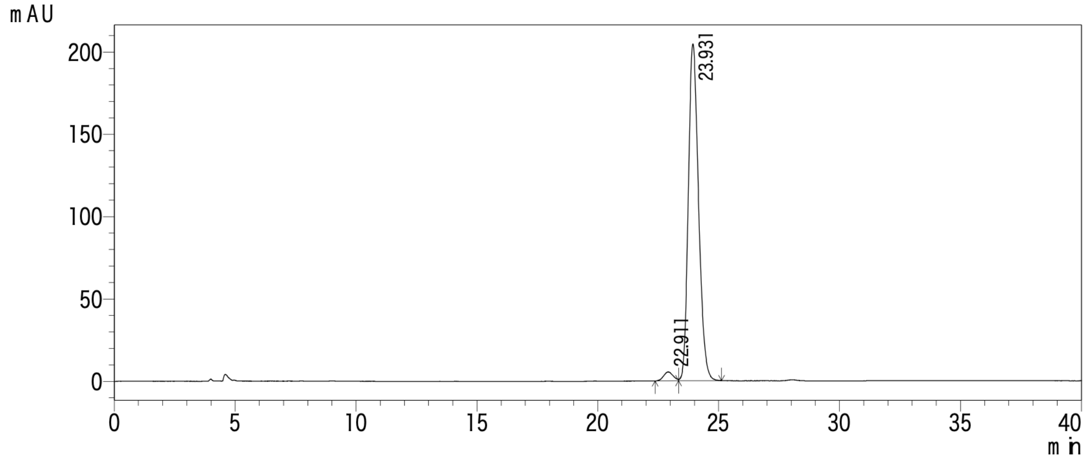

PDA Ch1 210nm

Peak No.	RT (min)	Area	Height	% Area
1	22.911	148195	5395	2.410
2	23.931	6002214	204641	97.590
Total		6150409	210036	100.000

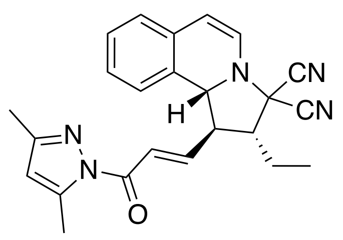

6ab

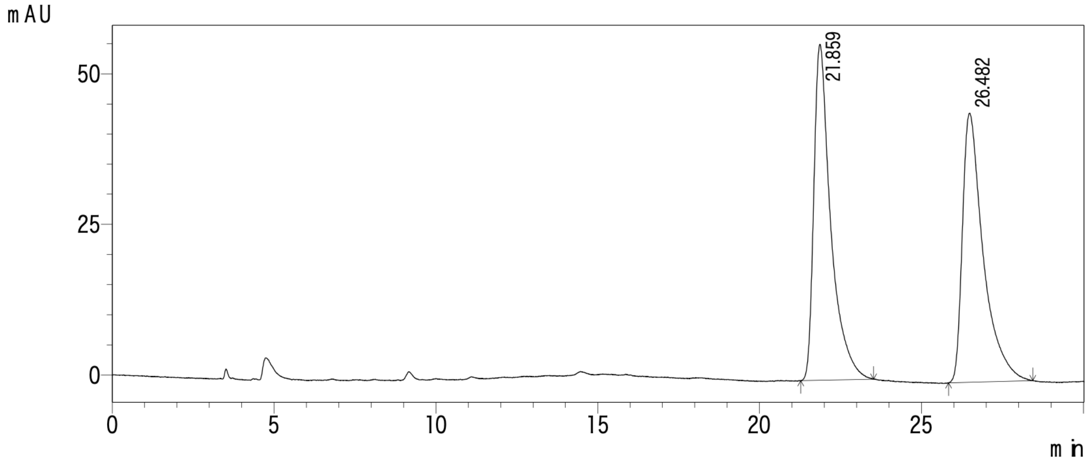

PDA Ch1 210nm

Peak No.	RT (min)	Area	Height	% Area
1	21.859	2031008	55811	50.400
2	26.482	1998807	44711	49.600
Total		4029815	100522	100.000

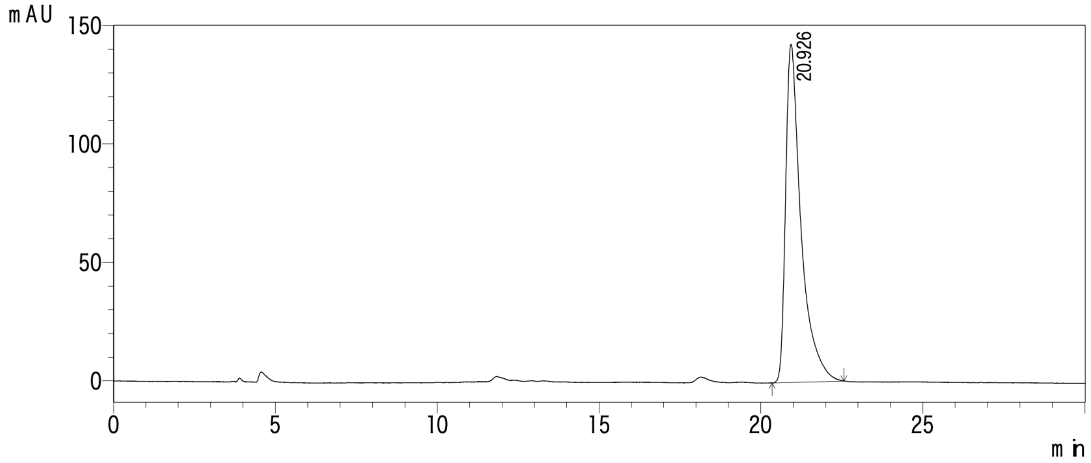

PDA Ch1 210nm

Peak No.	RT (min)	Area	Height	% Area
1	20.926	4727437	142783	100.000
Total		4727437	142783	100.000

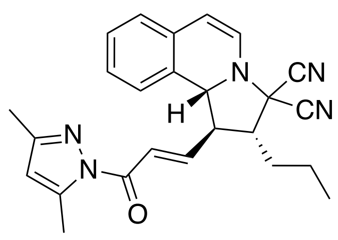

exo-**6ac**

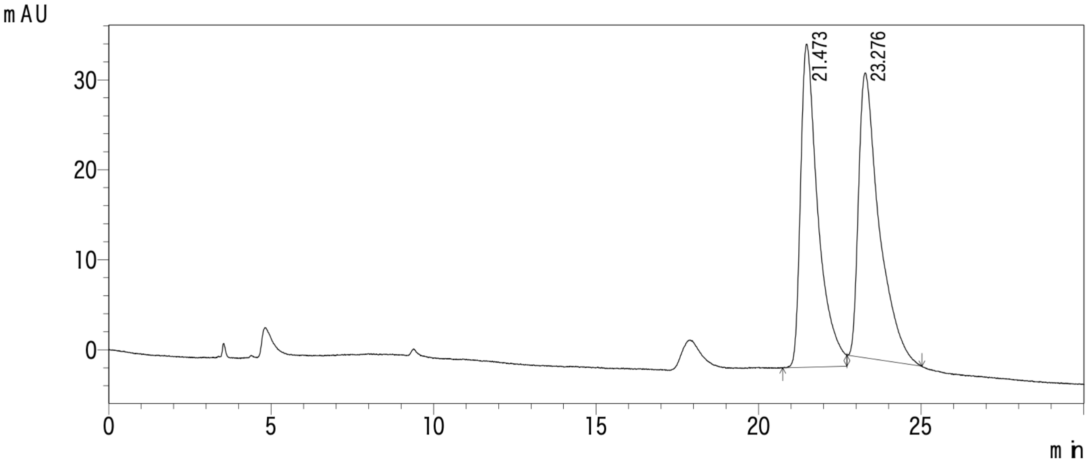

PDA Ch1 210nm

Peak No.	RT (min)	Area	Height	%Area
1	21.473	1346291	35921	49.106
2	23.276	1395338	31674	50.894
Total		2741629	67595	100.000

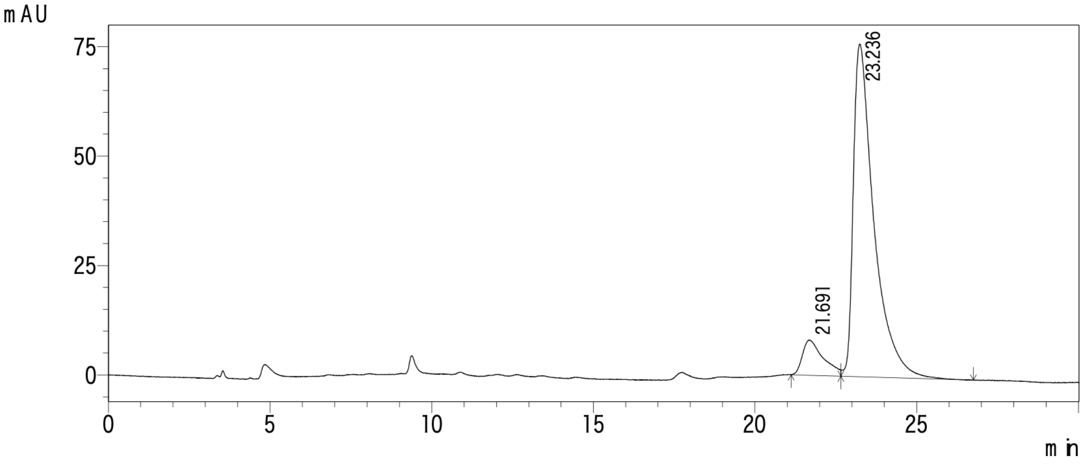

PDA Ch1 210nm

Peak No.	RT (min)	Area	Height	%Area
1	21.691	359162	8023	9.640
2	23.236	3366619	76010	90.360
Total		3725782	84032	100.000

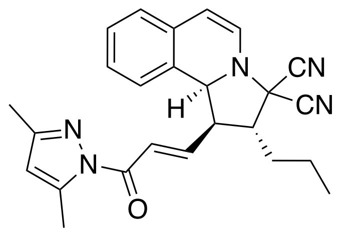

endo-**6ac**

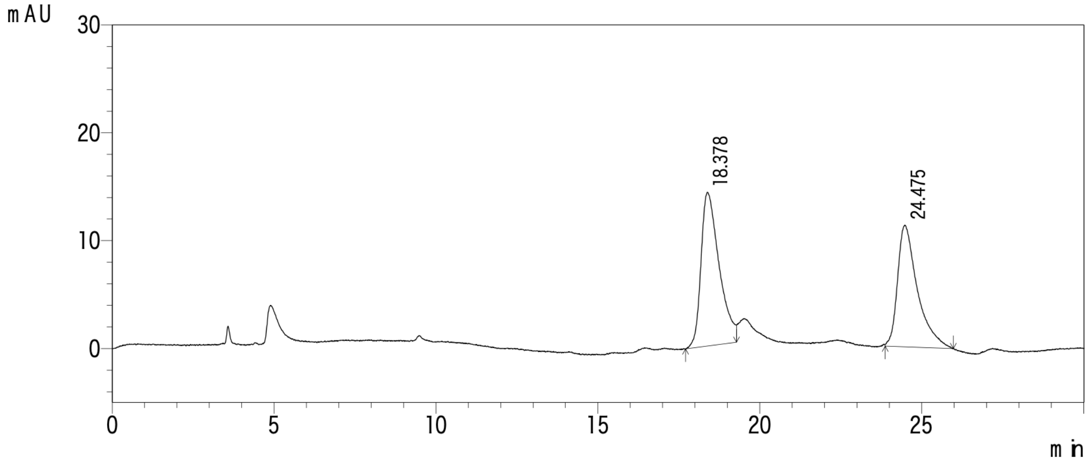

PDA Ch1 210nm

Peak No.	RT (min)	Area	Height	% Area
1	18.378	555750	14263	53.616
2	24.475	480785	11290	46.384
Total		1036535	25553	100.000

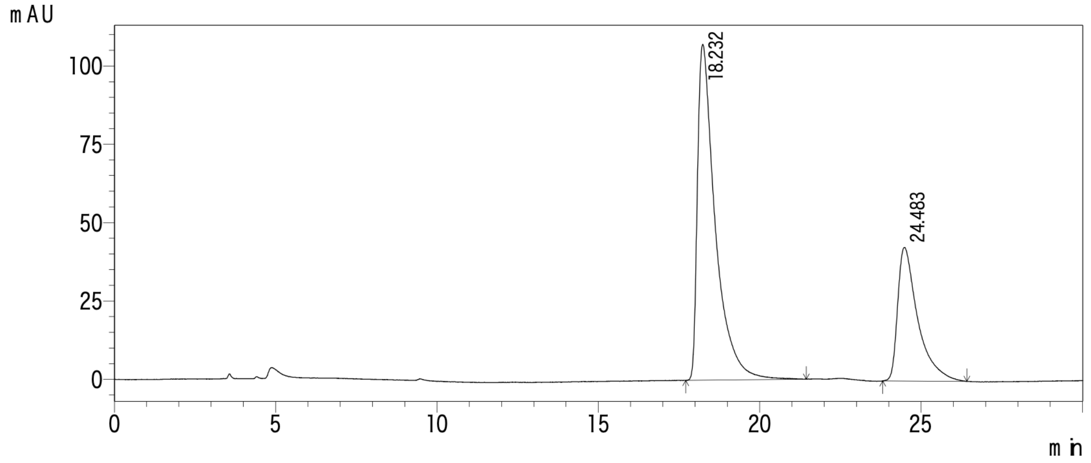

PDA Ch1 210nm

Peak No.	RT (min)	Area	Height	% Area
1	18.232	4112041	107257	69.036
2	24.483	1844335	42636	30.964
Total		5956377	149894	100.000

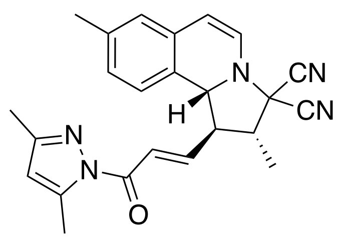

6ea

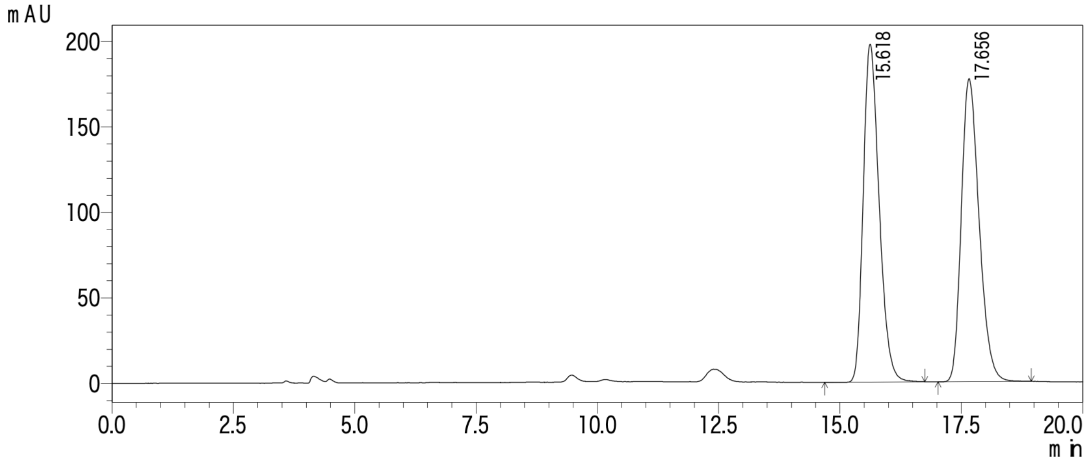

PDA Ch1 210nm

Peak No.	RT (min)	Area	Height	% Area
1	15.618	4497732	197626	49.918
2	17.656	4512490	177280	50.082
Total		9010222	374906	100.000

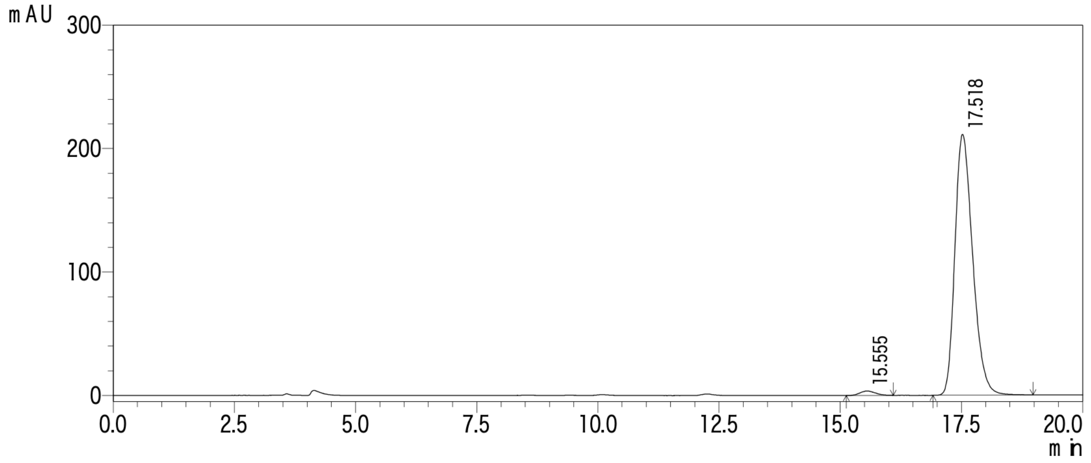

PDA Ch1 210nm

Peak No.	RT (min)	Area	Height	% Area
1	15.555	79754	3613	1.461
2	17.518	5379382	211240	98.539
Total		5459136	214853	100.000

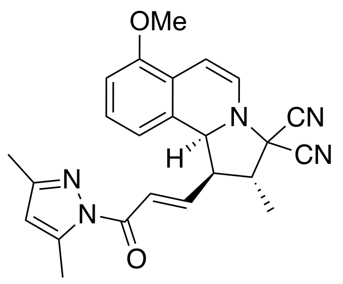

6fa

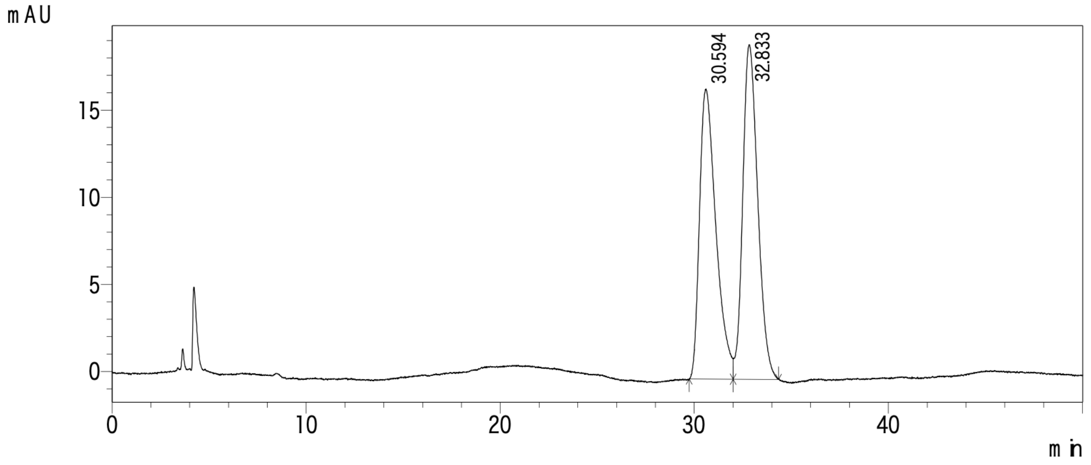

PDA Ch1 210nm

Peak No.	RT (min)	Area	Height	% Area
1	30.594	977706	16645	48.653
2	32.833	1031861	19205	51.347
Total		2009567	35849	100.000

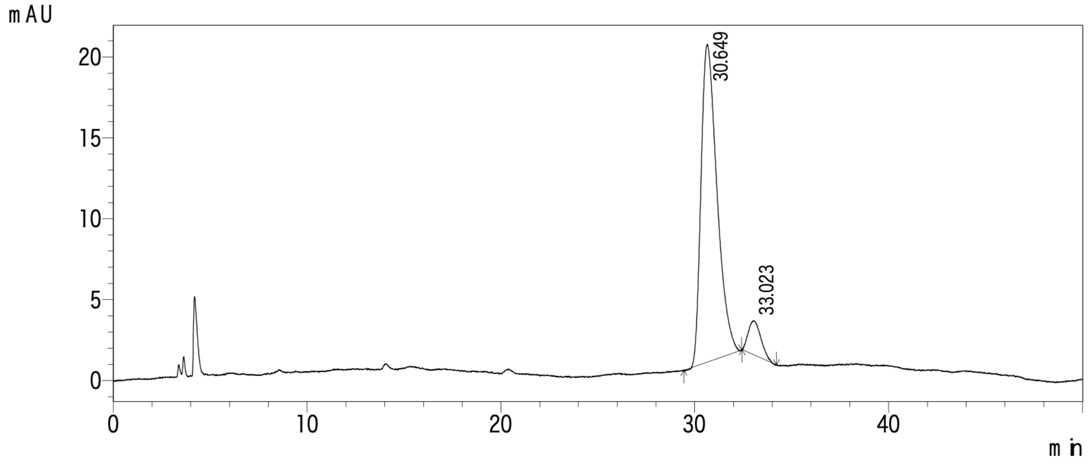

PDA Ch1 210nm

Peak No.	RT (min)	Area	Height	% Area
1	30.649	1123566	19640	92.192
2	33.023	95159	2090	7.808
Total		1218725	21729	100.000

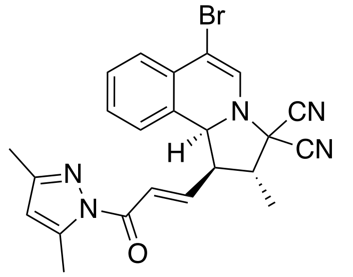

6ba

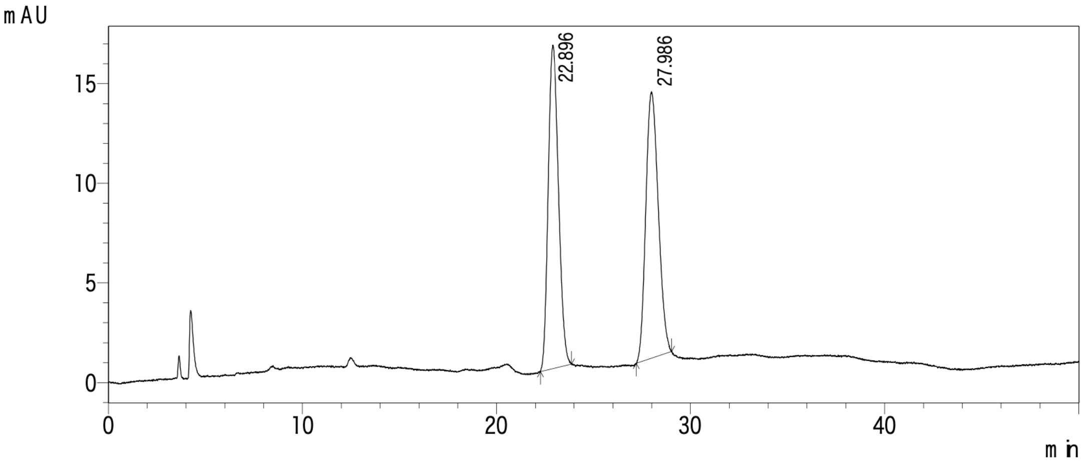

PDA Ch1 210nm

Peak No.	RT (min)	Area	Height	% Area
1	22.896	607764	16242	50.441
2	27.986	597142	13376	49.559
Total		1204906	29618	100.000

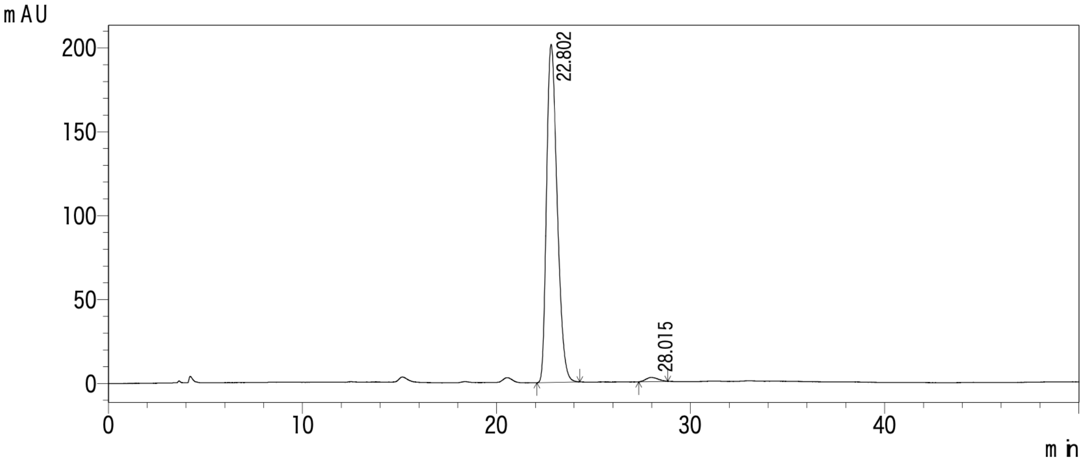

PDA Ch1 210nm

Peak No.	RT (min)	Area	Height	% Area
1	22.802	7771706	201632	98.651
2	28.015	106277	2457	1.349
Total		7877983	204088	100.000

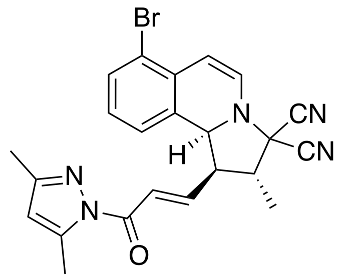

6ga

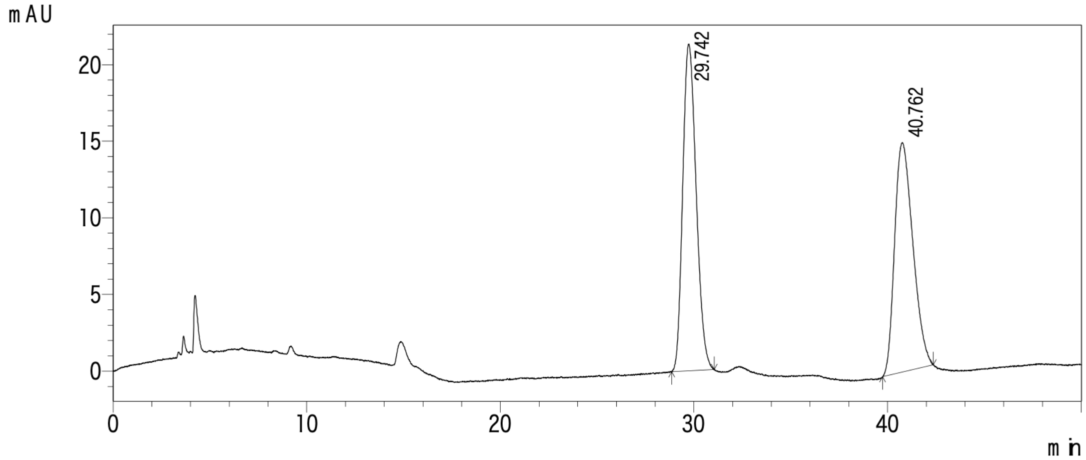

PDA Ch1 210nm

Peak No.	RT (min)	Area	Height	% Area
1	29.742	1027719	21329	51.203
2	40.762	979440	14972	48.797
Total		2007159	36300	100.000

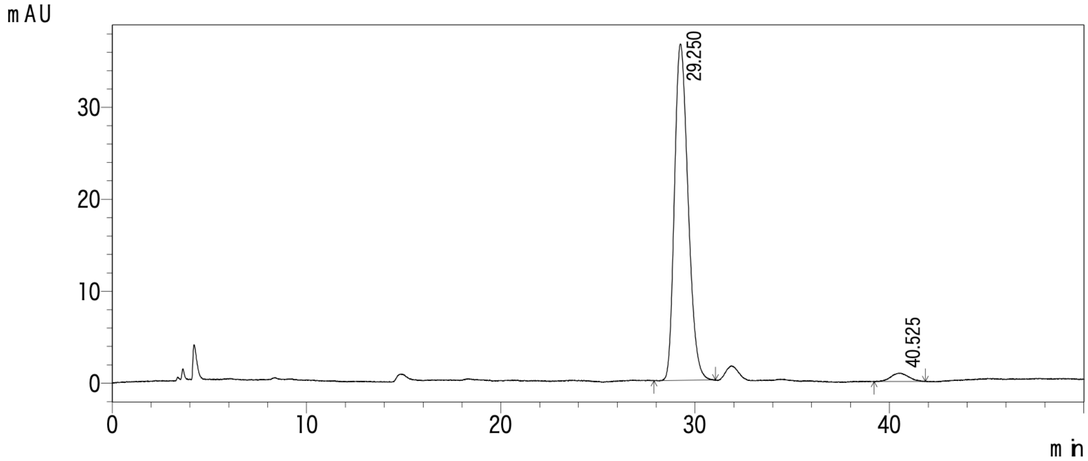

PDA Ch1 210nm

Peak No.	RT (min)	Area	Height	% Area
1	29.250	1785635	36599	96.862
2	40.525	57839	920	3.138
Total		1843475	37519	100.000

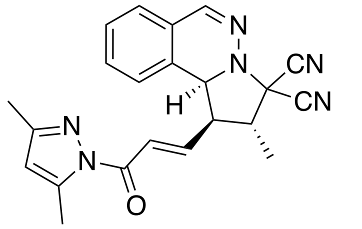

6da

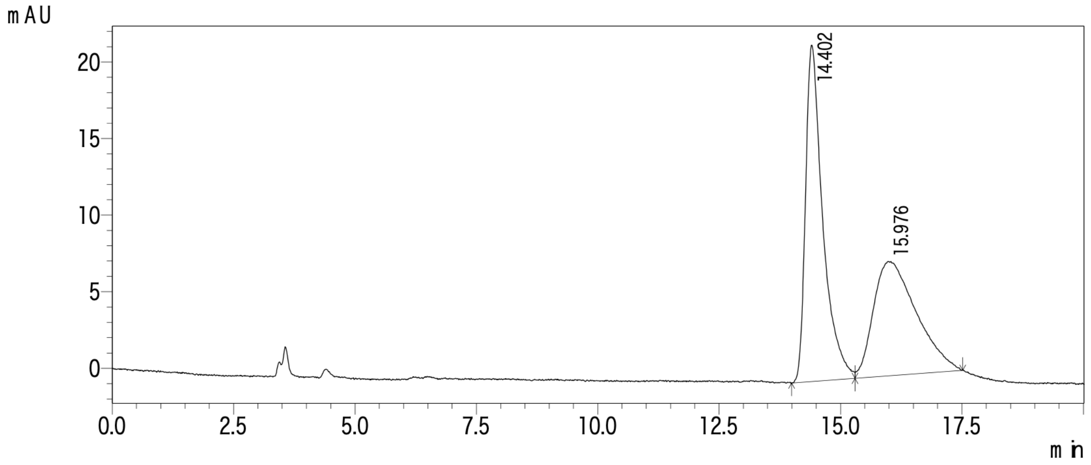

PDA Ch1 210nm

Peak No.	RT (min)	Area	Height	% Area
1	14.402	544071	21973	54.240
2	15.976	459017	7472	45.760
Total		1003089	29446	100.000

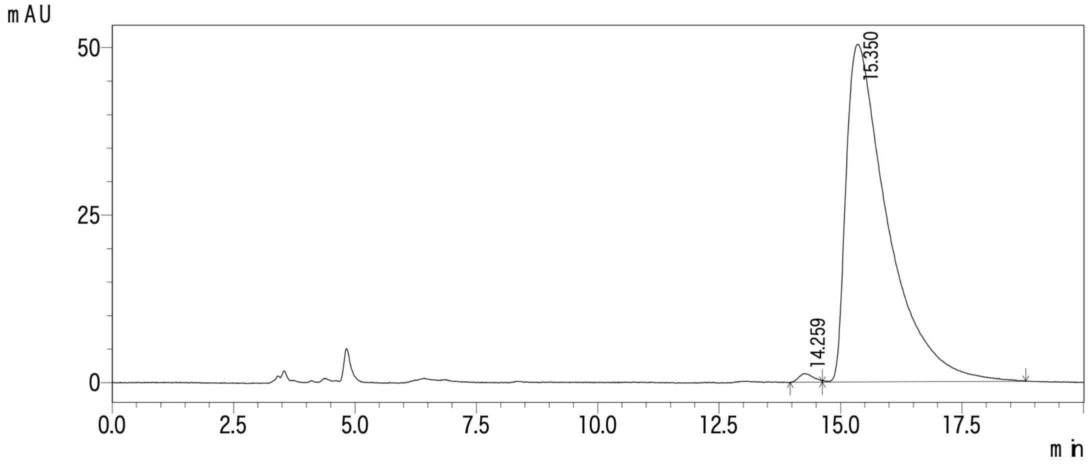

PDA Ch1 210nm

Peak No.	RT (min)	Area	Height	% Area
1	14.259	27227	1277	0.894
2	15.350	3017240	50420	99.106
Total		3044467	51697	100.000

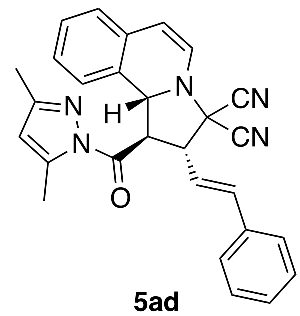

5ad

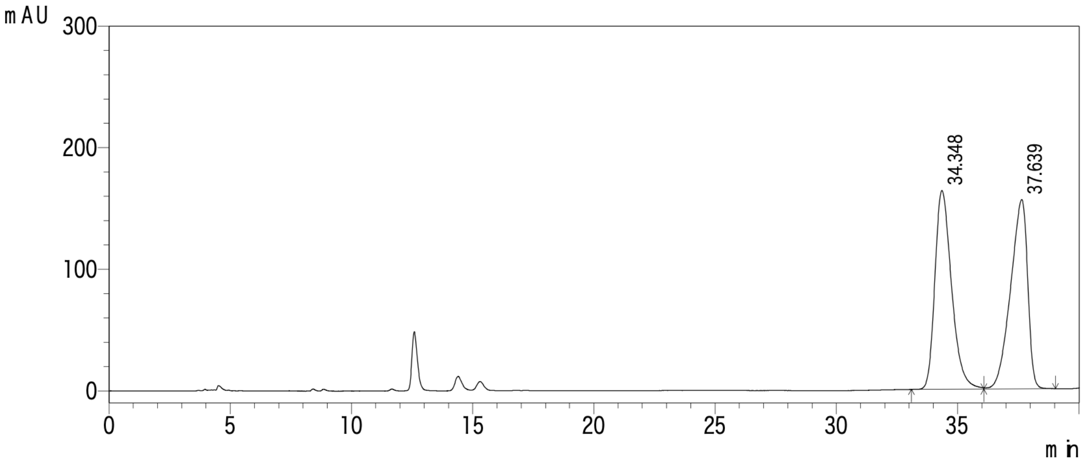

PDA Ch1 210nm

Peak No.	RT (min)	Area	Height	%Area
1	34.348	7816607	163545	50.643
2	37.639	7618070	155663	49.357
Total		15434677	319207	100.000

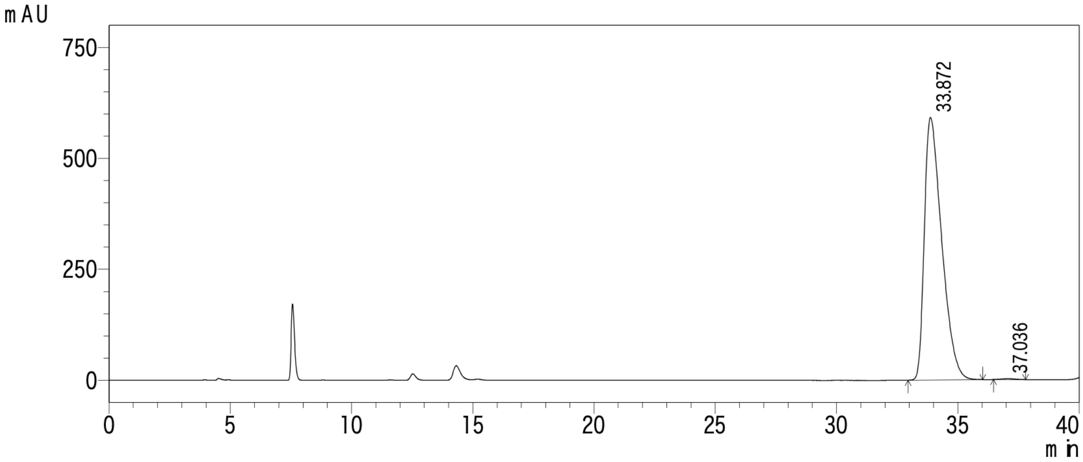

PDA Ch1 210nm

Peak No.	RT (min)	Area	Height	%Area
1	33.872	29439297	591577	99.720
2	37.036	82765	2038	0.280
Total		29522062	593615	100.000

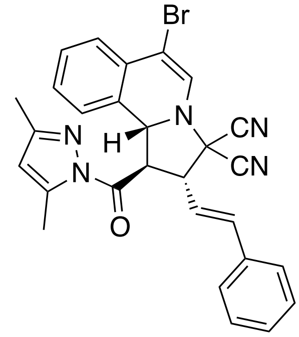

5bd

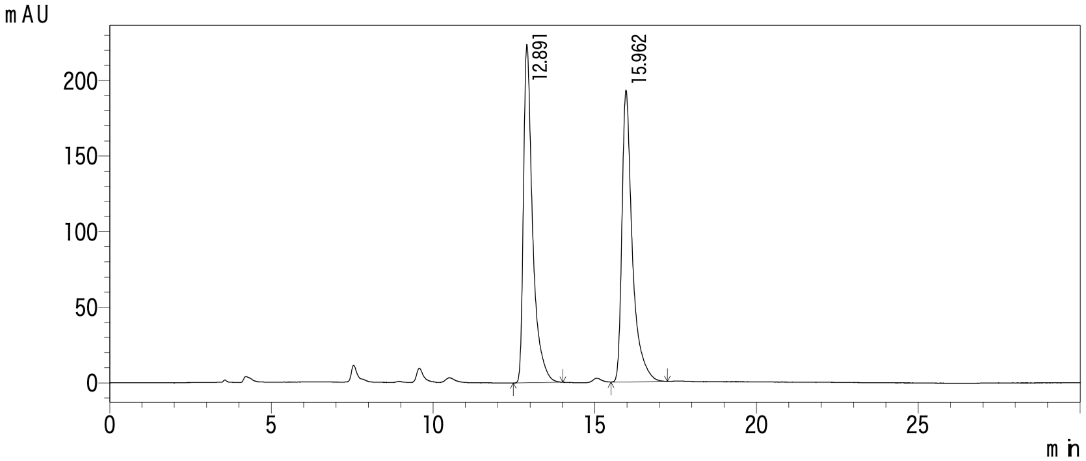

PDA Ch1 210nm

Peak No.	RT (min)	Area	Height	% Area
1	12.891	4394647	223988	50.492
2	15.962	4309031	193255	49.508
Total		8703678	417243	100.000

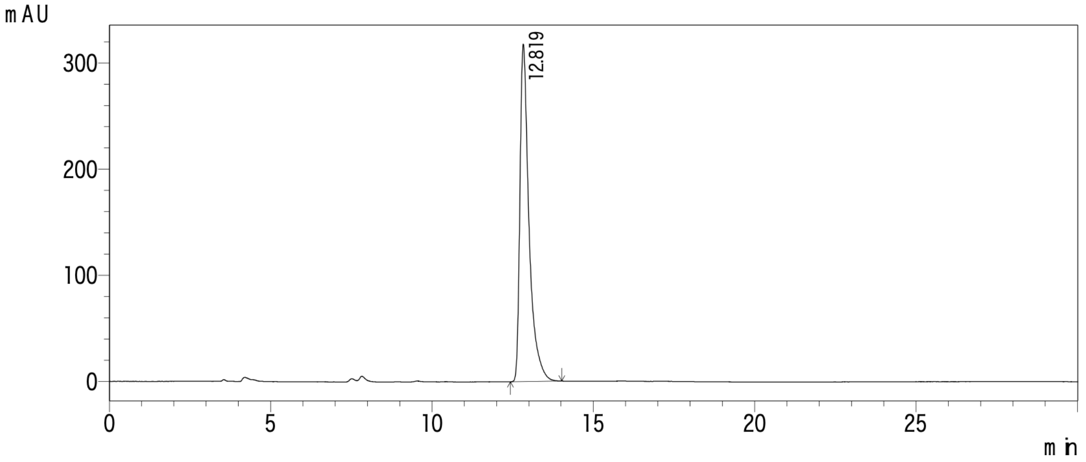

PDA Ch1 210nm

Peak No.	RT (min)	Area	Height	% Area
1	12.819	6226496	318223	100.000
Total		6226496	318223	100.000

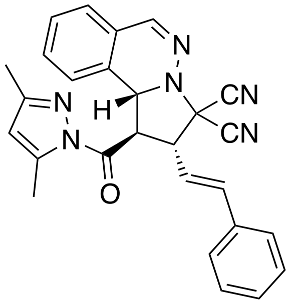

5dd

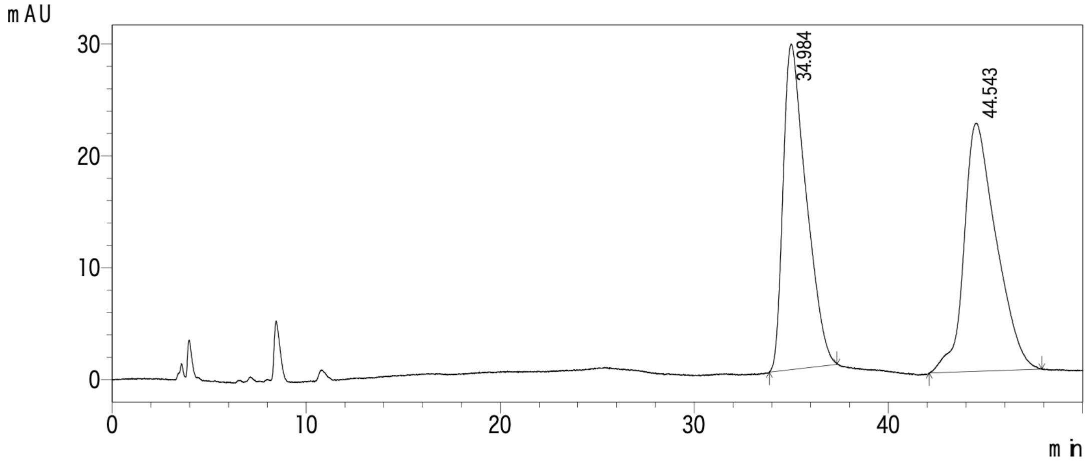

PDA Ch1 210nm

Peak No.	RT (min)	Area	Height	% Area
1	34.984	2397820	29123	48.366
2	44.543	2559880	22185	51.634
Total		4957699	51308	100.000

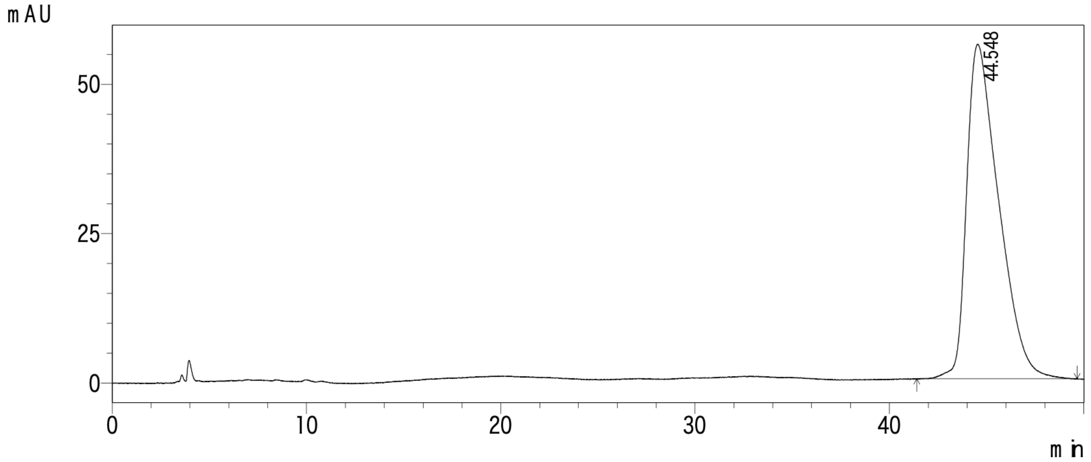

PDA Ch1 210nm

Peak No.	RT (min)	Area	Height	% Area
1	44.548	6423436	55970	100.000
Total		6423436	55970	100.000

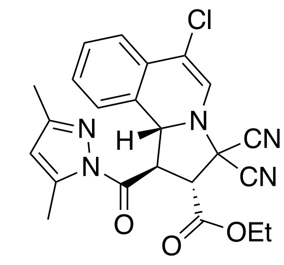

7

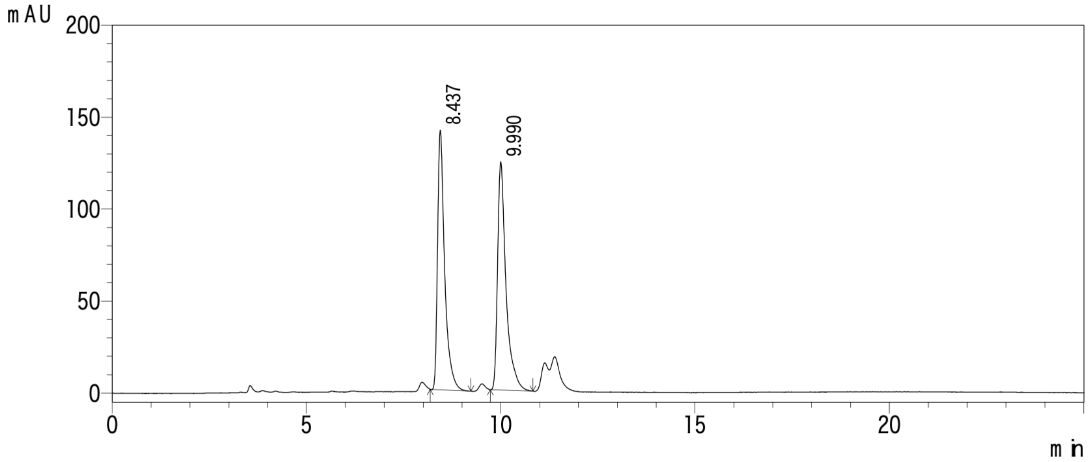

PDA Ch1 210nm

Peak No.	RT (min)	Area	Height	% Area
1	8.437	1797892	141142	49.104
2	9.990	1863478	124144	50.896
Total		3661370	265286	100.000

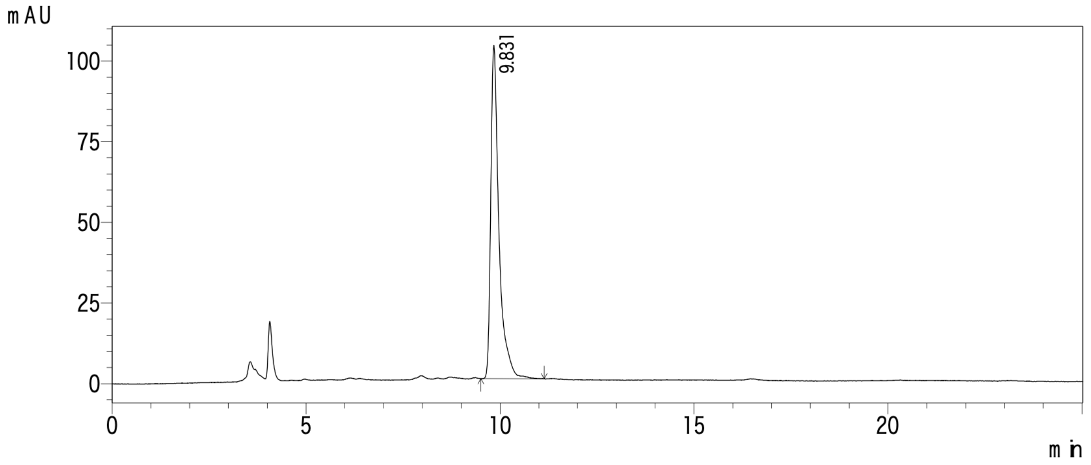

PDA Ch1 210nm

Peak No.	RT (min)	Area	Height	% Area
1	9.831	1521802	103360	100.000
Total		1521802	103360	100.000

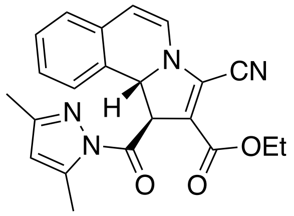

8

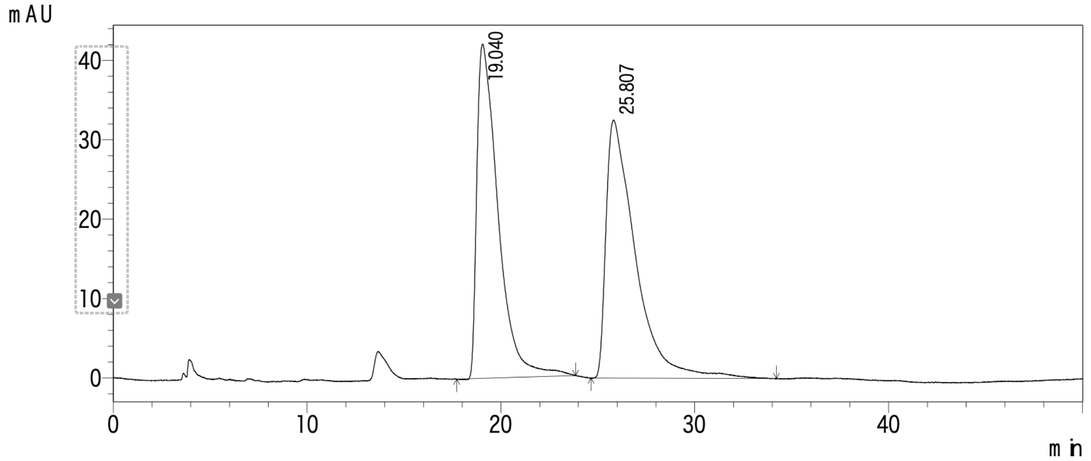

PDA Ch1 210nm

Peak No.	RT (min)	Area	Height	%Area
1	19.040	3199498	42103	48.436
2	25.807	3406090	32514	51.564
Total		6605588	74616	100.000

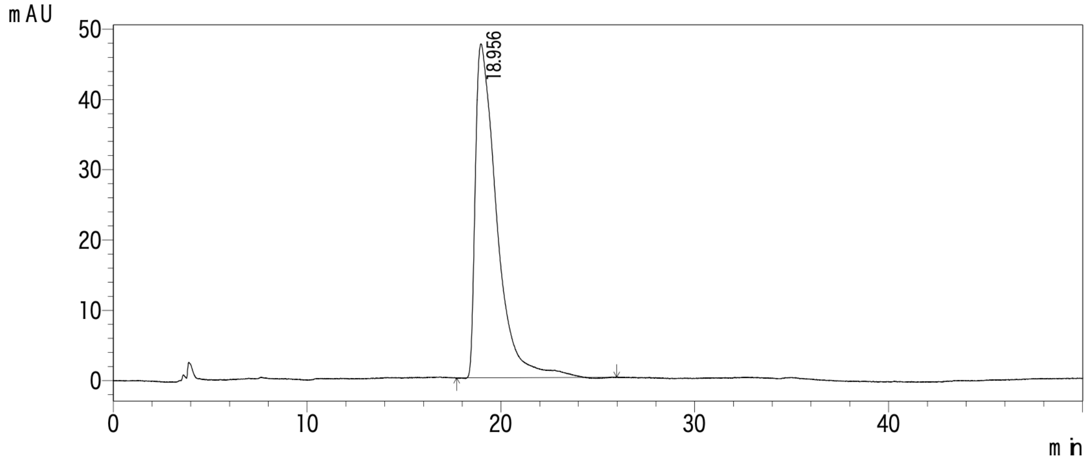

PDA Ch1 210nm

Peak No.	RT (min)	Area	Height	%Area
1	18.956	3620323	47535	100.000
Total		3620323	47535	100.000

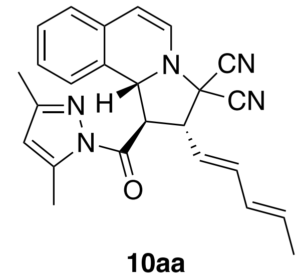

10aa

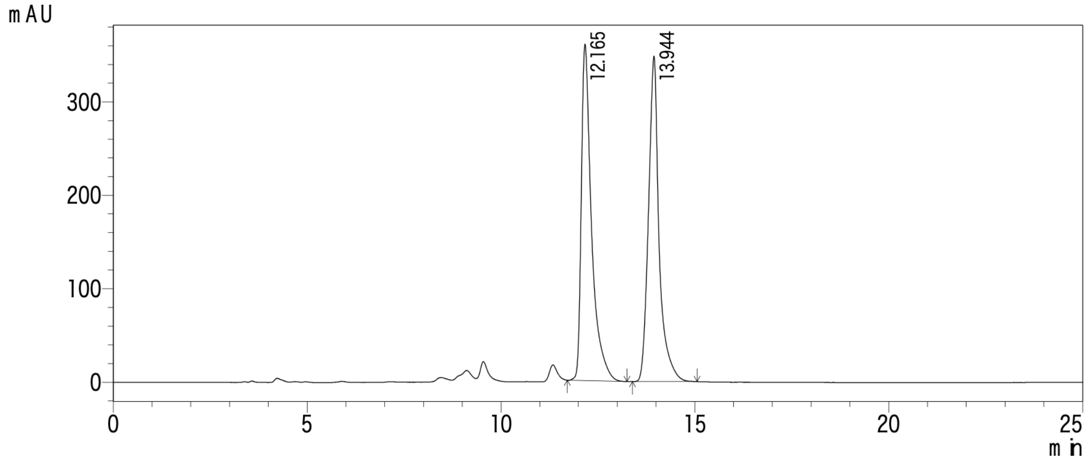

PDA Ch1 210nm

Peak No.	RT (min)	Area	Height	% Area
1	12.165	6599670	360162	50.016
2	13.944	6595524	348259	49.984
Total		13195194	708421	100.000

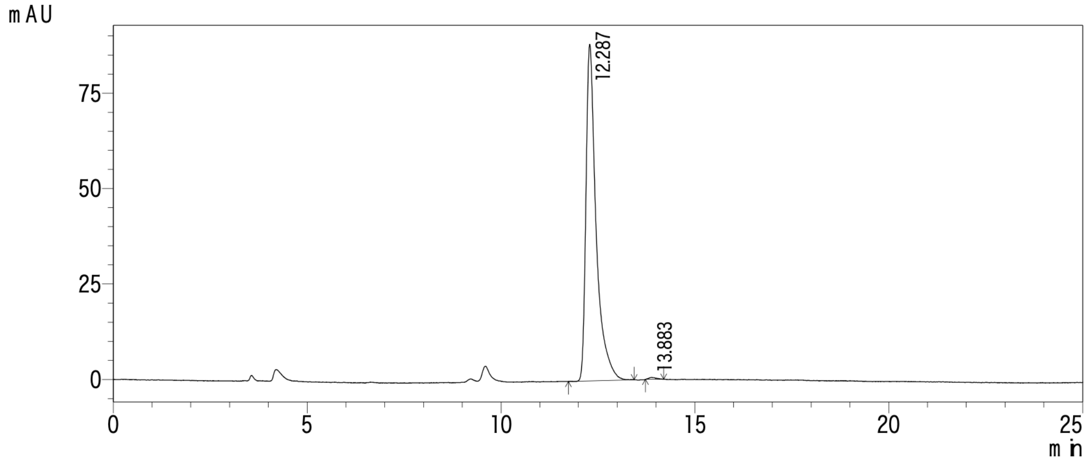

PDA Ch1 210nm

Peak No.	RT (min)	Area	Height	% Area
1	12.287	1603065	88172	99.578
2	13.883	6788	505	0.422
Total		1609853	88676	100.000

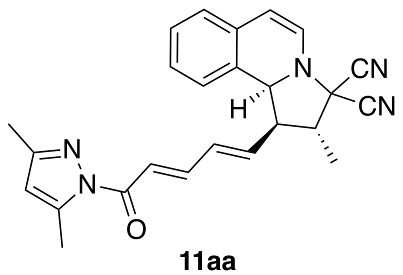

11aa

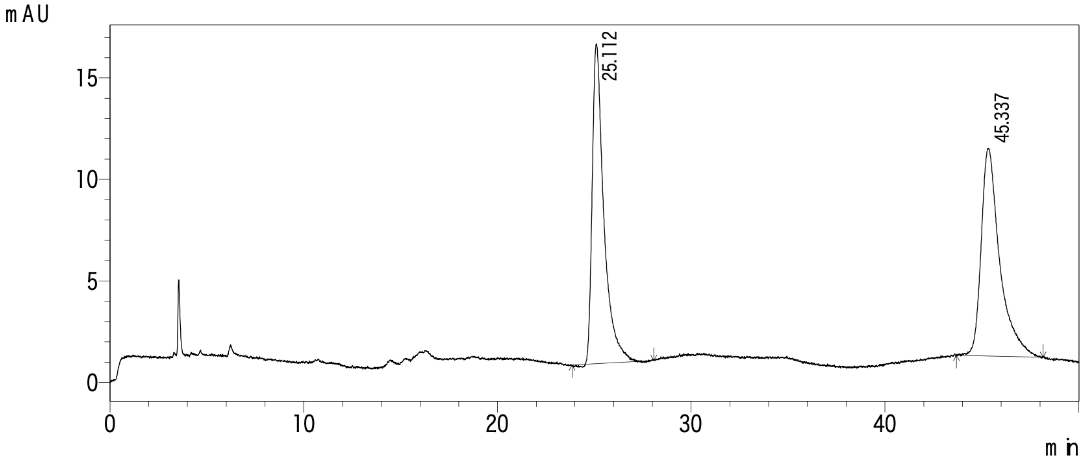

PDA Ch1 210nm

Peak No.	RT (min)	Area	Height	%Area
1	25.112	655406	15755	49.034
2	45.337	681227	10222	50.966
Total		1336633	25978	100.000

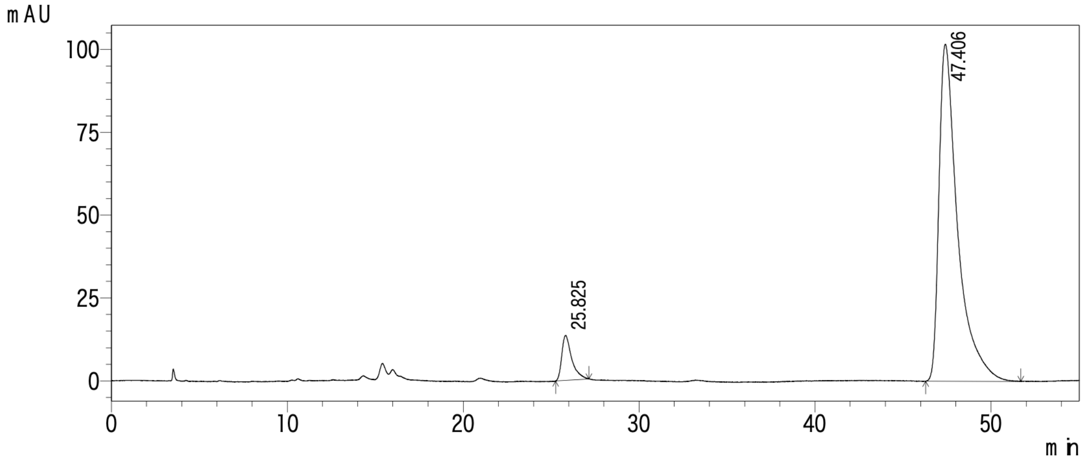

PDA Ch1 210nm

Peak No.	RT (min)	Area	Height	%Area
1	25.825	532181	13552	6.613
2	47.406	7515079	101794	93.387
Total		8047259	115346	100.000

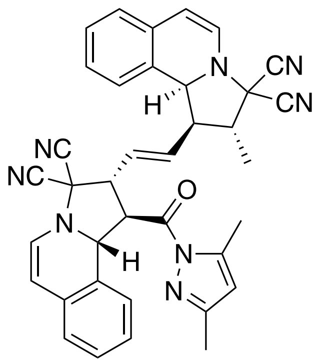

12aa

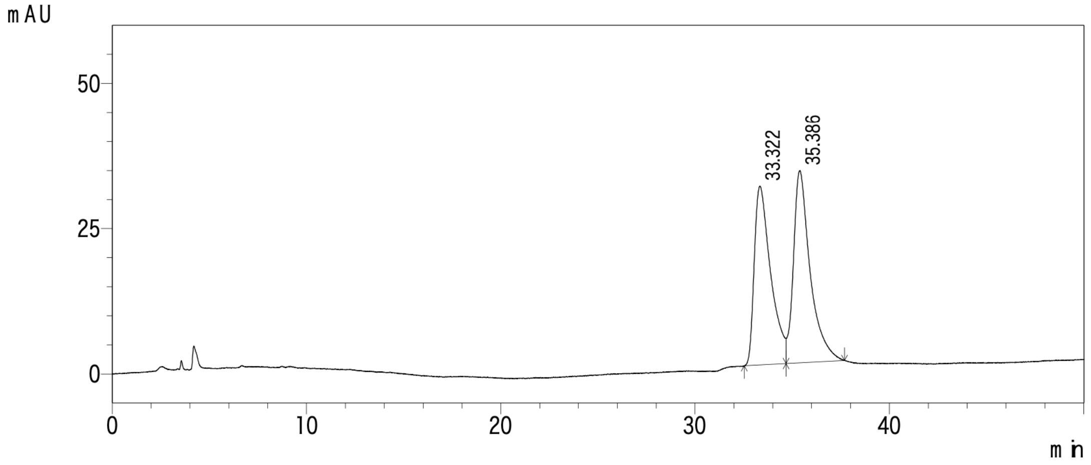

PDA Ch1 210nm

Peak No.	RT (min)	Area	Height	% Area
1	33.322	1812397	30762	47.739
2	35.386	1984047	33088	52.261
Total		3796445	63851	100.000

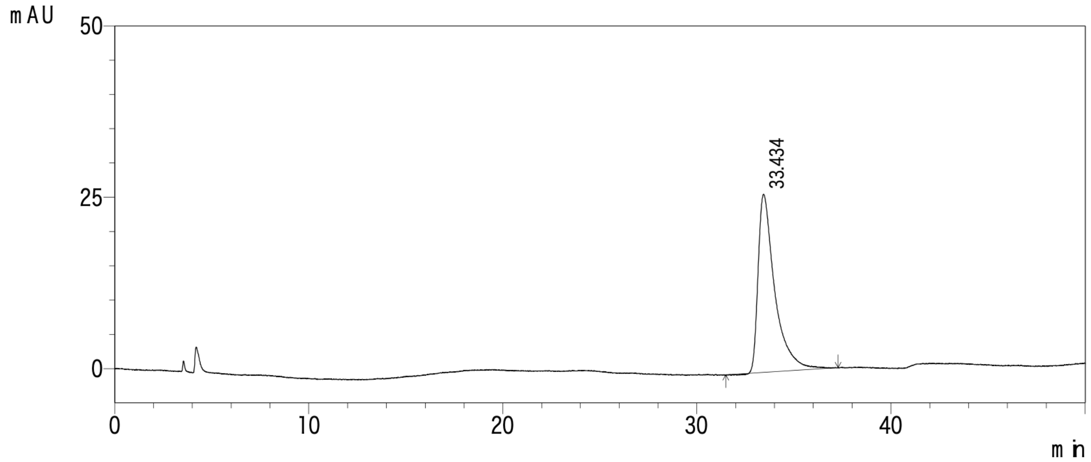

PDA Ch1 210nm

Peak No.	RT (min)	Area	Height	% Area
1	33.434	1556931	26009	100.000
Total		1556931	26009	100.000
